# Improving quality of care for pregnancy, perinatal and newborn care at district and sub-district public health facilities in three districts of Haryana, India: An Implementation study

**DOI:** 10.1371/journal.pone.0254781

**Published:** 2021-07-23

**Authors:** Manoja Kumar Das, Narendra Kumar Arora, Suresh Kumar Dalpath, Saket Kumar, Amneet P. Kumar, Abhishek Khanna, Ayushi Bhatnagar, Rajiv Bahl, Yasir Bin Nisar, Shamim Ahmad Qazi, Gulshan Kumar Arora, R. K. Dhankhad, Krishan Kumar, Ramesh Chander, Bhanwar Singh

**Affiliations:** 1 The INCLEN Trust International, New Delhi, India; 2 Department of Health and Family Welfare, Government of Haryana, Panchkula, Haryana, India; 3 Department of Maternal, Newborn, Child and Adolescent Health and Ageing, World Health Organization, Geneva, Switzerland; 4 Department of Health and Family Welfare, Office of Chief Medical Officer and Civil Surgeon (Faridabad), Government of Haryana, Faridabad, Haryana, India; 5 Department of Health and Family Welfare, Office of Chief Medical Officer and Civil Surgeon, (Jhajjar), Government of Haryana, Jhajjar, Haryana, India; 6 Department of Health and Family Welfare, Office of Chief Medical Officer and Civil Surgeon (Rewari), Government of Haryana, Rewari, Haryana, India; University of Mississippi Medical Center, UNITED STATES

## Abstract

**Introduction:**

Improving quality of care (QoC) for childbirth and sick newborns is critical for maternal and neonatal mortality reduction. Information on the process and impact of quality improvement at district and sub-district hospitals in India is limited. This implementation research was prioritized by the Haryana State (India) to improve the QoC for maternal and newborn care at the busy hospitals in districts.

**Methods:**

This study at nine district and sub-district referral hospitals in three districts (Faridabad, Rewari and Jhajjar) during April 2017-March 2019 adopted pre-post, quasi-experimental study design and plan-do-study-act quality improvement method. During the six quarterly plan-do-study-act cycles, the facility and district quality improvement teams led the gap identification, solution planning and implementation with external facilitation. The external facilitators monitored and collected data on indicators related to maternal and newborn service availability, patient satisfaction, case record quality, provider’s knowledge and skills during the cycles. These indicators were compared between baseline (pre-intervention) and endline (post-intervention) cycles for documenting impact.

**Results:**

The interventions closed 50% of gaps identified, increased the number of deliveries (1562 to 1631 monthly), improved care of pregnant women in labour with hypertension (1.2% to 3.9%, p<0.01) and essential newborn care services at birth (achieved ≥90% at most facilities). Antenatal identification of high-risk pregnancies increased from 4.1% to 8.8% (p<0.01). Hand hygiene practices improved from 35.7% to 58.7% (p<0.01). The case record completeness improved from 66% to 87% (p<0.01). The time spent in antenatal clinics declined by 19–42 minutes (p<0.01). The pooled patient satisfaction scores improved from 82.5% to 95.5% (p<0.01). Key challenges included manpower shortage, staff transfers, leadership change and limited orientation for QoC.

**Conclusion:**

This multipronged quality improvement strategy improved the maternal and newborn services, case documentation and patient satisfaction at district and sub-district hospitals. The processes and lessons learned shall be useful for replicating and scaling up.

## Introduction

Between 2000 and 2017, neonatal mortality rate (NMR) by 47%, infant mortality rate (IMR) declined by 51%, and maternal mortality rate (MMR) by 59% in India, which were slower than expected [1–3]. The Sustainable Development Goals have set ambitious health-related targets for mothers, newborns, and children under the umbrella of Universal Health Coverage (UHC) by 2030 [4]. The World Health Organisation (WHO) envisages that ‘every pregnant woman and newborn will receive quality care throughout pregnancy, childbirth and the postnatal period’ under the umbrella of UHC, which is aligned with ‘Ending Preventable Maternal Mortality’ and the ‘Every Newborn Action Plan’[5–7]. Good quality of care (QoC) is key to achieving these goals [8]. Estimates project that improved QoC can annually save about 1,325,000 neonatal deaths, 531,000 stillbirths and 113,000 maternal deaths globally [9]. The QoC includes the provision of effective, efficient, safe care that is accessible, acceptable, patient-centered, equitable with patient satisfaction [10]. Quality improvement (QI) strategies attempt to close the know-do gap using scientific thinking and simultaneous health systems strengthening [11]. The QI interventions have used combinations of the six dimensions of the health system: service delivery, health workforce, information, medical products/vaccines/technologies, financing and leadership and governance [12].

India’s ‘National Health Mission’ investments in infrastructure, manpower, transport facility and cash benefit schemes have increased the institutional deliveries from 38.7% in 2005–06 to 78.9% in 2015–16, but with disproportionate improvements in NMR, IMR, and MMR [13,14]. Recognizing the importance, the Government of India is pushing for the QI efforts at public health facilities through the ‘Quality Assurance Framework’. This framework includes National Quality Assurance Standards for public health facilities with operational guidelines toolkits, quality certification, patient feedback system, standard treatment guidelines, training and capacity building [15]. To ensure perinatal QoC and reducing neonatal mortality, the *LaQshya* initiative for labour rooms and the ‘*India Newborn Action Plan’* are being implemented [16,17]. Additionally, the state governments are also pushing the QoC agenda for maternal, newborn and child health (MNCH) services improvement.

Most evidence available on QoC and QI is from the high-income countries with good health systems and is limited from the low and middle-income countries [9]. The evidence from India on QoC and health system improvement is emerging. In Uttar Pradesh (India), the ‘*Safe Childbirth Checklist*’ use at facilities improved the essential birth practices adherence, but without significant changes in the maternal and perinatal mortality and morbidity [18]. In two districts (Ambala and Yamuna Nagar) of Haryana (India), implementation research with the QI interventions increased the number of deliveries at the primary health centres (PHCs, n = 15) with a modest rise in six perinatal practices and no change or worsening in four practices [19].

In Haryana, the district hospitals and the first referral units (FRUs) conduct about 60–70% of the total deliveries (administrative unpublished data). Assessments have documented poor birth preparedness, hygiene and infection prevention, and intrapartum practices in the labour room, and newborn care practices at the district hospitals and FRUs in Haryana, despite the availability of the infrastructure, equipment and supplies [20]. Perinatal asphyxia (47%), sepsis (22%), and complications of low birth weight (17%) were the major causes of newborn mortality [21]. Without improving the QoC at these referral hospitals, the maternal and newborn health indicators are unlikely to change.

The Government of Haryana partnered with the WHO and implementing partners for implementation researches to improve the QoC for MNCH services and document the processes. Based on the interaction with the state program managers, district health administrators and available literature, the challenge identified were: (a) despite reasonable infrastructure, supplies, manpower availability, management guidelines at the district hospitals and FRUs in the districts the quality of routine and emergency obstetric and newborn care remain poor; and (b) there was a need for adoption of context-specific measures to improve the quality of obstetric and newborn services addressing the identified determinants, bottlenecks within the local reality and public health program framework. This implementation research focused on improving the QoC for mothers and newborns at the district hospitals and FRUs in three districts of Haryana state.

We hypothesized that the QI activities driven by the district and facility quality management (QM) teams with external facilitation would identify gaps, design and implement context-specific interventions, linked capacity building, regular review of the inputs and outcomes would improve the QoC for the pregnant women and newborns at these facilities. The specific aims of this implementation research were: (a) to design and implement context-specific interventions that improve the QoC including care at birth, emergency obstetric care, sick newborn care and antenatal care for high-risk pregnancies; (b) to develop local capacity for adopting QI processes targeted at the maternal and newborn care; and (c) to assess the feasibility, acceptability and sustainability of these QI interventions.

## Methods

### Context

This study was conducted during April 2017 and March 2019 in three districts of Haryana—Faridabad, Rewari and Jhajjar, including a district hospital and all FRUs (two each) in each district. The districts were selected in consultation with state officials based on the administrative data for MNCH services. The district hospitals and FRUs are the busiest places in the district and are expected to provide 24x7 obstetric and newborn care. Details of the Indian public health system are given in S1 Table [22–24]. The three district hospitals (Faridabad and Rewari 200 beds each and Jhajjar 100 beds) and one sub-district hospital (200 beds) in Jhajjar have facilities to provide comprehensive obstetric and sick newborn care services. The districts have good road connectivity and the referral hospitals are reachable within one hour (PHC to FRU: 30 minutes, FRU to district hospital: 30–45 minutes and PHC to district hospital: 60 minutes). For complicated cases, the patients are referred to the nearest tertiary care hospitals (30–80 km). In 2017–18, Haryana recorded 91% institutional deliveries [25]. For Haryana, the NMR and IMR (22 and 30 per 1,000 livebirths, respectively) and MMR (98 per 100,000 livebirths) are relatively high for the state economic status compared to the national figures (S2 Table) [1,3,26]. This study was planned and implemented with the active involvement of the state and district officials. In these selected health facilities, four areas [labour rooms, postnatal wards, antenatal clinics and sick newborn care units (SNCU)] were targeted.

### Design and intervention(s)

We adopted a pre-post, quasi-experimental study design with repeated observations and no independent comparison group. Mixed (qualitative and quantitative) data collection techniques were used. The quantitative data documented the changes in the indicators related to the facility level structures, processes and outcomes. The qualitative data attempted to capture the perceptions and practices about the QoC, the efforts for QI and outcomes. The mixed-method design aimed at integrating the data for triangulation and obtaining a comprehensive understanding of the QoC efforts and gains. Implementation followed plan-do-study-act (PDSA) continuous QI cycle approach [27,28], which guides the QI identification of the problem(s), solution (plan), implementation (do), documentation and analysis (study) and planning the next action (act). The QI interventions included four core activities: (a) rapid process improvement cycles; (b) mortality and morbidity surveillance; (c) audits for processes and documents; and (d) using data for action. The theory of change logic model and pathways of improvement in the different domains of QoC was developed. The QI strategies, means of verifications and theory of change have been published in the study protocol (29). The theory of change and logic model targeted eight domains of QoC with the flow of actions and interventions (S1 Fig) [29].

### Implementation

At the hospitals, QM teams were established with the administration, doctors/specialists, nurses, head nurses, pharmacists and other concerned providers as members (S3 Table). At the district level, the QM teams were led by district health administrators with representation from the facility QM teams. The state steering group supervised the implementation. The study was implemented simultaneously at all the facilities in four phases.

**Fig 1:**
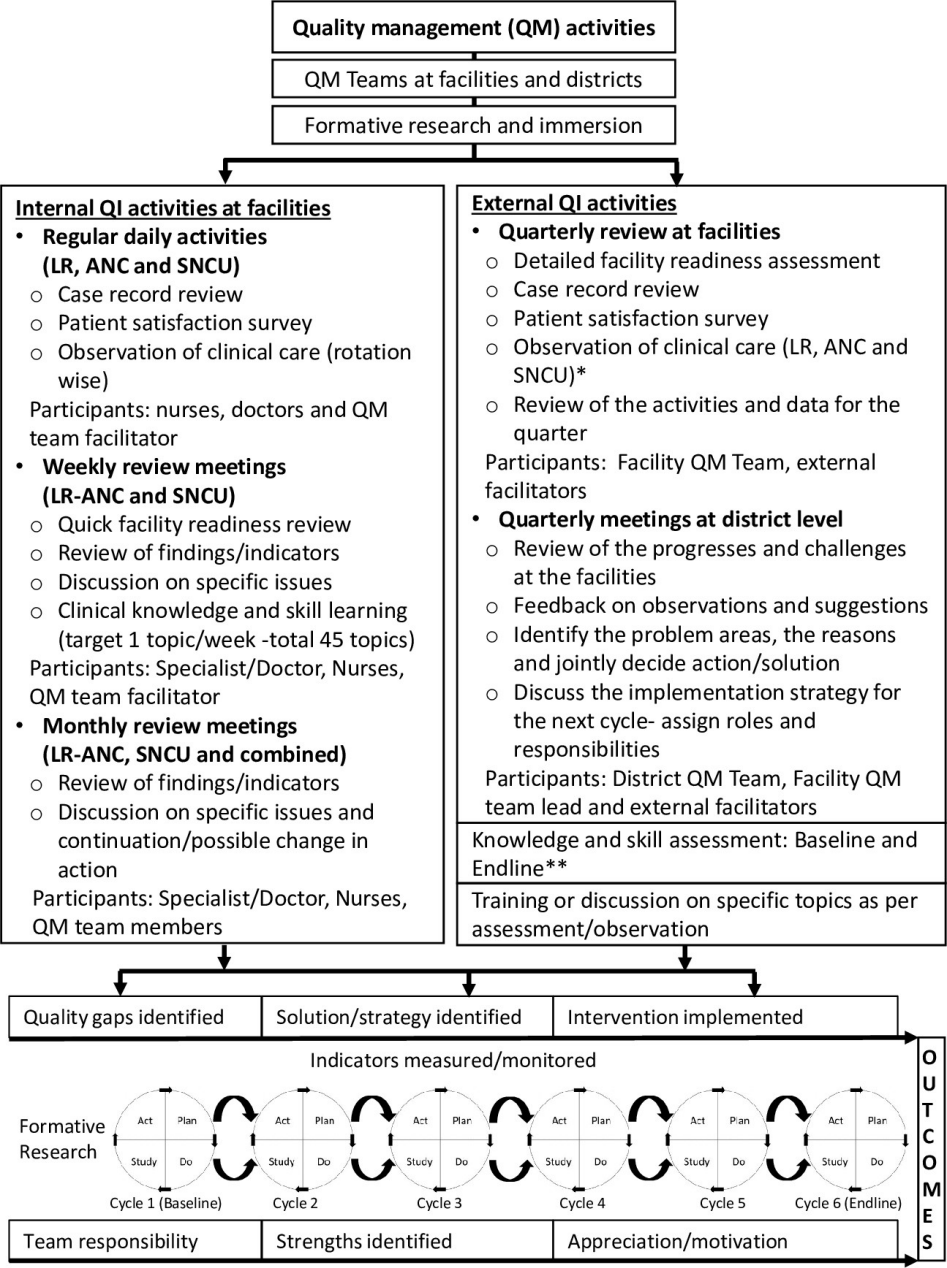
The quality management activities implemented during the project. Note: QI- Quality improvement and QM- Quality management. * Observations in labour room (LR)/antenatal clinic (ANC)/sick newborn care unit (SNCU) focused on the care for women in labour, antenatal care, essential and sick newborn care and hand hygiene at all places. ** The knowledge and skill assessments included respective care for women in labour and essential and sick newborn care in the labour room and SNCUs respectively.

(1) Phase 1- Formative research (duration: 3 months): For an in-depth understanding of the context, practices and processes, we documented the system ‘AS IT IS’ at these facilities targeting at (i) the process and functionality; (ii) communication, information and knowledge flow; and (iii) teams and accountability. Findings were shared with stakeholders for concurrence and obtaining buy-in.

(2) Phase 2- Participatory planning (duration: 3 months): The QM teams discussed and prioritized gaps for action. For the prioritized gaps, root-cause analyses were done using the ‘cause and effect diagram’ and ‘five why’ approaches followed by solution planning and measures of documentation [30,31].

(3) Phase 3- Implementation of the QI cycles (duration: 18 months): The identified solutions and strategies addressing the identified gaps were implemented through six quarterly PDSA cycles. Using the theory of change, the interventions and strategies focused on the infrastructural gaps, patient support facilities, clinical practices, capacity building, case documentation, supervision and monitoring, and communication [29]. During these cycles, weekly, monthly and quarterly meetings were held at the unit (labour room and SNCU), facility and district levels, respectively. During the weekly meetings, the QM teams discussed a topic on obstetrics and/or newborn care (total 45 topics; 20 maternal and 19 newborn and 6 common topics, adapted from the national protocols, S4 Table), and practiced the skills facilitated by the specialists (obstetrician/paediatrician). The minutes of the meeting were recorded and circulated to the QM team. External subject experts from the medical and nursing colleges and the technical support team conducted structured quarterly reviews including the infrastructure, equipment, supplies, human resources, care provider’s skills and knowledge, case records and patient satisfaction surveys. These experts reviewed the weekly and monthly meeting activities, collected data and interacted with the stakeholders to capture the feedback and challenges. The experts’ observations were shared with the facility and district QM teams and guided the incremental implementation for the next PDSA cycle. The frequencies, activities during these meetings and the participants are summarised in Fig 1.

Phase 4- Impact assessment (duration: 3 months): The primary outcomes focused on 20 indicators on the facility readiness, patient satisfaction, case record quality, knowledge and skills of care providers, including the 16 indicators on maternal and newborn care suggested by the WHO (S5 Table) [32]. Apart from these, changes in the time taken for key service availability through a client flow analysis, service delivery (number of deliveries, live births, stillbirths, referrals) and maternal and newborn mortality and perceptions, practices of the service providers and challenges experienced were also documented (secondary outcome indicators, S5 Table).

The QM teams were not provided with any financial or in-kind remuneration for participation in the QI activities. The nurses and doctors were provided with the training and skill development support for the QI processes, clinical care and case documentation as per the gaps identified.

### Data collection

The data collected included: facility assessment and service readiness (using WHO integrated tool to assess the facility level QoC in MNCH adapted for India) [33], case record review (for in-patients and antenatal) for quality of case documentation, client feedback on patient satisfaction, client flow analysis, knowledge and skill assessment for MNCH care and guides for in-depth interviews (IDIs) with stakeholders (data collection tools attached as Supplementary Document 2). The research team (three nurses and one public health professional per district) collected the quantitative data regularly using tablets with customized software. The research nurses didn’t participate in clinical care. The data were uploaded to a server in real-time without sharing with the facility QM teams. The supervisors checked the data for completeness and accuracy regularly. The knowledge and skill assessment was done by external paediatricians and obstetricians. The data on care at birth and hand hygiene practices were documented by the research team through direct observation. The service delivery data (for deliveries, admissions, antenatal attendance and deaths) were collected from the hospital administration. The investigators and external experts collected the qualitative data (in-depth interviews) with support from the research team. The interviews were recorded, transcribed, translated and entered into the computer. For participatory planning and prioritization, the root cause analysis or fishbone scheme was used and responsibility was assigned to the members of the QM team. The sampling and data collection for the quantitative and qualitative components progressed in parallel.

### Data management and analysis

The data from the server were downloaded periodically, cleaned and analyzed. The service readiness scores for the facilities were summarised as percentages based on the availability (of the items assessed using WHO integrated tool to assess the facility level QoC in MNCH adapted for India) under three broad heads: (a) general health infrastructure and services, (b) maternal health services and (c) newborn health services (33). The scores for the components under these heads were tracked to document the change in the service readiness status of the facilities. The completeness of documentation for different sections in the case records for the deliveries, sick newborns admitted and antenatal check-ups for pregnant women were summarised as percentages scores. The patient satisfaction for recently delivered women, sick newborns (from parents) and antenatal care were summarised as percentages scores. The times spent by pregnant women for antenatal care for registration, contact with nurse and doctor including the waiting time were documented and tracked to observe the changes. The knowledge and skill scores were summarised as percentages based on the responses and assessments by the assessors. The quantitative data were analyzed using descriptive statistics and expressed as proportions, means and standard deviation (SD) or medians with interquartile range (IQR). The figures between cycles and periods were compared either using chi-square or paired t-test, as appropriate. The changes in indicators and parameters between baseline (pre-intervention) and endline (post-intervention) and their statistical significance (p<0.05) have been presented in this manuscript. The maternal and newborn service delivery parameters for the intervention period (September 2017-March 2019) were compared with the pre-intervention period (April 2016-August 2017). The data were analyzed by STATA 15.0 (Stata Corporation, Texas, USA). The qualitative data were inductively analyzed following the steps: free listing, coding, axial coding and cross-tabulation. The integration of the quantitative and qualitative data was done at the analysis level. The quantitative and qualitative data were reviewed together for identifying the linkages and relationships for appropriate action at different levels. The WHO team was not involved in the implementation or data analysis.

### Ethical aspects

The protocol was reviewed and approved by institutional ethics committees at INCLEN (protocol ref: IIEC 025) and WHO (protocol ref: MCA00515). Written informed signed consent from the study participants was obtained using study information sheet and consent form in the local language before data collection and confidentiality ensured. Patients and the public were not involved in the design and conduct of the study. The feedback from patients was used for the interventions.

## Results

The quantity of data collected for various components is summarized in S6 Table. During the implementation, improvements across several domains of QoC were observed. The observations and results are presented under three components: changes in the processes, changes in the structures, and outcomes of the QI efforts.

### Changes in processes

#### Participation of the QM and facility teams in the QI processes

The health administration and the QM teams supported and actively participated in the implementation. Repeated discussions with the administration and push from external experts assisted in the renovation of one SNCU (which was pending despite repeated demands from the local team) and infrastructural changes at the other SNCUs and labour rooms. During the intervention period, out of the scheduled meetings, 95.0% (667/702) of the weekly, 88.8% (144/162) of the monthly and 84.7% (61/72) of the quarterly meetings were conducted. The nurses participated more often than doctors/specialists in the weekly meetings. At district and sub-district hospitals, at least one specialist attended 84.9% (265/312) of the weekly and all monthly and quarterly meetings. The SNCU nurses participated the most in the weekly self-learning activities and the specialists led the weekly training sessions for the nurses and other staff.

#### Changes in the quality of case record documentation

Significant improvements in case records and registers completeness and quality (correctness and clarity) for most components were observed at all the facilities (S10 Table). Improvements were observed in the labour rooms and postnatal wards for the maternal history (47.0% to 89.0%, p<0.05), baby details (40.0% to 85.6%, p<0.05), hospital course (54.9% to 81.1%, p<0.05) and discharge/referral advice (62.6% to 86.2%, p<0.05) at pooled level. For the antenatal care, improvements were observed for documentation of vitals (40.0% to 73.6%, p<0.05), examination (33.3% to 70.0%, p<0.05), investigations (64.2% to 92.2%, p<0.05), and counselling (21.0% to 57.3%) at pooled level. In the SNCUs, improvements were observed for the clinical history (45.3% to 79.3%, p<0.05), delivery information (61.0% to 84.3%, p<0.05), examination (6.5% to 86.8%, p<0.05) and discharge/referral advice (73.3% to 90.0%, p<0.05) at pooled level.

#### Changes in the time spent by patients for receiving care

At Faridabad district hospital, the waiting time for the first contact with nurses and total time spent in the clinic declined for both non-high-risk pregnant women (53 and 42 respectively, p<0.01) and high-risk pregnant women (56 and 19 minutes respectively, p<0.01) (S12 Table). At Rewari district hospital, the total time spent in the antenatal clinic reduced by 33 minutes and 80 minutes respectively for both non-high-risk and high-risk pregnant women (p<0.05 for all values), respectively, without much change in the time to contact with the nurse. At the Jhajjar district and sub-district hospitals, the antenatal clinics for non-high-risk and high-risk pregnant women were separate, thus not much change was observed. At some facilities including the FRUs, the time taken was longer during the endline cycle due to the reduced number of doctors in the clinics.

#### Changes in the knowledge and skill levels of the care providers

The pooled (nurses and doctors combined) changes in the knowledge and skills after at least 12 months for the districts are shown in S13 Table. Among the nurses in labour rooms, improvements related to delivery care (18.0%), newborn resuscitation (24.0%) and infection control (16.0%) were observed. Among the nurses in SNCUs, improvements related to newborn resuscitation (28%), temperature management (23.0%), clinical case management (17.0%), intravenous fluid and medication (21.0%) and supportive newborn care (14.0%) were observed.

### Changes in structures

#### Gaps identified and closed

During the quarterly assessments, gaps in 26.9% (1070/3972) of the items checked were identified at facilities under different sections and 50% of these were resolved (≥75% improvement) (pooled data in Table 1 and district-wise data in S7 Table). Except for several physical, infrastructural and human resource gaps, most of these gaps concerning processes, documentation, knowledge and skills and provision of care were resolved. The key gaps identified during the formative research are given in S8 Table. The gaps related to round-the-clock power supply, security, adequate manpower (doctors, nurses and support staff in the SNCUs) could not be addressed completely.

**Table 1:** The quality gaps identified and resolved during the intervention (all districts pooled).

Sl no	Nature of the quality gaps	Items checked, n	Gaps identified, n (%)*	Gaps resolved n (%)**
*1*	*General infrastructure and systems*			
1.1	Layout and accessibility^a^	99	20 (20)	9 (45)
1.2	Infrastructure (general)	225	54 (24)	26 (48)
1.3	Maternal care services	117	38 (32)	18 (47)
1.4	Newborn and child care	90	48 (53)	15 (31)
1.5	Shortage of staff	9	9 (100)	0 (0)
1.6	Information and records	99	17 (17)	8 (47)
1.7	Essential drugs and blood storage	18	10 (56)	2 (20)
1.8	Pharmacy and drugs	72	15 (21)	7 (47)
1.9	Laboratory services	153	40 (26)	27 (68)
1.10	Presence of guidelines and auditing	171	19 (11)	12 (63)
1.11	Supportive care	108	23 (21)	15 (65)
*2*	*Maternal health services*			
2.1	Infrastructure	171	66 (39)	32 (48)
2.2.	Equipment	207	52 (25)	36 (69)
2.3	Staff availability	9	8 (89)	0 (0)
2.4	Care in maternity wards	54	17 (31)	13 (76)
2.5	Case management	414	55 (13)	25 (45)
2.6	Monitoring and follow-up	216	43 (20)	31 (72)
2.7	Infection control	108	21 (19)	17 (81)
*3*	*Newborn health services*			
3.1	Infrastructure	297	121 (41)	49 (40)
3.2	Equipment	297	79 (27)	36 (46)
3.3	Staff availability	9	9 (100)	0 (0)
3.4	Case management	9	73 (48)	37 (51)
3.5	Monitoring and follow-up	63	22 (35)	13 (59)
3.6	Newborn care at birth	162	28 (17)	17 (61)
*4*	*Care provider’s knowledge and skills*			
4.1	Labour room	36	16 (43)	10 (62)
4.2	Sick newborn care unit	63	30 (48)	17 (57)
*5*	*Patient satisfaction level*			
5.1	Labour room	108	22 (20)	6 (28)
5.2	Antenatal clinic	81	13 (16)	3 (21)
5.3	Sick newborn care unit	90	13 (14)	3 (21)
*6*	*Clinical case record documentation*			
6.1	Labour room	72	23 (32)	7 (30)
6.2	Antenatal clinic	63	27 (43)	11 (42)
6.3	Sick newborn care unit	63	11 (17)	5 (43)
*7*	*Grand total*	3972	1070 (27)	530 (50)

Note: * The percentage estimated out of the total items checked

** The percentage estimated out of the gaps identified and considered resolved when ≥75% improvement was documented.

^**a**^ The layout and accessibility included the organisation of the units in the facility, signage and access to the labour room, antenatal clinic and sick newborn care unit.

#### Changes in the facility readiness

The service readiness status (scores) for the general, maternal and newborn health services at all the facilities in the districts improved (S9 Table). None of the FRUs except one in Jhajjar had a blood storage facility. CS was initially conducted in both FRUs in Faridabad but later stopped at FRU-2 as the obstetrician was transferred. One FRU in Jhajjar and none in Rewari conducted CS as no obstetrician was posted. At Jhajjar district hospital, no CS was conducted for some months as an obstetrician was unavailable. The sick newborn care was available at all facilities with the operationalization of the newborn stabilization units at five FRUs, although no dedicated staff was available. The Jhajjar district hospital SNCU was completely renovated. The kangaroo-mother care units were operationalized at all district hospitals. The reasons for no/minimal change for some components were due to the manpower shortage and persisting infrastructural gaps, which were beyond the scope of the hospital administration and required approval from higher authorities and funds. The decline in some components was due to the non-availability of the trained manpower due to relocation or strike of nurses.

### Outcomes of the QI efforts

#### Changes in the maternal and newborn care quality indicators

The changes in the key QI outcome indicators at the participating facilities related to maternal and newborn care are summarised in Table 2. The detection of pregnancy-induced hypertension (PIH) cases increased in all districts (Faridabad, 0.9% to 1.9%; Rewari, 1.0% to 5.0% and Jhajjar, 2.0% to 6.0%, p<0.01). In Faridabad district, the documentation of prolonged labour cases (0.5% to 4.1%, p<0.01), stillbirths (1.0% to 2.0%) and systemic infection (0% to 1.2%, p<0.01) increased, mostly due to improved classification and documentation. Significant improvements in newborn care at birth across the districts for skin-to-skin contact (39.0% to 50.0%, p<0.05), delayed cord clamping (10.0% to 29.0%, p<0.05) and breastfeeding initiation within the first hour of birth (16.0% to 40.0%, p<0.05) were observed. Identification of high-risk pregnancies during antenatal care increased from 5% to 7% and counselling and birth planning improved significantly from 33.0% to 40% (p<0.01) in all districts. A reduction (6.4% to 5.7%) in newborn deaths in Faridabad SNCU was observed and no deaths occurred in the SNCUs of the other two districts. Hand hygiene practices adherence improved in all districts by 15.0–45.0% (p<0.01). Case records completeness and accuracy improved significantly in all units; labour rooms and postnatal wards (23.0% to 36.0%, p<0.05), antenatal clinics (18.0% to 35.0%, p<0.05) and SNCUs (16.0% to 24.0%, p<0.05). The overall patient satisfaction improved for target beneficiaries; recently delivered women (9.0% to 20.0%, p<0.05), antenatal care (9.0% to 18.0%, p<0.05) and sick newborns (10.0% to 12.0%, p<0.05).

**Table 2:** Changes in the key outcome indicators with intervention in the districts.

Sl. no	Indicators /Period	Faridabad			Rewari			Jhajjar		
		Baseline cycle n/N (%)	Endline cycle n/N (%)	OR (95% CI)	Baseline cycle n/N (%)	Endline cycle n/N (%)	OR (95% CI)	Baseline cycle n/N (%)	Endline cycle n/N (%)	OR (95% CI)
1	*Obstetric care (Record review)*									
1.1	PIH cases detected^a^	30/3326 (0.9)	57/2982 (1.9)	2.1 (1.3, 3.3)†	14/1418 (1)	86/1725 (5)†	5.2 (2.9–9.2)†	32/1588 (2)	113/1891 (6)†	3 (2.0–4.6)†
1.2	Magnesium sulfate given- PIH cases^b^	30/30 (100)	57/57 (100)	-	14/14 (100)	86/86 (100)	-	32/32 (100)	113/113 (100)	-
1.3	Oxytocin given after delivery^c^	3102/3102 (100)	2626/2626 (100)	-	1185/1185 (100)	1615/1615 (100)	-	1425/1425 (100)	1568/1568 (100)	-
1.4	Prolonged labour cases^c^	16/3102 (0.5)	107/2626 (4.1)†	8.1 (4.8, 13.8)†	12/1185 (1)	0/1615 (0)	-	0/1425 (0)	0/1568 (0)	-
1.5	Still births^c^	30/3102 (1)	52/2626 (2)	2 (1.3–3.2)†	13/1185 (1.1)	18/1615 (1.1)	1 (0.5–2)	1/1425 (0.1)	0/1568 (0)	-
1.6	Severe systemic infection^c^	0/3102 (0)	32/2626 (1.2)†	-	0/1185 (0)	0/1615 (0)	-	0/1425 (0)	0/1568 (0)	-
1.7	Maternal deaths^a^	0/3326 (0)	0/2982 (0)	-	0/1418 (0)	0/1725 (0)	-	0/1588 (0)	0/1891 (0)	-
*2*	*Newborn care at birth (Observation)*									
2.1	Immediate drying^d^	165/165 (100)	188/188 (100)	-	161/161 (100)	232/232 (100)	-	323/323 (100)	347/347 (100)	-
2.2	Skin-to-skin contact^d^	81/165 (49)	179/188 (95)†	20 (9.8–43)†	72/161 (45)	220/232 (95)†	22 (11.7–43.8)†	165/323 (51)	312/347 (90)†	8.5 (5.6–12.8)†
2.3	Delayed cord clamping^d^	129/165 (78)	180/188 (96)†	6.2 (2.8–13.9)†	129/161 (80)	209/232 (90)*	2.5 (1.2–4)†	200/323 (62)	316/347 (91)†	6.2 (4–9.6)†
2.4	Breastfeeding initiation in first hour^d^	83/165 (50)	169/188 (90)†	8.7 (5–15.4)†	116/161 (72)	204/232 (88)†	2.8 (1.6–4.7)†	165/323 (51)	305/347 (88)†	6.9 (4.7–10.2)†
*3*	*Antenatal care (Observation) *									
3.1	High-risk pregnancies identified^e^	41/4093 (1)	531/6642 (8)†	8.6 (6.2–11.8)†	122/1356 (9)	324/2162 (15)†	1.78 (1.4–2.2)†	336/6728 (3)	776/9705 (8)†	1.6 (1.4–1.8)†
3.2	Counselling and birth planning^f^	46/152 (30)	100/151 (66)†	4.5 (2.7–7.3)†	10/98 (10)	119/277 (43)†	6.6 (3.3–13.3)†	29/130 (22)	123/199 (62)†	5.6 (3.4–9.3)†
*4*	*Sick newborn care (Record review)*									
4.1	Total newborn deaths^g^	42/664 (6.4)	38/663 (5.7)	0.9 (0.5–1.4)	0/280 (0)	0/289 (0)	-	0/558 (0)	0/338 (0)	-
4.2.1	Deaths- newborns weight >2500gms^g^	11/664 (1.6)	4/663 (0.6)	0.3 (0.1–1.1)*	0/280 (0)	0/289 (0)	-	0/558 (0)	0/338 (0)	-
4.2.2	Deaths-newborns weight <2500gms^g^	32/664 (4.8)	34/663 (5.1)	1.0 (0.6–1.7)	0/280 (0)	0/289 (0)	-	0/558 (0)	0/338 (0)	-
*5*	*Availability of services (Observation)*									
5.1	Bag, mask and oxygen availability^h^	12/12 (100)	12/12 (100)	-	12/12 (100)	12/12 (100)	-	12/12 (100)	12/12 (100)	-
5.2	Medicines availability^h^	12/12 (100)	12/12 (100)	-	12/12 (100)	12/12 (100)	-	12/12 (100)	12/12 (100)	-
*6*	*Infection control practices (Observation)*									
6.1	Soap and running water available^h^	12/12 (100)	12/12 (100)	-	12/12 (100)	12/12 (100)	-	12/12 (100)	12/12 (100)	-
6.2	Hand rub available^h^	0/12 (0)	11/12 (92)	-	0/12 (0)	12/12 (100)	-	0/12 (0)	12/12 (100)	-
6.3	Missed instances of hand hygiene^i^	1325/2366 (56)	442/1163 (38)†	0.48 (0.41–0.55)†	526/730 (72)	1224/ 2720 (45) †	0.31 (0.26–0.38)†	621/748 (83)	701/1844 (38)†	0.12 (0.1–0.15)†
*7*	*Case record documentation (Observations)*									
7.1	Women who delivered^k^	206/327 (63)	321/331 (97)*	18.8 (9.6–36.7)†	229/364 (63)	332/386 (86)†	3.6 (2.5–5.1)†	254/334 (76)	440/494 (89)†	2.5 (1.7–3.7)†
7.2	Women for antenatal check-up^k^	116/181 (64)	193/203 (95) †	10.8 (5.3–21.8)†	110/250 (44)	191/277 (69)†	2.8 (1.9–4)†	134/212 (63)	158/195 (81)*	2.4 (1.5–3.9)†
7.3	Sick newborns^k^	146/187 (78)	150/155 (97)*	8.2 (3.2–21.9)†	195/257 (76)	221/245 (90)†	2.9 (1.7–4.8)†	124/174 (71)	95/109 (87)*	2.7 (1.4–5.2)†
8	*Patient satisfaction (Interviews) *									
8.1	Women who delivered^j^	143/ 164 (87)	171/178 (96)†	3.5 (1.4–8.6)†	141/193 (73)	250/269 (93)†	4.8 (2.7–8.5)†	111/140 (79)	168/179 (94)†	3.9 (1.9–8.3)†
8.2	Women for antenatal check-up^j^	125/167 (75)	206/221 (93)†	4.6 (2.4–8.6)†	123/143 (86)	232/239 (97)†	5.3 (2.2–13)†	118/136 (87)	162/169 (96)†	3.5 (1.4–8.7)†
8.3	Mothers of sick newborns^j^	59/70 (84)	80/84 (95)†	3.2 (1.1–12.3)†	55/63 (87)	80/82 (97)	5.8 (1.1–28.4)*	47/54 (87)	75/76 (99)	11.1 (1.3–93.6)*

Notes: * Indicate the change is statistically significant (p<0.05)

† Indicate the change is statistically significant (p<0.01).

^a^ The denominator is the total number of pregnant women admitted to the hospitals.

^b^ The denominator is the total number of women with pregnancy-induced hypertension (PIH) admitted to the hospitals.

^c^ The denominator is the total number of pregnant women delivered at the hospitals.

^d^ The denominator is the total number of observations for newborn care at birth.

^e^ The denominator is the total number of pregnant women who attended antenatal clinics.

^f^ The denominator is the total number of observations for care in the antenatal clinics.

^g^ The denominator is the total number of sick newborns admitted to the hospitals.

^h^ The denominator is the total number of weekly/quarterly facility assessments conducted.

^i^ The denominator is the total number of observations for hand hygiene practices in the labour room and sick newborn care units at the hospitals. Missed instances of hand hygiene practices (cumulative instances of missing hand washing or alcohol hand rub or gloves use by the nurses and doctors), as observed.

^j^ The denominator is the total number of respondents who responded to the interview for patient satisfaction for different services.

^k^ The denominator is the total number of case records reviewed in the labour room, antenatal clinics and sick newborn care units at the hospitals.

#### Changes in the maternal and newborn service delivery

The changes in the maternal and newborn services delivered at these facilities between the intervention period (September 2017-March 2019) compared to the pre-intervention period (April 2016-August 2017) are shown in Table 3. The average monthly deliveries and antenatal clinic attendance increased in most of the facilities, mostly at the district and sub-district hospitals. At two FRUs, the monthly deliveries and antenatal attendance didn’t change due to the unavailability of specialists for few months. The detection of high-risk pregnancies increased by 2–4.5 fold in two districts (p<0.01). The marginal decline in stillbirths (by 0.2%-0.5% across the districts) and caesarean sections (CS) (by 0.9%-2.1% across the districts) were observed in the two districts. The rise in stillbirth in one district was due to revised labeing from intrauterine deaths. The average monthly sick newborns admission increased in the two districts (by 2.0%-14.0% across the districts). A reduction in newborn deaths (6.4% to 5.7%) was observed in Faridabad SNCU, primarily the deaths due to sepsis (which overlapped with the hand hygiene reinforcement period). At baseline, the labour room nurses didn’t resuscitate newborns and called the SNCU nurses. By the endline, the labour room nurses at several facilities resuscitated newborns successfully.

**Table 3:** Changes in the maternal and newborn service delivery indicators in the districts during the intervention period compared to the pre-intervention period.

District	Faridabad		Rewari		Jhajjar		Pooled	
Period	Pre-QI period	QI period	Pre-QI period	QI period	Pre-QI period	QI period	Pre-QI period	QI period
*Delivery services *								
Total deliveries (n)	13046	15712	6044	7205	7462	8067	26552	30984
Monthly deliveries (n)	767	827	355	379	439	425	1562	1631
Vaginal deliveries, % (95% CI)	80.8 (77.8–83.5)	81.9 (79–84.4)	86 (81.8–89.3)	78.7 (74.1–82.6)	88 (84.5–90.8)	89 (85.6–91.7)	84 (82.1–85.8)	83 (82–85.7)
Caesarean sections, % (95% CI)	18.9 (16.3–22)	17.8 (15.7–21)	11.2 (8.1–15)	14.5 (11.1–18.4)	13.1 (10.1–16.7)	11 (8.2–14.4)	15.6 (13.8–17.5)	15.5 (13.9–17.5)
Stillbirths, % (95% CI)	1.7 (0.0–2.8)	1.5 (0.7–2.5)	0.9 (0.1–2.4)	1.2 (0.4–3)	0.8 (0.2–2.3)	0.3 (0–1.3)	1.3 (0.7–1.9)	1.1 (0.6–1.7)
Pregnant women referred, % (95% CI)	9.5 (7.5–11.8)	8.6 (6.7–10.7)	17.6 (13.7–21.8)	13 (9.7–16.7)	10 (7.3-4-13.2)	16 (12.6–19.8)	11.3 (9.8–13)	11.4 (10–13.1)
*Antenatal services*								
Total antenatal clinic attendance (n)	30532	22720	13698	10680	24674	22342	55742	68904
Monthly antenatal clinic attendance (n)	1336	1607	628	721	1314	1299	3279	3627
Proportion of high-risk pregnancies, % (95% CI)	2 (1.3–2.9)	9 (7.6–10.5)†	14 (11.4–17)	8 (6.1–10.2)	5 (3.9–6.3)	10 (8.4–11.7)†	5 (4.7–6.3)	9 (8.2–10.1)†
*Newborn services*								
Total admissions (n)	3123	3567	1704	1590	1741	2197	6568	7354
Admissions per month (n)	184	188	100	84	102	116	386	387
Discharged, % (95% CI)	74 (66.9–80)	78.2 (71.6–83.8)	78 (68.6–85.6)	74 (63–82.8)	84 (75.7–90.7)	82 (73.6–88.4)	77 (72.4–81)	78 (73.6–82)
Referred/LAMA, % (95% CI)	20 (14.6–26.6)	17 (11.9–23.1)	22 (14.3–31.4)	26 (17.2–37)	16 (9.2–24.2)	16.5 (10.1–24.4)	20 (16–24.3)	19 (15.3–23.4)
Deaths, % (95% CI)	6.4 (3.4–11.1)	5.7 (2.9–10.2)	0	0	0	0	2.9 (1.4–5)	2.7 (1.2–4.7)
Inborn newborns, % (95% CI)	47 (39.3–54.2)	48 (40–55.2)	48 (37.9–58.2)	58 (47–69)	61 (50.6–70.3)	61.2 (51.7–70)	51 (45.9–56.1)	55 (49.9–60)
Newborn with birth weight <2500gms, % (95% CI)	43 (35.7–50.4)	52 (44.7–59.4)	64 (52.8–73.3)	45 (34.3–56.5)	68 (57.6–76.6)	65 (55.2–73.3)	51 (45.9–56.1)	54 (48.9–59)

Note: * Indicate the change is statistically significant (p<0.05)

† Indicate the change is statistically significant (p<0.01).

Pre-QI period: April 2016- August 2017 (prior to the quality improvement activities); QI period: September 2017- March 2019 (the period of quality improvement activities); LAMA: Left against medical advice.

#### Changes in the patient satisfaction

Significant improvements in pooled satisfaction levels among recently delivered women (79.0% to 94.2%, p<0.05), pregnant women attending antenatal clinics (84.2 to 96.6%, p<0.05) and mothers of sick newborns (86.0%-97.3%, p<0.05) were observed (S11 Table). Among the recently delivered women, improvements were observed for availability of nurses and doctors (74.0%-94.6%, p<0.05), cleanliness of the wards and toilets (64.0%-88.6%, p<0.05), care after delivery (79.8 to 93.3%, p<0.05), counselling at discharge (79.3% to 96.8%, p<0.05), diet services (68.0% to 86.0%), and overall comfort (79.0% to 93.6%, p<0.05), at pooled level. For the antenatal clinics, improvements were observed for toilets cleanliness (59.7% to 94.2%, p<0.05), reduction in waiting time (71.2% to 86.0%, p<0.05), and general cleanliness of the waiting area (81.0% to 94.2%, p<0.05), at the pooled level. For the SNCUs, improvements were documented for cleanliness (70.8% to 95.3%, p<0.05), appropriate space for the mothers/caretakers (78.5% to 93.85%, p<0.05) and signage and accessibility to the wards (79.5% to 95.5%, p<0.05), at the pooled level. At few facilities small or no changes in some components were due to the manpower-related challenges including transfers, relocations and strikes.

### Findings from the qualitative assessments

#### Understanding of QoC

At baseline, the functionaries had a limited understanding of QoC and the components. They considered QoC as availability of medicines, right medication, appropriate treatment, infection prevention, better facilities and cleanliness. The items like case documentation, essential physical facilities, effective communication and counseing and patient satisfaction feedback were not considered as part of the QoC. The review of protocol adherence, local efforts for continued education for knowledge and skill retention and action based on data were not practiced. Implementation of the QI processes assisted the functionaries at all levels to understand the components of the QoC and the processes.

*“Quality of care means patients should get prompt care, all medicines and equipment should be available.” (Specialist Medical Officer, District Hospital, Baseline cycle)*

*“According to me, quality of care means to provide good facility along with proper treatment and care to the patient”                (Nurse, FRU Hospital, Baseline cycle)*

*“Quality of care means appropriate treatment, good records and monitoring, good hygiene, sanitation, and most important thing is patient satisfaction.”*

                            *(Medical Officer, FRU Hospital, Endline cycle)*

*“Quality is not only about medicines and treatment. It also includes clean surroundings, biomedical segregation according to the guidelines. Adequate healthcare providers are also needed for good quality of services.”*

                            *(Nurse-in-charge, District Hospital, Endline cycle)*

#### Key changes noticed

The functionaries noticed changes in the infrastructure maintenance, cleanliness and functionality, biomedical waste management, disinfection and infection prevention practices, record keeping, patient counseing and also case management, especially the high-risk cases. The nurses and doctors appreciated the training and weekly meetings for self- and facilitated learnings, which improved their practices. They perceived the importance of the case record documentation and periodic review of the cases and deviations.

*“There are many changes. Biomedical waste management has improved. Staff is more responsible than earlier. Cleanliness has improved. Infection control practices are now followed. Training sessions are being regularly conducted for the staff. Regular visits of the officers are being done on a routine basis*.

                *(Specialist Medical Officer, District Hospital, Endline cycle)*

*“Many changes have happened like cleanliness in the hospital, improvement in record maintenance, and solution of the infrastructure issues. The way of working has changed now and we are working in a better way now.            (Nurse, FRU Hospital, Endline cycle)*

*“Yes, improvement in SNCU cleaning and hand hygiene. More hand rubs are provided. Cleaning checklist and register for clothes for cleaning started. Training provided on hand washing and cleaning.”            (Support staff, Sub-district hospital, Endline cycle)*

#### Facilitation by the QM team and external project team members

The functionaries recognized the role of the QM team with local staff and their participation in the gap identification, developing action plans and tracking. They were appreciative of the external experts for their review and guidance. The roles of project staff in case record review, observations and regular feedback, facilitating the training and weekly sessions and facilitating the function of the QM team were highly appreciated. Some of the nurse in-charges and nurses anticipated that the project nurses would assist them in the clinical and documentation activities. The specialists in SNCU and the labour room were appreciative of the external expert’s feedback and constant engagement of the project coordinators for pushing the infrastructural gaps and manpower reorganization issues with the administration, which were pending for some time. These QM and QI processes assisted the facilities to meet the national and state assessments for QoC.

*“The QM team and project team were very helpful. Due to the constant presence of the project team, hand washing practices have been improved. The team found out the gaps and implemented the solutions, which were very helpful to us. Regular discussions on the gaps identified were held with the staff and they were motivated to perform better in future.”*

                *(Specialist Medical Officer, District Hospital, Endline cycle)*

“The QM team *and project team were very helpful to us*. *We were guided about maintaining records and the importance of record-keeping and timely upgrading*. *Infection control and hand washing practices were encouraged*. *Weekly training was also very helpful*.*”*

                    *(Nurse, District Hospital, Endline cycle)*

*“By observing the things on a day-to-day basis, giving training, monitoring the things and telling the correct way of doing helped us to work properly.”*

            *(Support staff, Sub-district Hospital, Endline cycle)*

#### Sustenance of the changes and practices

The functionaries were hopeful that the changes in the practices shall continue and the checklists and reviews shall be included in the routine practices. The majority of them were of opinion that a dedicated staff should be there to undertake the QoC related activities routinely. Some of the nurses were of opinion that the nurse-in-charge can undertake some of these activities. Some of the staff were not sure and apprehensive about the sustenance and who shall be responsible.

*“These improvements should be sustained. One dedicated person should be appointed to supervise all the quality things. Daily visit of the concerned person should be done.”*

                *(Specialist Medical Officer, District Hospital, Endline cycle)*

*“People should be sensitized about how and why we need to improve the quality of health services. The Quality Manager and Hospital Administration department should be given this charge to continue the process.*

                *(Specialist Medical Officer, District Hospital, Endline cycle)*

*“These processes should be continued. I am not sure who will do this. But you should talk to our seniors to carry on these activities on regular basis in the future.”*

                        *(Nurse, District Hospital, Endline cycle)*

*No one from the hospital will work on improving the quality. Some people or teams from outside will have to come for this purpose.”*

                *(Support staff, District Hospital, Endline cycle)*

#### Challenges experienced during implementation

The challenges experienced and strategies to address them are summarised in Table 4 The specialists and facility leaderships had apprehension regarding the QI improvement activities and external facilitation at the beginning of the project. They perceived the QI process as a review, audit and monitoring of their activities by external teams. While orientation, repeated dialogues and joint working mechanism reduced their apprehension, but for some obstetricians, it persisted longer. The blood storage facility at five FRUs could not be functionalized during the period. Due to the non-availability of the obstetricians or doctors trained for CS, limited emergency obstetric services could be offered at the FRUs. At one district hospital, when the regular obstetrician was on long leave, a private obstetrician was engaged on-call basis and caesarean sections were conducted, but far less than expected. Most of the manpower gaps were beyond the scope of the hospital administration, which prevented improvements in the service readiness indicators. The non-availability of the manpower (because of relocation or transfer of doctors and nurses and strikes of contractual nurses) led to a decline in patient satisfaction scores and a longer waiting time for antenatal contacts for brief periods at some facilities. At six facilities (all district hospitals and three FRUs) the leadership changed and three facilities experienced the change more than once. The district health administrator changed in all districts and more than once in two districts. The specialists at three facilities and nurses at most of the facilities were transferred or rotated. Several nurses posted in labour rooms and SNCUs were contractual staff with lesser pay than the regular staff and were rotated within the hospital, which affected the performance and service delivery. During the study, two episodes of strikes by these contractual nurses were observed, which affected the service delivery.

**Table 4:** Challenges faced, strategies adopted and outcomes achieved during the implementation of quality improvement activities.

Challenge specification	Strategies adopted	Outcomes
*Maternal care related issues*		
• Hesitation and apprehension of specialists for the quality improvement effort • Concerns for various components	• Orientation of the health functionaries on various aspects of quality of care • Repeated dialogue and explanation about the activities and support from the quality implementation team	• Minimised the apprehensions of most of the doctors and nurses • Some apprehension of specialists persisted
• Low skills in resuscitation	• Orientation of the staffs regularly on resuscitation by technical experts and weekly self-learning	• Resuscitation skill and practice improved
• Participation in the weekly meetings and self-learning	• Flexibility in day and timing for the conduct of the review and self-learning meetings according to the availability of staffs	• 95% weekly meetings held • Participation of staffs improved
• Non-availability of the obstetrician at some FRUs from all three districts and for some months at one district hospital	• The district health administration had been informing the state about the manpower challenge. No short term specialist could be hired at the FRUs. • A contractual arrangement was made with a private obstetrician to provide on-call service for CS.	• No change was observed at the FRUs. • Some CS deliveries were conducted at the district hospital.
*Newborn care related issues*		
• Infrastructural challenges	• Repeated persuasion and engagement with the district administration for addressing the procurement, repair and renovation requests	• SNCU in one district renovated • Renovations and repairs done • KMC facilities operationalised
• Nurses availability	• Nurses were relocated from other areas and new recruitments	• Availability of nurses improved
• Low skill and knowledge and learning opportunity	• Orientation of the staffs regularly on critical aspects by technical experts and weekly self-learning with the availability of the protocols	• Improved knowledge and skills of the nurses in the labour room and SNCUs
• Non-availability of specialist and doctors for round the clock service at SNCU	• Discussion with the specialists for an evening round and on-call availability	• Evening rounds by specialists initiated • Doctors at one facility engaged
*Generic issues*		
• Limited understanding of the quality of care among the service providers and administrators	• Orientation of the staffs and administrators about the quality of care and its components • Repeated dialogue and feedback using the assessment results • The project team assisted in the general quality improvement process for the hospitals	• Improved in the understanding of the quality and activities • The leadership changes required repeat discussions
• Acceptance of the Quality Management team or Technical Support Unit	• Repeated meetings with the teams from various units • Non-interference by the project team in the clinical care • Supported in improving the documentation and facilitated discussion with the administration for approvals • Participation of the staffs in the identification of the problems and solutions further improved confidence and trust.	• A better understanding of the issues and challenges • The project team gained confidence and support from the various staffs
• Manpower shortage and engagement of contractual staffs • Shifting and relocation of staffs	• Identification of the areas and persons for possible relocation and posting in SNCU and Labour room • Discussion with the hospital and district administration	• Relocation of the nurses and staffs for SNCU and labour room • Initiation for new recruitments
• The conflict between health service staff and NHM staffs	• The challenges were beyond the scope of the Quality Management team or Technical Support Unit	• Could not achieve much change
*Administrative issues*		
• Pending requests from the SNCU and labour room related to infrastructure, equipment, staff, medication and other • Time taken and procedure for approval of expenses and payments	• The project team pursued with the administration and finance team at the district and facility level for action on the requests • The project team assisted in preparing the request and proposals for various gaps identified • Review meetings were used as a platform for addressing various issues and challenges	• Infrastructural gaps including repairs and renovations and small procurements
• Frequent changes in the administrative leadership • Strikes by the contractual staffs	• The challenges were beyond the scope of the Quality Management team or Technical Support Unit	• Could not achieve much change

Note: SNCU: Sick newborn care unit; NHM: National Health Mission; KMC: Kangaroo mother care; CS: Caesarean section.

## Discussion

The quality improvement intervention demonstrated improvements in the service readiness, especially the labour rooms and SNCUs across the facilities, except for the manpower, availability of operation facility, and blood bank at the FRUs. The quality and completeness of case records for deliveries, sick newborns and pregnant women improved significantly at all facilities. The patient satisfaction levels improved significantly for all components assessed, especially for the overall cleanliness and functional toilets, dietary provisions, and counseing at discharge. Significant improvements were observed at all facilities for care at birth, skin-to-skin contact and breastfeeding initiation. Reductions in the waiting time for antenatal check-ups were observed, especially for the high-risk pregnancies at some district hospitals. There were improvements in the knowledge and skill status of the nurses for delivery and newborn care through training and self-learning opportunities. Overall, the number of deliveries, antenatal clinic attendance and detection of high-risk pregnancies at the facilities were increased during the intervention period compared to the pre-intervention period. The sick newborns’ admissions increased for two SNCUs and the proportion of newborn deaths at one SNCU declined.

During the study period, half of the gaps identified under various domains of QoC were closed. Most of these were related to the infrastructure and processes, which were feasible for the local administration. The pending gaps required actions at a higher level, budget and motivation of the officials to pursue with the higher administration. The understanding of the hospital staff across all levels about QoC improved during the life of the project. The experiences of the functionaries were overall positive, which corroborated with the changes observed in the processes, structures and outcomes. The processes related to the QoC, external project team support, weekly training linked to the meetings, constant feedback and participation of the local members in the QM teams throughout were perceived positively. The QI improvement processes were acceptable to the healthcare functionaries (doctors, nurses and other staffs), facility and district management. The implementation of QI processes was feasible with regular adaptation based on the data and feedback, with support from the technical support and facilitation by the project team. The sustainability of the QI processes and practices after the withdrawal of the project team support is yet to be seen. The stakeholders had mixed reactions regarding the sustenance of the efforts without external facilitation and monitoring. Low level of motivation for improving the QoC and maintaining the improvements achieved and accountability among some service providers and leadership and contextual influences make the health system and the facilities vulnerable to slip into the pre-intervention phase again. Effective implementation and sustainability of QI interventions require regular review, identification of gaps, addressing them with high motivation and skills, supportive environment and administrative facilitation.

Several solutions need customization according to local context and thus the QI interventions and processes cannot be prescriptive. To scale up QI efforts, guidelines are needed, but local capacity building and motivation for adopting these processes and tracking the indicators are critical to identifying the gaps and design context-specific solutions and monitor. Intensive external technical and/or financial support can make a visible impact, but sustainability is a challenge. The government’s present QI (LaQshya) initiative for labour rooms focuses primarily on the structural aspects with limited inclusion of functional and client experience aspects. The ongoing QI efforts can be leveraged for furthering the QI efforts, especially in busy hospitals. For scaling up at the state level, technical support mechanisms for the districts are to be coupled with capacity building, monitoring and analysis. For impact on the maternal and neonatal indicators, efforts at facilities across all levels with a continuum of care linked to the community are needed.

Facility service readiness assessment is considered important for QoC, but the available tools capture the components variably with limited documentation for the outcomes [34]. A study in Bangladesh, Ghana and Tanzania documented the feasibility of using a tool for assessment of the three domains including clinical care, patient rights and crosscutting components [35]. The WHO integrated tool to assess the facility level QoC in MNCH adapted for India was used in this study for documenting the service readiness, which performed well. The suitability of a similar tool was documented in Malawi for quality improvement [33,36]. Clinical case assessment and management documentation is a critical part of QoC. Despite the use of formatted case sheets with checklists for maternal and newborn care in Haryana, several documentation gaps were observed. With the QI efforts, the completeness and quality of documentation increased to over 90% level for most and quite substantially for some components for delivery, antenatal and newborn care. Constant review and feedback by the project team were critical for achieving and maintaining the level of documentation.

Case record completeness for obstetric care has been a challenge across several countries. In a study across five countries (Bangladesh, Guinea, Mali, Niger, and Uganda), case records missed the key clinical information, partograph (0%-23.9%) or inappropriate charting (35.0%-98.0%) and missing fetal (40%) and maternal (10%) outcome information [37]. In Ethiopia, partographs were charted in 12% deliveries and not used for CS decisions [38]. Another study from Africa (Burkina Faso, Ghana and Tanzania) documented the childbirth record completeness in 64%-74% of cases with the newborn monitoring component being the lowest (29%-65%). For the antenatal cases, the overall completeness was about 82%-88% with lower quality for treatment (27%-78%) and laboratory findings (35%-43%) [39]. Another study from South African observed missing information for 54%-98% of antenatal cases [40]. We also documented lower (case record completeness and quality at baseline, which improved to 90% for most of the components with the QI efforts across the facilities and districts.

Consistent positive associations between patient experience, patient safety and clinical effectiveness for a wide range of diseases, settings, outcome measures and study designs have been reported [41]. Although patients’ satisfaction and experience are important measures of the QoC, there is no consensus in the literature about the measurement. Higher patient satisfaction with delivery and antenatal care was associated with lesser waiting time, privacy, better health providers’ attitude and communication, availability of equipment and facility, cleanliness, lower expenses [42–48]. In our study, significant improvement in satisfaction scores among all three categories of beneficiaries was observed, which paralleled with the changes in the scores for facility readiness, cleanliness and facilities, availability and counseling by the healthcare providers, reduction in waiting time. Presence of doctors for round-the-clock services in SNCUs and specialists (obstetrician and paediatrician) at the FRUs can improve obstetric and newborn care. Function and performance of the FRUs can be improved with provision of uninterrupted grid power supply, 24x7 security and regular kitchen facility for patients.

Rapid assessment, triaging, optimizing patient flow and time spent at the clinics and hospitals improve the performance, patient satisfaction and QoC. In our study, at two district hospitals the waiting time and total time spent by patients in the antenatal clinics decreased. The time to first contact in labour rooms was reduced in two district hospitals. This practice has been studied sporadically and documented improvements through altering the patient flow or organizing the facility for emergency room [49,50], general OPD [51], pain clinics [52] and daycare oncology clinics [53] have been reported. We couldn’t find any other study using client flow analysis for the pregnancy, delivery or newborn care for comparison.

The knowledge and skill status of the nurses improved with the modest retention after 15 months period through periodic self-learning and facilitated practice. The antenatal clinic attendance and the number of deliveries increased at most of the facilities during the intervention period. A marginal decline in the stillbirths and CS in two hospitals was observed. The pooled CS rates were 15.5% (11%-17.8%), which were comparable to the reports from India [54,55]. About 30% of the women in labour from lower-level facilities were referred to these facilities [19,56]. Thus higher CS rates were expected at these facilities. The CS at these facilities constituted 3%-5% of the total deliveries in the districts. The rise in newborn admissions and drop in referral and death (especially due to sepsis) were observed in our study. Use of ‘Safe Childbirth Checklist’ across 24 districts of Uttar Pradesh, India improved birth attendants’ adherence to practices at one year (intervention- 62% vs. comparison- 44%), but no difference in the maternal or neonatal outcomes [18] was reported. In Haryana, a study using multipronged technical support similar to our study in 15 PHCs from two districts (Ambala and Yamunanagar) increased the deliveries and improvement in six of twelve practices, but the neonatal mortality and stillbirth remained unchanged [19]. Our study results are similar to the QI studies from Haryana and Uttar Pradesh, which also observed the improvements in the processes and practices related to maternal and newborn services, but not in the mortality and morbidity outcomes [18,19].

Our study had some limitations and potential biases. First, the study was implemented at nine facilities from three districts and was not adequately powered to detect the change in mortality. Second, we used multiple processes and service delivery-related intermediary indicators. The changes in these process indicators were documented separately and the effect of multiple factors was not considered. Third, there was no control or comparison group. In the absence of a suitable control/comparison group of participants or facilities, we used the pre-intervention period for comparison. Fourth, we didn’t document the cost-benefit and cost-effectiveness of the interventions. Fifth, the QI steps were implemented with support from the external project team and the results may not be the same if implemented without it in a real setting. Sixth, the variations in the infrastructural, manpower and leadership factors across the districts and facilities could have affected the processes and outcomes. Finally, due to limited resources, we couldn’t plan for documenting the sustainability of the QI processes and outcomes after the withdrawal of the external monitoring and support.

Our study had several strengths. First, we implemented incremental PDSA QI cycles. Second, the use of mixed-method design provided an opportunity for corroborating and confirming the changes in the QoC components as observed in the processes, structures and outcomes. The quantitative and qualitative data allowed triangulation of the changes in the processes and outcomes with the perceptions of the various stakeholders and the potential sustenance. Third, we focused on multiple domains of QoC. Fourth, the QM teams were led by the functionaries from the districts and facilities, facilitated and supported by the external project team and technical experts. Finally, we involved multidisciplinary technical experts for documentation, facilitation and mentoring processes.

## Conclusion

The study demonstrated improvements in the facility readiness, clinical care at birth, maternal problems and during pregnancy, quality of case records, knowledge and skill status of service providers, service response time, patient satisfaction level and also the number of deliveries, ANC attendance at all the facilities and admission of sick newborns at the designated facilities. Several identified gaps could not be closed due to administrative, financial and procedural requirements. Despite the observed improvements, there is a risk of reversal of the achieved improvements in the practices and indicators in absence of push from the leadership, regular feedback to the service providers and motivation of the QM team. To sustain the gains in QoC, concerted efforts from the facility and district leaderships and facilitation from the state level are needed. For scaling up of QoC processes and interventions, dedicated teams at state and district levels are needed, who can implement the processes with technical support from the academic, research and implementation specialists. Thus, for achieving, monitoring and sustaining the QI gains and to keep the QM teams motivated, the use of appropriate tools along with a facilitating and functional health system at all levels is essential. The QI must be made an integral part of the health system processes and institutionalized across all levels of facilities with an appropriate technical and supportive supervision framework.

## Supporting information

S1 Checklist Good Reporting of A Mixed Methods Study (GRAMMS) checklist. (DOCX)

S2 Checklist Checklist—Standards for Quality Improvement Reporting Excellence (SQUIRE 2.0). (DOCX)

S3 Checklist. Completed StaRI checklist (DOCX)

S1 Fig Theory of change and logic model for the quality improvement in maternal and newborn care. (TIF)

S1 Table Organisation of the public health system in India and the maternal and newborn services. (PDF)

S2 Table Demographic and health profile of Haryana and study districts. (PDF)

S3 Table Quality management teams at the facilities in the districts and their compositions. (PDF)

S4 Table List of topics for self-learning and facilitated learning during weekly meetings. (PDF)

S5 Table Outcome indicators for the impact of quality improvement and frequency of data collection. (PDF)

S6 Table Data collected related to the quality improvement interventions in the study districts. (PDF)

S7 Table. The quality gaps identified and resolved during the intervention period for each district (PDF)

S8 Table Key quality gaps observed at the facilities in the districts during formative research phase. (PDF)

S9 Table Changes in the infrastructure, manpower and processes at the hospitals in the three districts. (PDF)

S10 Table Changes in the quality of case record documentation at the hospitals in the three districts. (PDF)

S11 Table Changes in the patient satisfaction status at the hospitals in the three districts. (PDF)

S12 Table Time spent (median and IQR in minutes) in minutes by pregnant women in antenatal clinics in Faridabad district. (PDF)

S13 Table Change in knowledge and skill status of the care providers in labour room and sick newborn care units. (PDF)

S1 File Quantitative data used for analysis. (XLSX)

S2 File Qualitative data used for analysis. (PDF)

S3 File Tools used for data collection. (PDF)

S1 Appendix Supplementary information. (DOCX)

S1 Dataset (RAR)

## References

[1] Registrar General of India. SRS Bulletin. Sample Registration System, 2017, Vol. 52, No.1 [Internet]. Registrar General of India, Government of India; 2917 [cited 2020 Mar 21]. Available from: http://censusindia.gov.in/vital_statistics/SRS_Bulletins/SRS_Bulletin-Rate-2017-_May_2019.pdf.

[2] UN Inter-agency Group for Child Mortality Estimation. Estimates Developed by the UN Inter-agency Group for Child Mortality Estimation (UNICEF, WHO, World Bank, UN DESA Population Division) [Internet]. [cited 2020 Mar 21]. Available from: https://data.worldbank.org/indicator/SH.DYN.NMRT?locations=IN.

[3] Registrar General of India. Special Bulletin on Maternal Mortality in India 2015–17, Sample Registration System November 2019 [Internet]. Registrar General of India, Government of India; 2019. [cited 2020 Mar 21] Available from: http://censusindia.gov.in/vital_statistics/SRS_Bulletins/MMR_Bulletin-2015-17.pdf.

[4] World Health Organisation. The goals within a goal: Health targets for SDG 3. SDG 3: Ensure healthy lives and promote wellbeing for all at all ages. [Internet]. [cited 2020 Apr 20]. Available from: https://www.who.int/sdg/targets/en/.

[5] World Health Organisation. Standards for improving quality of maternal and newborn care in health facilities [Internet]. World Heal Organisation, Geneva; 2016 [cited 2020 Apr 20]. Available from: https://apps.who.int/iris/bitstream/handle/10665/249155/9789241511216-eng.pdf?sequence=1.

[6] World Health Organization. Strategies toward ending preventable maternal mortality (EPMM) [Internet]. World Health Organization, Geneva; 2015 [cited 2020 Apr 20]. Available from: https://www.who.int/reproductivehealth/topics/maternal_perinatal/epmm/en/.

[7] World Health Organisation. Every Newborn: an action plan to end preventable deaths [Internet]. World Heal Organisation, Geneva; 2014 [cited 2020 Apr 20]. Available from: http://www.healthynewbornnetwork.org/hnn-content/uploads/Every_Newborn_Action_Plan-ENGLISH_updated_July2014.pdf.

[8] Tunçalp Ӧ., Were W, MacLennan C, Oladapo O, Gülmezoglu A, Bahl R, et al. Quality of care for pregnant women and newborns-the WHO vision. BJOG Int J Obstet Gynaecol. 2015;122(8):1045–9. doi: 10.1111/1471-0528.13451 25929823 PMC5029576

[9] Bhutta ZA, Salam RA, Lassi ZS, Austin A, Langer A. Approaches to improve Quality of Care (QoC) for women and newborns: conclusions, evidence gaps and research priorities. Reprod Health. 2014;11(S2):S5. doi: 10.1186/1742-4755-11-S2-S5 25208572 PMC4160923

[10] Nair M, Yoshida S, Lambrechts T, Boschi-Pinto C, Bose K, Mason EM, et al. Facilitators and barriers to quality of care in maternal, newborn and child health: a global situational analysis through metareview. BMJ Open. 2014;4(5):e004749. doi: 10.1136/bmjopen-2013-004749 24852300 PMC4039842

[11] Leatherman S, Ferris TG, Berwick D, Omaswa F, Crisp N. The role of quality improvement in strengthening health systems in developing countries. Int J Qual Health Care. 2010;22(4):237–43. doi: 10.1093/intqhc/mzq028 20543209

[12] Althabe F, Bergel E, Cafferata ML, Gibbons L, Ciapponi A, Alemán A, et al. Strategies for improving the quality of health care in maternal and child health in low- and middle-income countries: an overview of systematic reviews. Paediatr Perinat Epidemiol. 2008;22(s1):42–60. 18237352 10.1111/j.1365-3016.2007.00912.x

[13] International Institute for Population Sciences. State Fact Sheet-Haryana. National Family Health Survey -4 (2015–16) [Internet]. Government of India; 2017 [cited 2020 Apr 20]. Available from: http://rchiips.org/NFHS/pdf/NFHS4/HR_FactSheet.pdf.

[14] Lim SS, Dandona L, Hoisington JA, James SL, Hogan MC, Gakidou E. India’s Janani Suraksha Yojana, a conditional cash transfer programme to increase births in health facilities: an impact evaluation. The Lancet. 2010;375(9730):2009–23. doi: 10.1016/S0140-6736(10)60744-1 20569841

[15] National Health Systems Resource Centre. Quality Improvement-National Quality Framework for Public Health Facilities [Internet]. Quality Improvement. 2020 [cited 2020 Apr 20]. Available from: http://nhsrcindia.org/quality-improvement.

[16] National Health Mission. LAQSHYA- Labour room quality improvement initiative [Internet]. Ministry of Health & Family Welfare Government of India; 2017 [cited 2020 Apr 20]. Available from: http://nhsrcindia.org/sites/default/files/LaQshya-%20Labour%20Room%20Quality%20Improvement%20Initiative%20Guideline.pdf.

[17] National Health Mission. India Newborn Action Plan (INAP) [Internet]. Ministry of Health & Family Welfare Government of India; 2014 [cited 2020 Apr 20]. Available from: https://nhm.gov.in/index4.php?lang=1&level=0&linkid=153&lid=174.

[18] Semrau KEA, Hirschhorn LR, Marx Delaney M, Singh VP, Saurastri R, Sharma N, et al. Outcomes of a Coaching-Based WHO Safe Childbirth Checklist Program in India. N Engl J Med. 2017;377(24):2313–24. doi: 10.1056/NEJMoa1701075 29236628 PMC5672590

[19] Agarwal R, Chawla D, Sharma M, Nagaranjan S, Dalpath SK, Gupta R, et al. Improving quality of care during childbirth in primary health centres: a stepped-wedge cluster-randomised trial in India. BMJ Glob Health. 2018;3(5):e000907. doi: 10.1136/bmjgh-2018-000907 30364301 PMC6195146

[20] Maternal and Child Health Integrated Program (MCHIP). Strengthening Essential Newborn Care: Facility Readiness and Supportive Supervision in Haryana. [Internet]. Maternal and Child Health Integrated Program (MCHIP), USAID; 2014 [cited 2020 Apr 20]. Available from: http://pdf.usaid.gov/pdf_docs/PA00JZDJ.pdf.

[21] Neogi SB, Malhotra S, Zodpey S, and Mohan P. Assessment of Special Care Newborn Units in India. J Health Population. 2011;29(5):500–509. doi: 10.3329/jhpn.v29i5.8904 22106756 PMC3225112

[22] Department of Health and Family Welfare. Infrastructure, Health Department, Government of [Internet]. Government of Haryana; [cited 2020 Mar 28]. Available from: http://haryanahealth.nic.in/Infrastructure.html.

[23] Ministry of Health and Family Welfare, Government of India. Indian Public Health Standards (IPHS) for Sub-centres, Primary Health Centres (PHCs), Community Health Centres (CHCs), Sub-District and District Hospitals [Internet]. 2012. [cited 2020 Apr 2020]. Available from: https://nhm.gov.in/index1.php?lang=1&level=2&sublinkid=971&lid=154#:~:text=IPHS%20are%20a%20set%20of,especially%20for%20Non%2DCommunicable%20Diseases.

[24] Ministry of Health and Family Welfare. Ayushman Bharat. Comprehensive Primary Health Care through Health and Wellness Centers- Operational Guidelines. [Internet]. Government of India; 2018 [cited 2020 Apr 20]. Available from: https://ab-hwc.nhp.gov.in/download/document/45a4ab64b74ab124cfd853ec9a0127e4.pdf.

[25] Directorate of Information, Public Relations & Languages. The ‘Zero Home Delivery Campaign’ Launched In Haryana Has Resulted In An Increase In Institutional Deliveries To 91.1 Percent From 88 Percent In April 2017. [Internet]. Government of Haryana; 2017 [cited 2020 Mar 21]. Available from: https://www.prharyana.gov.in/en/the-zero-home-delivery-campaign-launched-in-haryana-has-resulted-in-an-increase-in-institutional.

[26] Department of Economic and Statistical Analysis, Haryana. Economic Survey of Haryana (2017–18) [Internet]. Government of Haryana; 2018 [cited 2020 Mar 28]. Available from: http://esaharyana.gov.in/Portals/0/ES%202017-18%20English.pdf.

[27] Davidoff F, Batalden P, Stevens D, Ogrinc G, Mooney S, for the SQUIRE development group. Publication guidelines for quality improvement in health care: evolution of the SQUIRE project. Qual Saf Health Care. 2008;17(Suppl 1):i3–9. 18836063 10.1136/qshc.2008.029066 PMC2773518

[28] Peters DH, Tran NT, Adam T, Alliance for Health Policy and Systems Research, World Health Organization, editors. Implementation research in health: a practical guide. Geneva, Switzerland: World Health Organization; 2013. [cited 2020 April 20]. Available from: https://www.who.int/alliance-hpsr/resources/implementationresearchguide/en/.

[29] Das MK, Arora NK, Dalpath S, Kumar S, Qazi SA, Bahl R. Improving quality of care for perinatal and newborn care at district and subdistrict hospitals in Haryana, India: Implementation research protocol. J Adv Nurs. 2018;74(12):2904–11. doi: 10.1111/jan.13791 29989201

[30] Harel Z, Silver SA, McQuillan RF, Weizman AV, Thomas A, Chertow GM, et al. How to Diagnose Solutions to a Quality of Care Problem. Clin J Am Soc Nephrol CJASN. 2016;11(5):901–7. doi: 10.2215/CJN.11481015 27016495 PMC4858489

[31] Pojasek RB. Asking “Why?” Five Times. Environ Qual Manag. 2000;10(1):79–84.

[32] World Heal Organisation. Consultation on Improving measurement of the quality of maternal, newborn and child care in health facilities. WHO and PMNCH December 9–11, 2013. World Health Organization, Geneva; 2014. [cited 2020 April 20]. Available from: https://apps.who.int/iris/bitstream/handle/10665/128206/9789241507417;jsessionid=0DC0D92B1EA4525DE048B50E7557ED79?sequence=1.

[33] World Health Organisation. Hospital care for mothers and newborn babies quality assessment and improvement tool [Internet]. World Heal Organisation, Regional Offices for Europe; 2014 [cited 2020 Mar 28]. Available from: https://www.euro.who.int/en/health-topics/Life-stages/maternal-and-newborn-health/publications/2014/hospital-care-for-mothers-and-newborn-babies-quality-assessment-and-improvement-tool.

[34] Brizuela V, Leslie HH, Sharma J, Langer A, Tunçalp Ö. Measuring quality of care for all women and newborns: how do we know if we are doing it right? A review of facility assessment tools. Lancet Glob Health. 2019;7(5):e624–32. doi: 10.1016/S2214-109X(19)30033-6 30898495

[35] Manu A, Arifeen S, Williams J, Mwasanya E, Zaka N, Plowman BA, et al. Assessment of facility readiness for implementing the WHO/UNICEF standards for improving quality of maternal and newborn care in health facilities–experiences from UNICEF’s implementation in three countries of South Asia and sub-Saharan Africa. BMC Health Serv Res. 2018;18(1):531–543. doi: 10.1186/s12913-018-3334-0 29986692 PMC6038273

[36] Smith H, Asfaw AG, Aung KM, Chikoti L, Mgawadere F, d’Aquino L, et al. Implementing the WHO integrated tool to assess quality of care for mothers, newborns and children: results and lessons learnt from five districts in Malawi. BMC Pregnancy Childbirth. 2017;17(1):271–281. doi: 10.1186/s12884-017-1461-y 28841850 PMC5572070

[37] Landry E, Pett C, Fiorentino R, Ruminjo J, Mattison C. Assessing the quality of record keeping for cesarean deliveries: results from a multicenter retrospective record review in five low-income countries. BMC Pregnancy Childbirth. 2014;14(1):139–149. doi: 10.1186/1471-2393-14-139 24726010 PMC4021100

[38] Fesseha N, Getachew A, Hiluf M, Gebrehiwot Y, Bailey P. A national review of cesarean delivery in Ethiopia. Int J Gynecol Obstet. 2011;115(1):106–11. doi: 10.1016/j.ijgo.2011.07.011 21872239

[39] Duysburgh E, Zhang W-H, Ye M, Williams A, Massawe S, Sié A, et al. Quality of antenatal and childbirth care in selected rural health facilities in Burkina Faso, Ghana and Tanzania: similar finding. Trop Med Int Health. 2013;18(5):534–47. doi: 10.1111/tmi.12076 23398053

[40] Patience NTS, Sibiya NM, Gwele NS. Evidence of application of the Basic Antenatal Care principles of good care and guidelines in pregnant women’s antenatal care records. Afr J Prim Health Care Fam Med. 2016 May 31;8(2):e1–6. doi: 10.4102/phcfm.v8i2.1016 27380852 PMC4913450

[41] Doyle C, Lennox L, Bell D. A systematic review of evidence on the links between patient experience and clinical safety and effectiveness. BMJ Open. 2013;3:e001570. doi: 10.1136/bmjopen-2012-001570 23293244 PMC3549241

[42] Dewana Z, Fikadu T, G/ Mariam A, Abdulahi M. Client perspective assessment of women’s satisfaction towards labour and delivery care service in public health facilities at Arba Minch town and the surrounding district, Gamo Gofa zone, south Ethiopia. Reprod Health. 2016 Feb 11;13:11. doi: 10.1186/s12978-016-0125-0 26867797 PMC4750206

[43] Kumsa A, Tura G, Nigusse A, Kebede G. Satisfaction with emergency obstetric and new born care services among clients using public health facilities in Jimma Zone, Oromia Regional State, Ethiopia; a cross sectional study. BMC Pregnancy Childbirth. 2016;16(1). 2016 Apr 25;16:85. doi: 10.1186/s12884-016-0877-0 27113573 PMC4843201

[44] Bishanga D, Massenga J, Mwanamsangu A, Kim Y-M, George J, Kapologwe N, et al. Women’s Experience of Facility-Based Childbirth Care and Receipt of an Early Postnatal Check for Herself and Her Newborn in Northwestern Tanzania. Int J Environ Res Public Health. 2019 Feb 7;16(3):481. doi: 10.3390/ijerph16030481 PMCID: PMC6388277. 30736396 PMC6388277

[45] Paul J, Jordan R, Duty S, Engstrom JL. Improving Satisfaction with Care and Reducing Length of Stay in an Obstetric Triage Unit Using a Nurse-Midwife-Managed Model of Care. J Midwifery Womens Health. 2013;58(2):175–81. doi: 10.1111/j.1542-2011.2012.00239.x 23489525

[46] Jha P, Larsson M, Christensson K, Skoog Svanberg A. Satisfaction with childbirth services provided in public health facilities: results from a cross- sectional survey among postnatal women in Chhattisgarh, India. Glob Health Action. 2017;10(1):1386932. doi: 10.1080/16549716.2017.1386932 29087240 PMC5678347

[47] Lakew S, Ankala A, Jemal F. Determinants of client satisfaction to skilled antenatal care services at Southwest of Ethiopia: a cross-sectional facility based survey. BMC Pregnancy Childbirth. 2018 Dec 6;18(1):479. doi: 10.1186/s12884-018-2121-6 30522442 PMC6282368

[48] Mutaganzwa C, Wibecan L, Iyer HS, Nahimana E, Manzi A, Biziyaremye F, et al. Advancing the health of women and newborns: predictors of patient satisfaction among women attending antenatal and maternity care in rural Rwanda. Int J Qual Health Care. 2018;30(10):793–801. doi: 10.1093/intqhc/mzy103 29767725 PMC6340346

[49] Khanna S, Sier D, Boyle J, Zeitz K. Discharge timeliness and its impact on hospital crowding and emergency department flow performance: Discharge Timeliness and Its Impact. Emerg Med Australas. 2016;28(2):164–170. doi: 10.1111/1742-6723.12543 26845068

[50] Miro O. Analysis of patient flow in the emergency department and the effect of an extensive reorganisation. Emerg Med J. 2003;20(2):143–148. doi: 10.1136/emj.20.2.143 12642527 PMC1726061

[51] Bard JF, Shu Z, Morrice DJ, Wang D, Poursani R, Leykum L. Improving patient flow at a family health clinic. Health Care Manag Sci. 2016;19(2):170–191. doi: 10.1007/s10729-014-9294-y 25155098

[52] Potisek NM, Malone RM, Shilliday BB, Ives TJ, Chelminski PR, DeWalt DA, et al. Use of patient flow analysis to improve patient visit efficiency by decreasing wait time in a primary care-based disease management programs for anticoagulation and chronic pain: a quality improvement study. BMC Health Serv Res. 2007 Jan 15;7:8. doi: 10.1186/1472-6963-7-8 17224069 PMC1784086

[53] Gjolaj LN, Campos GG, Olier-Pino AI, Fernandez GL. Delivering Patient Value by Using Process Improvement Tools to Decrease Patient Wait Time in an Outpatient Oncology Infusion Unit. J Oncol Pract. 2016 Jan;12(1):e95–e100. doi: 10.1200/JOP.2015.006155 26420892

[54] Neuman M, Alcock G, Azad K, Kuddus A, Osrin D, More NS, et al. Prevalence and determinants of caesarean section in private and public health facilities in underserved South Asian communities: cross-sectional analysis of data from Bangladesh, India and Nepal. BMJ Open. 2014;4:e005982. doi: 10.1136/bmjopen-2014-005982 25550293 PMC4283435

[55] Golandaj JA, Hallad JS. Levels, trends and socio-economic correlates of caesarean section deliveries: District level analysis in Karnataka, India. J Health Res. 2019;33(4):323–335.

[56] Kant S, Kaur R, Malhotra S, Haldar P, Goel A. Audit of emergency obstetric referrals from a secondary level hospital in Haryana, North India. J Fam Med Prim Care. 2018;7(1):137–141. doi: 10.4103/jfmpc.jfmpc_16_17 29915747 PMC5958555

