# Supplementary material for: Improving quality of care for pregnancy, perinatal and newborn care at district and sub-district public health facilities in three districts of Haryana, India: An Implementation study
Source: PLoS One. 2021 Jul 23;16(7):e0254781. doi: 10.1371/journal.pone.0254781 (PMC8301676; doi:10.1371/journal.pone.0254781)
Supplement: S1 Checklist — (DOCX) [file pone.0254781.s001.docx]

Good Reporting of A Mixed Methods Study (GRAMMS) checklist

Title: Improving quality of care for pregnancy, perinatal and newborn care at district and sub-district public health facilities in three districts of Haryana, India: An implementation study

| Guideline | Section: page |
| --- | --- |
| Describe the justification for using a mixed methods approach to the research question | Methods- under Design and intervention(s); page 8-9 |
| Describe the design in terms of the purpose, priority and sequence of methods | Methods- under Data Collection, Page 8-9 |
| Describe each method in terms of sampling, data collection and analysis | Methods- under Data Collection, Page 12  Methods- under Data Management and Analysis, Page 12-13 |
| Describe where integration has occurred, how it has occurred and who has participated in it | Methods- under Data Management and Analysis, Pages 9 and 27-32 |
| Describe any limitation of one method associated with the present of the other method | Discussion, Page 39-40 |
| Describe any insights gained from mixing or integrating methods | Discussion, Page 40-41 |

*Ref: O'Cathain A, Murphy E, Nicholl J. The quality of mixed methods studies in health services research. J Health Serv Res Policy. 2008;13: 92-98*
