# Supplementary material for: Improving quality of care for pregnancy, perinatal and newborn care at district and sub-district public health facilities in three districts of Haryana, India: An Implementation study
Source: PLoS One. 2021 Jul 23;16(7):e0254781. doi: 10.1371/journal.pone.0254781 (PMC8301676; doi:10.1371/journal.pone.0254781)
Supplement: S3 Checklist. Completed StaRI checklist — (DOCX) [file pone.0254781.s003.docx]

**Title: Improving quality of care for pregnancy, perinatal and newborn care at district and sub-district public health facilities in three districts of Haryana, India: An implementation study**

**Completed StaRI checklist**

| Checklist item | | Reported on page # | Implementation Strategy | Reported on page # | Intervention |  |
| --- | --- | --- | --- | --- | --- | --- |
|  | |  | “Implementation strategy” refers to how the intervention was implemented |  | “Intervention” refers to the healthcare or public health intervention that is being implemented. |  |
| Title and abstract | | | | | |  |
| Title | 1 | **Title**  **(Page 1)** | Identification as an implementation study, and description of the methodology in the title and/or keywords | | |  |
| Abstract | 2 | **Abstract**  **(Page 3)** | Identification as an implementation study, including a description of the implementation strategy to be tested, the evidence-based intervention being implemented, and defining the key implementation and health outcomes. | | |  |
| Introduction | | | | | |  |
| Introduction | 3 | **Introduction (Pages 5-7)** | Description of the problem, challenge or deficiency in healthcare or public health that the intervention being implemented aims to address. | | |  |
| Rationale | 4 | **Introduction**  **(Pages 5-7) and**  **Methods (Page 8)** | The scientific background and rationale for the implementation strategy (including any underpinning theory/framework/model, how it is expected to achieve its effects and any pilot work). | **Introduction**  **(Pages 5-7) and**  **Methods (Page 8)** | The scientific background and rationale for the intervention being implemented (including evidence about its effectiveness and how it is expected to achieve its effects). |  |
| Aims and objectives | 5 | **Introduction (Page 7)** | The aims of the study, differentiating between implementation objectives and any intervention objectives. | | |  |
| Methods: description | | | | | |  |
| Design | 6 | **Methods (Pages 8-9)** | The design and key features of the evaluation, (cross referencing to any appropriate methodology reporting standards) and any changes to study protocol, with reasons | | |  |
| Context | 7 | **Methods (Pages 8)** | The context in which the intervention was implemented. (Consider social, economic, policy, healthcare, organisational barriers and facilitators that might influence implementation elsewhere). | | |  |
| Targeted ‘sites’ | 8 | **Methods**  **(Pages 8)** | The characteristics of the targeted ‘site(s)’ (e.g locations/personnel/resources etc.) for implementation and any eligibility criteria. | **Methods (Page 8)** | The population targeted by the intervention and any eligibility criteria. |  |
| Description | 9 | **Methods (Pages 8-13)** | A description of the implementation strategy | **Methods**  **(Pages 8-13)** | A description of the intervention |  |
| Sub-groups | 10 | **Not applicable** | Any sub-groups recruited for additional research tasks, and/or nested studies are described | | |  |
| Methods: evaluation | | | | | |  |
| Outcomes | 11 | **Methods**  **(Pages 9-13)** | Defined pre-specified primary and other outcome(s) of the implementation strategy, and how they were assessed. Document any pre-determined targets | **Method**  **(Pages 9-13)** | Defined pre-specified primary and other outcome(s) of the intervention (if assessed), and how they were assessed. Document any pre-determined targets |  |
| Process evaluation | 12 | **Methods (Pages 9-13)** | Process evaluation objectives and outcomes related to the mechanism by which the strategy is expected to work | | |  |
| Economic evaluation | 13 | **Not applicable** | Methods for resource use, costs, economic outcomes and analysis for the implementation strategy | **Not applicable** | Methods for resource use, costs, economic outcomes and analysis for the intervention |  |
| Sample size | 14 | **Not applicable** | Rationale for sample sizes (including sample size calculations, budgetary constraints, practical considerations, data saturation, as appropriate) | | |  |
| Analysis | 15 | **Methods (Pages 12-13)** | Methods of analysis (with reasons for that choice) | | |  |
| Sub-group analyses | 16 | **Not applicable** | Any a priori sub-group analyses (e.g. between different sites in a multicentre study, different clinical or demographic populations), and sub-groups recruited to specific nested research tasks | | |  |
| Results | | | | | | |
| Characteristics | | 17 | **Not applicable** | Proportion recruited and characteristics of the recipient population for the implementation strategy | **Not applicable** | Proportion recruited and characteristics (if appropriate) of the recipient population for the intervention |
| Outcomes | | 18 | **Results (Pages 14-34)** | Primary and other outcome(s) of the implementation strategy | **Results**  **(Pages 14-34)** | Primary and other outcome(s) of the Intervention (if assessed) |
| Process outcomes | | 19 | **Results**  **(Pages 14-34)** | Process data related to the implementation strategy mapped to the mechanism by which the strategy is expected to work | | |
| Economic evaluation | | 20 | **Not applicable** | Resource use, costs, economic outcomes and analysis for the implementation strategy | **Not applicable** | Resource use, costs, economic outcomes and analysis for the intervention |
| Sub-group analyses | | 21 | **Not applicable** | Representativeness and outcomes of subgroups including those recruited to specific research tasks | | |
| Fidelity/ adaptation | | 22 | **Results (Pages 31-34)** | Fidelity to implementation strategy as planned and adaptation to suit context and preferences | **Results (Pages 31-34)** | Fidelity to delivering the core components of intervention (where measured) |
| Contextual changes | | 23 | **Results (Pages 31-34)** | Contextual changes (if any) which may have affected outcomes | | |
| Harms | | 24 | **Not applicable** | All important harms or unintended effects in each group | | |
| Discussion | | | | | | |
| Structured discussion | | 25 | **Discussion (Pages 35-40)** | Summary of findings, strengths and limitations, comparisons with other studies, conclusions and implications | | |
| Implications | | 26 | **Discussion (Pages 35-41)** | Discussion of policy, practice and/or research implications of the implementation strategy (specifically including scalability) | **Discussion (Pages 35-41)** | Discussion of policy, practice and/or research implications of the intervention (specifically including sustainability) |
| General | | | | | | |
| Statements | | 27 | **Declarations section**  **(Pages 13-14; 42-44)** | Include statement(s) on regulatory approvals (including, as appropriate, ethical approval, confidential use of routine data, governance approval), trial/study registration (availability of protocol), funding and conflicts of interest | | |

*Ref: Pinnock H, Barwick M, Carpenter C R, Eldridge S, Grandes G, Griffiths C J et al. Standards for Reporting Implementation Studies (StaRI) Statement BMJ 2017; 356 :i6795 doi:10.1136/bmj.i6795*
