# Supplementary material for: Improving quality of care for pregnancy, perinatal and newborn care at district and sub-district public health facilities in three districts of Haryana, India: An Implementation study
Source: PLoS One. 2021 Jul 23;16(7):e0254781. doi: 10.1371/journal.pone.0254781 (PMC8301676; doi:10.1371/journal.pone.0254781)
Supplement: S1 Table — (PDF) [file pone.0254781.s005.pdf]

S1 Table. Organisation of the public health system in India and the maternal and newborn services[1-3]

Facility	Facilities and services related to maternal and newborn care
Tertiary care facilities (Medical colleges and other referral hospital)	• Comprehensive and advanced care for all types of diseases<br>• Variable bed strength, doctor (including specialists and super-specialists) and paramedical staffs<br>• Comprehensive pregnancy and childbirth care, comprehensive newborn care including critical care, child health, immunization, nutrition, family planning, adolescent health, care for communicable diseases, non-communicable diseases, mental health, cancer, oral health, elderly care and 24x7 emergency care<br>• Referral for multiple districts and states
District hospital (DH)*	• 1,000,000-6,000,000 population<br>• Outpatients and in-patient services (100-500 beds), labour room, operation theatres, laboratory, radiology, blood bank and ambulance<br>• 32-68 doctors (19-40 specialists including >1 obstetrician and paediatrician) and 76-325 nursing and paramedical staffs<br>• Pregnancy and childbirth (including caesarean section), postnatal care, essential newborn care, newborn illness care (SNCU 12-24 beds), child health, immunization and nutrition, family planning, adolescent health, communicable disease care and surveillance, non-communicable disease, mental health, oral health program implementation, elderly care and 24x7 emergency care<br>• Referral, supervision and monitoring for the whole district
Sub-district hospital (SDH)*	• 500,000-600,000 population<br>• Outpatients and in-patient services (31-100 beds), labour room, operation theatre, laboratory, radiology facility, blood bank/storage and ambulance<br>• 20-30 doctors (10 specialists including obstetrician and paediatrician) and 45-75 nursing and paramedical staffs<br>• Pregnancy and childbirth (including caesarean section), postnatal care, essential newborn care, newborn illness care (SNCU 6-12 beds), child health, immunization and nutrition, family planning, adolescent health, communicable disease care and surveillance, non-communicable disease, mental health, oral health program implementation, elderly care and 24x7 emergency care<br>• First referral unit for multiple PHCs and CHCs
First referral unit (FRU)*	• 120,000 population (80,000 in hilly/tribal areas)<br>• Outpatients and in-patient services (30 beds), labour room, operation theatre, laboratory, x-ray facility, blood storage and ambulance<br>• 11 doctors (4-5 specialists including obstetrician and paediatrician) and 35-41 nursing and paramedical staffs<br>• Pregnancy and childbirth (including caesarean section), postnatal care, essential newborn care, newborn illness care (NBSU), child health, immunization and nutrition, family planning, adolescent health, communicable disease care and surveillance, non-communicable disease, mental health, oral health program implementation, elderly care and 24x7 emergency care<br>• Referral, supervision and monitoring of 4-5 PHCs
Community Health Centre (CHC)	• 120,000 population (80,000 in hilly/tribal areas)<br>• Outpatients and in-patient services (30 beds) with labour room, operation theatre, laboratory, x-ray facility, blood storage (may) and ambulance<br>• 11 doctors (4-5 specialists including obstetrician and paediatrician) and 35-41 nursing and paramedical staffs<br>• Pregnancy and childbirth (including caesarean section), postnatal care, essential newborn care, newborn illness detection and referral, child health, immunization and nutrition, family planning, adolescent health, communicable disease care and

	surveillance, non-communicable disease, mental health, oral health program implementation, elderly care and emergency care (24 hours)<br>• Referral, supervision and monitoring of 4-5 PHCs
Primary Health Centre (PHC)	• 30,000-50,000 population (20,000-25,000 in hilly/tribal areas)<br>• Outpatients and in-patient services (6 beds) with labour room, laboratory, x-ray facility and ambulance<br>• 1-3 doctors and 12-19 nursing and paramedical staffs<br>• Pregnancy and childbirth (including assisted deliveries), postnatal care, essential newborn care, illness detection and referral, child health, immunization and nutrition, family planning, adolescent health, communicable disease care and surveillance, non-communicable disease, mental health, oral health program implementation, elderly care and emergency care (24 hours)<br>• Supervision and monitoring of 5 Sub health centres
Health and Wellness Centre (HWC)[(2)]	• 5000-20000 population<br>• Outpatients and outreach services (no inpatient service)<br>• 2-3 MPW for rural and one MPW per 10000 population (urban)<br>• Pregnancy and childbirth, essential newborn care, illness detection and referral, child health, immunization and nutrition, family planning, adolescent health, communicable disease care and surveillance, non-communicable disease, mental health and oral health program implementation, elderly care and emergency care<br>• Supervision and monitoring of 4-5 ASHAs by each MPW
Sub Health Centre (SCH)	• One per 5000 populations (3000 in hilly/tribal/desert area)<br>• Outpatients and outreach services (no inpatient service)<br>• 1-2 Auxillary Nurse and Mid-wife (ANM)<br>• Antenatal care, deliveries (if needed), essential newborn care, immunization, illness detection and referral, family planning, adolescent health, disease surveillance and control of epidemics and non-communicable disease program implementation<br>• Supervision and monitoring of 5 ASHAs
Community level	• 1 ASHA per 1000 (rural) or 2500 (urban) population<br>• Mobilize pregnant women for antenatal care and institutional delivery, home visits for newborn care, mobilise for immunization, assist in referral, dispensing drugs, contraception and supplements

*Note: * The facilities included in the current study were FRUs, SDH and DHs.*
ASHA: Accredited Social Health Activist; MPW: Multipurpose worker; NBSU: Newborn stabilisation unit; SNCU: Sick newborn care unit-NBSU,

References

1. Ministry of Health and Family Welfare, Government of India. Indian Public Health Standards (IPHS) for Sub-centres, Primary Health Centres (PHCs), Community Health Centres (CHCs), Sub-District and District Hospitals. 2012. Available from: https://nhm.gov.in/index1.php?lang=1&level=2&sublinkid=971&lid=154#:~:text=IPHS%20are%20a%20set%20of,especially%20for%20Non%2DCommunicable%20Diseases.
2. Ministry of Health and Family Welfare. Ayushman Bharat. Comprehensive Primary Health Care through Health and Wellness Centers- Operational Guidelines. Government of India; 2018. Available from: https://ab-hwc.nhp.gov.in/download/document/45a4ab64b74ab124cfd853ec9a0127e4.pdf
3. Ministry of Health and Family Welfare, Government of India. Infrastructure. Health System Strengthening, National Health Mission. Available from: https://nhm.gov.in/index1.php?lang=1&level=2&sublinkid=1220&lid=190
