# Supplementary material for: Improving quality of care for pregnancy, perinatal and newborn care at district and sub-district public health facilities in three districts of Haryana, India: An Implementation study
Source: PLoS One. 2021 Jul 23;16(7):e0254781. doi: 10.1371/journal.pone.0254781 (PMC8301676; doi:10.1371/journal.pone.0254781)
Supplement: S2 Table — (PDF) [file pone.0254781.s006.pdf]

S2 Table. Demographic and health profile of Haryana and study districts

	Haryana				India
Parameters	Faridabad	Rewari	Jhajjar	State	
Population demography					
Total population, millions (2011)[a]	1.8	0.90	0.95	25.35	1,210
Population density (2011)[a]	2442	565	523	573	382
Rural population (%) (2011)[a]	20.5	74	74.6	651.	68.8
Scheduled caste population (%) (2011)[a]	12.3	20	17.8	19.3	16.6
Female literacy (%) (2011)[a]	73.8	69.6	70.7	65.9	65.5
Socioeconomic indicator					
Per capita income, Indian Rupees/ US$* (2017-18)[b]	-	-		178,890 /2702	103,870 /1569
Below poverty line population (%) (2011-12)[c]	-	-		11.16	21.92
Maternal and child health indicators					
Pregnant women with full antenatal check-ups (%) (2015-16)[d]	49.3	45.7	28.5	49.3	66.4
Pregnant women protected against tetanus (%) (2015-16)[d]	93	91.2	80.8	93.0	89.9
Institutional delivery (%) (2015-16)[d]	80.6	90.2	90.6	80.6	88.7
Institutional delivery at public health facilities (%) (2015-16)[d]	46.8	69.2	50.7	46.3	46.2
Caesarean section deliveries (%) (2015-16) [d]	13.6	12.8	14.5	13.6	28.2
Children breastfed within one hour (%) (2015-16)[d]	38.3	41.2	36.7	38.3	42.8
Infant mortality rate (2017)[e]	-	-	-	30	33
Neonatal mortality rate (2017)[f]	-	-	-	22	23.7
Maternal mortality ratio (2015-17)[g]	-	-	-	98	122
Public Health Facilities					
Community Health Centres [h]	4	5	6	128	5,624
First Referral Units [h]	2	2	2	20	3,057
Primary Health Centres [h]	16	21	27	529	25,743
Sub Health Centres [h]	58	112	126	2,650	158,417

Notes: a Census of India 2011, Registrar General of India (1)
b Economic Survey of Haryana (2017-18) (2)
c Reserve Bank of India (2011-12) (3)
d National Family Health Survey, India (2015-16) (4)
e SRS Bulletin (2017), Sample Registration System, Registrar General, India (5)
f^ UN Inter-agency Group for Child Mortality Estimation (www.childmortality.org.)(6)
g Special Bulletin on Maternal Mortality in India (2015-17), Registrar General, India (7)
h Infrastructure, Health Department, Government of Haryana (8)
** Average US $ to INR 66.2 for 2017-18*
Population density: number of persons per square kilometre area
Infant mortality rate per 1000 live births; Maternal mortality ratio- number of maternal deaths per 100000 live births; Neonatal mortality rate per 1000 live births

References

1. Registrar General of India. Census of India 2011 [Internet]. Government of India; [cited 2020 Mar 28]. Available from: http://censusindia.gov.in/2011-Common/CensusData2011.html
2. Department of Economic and Statistical Analysis, Haryana. Economic Survey of Haryana (2017-18) [Internet]. Government of Haryana; 2018 [cited 2020 Mar 28]. Available from: http://esaharyana.gov.in/Portals/0/ES%202017-18%20English.pdf
3. Reserve Bank of India. Table 162 : Number and Percentage of Population Below Poverty Line [Internet]. Reserve Bank of India; 2017 [cited 2020 Mar 28]. Available from: https://www.rbi.org.in/scripts/PublicationsView.aspx?id=17937
4. International Institute for Population Sciences. Fact Sheets for Key Indicators, National Family Health Survey-4, India (2015-16) [Internet]. International Institute for Population Sciences; [cited 2020 Mar 28]. Available from: http://rchiips.org/NFHS/factsheet_NFHS-4.shtml
5. Registrar General of India. SRS Bulletin. Sample Registration System, 2017, Vol. 52, No.1 [Internet]. Registrar General of India, Government of India; 2917. Available from: http://censusindia.gov.in/vital_statistics/SRS_Bulletins/SRS_Bulletin-Rate-2017-_May_2019.pdf
6. UN Inter-agency Group for Child Mortality Estimation. Estimates Developed by the UN Inter-agency Group for Child Mortality Estimation (UNICEF, WHO, World Bank, UN DESA Population Division) [Internet]. [cited 2020 Mar 21]. Available from: https://data.worldbank.org/indicator/SH.DYN.NMRT?locations=IN
7. Registrar General of India. Special Bulletin on Maternal Mortality in India 2015-17, Sample Registration System November 2019 [Internet]. Registrar General of India, Government of India; 2019. Available from: http://censusindia.gov.in/vital_statistics/SRS_Bulletins/MMR_Bulletin-2015-17.pdf
8. Department of Health and Family Welfare. Infrastructure, Health Department, Government of [Internet]. Government of Haryana; [cited 2020 Mar 28]. Available from: http://haryanahealth.nic.in/Infrastructure.html
