# Supplementary material for: Improving quality of care for pregnancy, perinatal and newborn care at district and sub-district public health facilities in three districts of Haryana, India: An Implementation study
Source: PLoS One. 2021 Jul 23;16(7):e0254781. doi: 10.1371/journal.pone.0254781 (PMC8301676; doi:10.1371/journal.pone.0254781)
Supplement: S3 Table — (PDF) [file pone.0254781.s007.pdf]

S3 Table. Quality management (QM) teams at the facilities in the districts and their compositions

Members Designation	No.	Members Designation	No.
1. Faridabad district			
1.1 Faridabad District QM Committee (n=10)			
Chief Medical Officer<br>Medical Superintendent of DH<br>Medical Officer In-charge FRU 1<br>Medical Officer In-charge FRU 2<br>External QI Project Investigator	1<br>1<br>1<br>1<br>1	RCH Officer<br>District Quality Monitor<br>HOD- Obstetrics<br>HOD-Pediatrics<br>State Child Health Consultant	1<br>1<br>1<br>1<br>1
1.2. Faridabad District Hospital QM Committee (n=12)			
Principal Medical Officer<br>Hospital Administration Medical Officers<br>District Quality Monitor<br>External QI Project Investigator<br>External QI Project District Coordinator	1<br>2<br>1<br>1<br>1	Surgery Specialist<br>HOD-Obstetrics<br>HOD-Pediatrics<br>Nursing Superintendent<br>Staff Nurse (LR)- Quality &<br>Infection Control	1<br>1<br>1<br>1<br>1<br>1
1.3. Faridabad FRU 1 QM Committee (n=7)			
Medical Officer in-charge & Obstetrician<br>Paediatrician<br>Dental Surgeon<br>External QI Project District Coordinator	1<br>1<br>1<br>1	Nursing In-charge<br>Pharmacist<br>Lab Technician	1<br>1<br>1
1.4 Faridabad FRU 2 QM Committee (n=7)			
Medical Officer in-charge<br>Obstetrician<br>Paediatrician<br>External QI Project District Coordinator	1<br>1<br>1<br>1	Nursing In-charge<br>Pharmacist<br>Lab Technician	1<br>1<br>1
2. Rewari district			
2.1 Rewari District QM Committee (n=10)			
Chief Medical Officer<br>Medical Superintendent of DH<br>Medical Officer In-charge FRU 1<br>Medical Officer In-charge FRU 2<br>External QI Project Investigator	1<br>1<br>1<br>1<br>1	RCH Officer<br>District Quality Monitor<br>Obstetrician<br>Paediatrician<br>State Child Health Consultant	1<br>1<br>1<br>1<br>1
2.2. Rewari District Hospital QM Committee (n=7)			
Medical Superintendent<br>District Quality Monitor<br>External QI Project Investigator<br>External QI Project District Coordinator	1<br>1<br>1<br>1	HOD-Obstetrics<br>HOD-Pediatrics<br>Nursing Superintendent	1<br>1<br>1
2.3. Rewari FRU 1 QM Committee (n=5)			
Medical Officer in-charge<br>External QI Project District Coordinator	1<br>1	Staff nurses<br>Pharmacist	2<br>1
2.4. Rewari FRU 2 QM Committee (n=4)			
Medical Officer in-charge<br>External QI Project District Coordinator	1<br>1	Staff nurse<br>Pharmacist	1<br>1

3. Jhajjar district			
3.1 Jhajjar District QM Committee (n=10)			
Chief Medical Officer	1	RCH Officer	1
Medical Superintendent of DH	1	District Quality Monitor	1
Medical Officer In-charge SDH	1	Obstetrician	1
Medical Officer In-charge FRU 2	1	Paediatrician	1
External QI Project Investigator	1	State Child Health Consultant	1
3.2. Jhajjar District Hospital QM Committee (n=12)			
Medical Superintendent	1	HOD-Pediatrics	1
Medical Officer- Quality in-charge	1	Nursing Superintendent	1
District Quality Monitor	1	Nurse- Quality management	1
HOD-Obstetrics	1	Nurse- infection control	1
External QI Project Investigator	1	Nurse in-charge- LR	1
External QI Project District Coordinator	1	Nurse in-charge- SNCU	1
3.3. Jhajjar SDH QM Committee (n=12)			
Medical Superintendent	1	HOD-Pediatrics	1
Medical Officer- Hospital administration	1	Nursing Superintendent	1
Medical Officer- Quality in-charge	1	Nurse- Quality management	1
HOD-Obstetrics	1	Nurse- infection control	1
External QI Project Investigator	1	Nurse in-charge- LR	1
External QI Project District Coordinator	1	Nurse in-charge- SNCU	1
3.4. Jhajjar FRU 2 QM Committee (n=4)			
Medical Officer in-charge	1	Medical officer	1
External QI Project District Coordinator	1	Staff nurse	1

Note: DH: District hospital; FRU: First referral unit; HOD: Head of department; LR: Labour room; QI: Quality improvement; QM: Quality management; RCH: Reproductive and child health; SDH: Sub-district hospital; SNCU: Sick newborn care unit
