# Supplementary material for: Improving quality of care for pregnancy, perinatal and newborn care at district and sub-district public health facilities in three districts of Haryana, India: An Implementation study
Source: PLoS One. 2021 Jul 23;16(7):e0254781. doi: 10.1371/journal.pone.0254781 (PMC8301676; doi:10.1371/journal.pone.0254781)
Supplement: S4 Table — (PDF) [file pone.0254781.s008.pdf]

S4 Table. List of topics for self-learning and facilitated learning during weekly meetings

Sl no	Topic	Types of resource materials
1	*Section 1. Essential newborn care*	
1.1	Care of normal newborn at birth and postpartum period	Reading material, Job aid, Checklist, Video
1.2	Essential newborn care	Reading material, Job aid, Checklist
1.3	Hypothermia and thermal control	Reading material, Job aid
1.4	Breastfeeding	Reading material, Job aid, Checklist, Poster, Video
1.5	Neonatal Resuscitation	Reading material, Job aid, Video
2	*Section 2: Sick newborn care*	
2.1	Emergency triage assessment and treatment	Reading material, Job aid, Checklist, Video
2.2	Management of low birth weight and preterm newborn	Reading material, Job aid
	Hypothermia and Kangaroo mother care	Reading material, Job aid, Video
2.3	Neonatal jaundice	Reading material, Job aid
2.4	Management of hypoglycaemia	Reading material, Job aid, Video
2.5	Neonatal sepsis	Reading material, Job aid, Video
2.6	Neonatal shock and fluid management	Reading material, Job aid
2.7	Respiratory distress in newborn	Reading material, Job aid, Video
2.8	Neonatal seizures	Reading material, Job aid, Video
2.9	Post resuscitation management of an asphyxiated neonate	Reading material, Job aid
2.10	Anaemia & bleeding in neonates	Reading material
2.11	Fluid management	Reading material
2.12	Neonatal transport	Reading material
2.13	Follow up of high risk newborns	Reading material
2.14	Infection prevention and control in SNCU, hand Hygiene, disinfection	Reading materials, Job aid, Poster, Video
3	*Section 3: Obstetric care*	
3.1	Normal labour and delivery: Part 1- stages, assessment and monitoring including partograph	Reading materials, Guidelines, Checklist, Poster
3.2	Normal labour and delivery: Part 2- normal delivery, empowering birth companion	Reading materials, Guidelines, Checklist, Poster
3.3	Active management of third stage of labour	Reading materials, Guidelines, Checklist, Poster
3.4	Preterm labour: identification and management	Reading materials, Guidelines, Poster
3.5	Prolonged labour: Identification and management	Reading materials, Guidelines, Checklist, Poster
3.6	Assisted delivery: Forceps and vacuum assisted delivery	Reading materials, Guidelines, Videos
3.7	Caesarean section: when to consider and care after CS delivery	Reading materials, Guidelines, Checklist
3.8	Newborn resuscitation	Reading materials, Guidelines, Videos

Sl no	Topic	Types of resource materials
3.9	Infection prevention and control in LR, hand hygiene, disinfection	Reading materials, Job aid, Poster, Video
4	*Section 4: Postpartum care*	
4.1	Postpartum care- Part 1: Mother-assessments of mother, care and monitoring	Reading materials, Guidelines, Checklist, Videos
4.2	Postpartum care- Part 2: Newborn-Assessments of newborn, temperature maintenance, family planning counselling, danger signs	Reading materials, Guidelines, Checklist, Videos
4.3	Postpartum care- Part 3: Supporting breastfeeding	Reading material, Job aid, Checklist, Poster, Video
4.4	Puerperal sepsis: Identification management and prevention	Reading materials, Guidelines, Checklist
4.5	Postpartum haemorrhage: identification and management	Reading materials, Guidelines, Checklist, Videos
4.6	Counselling of mother at discharge	
5	*Section 5: Antenatal care*	
5.1	ANC, EDD, abdominal examination, laboratory investigations, counselling, birth planning & screening for high risk pregnancy	Reading materials, Job aid, ANC card, Poster
5.2	PIH, detection, monitoring, management of eclampsia including Inj. $MgSO_4$	Reading materials, Job aid, ANC card, Poster
5.3	Gestational diabetes: Identification, management and counselling	Reading materials, Job aid
5.4	Anaemia: Identification, management and prevention	Reading materials, Job aid
5.5	Abortion and antepartum haemorrhage: Identification, management and counselling	Reading materials, Job aid
6	*Section 6: General*	
6.1	Handwashing and infection control training for all support workers	Reading materials, Poster, Video
6.2	Biomedical waste management	Reading materials, Poster, Video
6.3	Equipment handling and maintenance demonstration	Demonstration and hands-on practice
6.4	Communication	Reading material, Job aid, Video
7	*Section 7: Quality Improvement*	
7.1	Quality management system- Session 1: Principles and components	Reading material, Job aid, Discussion
7.2	Quality management system- Session 2: Documentation, monitoring and sustenance	Reading material, Job aid, Discussion

Note: ANC: Antenatal care; CS: Caesarean section; EDD: Expected date of delivery; LR: Labour room; $MgSO_4$: Magnesium Sulfate; PIH: Pregnancy induced hypertension
