# Supplementary material for: Improving quality of care for pregnancy, perinatal and newborn care at district and sub-district public health facilities in three districts of Haryana, India: An Implementation study
Source: PLoS One. 2021 Jul 23;16(7):e0254781. doi: 10.1371/journal.pone.0254781 (PMC8301676; doi:10.1371/journal.pone.0254781)
Supplement: S5 Table — (PDF) [file pone.0254781.s009.pdf]

S5 Table. Outcome Indicators for the impact of quality improvement and frequency of the data collection

Sl.no.	Indicators	Data Source	Baseline	Quarterly	Endline
	Primary Outcome Indicators				
1	*Obstetric care (records)*				
1.1	PIH cases detected (%)	CRR, Register review	X	X	X
1.2	MagSulf given to PIH cases (%)		X	X	X
1.3	Oxytocin given after delivery (%)		X	X	X
1.4	Prolonged labour cases (%)		X	X	X
1.5	Still births (%)		X	X	X
1.6	Severe systemic infection (%)		X	X	X
1.7	Maternal deaths (%)		X	X	X
2	*Newborn care at birth (observed)*				
2.1	Immediate drying (%)	DO	X	X	X
2.2	Skin-to-skin contact (%)		X	X	X
2.3	Delayed cord clamping (%)		X	X	X
2.4	BF initiation (%)		X	X	X
3	*Antenatal care (observation)*				
3.1	HRP cases identified (%)	CRR, DO	X	X	X
3.2	ANC counselling & birth planning (%)		X	X	X
4	*Sick newborn care (records)*				
4.1	Total deaths (%)	Hospital data, CRR	X	X	X
4.2.1	Deaths- weight >2500gms (%)		X	X	X
4.2.2	Deaths- weight <2500gms (%)		X	X	X
5	*Availability of services (review)*				
5.1	Bag & mask, oxygen- LR/PNW/SNCUs (%)	DO, Stock register review	X	X	X
5.2	Medicines-obstetrics & neonates (%)		X	X	X
6	*Disinfection practice (observation)*				
6.1	Soap & water in LR and SNCUs (%)	DO	X	X	X
6.2	Hand rub in LR and SNCU (%)		X	X	X
7	*Patient satisfaction (interview)*				
7.1	Women who delivered (%)	Survey-mothers/ family	X	X	X
7.2	Women attending ANC (%)		X	X	X
7.3	Mothers of sick newborns (%)		X	X	X
B	*Secondary Outcome Indicators*				
8	*Obstetric patient service delivery*				
8.1	Total number of deliveries (n)	Hospital data, CRR	X	X	X
8.2	Vaginal delivery (%)		X	X	X
8.3	Caesarean section (%)		X	X	X
8.4	Stillbirth (%)		X	X	X
8.5	Referred (%)		X	X	X
8.6	Antenatal clinical attendance (n)		X	X	X
8.7	High risk pregnancies (%)		X	X	X
9	*Newborn patient service delivery*				
9.1	Total admissions (n)	Hospital data, CRR	X	X	X
9.2	Inborn (%)		X	X	X
9.3	Discharge (%)		X	X	X

Sl.no.	Indicators	Data Source	Baseline	Quarterly	Endline
9.4	Referred/LAMA (%)		X	X	X
10	*Time spent in ANC facility*				
10.1	ANC- time to 1st contact with nurse (min)	DO	X	X	X
10.2	ANC- Total time spent (min)		X	X	X
11	*Time for response in LR*				
11.1	Time to 1st contact (min)				
12	*Time for response in SNCU*				
12.1	Time to 1st contact (min)				

Note: BL: Baseline; EL: Endline; LR: Labour room; PNW: Postnatal ward; ANC: Antenatal care; SNCU: Sick newborn care unit; BF: Breastfeeding; HRP: high risk pregnancy; PIH: Pregnancy induced hypertension; MagSulf: Magnesium Sulfate injection; LAMA: Left against medical advice; CRR: Case record review; DO: Direct observation
