# Supplementary material for: Improving quality of care for pregnancy, perinatal and newborn care at district and sub-district public health facilities in three districts of Haryana, India: An Implementation study
Source: PLoS One. 2021 Jul 23;16(7):e0254781. doi: 10.1371/journal.pone.0254781 (PMC8301676; doi:10.1371/journal.pone.0254781)
Supplement: S6 Table — (PDF) [file pone.0254781.s010.pdf]

S6 Table. Data collected related to the quality improvement interventions in the study districts

Sl.No	Data collected	Quality Improvement Cycles						Total
		First	Second	Third	Fourth	Fifth	Sixth	
A	Quantitative data collected							
1	*Faridabad district*							
1.1	Facility assessment (n)*	3+12	3+12	3+12	3+12	3+12	3+12	18+72
1.2	Case record review (n)	666	921	857	544	620	637	4439
1.3	Patient satisfaction status (n)	401	665	649	422	678	483	3453
1.4	Observation of care at birth (n)	0	0	132	181	206	188	707
1.5	Hand hygiene observations (n)	0	0	1646	1911	1201	728	5486
1.6	Patient flow analysis (n)	171	226	227	185	188	157	1265
1.7	Knowledge assessment (n)	0	0	0	0	0	20	48
2	*Rewari district*							
2.1	Facility assessment (n)*	3+12	3+12	3+12	3+12	3+12	3+12	18+72
2.2	Case record review (n)	719	811	735	742	785	908	4934
2.3	Patient satisfaction status (n)	399	597	519	439	746	590	3434
2.4	Observation of care at birth (n)	0	0	43	161	180	232	616
2.5	Hand hygiene observations (n)	0	0	630	1774	2200	2192	6796
2.6	Patient flow analysis (n)	120	130	117	98	96	98	735
2.7	Knowledge assessment (n)	0	0	0	0	0	32	58
3	*Jhajjar district*							
3.1	Facility assessment (n)*	3+12	3+12	3+12	3+12	3+12	3+12	18+72
3.2	Case record review (n)	651	1057	877	759	770	861	5171
3.3	Patient satisfaction status (n)	330	353	354	377	395	424	2371
3.4	Observation of care at birth (n)	0	0	105	310	331	347	1093
3.5	Hand hygiene observations (n)	0	0	424	534	700	882	2540
3.6	Patient flow analysis (n)	151	138	150	129	141	141	953
3.7	Knowledge assessment (n)	0	0	0	0	0	22	43
4	*Pooled (all three districts)*							
4.1	Facility assessment (n)*	9+36	9+36	9+36	9+36	9+36	9+36	54+216
4.2	Case record review (n)	2036	2789	2469	2045	2175	2406	14544
4.3	Patient satisfaction status (n)	1130	1615	1522	1238	1819	1497	9258
4.4	Observation of care at birth (n)	0	0	280	652	717	767	2416
4.5	Hand hygiene observations (n)	0	0	2700	4219	4101	3802	14822
4.6	Patient flow analysis (n)	442	494	494	412	425	396	2953
4.7	Knowledge assessment (n)	0	0	0	0	0	74	149
B	Qualitative data collected							
5	In-depth interviews							
5.1	Faridabad	28	-	-	-	-	27	55
5.2	Rewari	25	-	-	-	-	24	49
5.3	Jhajjar	29	-	-	-	-	28	57
	Pooled	82	-	-	-	-	79	161

Note: First cycle was the baseline period.

** The facility assessments: n1+n2; n1: quarterly detailed assessments and n2: weekly rapid assessments in the labour room, antenatal clinics and sick newborn units.*
