# Supplementary material for: Improving quality of care for pregnancy, perinatal and newborn care at district and sub-district public health facilities in three districts of Haryana, India: An Implementation study
Source: PLoS One. 2021 Jul 23;16(7):e0254781. doi: 10.1371/journal.pone.0254781 (PMC8301676; doi:10.1371/journal.pone.0254781)
Supplement: S7 Table. The quality gaps identified and resolved during the intervention period for each district — (PDF) [file pone.0254781.s011.pdf]

S7 Table. The quality gaps identified and resolved during the intervention period for each district

Sl no	Nature of the quality gaps	Items checked, n	Faridabad		Rewari		Jhajjar	
			Gaps identified, n (%)*	Gaps resolved n (%)**	Gaps identified, n (%)*	Gaps resolved n (%)**	Gaps identified, n (%)*	Gaps resolved n (%)**
1	*General Infrastructure and Systems*							
1.1	Layout	99	3 (9)	2 (67)	9 (27)	4 (44)	8 (24)	3 (38)
1.2	Infrastructure (general)	225	10 (13)	7 (70)	27 (36)	6 (22)	17 (23)	13 (76)
1.3	Maternal care services	117	7 (18)	5 (71)	16 (41)	5 (31)	15 (38)	8 (53)
1.4	Newborn and child care	90	13 (43)	6 (46)	18 (60)	5 (28)	17 (57)	4 (24)
1.5	Staffing	9	3 (100)	0 (0)	3 (100)	0 (0)	3 (100)	0 (0)
1.6	Information & records	99	4 (12)	0 (0)	7 (21)	3 43)	6 (18)	5 (83)
1.7	Essential drugs & blood storage	18	2 (33)	0 (0)	3 (50)	1 (33)	5 (83)	1 (20)
1.8	Pharmacy and drugs	72	3 (13)	0 (0)	6 (25)	3 (50)	6 (25)	4 (67)
1.9	Laboratory services	153	11 (22)	10 (91)	13 (25)	4 (31)	16 (31)	13 (81)
1.10	Guidelines & auditing	171	5 (9)	3 (60)	7 (12)	4 (57)	7 (12)	5 (71)
1.11	Supportive Care	108	8 (22)	3 (38)	5 (14)	3 (60)	10 (28)	9 (90)
2	*Maternal Health Services*							
2.1	Infrastructure	171	18 (32)	12 (67)	26 (46)	9 (35)	22 (39)	11 (50)
2.2.	Equipment	207	12 (17)	7 (58)	21 (30)	10 (48)	19 (28)	19 (100)
2.3	Staff availability	9	2 (67)	0 (0)	3 (100)	0 (0)	3 (100)	0 (0)
2.4	Care in maternity wards	54	6 (33)	6 (100)	5 (28)	2 (40)	6 (33)	5 (83)
2.5	Case management	414	7 (5)	6 (86)	25 (18)	6 (24)	23 (17)	13 (57)
2.6	Monitoring & follow-up	216	5 (7)	4 (80)	16 (22)	5 (31)	22 (31)	22 (100)
2.7	Infection control	108	5 (14)	4 (80)	11 (31)	8 (73)	5 (14)	5 (100)
3	*Newborn Health Services*							
3.1	Infrastructure	297	29 (29)	25 (86)	53 (54)	7 (13)	39 (39)	17 (44)
3.2	Equipment	297	19 (19)	18 (95)	21 (21)	1 (5)	39 (39)	17 (44)
3.3	Staff availability	9	3 (100)	0 (0)	3 (100)	0 (0)	3 (100)	0 (0)
3.4	Case management	153	25 (49)	23 (92)	28 (55)	7 (25)	20 (39)	7 (35)
3.5	Monitoring & follow-up	63	3 (14)	3 (100)	13 (62)	7 (54)	6 (29)	3 (50)

Sl no	Nature of the quality gaps	Items checked, n	Faridabad		Rewari		Jhajjar	
			Gaps identified, n (%)*	Gaps resolved n (%)**	Gaps identified, n (%)*	Gaps resolved n (%)**	Gaps identified, n (%)*	Gaps resolved n (%)**
3.6	Newborn care at birth	162	6 (11)	5 (83)	9 (17)	5 (56)	13 (24)	7 (54)
4	*Skill of health providers*							
4.1	Knowledge & skills-LR	36	5 (40)	2 (50)	6 (48)	5 (83)	5 (42)	3 (50)
4.2	Knowledge & skills-SNCU	63	10 (46)	4 (43)	11 (51)	8 (78)	10 (46)	5 (48)
5	*Patient satisfaction*							
5.1	Patient satisfaction -LR	108	5 (13)	1 (31)	10 (27)	2.52 (26)	8 (21)	2 (29)
5.2	Patient satisfaction- ANC	81	6 (22)	1 (18)	4 (13)	0.54 (15)	3 (12)	1 (33)
5.3	Patient satisfaction- SNCU	90	5 (16)	2 (31)	4 (13)	1 (23)	4 (13)	0.3 (8)
6	*Clinical case record documentation*							
6.1	Clinical case record-LR	72	9 (37)	1 (8)	9 (37)	3 (38)	6 (23)	3 (52)
6.2	Clinical case record-ANC	63	8 (36)	1 (14)	12 (56)	6 (54)	8 (37)	4 (51)
6.3	Clinical case record-SNCU	63	3 (14)	0.4 (14)	2 (8)	1 (88)	6 (29)	3 (45)
7	*Grand total*	3972	268 (20)	171 (64)	409 (31)	139 (34)	393 (30)	220 (56)

Note: ANC: Antenatal clinic; LR: Labour room; SNCU: Sick newborn care unit

** The percentage estimated out of the total items checked, ** The percentage estimated out of the gaps identified*
