# Supplementary material for: Improving quality of care for pregnancy, perinatal and newborn care at district and sub-district public health facilities in three districts of Haryana, India: An Implementation study
Source: PLoS One. 2021 Jul 23;16(7):e0254781. doi: 10.1371/journal.pone.0254781 (PMC8301676; doi:10.1371/journal.pone.0254781)
Supplement: S8 Table — (PDF) [file pone.0254781.s012.pdf]

S8 Table. Key quality gaps observed at the facilities in the districts during formative research phase

Sl.no	Areas	Activities done/Facilitated
1	*Faridabad district*	
1.1	Gaps in facility assessment	• District hospital labour room: air conditioner not functional, safe drinking water not available, poor status of the toilets, bed sheet not changed daily, no specified waiting area for the family members and attendants<br>• District hospital SNCU: heating facility for baby feed preparation not available, sitting facility for doctors/nurses not adequate, breastfeeding room not operational, problem in water supply for handwashing area and drainage challenge<br>• FRUs: safe drinking water availability and maintenance problem, signage for various areas not available, diet for patients not regularly available, power backup challenge, no changing room for nurses
1.2	Gaps in clinical care practices	• District hospital labour room: Inadequate documentation of the clinical case records and registers, partograph documentation infrequent and incomplete, detailed delivery and birth register not used.<br>• District hospital SNCU: Inadequate clinical case record documentation<br>• FRU: partograph documentation infrequent and incomplete, poor counselling of mothers at discharge
1.3	Gaps in infection control	• District hospital labour room: autoclave facility and practice not organised, no routine schedule for equipment sterilization, hand washing facility in labour room not functional<br>• District hospital SNCU: water source is common with other ward-creating challenge for uninterrupted water supply, water drainage challenge from wash basin<br>• FRUs: equipment trays inadequate, autoclave not functional
1.4	Gaps in supervision & monitoring	• District hospital SNCU: No schedule for monitoring and supervision<br>• FRUs: no schedule for monitoring and supervision
1.5	Gaps in skill and knowledge	• District hospital Sick newborn care unit: Inadequate knowledge and skills in essential newborn care, resuscitation, sick newborn care, waste management<br>• FRUs: inadequate knowledge in partograph use, high risk pregnancy management, emergency obstetric case management, breastfeeding, essential newborn care, waste management, and infection control
2	*Rewari district*	
2.2	Gaps in facility assessment	• District hospital labour room: The signage and boards missing, intrapartum and postpartum case management protocols not displayed<br>• District hospital SNCU: Crowding inside the unit for sample collection<br>• FRUs: rregistration for antenatal check-up of low risk and high risk pregnant women same causing higher waiting time

Sl.no	Areas	Activities done/Facilitated
2.3	Clinical care practices	• District hospital SNCU: inadequate documentation of case records<br>• FRU: inadequate partograph use and APGAR recording, poor counselling at discharge, no antenatal ultrasound service
2.4	Infection control	• District hospital labour room: autoclave not functional<br>• FRUs: autoclave not functional, inadequate segregated waste management
2.5	Supervision & Monitoring	• District hospital SNCU: no schedule for supervision and monitoring<br>• FRUs: no schedule for supervision and monitoring
2.6	Skill building	• District hospital labour room: poor knowledge and skill on essential newborn care, resuscitation, partograph, safe child birth checklist use<br>• District hospital SNCU: poor knowledge and skill on essential newborn care, resuscitation, breastfeeding and alternate feeding, sick newborn management, waste management<br>• FRUs: poor knowledge and skill on partograph use, APGAR, child birth checklist, newborn resuscitation, breastfeeding, infection control
3	*Jhajjar district*	
3.1	Gaps in facility assessment	• District hospital labour room: Shortage of equipment and instrument, intrapartum and postpartum case management protocols not displayed<br>• District hospital SNCU: ceiling full of pigeon droppings, no privacy in KMC area, poor security for SNCU and KMC area<br>• Sub-district hospital labour room: shortage of equipment and instrument<br>• SDH SNCU: Security guards for SNCU and KMC ward, curtains for privacy in KMC ward
3.2	Clinical care practices	• District hospital labour room: Poor partograph documentation, poor counselling at discharge<br>• District hospital SNCU: poor and incomplete case documentation and clinical record keeping<br>• Sub-district hospital SNCU: poor and incomplete case documentation and clinical record keeping
3.3	Infection control	• District hospital labour room: autoclave not functional, no schedule<br>• Sub-district hospital labour room: no disinfection schedule and tracking
3.4	Supervision & Monitoring	• District hospital SNCU: no schedule for supervision and monitoring<br>• Sub-district hospital SNCU: no schedule for supervision and monitoring
3.5	Skill building	• District hospital labour room: poor knowledge on essential newborn care, resuscitation<br>• District hospital SNCU: poor knowledge on sick newborn care, resuscitation, breastfeeding, waste management

Sl.no	Areas	Activities done/Facilitated
		• FRU: poor knowledge and skill of partograph use, newborn resuscitation, breastfeeding, sick newborn identification, infection control
4	*Generic observations*	
4.1	Orientation about Quality of Care	• The orientation and understanding of quality of care were limited among the health care providers across all levels, support team members and the administrators.<br>• But most administrators and quality team members were aware of the quality improvement efforts by Government.
4.2	Willingness for Quality of Care	• Majority of the service providers (doctors and nurses) had apprehension and hesitation about the quality improvement effort. Their concerns were regarding:<br>- The purpose, components, and activities;<br>- Perceived that these would add to work load (documentation, processes), as there was no cross check of records, no schedule/monitoring, documentation; and<br>- Several of the specialists/doctors had apprehension and response that "it cannot be done";<br>• The FRU teams had enthusiasm, as they expected to gain in knowledge and practice skills through the quality improvement process.
4.3	Willingness of the administrator	• Willingness of the administrator for quality improvement was evident.<br>• They were willing to facilitate and support the Quality Improvement team for the implementation of desired activities.<br>• They were open to receive the findings and expressed willingness to address the gaps as per the administrative and financial procedures.
4.4	Administrative challenges	• Some administrative and staff related conflicts between health services and National Health Mission for SNCU was observed.<br>• Poor timeline for payment to vendors for services/repair etc., which challenged the regular service availability.<br>• On the face "no shortage of funds" but release of fund and related procedures were challenge for the service providers.
4.5	Counselling of patients	• Counselling in the postnatal wards, at discharge and antenatal clinics was a challenge as no dedicated person was allocated and no protocol followed.
4.6	Manpower shortage	• Manpower shortage was universal and critical constraint.<br>• Relocation of staffs on adhoc basis between departments and units were being done to meet the challenges.<br>• Several staffs were contractual in nature.

Note: SNCU: Sick newborn care unit; FRU: First referral unit; KMC: Kangaroo mother care; APGAR: appearance (colour), pulse (heart rate), grimace (reflexes), activity (muscle tone), and respiration
