# Supplementary material for: Improving quality of care for pregnancy, perinatal and newborn care at district and sub-district public health facilities in three districts of Haryana, India: An Implementation study
Source: PLoS One. 2021 Jul 23;16(7):e0254781. doi: 10.1371/journal.pone.0254781 (PMC8301676; doi:10.1371/journal.pone.0254781)
Supplement: S9 Table — (PDF) [file pone.0254781.s013.pdf]

S9 Table. Changes in the infrastructure, manpower and processes at the hospitals in the three districts

Sl. No	Health services components	Faridabad								Rewari								Jhajjar							
		District hospital		FRU-1		FRU-2		District Pooled		District hospital		FRU 1		FRU 2		District Pooled		District hospital		FRU1/SDH		FRU 2		District Pooled	
		Base-line	End-line	Base-line	End-line	Base-line	End-line	Base-line	End-line	Base-line	End-line	Base-line	End-line	Base-line	End-line	Base-line	End-line	Base-line	End-line	Base-line	End-line	Base-line	End-line	Base-line	End-line
A	*General Health Services, n*	12	12	12	12	12	12	12	12	12	12	12	12	12	12	12	12	12	12	12	12	12	12	12	12
A1	Layout (%)	100	100	82	91	91	91	91	94	91	91	73	82	73	82	79	85	100	100	100	100	25	50	75	83
A2	Infrastructure (general) (%)	86	89	96	100	92	100	91	96	86	86	68	71	64	71	73	76	92	96	75	96	50	90	72	94
A3	Maternal care services (%)	85	92	85	92	92	100	87	95	69	85	38	38	54	78	54	67	77	92	77	100	46	62	67	85
A4	Newborn & child care (%)	70	80	50	70	63	100	61	83	50	80	35	50	20	45	35	58	60	90	70	80	NA	NA	43	57
A5	Staffing (%)	56	67	89	89	84	63	76	73	66	78	78	63	72	86	72	76	87	87	84	84	35	50	69	74
A6	Information & records (%)	100	100	82	82	82	82	88	88	100	100	73	88	65	73	79	87	82	100	100	100	64	100	82	100
A7	Blood bank/storage (%)	100	100	0	0	0	0	33	33	75	100	0	0	0	0	25	33	75	100	25	25	0	0	33	42
A8	Pharmacy (%)	74	95	94	95	74	91	81	94	86	92	86	95	77	84	83	90	86	92	86	95	77	84	83	90
A9	Laboratory services (%)	90	100	71	100	76	100	79	100	72	80	68	88	50	67	63	78	71	94	82	100	71	88	75	94
A10	Guidelines and auditing (%)	95	100	100	100	95	100	97	100	95	100	50	75	50	75	65	83	100	100	100	100	63	91	88	97
A11	Supportive care (%)	83	100	67	75	75	75	75	83	83	100	50	75	50	75	61	83	100	100	67	100	83	89	83	96
	Sub-total general health	85	93	74	81	75	82	78	85	79	90	56	66	52	67	63	74	85	96	79	89	47	64	70	83
B	*Maternal Health Services, n*	12	12	12	12	12	12	12	12	12	12	12	12	12	12	12	12	12	12	12	12	12	12	12	12
B1	Infrastructure (%)	84	95	82	88	71	94	79	92	68	79	47	79	58	74	58	77	68	95	68	95	42	58	59	83
B2	Equipment (%)	83	91	65	95	74	87	74	91	78	94	91	100	52	61	74	85	75	100	91	100	75	100	80	100
B3	Staff availability (%)	81	74	75	75	89	89	82	79	76	86	78	80	72	73	75	80	98	98	86	88	45	66	76	84
B4	Care in maternity wards (%)	50	100	83	100	83	100	72	100	67	89	50	92	50	89	56	90	33	100	100	100	50	65	61	88
B5	Case management (%)	100	100	96	98	96	98	97	99	91	92	95	100	59	70	82	87	100	75	100	100	100	100	100	92
B6	Monitoring & follow-up (%)	100	100	96	100	92	100	96	100	96	94	79	79	71	82	82	85	83	100	60	100	63	100	69	100
B7	Infection control	83	100	92	100	83	100	86	100	92	100	83	100	42	88	72	96	100	100	92	100	100	100	97	100
	Sub-total maternal health	83	94	84	94	84	95	84	94	81	91	75	90	58	77	71	86	80	95	85	98	68	84	78	92
C	*Newborn Health Services, n*	12	12	12	12	12	12	12	12	12	12	12	12	12	12	12	12	12	12	12	12	12	12	12	12
C1	Infrastructure (%)	88	100	73	100	70	100	77	100	73	85	45	58	30	30	49	58	67	100	94	100	0	0	64	77
C2	Equipment (%)	100	100	85	97	75	100	87	99	94	94	79	79	67	67	80	80	75	100	91	100	0	0	66	78
C3	Staff availability (%)	68	91	0	0	0	0	23	30	66	86	0	0	0	0	22	29	50	83	86	86	0	0	45	56
C4	Case management (%)	94	100	82	100	71	94	82	98	94	100	57	51	22	22	58	58	67	100	100	100	0	0	63	74
C5	Monitoring & follow-up (%)	100	100	100	100	71	100	90	100	68	100	51	52	28	56	49	69	100	100	100	100	0	0	76	78
C6	Newborn care at birth (%)	100	100	83	100	89	100	91	100	83	100	94	100	78	78	85	93	83	100	94	100	0	0	67	83
	Sub-total newborn health	92	99	71	83	63	82	75	88	80	94	54	57	38	42	57	64	74	97	94	98	23	28	64	74

Note: FRU: First referral unit SDH: Sub-district hospital
