# Supplementary material for: Improving quality of care for pregnancy, perinatal and newborn care at district and sub-district public health facilities in three districts of Haryana, India: An Implementation study
Source: PLoS One. 2021 Jul 23;16(7):e0254781. doi: 10.1371/journal.pone.0254781 (PMC8301676; doi:10.1371/journal.pone.0254781)
Supplement: S10 Table — (PDF) [file pone.0254781.s014.pdf]

S10 Table. Changes in the quality of case record documentation at the hospitals in the three districts

Sl no	Components assessed	Faridabad								Rewari								Jhajjar							
		District hospital		FRU-1		FRU-2		District Pooled		District hospital		FRU 1		FRU 2		District Pooled		District hospital		FRU1/SDH		FRU 2		District Pooled	
		Base-line	End-line	Base-line	End-line	Base-line	End-line	Base-line	End-line	Base-line	End-line	Base-line	End-line	Base-line	End-line	Base-line	End-line	Base-line	End-line	Base-line	End-line	Base-line	End-line	Base-line	End-line
1	Labour room and postnatal wards, n	169	178	79	97	79	56	327	361	210	234	79	111	75	81	364	426	289	366	79	75	57	57	425	498
1.1	General information (%)	100	100	100	100	100	100	100	100	100	100	99	100	100	100	100	100	100	100	100	100	100	100	100	100
1.2	Pregnancy history (%)	76	85	89	**95**	84	**98**	83	**93**	76	**85**	65	**92**	72	**95**	71	**91**	95	99	87	**93**	80	**93**	87	**95**
1.3	Vitals (%)	67	**99**	83	**98**	65	**99**	72	**99**	67	**99**	67	**80**	65	**98**	66	**92**	93	**99**	84	**90**	64	**90**	80	**93**
1.4	Maternal history (%)	51	**99**	24	**100**	40	**100**	38	**100**	31	**99**	45	**55**	31	**85**	36	**80**	89	91	48	**78**	64	**95**	67	**88**
1.5	Labour and delivery (%)	75	**87**	83	**94**	71	**100**	76	**94**	75	**87**	88	**99**	88	**91**	84	**92**	90	**100**	90	95	70	**90**	83	**95**
1.6	Details of baby (%)	28	**99**	25	**99**	20	**99**	24	**99**	28	**99**	41	**50**	27	**76**	32	**75**	67	**75**	47	**75**	77	**98**	64	**83**
1.7	Hospital course (%)	40	**96**	57	**97**	38	**74**	45	**89**	40	**96**	48	**51**	54	**76**	47	**74**	73	78	65	**82**	79	80	72	**80**
1.8	Discharge/ Referral (%)	68	**100**	63	**100**	54	**100**	62	**100**	68	**100**	59	**75**	77	**82**	68	**86**	50	**75**	74	74	50	**70**	58	**73**
	Pooled- labour room and postnatal ward (%)	63	**96**	66	**98**	59	**96**	63	**97**	61	**96**	64	**75**	64	**88**	63	**86**	82	**90**	74	**86**	73	**90**	76	**89**
2	Antenatal check-up, n	113	116	41	39	39	38	193	193	178	183	91	84	24	24	293	291	172	180	48	43	12	12	232	235
2.1	Age of patient (%)	100	100	100	100	100	100	100	100	100	100	100	100	100	100	100	100	100	100	100	100	100	100	100	100
2.2	Pregnancy history (%)	94	99	70	**100**	87	**100**	84	**100**	73	85	26	**66**	33	**69**	44	**73**	93	97	90	96	100	100	94	98
2.3	Vitals (%)	22	**100**	50	**100**	44	**100**	39	**100**	29	**70**	38	**50**	25	**40**	31	**53**	38	**62**	75	**84**	40	**56**	51	**67**
2.4	Abdomen examination (%)	56	**100**	67	**100**	38	**100**	54	**100**	25	**60**	12	**45**	32	**64**	23	**56**	25	**60**	20	**57**	25	**45**	23	**54**
2.5	Investigation (%)	62	**100**	64	**100**	44	**100**	57	**100**	61	**76**	61	**79**	39	**77**	54	**77**	86	**95**	65	**82**	96	**94**	82	**90**
2.6	Medication (%)	62	**98**	98	100	95	100	85	**99**	53	**78**	42	**75**	38	**96**	44	**83**	85	**95**	50	**98**	75	**90**	70	**94**
2.7	Counselling (%)	11	**65**	40	**67**	40	**67**	30	**66**	10	**45**	20	**35**	0	**50**	10	**43**	17	**73**	0	**54**	50	**60**	22	**62**
	Pooled- Antenatal check-up (%)	58	**95**	70	**95**	64	**95**	64	**95**	50	**73**	43	**64**	38	**71**	44	**69**	63	**83**	57	**82**	69	**78**	63	**81**
3	Sick newborn care unit, n	237	255	-	-	-	-	237	255	257	245	-	-	-	-	257	245	174	159	122	125	-	-	296	284
3.1	General information (%)	92	**100**	-	-	-	-	92	100	91	95	-	-	-	-	91	95	99	100	85	98	-	-	92	99
3.2	Admission/discharge information (%)	94	98	-	-	-	-	94	98	99	99	-	-	-	-	99	99	97	94	81	93	-	-	89	94
3.3	Delivery information (%)	89	90	-	-	-	-	89	90	28	**75**	-	-	-	-	28	**75**	87	95	40	**77**	-	-	64	**86**
3.4	History (%)	27	**94**	-	-	-	-	27	**94**	58	**85**	-	-	-	-	58	**85**	84	**94**	12	**44**	-	-	48	**69**
3.5	Examination and clinical details (%)	58	**100**	-	-	-	-	58	**100**	66	**88**	-	-	-	-	66	**88**	85	**95**	37	**64**	-	-	61	**80**
3.6	Hospital course (%)	97	**100**	-	-	-	-	97	100	96	93	-	-	-	-	96	93	94	**100**	80	91	-	-	87	**96**
3.7	Discharge/Referral information (%)	86	**98**	-	-	-	-	86	**98**	92	93	-	-	-	-	92	93	64	**84**	51	**84**	-	-	58	**84**
	Pooled- Sick newborn care unit (%)	78	**97**	-	-	-	-	78	**97**	76	**90**	-	-	-	-	76	**90**	87	**95**	55	**79**	-	-	71	**87**

Note: The figures in bold indicate the change is statistically significant ($p < 0.05$); FRU: First referral unit; SDH: Sub-district hospital
