# Supplementary material for: Improving quality of care for pregnancy, perinatal and newborn care at district and sub-district public health facilities in three districts of Haryana, India: An Implementation study
Source: PLoS One. 2021 Jul 23;16(7):e0254781. doi: 10.1371/journal.pone.0254781 (PMC8301676; doi:10.1371/journal.pone.0254781)
Supplement: S11 Table — (PDF) [file pone.0254781.s015.pdf]

S11 Table. Changes in the patient satisfaction status at the hospitals in the three districts

Sl. No	Components assessed	Faridabad								Rewari								Jhajjar							
		District hospital		FRU-1		FRU-2		Pooled		District hospital		FRU-1		FRU-2		Pooled		District hospital		FRU-1/SDH		FRU-2		Pooled	
		Base-line	End-line	Base-line	End-line	Base-line	End-line	Base-line	End-line	Base-line	End-line	Base-line	End-line	Base-line	End-line	Base-line	End-line	Base-line	End-line	Base-line	End-line	Base-line	End-line	Base-line	End-line
1	*Labour rooms (LR) and postnatal wards, n*	133	123	15	38	16	17	164	178	149	133	67	123	14	13	230	269	94	84	59	66	14	29	167	179
1.1	Assistance in registration (%)	93	96	80	**100**	100	100	91	99	93	100	70	**95**	72	**90**	78	**95**	87	**100**	100	100	100	100	96	100
1.2	Accessibility (%)	90	91	87	**100**	100	100	92	97	90	**99**	65	**100**	52	**95**	69	**98**	98	100	85	**100**	100	100	94	100
1.3	Admission process (%)	81	**100**	60	**100**	100	100	80	**100**	72	**100**	73	**95**	75	**85**	73	**93**	86	**95**	85	**97**	85	90	85	**94**
1.4	Wait for nurse/doctor (%)	81	**100**	33	**100**	100	100	71	**100**	57	**83**	85	**100**	80	**92**	74	**92**	88	**96**	67	**90**	75	**90**	77	**92**
1.5	Comfort Level (%)	85	**98**	84	**99**	100	100	90	**99**	94	97	69	**97**	71	**94**	78	**96**	87	**97**	81	**90**	40	**70**	69	**86**
1.6	Care after delivery (%)	77	**88**	78	**94**	75	**85**	77	**89**	96	100	68	**100**	69	**85**	78	**95**	95	100	80	**98**	80	**90**	85	**96**
1.7	Behavior of staffs (%)	94	96	93	100	100	100	96	99	86	**96**	55	**100**	50	**89**	64	**95**	90	**99**	96	100	90	**100**	92	100
1.8	Cleanliness (%)	60	**84**	65	**75**	48	**75**	58	**78**	94	99	83	**100**	50	**95**	76	**98**	50	**69**	100	100	25	**100**	58	**90**
1.9	Diet supplied (%)	99	100	93	**100**	100	100	97	100	51	**80**	49	**50**	50	50	50	**60**	73	**100**	33	**95**	65	**100**	57	**98**
1.10	Other facilities (%)	96	91	96	91	100	100	97	94	70	**95**	65	**100**	74	**87**	70	**94**	48	**61**	48	**55**	50	**90**	49	**69**
1.11	No payment made (%)	100	100	100	100	100	100	100	100	96	96	78	**100**	100	100	91	99	100	100	100	100	100	100	100	100
1.12	Discharge process (%)	87	**96**	77	**100**	100	100	88	**99**	72	**90**	72	**100**	69	**90**	71	**93**	92	**100**	99	100	50	**95**	80	**98**
	Pooled (%)	87	**95**	79	**97**	94	97	87	**96**	81	**95**	69	**95**	68	**88**	73	**93**	83	**93**	81	**94**	72	**94**	79	**94**
2	*Antenatal clinics, n*	122	158	77	69	48	44	247	271	149	133	108	93	29	13	286	269	84	94	49	56	14	19	147	169
2.1	Accessibility (%)	65	**95**	64	**98**	91	**100**	73	**98**	83	**91**	100	100	98	99	94	97	95	97	96	98	98	99	96	98
2.2	Time taken (%)	40	**69**	85	90	41	**92**	55	**84**	75	**85**	99	100	100	100	91	95	51	**67**	50	**70**	100	100	67	**79**
2.3	Comfortable (%)	81	**99**	70	**100**	75	**100**	75	**100**	97	100	100	100	96	100	98	100	94	100	100	100	96	100	97	100
2.4	Staff attitude (%)	82	**99**	67	**100**	99	100	83	**100**	82	**92**	88	**95**	99	100	90	96	95	100	99	100	99	100	98	100
2.5	General cleanliness (%)	62	62	70	**100**	82	**100**	71	**87**	83	**100**	74	**100**	86	**94**	81	**98**	87	**100**	100	100	86	**94**	91	98
2.6	Toilet cleanliness (%)	65	**87**	79	**100**	80	**100**	75	**96**	44	**95**	50	**100**	45	**87**	46	**94**	44	**100**	85	**92**	45	**87**	58	**93**
2.7	Other facilities (%)	99	99	89	**100**	66	**100**	85	**100**	74	**100**	91	**100**	100	100	88	**100**	86	**100**	100	100	100	100	95	100
2.8	No payment made (%)	99	100	100	100	100	100	100	100	100	100	100	100	100	100	100	100	100	100	100	100	100	100	100	100
2.9	HCP's care and response (%)	59	**100**	91	**100**	100	100	83	**100**	97	100	100	100	78	100	92	100	99	99	99	99	90	90	96	96
	Pooled (%)	72	**90**	79	**99**	82	**99**	78	**96**	82	**96**	89	**99**	89	**98**	87	**98**	83	**96**	92	95	90	**97**	88	**96**
3	*Sick newborn care (SNCU), n*	70	84					70	84	73	82					73	82	39	34	20	47			59	81
3.1	Accessibility (%)	62	**94**					62	**94**	88	89					88	**89**	97	100	71	**99**			84	**100**
3.2	Response at admission (%)	96	100					96	100	88	**98**					88	**98**	92	**100**	95	97			94	99
3.3	Care in SNCU (%)	88	**98**					88	**98**	90	90					90	90	56	**100**	95	96			76	**98**
3.4	General cleanliness (%)	59	**81**					59	**81**	60	100					60	**100**	64	**100**	100	100			82	**100**

3.5	Drinking water (%)	84	**94**					84	**94**	100	100					100	100	97	100	75	**99**			86	**100**
3.6	Clean toilets (%)	90	**100**					90	**100**	100	100					100	100	85	**100**	100	100			93	100
3.7	Shelter for caretakers (%)	75	**87**					75	**87**	43	**88**					43	**88**	96	100	100	100			98	100
3.8	No payment made (%)	98	98					98	98	100	100					100	100	71	**100**	96	100			84	**100**
3.9	Services by doctors (%)	93	**100**					93	100	100	100					100	100	98	98	100	100			99	99
3.10	Discharge process (%)	97	100					97	100	97	100					97	100	77	82	67	**100**			72	**91**
	Pooled (%)	84	**95**					84	**95**	87	**97**					87	**97**	83	**98**	90	**99**			87	**99**

Note: The figures in bold indicate the change is statistically significant (p <0.05); FRU: First referral unit; SDH: Sub-district hospital; SNCU: Sick newborn care unit
