# Supplementary material for: Improving quality of care for pregnancy, perinatal and newborn care at district and sub-district public health facilities in three districts of Haryana, India: An Implementation study
Source: PLoS One. 2021 Jul 23;16(7):e0254781. doi: 10.1371/journal.pone.0254781 (PMC8301676; doi:10.1371/journal.pone.0254781)
Supplement: S12 Table — (PDF) [file pone.0254781.s016.pdf]

S12 Table. Time spent (median and IQR in minutes) in minutes by pregnant women in antenatal clinics in Faridabad district

Parameters	Faridabad				Rewari				Jhajjar			
	Non-high risk pregnant women median time (IQR)		High risk pregnant women median time (IQR)		Non-high risk pregnant women median time (IQR)		High risk pregnant women median time (IQR)		Non-high risk pregnant women median time (IQR)		High risk pregnant women median time (IQR)	
	Baseline	Endline	Baseline	Endline	Baseline	Endline	Baseline	Endline	Baseline	Endline	Baseline	Endline
1. District hospital, observations, n	57	22	18	15	26	15	18	13	24	14	38	25
1.1 Time for registration (minutes)	24 (14-47)	15 (12-18)†	31 (16-39)	17 (14-19)†	1 (1-1)	1 (1-2)	1 (1-2)	1 (1-1)	10 (5-20)	6 (5-7)†	5 (5-16)	6 (5-7)*
1.2 Time to 1st contact-Nurse (minutes)	87 (59-157)	34 (29-39)†	104 (84-148)	48 (32-55)†	3.5 (2-11)	12 (12-14)†	10 (4-10)	8 (3-61)†	7 (5-10)	6 (5-8)*	4.5 (3-6)	4 (3.5-6)
1.3. Time to 1st contact-Doctor (minutes)	4 (3-6)	9 (4-13)†	3 (2-5)	8 (7-12)†	3 (2-4)	10 (6-15)†	2 (2-2)	4 (1-6)†	15 (8-22)	14 (8-20)	14.5 (8-19)	7 (2.5-14)†
1.4. Total time taken (minutes)	142 (87-198)	100 (76-113)†	126 (101-162)	107 (67-120)†	107 (65-211)	74 (70-171)*	150 (111-245)	70 (60-111)†	50 (35-74)	78 (62-110)†	41 (30-51)	33 (21-46)†
2. FRU 1, observations, n	16	16	8	19	15	13	6	10	18	14	12	17
2.1 Time for registration (minutes)	18 (5-22)	16 (15-19)	9.5 (6-23)	12 (10-14)	2 (1-2)	2 (1-3)	2 (2-2)	2 (2-2)	10 (5-15)	5 (0-12)†	0	0
2.2. Time to 1st contact-Nurse (minutes)	68 (42-93)	50 (46-59)†	18 (11.5-54)	34 (33-46)†	6 (2-15)	6 (3-12)	0	6 (5-7)	8 (4-15)	7 (4-15)	2 (1-2.5)	5 (5-7.5)†
2.3. Time to 1st contact-Doctor (minutes)	2 (2-3)	3 (3-5)	3.5 (2-4.5)	3 (2-4)	9.5 (3-75)	16 (6-28)	52 (38-55)	8 (6-17)†	9 (9-11)	14 (12-19)†	11 (10-13)	40 (15-80)†
2.4. Total time taken (minutes)	95 (52-145)	72 (64-86)†	31 (22-70)	53 (44-58)†	101 (64-181)	123 (123-183)*	85 (80-90)	32 (26-43)†	62 (38-102)	92 (75-137)†	73 (40-121)	125 (90-155)†
3. FRU 2, observations, n	11	17	5	16	6	10	12	11	14	15	11	13
1.1. Time for registration (minutes)	4 (3-15)	16 (10-20)†	13 (10-16)	8 (5-10)†	1.5 (1-2)	2 (1-3)	2 (1-4)	2 (1-3)	0 (0-30)	3 (2-5)†	10 (7-15)	11 (9-14)
3.2. Time to 1st contact-Nurse(minutes)	40 (18-70)	35 (29-37)*	73 (65-83)	22 (11-38)†	5 (3-10)	10.5 (4-17)*	5 (2-8)	7 (5-9)†	20 (5-35)	5 (3-9)†	5 (3-7)	2 (1-3)†
3.3. Time to 1st contact-Doctor (minutes)	3 (2-5)	5 (3-7)†	12 (11-13)	7.5 (6-8)†	5.5 (2-49)	4 (2-8)*	55 (42-62)	9 (6-11)†	10 (8-11)	20 (12-32)†	10 (8-12)	3 (2-4)†
3.4. Total time taken (minutes)	79 (43-88)	56 (46-61) †	123 (113-133)	52 (34-63)†	87 (60-101)	60 (30-96)*	90 (70-112)	48 (34-60)†	135 (70-195)	40 (25-80)†	75 (55-90)	72 (62-82)

*Notes: * Indicate the change is statistically significant (p<0.05); † Indicate the change is statistically significant (p<0.01)*
