# Supplementary material for: Improving quality of care for pregnancy, perinatal and newborn care at district and sub-district public health facilities in three districts of Haryana, India: An Implementation study
Source: PLoS One. 2021 Jul 23;16(7):e0254781. doi: 10.1371/journal.pone.0254781 (PMC8301676; doi:10.1371/journal.pone.0254781)
Supplement: S13 Table — (PDF) [file pone.0254781.s017.pdf]

S13 Table. Change in knowledge and skill status of the care providers in labour room and sick newborn care units

Sl.no	Domains	Faridabad		Rewari		Jhajjar		Pooled	
	Cycle	Baseline	Endline	Baseline	Endline	Baseline	Endline	Baseline	Endline
1	*Labour rooms and postnatal wards (Delivery and essential newborn care)*								
	Respondents (n)	22	24	20	22	22	24	64	70
1.1	Delivery (%)	48	71	34	40	46	71	43	61
1.2	Essential newborn care (%)	77	92	72	75	86	90	78	86
1.3	Resuscitation (%)	48	71	42	63	48	77	46	70
1.4	Infection control (%)	68	86	60	63	53	78	60	76
1.5	Sub-total delivery and essential newborn care (%)	60	80	52	60	58	79	57	73
2	*Sick newborn care units (essential and sick newborn care))*								
	Respondents (n)	8	8	8	8	12	12	28	28
2.1	Supportive care (%)	58	79	54	65	65	74	59	73
2.2	Temperature (%)	40	74	23	34	43	65	35	58
2.3	Infection control (%)	83	90	59	68	78	95	73	84
2.4	Resuscitation (%)	39	83	25	33	36	67	33	61
2.5	Feeding (%)	52	74	58	61	39	81	50	72
2.6	Case management (%)	63	82	38	50	66	86	56	73
2.7	Intravenous fluid and medication (%)	42	76	88	90	52	81	61	82
2.8	Sub-total essential and sick newborn care (%)	54	80	49	57	54	78	52	72
