# Supplementary material for: Improving quality of care for pregnancy, perinatal and newborn care at district and sub-district public health facilities in three districts of Haryana, India: An Implementation study
Source: PLoS One. 2021 Jul 23;16(7):e0254781. doi: 10.1371/journal.pone.0254781 (PMC8301676; doi:10.1371/journal.pone.0254781)
Supplement: S2 File — (PDF) [file pone.0254781.s019.pdf]

ID: 1108101	
**1.1 Type of Health Facility dh, fbd**	**1.2 Designation: MO**

2. General

**2.1**	How long have you been working in this health facility? (months/years)	8 years in total
**2.2**	Total months/years of service	18 years of total exp
**2.3**	What are your current roles and responsibility with respect to maternal and neonatal care? I am appointed here for NICU through NHM. I also look for administrative work, do rounds at causality, pediatric ward, attend C/S and OPD.	
**2.4**	How many deliveries and resuscitations of newborns have you attended in last 1 month?	
	A. No. of deliveries attended in last 1 month	120 Cs
	B. No. of newborn resuscitations attended in last 1 month	15

3. Service Delivery

3.1 In routine practice, which health staff performs the following services?

Services	Staff performing the services
Delivery without complication	-
Delivery with complication/ high risk delivery	-
Caesarean section	Attend Cs
Newborn care at birth	Doctor for Cs; staff nurse for normal newborn
Sick newborn care	Doctor
Breastfeeding support	Staff nurse under supervision of doctor

3.2 What are the challenges faced by you and your colleagues for delivering the desired mother and newborn care services?

Challenges faced	How do you manage these challenges
Infrastructure	No regular water supply; AC is not working
Equipment	Some warmers are not working. If equipments are not working, it takes time to repair.
Drugs and supplies	Do not get medicines on time. Bleaching solution, bacilloid are out of stock.
Support services	Occasionally, ambulance gets late. I am not sure if EMT goes along with referred patient or not.
Other	No small ambu bags in ambulance. No system of temperature maintenance. Newborns are not sent through casuality. They should at least do initial examination.

3.3 What challenges do you face while delivering essential newborn care services and how do you manage these?

Challenges faced	How do you manage these challenges
Care at delivery	
Care in the ward Do not take round because of shortage of doctor.	No one watch the baby. Mothers bring their baby to NICU for checkup at scheduled time.
Care of sick newborns No problem in NICU. Problem occurs when no doctor is present.	Staff nurse manage everything and give immediate care.

3.4 What challenges do you face while delivery of pregnant women?

Challenges faced	How do you manage these challenges
Delivery without complication	NA

	Delivery with complication	NA
	Caesarean section	NA
	Referred cases with complication	NA
**3.5**	How long usually the mothers stay at the facility after the delivery?	
	Normal Delivery	NA
	Caesarean Delivery	NA

**4. Manpower**	
**4.1**	How many positions of doctors are lying vacant in your health facility?<br>Post of 4 MO's is vacant. Currently, we are 4 doctors; 1 from NHM and 3 from HCMS, 1 MO from HCMS.
**4.2**	If there is a shortage of manpower who addresses the issue so that is does not hinders routine work?<br>We raise the issue in front of PMO & CMO. Usually addresses the issue but if she is on night shift then anyone can go. It takes time to meet CMO and we have to wait there.
**4.3**	Do you have adequate staff inside labor room, ANC clinic and SNCU's?<br>4 staff nurse are short and post for 4 MO's are short. We need to increase position of staff nurse. 1 post is vacant, 3 are shifted to other departments. We do not have any staff to manage step down.
**4.4**	What happens if a particular department is having more flow of patients? Is there any flexibility in assigning inter departmental responsibilities among staff?<br>2 doctors are posted at a time, so we manage by personal understanding only if free. But not from other department.
**4.5**	What is the mechanism of taking leave and who sanctions it?<br>We give application to PMO. He only sanctions the leave. 20 CLs are granted to us. We cannot take leave during shortage of staff.
**5. Duty Roster**	
**5.1**	Who prepares the duty roster for you?<br>Most of the times, Dr. prepares the roster for us. We keep a printout of it.
**5.2**	Who follows up the prepared roster so that the shifts are routinely changed?<br>It is always in rotation. Usually MO takes night shift but no MO is posted at present, so we manage and change the shifts regularly.
**5.3**	How many Medical Officers are posted at one time in your department? What is the pattern of shift?<br>Presently no MO is posted. Post id vacant for 4 MO's.
**5.4**	Do you have flexibility in changing the shifts?<br>Yes, we do have flexibility of changing shifts by mutual understanding if other doctor agrees.
**5.5**	How do you manage when you have double shifts?<br>Nowadays, we are doing double shifts. Normal timings are 8-2 PM but we stay till 3 PM. Rest of the time we are available on call till 5 PM. After 5 PM, the doctor posted for night shift is available on call till 8 PM.
**6. Infrastructure**	
**6.1**	Do you have space to accommodate changes inside the department?<br>Yes, we have lot of space but we never thought of doing any changes.
**6.2**	Do you have enough beds to accommodate increased number of patients?<br>No, we usually do doubling and tripling of babies. Already 2 warmers are extra.

**6.3**	Is their regular power supply and clean water for drinking? Any substitute available in case of power cut or irregular water supply?<br>Usually there is no issue of power cut as we have generator back up. We do not have facility of clean drinking water. We have to get water from our home.
**7. Training /Skills**	
**7.1**	How many of the total staff are trained for MCH services?<br>NSSK and NRP training is given to all the staff. 2 staff are left for FBNC.
**7.2**	Is there any pre job posting training for newly joined staff?<br>No, there is no separate training for newly joined staff. We give 15 days' cover duty in morning with senior staff. Independent charge is given after they attain confidence.
**7.3**	Is there any on- job training for the staff?<br>Yes, regular trainings are scheduled for all staff.
**7.4**	Please let us know about the last training on attended by you?<br>Ventilator & CPAP workshop in Delhi.
	Timing (Month/Year): October 2015
	Place: New Delhi
	Duration (in days): 2 days
	What did you like the most in the training?<br>We get leave from work and we get to know new things. Get revision of previously learnt things.
	What did you dislike the most in the training?<br>If nothing new is learnt. If I am already doing something then why there is need of training. Ventilators are not present here so there is no point of conducting such trainings.
	What was the training methodology used (Lectures/ Hands-on / Practical's)?<br>PPT's, Lectures, Practical, on mannequin, ask us to demonstrate.
	Who conducts the workshop? Who prepares roster for workshop/training and how it is notified? How it is monitored?<br>District training centre prepares roster for us. We get notified by mail/letter from PMO. They only monitor from district training centre about attendance.
**7.5**	What are the opportunities and mechanisms currently in place/adopted to retain the skills of Nurses/ANMs/Doctors?<br>Trainings are only way to retain skills otherwise no other mechanism. We ourselves can take initiatives.
**7.6**	How the training related to care during delivery and newborn period can be further improved?<br>Training sessions should be more interactive, interesting and to the point. They should first know about our resources and equipments. They should not give us training for using equipments which we do not have in our hospitals or which we are not using regularly. If equipments are in stock then it should be used.
**7.7**	Have you ever visited/attended the skill labs operational in your district? Where it was conducted? Who conducted it? What was the time duration of skill lab training?<br>No, I never attended skill lab but I might get notification soon. Currently our staff nurse is on training for skill lab. Rest all staff nurse already received skill lab training.
**7.8**	What are the good things about this skill lab?
**7.9**	What are the challenges related to skill lab?
**7.10**	In your opinion, how many health staffs might have used or visited the skill labs?<br>All staff nurse visited skill lab. None of the doctor had attended/ called for skill lab.

**7.11**	Did somebody advise or persuade you to attend the skill lab?
**8. Referral services**	
**8.1**	In what situation usually the newborns/mothers (pregnant/recently delivered) are referred to the next level of healthcare?<br>We refer the patient if they are on ventilator, SPO2 not maintained, for pediatric surgery, severely sick, repeated seizures or if patient fights or ask for referral.
**8.2**	Where the newborns/ pregnant women/ mothers are usually referred, what is the usual mode of transportation and how long does it takes to reach the next level health facility in your area?<br>We refer all patients to safdarjung hospital. Ambulance is always available for transfer.
**8.3**	What facilitation is done from facility side for referral and what difficulties/challenges do you face while transporting the sick newborn and mother to next level? *(Probe: monetary/logistics)*<br>We do not refer to kalawati now as they do not give reimbursement of petrol for ambulance.
**8.4**	What are the challenges faced related to referral transport experienced by this facility and how are they handled?<br>Patients usually do not want to go to safdarjung. It is difficult to convince them. Some patients return back due to overcrowding. Sometimes ventilators are not available there.
**9. Logistics**	
**9.1**	Are you aware of any shortage/irregular supply of drugs and/or supplies needed for care during delivery and newborn period in the last one year? What were the reasons for this shortage and how these situations were managed?<br>Some antibiotics are short like piperacillin, meropenem, vancomycin since last 1 year. Ceftriaxone is available because other departments use it. They give same medicines to us. Phenobarbitone, dopamine, adrenaline are also out of stock.
**9.2**	How frequently the families/ parents asked to procure drugs from outside/ store?<br>We ask to get all higher antibiotics from outside on daily basis. Some investigations are also not available here. So we ask patients to get it from outside.
**9.3**	How many equipments essential for management of delivery or newborn care are out of order at this moment?<br>8 warmers at step down and 2 warmers in out-born unit are not working. 4-5 monitors are also not functional.
**9.4**	What is the usual mechanism of repair and maintenance of these equipments? *(probe: who is responsible and what is the duration of repair*)<br>Sister in-charge write letter to PMO. He informs the biomedical engineer. They call concerned company for repair. Duration is not fixed. It varies from 15 days to 6 months.
**9.5**	What are the supervisory mechanisms in place at present for maternal and newborn care services?
	Who supervises<br>PMO, NHM
	What is the frequency of supervisory visits<br>Monthly visits for 1-2 days. No sudden visits.
	Is any feedback/report provided usually after the supervision?<br>Everything is set for 1-2 days. We do double shifts. There is no feedback or reporting mechanism.
	What actions are taken after last supervisory visit?

	Usually nothing is done.
**9.6**	Please let us know about the last supervisory visit to the facility related to maternal and newborn care services?
	Who came for last supervisory visit?<br>15 days back from NHM.
	How long ago the supervisory visit took place?<br>15 days back with PMO and CMO.
	What all components were observed?<br>NICU and mother side were observed.
	What feedback was given and what actions were taken?<br>No action/feedback was given. No point of doing supervision. All reports are piling up on tables only.
**10. Perceptions regarding Quality of care**	
**10.1**	According to you, what is the meaning of quality?<br>Patient should get prompt care, prompt response. All medicines and equipments should be available every time.
**10.2**	According to you, what are the issues that affect the quality of health services?<br>Staff shortage is a big reason. Without doctor, nothing is possible.
**10.3**	What can you do to improve the quality of the health services?<br>We can only sit here. Doctors are already doing their best.
**10.4**	Did any of your relatives, friends or acquaintances ever availed health services at this hospital? If not, any reason?<br>No, none of my relatives/friends availed health services from this hospital. Also, my relatives stay too far as I belong to Maharashtra.
**11. Others**	
11.1	If any shortage of blood and how is it tackled?<br>We need pediatric blood band like 100 ml, 30 ml. they are not available here. Usually there is no shortage of blood but wastage can be seen
11.2	Do you arrange blood donation camps on facility basis? Yes, through blood banks.

ID: 1108103			
**1.1 Type of Health Facility- DH fbd**		**1.2 Designation- MO**	
**2. General**			
**2.1**	How long have you been working in this health facility? (months/years)	4 years	
**2.2**	Total months/years of service	26 years	
**2.3**	What are your current roles and responsibility with respect to maternal and neonatal care? SNCU Pediatrician in charge, Administration, OPD, Nursery and all clinical.		
**2.4**	How many deliveries and resuscitations of newborns have you attended in last 1 month?		
	A. No. of deliveries attended in last 1 month	Only Caesarian section- 35	
	B. No. of newborn resuscitations attended in last 1 month	12-13	
**3. Service Delivery**			
**3.1**	In routine practice, which health staff performs the following services?		
	**Services**	**Staff performing the services**	
	Delivery without complication	NA	
	Delivery with complication/ high risk delivery	NA	
	Caesarean section	NA	
	Newborn care at birth	LR/ SNCU	
	Sick newborn care	SNCU Doctors and staff nurse	
	Breastfeeding support	For all sick newborn, class 4/ sweeper	
**3.2**	What are the challenges faced by you and your colleagues for delivering the desired mother and newborn care services?		
	**Challenges faced**	**How do you manage these challenges**	
	Infrastructure: there is shortage of manpower and staff nurse shortage. There is no supply of mycochloride and bacilloids at SNCU.	I made a register for regular update, but class 4 workers don't take it seriously and staff nurses also don't care about it.	
	Equipment; Boiler is not working, maintenance is very poor.	Nursing in charge does not work properly. They don't clean NICU in morning shift.	
	Drugs and supplies; Medicines are not available. PMO had purchased it but bio medical engineer said that pharmacist doesn't supply.	Patients purchase from outside,	
	Support services; Quality of bleaching powder is very poor; there is no supply of baciloacid and sodium hypochloride.		
	Other; Inadequate supply of drug, There is no any training conduction provision of staff. Demands are being sent regularly but there is no supply.	Patients purchase medicine and injection from outside.	
**3.3**	What challenges do you face while delivering essential newborn care services and how do you manage these?		
	**Challenges faced**	**How do you manage these challenges**	
	Care at delivery It is manageable, once baby cry.		

	Care in the ward	
	Care of sick newborn: Mostly Outborn babys come with complaints of cord sepsis, their parents don’t come with their baby, and sometimes they tell us wrong identity	
**3.4**	What challenges do you face while delivery of pregnant women?	
	**Challenges faced**	**How do you manage these challenges**
	Delivery without complication	NA
	Delivery with complication	NA
	Caesarean section	NA
	Referred cases with complication	NA
**3.5**	How long usually the mothers stay at the facility after the delivery?	
	Normal Delivery	NA
	Caesarean Delivery	NA
**4. Manpower**		
**4.1**	How many positions of doctors are lying vacant in your health facility?<br>There is shortage of 4 to 5 doctor. One pediatrician and	
**4.2**	If there is a shortage of manpower who addresses the issue so that is does not hinders routine work?<br>We write a letter to PMO, then PMO conveys to CMO.	
**4.3**	Do you have adequate staff inside labor room, ANC clinic and SNCU’s?<br>No, there are no dedicated staff nurses inside SNCU, At least 11 to 12 staff nurses should appoint at SNCU. At present only 6 staff nurses are available.	
**4.4**	What happens if a particular department is having more flow of patients? Is there any flexibility in assigning inter departmental responsibilities among staff?<br>We don’t have any option, We do it alone. Numbers of class 4 workers should increase. Inter departmental rarely help into this.	
**4.5**	What is the mechanism of taking leave and who sanctions it?<br>I fill application of CL ,I can take 12 Casual leave but mostly cmo sanctions 8 -10 CL.	
**5. Duty Roster**		
**5.1**	Who prepares the duty roster for you?<br>I prepare the duty roaster.	
**5.2**	Who follows up the prepared roster so that the shifts are routinely changed?<br>I do it.	
**5.3**	How many Medical Officers are posted at one time in your department? What is the pattern of shift?<br>There No medical officer here. I send require regularly but they don’t take care of it.<br>Dependant on staff nurse.	
**5.4**	Do you have flexibility in changing the shifts?<br>Yes, we have flexibility in changing shifts.	
**5.5**	How do you manage when you have double shifts?	
**6. Infrastructure**		
**6.1**	Do you have space to accommodate changes inside the department?<br>Firstly, we need adequate supply. And there is drainage problem at NICU. Fumigator is not	

	working, was basin is not in good condition.
**6.2**	Do you have enough beds to accommodate increased number of patients?<br>Number of patients is increase in winter season. Sometime we have to shift 3 babies over one bed.
**6.3**	Is their regular power supply and clean water for drinking? Any substitute available in case of power cut or irregular water supply?<br>Yes, we have generators for regular power supply. RO is installed but not maintained.
**7. Training /Skills**	
**7.1**	How many of the total staff are trained for MCH services?<br>Every staffs are trained, and they are also trained in FIMNCI.
**7.2**	Is there any pre job posting training for newly joined staff?
**7.3**	Is there any on- job training for the staff?<br>DTC send notice for job training.
**7.4**	Please let us know about the last training on attended by you?<br>Last year, I attended TOT F- IMNCI.
	Timing (Month/Year): 2016 September
	Place: Panchkula
	Duration (in days): 5 Days.
	What did you like the most in the training?
	What did you dislike the most in the training?
	What was the training methodology used (Lectures/ Hands-on / Practical's)?<br>Lectures, videos, pre and post test, Group discussion and hands on.
	Who conducts the workshop? Who prepares roster for workshop/training and how it is notified? How it is monitored?<br>DTC notify us, I don't have more idea about it.
**7.5**	What are the opportunities and mechanisms currently in place/adopted to retain the skills of Nurses/ANMs/Doctors?<br>SBA training for all the staff nurses, How to maintain Hygiene Practice at NICU, How to autoclave instruments and sterilization process. How to clean Radiant warmer.
**7.6**	How the training related to care during delivery and newborn period can be further improved?
**7.7**	Have you ever visited/attended the skill labs operational in your district? Where it was conducted? Who conducted it? What was the time duration of skill lab training?<br>I didn't attend.
**7.8**	What are the good things about this skill lab?<br>I don't have fair idea about it as I didn't take training.
**7.9**	What are the challenges related to skill lab?<br>NA.
**7.10**	In your opinion, how many health staffs might have used or visited the skill labs?<br>All staff nurses from SNCU should visit the skill labs.
**7.11**	Did somebody advise or persuade you to attend the skill lab?<br>Not yet.
**8. Referral services**	

**8.1**	In what situation usually the newborns/mothers (pregnant/recently delivered) are referred to the next level of healthcare?<br>In case of severe sepsis, extremely LBW baby, severe birth asphyxia, uncontrolled seizers and Where ventilation is required in that condition we refer patients to higher facility.
**8.2**	Where the newborns/ pregnant women/ mothers are usually referred, what is the usual mode of transportation and how long does it takes to reach the next level health facility in your area?<br>Safdarjung, mode of transportation is ambulance.
**8.3**	What facilitation is done from facility side for referral and what difficulties/challenges do you face while transporting the sick newborn and mother to next level? *(Probe: monetary/logistics)*
**8.4**	What are the challenges faced related to referral transport experienced by this facility and how are they handled?
**9. Logistics**	
**9.1**	Are you aware of any shortage/irregular supply of drugs and/or supplies needed for care during delivery and newborn period in the last one year? What were the reasons for this shortage and how these situations were managed?<br>Yes, Injection piperacillin and vancomycin are not available since long time. We do not know about reason for shortage. We ask patients to get it from outside.
**9.2**	How frequently the families/ parents asked to procure drugs from outside/ store?<br>Only for inj piperacillin and inj vancomycin if required for any baby, otherwise we ask every patients to get 2 packets of huggies from outside. We also ask patients to purchase soap because liquid soap is not good which we get from hospital.
**9.3**	How many equipments essential for management of delivery or newborn care are out of order at this moment?<br>Step down room is not functional due to shortage of staff. 8 warmers are functional but not in use. Rest equipments are functional.
**9.4**	What is the usual mechanism of repair and maintenance of these equipments? *(probe: who is responsible and what is the duration of repair*)<br>We call bio medical engineer for repairing. It depends upon problem that how long they take to repair<br>It. Usually it takes from 1 week to 3 to 4 months.
**9.5**	What are the supervisory mechanisms in place at present for maternal and newborn care services?
	Who supervises<br>PMO and CMO monitors.
	What is the frequency of supervisory visits<br>They visit once in a month.
	Is any feedback/report provided usually after the supervision?<br>There is no any proper format, but they discuss the issues in meeting.
	What actions are taken after last supervisory visit?<br>No, but staff get scolded if any serious issue reported.
**9.6**	Please let us know about the last supervisory visit to the facility related to maternal and newborn care services?
	Who came for last supervisory visit?<br>PMO visited last week.
	How long ago the supervisory visit took place?

	He came for 10-15 minutes and spoke to patients.
	What all components were observed?<br>Mother side ward, SNCU then he went toward LR and other parts of hospital.
	What feedback was given and what actions were taken?<br>He scolded staffs for cleanliness as it was not maintained. No action taken.
**10. Perceptions regarding Quality of care**	
**10.1**	According to you, what is the meaning of quality?
**10.2**	According to you, what are the issues that affect the quality of health services?<br>Man power issues and there is no MO appointed here.
**10.3**	What can you do to improve the quality of the health services?
**10.4**	Did any of your relatives, friends or acquaintances ever availed health services at this hospital? If not, any reason?<br>No, there is no guarantee for proper care.
**11. Others**	
**11.1**	If any shortage of blood and how is it tackled?<br>We refer patients to Blood bank in case of unavailability they arrange blood from private blood bank.
**11.2**	Do you arrange blood donation camps on facility basis?<br>NO.

ID: 1108201		
**1.1 Type of Health Facility: DH, FBD**		**1.2 Designation: Staff nurse**
**2. General**		
**2.1**	How long have you been working in this health facility? (months/years)<br>I am working in this hospital since august 2012 under NHM.	
**2.2**	Total months/years of service<br>My total work experience in 8 years. I joined in May 2009 in private sector.	
**2.3**	What are your current roles and responsibility with respect to maternal and neonatal care?<br>My current job involve nursing care for babies and giving medicines, maintaining records of SNCU, maintaining registers and making indent for medicines.	
**2.4**	How many deliveries and resuscitations of newborns have you attended in last 1 month?	
	A. No. of deliveries attended in last 1 month	NA
	B. No. of newborn resuscitations attended in last 1 month	3-4
**2.5**	Who did you receive the training from:	

	Area	Name of Trainning	Year
A	Care during delivery (S.B.A.)	SBA	2015
B	Neonatal Resuscitation Program (In RP / NSS)	NSSK; GOLDEN 1 MINUTE	2016; 2013
C	Sick Newborn Care (FBNC)	FBNC	2013

3. Service Delivery

3.1 What are the challenges faced by you and your colleagues for delivering the desired mother and newborn care services?

Challenges faced

	Mother care	Newborn care
Infrastructure	NA	No issues
Equipment	NA	All equipments are working. If any instrument is nonfunctional then it takes 15-30 days to repair it. If it need to get repair from outside then it can take more than 30 days as well.
Drugs and supplies	NA	Some injections are not available since long time like Inj Piperacin, merobilum, vancomycin.
Support services	NA	Ambulance is not available instantly. Sometime patients have to wait for more than half an hour. At the time of shift change, 1st driver leave before time and the next person come late. In this meantime, no driver is available.
Other	NA	Due to shortage of staff it is difficult to resuscitate and simultaneously look for other babies as there is only one staff nurse in evening and night shift.

3.2 What challenges do you face while delivering essential newborn care services and how do you manage these?

Challenges faced	How do you manage these challenges
Care at delivery	NA
Care in the ward	NA
Care of sick newborns: Due to shortage of staff, it is difficult to	We take help from class IV. We trained all class IV for assist in case of emergency or if patient load is more.

	resuscitate and manage other patient simultaneously.	
**3.3**	What challenges do you face while delivery of pregnant women?	
	**Challenges faced**	**How do you manage these challenges**
	Delivery without complication	NA
	Delivery with complication	NA
	Caesarean section	NA
	Referred cases with complication	NA
**3.4**	How long usually the mothers stay at the facility after the delivery?	
	Normal Delivery	NA
	Caesarean Delivery	NA
**4. Manpower**		
**4.1**	How many posts of Staff Nurse / ANM are vacant in your health facility?<br>Sanctioned post for SNCU is 10 but only 6 are posted here at present. 1 nurse left the job whereas other 3 are posted in other departments. 1 is posted in medicine department and other 2 are posted in casualty. Post should increase as there are more number of patients.	
**4.2**	What difficulties do you face in providing mother and newborn care services to existing employees? (Doctors, nurses and other staff)<br>No issues with any staff, doctor or class IV. Everyone is very cooperative.	
**4.3**	What is the mechanism of taking leave and who sanctions it?<br>We give cover duty to new staff for 1 month. Usually they get posted in morning hours as more number of patients visit during this time and even staff strength is good to help them. If some experienced person joins then we give them independent posting early in comparison to new staff. Independent posting is given once the staff gains confidence of working alone.	
**5. Duty Roster**		
**5.1**	Who prepares the duty roster for you?<br>All staff nurse of SNCU have to prepare duty roster on shift basis because there is no nursing sister for our department. One of us is made incharge for a month to prepare roster for everyone and the charge is shifted to other in next month.	
**5.2**	Do you have flexibility in changing the shifts?<br>Yes, we do have flexibility but doctor on duty sanction it.	
**5.3**	How do you manage when you have double shifts?<br>It rarely happened but if needed then only male staff (Kartar) do the double shift. We get 1 extra day off after doing 1 double shift. There is another provision of 1 extra shift from 10-4 PM simultaneously with normal shifts. We get a good support with this shift.	
**5.4**	What is the procedure for taking leave and who approves it?<br>We have to do little buttering to dr. vinny and she permit us. We change with other staff with mutual adjustments but it is accepted by dr. vinny.	
**5.5**	Who prepares rosters for emergency / regular service?<br>There is no separate roster for emergency duty. If needed, we have to work extra.	
**6. Infrastructure**		
**6.1**	Do you have space to accommodate changes inside the department?<br>Yes, we have lot of space to make changes in the department.	
**6.2**	Do you have enough beds to accommodate increased number of patients?<br>In case of more number of patients, we do doubling or tripling of babies on 1 radiant warmer. If the baby is stable then we shift the baby to mother side.	
**6.3**	Is their regular power supply and clean water for drinking? Any substitute available in case of power cut or irregular water supply?<br>There is no problem of power cut and we have generators for power back up but clean drinking water is not available. We get it from our home or else we have to buy from paid water cooler which is located downstairs in hospital campus.	

.

7. Data management		
**7.1**	How do you record data?<br>All the data is recorded on register. We have total 12 registers. They are admission, report, refer, death, o2, crash card, round book, complaint, demand, indent, dispatch and stock register.	
**7.2**	How do you maintain a register?<br>We update all the registers on daily basis and send it to staff.	
**7.3**	Where do you send the record?<br>Yearly records are send to store room whereas HMIS data is sent to staff.	
**7.4**	How often is the data sent?<br>Morning and evening staff update on same day whereas staff on night shift update the data on next day.	
**8. Blood bank**		
**8.1**	How long does it take for a needy person to get blood?<br>Blood can be arranged within 1 hour if it is available in blood bank otherwise patient have to arrange it from private blood bank and it usually takes time.	
**9. Training /Skills**		
**9.1**	Could you tell us about your previous NSSK / Neonatal Resuscitation Training?	
	Time (month / year)	2015
	place	District training centre, DI office
	Duration (in days)	2 days
**9.2**	Who conducts the workshop? Who prepares roster for workshop/training and how it is notified? How it is monitored?<br>We get notification from DI office. First they ask for any volunteers but finally they make according to their list only.	
**9.3**	What did you like the most in the training?<br>Best thing is that we get relief from ward posting and get a day off. We get to do practical over dummies. We get a change and new learning experience.	
**9.4**	What did you dislike the most in the training?<br>Everything is good. There is nothing to dislike about the training.	
**9.5**	what was the training methodology used (Lectures/ Hands-on / Practical's)?<br>Training is done through lectures and powerpoint slides, practical over dummies, group discussions, role play etc. there is one pre and post training test as well. Sometime they display videos and finally they take group photographs.	
**9.6**	How did you like meeting / interacting with the trainers?<br>Now all the trainers are new and they are fine. Previous trainers were good. They used to teach according to our level only. Nurses teach better than doctors because all doctors strart teaching according to their level.	
**9.7**	What was their level of knowledge / skills?	It is good. They do cramming of the subject before training.
	How was your conversation with him?	It is good. All trainers speak nicely.
**9.8**	What are the opportunities and mechanisms currently in place/adopted to retain the skills of Nurses/ANMs/Doctors?<br>They should give us demo in ward because there is difference between theoretical knowledge and its practical implementation. We face real time challenges while doing practically. It will help us in gaining more skills.	
**9.9**	What challenges do you have with the skills of nurses and the support of staff in delivery rooms, perinatal wards and newborn care units? In your opinion, how can this be controlled?<br>No problem at all.	

**9.10**	How the training related to care during delivery and newborn period can be further improved?<br>Everything is good. If possible then we should be trained over patient in the ward.
**9.11**	Have you been to a skill lab set up in your district?<br>Yes, I have been to skill lab for training.
**9.12**	What are the good things about this skill lab?<br>Maintenance of skill lab is good. They demonstrate very nicely at skill lab.
**9.13**	What are the challenges related to skill lab?<br>They should also teach us on patient. The equipments they show us and give training are not available with us hence we cannot follow what we learn from there. Sometime we even forget the name of instrument which we see there.
**9.14**	In your opinion, how many health staffs might have used or visited the skill labs?<br>1 staff nurse (Madhu) is currently on training for skill lab. All other staff nurses have been to skill lab training.
**9.15**	Did somebody advise or persuade you to attend the skill lab?<br>We get notification from DI office. They only decide about the staff which will get training.
**9.16**	How does the Skill Lab help in Neonatal Resuscitation and Neonatal Care?<br>We already knew everything about newborn resuscitation and care of newborn. Nothing new was learnt. it was good in gaining knowledge about delivery patients. It will definitely help the staff posted in gynae department.
**10. Referral services**	
**10.1**	In what situation usually the newborns/mothers (pregnant/recently delivered) are referred to the next level of healthcare?<br>We refer newborn who require ventilator support or if they are getting continuous seizures. We initially give them phenobarbitone but cannot repeat it for long so we prefer them to refer to higher facility.
**10.2**	Where the newborns/ pregnant women/ mothers are usually referred, what is the usual mode of transportation and how long does it takes to reach the next level health facility in your area?<br>We always refer the patient to safdarjung hospital in delhi. Ambulance from hospital take the patient. It takes about 45 minutes to reach there.
**10.3**	What facilitation is done from facility side for referral and what difficulties/challenges do you face while transporting the sick newborn and mother to next level? *(Probe: monetary/logistics)*<br>EMT accompanies the patient till safdarjung hospital. He provide all care needed during transfer.
**10.4**	What are the challenges faced related to referral transport experienced by this facility and how are they handled?<br>Patient do not get convince easily to go to safdarjung. We have to make lot of efforts to convince them. Some patient prefer to go to private hospital because they feel good care is not provided at safdarjung hospital.
**11. Logistics**	
**11.1**	Are you familiar with any scarcity / irregular supply of medicines and / or supplies required for care during delivery and newborn in the last one year? What were the reasons for this deficiency and how were these conditions managed?<br>Some medicines are not available. We ask patients to get it from outside. We also do local purchase of some medicines.
**11.2**	How frequently the families/ parents asked to procure drugs from outside/ store?<br>Patients get injections and pampers from outside. Almost every patient have to get these from outside. We try to stabilize the baby with cefotaxime but if needed then we have to go for higher antibiotics. In that case patient have to get injections like vancomycin and piperamycin from outside.
**11.3**	What are the supervisory mechanisms in place at present for maternal and newborn care services?<br>Yes, regular supervision takes place in hospital.

.

	Who supervises<br>Usually PMO and CMO do regular visits. Occasionally NHM team comes for visit. Once NQAS team came for visit.
	What is the frequency of supervisory visits<br>They can come anytime. Nothing is fixed.
	Is any feedback/report provided usually after the supervision?<br>All reports are made at official level. We do not have much idea about it.
	What actions are taken after last supervisory visit?<br>NHM team visited last week. They scolded us because cleanliness was not upto the mark.
**11.4**	Please let us know about the last supervisory visit to the facility related to maternal and newborn care services?
	Who came for last supervisory visit?<br>Last week by NHM team. I do not know name of the officials.
	How long ago the supervisory visit took place?<br>The visit lasted for 1-1.5 hours.
	What all components were observed?<br>They came to SNCU and mother side ward. After that they went to labor room and PNC ward. They spoke to patients as well and took there feedback.
	What feedback was given and what actions were taken?<br>They asked patients about any problems which they are facing and later they discuss them with the doctors.
**12. others**	
**12.1**	In your view, what are the perceived barriers between families in the use of public health services for newborns of pregnant women?<br>Discharge slip is same as admission file. We give all information of admission details, treatment done and advice to the patient.
**12.2**	In your view, what are the perceived barriers between families in the use of public health services for newborns of pregnant women?<br>Parents feel that we do not allow to visit the baby intentionally neither we update them about status of the baby. But reality is that workload is more and number of staff nurse are less. Hence we face problems in dealing with patient everytime.<br>How can these obstacles be overcome?<br>We try to convince them. If they shout or misbehave then we call security guard and sometime even call police.
**12.3**	How much additional effort is needed to reduce the neonatal mortality rate in your area?<br>There should be 1 baby on each warmer. We should think of solution for arranging extra patients. Scarcity of staff should be fulfilled. We face problem while resuscitating a patient. Cannot manage everything alone.
**12.4**	According to you, what is the meaning of quality?<br>Better services will lead to better quality. We should do our work in better way.
**12.5**	What can you do to improve the quality of the health services?<br>She should stop attendants to frequently visit the patient as they bring infection with them. Doubling of baby should stop and more staff should be employed to provide services.
**12.6**	What can you do to improve the quality of the health services?<br>There should be regular supply of drugs and material. Baby should be given sponge in NICU.
**12.7**	Did any of your relatives, friends or acquaintances ever availed health services at this hospital? If not, any reason?<br>My relatives and friends come if they need any treatment in nursery but I never advise them to visit other departments specially labor room because of aseptic conditions.

.

ID: 1108202		
**1.1 Type of Health Facility : DH, FBD**		**1.2 Designation: STAFF NURSE**
**1.2 General**		

**2.1**	How long have you been working in this health facility? (months/years)<br>I joined here in December 2013. In total, I am here since last 4 years	
**2.2**	Total months/years of service<br>My total work experience is 10 years. Earlier I worked in private sector hospitals	
**2.3**	What are your current roles and responsibility with respect to maternal and neonatal care?<br>My current responsibilities includes conducting deliveries, manage bleeding during labor, admit the sick babies to nursery, all routine care of mother and newborn during normal deliveries, give medicines to patients in ward, give advice to patient during discharge.	
**2.4**	How many deliveries and resuscitations of newborns have you attended in last 1 month?	
	A. No. of deliveries attended in last 1 month	10
	B. No. of newborn resuscitations attended in last 1 month	None

2.5 Who did you receive the training from:

	Area	Training name	Year
A	Care during delivery (S.B.A.)	SBA	2009
B	Neonatal Resuscitation Program (In RP / NSS)	No	
C	Sick Newborn Care (FBNC)	No	

3. Service Delivery

3. Challenges faced

3.1 What are the challenges faced by you and your colleagues for delivering the desired mother and newborn care services?

Challenges faced

	**Mother care**	**Newborn care**
Infrastructure	No problem related to infrastructure.	There are no issues.
Equipment	Equipments are available and functional. Some patient comes to us without ultrasound and previous reports. That time we face problem in managing.	No issues
Drugs and supplies	It rarely happens that we have shortage of medicines. Sometimes we do not get sinto gel. Rest all medicines are available.	We get all medicines and vaccines on time. No issues in this.
Support services	No issues	Earlier used to feed babies but now we have to do it.
Other	No issues	No issues

3.2 What challenges do you face while delivering essential newborn care services and how do you manage these?

**Challenges faced**	**How do you manage these challenges**

	Care at delivery: Patients do not cooperate with us. They are always in hurry to get discharge and leave for home. Sometimes they misbehave as well.	We tell their relatives to stop them and convince them about benefits and loss of leaving hospital early.
	Care in the ward: I never had any issues/problems during my duty.	
	Care of sick newborns: No problem in managing babies. We are capable of doing everything.	Critically ill babies are referred to nursery.
**3.3**	What challenges do you face while delivery of pregnant women?	
	**Challenges faced**	**How do you manage these challenges**
	Delivery without complication	We face problem in managing women with PIH, PPH and post-delivery hypotoma. Doctors give them treatment or otherwise they refer the patient to higher facility.
	Delivery with complication	If patient bleeds more or if tear occurs, then we have to call the doctor to manage it.
	Caesarean section	We only do cross matching for blood transfusion. Only doctors manage cesarean cases.
	Referred cases with complication	Some patients do not agree to go to safdarjung. Sometimes, the ambulance drivers do not agree to take the patient as they already have bookings.
**3.4**	How long usually the mothers stay at the facility after the delivery?	
	Normal Delivery	Usually from 24-48 hours. If patient has low hb then they stay for long.
	Caesarean Delivery	It depends upon doctor. Usually they discharge on 4th day. Rest it depends upon condition of the patient.
**4. Manpower**		
**4.1**	How many posts of Staff Nurse / ANM are vacant in your health facility?<br>I don't know about this but the staff strength is low at present. Those who are working are also not regular. They take too many leaves. At present we are 12 but there should be 15-16 nurses.	
**4.2**	What difficulties do you face in providing mother and newborn care services to existing employees? (Doctors, nurses and other staff)<br>No problem with anyone. Everyone is cooperative and do their job well.	
**4.3**	What is the mechanism of taking leave and who sanctions it?<br>Yes, newly joined staff gets training in labor room. They work under us for few days. We orient them about whole processes. They are posted in morning shift only for initially few days.	
**5. Duty Roster**		
**5.1**	Who prepares the duty roster for you?<br>Nursing sister prepares roster for us.	
**5.2**	Do you have flexibility in changing the shifts?<br>Yes, we have flexibility. Although I never change my duty but other staff changes by themselves.	
**5.3**	How do you manage when you have double shifts?<br>We rarely did double shifts. It happened just once or twice in past and we get 1 extra off after that. It can be managed easily.	
**5.4**	What is the procedure for taking leave and who approves it?<br>Nursing sister approves our holiday. We have to inform her 1-2 days in advance. In case we need to go for long period then we inform her in 1 week advance.	
**5.5**	Who prepares rosters for emergency / regular service?<br>We do not have any such roster.	
**6. Infrastructure**		

**6.1**	Do you have space to accommodate changes inside the department?<br>Yes, changes are possible in labor room. We sometimes shift tables while doing deliveries.	
**6.2**	Do you have enough beds to accommodate increased number of patients?<br>No, beds are not adequate. Post-operative patients get 1 bed per patient but not for others. Sometimes, 2-3 patients have to adjust on 1 bed.	
**6.3**	Is their regular power supply and clean water for drinking? Any substitute available in case of power cut or irregular water supply?<br>There are no issues related to power cuts. We have generators for power back up as well. But there is no drinking water. We have to get that from our home.	
**7. Data management**		
**7.1**	How do you record data?<br>Our nursing sister is responsible for that. She only manages all the registers.	
**7.2**	How do you maintain a register?<br>Only nursing sister is responsible for it. We only manage deliveries.	
**7.3**	Where do you send the record?<br>I don’t know. Ask sister.	
**7.4**	How often is the data sent?<br>I don’t know.	
**8. Blood bank**		
**8.1**	How long does it take for a needy person to get blood?<br>It usually takes half an hour to get blood. Sometimes it may take little longer. It depends upon availability of drugs.	
**9. Training /Skills**		
**9.1**	Could you tell us about your previous NSSK / Neonatal Resuscitation Training?	
	Time (month / year)	No training for nssk/nrp. I attended ppiucd in 2016.
	place	DI office
	Duration (in days)	6 days.
**9.2**	Who conducts the workshop? Who prepares roster for workshop/training and how it is notified? How it is monitored?<br>We get notification from district training centre. Our incharge receives the information from them and she forwards the details of training to us.	
**9.3**	What did you like the most in the training?<br>They teach well. We get to learn new things. Also, the food is good.	
**9.4**	What did you dislike the most in the training?<br>Earlier we used to get payment for attending the training sessions but now they stopped. Tea was also not good last time.	
**9.5**	what was the training methodology used (Lectures/ Hands-on / Practical’s)?They take lectures through powerpoint and books, practicals, group discussions, videos, pre and post training test.	
**9.6**	How did you like meeting / interacting with the trainers?<br>I felt good. They are good in talking and they answer to all our queries very well.	
**9.7**	What was their level of knowledge / skills?	Some are good and have good knowledge but some do not know much. Dr. had good knowledge.
	How was your conversation with him	It was good. They replied to whatever we asked.
**9.8**	What are the opportunities and mechanisms currently in place/adopted to retain the skills of Nurses/ANMs/Doctors?<br>We should get more of practical experience. Rest I don’t know.	
**9.9**	What challenges do you have with the skills of nurses and the support of staff in delivery rooms, perinatal wards and newborn care units? In your opinion, how can this be controlled?	

**9.10**	How the training related to care during delivery and newborn period can be further improved?<br>No improvement is required. Only some facilities are deficient. They only give us lunch but no snacks with tea. We should get snacks with tea as well and the tea should be good. Drinking plain tea causes acidity.
**9.11**	Have you been to a skill lab set up in your district?<br>Yes, I went there last year.
**9.12**	What are the good things about this skill lab?<br>Everything was fine. We get a good practical experience and theoretical knowledge was also delivered very well. Everything was good from there end but we are the one who have to understand everything.
**9.13**	What are the challenges related to skill lab?<br>I did not had any problem. Whatever I learned I am using it here in daily practice. Already we were doing same things since last 4 years.
**9.14**	In your opinion, how many health staffs might have used or visited the skill labs?<br>I do not have any idea about this.
**9.15**	Did somebody advise or persuade you to attend the skill lab?<br>Our nursing in-charge informed me to attend the training. She got notification from district training centre.
**9.16**	How does the Skill Lab help in Neonatal Resuscitation and Neonatal Care?<br>I already knew everything. Nothing was new for me as we are already doing everything in our daily practice.
**10. Referral services**	
**10.1**	In what situation usually the newborns/mothers (pregnant/recently delivered) are referred to the next level of healthcare?<br>Mothers are referred in case of meconium stains, PIH, placenta Previa, hypertension. We refer babies to NICU if they have breathing problem, delayed cry, if they do not open their eyes.
**10.2**	Where the newborns/ pregnant women/ mothers are usually referred, what is the usual mode of transportation and how long does it takes to reach the next level health facility in your area?<br>We refer babies to NICU and mothers to safdarjung. Ambulance makes delays occasionally. I am not sure how much time it takes to reach there.
**10.3**	What facilitation is done from facility side for referral and what difficulties/challenges do you face while transporting the sick newborn and mother to next level? *(Probe: monetary/logistics)*<br>Sometime patients are not ready to move to safdarjung. Also, the ambulance driver denies taking patients.
**10.4**	What are the challenges faced related to referral transport experienced by this facility and how are they handled?<br>We ask class IV to follow up. Duty doctor also call the ambulance center.
**11. Logistics**	
**11.1**	Are you familiar with any scarcity / irregular supply of medicines and / or supplies required for care during delivery and newborn in the last one year? What were the reasons for this deficiency and how were these conditions managed?<br>No shortage of medicines. We have all the medicines available here.
**11.2**	How frequently the families/ parents asked to procure drugs from outside/ store?<br>We never ask patients to get medicines from outside. All the medicines are available with us.
**11.3**	What are the supervisory mechanisms in place at present for maternal and newborn care services?<br>Yes
	Who supervises<br>Sometimes CMO and sometimes NHM officials visit the hospital for supervision.
	What is the frequency of supervisory visits<br>It is not fixed or scheduled. They can come anytime.

.

	Is any feedback/report provided usually after the supervision?<br>I do not know about it. Nursing sister accompanies them so she must know about it.
	What actions are taken after last supervisory visit?<br>If any patient complaints about the staff then they take action.
**11.4**	Please let us know about the last supervisory visit to the facility related to maternal and newborn care services?
	Who came for last supervisory visit?<br>CMO visited just 1 hour back.
	How long ago the supervisory visit took place?<br>He came here for 15-20 minutes.
	What all components were observed?<br>He visited labor room, ward and corridor. I was inside the labor room.
	What feedback was given and what actions were taken?<br>He did not say anything till now. Otherwise also we are not aware about any actions. We just do our work. Nursing incharge knows better about this.
**12. others**	
**12.1**	How wide do the events in discharge / record slip?<br>Gravida, Para, cesarean, breach or any other diagnosis, details of babies like sex, live or dead and we also put a stamp for medicines.
**12.2**	How much additional effort is needed to reduce the neonatal mortality rate in your area?<br>Many patients shout and ask for discharge. Some patients go back to home without informing but they come back after 1-2 days to take the discharge slip.<br>How can these obstacles be overcome?<br>We only advise them not to do so. We also inform the doctor.
**12.3**	How much additional effort is needed to reduce the neonatal mortality rate in your area?<br>Doctors know better. I just know that mothers are also responsible for increased mortality rate of newborns. They do not come for regular ANC visits. Mothers do not know about condition of the baby.
**12.4**	According to you, what is the meaning of quality?<br>Mother should care about herself and feed the baby properly. Nurse and other staff should also behave politely. Politeness is the quality of a nurse.
**12.5**	What can you do to improve the quality of the health services?<br>There are no fans at some points inside ward. There should be coolers in the ward as well. All patients should get the basic facilities. There should be some facilities for us as well.
**12.6**	What can you do to improve the quality of the health services?<br>I will install 1 cooler and fans wherever required. I will provide all basic facilities to the patients.
**12.7**	Did any of your relatives, friends or acquaintances ever availed health services at this hospital? If not, any reason?<br>No, I never get my relatives or friends here for the treatment because it is very unhygienic and dirty. Also, I am here only for 6 hours hence it is difficult to manage them along with the work.

.

ID: 1108203	
1 Type of Health Facility : DH, FBD	1.2 Designation: STAFF NURSE

2. General

**2.1**	How long have you been working in this health facility? (months/years)<br>I joined NICU in 2015. Prior to that, I was posted in labor room for 3-4 months and then to casualty for 5-6 months.
**2.2**	Total months/years of service<br>My total work experience is 6 years. I started working in 2011.
**2.3**	What are your current roles and responsibility with respect to maternal and neonatal care?<br>My responsibilities include preparing patient's file and maintaining of registers. I make monthly reports and maintain admission and death registers. I give antibiotics to patients and help them in feeding. I do eye and mouth care of the babies and general care required for newborn. I also check vitals of out born patients. I maintain fumigation register, admission register, death register, inborn patient register and referral register.
**2.4**	How many deliveries and resuscitations of newborns have you attended in last 1 month?

A. No. of deliveries attended in last 1 month	NA
B. No. of newborn resuscitations attended in last 1 month	10-15

2.5 Who did you receive the training from:

	Area	Training name	Year
A	Care during delivery (S.B.A.)	SBA	2014
B	Neonatal Resuscitation Program (In RP / NSS)	NRP and NSSK	2015
C	Sick Newborn Care (FBNC)	FBNC	2016

3. Service Delivery

3.1 What are the challenges faced by you and your colleagues for delivering the desired mother and newborn care services?

	Challenges faced	
	**Challenges faced**	**Challenges faced**
Infrastructure	NA	It is difficult to fumigate step down room as the entry gate is small.
Equipment	NA	There are no issues with equipments. Only problem is that it takes about a month to repair any equipment. Nursing sister is incharge for that.
Drugs and supplies	NA	We do not get medicines and supplies on time. Inj phenobard and tempers are not available at present. We ask patient to get it from outside.
Support services	NA	No issues
Other	NA	No issues

3.2 What challenges do you face while delivering essential newborn care services and how do you manage these?

Challenges faced	How do you manage these challenges
Care at delivery	
Care in the ward	
Care of sick newborns: Some parents leave their girl child here.	We call police and register complaint to call back parents.

3.3 What challenges do you face while delivery of pregnant women?

Challenges faced	How do you manage these challenges
Delivery without complication	NA

	Delivery with complication	NA
	Caesarean section	NA
	Referred cases with complication	NA
**3.4**	How long usually the mothers stay at the facility after the delivery?	
	Normal Delivery	NA
	Caesarean Delivery	NA
**4. Manpower**		
**4.1**	How many posts of Staff Nurse / ANM are vacant in your health facility?<br>Required post for staff nurse in NICU is 10, out of which 1 post is vacant whereas 2 are shifted to casualty department and 1 is on maternity leave. Hence, as of now we are only 6 staff nurses here.	
**4.2**	What difficulties do you face in providing mother and newborn care services to existing employees? (Doctors, nurses and other staff)<br>No issues with anyone. Everyone is cooperative and listen to each other, hence it makes our work easy.	
**4.3**	Are there training postings for newly appointed staff?<br>There is no separate training for new staff. They work with us for initial days and learn about all the processes.	
**5. Duty Roster**		
**5.1**	Who prepares the duty roster for you?<br>One of the staff nurse out of 6 is made incharge of making duty register on monthly basis. The charge is rotated every month and everyone gets equal opportunity to make the duty roster.	
**5.2**	Do you have flexibility in changing the shifts?<br>Yes, changes are possible. We just have to inform the doctor on duty and it is done.	
**5.3**	How do you manage when you have double shifts?<br>We rarely have to do double shifts. It is not very difficult as other staff is also there to help.	
**5.4**	What is the procedure for taking leave and who approves it?<br>For CL, Dr. approves our leave. We get approval only if any other staff is available.	
**5.5**	Who prepares rosters for emergency / regular service?<br>There is no separate register for emergency duty. We just make 1 register.	
**6. Infrastructure**		
**6.1**	Do you have space to accommodate changes inside the department?<br>Yes, changes are possible within department. We have lot of space now.	
**6.2**	Do you have enough beds to accommodate increased number of patients?<br>No, we do not have sufficient space to keep babies. Sometime we have to do doubling or even tripling of babies on 1 radiant warmer.	
**6.3**	Is their regular power supply and clean water for drinking? Any substitute available in case of power cut or irregular water supply??<br>There is no problem of power cut. Power cut occurs hardly for 1-2 minute. We do not have access to clean drinking water. I get drinking water from home.	
**7. Data management**		
**7.1**	How do you record data?<br>We update all data on registers. We have different registers i.e. daily report book, admission register, refer register, death register, daily round of doctor register.	
**7.2**	How do you maintain a register?<br>We update all the registers on daily basis. Report book is prepared twice in a day.	
**7.3**	Where do you send the record?<br>We give all the data to DEO at NICU. He maintains all the data on computer.	
**7.4**	How often is the data sent?<br>All administrative data is forwarded on monthly basis. Other data is also sent on regular basis. DEO is responsible for it so he send the data every month.	

.

**8. Blood bank**	
**8.1**	How long does it take for a needy person to get blood?<br>It takes about 1-2 hours to get blood for the patient. The process of taking blood from the blood bank is too long.
**9. Training /Skills**	
**9.1**	Could you tell us about your previous NSSK / Neonatal Resuscitation Training?

	Time (month / year)	I do not remember about the time
	place	BK Hospital
	Duration (in days)	2 days

**9.2**	Who conducts the workshop? Who prepares roster for workshop/training and how it is notified? How it is monitored?<br>Officials at District training centre prepare our roster for the training. They have details of everyone, hence they themselves prepare a list of trainees and notify us.
**9.3**	What did you like the most in the training?<br>Training sessions are good to update our learnings. We get to know new things. We meet new people so it is good.
**9.4**	What did you dislike the most in the training?<br>Training sessions are very long and boring. It is good initially but lectures after lunch are very difficult to attend.
**9.5**	What was the training methodology used (Lectures/ Hands-on / Practical's)?<br>They give us demo and teach from books. Some lectures involve PowerPoint slides as well.
**9.6**	How did you like meeting / interacting with the trainers?<br>Everyone know us so they talk very nicely.

**9.7**	What was their level of knowledge / skills?	Dr. had a very good knowledge. Present trainers are just fine.
	How was your conversation with him	It was fine but now they usually teach us only from books.

**9.8**	What are the opportunities and mechanisms currently in place/adopted to retain the skills of Nurses/ANMs/Doctors?<br>Trainings are the only way to update knowledge and skills. But no training has been conducted since long time. Last training I attended was on skill lab which held almost 3 months back.
**9.9**	What challenges do you have with the skills of nurses and the support of staff in delivery rooms, perinatal wards and newborn care units? In your opinion, how can this be controlled?<br>Senior staff is helpful and they teach us here only. Practical challenges can be well understood on patients only.
**9.10**	How the training related to care during delivery and newborn period can be further improved?<br>No other problem except that if number of participants is more than quality of food is not good.
**9.11**	Have you been to a skill lab set up in your district?<br>Yes, I went there 3 months back.
**9.12**	What are the good things about this skill lab?<br>They give demo very nicely. They have different cabins for every section and they are managed very well.
**9.13**	What are the challenges related to skill lab?<br>I do not have to face any challenges related to skill lab. Everything was good.
**9.14**	In your opinion, how many health staffs might have used or visited the skill labs?<br>All other staff nurse except have attended skill lab. She went today for the training.
**9.15**	Did somebody advise or persuade you to attend the skill lab?<br>We get notification from district training centre only.
**9.16**	How does the Skill Lab help in Neonatal Resuscitation and Neonatal Care?<br>There was nothing new in relation to newborn care or resuscitation. We already resuscitate newborns in routine work while working in NICU. It would have been beneficial for staff from

.

	dispensary or other health units.
**10. Refferal Service**	
**10.1**	In what situation usually the newborns/mothers (pregnant/recently delivered) are referred to the next level of healthcare?<br>We refer patients with congenital anomaly, babies with bleeding, severe sepsis, continuous seizures and the patients who need ventilator support. Many parents refuse to take their babies as they do not like to visit safdarjung hospital.
**10.2**	Where the newborns/ pregnant women/ mothers are usually referred, what is the usual mode of transportation and how long does it takes to reach the next level health facility in your area?<br>We refer the patients to safdarjung, rest is on choice of the family. They can go to private as well. We provide ambulance services to transfer the patient. It takes around 30-45 minutes to reach safdarjung. Ambulance services are free of cost for the patients.
**10.3**	What facilitation is done from facility side for referral and what difficulties/challenges do you face while transporting the sick newborn and mother to next level? *(Probe: monetary/logistics)*<br>If a baby is send with ambu bag than EMT accompanies the child till next facility or else they go alone.
**10.4**	What are the challenges faced related to referral transport experienced by this facility and how are they handled?<br>Ambulance is not available everytime especially the one with oxygen support. Many babies die due to this reason. We do whatever is possible from our side. Some patients arrange for private ambulance as well.
**11. Logistics**	
**11.1**	Are you familiar with any scarcity / irregular supply of medicines and / or supplies required for care during delivery and newborn in the last one year? What were the reasons for this deficiency and how were these conditions managed?<br>Many medicines are not available in stock with us. Patients have to buy it from outside. Inj dopamine, merupinum, pipramycin and vancomycin are presently not available with us. We do not know about the reason of shortage.
**11.2**	How frequently the families/ parents asked to procure drugs from outside/ store?<br>Most of the patients have to buy medicines from outside.
**11.3**	What are the supervisory mechanisms in place at present for maternal and newborn care services?<br>Yes, regular supervision visits takes place.
	Who supervises<br>PMO, CMO and sometime NHM team from Panchkula visit the facility for supervision.
	What is the frequency of supervisory visits<br>NHM team visited just once or twice but CMO and PMO visit regularly.
	Is any feedback/report provided usually after the supervision?<br>No reports are made after visits. There are no complaints from patients or from other things so it was never required.
	What actions are taken after last supervisory visit?<br>We as patients to get hand washing soaps as liquid soap which we get from hospital is not of good quality. The supervisory team asked us not to ask patients to get soap.
**11.4**	Please let us know about the last supervisory visit to the facility related to maternal and newborn care services?
	Who came for last supervisory visit?<br>Officials from NHM visited last week.
	How long ago the supervisory visit took place?<br>They came here for very less time.
	What all components were observed?<br>First they met patients on mother side then they visited our department as well.

	What feedback was given and what actions were taken?<br>We asked patients to get soap and Paracetomol (Susp) for which they advised us not to repeat this again. They took photograph of the slip on which we wrote to bring these items.
**12. others**	
**12.1**	How wide do the events in discharge / record slip?<br>The discharge slip is exactly same as admission slip. We write details of admission, antibiotic given to the baby and details of all investigations done during the period of stay.
**12.2**	In your view, what are the perceived barriers between families in the use of public health services for newborns of pregnant women?<br>Some patients are good some argue and fight with us if we do not allow them to enter inside NICU or if we refuse to show their baby everytime.<br>How can these obstacles be overcome?<br>We call the security guard from outside and sometime we have to call police as well if the patient misbehave and continuously argue with us.
**12.3**	How much additional effort is needed to reduce the neonatal mortality rate in your area?<br>Increase our staff strength. Post atleast 3 staff nurse in morning shift and 2 each in evening and night shift. We face problem in resuscitate a baby and sometimes we have to take help from class IV.
**12.4**	According to you, what is the meaning of quality?<br>Infection should be prevented. Rest, I do not have much idea about it.
**12.5**	What can you do to improve the quality of the health services?<br>Sometimes, number of patients are more. In such situation, we face shortage of beds. Then we have to do doubling and tripling of patients. Scarcity of staff should be considered. Atleast our sanctioned post of 10 staff nurse should be fulfilled.
**12.6**	What can you do to improve the quality of the health services?<br>Increase the number of staff, quality will improve automatically. We should fix the number of patients and should not allow any further admission if the admission exceeds the desired number.
**12.7**	Did any of your relatives, friends or acquaintances ever availed health services at this hospital? If not, any reason?<br>Yes, they had visited for treatment but only for NICU. I never advise for any other department because they are always overcrowded.

ID: 1108204	
**1.1 Type of Health Facility DH, Faridabad**	**1.2 Designation: staff nurse**
**2. General**	

**2.1**	How long have you been working in this health facility? (months/years)<br>I am working in this hospital since 2009. Since then I am posted here only.
**2.2**	Total months/years of service<br>I am working since more than 30 years now. I joined my job in 1983. I am posted in Faridabad since then.
**2.3**	What are your current roles and responsibility with respect to maternal and neonatal care?<br>I am nursing sister here so I supervise all staff nurse, check daily entries in register, check bedding and assure that bedsheets are being changed regularly. I also listen to problems of the patients. I make indent for medicines and prepare daily and monthly reports as well. I maintain all records and registers of patient’s data as well as of staff.
**2.4**	How many deliveries and resuscitations of newborns have you attended in last 1 month?

A. No. of deliveries attended in last 1 month	20-25
B. No. of newborn resuscitations attended in last 1 month	NA

2.5 Who did you receive the training from:

	Area	Training name	Year
A	Care during delivery (S.B.A.)	SBA	2013
B	Neonatal Resuscitation Program (In RP / NSS)	NSSK; NRP	2015
C	Sick Newborn Care (FBNC)	Not done	NA

3. Service Delivery

3.1 What are the challenges faced by you and your colleagues for delivering the desired mother and newborn care services?

Challenges faced		
	**Mother care**	**Newborn care**
Infrastructure	No issues	No issues
Equipment	No issues	No issues
Drugs and supplies	Sometimes medicines are not available. We usually replace the medicine if there is shortage or do local purchase.	No issues
Support services	No issues	No issues
Other	Issues in breastfeeding support. Some patients disturb us deliberately	No issues

3.2 What challenges do you face while delivering essential newborn care services and how do you manage these?

**Challenges faced**	**How do you manage these challenges**
Care at delivery: no issues or challenges	
Care in the ward: patients do not maintain cleanliness in ward. They through all the garbage in ward and gallery/corridor.	We advise them not to do so and sometime scold them as well. Ward should have mesh windows to maintain cleanliness.
Care of sick newborns	We refer all the sick newborns to SNCU.

3.3 What challenges do you face while delivery of pregnant women?

	**Challenges faced**	**Challenges faced**
	Delivery without complication	No issues
	Delivery with complication	We ask doctor to consult. We refer patients with pre-eclampsia to safdarjung.
	Caesarean section	Doctors do all cesarean cases. Staff at OT usually assist them.
	Referred cases with complication	Patients do not agree to visit safdarjung. If they still not listen then we make them sign consent about high risk.
**3.4**	How long usually the mothers stay at the facility after the delivery?	
	Normal Delivery	Usually for 24 hours because of shortage of beds. Maximum upto 48 hours.
	Caesarean Delivery	Upto 4 days for C/s cases
**4. Manpower**		
**4.1**	How many posts of Staff Nurse / ANM are vacant in your health facility?<br>We do not have any ANM in department. Total 13 staff nurse are in position for the department but 2 are posted in other department and 1 is on maternity leave. Total 18 staff nurse are sanctioned for labor room but only 10 are present at present.	
**4.2**	What difficulties do you face in providing mother and newborn care services to existing employees? (Doctors, nurses and other staff)<br>No problem with anyone. Everyone is cooperative and work with good understanding.	
**4.3**	What is the mechanism of taking leave and who sanctions it?<br>The new staff get training at DI office. We also give them initial training about the system. New staff is posted in morning shift for initial 15-20 days under supervision of senior staff.	
**5. Duty Roster**		
**5.1**	Who prepares the duty roster for you?<br>I prepare the roster for all staff nurse as I am the nursing sister.	
**5.2**	Do you have flexibility in changing the shifts?<br>Yes, most of the staff changes their shift by themselves. No one listen to me. Sometimes they even do not inform me before changing the roster.	
**5.3**	How do you manage when you have double shifts?<br>I did double shift a lot of times. It is not very difficult. I can manage easily.	
**5.4**	What is the procedure for taking leave and who approves it?<br>Our matron sister sanctions holiday for us. We do not have to take consent of doctor before taking any leave.	
**5.5**	Who prepares rosters for emergency / regular service?<br>No separate roster is prepared for emergency duties. We prepare a separate roster for public holidays. Everyone have to follow it. We make sure that no staff is getting double or repeat shift on that roster.	
**6. Infrastructure**		
**6.1**	Do you have space to accommodate changes inside the department?<br>We need lot of changes to be done within department but we do not have any extra space. We already have extra number of beds in the ward.	
**6.2**	Do you have enough beds to accommodate increased number of patients?<br>In case of more number of patients, we do doubling of patients and sometimes if required we put 3 patients on 1 bed.	
**6.3**	Is their regular power supply and clean water for drinking? Any substitute available in case of power cut or irregular water supply?<br>Power supply is regular but we have to get our own water from home. RO is installed on floor but getting clean water from it is not assured.	
**7. Data management**		

**7.1**	How do you record data?<br>We do entries in register. There are separate registers for everything.	
**7.2**	How do you maintain a register?<br>We regularly update all the registers and send a monthly report for everything. Live births are reported on daily basis to PMO office.	
**7.3**	Where do you send the record?<br>Records are sent to PMO office. They send it to DI office or Panchkula. I am not sure about it.	
**7.4**	How often is the data sent?<br>Some send some data on daily basis like total live births otherwise complete data is sent every month to higher authorities.	
**8. Blood bank**		
**8.1**	How long does it take for a needy person to get blood?<br>If blood is available in blood bank then it takes about half an hour to get it for the patient. If patient have to get it from outside, then it takes time. Almost 10 out of 80 patients have to get blood from private blood banks.	
**9. Training /Skills**		
**9.1**	Could you tell us about your previous NSSK / Neonatal Resuscitation	
	Time (month / year)	2016
	place	Munirka
	Duration (in days)	1 week
**9.2**	Who conducts the workshop? Who prepares roster for workshop/training and how it is notified? How it is monitored?<br>DI office generate list of trainees.	
**9.3**	What did you like the most in the training?<br>Best thing is that I get leave from labor room. I learned new things and met new people.	
**9.4**	What did you dislike the most in the training?<br>We have to leave for children at home. We have to travel too far but we do not get any TA/DA now.	
**9.5**	What was the training methodology used (Lectures/ Hands-on / Practical's)?<br>Training was done through lectures and demos. Powerpoint slides and videos were also used. We did practical over dummies. Group discussion was the best part of the training. Pre and post training test were done and the training finally ended up by filling a feedback form.	
**9.6**	How did you like meeting / interacting with the trainers?<br>Interaction with trainer is good. They always tell us something new.	
**9.7**	What was their level of knowledge / skills?	It was good. They had good knowledge about the subject.
	How was your conversation with him	Discussion was good. The trainers were very friendly.
**9.8**	What are the opportunities and mechanisms currently in place/adopted to retain the skills of Nurses/ANMs/Doctors?<br>Training should be conducted in regular intervals and everyone should follow what they learn from the sessions.	
**9.9**	What challenges do you have with the skills of nurses and the support of staff in delivery rooms, perinatal wards and newborn care units? In your opinion, how can this are controlled?<br>No issues at all. We discuss any issues and find the result.	
**9.10**	How the training related to care during delivery and newborn period can be further improved? Everything is good. Only problem is that we have to travel too far and we do not get TA/DA for it.	
**9.11**	Have you been to a skill lab set up in your district?<br>Yes, I have been there. It is located on back side of the hospital at DI office.	
**9.12**	What are the good things about this skill lab?<br>We learnt from munirka and then we set everything here. I am trainer for skill lab as well. Overall set up of skill lab is good.	
**9.13**	What are the challenges related to skill lab?	

.

	Practical experience is different from what we learnt at skill lab. We cannot apply the techniques practically for which we were trained.
**9.14**	In your opinion, how many health staffs might have used or visited the skill labs?<br>All staff nurse had attended the skill lab. No one is left.
**9.15**	Did somebody advise or persuade you to attend the skill lab?<br>DI office prepares the list and notify to PMO office. We get a call to attend the training session from PMO office.
**9.16**	How does the Skill Lab help in Neonatal Resuscitation and Neonatal Care?<br>We learned a lot of new things but we do not apply them practically.
**10. Referral services**	
**10.1**	In what situation usually the newborns/mothers (pregnant/recently delivered) are referred to the next level of healthcare?<br>We refer mothers in case of PPH, eclampsia and severe anemia. All sick newborns are referred to SNCU. They decide if they want to refer further or they will treat the baby at SNCU only.
**10.2**	Where the newborns/ pregnant women/ mothers are usually referred, what is the usual mode of transportation and how long does it takes to reach the next level health facility in your area?<br>We refer the patient to safdarjung hospital by ambulance. It takes about 40 minutes to reach there.
**10.3**	What facilitation is done from facility side for referral and what difficulties/challenges do you face while transporting the sick newborn and mother to next level? *(Probe: monetary/logistics)*<br>EMT accompanies the patient till next facility. There is no problem in referring except that sometime patient do not agrees to go to Delhi or occasionally ambulance is not available.
**10.4**	What are the challenges faced related to referral transport experienced by this facility and how are they handled?<br>Sometimes patient do not agree to go to other hospital. Sometimes ambulance is not available. In that case we ask patient to either wait or the ambulance driver adjust the patient with other in 1 ambulance.
**11. Logistics**	
**11.1**	Are you familiar with any scarcity / irregular supply of medicines and / or supplies required for care during delivery and newborn in the last one year? What were the reasons for this deficiency and how were these conditions managed?<br>We do local purchase of drugs which are not available in stock. At present, seru gel is not available with us.
**11.2**	How frequently the families/ parents asked to procure drugs from outside/ store?<br>It happened very rarely that we asked patients to get anything from outside. Most of the patients are aware now. They can also file a complaint against us, hence we avoid this situation.
**11.3**	What are the supervisory mechanisms in place at present for maternal and newborn care services<br>Yes, supervision visits are done by higher authorities.
	Who supervises<br>PMO and sometimes NHM team.
	What is the frequency of supervisory visits<br>Earlier PMO used to visit on weekly basis. Now he can come whenever he want to. Nothing is fixed.
	Is any feedback/report provided usually after the supervision?<br>Yes, they make a report about the visit. They have also given us a register to write complaints.
	What actions are taken after last supervisory visit?<br>Yes, PMO scolded us in meeting.
**11.4**	Please let us know about the last supervisory visit to the facility related to maternal and newborn care services?
	Who came for last supervisory visit?<br>Dr. from NHM visited last week.
	How long ago the supervisory visit took place?

.

	He came in evening for about 1 hour.
	What all components were observed?<br>He visited labor room and maternity ward at our side. Then he went to other side of the hospital.
	What feedback was given and what actions were taken?<br>He scolded everyone because of cleanliness issues. We took action after that and a sweeper cleaned everything in morning but again the patients have made a mess in the ward. The situation is back to the same point.
**12. others**	
**12.1**	How wide do the events in discharge / record slip?<br>We write ANC history details of medicines, baby's detail and details about follow up visit on discharge slip. We have a preformed stamp which we use for all patients.
**12.2**	In your view, what are the perceived barriers between families in the use of public health services for newborns of pregnant women?<br>All patients want VIP treatment and they expect us to sit with them every time. Most of the patients want that their treatment should be done in priority so that they can go back home soon. No one wants to wait.<br>How can these obstacles be overcome?<br>We should advise the patient about the benefits of care in hospital. Sometime we have to scold them and fight as well.
**12.3**	How much additional effort is needed to reduce the neonatal mortality rate in your area?<br>Public should be aware about preventions and precautions. They should come for regular ANC visits.
**12.4**	According to you, what is the meaning of quality?<br>Quality means to improve our work. If quantity will decrease then only we can expect good quality for patients or else staff position should increase.
**12.5**	According to you, what are the issues affecting the quality (quality) of health services?<br>The main issues which affect the quality are shortage of staff, illiteracy among patients, lack of awareness, lack of facilities in hospitals.
**12.6**	What can you do to improve the quality of the health services?<br>Quality can be improved by giving timely training to the staff. If the staff is skilled and capable then lot of improvement can be expected.
**12.7**	Did any of your relatives, friends or acquaintances ever availed health services at this hospital? If not, any reason?<br>Yes, my friends and relative visit this hospital for treatment. They get good treatment here.

.

ID :1108205			
**1.1 Type of Health Facility: Dh, fbd**			**1.2 Designation: STAFF NURSE**
**2. General**			
**2.1**	How long have you been working in this health facility? (months/years)		
**2.2**	Total months/years of service		
**2.3**	What are your current roles and responsibility with respect to maternal and neonatal care?		
**2.4**	How many deliveries and resuscitations of newborns have you attended in last 1 month?		
	A. No. of deliveries attended in last 1 month		
	B. No. of newborn resuscitations attended in last 1 month		
**2.5**	Who did you receive the training from:		
	Area	Training name	Year
	A Care during delivery (S.B.A.)		
	B Neonatal Resuscitation Program (In RP / NSS)		
	C Sick Newborn Care (FBNC)		
**3. Service Delivery**			
**3.1**	What are the challenges faced by you and your colleagues for delivering the desired mother and newborn care services?		
	**Challenges faced**		
		**Mother care**	**Newborn care**
	Infrastructure		
	Equipment		
	Drugs and supplies		
	Support services		
	Other		
**3.2**	What challenges do you face while delivering essential newborn care services and how do you manage these?		
	**Challenges faced**	**How do you manage these challenges**	
	Care at delivery		
	Care in the ward		
	Care of sick newborns		
**3.3**	What challenges do you face while delivery of pregnant women?		
	**Challenges faced**	**How do you manage these challenges**	
	Delivery without complication		
	Delivery with complication		
	Caesarean section		
	Referred cases with complication		
**3.4**	How long usually the mothers stay at the facility after the delivery?		
	Normal Delivery		
	Caesarean Delivery		
**4. Manpower**			
**4.1**	How many posts of Staff Nurse / ANM are vacant in your health facility?		
**4.2**	What difficulties do you face in providing mother and newborn care services to existing employees? (Doctors, nurses and other staff)		
**4.3**	What is the mechanism of taking leave and who sanctions it?		

**5. Duty Roster**		
**5.1**	Who prepares the duty roster for you?	
**5.2**	Do you have flexibility in changing the shifts?	
**5.3**	How do you manage when you have double shifts?	
**5.4**	What is the procedure for taking leave and who approves it?	
**5.5**	Who prepares rosters for emergency / regular service?	
**6. Infrastructure**		
**6.1**	Do you have space to accommodate changes inside the department?	
**6.2**	Do you have enough beds to accommodate increased number of patients?	
**6.3**	Is their regular power supply and clean water for drinking? Any substitute available in case of power cut or irregular water supply?	
**7. Data management**		
**7.1**	How do you record data?	
**7.2**	How do you maintain a register?	
**7.3**	Where do you send the record?	
**7.4**	How often is the data sent?	
**8. Blood bank**		
**8.1**	How long does it take for a needy person to get blood?	
**9. Training /Skills**		
**9.1**	Could you tell us about your previous NSSK / Neonatal Resuscitation Training?	
	Time (month / year)	I don't remember
	place	faridabad
	Duration (in days)	
**9.2**	Who conducts the workshop? Who prepares roster for workshop/training and how it is notified? How it is monitored?<br>Incharge get the details through e mail from DI office.	
**9.3**	What did you like the most in the training?<br>Teaching staff was good. We learned something new. The session refreshed our knowledge.	
**9.4**	What did you dislike the most in the training?<br>Everything was good.	
**9.5**	What was the training methodology used (Lectures/ Hands-on / Practical's)?<br>Lectures, dummy, projector, notes, group discussions, pre & post test, role play.	
**9.6**	How did you like meeting / interacting with the trainers?<br>It was good. We learnt something new	
**9.7**	What was their level of knowledge / skills?	It was very high. Dr. is the best
	How was your conversation with him	It was very good and informative
**9.8**	What are the opportunities and mechanisms currently in place/adopted to retain the skills of Nurses/ANMs/Doctors?<br>Apart from training, we learn from experience. Every day we learn something new while working on patients.	
**9.9**	What challenges do you have with the skills of nurses and the support of staff in delivery rooms, perinatal wards and newborn care units? In your opinion, how can this be controlled?	
**9.10**	How the training related to care during delivery and newborn period can be further improved?<br>No change is required in the sessions. We should get reimbursement for attending trainings. They do not pay even basic TA/DA to us.	
**9.11**	Have you been to a skill lab set up in your district?<br>It is situated at DI Office	
**9.12**	What are the good things about this skill lab?<br>Training was related to our profession and it refreshed our knowledge. We learnt many new things as well.	

**9.13**	What are the challenges related to skill lab?<br>Nothing. Whatever we learnt from there, we can apply it easily at the labor room.
**9.14**	In your opinion, how many health staffs might have used or visited the skill labs?<br>2-3 staff members have attended the skill lab training till now. I am not sure about others.
**9.15**	Did somebody advise or persuade you to attend the skill lab?<br>Nursing sister informed me about training session. She got the notification from DI office.
**9.16**	How does the Skill Lab help in Neonatal Resuscitation and Neonatal Care?<br>We learnt the resuscitation process over a dummy. We practiced it again and again. Now I know how to use the ambu bag.
**10. Referral services**	
**10.1**	In what situation usually the newborns/mothers (pregnant/recently delivered) are referred to the next level of healthcare?<br>We refer the baby to NICU if the baby do not cry at birth, low birth weight, pre mature, congenital problem.<br>Mothers are referred for PIH, PPH, severe anemia or if blood is not available.
**10.2**	Where the newborns/ pregnant women/ mothers are usually referred, what is the usual mode of transportation and how long does it takes to reach the next level health facility in your area?<br>We refer the patients to safdurjung hospital. I am not sure about the time as I never accompany the patient.
**10.3**	What facilitation is done from facility side for referral and what difficulties/challenges do you face while transporting the sick newborn and mother to next level? *(Probe: monetary/logistics)*EMT accompanies the newborn in the ambulance. I am not sure about other things.
**10.4**	What are the challenges faced related to referral transport experienced by this facility and how are they handled?<br>We inform the patient or attendant about referral but many times they refuse to go. We make them understand that we do not have ICU and ventilator facilities. We can keep critical patients only if we have staff and adequate equipments.
**11. Logistics**	
**11.1**	Are you familiar with any scarcity / irregular supply of medicines and / or supplies required for care during delivery and newborn in the last one year? What were the reasons for this deficiency and how were these conditions managed?<br>Nowadays we are getting everything. Medicines and supply are fulfilled by any method. I am not sure about that.
**11.2**	How frequently the families/ parents asked to procure drugs from outside/ store?<br>Never. All the medicines are available from the department only.
**11.3**	What are the supervisory mechanisms in place at present for maternal and newborn care services?<br>Yes
	Who supervises<br>CMO, PMO and NHM team
	What is the frequency of supervisory visits<br>It depends upon their wish. Sometimes daily or else monthly
	Is any feedback/report provided usually after the supervision?<br>Nursing sister must be aware. We do not get any details.
	What actions are taken after last supervisory visit?<br>If they find any problem then they take some action. It never happened till now.
**11.4**	Please let us know about the last supervisory visit to the facility related to maternal and newborn care services?
	Who came for last supervisory visit?<br>So many teams visit so we don’t know. CMO visited the department 4-5 days back.
	How long ago the supervisory visit took place?

	He took a round for about 30 minutes.
	What all components were observed?<br>Maternity ward, labor room in our department and then he went to other parts of the hospital.
	What feedback was given and what actions were taken?<br>I had an day off so I don't know.
**12. others**	
**12.1**	How wide do the events in discharge / record slip?<br>Discharge slip includes name, age, sex and other general details, previous medical history, procedures and treatment performed, advice, medications and follow up visits.
**12.2**	In your view, what are the perceived barriers between families in the use of public health services for newborns of pregnant women?<br>They do not have patience. They are always in hurry and do not listen to the instructions given by us.<br>How can these obstacles be overcome?<br>We ask the patients to come 1 by 1. We tell them that they will be confused but still they don't agree.
**12.3**	How much additional effort is needed to reduce the neonatal mortality rate in your area?<br>Institutional deliveries should be promoted. Immunization should be done at birth only.
**12.4**	According to you, what is the meaning of quality?<br>Providing good health services to the patients, health of patient should improve. Reduction in rate of mortality. Instruments should be sterilized. There should be no cross infection. Timely treatment should be available for all the patients. All patients should receive counseling.
**12.5**	According to you, what are the issues affecting the quality (quality) of health services?<br>There are many things which affect the quality. We are not regular, so permanent staff have a higher hand on us. Our salaries are low and hence it will affect the quality.
**12.6**	What can you do to improve the quality of the health services?<br>We should get permanent job and good salaries then only we can improve the quality. Salary should be according to the qualification.
**12.7**	Did any of your relatives, friends or acquaintances ever availed health services at this hospital? If not, any reason?<br>There are no private rooms here. So we do not call our relatives here. They usually come to take advice at OPD.

.

ID: 1108301	
**AREA DH, fbd**	**DESIGNATION : class IV**
**S.No.**	**Questions**
**1.**	**General Information**
1.1	Are you an employee of the hospital or are you contracted for the same?<br>I have been working in this hospital since 2011. I am appointed through NHM.
1.2	Please tell us about the duty of duty and how many people are present in the cross<br>There are 4 class IV working here. 1 staff at each shift.
**2.**	**Specific Information**
2.1	Please tell us about the duty of duty and how many people are present in the cross<br>There are total 8 shifts at night and 8 off in one month. Rest I work in morning shift and evening shift. There is only one staff available at SNCU at a time.
2.2	Your responsibility is only of one department, of all the other departments of the hospital?<br>My work is assigned for SNCU and mother's waiting area.
2.3	explain your responsibilities<br>My work is mopping, cleaning, dusting. I change bed sheets. I also assist staff nurses to their work like to switch on the radiant warmer, to call patient's attendant. I also clean instruments and Organize sterilized sheets inside in drum.
2.4	Do you have a duty even during the night shift?<br>Yes.
2.5	Tell us which places in this hospital do you take care of cleanliness?<br>My duty is only assigned to SNCU and mother's waiting area.
2.6	Tell us about your family's success?<br>As soon as I arrive, I clean it and before going I clean whole area of SNCU.
2.7	What media do you use for cleaning many types of goods?<br>I use gloves for making bleaching solution. For doors and tiles we use detergent powder and for dusting. I use dusting .I do cleaning the floor with phenyl.
2.8	Does anyone check your work?<br>Yes, Dr. monitors my work.
**3.**	**Disposal**
3.1	How you are you dispose the waste?<br>A boy daily comes to collect wastes.
3.2	Do you use color coding systems for waste disposal?<br>Detailed Yes, I segregate waste and discard it to color coded plastic bags. I use black plastic bags for general waste, red plastic bags for plastic items, and use blue bags for glass made materials and yellow bags for blood, and wet waste
3.3	There is any vehicle for pickup the waste?<br>I ant tell you. I dispose waste at ground floor. I don't know how they transfer waste to van
3.4	If yes, then how many times is it come in the hospital?<br>I don't have fair idea about it.
3.5	Do you burn the waste room? If so where?<br>I don't burn waste. I only puncture syringes.
**4.**	**Issues/Suggestions**
4.1	tell us how other staff members treat you in hospital<br>Every staff of SNCU behaves well, we work together.
4.2	tell us how patients treat you in the hospital

	Sometimes patients misbehave with us.
4.3	tell us what difficulty you face during your work
4.4	Want to change the way you do for better service?<br>Patient does not listen to us. Supply mechanism should be regular, so that it has not to buy any medicine from outside
**4.5**	What do you mean by quality?<br>I don't have any about it.
**4.6**	According to you, what are the issues affecting the quality of health services?
**4.7**	What can you do to improve the quality of health services?<br>Patients are very rude here, they misbehave.
**4.8**	Have your relatives, friends or acquaintances ever taken advantage of the health services of this hospital? If not, any reason?<br>Yes, my nephew & niece was admitted, almost all relatives want to come.

ID: 1108302	
**AREA DH, fbd**	**DESIGNATION : class IV**
**1.**	**General Information**
1.1	Are you an employee of the hospital or are you contracted for the same?<br>I am on contractual basis. I am recruited by private contractor.
1.2	How many class 4 are there in this hospital?<br>I don't have fair idea about it.
**2.**	**Specific Information**
2.1	Please tell us about the duty of duty and how many people are present in the cross<br>Our duty runs shift wise, There are three shifts scheduled for a day, morning evening and night. Two staffs depute for morning shift, two Staffs for Night shift and one staff for evening.
2.2	Your responsibility is only of one department, of all the other departments of the hospital?<br>Mostly my duty is at labour room and maternity ward, but sometimes it changes, and I am assigned in medical ward, OPD and surgical ward as well.
2.3	explain your responsibilities<br>I Do dusting of labour room and waiting area, scrubbing and making bleaching solution. Taking patient from LR to OT , I wipe out newborn and perform the sterilization of equipments/ instruments.
2.4	Do you have a duty even during the night shift?<br>Yes, sometimes.
2.5	Tell us which places in this hospital do you take care of cleanliness?<br>I mostly work inside labour room, maternal ward. Also I do clean tiles, windows and side trolley.
2.6	Tell us about your family's success?<br>Since, this duty is assigned for class IV, we do it duty wise. A part from this whenever I see any filth, I clean it immediately.
2.7	What media do you use for cleaning many types of goods?<br>I use gloves for making bleaching solution. For doors and tiles we use detergent powder and for dusting we use dusting mops.
2.8	Does anyone check your work?<br>Yes, my work is checked by nursing sister and class IV supervisor.
**3.**	**Disposal**
3.1	How you are you dispose the waste?<br>I collect the filths form LR and wards and put it transfer to Waste disposal van.
3.2	Do you use color coding systems for waste disposal? Detailed<br>Yes, I segregate waste and discard it to color coded plastic bags. I use black plastic bags for general waste, red plastic bags for plastic items, and use blue bags for glass made materials and yellow bags for blood, and wet waste.
3.3	Is there any vehicle for pickup the waste?<br>Yes, Daily waste disposal van comes to take waste.
3.4	If yes, then how many times is it come in the hospital?<br>Sweeper will tell you.
3.5	Do you burn the waste room? If so where<br>No.
**4.**	**Issues/Suggestions**
4.1	tell us how other staff members treat you in hospital<br>Everything is fine here, good , helpful. and polite

4.2	tell us how patients treat you in the hospital<br>Few, patients misbehave with me but overall it is good.

ID: 1208101	
**1.1 Type of Health Facility: FRU 2, REWARI**	**1.2 Designation:** Medical Officer

2. General

**2.1**	How long have you been working in this health facility? (months/years)	It’s been too many years
**2.2**	Total months/years of service	5 years
**2.3**	What are your current roles and responsibility with respect to maternal and neonatal care?<br>My responsibility is of the general OPD along with it management of the facility as presently here no SMO is available	
**2.4**	How many deliveries and resuscitations of newborns have you attended in last 1 month?<br>NA	
	A. No. of deliveries attended in last 1 month	NA
	B. No. of newborn resuscitations attended in last 1 month	NA

3. Service Delivery

3.1 In routine practice, which health staff performs the following services?

Services	Staff performing the services
Delivery without complication	Nurse
Delivery with complication/ high risk delivery	Referred
Caesarean section	Referred
Newborn care at birth	Nurse
Sick newborn care	Referred
Breastfeeding support	Nurse

3.2 What are the challenges faced by you and your colleagues for delivering the desired mother and newborn care services?

Challenges faced	How do you manage these challenges
Infrastructure Here the infrastructure is good we have adequate space also.	
Equipment Here we don’t have Ultrasound machine which I think is very much necessary.	We tell them to do from only what we can do.
Drugs and supplies Everything is available there is no issue in that	
Support services	
Other Here there is no LMO due to which most of the cases are referred which can be easily managed here.	We try to do only normal cases after seeing the USG if there is any slight problem we refer as we can’t take risk in the unavailability if the Dr.

3.3 What challenges do you face while delivering essential newborn care services and how do you manage these?

Challenges faced	How do you manage these challenges
Care at delivery There is no challenge other than the unavailability of permanent LMO	Nurses are managing, I use to write the prescriptions on the card
Care in the ward Here people are not willing t stay for long after the delivery; they start saying next day that they want to go home.	We tell them that if you want to go it will be on your risk and if they still want to go they we do LAMA and send them
Care of sick newborns No specialist Dr. for the newborn	Referred to DH

3.4 What challenges do you face while delivery of pregnant women?

Challenges faced	How do you manage these challenges
Delivery without complication	NA
Delivery with complication	NA
Caesarean section	NA
Referred cases with complication	NA

3.5 How long usually the mothers stay at the facility after the delivery?

	Normal Delivery	NA
	Caesarean Delivery	NA

**4. Manpower**	
**4.1**	How many positions of doctors are lying vacant in your health facility<br>Here many posts are vacant for Dr. may be in few days we may recruit also.
**4.2**	If there is a shortage of manpower who addresses the issue so that is does not hinders routine work?<br>It is adjusted among each other
**4.3**	Do you have adequate staff inside labor room, ANC clinic and SNCU's?<br>Yes nursing staff is adequate
**4.4**	What happens if a particular department is having more flow of patients? Is there any flexibility in assigning inter departmental responsibilities among staff?<br>Here we have one area only we admit there. Very rarely only we have more patient flow here if it exceeds the bed limits then we admit on one bed 2 patient each.
**4.5**	What is the mechanism of taking leave and who sanctions it?
**5. Duty Roster**	
**5.1**	Who prepares the duty roster for you?<br>There is no duty roster for me
**5.2**	Who follows up the prepared roster so that the shifts are routinely changed?<br>Dr. has been given that charge now
**5.3**	How many Medical Officers are posted at one time in your department? What is the pattern of shift?<br>Here we have 2 MOs. Shift is mostly morning and one MO will be on call for emergency
**5.4**	Do you have flexibility in changing the shifts?
**5.5**	How do you manage when you have double shifts?
**6. Infrastructure**	
**6.1**	Do you have space to accommodate changes inside the department?<br>Yes we have enough space here
**6.2**	Do you have enough beds to accommodate increased number of patients?<br>No our beds are limited only and actually very rarely we can see any increased number of patients. If it happens we admit on one bed 2 patients each
**6.3**	Is their regular power supply and clean water for drinking? Any substitute available in case of power cut or irregular water supply?<br>Yes we don't face any issues of electricity & water supply.
**7. Training /Skills**	
**7.1**	How many of the total staff are trained for MCH services?<br>Staff nurses here all are trained
**7.2**	Is there any pre job posting training for newly joined staff?
**7.3**	Is there any on- job training for the staff?<br>Yes
**7.4**	Please let us know about the last training on attended by you?<br>I didn't attend any such sort of trainings

**7.4**	Timing (Month/Year)	
	Place	
	Duration (in days)	
	What did you like the most in the training?	
	What did you dislike the most in the training?	
	What was the training methodology used (Lectures/ Hands-on / Practical's)?	
	Who conducts the workshop? Who prepares roster for workshop/training and how it is notified? How it is monitored?	
**7.5**	What are the opportunities and mechanisms currently in place/adopted to retain the skills of Nurses/ANMs/Doctors?	

	Here there is no opportunity due to limited number of staff
**7.6**	How the training related to care during delivery and newborn period can be further improved?
**7.7**	Have you ever visited/attended the skill labs operational in your district? Where it was conducted? Who conducted it? What was the time duration of skill lab training?
**7.8**	What are the good things about this skill lab?
**7.9**	What are the challenges related to skill lab?
**7.10**	In your opinion, how many health staffs might have used or visited the skill labs?
**7.11**	Did somebody advise or persuade you to attend the skill lab?
**8. Referral services**	
**8.1**	In what situation usually the newborns/mothers (pregnant/recently delivered) are referred to the next level of healthcare?<br>Here we take only normal delivery cases which can be managed by the nurses if there is any complications that we see in USG or blood report then we refer
**8.2**	Where the newborns/ pregnant women/ mothers are usually referred, what is the usual mode of transportation and how long does it takes to reach the next level health facility in your area?<br>We refer to DH in ambulance with the EMT staff. It takes half an hour from here
**8.3**	What facilitation is done from facility side for referral and what difficulties/challenges do you face while transporting the sick newborn and mother to next level? *(Probe: monetary/logistics)*<br>I didn't face any challenges though. Everything is managed here easily
**8.4**	What are the challenges faced related to referral transport experienced by this facility and how are they handled?<br>Sometimes if ambulance is not available patient has to wait.
**9. Logistics**	
**9.1**	Are you aware of any shortage/irregular supply of drugs and/or supplies needed for care during delivery and newborn period in the last one year? What were the reasons for this shortage and how these situations were managed?<br>Here there is no shortage of medicines
**9.2**	How frequently the families/ parents asked to procure drugs from outside/ store?<br>It happens rarely, in case if supply is finished from our side due to excessive case load then only they have to buy from outside
**9.3**	How many equipments essential for management of delivery or newborn care are out of order at this moment?<br>All the equipments are functional
**9.4**	What is the usual mechanism of repair and maintenance of these equipments? *(probe: who is responsible and what is the duration of repair*)<br>Biomedical engineer from DH is informed about it and he use to come and repair it.
**9.5**	What are the supervisory mechanisms in place at present for maternal and newborn care services?
	Who supervises<br>Nursing staff
	What is the frequency of supervisory visits<br>Whenever it's necessary. If there is any sick case here then I also go for supervision
	Is any feedback/report provided usually after the supervision?<br>No
	What actions are taken after last supervisory visit?<br>We decide if the case is needed for discharge or referral
**9.6**	Please let us know about the last supervisory visit to the facility related to maternal and newborn care services?
	Who came for last supervisory visit?
	How long ago the supervisory visit took place?
	What all components were observed?
	What feedback was given and what actions were taken?

10. Perceptions regarding Quality of care	
**10.1**	According to you, what is the meaning of quality?<br>Quality means using our available resources to provide better care
**10.2**	According to you, what are the issues that affect the quality of health services?<br>Unavailability of staff such as Dr, Class 4, sweeper etc is here. Other than this there is no good knowledge amongst the staff as they are only learning what they are doing daily. There should be ultrasound machine here as they have to spend money for this at private.
**10.3**	What can you do to improve the quality of the health services?<br>Obviously staff strength improvement. Then there should be one mechanism like for USG there should be 1 hour kept vacant for bawal cases who has to do USG and according to that cases of USG will be called here and send to DH in ambulance or like for a day 4-5 cases for bawal should be left vacant. So that patients from here will go there and do the USG.
**10.4**	Did any of your relatives, friends or acquaintances ever availed health services at this hospital? If not, any reason?<br>No, here facilities are very limited.
**11. Others**	
**11.1**	If any shortage of blood and how is it tackled?
**11.2**	Do you arrange blood donation camps on facility basis?

ID: 1208201		
**1 Type of Health Facility : fru1, fbd**		**1.2 Designation: staff nurse**
**2. General**		
**2.1**	How long have you been working in this health facility? (months/years) -Since 2005	
**2.2**	Total months/years of service -Since 1999 (Hodal)	
**2.3**	What are your current roles and responsibility with respect to maternal and neonatal care?<br>Normal delivery , episiotomy, tear stitch , PPH management, Placenta removal , Case after birth for newborn, Marinating all register, Birth form, Death form.	
**2.4**	How many deliveries and resuscitations of newborns have you attended in last 1 month?	
	A. No. of deliveries attended in last 1 month	9
	B. No. of newborn resuscitations attended in last 1 month	_
**2.5**	Who did you receive the training from	

	Area	Training name	year
A	Care during delivery (S.B.A.)	SBA	2000
B	Neonatal Resuscitation Program (In RP / NSS)	NSSK	2007
C	Sick Newborn Care (FBNC)	FBNC/IMNCI	2012/ 2012

3. Service Delivery

3.1 What are the challenges faced by you and your colleagues for delivering the desired mother and newborn care services?

Challenges faced		
	**Mother care**	**Newborn care**
Infrastructure	Power cut, no drinking water	Power cut
Equipment	No issues	No issues
Drugs and supplies	No issues, In case of shortage give demand receive immediately	No issues
Support services	No ambulance, private ambulance reach late, shortage of ward boy, security guard.	No issues
Other		

3.2 What challenges do you face while delivering essential newborn care services and how do you manage these?

Challenges faced	How do you manage these challenges
Care at delivery No Dr. in ever night, No Dr. available on call	Call dr. & take guidance on home, Refer to BH
Care in the ward Only / Nurse , Difficult to manage	Call ward boy for help
Care of sick newborns<br>Dr. not available in evening & night	Refer to BH

3.3 What challenges do you face while delivering essential newborn care services and how do you manage these?

Challenges faced	How do you manage these challenges
Delivery without complication	No issues
Delivery with complication	PPH, Eclampsia , breach
Caesarean section	Done by Dr. only, so no Problem
Referred cases with complication	Ambulance do not reach on time

3.4 How long usually the mothers stay at the facility after the delivery?

Normal Delivery	48-72 hrs
Caesarean Delivery	4-5 days

**4. Manpower**		
**4.1**	How many posts of Staff Nurse / ANM are vacant in your health facility?<br>1 Staff Nurse is short, Need more staff nurse	
**4.2**	What difficulties do you face in providing mother and newborn care services to existing employees? (Doctors, nurses and other staff)<br>No issues only ward boy enter labor room	
**4.3**	What is the mechanism of taking leave and who sanctions it?<br>Yes, done at B.H. Hospital	
**5. Duty Roster**		
**5.1**	Who prepares the duty roster for you?<br>Nursing sister is responsible for roster	
**5.2**	Do you have flexibility in changing the shifts?<br>Yes, ask nursing sister to change	
**5.3**	How do you manage when you have double shifts?<br>All staff want for interview, residence is upstairs so ward boy call if patient arrives.	
**5.4**	What is the procedure for taking leave and who approves it?<br>CL is available to Dr. Rita, signed by MOIC & nursing sister	
**5.5**	Who prepares rosters for emergency / regular service?<br>Made by nursing sister	
**6. Infrastructure**		
**6.1**	Do you have space to accommodate changes inside the department?<br>Yes, changes cab by done	
**6.2**	Do you have enough beds to accommodate increased number of patients?<br>Have extra beds in children ward, although it req. occasionally	
**6.3**	Is their regular power supply and clean water for drinking? Any substitute available in case of power cut or irregular water supply?<br>Occasionally power cut, no drinking water	
**7. Data management**		
**7.1**	How do you record data?<br>Done on register, monthly	
**7.2**	How do you maintain a register?<br>Immediately after delivery, most by at same time, monthly data attend like, PP/UCD, referral , copper T, ambulance	
**7.3**	Where do you send the record?<br>Sent to BH hospital	
**7.4**	How often is the data sent?<br>Every month end.	
**8. Blood bank**		
**8.1**	How long does it take for a needy person to get blood?<br>Refer to B.H. never arrange here	
**9. Training /Skills**		
**9.1**	Could you tell us about your previous NSSK / Neonatal Resuscitation Training?	
	Time (month / year)	2007
	place	BH hospital
	Duration (in days)	3 days
**9.2**	Who conducts the workshop? Who prepares roster for workshop/training and how it is notified? How it is monitored?<br>Dr. prepare roster and notify lost of training prepared at B.H. hospital	
**9.3**	What did you like the most in the training?<br>Food is good, setup of instrument is good , get to learn new things, get money	

.

**9.4**	What did you dislike the most in the training?<br>Training is good
**9.5**	What was the training methodology used (Lectures/ Hands-on / Practical's)?<br>Lecturer , books, exam at start & end, hands on practical
**9.6**	How did you like meeting / interacting with the trainers?<br>All are good, Dr. is best

**9.7**	What was their level of knowledge / skills?	Excellent knowledge
	How was your conversation with him	It was good

**9.8**	What are the opportunities and mechanisms currently in place/adopted to retain the skills of Nurses/ANMs/Doctors?<br>Only training
**9.9**	What challenges do you have with the skills of nurses and the support of staff in delivery rooms, perinatal wards and newborn care units? In your opinion, how can this be controlled?<br>All are good, no issues held , only if sweeper is absent , then it is difficult
**9.10**	how the training related to care during delivery and newborn period can be further improved?<br>As of now , it was good, no change is required
**9.11**	Have you been to a skill lab set up in your district?<br>Yes went there twice at B.H. hospital
**9.12**	What are the good things about this skill lab?<br>HB test, procedures were reviewed trainer ware good, equipments , were good
**9.13**	What are the challenges related to skill lab?<br>No challenges
**9.14**	In your opinion, how many health staffs might have used or visited the skill labs?<br>Almost everyone from FRU-1
**9.15**	Did somebody advise or persuade you to attend the skill lab?<br>Got notification from Dr.
**9.16**	How does the Skill Lab help in Neonatal Resuscitation and Neonatal Care?<br>Yes, it is helpful, child get benefits
**10. Referral services**	
**10.1**	In what situation usually the newborns/mothers (pregnant/recently delivered) are referred to the next level of healthcare?<br>PPH, severe anemia, eclampsia , pre term, birth asphyxia , congenital abnormally , feeding issues
**10.2**	Where the newborns/ pregnant women/ mothers are usually referred, what is the usual mode of transportation and how long does it takes to reach the next level health facility in your area?<br>B.H. Hospital, call 10-02-2015, 20 min.
**10.3**	What facilitation is done from facility side for referral and what difficulties/challenges do you face while transporting the sick newborn and mother to next level? *(Probe: monetary/logistics)*<br>Stabilize the child, then refer, dr. is not present every time, so it is issue
**10.4**	What are the challenges faced related to referral transport experienced by this facility and how are they handled?<br>Call 102, if not on time then call auto, patient for auto rickshaws
**11. Logistics**	
**11.1**	Are you familiar with any scarcity / irregular supply of medicines and / or supplies required for care during delivery and newborn in the last one year? What were the reasons for this deficiency and how were these conditions managed?<br>No shortage, always available on time
**11.2**	How frequently the families/ parents asked to procure drugs from outside/ store?<br>Rarely , if pharmacy is closed
**11.3**	What are the supervisory mechanisms in place at present for maternal and newborn care services?
	Who supervises

	CMO, usually do visits other Dr. from B.K.
	What is the frequency of supervisory visits<br>Anytime without notification. monthly
	Is any feedback/report provided usually after the supervision?<br>No reporting , shortenings are inform to Dr. she inform others on next day
	What actions are taken after last supervisory visit?<br>Yes, register were not maintained no partograph, poor cleanliness , action were taken immediately to improve.
**11.4**	Please let us know about the last supervisory visit to the facility related to maternal and newborn care services?
	Who came for last supervisory visit?<br>DAM, DPM
	How long ago the supervisory visit took place?<br>2 days, but not severe as she was on leave
	What all components were observed?<br>Labour room, register, PNC ward, toilets
	What feedback was given and what actions were taken?<br>Took photographs & asked to take immediate action , sick were not clean , nursing stations register to complete entries
**12. others**	
**12.1**	How wide do the events in discharge / record slip?<br>Write everything, date of admit to date of discharge, everything is recorded written
**12.2**	In your view, what are the perceived barriers between families in the use of public health services for newborns of pregnant women?<br>No such issues , they don't as cut to go to B.H. because of crowding<br>How can these obstacles be overcome?<br>Convince them to refer in case of severe case
**12.3**	How much additional effort is needed to reduce the neonatal mortality rate in your area?<br>Need more doctor's they can manage all emergency , at present , no dr. is available on call
**12.4**	According to you, what is the meaning of quality?
**12.5**	According to you, what are the issues affecting the quality (quality) of health services?
**12.6**	What can you do to improve the quality of the health services?
**12.7**	Did any of your relatives, friends or acquaintances ever availed health services at this hospital? If not, any reason?

.

ID: 1208202				
**1.1 Type of Health Facility : FRU 1, FBD**			**1.2 Designation: STAFF NURSE**	
**2. General**				
**2.1**	How long have you been working in this health facility? (months/years)- Since 2000			
**2.2**	Total months/years of service - Since 2000 , joined in same facility			
**2.3**	What are your current roles and responsibility with respect to maternal and neonatal care?<br>Record review , maintenance of birth record, daily register, maintain list of equipments, functionality of equipments, preparing roster for S/N, ward boy, class iv, all reports, cleanliness , promote family planning case, PPIUCD insertion			
**2.4**	How many deliveries and resuscitations of newborns have you attended in last 1 month?			
	A. No. of deliveries attended in last 1 month			80-90
	B. No. of newborn resuscitations attended in last 1 month			1
**2.5**	Who did you receive the training from:			
	Area		Training name	Year
	A	Care during delivery (S.B.A.)	SBA	2001
	B	Neonatal Resuscitation Program (In RP / NSS)	NSSK/NRP	2007/2009
	C	Sick Newborn Care (FBNC)	FBNC	2012

3. Service Delivery

3.1 What are the challenges faced by you and your colleagues for delivering the desired mother and newborn care services?

Challenges faced		
	**Mother care**	**Newborn care**
Infrastructure	No problem, space is enough	No issues
Equipment	Need a separate dress all other equipments available & functional	All equipments are available & functional
Drugs and supplies	No issue enough staff, drug supply is regular	No issues
Support services	No ambulance , diet should change, patient should get khichdi, daliya	No issues
Other		

3.2 What challenges do you face while delivering essential newborn care services and how do you manage these?

Challenges faced	How do you manage these challenges
Care at delivery<br>24 x7 permanent dr. have to refer 25% patients cog. Dr. are on call, no dr. came on call	Usually refer to higher facility
Care in the ward<br>Water is not available for patient	Attendant on ward boy & nurse get water from downstairs
Care of sick newborns<br>Shortage of Dr. after OPD timings	Usually refer to B.H

3.3 What challenges do you face while delivery of pregnant women?

Challenges faced	How do you manage these challenges
Delivery without complication	Done by S/N , no issues , at delivery , only nurse manage

		mother & newborn
	Delivery with complication	Dr.s are not available cannot manage alone
	Caesarean section	No C/S in evening & night due to unavailability of doctor
	Referred cases with complication	Dr. not get ambulance on time, w/boy is sent to BH by auto rickshaw
**3.4**	How long usually the mothers stay at the facility after the delivery?	
	Normal Delivery	48-72 hrs (min 48 hrs)
	Caesarean Delivery	4-5 days, If patient to leave then he write over register
**4. Manpower**		
**4.1**	How many posts of Staff Nurse / ANM are vacant in your health facility?<br>Yes, it is in demand , need at least 2 more staff nurse, S/N is deputed to FRU-2,<br>only ANM who recently joined , need 3 more ANM	
**4.2**	What difficulties do you face in providing mother and newborn care services to existing employees? (Doctors, nurses and other staff)<br>Occasionally have problem when staff nurse is on leave, all staff members are supportive	
**4.3**	What is the mechanism of taking leave and who sanctions it?<br>Yes, they get training at B.H. hospital	
**5. Duty Roster**		
**5.1**	Who prepares the duty roster for you?<br>Roster is prepared by herself , she maker duly roster for others	
**5.2**	Do you have flexibility in changing the shifts?<br>She can change but with permission from in charge or doctor on call	
**5.3**	How do you manage when you have double shifts?<br>Never needed, other staff nurse are available.	
**5.4**	What is the procedure for taking leave and who approves it?<br>CL's & EL'S are available , take permission from MOIC	
**5.5**	Who prepares rosters for emergency / regular service?<br>She her staff male register for all staff	
**6. Infrastructure**		
**6.1**	Do you have space to accommodate changes inside the department?<br>Yes, changes are possible, have good space	
**6.2**	Do you have enough beds to accommodate increased number of patients?<br>Yes, we have 30 beds, extra space can be made are to need, although never required	
**6.3**	Is their regular power supply and clean water for drinking? Any substitute available in case of power cut or irregular water supply?<br>Power cut is minimum, generally 1-2 hrs, it can by managed by invertors, drinking water only for staff & patient need water cooler	
**7. Data management**		
**7.1**	How do you record data?<br>Have separate register of delivery, admin, daily, newborn, NBCC, NBSUI, Copper T, PPIUCD, MTD, refer, complaint, call register, expense register	
**7.2**	How do you maintain a register?<br>All registers are updated regularly on daily basis.	
**7.3**	Where do you send the record?<br>Send during yearly audit to NHM staff & karyon govt. / delivery & newborn data to B.H. hospital, DHSI -2 is sent directly to CHD every month	
**7.4**	How often is the data sent?<br>Every month , quarterly reports	
**8. Blood bank**		

.

**8.1**	How long does it take for a needy person to get blood?<br>Can arrange in 2-3 hrs in morning hrs. Take time in evening & night so they refer to B.H.	
**9. Training /Skills**		
**9.1**	Could you tell us about your previous NSSK / Neonatal Resuscitation Training?	
	Time (month / year)	2007
	place	Dist Hospital
	Duration (in days)	2 days
**9.2**	Who conducts the workshop? Who prepares roster for workshop/training and how it is notified? How it is monitored?<br>She herself prepare roster for training also inform higher officials about who is left for training	
**9.3**	What did you like the most in the training?<br>Get to learn new things & attain more knowledge	
**9.4**	What did you dislike the most in the training?<br>Food is very oily, quality of rice is not good, and tea is not good, no inverter in case of power cut.	
**9.5**	what was the training methodology used (Lectures/ Hands-on / Practical's)?<br>Modules , practical, hands on session, power point based lectures.	
**9.6**	How did you like meeting / interacting with the trainers?<br>They are good & satisfactory, always get good trainings.	
**9.7**	What was their level of knowledge / skills?	Had moderate knowledge , Dr. anup had high knowledge
	How was your conversation with him	Experience was good, especially with doctor
**9.8**	What are the opportunities and mechanisms currently in place/adopted to retain the skills of Nurses/ANMs/Doctors?<br>Nothing as of now, no training is conducted since more than 1 years. There should be regular Training	
**9.9**	What challenges do you have with the skills of nurses and the support of staff in delivery rooms, perinatal wards and newborn care units? In your opinion, how can this are controlled?<br>Regular training should be done, usually call to CMO office to arrange training of staff.	
**9.10**	How the training related to care during delivery and newborn period can be further improved?<br>Use live newborn & live delivery to training staff instead of conducting training on dummier, trained doctors should conduct session.	
**9.11**	Have you been to a skill lab set up in your district?<br>At B.H. Hospital, At PIO office	
**9.12**	What are the good things about this skill lab?<br>Arrangement was good.	
**9.13**	What are the challenges related to skill lab?<br>No challenges	
**9.14**	In your opinion, how many health staffs might have used or visited the skill labs?<br>Still has training was done from everyone , but never implemented	
**9.15**	Did somebody advise or persuade you to attend the skill lab?<br>From COM office	
**9.16**	How does the Skill Lab help in Neonatal Resuscitation and Neonatal Care?<br>Was beneficial beet it should be done on live newborns.	
**10. Referral services**		
**10.1**	What situation usually the newborns/mothers (pregnant/recently delivered) are referred to the next level of healthcare?<br>Obs labour , prev LS, PPH, not progress , short stretcher units LS/CS, severe anemia<br>Birth asphyxia, severe jaundice, anomalies	

.

**10.2**	Where the newborns/ pregnant women/ mothers are usually referred, what is the usual mode of transportation and how long does it takes to reach the next level health facility in your area?<br>B.H. hospital, call 102 or if patient want then they take personal vehicle.
**10.3**	What facilitation is done from facility side for referral and what difficulties/challenges do you face while transporting the sick newborn and mother to next level? *(Probe: monetary/logistics)*<br>Sometimes ambulance is not available.
**10.4**	What are the challenges faced related to referral transport experienced by this facility and how are they handled?<br>In that case, staff arrange auto & send ward boy or S/N in case of severe case. Patient can arrange private vehicle.
**11. Logistics**	
**11.1**	Are you familiar with any scarcity / irregular supply of medicines and / or supplies required for care during delivery and newborn in the last one year? What were the reasons for this deficiency and how were these conditions managed?<br>No shortage since 1 year
**11.2**	How frequently the families/ parents asked to procure drugs from outside/ store?<br>Sometimes but she never prescribed anyone, especially in morning hours.
**11.3**	What are the supervisory mechanisms in place at present for maternal and newborn care services?<br>Yes, but not regular
	Who supervises<br>CMO, NHM team
	What is the frequency of supervisory visits<br>Not scheduled can be delayed or happen anytime.
	Is any feedback/report provided usually after the supervision?<br>Yes, to NHM, send a copy & ask to submit update
	What actions are taken after last supervisory visit?<br>Yes, action is taken memo is given & ask for updates.
**11.4**	Please let us know about the last supervisory visit to the facility related to maternal and newborn care services?
	Who came for last supervisory visit?<br>DAM in a/april 2017, urban consultant
	How long ago the supervisory visit took place?<br>3 hrs
	What all components were observed?<br>Labour room, ward, tailed , OPD
	What feedback was given and what actions were taken?<br>It was gen visit, took photographs. & send a report for up gradation , actions were taken immediately
**12. others**	
**12.1**	How wide do the events in discharge / record slip?<br>Write everything in detail
**12.2**	In your view, what are the perceived barriers between families in the use of public health services for newborns of pregnant women?<br>Need kitchen for them, no one at gate to guide, no water, relatives sometime comes upstairs to call us as they are not aware that it is residence.<br>How can these obstacles be overcome?<br>Call to duty nurse if someone come upstairs.
**12.3**	How much additional effort is needed to reduce the neonatal mortality rate in your area?<br>24x7 doctors either MO or specialist should be available, ambulance for quick services. Some patients died because ambulance was available.

.

**12.4**	According to you, what is the meaning of quality?
**12.5**	According to you, what are the issues affecting the quality (quality) of health services?
**12.6**	What can you do to improve the quality of the health services?
**12.7**	Did any of your relatives, friends or acquaintances ever availed health services at this hospital? If not, any reason?

.

ID: 1308101		
**1.1 Type of Health Facility: FRU2 , REWARI**	**1.2 Designation:** Medical Officer	
**2. General**		
**2.1**	How long have you been working in this health facility? (months/years)	8 years.
**2.2**	Total months/years of service	36 years
**2.3**	What are your current roles and responsibility with respect to maternal and neonatal care?<br>I work in emergency at delivery and caesarean, I do run regular OPD (paed). I attend outreach community program, I also do other program like IDCF, IPPI. I work as a NSSK trainer at DI office, I work any form of immunization.	
**2.4**	How many deliveries and resuscitations of newborns have you attended in last 1 month?	
	A. No. of deliveries attended in last 1 month	
	B. No. of newborn resuscitations attended in last 1 month	2
**3. Service Delivery**		
**3.1**	In routine practice, which health staff performs the following services?	
	**Services**	**Staff performing the services**
	Delivery without complication	NA
	Delivery with complication/ high risk delivery	NA
	Caesarean section	NA
	Newborn care at birth	Only room in facility care at maternity ward- Doctor ward/ Nurse
	Sick newborn care	We try to first stabilize the sick baby. If baby is very serious then this condition I refer patient to BK Hospital.
	Breastfeeding support	Nurse does it
**3.2**	What are the challenges faced by you and your colleagues for delivering the desired mother and newborn care services?	
	**Challenges faced**	**How do you manage these challenges**
	Infrastructure; there are problem of water Shortage, irregular power cut, and oxygen shortage	I keep bucket of water, Use inverter to support, Use cylinder for oxygen. Otherwise I refer baby.
	Equipment; Mostly Working	If equipment doesn't function, I call Bio-medical engineer, it takes 7 to 15 days.
	Drugs and supplies ; only vitamin K injection is not available	Patients purchase from outside,
	Support services; All available, no issues.	
	Other; There is no logistic support in immunization.	Recall patient.
**3.3**	What challenges do you face while delivering essential newborn care services and how do you manage these?	
	**Challenges faced**	**How do you manage these challenges**
	Care at delivery It is manageable, once baby cry.	
	Care in the ward There is no water and no proper light in the ward.	
	Care of sick newborns In case of delayed cry, chance of convulsion increases. There is no Laboratory support.	Refer to Bk
**3.4**	What challenges do you face while delivery of pregnant women?	
	**Challenges faced**	**How do you manage these challenges**
	Delivery without complication	NA
	Delivery with complication	NA
	Caesarean section	NA
	Referred cases with complication	NA

**3.5**	How long usually the mothers stay at the facility after the delivery?	
	Normal Delivery	NA
	Caesarean Delivery	NA

**4. Manpower**	
**4.1**	How many positions of doctors are lying vacant in your health facility?
**4.2**	If there is a shortage of manpower who addresses the issue so that is does not hinders routine work?<br>We convey to CMO, MOIC about all the issues, They address the issues. Problems and issues are discussed in meeting.
**4.3**	Do you have adequate staff inside labor room, ANC clinic and SNCU's?<br>No, there is no dedicated staff inside LR, ANC and NBSU. Maternity Nurses monitor baby at NBSU.
**4.4**	What happens if a particular department is having more flow of patients? Is there any flexibility in assigning inter departmental responsibilities among staff?
**4.5**	What is the mechanism of taking leave and who sanctions it?<br>CMO sir sanction leave, however there are also online system of taking leave.
**5. Duty Roster**	
**5.1**	Who prepares the duty roster for you?<br>There is no duty roster available, we are available at round the clock.
**5.2**	Who follows up the prepared roster so that the shifts are routinely changed?<br>NA
**5.3**	How many Medical Officers are posted at one time in your department? What is the pattern of shift?<br>There No medical officer here.
**5.4**	Do you have flexibility in changing the shifts?<br>No, we don't do our duty shift wise. We work round the clock. In case of any emergency I come on call. My residence is at hospital premises.
**5.5**	How do you manage when you have double shifts?<br>NA.
**6. Infrastructure**	
**6.1**	Do you have space to accommodate changes inside the department?<br>Yes,
**6.2**	Do you have enough beds to accommodate increased number of patients?<br>Yes beds are enough but we don't need to increase number of beds,
**6.3**	Is their regular power supply and clean water for drinking? Any substitute available in case of power cut or irregular water supply?<br>Inverters are installed but it run for short period, a water cooler is
**7. Training /Skills**	
**7.1**	How many of the total staff are trained for MCH services?<br>They all are trained.
**7.2**	Is there any pre job posting training for newly joined staff?<br>No. Induction program is facilitated at BK.
**7.3**	Is there any on- job training for the staff?<br>Yes.
**7.4**	Please let us know about the last training on attended by you?<br>I had attended NRC training.

**7.4**	Timing (Month/Year)	2015
	Place	Kalawati Hospital, Delhi
	Duration (in days)	3 days
	What did you like the most in the training?<br>Demo was good, I had learned new things. I have learned formulae for feeding the malnourished children.	
	What did you dislike the most in the training?	

	Everything was fine. I enjoyed
	What was the training methodology used (Lectures/ Hands-on / Practical's)?<br>Lectures, live demo Group discussion Pre and post test.
	Who conducts the workshop? Who prepares roster for workshop/training and how it is notified? How it is monitored?<br>State team has conducted this training. They notify via email to districts.
**7.5**	What are the opportunities and mechanisms currently in place/adopted to retain the skills of Nurses/ANMs/Doctors?<br>Group discussion among staff and day to day meeting can be arranged.
**7.6**	How the training related to care during delivery and newborn period can be further improved?
**7.7**	Have you ever visited/attended the skill labs operational in your district? Where it was conducted? Who conducted it? What was the time duration of skill lab training?<br>I never attended the skill labs training.
**7.8**	What are the good things about this skill lab?<br>NA
**7.9**	What are the challenges related to skill lab?<br>NA
**7.10**	In your opinion, how many health staffs might have used or visited the skill labs?<br>NA
**7.11**	Did somebody advise or persuade you to attend the skill lab?<br>NA
**8. Referral services**	
**8.1**	In what situation usually the newborns/mothers (pregnant/recently delivered) are referred to the next level of healthcare?<br>Severe birth asphyxia, convulsion, congenital malformation.
**8.2**	Where the newborns/ pregnant women/ mothers are usually referred, what is the usual mode of transportation and how long does it takes to reach the next level health facility in your area?<br>Newborns/ pregnant women are usually referred to BK Hospital, They reach hospital by ambulance.
**8.3**	What facilitation is done from facility side for referral and what difficulties/challenges do you face while transporting the sick newborn and mother to next level? *(Probe: monetary/logistics)*<br>There is no issue, EMT takes patient to hospital.
**8.4**	What are the challenges faced related to referral transport experienced by this facility and how are they handled?<br>Sometimes ambulance is not available at time also it takes time to reach hospital.
**9. Logistics**	
**9.1**	Are you aware of any shortage/irregular supply of drugs and/or supplies needed for care during delivery and newborn period in the last one year? What were the reasons for this shortage and how these situations were managed?<br>Only Vit K is not available. There is no need of vit. K for every child, if required then patients purchase it from outside. MOIC knows the standard protocol.
**9.2**	How frequently the families/ parents asked to procure drugs from outside/ store?<br>It happens very rarely.
**9.3**	How many equipments essential for management of delivery or newborn care are out of order at this moment?<br>All essential equipments for management of delivery or newborn care are there at hospital.
**9.4**	What is the usual mechanism of repair and maintenance of these equipments? *(probe: who is responsible and what is the duration of repair*)<br>I call bio medical engineer from BK. It takes minimum 10 days to repair.
**9.5**	What are the supervisory mechanisms in place at present for maternal and newborn care services?
	Who supervises<br>State head quarter, central team and NHM

	What is the frequency of supervisory visits<br>Almost in six months.
	Is any feedback/report provided usually after the supervision?<br>Yes, Report is prepared.
	What actions are taken after last supervisory visit?<br>They take actions if found any issues,
**9.6**	Please let us know about the last supervisory visit to the facility related to maternal and newborn care services?
	Who came for last supervisory visit?<br>Central govt team from mohfw
	How long ago the supervisory visit took place?<br>It has been 2 months.
	What all components were observed?<br>Maternity ward, child health service, OPD
	What feedback was given and what actions were taken?
**10. Perceptions regarding Quality of care**	
**10.1**	According to you, what is the meaning of quality?<br>Health services with complete satisfaction of patients.
**10.2**	According to you, what are the issues that affect the quality of health services?<br>Infrastructure issues, shortage of staffs, no guard, irregular power supply and water problem.
**10.3**	What can you do to improve the quality of the health services?<br>Discuss the issues and challenges, and resolve it timely.
**10.4**	Did any of your relatives, friends or acquaintances ever availed health services at this hospital? If not, any reason?<br>No, There is no proper facility t hospital, who will take risk for their own relatives.
**11. Others**	
**11.1**	If any shortage of blood and how is it tackled?<br>There is no blood bank here; Patients get it from BK hospital. we refer them
**11.2**	Do you arrange blood donation camps on facility basis?<br>No.

ID: 1208201	
**1 Type of Health Facility : fru1, fbd**	**1.2 Designation: staff nurse**

2. General

**2.1**	How long have you been working in this health facility? (months/years) -Since 2005
**2.2**	Total months/years of service -Since 1999 (Hodal)
**2.3**	What are your current roles and responsibility with respect to maternal and neonatal care?<br>Normal delivery , episiotomy, tear stitch , PPH management, Placenta removal , Case after birth for newborn, Marinating all register, Birth form, Death form.
**2.4**	How many deliveries and resuscitations of newborns have you attended in last 1 month?

A. No. of deliveries attended in last 1 month	9
B. No. of newborn resuscitations attended in last 1 month	_

2.5 Who did you receive the training from

	Area	Training name	year
A	Care during delivery (S.B.A.)	SBA	2000
B	Neonatal Resuscitation Program (In RP / NSS)	NSSK	2007
C	Sick Newborn Care (FBNC)	FBNC/IMNCI	2012/ 2012

3. Service Delivery

3.1 What are the challenges faced by you and your colleagues for delivering the desired mother and newborn care services?

	Challenges faced	
	**Mother care**	**Newborn care**
Infrastructure	Power cut, no drinking water	Power cut
Equipment	No issues	No issues
Drugs and supplies	No issues, In case of shortage give demand receive immediately	No issues
Support services	No ambulance, private ambulance reach late, shortage of ward boy, security guard.	No issues
Other		

3.2 What challenges do you face while delivering essential newborn care services and how do you manage these?

Challenges faced	How do you manage these challenges
Care at delivery No Dr. in ever night, No Dr. available on call	Call dr. & take guidance on home, Refer to BH
Care in the ward Only / Nurse , Difficult to manage	Call ward boy for help
Care of sick newborns Dr. not available in evening & night	Refer to BH

3.3 What challenges do you face while delivering essential newborn care services and how do you manage these?

Challenges faced	How do you manage these challenges
Delivery without complication	No issues
Delivery with complication	PPH, Eclampsia , breach
Caesarean section	Done by Dr. only, so no Problem
Referred cases with complication	Ambulance do not reach on time

3.4 How long usually the mothers stay at the facility after the delivery?

Normal Delivery	48-72 hrs
Caesarean Delivery	4-5 days

**4. Manpower**		
**4.1**	How many posts of Staff Nurse / ANM are vacant in your health facility?<br>1 Staff Nurse is short, Need more staff nurse	
**4.2**	What difficulties do you face in providing mother and newborn care services to existing employees? (Doctors, nurses and other staff)<br>No issues only ward boy enter labor room	
**4.3**	What is the mechanism of taking leave and who sanctions it?<br>Yes, done at B.H. Hospital	
**5. Duty Roster**		
**5.1**	Who prepares the duty roster for you?<br>Nursing sister is responsible for roster	
**5.2**	Do you have flexibility in changing the shifts?<br>Yes, ask nursing sister to change	
**5.3**	How do you manage when you have double shifts?<br>All staff want for interview, residence is upstairs so ward boy call if patient arrives.	
**5.4**	What is the procedure for taking leave and who approves it?<br>CL is available to Dr. Rita, signed by MOIC & nursing sister	
**5.5**	Who prepares rosters for emergency / regular service?<br>Made by nursing sister	
**6. Infrastructure**		
**6.1**	Do you have space to accommodate changes inside the department?<br>Yes, changes cab by done	
**6.2**	Do you have enough beds to accommodate increased number of patients?<br>Have extra beds in children ward, although it req. occasionally	
**6.3**	Is their regular power supply and clean water for drinking? Any substitute available in case of power cut or irregular water supply?<br>Occasionally power cut, no drinking water	
**7. Data management**		
**7.1**	How do you record data?<br>Done on register, monthly	
**7.2**	How do you maintain a register?<br>Immediately after delivery, most by at same time, monthly data attend like, PP/UCD, referral , copper T, ambulance	
**7.3**	Where do you send the record?<br>Sent to BH hospital	
**7.4**	How often is the data sent?<br>Every month end.	
**8. Blood bank**		
**8.1**	How long does it take for a needy person to get blood?<br>Refer to B.H. never arrange here	
**9. Training /Skills**		
**9.1**	Could you tell us about your previous NSSK / Neonatal Resuscitation Training?	
	Time (month / year)	2007
	place	BH hospital
	Duration (in days)	3 days
**9.2**	Who conducts the workshop? Who prepares roster for workshop/training and how it is notified? How it is monitored?<br>Dr. prepare roster and notify lost of training prepared at B.H. hospital	
**9.3**	What did you like the most in the training?<br>Food is good, setup of instrument is good , get to learn new things, get money	

.

**9.4**	What did you dislike the most in the training?<br>Training is good	
**9.5**	What was the training methodology used (Lectures/ Hands-on / Practical's)?<br>Lecturer , books, exam at start & end, hands on practical	
**9.6**	How did you like meeting / interacting with the trainers?<br>All are good, Dr. is best	
**9.7**	What was their level of knowledge / skills?	Excellent knowledge
	How was your conversation with him	It was good
**9.8**	What are the opportunities and mechanisms currently in place/adopted to retain the skills of Nurses/ANMs/Doctors?<br>Only training	
**9.9**	What challenges do you have with the skills of nurses and the support of staff in delivery rooms, perinatal wards and newborn care units? In your opinion, how can this be controlled?<br>All are good, no issues held , only if sweeper is absent , then it is difficult	
**9.10**	how the training related to care during delivery and newborn period can be further improved?<br>As of now , it was good, no change is required	
**9.11**	Have you been to a skill lab set up in your district?<br>Yes went there twice at B.H. hospital	
**9.12**	What are the good things about this skill lab?<br>HB test, procedures were reviewed trainer ware good, equipments , were good	
**9.13**	What are the challenges related to skill lab?<br>No challenges	
**9.14**	In your opinion, how many health staffs might have used or visited the skill labs?<br>Almost everyone from FRU-1	
**9.15**	Did somebody advise or persuade you to attend the skill lab?<br>Got notification from Dr.	
**9.16**	How does the Skill Lab help in Neonatal Resuscitation and Neonatal Care?<br>Yes, it is helpful, child get benefits	
**10. Referral services**		
**10.1**	In what situation usually the newborns/mothers (pregnant/recently delivered) are referred to the next level of healthcare?<br>PPH, severe anemia, eclampsia , pre term, birth asphyxia , congenital abnormally , feeding issues	
**10.2**	Where the newborns/ pregnant women/ mothers are usually referred, what is the usual mode of transportation and how long does it takes to reach the next level health facility in your area?<br>B.H. Hospital, call 10-02-2015, 20 min.	
**10.3**	What facilitation is done from facility side for referral and what difficulties/challenges do you face while transporting the sick newborn and mother to next level? *(Probe: monetary/logistics)*<br>Stabilize the child, then refer, dr. is not present every time, so it is issue	
**10.4**	What are the challenges faced related to referral transport experienced by this facility and how are they handled?<br>Call 102, if not on time then call auto, patient for auto rickshaws	
**11. Logistics**		
**11.1**	Are you familiar with any scarcity / irregular supply of medicines and / or supplies required for care during delivery and newborn in the last one year? What were the reasons for this deficiency and how were these conditions managed?<br>No shortage, always available on time	
**11.2**	How frequently the families/ parents asked to procure drugs from outside/ store?<br>Rarely , if pharmacy is closed	
**11.3**	What are the supervisory mechanisms in place at present for maternal and newborn care services?	
	Who supervises	

.

	CMO, usually do visits other Dr. from B.K.
	What is the frequency of supervisory visits<br>Anytime without notification. monthly
	Is any feedback/report provided usually after the supervision?<br>No reporting , shortenings are inform to Dr. she inform others on next day
	What actions are taken after last supervisory visit?<br>Yes, register were not maintained no partograph, poor cleanliness , action were taken immediately to improve.
**11.4**	Please let us know about the last supervisory visit to the facility related to maternal and newborn care services?
	Who came for last supervisory visit?<br>DAM, DPM
	How long ago the supervisory visit took place?<br>2 days, but not severe as she was on leave
	What all components were observed?<br>Labour room, register, PNC ward, toilets
	What feedback was given and what actions were taken?<br>Took photographs & asked to take immediate action , sick were not clean , nursing stations register to complete entries
**12. others**	
**12.1**	How wide do the events in discharge / record slip?<br>Write everything, date of admit to date of discharge, everything is recorded written
**12.2**	In your view, what are the perceived barriers between families in the use of public health services for newborns of pregnant women?<br>No such issues , they don't as cut to go to B.H. because of crowding<br>How can these obstacles be overcome?<br>Convince them to refer in case of severe case
**12.3**	How much additional effort is needed to reduce the neonatal mortality rate in your area?<br>Need more doctor's they can manage all emergency , at present , no dr. is available on call
**12.4**	According to you, what is the meaning of quality?
**12.5**	According to you, what are the issues affecting the quality (quality) of health services?
**12.6**	What can you do to improve the quality of the health services?
**12.7**	Did any of your relatives, friends or acquaintances ever availed health services at this hospital? If not, any reason?

<table>
<tr><td colspan="3">ID: 1208202</td></tr>
<tr><td colspan="3">1.1 Type of Health Facility : FRU 1, FBD   1.2 Designation: STAFF NURSE</td></tr>
<tr><td colspan="3">2. General</td></tr>
<tr><td>2.1</td><td colspan="2">How long have you been working in this health facility? (months/years)- Since 2000</td></tr>
<tr><td>2.2</td><td colspan="2">Total months/years of service - Since 2000 , joined in same facility</td></tr>
<tr><td>2.3</td><td colspan="2">What are your current roles and responsibility with respect to maternal and neonatal care?
Record review , maintenance of birth record, daily register, maintain list of equipments, functionality of equipments, preparing roster for S/N, ward boy, class iv, all reports, cleanliness , promote family planning case, PPIUCD insertion</td></tr>
<tr><td>2.4</td><td colspan="2">How many deliveries and resuscitations of newborns have you attended in last 1 month?</td></tr>
<tr><td></td><td>A. No. of deliveries attended in last 1 month</td><td>80-90</td></tr>
<tr><td></td><td>B. No. of newborn resuscitations attended in last 1 month</td><td>1</td></tr>
<tr><td>2.5</td><td colspan="2">Who did you receive the training from:</td></tr>
</table>

	Area	Training name	Year
A	Care during delivery (S.B.A.)	SBA	2001
B	Neonatal Resuscitation Program (In RP / NSS)	NSSK/NRP	2007/2009
C	Sick Newborn Care (FBNC)	FBNC	2012

3. Service Delivery

3.1 What are the challenges faced by you and your colleagues for delivering the desired mother and newborn care services?

Challenges faced		
	**Mother care**	**Newborn care**
Infrastructure	No problem, space is enough	No issues
Equipment	Need a separate dress all other equipments available & functional	All equipments are available & functional
Drugs and supplies	No issue enough staff, drug supply is regular	No issues
Support services	No ambulance , diet should change, patient should get khichdi, daliya	No issues
Other		

3.2 What challenges do you face while delivering essential newborn care services and how do you manage these?

**Challenges faced**	**How do you manage these challenges**
Care at delivery<br>24 x7 permanent dr. have to refer 25% patients cog. Dr. are on call, no dr. came on call	Usually refer to higher facility
Care in the ward<br>Water is not available for patient	Attendant on ward boy & nurse get water from downstairs
Care of sick newborns<br>Shortage of Dr. after OPD timings	Usually refer to B.H

3.3 What challenges do you face while delivery of pregnant women?

**Challenges faced**	**How do you manage these challenges**
Delivery without complication	Done by S/N , no issues , at delivery , only nurse manage

		mother & newborn
	Delivery with complication	Dr.s are not available cannot manage alone
	Caesarean section	No C/S in evening & night due to unavailability of doctor
	Referred cases with complication	Dr. not get ambulance on time, w/boy is sent to BH by auto rickshaw
**3.4**	How long usually the mothers stay at the facility after the delivery?	
	Normal Delivery	48-72 hrs (min 48 hrs)
	Caesarean Delivery	4-5 days, If patient to leave then he write over register
**4. Manpower**		
**4.1**	How many posts of Staff Nurse / ANM are vacant in your health facility?<br>Yes, it is in demand , need at least 2 more staff nurse, S/N is deputed to FRU-2,<br>only ANM who recently joined , need 3 more ANM	
**4.2**	What difficulties do you face in providing mother and newborn care services to existing employees? (Doctors, nurses and other staff)<br>Occasionally have problem when staff nurse is on leave, all staff members are supportive	
**4.3**	What is the mechanism of taking leave and who sanctions it?<br>Yes, they get training at B.H. hospital	
**5. Duty Roster**		
**5.1**	Who prepares the duty roster for you?<br>Roster is prepared by herself , she maker duly roster for others	
**5.2**	Do you have flexibility in changing the shifts?<br>She can change but with permission from in charge or doctor on call	
**5.3**	How do you manage when you have double shifts?<br>Never needed, other staff nurse are available.	
**5.4**	What is the procedure for taking leave and who approves it?<br>CL's & EL'S are available , take permission from MOIC	
**5.5**	Who prepares rosters for emergency / regular service?<br>She her staff male register for all staff	
**6. Infrastructure**		
**6.1**	Do you have space to accommodate changes inside the department?<br>Yes, changes are possible, have good space	
**6.2**	Do you have enough beds to accommodate increased number of patients?<br>Yes, we have 30 beds, extra space can be made are to need, although never required	
**6.3**	Is their regular power supply and clean water for drinking? Any substitute available in case of power cut or irregular water supply?<br>Power cut is minimum, generally 1-2 hrs, it can by managed by invertors, drinking water only for staff & patient need water cooler	
**7. Data management**		
**7.1**	How do you record data?<br>Have separate register of delivery, admin, daily, newborn, NBCC, NBSUI, Copper T, PPIUCD, MTD, refer, complaint, call register, expense register	
**7.2**	How do you maintain a register?<br>All registers are updated regularly on daily basis.	
**7.3**	Where do you send the record?<br>Send during yearly audit to NHM staff & karyon govt. / delivery & newborn data to B.H. hospital, DHSI -2 is sent directly to CHD every month	
**7.4**	How often is the data sent?<br>Every month , quarterly reports	
**8. Blood bank**		

.

**8.1**	How long does it take for a needy person to get blood?<br>Can arrange in 2-3 hrs in morning hrs. Take time in evening & night so they refer to B.H.	
**9. Training /Skills**		
**9.1**	Could you tell us about your previous NSSK / Neonatal Resuscitation Training?	
	Time (month / year)	2007
	place	Dist Hospital
	Duration (in days)	2 days
**9.2**	Who conducts the workshop? Who prepares roster for workshop/training and how it is notified? How it is monitored?<br>She herself prepare roster for training also inform higher officials about who is left for training	
**9.3**	What did you like the most in the training?<br>Get to learn new things & attain more knowledge	
**9.4**	What did you dislike the most in the training?<br>Food is very oily, quality of rice is not good, and tea is not good, no inverter in case of power cut.	
**9.5**	what was the training methodology used (Lectures/ Hands-on / Practical's)?<br>Modules , practical, hands on session, power point based lectures.	
**9.6**	How did you like meeting / interacting with the trainers?<br>They are good & satisfactory, always get good trainings.	
**9.7**	What was their level of knowledge / skills?	Had moderate knowledge , Dr. anup had high knowledge
	How was your conversation with him	Experience was good, especially with doctor
**9.8**	What are the opportunities and mechanisms currently in place/adopted to retain the skills of Nurses/ANMs/Doctors?<br>Nothing as of now, no training is conducted since more than 1 years. There should be regular Training	
**9.9**	What challenges do you have with the skills of nurses and the support of staff in delivery rooms, perinatal wards and newborn care units? In your opinion, how can this are controlled?<br>Regular training should be done, usually call to CMO office to arrange training of staff.	
**9.10**	How the training related to care during delivery and newborn period can be further improved?<br>Use live newborn & live delivery to training staff instead of conducting training on dummier, trained doctors should conduct session.	
**9.11**	Have you been to a skill lab set up in your district?<br>At B.H. Hospital, At PIO office	
**9.12**	What are the good things about this skill lab?<br>Arrangement was good.	
**9.13**	What are the challenges related to skill lab?<br>No challenges	
**9.14**	In your opinion, how many health staffs might have used or visited the skill labs?<br>Still has training was done from everyone , but never implemented	
**9.15**	Did somebody advise or persuade you to attend the skill lab?<br>From COM office	
**9.16**	How does the Skill Lab help in Neonatal Resuscitation and Neonatal Care?<br>Was beneficial beet it should be done on live newborns.	
**10. Referral services**		
**10.1**	What situation usually the newborns/mothers (pregnant/recently delivered) are referred to the next level of healthcare?<br>Obs labour , prev LS, PPH, not progress , short stretcher units LS/CS, severe anemia<br>Birth asphyxia, severe jaundice, anomalies	

.

**10.2**	Where the newborns/ pregnant women/ mothers are usually referred, what is the usual mode of transportation and how long does it takes to reach the next level health facility in your area?<br>B.H. hospital, call 102 or if patient want then they take personal vehicle.
**10.3**	What facilitation is done from facility side for referral and what difficulties/challenges do you face while transporting the sick newborn and mother to next level? *(Probe: monetary/logistics)*<br>Sometimes ambulance is not available.
**10.4**	What are the challenges faced related to referral transport experienced by this facility and how are they handled?<br>In that case, staff arrange auto & send ward boy or S/N in case of severe case. Patient can arrange private vehicle.
**11. Logistics**	
**11.1**	Are you familiar with any scarcity / irregular supply of medicines and / or supplies required for care during delivery and newborn in the last one year? What were the reasons for this deficiency and how were these conditions managed?<br>No shortage since 1 year
**11.2**	How frequently the families/ parents asked to procure drugs from outside/ store?<br>Sometimes but she never prescribed anyone, especially in morning hours.
**11.3**	What are the supervisory mechanisms in place at present for maternal and newborn care services?<br>Yes, but not regular
	Who supervises<br>CMO, NHM team
	What is the frequency of supervisory visits<br>Not scheduled can be delayed or happen anytime.
	Is any feedback/report provided usually after the supervision?<br>Yes, to NHM, send a copy & ask to submit update
	What actions are taken after last supervisory visit?<br>Yes, action is taken memo is given & ask for updates.
**11.4**	Please let us know about the last supervisory visit to the facility related to maternal and newborn care services?
	Who came for last supervisory visit?<br>DAM in a/april 2017, urban consultant
	How long ago the supervisory visit took place?<br>3 hrs
	What all components were observed?<br>Labour room, ward, tailed , OPD
	What feedback was given and what actions were taken?<br>It was gen visit, took photographs. & send a report for up gradation , actions were taken immediately
**12. others**	
**12.1**	How wide do the events in discharge / record slip?<br>Write everything in detail
**12.2**	In your view, what are the perceived barriers between families in the use of public health services for newborns of pregnant women?<br>Need kitchen for them, no one at gate to guide, no water, relatives sometime comes upstairs to call us as they are not aware that it is residence.<br>How can these obstacles be overcome?<br>Call to duty nurse if someone come upstairs.
**12.3**	How much additional effort is needed to reduce the neonatal mortality rate in your area?<br>24x7 doctors either MO or specialist should be available, ambulance for quick services. Some patients died because ambulance was available.

.

**12.4**	According to you, what is the meaning of quality?
**12.5**	According to you, what are the issues affecting the quality (quality) of health services?
**12.6**	What can you do to improve the quality of the health services?
**12.7**	Did any of your relatives, friends or acquaintances ever availed health services at this hospital? If not, any reason?

.

<table>
<tr><td colspan="4">ID: 1308101</td></tr>
<tr><td colspan="2">1.1 Type of Health Facility: FRU2 , REWARI</td><td colspan="2">1.2 Designation: Medical Officer</td></tr>
<tr><td colspan="4">2. General</td></tr>
<tr><td>2.1</td><td colspan="2">How long have you been working in this health facility? (months/years)</td><td>8 years.</td></tr>
<tr><td>2.2</td><td colspan="2">Total months/years of service</td><td>36 years</td></tr>
<tr><td>2.3</td><td colspan="3">What are your current roles and responsibility with respect to maternal and neonatal care?
I work in emergency at delivery and caesarean, I do run regular OPD (paed). I attend outreach community program, I also do other program like IDCF, IPPI. I work as a NSSK trainer at DI office, I work any form of immunization.</td></tr>
<tr><td rowspan="3">2.4</td><td colspan="3">How many deliveries and resuscitations of newborns have you attended in last 1 month?</td></tr>
<tr><td colspan="2">A. No. of deliveries attended in last 1 month</td><td></td></tr>
<tr><td colspan="2">B. No. of newborn resuscitations attended in last 1 month</td><td>2</td></tr>
<tr><td colspan="4">3. Service Delivery</td></tr>
<tr><td rowspan="8">3.1</td><td colspan="3">In routine practice, which health staff performs the following services?</td></tr>
<tr><td>Services</td><td colspan="2">Staff performing the services</td></tr>
<tr><td>Delivery without complication</td><td colspan="2">NA</td></tr>
<tr><td>Delivery with complication/ high risk delivery</td><td colspan="2">NA</td></tr>
<tr><td>Caesarean section</td><td colspan="2">NA</td></tr>
<tr><td>Newborn care at birth</td><td colspan="2">Only room in facility care at maternity ward- Doctor ward/ Nurse</td></tr>
<tr><td>Sick newborn care</td><td colspan="2">We try to first stabilize the sick baby. If baby is very serious then this condition I refer patient to BK Hospital.</td></tr>
<tr><td>Breastfeeding support</td><td colspan="2">Nurse does it</td></tr>
<tr><td rowspan="7">3.2</td><td colspan="3">What are the challenges faced by you and your colleagues for delivering the desired mother and newborn care services?</td></tr>
<tr><td>Challenges faced</td><td colspan="2">How do you manage these challenges</td></tr>
<tr><td>Infrastructure; there are problem of water Shortage, irregular power cut, and oxygen shortage</td><td colspan="2">I keep bucket of water, Use inverter to support, Use cylinder for oxygen. Otherwise I refer baby.</td></tr>
<tr><td>Equipment; Mostly Working</td><td colspan="2">If equipment doesn’t function, I call Bio-medical engineer, it takes 7 to 15 days.</td></tr>
<tr><td>Drugs and supplies ; only vitamin K injection is not available</td><td colspan="2">Patients purchase from outside,</td></tr>
<tr><td>Support services; All available, no issues.</td><td colspan="2"></td></tr>
<tr><td>Other; There is no logistic support in immunization.</td><td colspan="2">Recall patient.</td></tr>
<tr><td rowspan="5">3.3</td><td colspan="3">What challenges do you face while delivering essential newborn care services and how do you manage these?</td></tr>
<tr><td>Challenges faced</td><td colspan="2">How do you manage these challenges</td></tr>
<tr><td>Care at delivery It is manageable, once baby cry.</td><td colspan="2"></td></tr>
<tr><td>Care in the ward There is no water and no proper light in the ward.</td><td colspan="2"></td></tr>
<tr><td>Care of sick newborns In case of delayed cry, chance of convulsion increases. There is no Laboratory support.</td><td colspan="2">Refer to Bk</td></tr>
<tr><td rowspan="6">3.4</td><td colspan="3">What challenges do you face while delivery of pregnant women?</td></tr>
<tr><td>Challenges faced</td><td colspan="2">How do you manage these challenges</td></tr>
<tr><td>Delivery without complication</td><td colspan="2">NA</td></tr>
<tr><td>Delivery with complication</td><td colspan="2">NA</td></tr>
<tr><td>Caesarean section</td><td colspan="2">NA</td></tr>
<tr><td>Referred cases with complication</td><td colspan="2">NA</td></tr>
</table>

**3.5**	How long usually the mothers stay at the facility after the delivery?	
	Normal Delivery	NA
	Caesarean Delivery	NA

**4. Manpower**	
**4.1**	How many positions of doctors are lying vacant in your health facility?
**4.2**	If there is a shortage of manpower who addresses the issue so that is does not hinders routine work?<br>We convey to CMO, MOIC about all the issues, They address the issues. Problems and issues are discussed in meeting.
**4.3**	Do you have adequate staff inside labor room, ANC clinic and SNCU's?<br>No, there is no dedicated staff inside LR, ANC and NBSU. Maternity Nurses monitor baby at NBSU.
**4.4**	What happens if a particular department is having more flow of patients? Is there any flexibility in assigning inter departmental responsibilities among staff?
**4.5**	What is the mechanism of taking leave and who sanctions it?<br>CMO sir sanction leave, however there are also online system of taking leave.
**5. Duty Roster**	
**5.1**	Who prepares the duty roster for you?<br>There is no duty roster available, we are available at round the clock.
**5.2**	Who follows up the prepared roster so that the shifts are routinely changed?<br>NA
**5.3**	How many Medical Officers are posted at one time in your department? What is the pattern of shift?<br>There No medical officer here.
**5.4**	Do you have flexibility in changing the shifts?<br>No, we don't do our duty shift wise. We work round the clock. In case of any emergency I come on call. My residence is at hospital premises.
**5.5**	How do you manage when you have double shifts?<br>NA.
**6. Infrastructure**	
**6.1**	Do you have space to accommodate changes inside the department?<br>Yes,
**6.2**	Do you have enough beds to accommodate increased number of patients?<br>Yes beds are enough but we don't need to increase number of beds,
**6.3**	Is their regular power supply and clean water for drinking? Any substitute available in case of power cut or irregular water supply?<br>Inverters are installed but it run for short period, a water cooler is
**7. Training /Skills**	
**7.1**	How many of the total staff are trained for MCH services?<br>They all are trained.
**7.2**	Is there any pre job posting training for newly joined staff?<br>No. Induction program is facilitated at BK.
**7.3**	Is there any on- job training for the staff?<br>Yes.
**7.4**	Please let us know about the last training on attended by you?<br>I had attended NRC training.

	Timing (Month/Year)	2015
	Place	Kalawati Hospital, Delhi
	Duration (in days)	3 days
	What did you like the most in the training?<br>Demo was good, I had learned new things. I have learned formulae for feeding the malnourished children.	
	What did you dislike the most in the training?	

	Everything was fine. I enjoyed
	What was the training methodology used (Lectures/ Hands-on / Practical's)?<br>Lectures, live demo Group discussion Pre and post test.
	Who conducts the workshop? Who prepares roster for workshop/training and how it is notified? How it is monitored?<br>State team has conducted this training. They notify via email to districts.
**7.5**	What are the opportunities and mechanisms currently in place/adopted to retain the skills of Nurses/ANMs/Doctors?<br>Group discussion among staff and day to day meeting can be arranged.
**7.6**	How the training related to care during delivery and newborn period can be further improved?
**7.7**	Have you ever visited/attended the skill labs operational in your district? Where it was conducted? Who conducted it? What was the time duration of skill lab training?<br>I never attended the skill labs training.
**7.8**	What are the good things about this skill lab?<br>NA
**7.9**	What are the challenges related to skill lab?<br>NA
**7.10**	In your opinion, how many health staffs might have used or visited the skill labs?<br>NA
**7.11**	Did somebody advise or persuade you to attend the skill lab?<br>NA
**8. Referral services**	
**8.1**	In what situation usually the newborns/mothers (pregnant/recently delivered) are referred to the next level of healthcare?<br>Severe birth asphyxia, convulsion, congenital malformation.
**8.2**	Where the newborns/ pregnant women/ mothers are usually referred, what is the usual mode of transportation and how long does it takes to reach the next level health facility in your area?<br>Newborns/ pregnant women are usually referred to BK Hospital, They reach hospital by ambulance.
**8.3**	What facilitation is done from facility side for referral and what difficulties/challenges do you face while transporting the sick newborn and mother to next level? *(Probe: monetary/logistics)*<br>There is no issue, EMT takes patient to hospital.
**8.4**	What are the challenges faced related to referral transport experienced by this facility and how are they handled?<br>Sometimes ambulance is not available at time also it takes time to reach hospital.
**9. Logistics**	
**9.1**	Are you aware of any shortage/irregular supply of drugs and/or supplies needed for care during delivery and newborn period in the last one year? What were the reasons for this shortage and how these situations were managed?<br>Only Vit K is not available. There is no need of vit. K for every child, if required then patients purchase it from outside. MOIC knows the standard protocol.
**9.2**	How frequently the families/ parents asked to procure drugs from outside/ store?<br>It happens very rarely.
**9.3**	How many equipments essential for management of delivery or newborn care are out of order at this moment?<br>All essential equipments for management of delivery or newborn care are there at hospital.
**9.4**	What is the usual mechanism of repair and maintenance of these equipments? *(probe: who is responsible and what is the duration of repair*)<br>I call bio medical engineer from BK. It takes minimum 10 days to repair.
**9.5**	What are the supervisory mechanisms in place at present for maternal and newborn care services?
	Who supervises<br>State head quarter, central team and NHM

	What is the frequency of supervisory visits<br>Almost in six months.
	Is any feedback/report provided usually after the supervision?<br>Yes, Report is prepared.
	What actions are taken after last supervisory visit?<br>They take actions if found any issues,
**9.6**	Please let us know about the last supervisory visit to the facility related to maternal and newborn care services?
	Who came for last supervisory visit?<br>Central govt team from mohfw
	How long ago the supervisory visit took place?<br>It has been 2 months.
	What all components were observed?<br>Maternity ward, child health service, OPD
	What feedback was given and what actions were taken?
**10. Perceptions regarding Quality of care**	
**10.1**	According to you, what is the meaning of quality?<br>Health services with complete satisfaction of patients.
**10.2**	According to you, what are the issues that affect the quality of health services?<br>Infrastructure issues, shortage of staffs, no guard, irregular power supply and water problem.
**10.3**	What can you do to improve the quality of the health services?<br>Discuss the issues and challenges, and resolve it timely.
**10.4**	Did any of your relatives, friends or acquaintances ever availed health services at this hospital? If not, any reason?<br>No, There is no proper facility t hospital, who will take risk for their own relatives.
**11. Others**	
**11.1**	If any shortage of blood and how is it tackled?<br>There is no blood bank here; Patients get it from BK hospital. we refer them
**11.2**	Do you arrange blood donation camps on facility basis?<br>No.

ID: 1308201			
**1.1 Type of Health Facility FRU 2 , FBD**		**1.2 Designation: STAFF NURSE**	
**2. General**			
**2.1**	How long have you been working in this health facility? (months/years)<br>I am working in this hospital since December 2012 under NHM.		
**2.2**	Total months/years of service<br>My total work experience is 7 years. I am working since august 2010.		
**2.3**	What are your current roles and responsibility with respect to maternal and neonatal care?<br>I do lot of work here. I conduct normal deliveries and provide post natal care to the patient. I also manage ANC patients by putting drip for glucose or iron sucrose. I do vital checkups of the patients and provide emergency services as well. Apart from that, I maintain all the data on register.		
**2.4**	How many deliveries and resuscitations of newborns have you attended in last 1 month?		
	A. No. of deliveries attended in last 1 month		7-8 deliveries
	B. No. of newborn resuscitations attended in last 1 month		None
**2.5**	Who did you receive the training from?		
	Area	Training name	year
	A Care during delivery (S.B.A.)	SBA	2011
	B Neonatal Resuscitation Program (In RP / NSS)	NSSK	2016
	C Sick Newborn Care (FBNC)	No such training done till now	
**3. Service Delivery**			
**3.1**	What are the challenges faced by you and your colleagues for delivering the desired mother and newborn care services?		
	**Challenges faced**		
		**Mother care**	**Newborn care**
	Infrastructure	Power supply is not regular. Sometime we have to use mobile or torch to conduct deliveries. There is shortage of drinking water as well. We get it from our home. Some patients who come from far have to face problems.	Same issues
	Equipment	Currently there is shortage of gloves. Rest all equipments and material are available. Never had any big issues.	No issues
	Drugs and supplies	Medicines are always available. No issues in relation to drugs and supply	No emergency medicines are available in night
	Support services	There is no security guard in the hospital. It hampers security issues as some patient misbehave with us. If not full time, then atleast there should be 1 guard in night.	No issues
	Other	No issues	No issues
**3.2**	What challenges do you face while delivering essential newborn care services and how do you manage these?		
	**Challenges faced**	**How do you manage these challenges**	
	Care at delivery: No issues. We do normal deliveries every day. So we are in practice.		
	Care in the ward: No problem in doing care at ward.		
	Care of sick newborns: No, we do not have to do anything.		

	Dr. is always available.	
3.3	What challenges do you face while delivery of pregnant women?	
	**Challenges faced**	**How do you manage these challenges**
	Delivery without complication	No issues
	Delivery with complication	Doctors are always present to attend high risk deliveries or we do in their supervision.
	Caesarean section	Doctors do caesarean. We only assist them in surgery. Earlier there were 2 doctors in hospital. It was more convenient then.
	Referred cases with complication	Biggest issue is that patients do not get convince easily. They shout and sometimes fight as well.
3.4	How long usually the mothers stay at the facility after the delivery?	
	Normal Delivery	48 hours
	Caesarean Delivery	Min 3 days
**4. Manpower**		
4.1	How many posts of Staff Nurse / ANM are vacant in your health facility?<br>At present, all post are complete. Shortage is felt when we have busy schedule. But still we can manage. We are 4 staff nurse and 1 staff nurse is deputed from fru 1.	
4.2	What difficulties do you face in providing mother and newborn care services to existing employees? (Doctors, nurses and other staff)<br>There are no issues. Everyone listen to each other and work with mutual understanding.	
4.3	What is the mechanism of taking leave and who sanctions it?<br>New staff get training at BK hospital. All staff have to attend training sessions before joining.	
**5. Duty Roster**		
5.1	Who prepares the duty roster for you?<br>Our nursing sister prepares the duty roster for us.	
5.2	Do you have flexibility in changing the shifts?<br>Yes, changes are possible according to our convenience.	
5.3	How do you manage when you have double shifts?<br>It was never required till now. But we do work for some extra hours if other staff is not present. It depends upon our mutual understanding.	
5.4	What is the procedure for taking leave and who approves it?<br>We have casual leaves. We apply to nursing sister and it is approved by MOIC.	
5.5	Who prepares rosters for emergency / regular service?<br>Nursing sister prepares the roster for emergency duty. It only happens when other staff is on leave.	
**6. Infrastructure**		
6.1	Do you have space to accommodate changes inside the department?<br>I don't think so. Already beds are sufficient. No changes are possible.	
6.2	Do you have enough beds to accommodate increased number of patients?<br>In case of more number of patients, we shift them to NRC ward or discharge them early if no extra care is required. Occasionally, 2 patients share 1 bed as well.	
6.3	Is their regular power supply and clean water for drinking? Any substitute available in case of power cut or irregular water supply?<br>There are big issues of water and electricity. Clean drinking water is not available in hospital neither there is regular power supply. Situation is worse in summers. Back up source is not sufficient as it runs for few hours. There should be a generator for power back up. PNC ward is not even connected with invertor.	
**7. Data management**		
7.1	How do you record data?<br>We maintain registers for everything. All entries are done regularly and updated.	

.

**7.2**	How do you maintain a register?<br>We regularly update our registers. Entries are done every day.	
**7.3**	Where do you send the record?<br>We send all the data to computer room every day. They send it to CMO office on monthly basis.	
**7.4**	How often is the data sent?<br>We send all the records on daily basis to computer room. Earlier, we used to send birth form in 10 days but they are not given as of now because of no stationary.	
**8. Blood bank**		
**8.1**	How long does it take for a needy person to get blood?<br>We call at blood bank in BK hospital and it can be arranged within 1 hour. Occasionally it takes time in case of shortage of blood. In this situation patient have to arrange by himself or go to private blood banks. Emergency cases are referred to BK hospital.	
**9. Training /Skills**		
**9.1**	Could you tell us about your previous NSSK / Neonatal Resuscitation Training?	
	Time (month / year)	April, 2016
	place	BK hospital
	Duration (in days)	3 days
**9.2**	Who conducts the workshop? Who prepares roster for workshop/training and how it is notified? How it is monitored?<br>We get call from BK hospital regarding trainings. Nursing sister or clerk sends us the notification.	
**9.3**	What did you like the most in the training?<br>We get to learn new things. Another good point is that we get extra payment to attend training.	
**9.4**	What did you dislike the most in the training?<br>No one actually likes the session. We just go for formality. Now we do not get payment or TA/DA so it is worse now.	
**9.5**	What was the training methodology used (Lectures/ Hands-on / Practical's)?<br>We do practical on dummies. There are lecture sessions with PowerPoint slides as well.	
**9.6**	How did you like meeting / interacting with the trainers?<br>Dr. is the best trainer. We had a good learning experience from them. Rest of the trainers are boring.	
**9.7**	What was their level of knowledge / skills?	They had good knowledge about the subject
	How was your conversation with him	I was afraid initially but now we have good interaction with them.
**9.8**	What are the opportunities and mechanisms currently in place/adopted to retain the skills of Nurses/ANMs/Doctors?<br>Only training is the source to improve our capability.	
**9.9**	What challenges do you have with the skills of nurses and the support of staff in delivery rooms, perinatal wards and newborn care units? In your opinion, how can this be controlled?	
**9.10**	How the training related to care during delivery and newborn period can be further improved?<br>Trainings are fine. There is no need to improve them. Only thing which can be added is that we should get payment and TA/DA for training. Food menu should also be pre decided.	
**9.11**	Have you been to a skill lab set up in your district?<br>Yes, I went to skill lab for training. The skill lab is situated at BK hospital.	
**9.12**	What are the good things about this skill lab?<br>Best part was that everything was taught practically. We got practical experience of Hb.	
**9.13**	What are the challenges related to skill lab?<br>No, there are no issues. Everything is good.	
**9.14**	In your opinion, how many health staffs might have used or visited the skill labs?<br>Most of the staff is done with skill lab training. All staff nurse from our hospital have gone to skill lab.	
**9.15**	Did somebody advise or persuade you to attend the skill lab?<br>We got a call from BK hospital to attend the training. They only generate the list from DI office and	

	inform the staff about it.
**9.16**	How does the Skill Lab help in Neonatal Resuscitation and Neonatal Care?<br>Yes, it was helpful. We got to know many new things. Now we know about the criteria to resuscitate a newborn if required.
**10. Referral services**	
**10.1**	In what situation usually the newborns/mothers (pregnant/recently delivered) are referred to the next level of healthcare?<br>We refer patients with severe PPH, eclampsia, low BP, anemia to higher facility.<br>Newborn with low birth weight, meconium strain and if the baby is not breastfeeding then we refer them to BK hospital.
**10.2**	Where the newborns/ pregnant women/ mothers are usually referred, what is the usual mode of transportation and how long does it takes to reach the next level health facility in your area?<br>We refer the patients to BK hospital. Patient can go to private hospital by their choice. Ambulance is always available. It takes about 15-30 minutes to reach the hospital. it depends upon traffic.
**10.3**	रे What facilitation is done from facility side for referral and what difficulties/challenges do you face while transporting the sick newborn and mother to next level? *(Probe: monetary/logistics)*<br>We provide them wheel chair and 1 staff nurse accompany the patient. Nowadays, no staff nurse is going because the ambulance driver denies to drop back the staff to hospital if they get another call from there. Sometimes sweeper or class IV go with the patient.
**10.4**	What are the challenges faced related to referral transport experienced by this facility and how are they handled?<br>No issues as of now. Driver may get call for other patient and so they leave. Nowadays it happened frequently otherwise everything was going smoothly.
**11. Logistics**	
**11.1**	Are you familiar with any scarcity / irregular supply of medicines and / or supplies required for care during delivery and newborn in the last one year? What were the reasons for this deficiency and how were these conditions managed?<br>There is shortage of medicines but very occasionally. If any medicine is not available then we do local purchase and complete the stock.
**11.2**	How frequently the families/ parents asked to procure drugs from outside/ store?<br>Only gloves are required as we are not getting it from warehouse and now it is out of stock. Patient bring it for us from local market. But that too occasionally.
**11.3**	What are the supervisory mechanisms in place at present for maternal and newborn care services?
	Who supervises<br>Dr. or CMO come for supervision visits.
	What is the frequency of supervisory visits<br>It happens in every 2-3 months.
	Is any feedback/report provided usually after the supervision?<br>They write in a diary and inform about any gaps to nursing sister.
	What actions are taken after last supervisory visit?<br>We were informed about shortcomings and gaps. We immediately did the action as it was recommended and informed to CMO.
**11.4**	Please let us know about the last supervisory visit to the facility related to maternal and newborn care services?
	Who came for last supervisory visit?<br>Last visit was done by DAM. I do not know his name.
	How long ago the supervisory visit took place?<br>The visit lasted for 3-4 hours.

	What all components were observed?<br>Supervision of labor room, OPD and ward was done.
	What feedback was given and what actions were taken?<br>I am not sure as I was not on that shift.
**12. Others**	
**12.1**	How wide do the events in discharge / record slip?<br>We write everything on the discharge slip. Whatever treatment we did on patient, we have to write on the discharge slip.
**12.2**	In your view, what are the perceived barriers between families in the use of public health services for newborns of pregnant women?<br>Family members fight and do not listen to us when we advise them to refer.<br>How can these obstacles be overcome?<br>At last we leave on choice of patient and family members as there is no other solution.
**12.3**	How much additional effort is needed to reduce the neonatal mortality rate in your area?<br>Complicated and high risk deliveries should be referred in advance. But patient do not listen. ANC checkups should be mandatory and there should be some written guidelines about personal care and care of baby at home after birth.
**12.4**	How much additional effort is needed to reduce the neonatal mortality rate in your area?
**12.5**	According to you, what are the issues affecting the quality (quality) of health services?
**12.6**	What can you do to improve the quality of the health services?
**12.7**	Did any of your relatives, friends or acquaintances ever availed health services at this hospital? If not, any reason?

.

ID: 1308302	
AREA DH, fbd	DESIGNATION : class IV
**S.No.**	**Questions**
**1.**	**General Information**
1.1	Are you an employee of the hospital or are you contracted for the same?<br>I work in this hospital on contractual basis.
1.2	How many class 4 are there in this hospital?<br>There are 14 to 15 class IV working in this hospital.
**2.**	**Specific Information**
2.1	Please tell us about the duty of duty and how many people are present in the cross<br>One staff is assigned for morning shift, Two classIV and one sweeper work in the evening and at night shift, two classIV and sweeper.
2.2	Your responsibility is only of one department, of all the other departments of the hospital?<br>Mostly my duty is at labour room and maternity ward, but sometimes it changes, and I am assigned in medical ward, OPD and surgical ward as well.
2.3	explain your responsibilities<br>I do sterilization of instruments, dusting, and bedding. I also assist staff nurses to prepare glucose solution and also assist in delivery process.
2.4	Do you have a duty even during the night shift?<br>Yes,
2.5	Tell us which places in this hospital do you take care of cleanliness?<br>I clean all the ward, LR, and OPD.
2.6	Tell us about your family's success?<br>We do it duty wise. I clean LR at night and rest area in the morning time.
2.7	What media do you use for cleaning many types of goods?<br>I use Harpic and for sink and toilet. For mopping floor We use surf, liquid and phenyl. For dusting we use dry piece of cloth.
2.8	Does anyone check your work?<br>Yes, my work is checked by nursing sister. If she is busy in her work then next morning she checks my work.
**3.**	**Disposal**
3.1	How you are you dispose the waste?<br>I collect the filths form LR and wards and put it transfer to Waste disposal van.
3.2	Do you use color coding systems for waste disposal? Detailed<br>Yes, I segregate waste and discard it to color coded plastic bags. I use black plastic bags for general waste, red plastic bags for plastic items, and use blue bags for glass made materials and yellow bags for blood, and wet waste.
3.3	There is any vehicle for pickup the waste?<br>Yes, Daily waste disposal van comes to take waste.
3.4	If yes, then how many times is it come in the hospital??<br>Mostly daily if miss someday then we arrange private vehicles.
3.5	Do you burn the waste room? If so where<br>No, a strict order by MOIC.
**4.**	**Issues/Suggestions**
4.1	tell us how other staff members treat you in hospital<br>Very Good it is. Everyone in this hospital is behaving well.
4.2	tell us how patients treat you in the hospital

	My behavior is good with patients, sometimes I have to deal strictly with them. I try to make them satisfy with my work.
4.3	Want to change the way you do for better service?<br>I don't have much problem to work here, sometimes small problem arises.
4.4	Want to change the way you do for better service?<br>I have not got my salary for two years. Nobody cares about us. I don't have any option but working here. If our pay will increase the quality of work can be better.
**4.5**	What do you mean by quality?<br>Government money should use correctly and right way, so that patients get all the benefits.
**4.6**	According to you, what are the issues affecting the quality of health services?<br>Patients do not have their treatment properly. Patients always ask for discharge before complete treatment. If Dr. refers the patients they denied going higher facility.
**4.7**	What can you do to improve the quality of health services?<br>I am always ready to do work, but for quality of work, government should increase my payment.
**4.8**	Have your relatives, friends or acquaintances ever taken advantage of the health services of this hospital? If not, any reason?<br>Yes, they come, but most of the time they take service from nearby because this facility is far away.

ID: 1308302	
**AREA DH, fbd**	**DESIGNATION : class IV**
**S.No.**	**Questions**
**1.**	**General Information**
1.1	Are you an employee of the hospital or are you contracted for the same?<br>I work in this hospital on contractual basis.
1.2	How many class 4 are there in this hospital?<br>There are 14 to 15 class IV working in this hospital.
**2.**	**Specific Information**
2.1	Please tell us about the duty of duty and how many people are present in the cross<br>One staff is assigned for morning shift, Two classIV and one sweeper work in the evening and at night shift, two classIV and sweeper.
2.2	Your responsibility is only of one department, of all the other departments of the hospital?<br>Mostly my duty is at labour room and maternity ward, but sometimes it changes, and I am assigned in medical ward, OPD and surgical ward as well.
2.3	explain your responsibilities<br>I do sterilization of instruments, dusting, and bedding. I also assist staff nurses to prepare glucose solution and also assist in delivery process.
2.4	Do you have a duty even during the night shift?<br>Yes,
2.5	Tell us which places in this hospital do you take care of cleanliness?<br>I clean all the ward, LR, and OPD.
2.6	Tell us about your family's success?<br>We do it duty wise. I clean LR at night and rest area in the morning time.
2.7	What media do you use for cleaning many types of goods?<br>I use Harpic and for sink and toilet. For mopping floor We use surf, liquid and phenyl. For dusting we use dry piece of cloth.
2.8	Does anyone check your work?<br>Yes, my work is checked by nursing sister. If she is busy in her work then next morning she checks my work.
**3.**	**Disposal**
3.1	How you are you dispose the waste?<br>I collect the filths form LR and wards and put it transfer to Waste disposal van.
3.2	Do you use color coding systems for waste disposal? Detailed<br>Yes, I segregate waste and discard it to color coded plastic bags. I use black plastic bags for general waste, red plastic bags for plastic items, and use blue bags for glass made materials and yellow bags for blood, and wet waste.
3.3	There is any vehicle for pickup the waste?<br>Yes, Daily waste disposal van comes to take waste.
3.4	If yes, then how many times is it come in the hospital??<br>Mostly daily if miss someday then we arrange private vehicles.
3.5	Do you burn the waste room? If so where<br>No, a strict order by MOIC.
**4.**	**Issues/Suggestions**
4.1	tell us how other staff members treat you in hospital<br>Very Good it is. Everyone in this hospital is behaving well.
4.2	tell us how patients treat you in the hospital

	My behavior is good with patients, sometimes I have to deal strictly with them. I try to make them satisfy with my work.
4.3	Want to change the way you do for better service?<br>I don't have much problem to work here, sometimes small problem arises.
4.4	Want to change the way you do for better service?<br>I have not got my salary for two years. Nobody cares about us. I don't have any option but working here. If our pay will increase the quality of work can be better.
**4.5**	What do you mean by quality?<br>Government money should use correctly and right way, so that patients get all the benefits.
**4.6**	According to you, what are the issues affecting the quality of health services?<br>Patients do not have their treatment properly. Patients always ask for discharge before complete treatment. If Dr. refers the patients they denied going higher facility.
**4.7**	What can you do to improve the quality of health services?<br>I am always ready to do work, but for quality of work, government should increase my payment.
**4.8**	Have your relatives, friends or acquaintances ever taken advantage of the health services of this hospital? If not, any reason?<br>Yes, they come, but most of the time they take service from nearby because this facility is far away.

ID: 2108101	
**1 Type of Health Facility: DH, REWARI**	**1.2 Designation: STAFF NURSE**

2. General

**2.1**	How long have you been working in this health facility? (months/years)- Since December 2011
**2.2**	Total months/years of service - 6 years
**2.3**	What are your current roles and responsibility with respect to maternal and neonatal care?<br>Child should feed properly, teaching mother about the hygienic system, Doubling the treatment of LBW, And when discharged it should be done properly.
**2.4**	How many deliveries and resuscitations of newborns have you attended in last 1 month?

A. No. of deliveries attended in last 1 month	NA
B. No. of newborn resuscitations attended in last 1 month	No. As I am posted here in the Ward and resuscitation cases are done in SNCU only. So I could not do

2.5 Who did you receive the training from:

	Area	Training name	Year
A	Care during delivery (S.B.A.)		
B	Neonatal Resuscitation Program (In RP / NSS)	NSSK	2014
C	Sick Newborn Care (FBNC)	FBNC	2012

3. Service Delivery

3.1 What are the challenges faced by you and your colleagues for delivering the desired mother and newborn care services?

Challenges faced

	**Mother care**	**Newborn care**
Infrastructure		
Equipment		Phototherapy machine is not working and after saying so many times new machines are not installed yet.
Drugs and supplies		Medicines are kept in spare for 1 month and prescriptions are done accordingly. It is very rare that medicine is unavailable.
Support services		In the night 4th class staff is no available so if there is any problem in the night we ourselves have to manage even if attendant of the patient Is fighting with us also.
Other	One more problem for the mother is that they gets discharged from the gynae ward and they all come and sit inside the SNCU as after discharge from gynae they don't have any other place to go as their babies are still admitted here	Here we have only single surface phototherapy machine and most of newborn admitted here are coming with jaundice level of 20. Due to the unavailability of the machines we have to admit on one phototherapy machine 3 newborns and which is not affecting properly the treatment

3.2 What challenges do you face while delivering essential newborn care services and how do you manage these?

**Challenges faced**	**How do you manage these challenges**
Care at delivery	
Care in the ward	
Care of sick newborns	

<table>
<tr><td>3.3</td><td colspan="2">What challenges do you face while delivery of pregnant women?</td></tr>
<tr><td></td><td>Challenges faced</td><td>How do you manage these challenges</td></tr>
<tr><td></td><td>Delivery without complication</td><td></td></tr>
<tr><td></td><td>Delivery with complication</td><td></td></tr>
<tr><td></td><td>Caesarean section</td><td></td></tr>
<tr><td></td><td>Referred cases with complication</td><td></td></tr>
<tr><td>3.4</td><td colspan="2">How long usually the mothers stay at the facility after the delivery?</td></tr>
<tr><td></td><td>Normal Delivery</td><td></td></tr>
<tr><td></td><td>Caesarean Delivery</td><td></td></tr>
<tr><td colspan="3">4. Manpower</td></tr>
<tr><td>4.1</td><td colspan="2">How many posts of Staff Nurse / ANM are vacant in your health facility?
Earlier there were 10 staff in SNCU but now as 8 staff are available maybe 2 post are vacant. It is like this since 2-3 years but now I heard 1 staff is hired but don’t now in which department</td></tr>
<tr><td>4.2</td><td colspan="2">What difficulties do you face in providing mother and newborn care services to existing employees? (Doctors, nurses and other staff)
If staff duty is mentioned on the duty then that particular staff is available.</td></tr>
<tr><td>4.3</td><td colspan="2">What is the mechanism of taking leave and who sanctions it?
Yes</td></tr>
<tr><td colspan="3">5. Duty Roster</td></tr>
<tr><td>5.1</td><td colspan="2">Who prepares the duty roster for you?
Nursing sister and Dr. both make the roster together</td></tr>
<tr><td>5.2</td><td colspan="2">Do you have flexibility in changing the shifts?
Yes we inform to the nursing sister and ask the consent of Staff whether they are ready for this change, if she is ready then adjustment can be done.</td></tr>
<tr><td>5.3</td><td colspan="2">How do you manage when you have double shifts?
There are too many of night shifts due to which it comes frequently as in both the areas 4-4 staff are available, so in a month around 10 night shifts we are doing. If other shift staff is unavailable due to any emergency reasons we had to come for evening shift just after completing night shifts on the same day.</td></tr>
<tr><td>5.4</td><td colspan="2">What is the procedure for taking leave and who approves it?
That we have to inform nursing sister we get leaves if needed.</td></tr>
<tr><td>5.5</td><td colspan="2">Who prepares rosters for emergency / regular service?
Nursing sister</td></tr>
<tr><td colspan="3">6. Infrastructure</td></tr>
<tr><td>6.1</td><td colspan="2">Do you have space to accommodate changes inside the department?
No there is no space as you can see limited space is here,, no extra beds can be kept here.</td></tr>
<tr><td>6.2</td><td colspan="2">Do you have enough beds to accommodate increased number of patients?
Yes in case of high patient load we have to do doubling or tripling in the beds. Tripling only done on the bigger beds.</td></tr>
<tr><td>6.3</td><td colspan="2">Is their regular power supply and clean water for drinking? Any substitute available in case of power cut or irregular water supply?
There is no problem for that. If light is not available then automatically inverter starts.</td></tr>
<tr><td colspan="3">7. Data management</td></tr>
<tr><td>7.1</td><td colspan="2">How do you record data?
Here we have admission, discharge, refer, and bed sheet, medicine stock registers where we do daily entries.</td></tr>
<tr><td>7.2</td><td colspan="2">How do you maintain a register?</td></tr>
<tr><td>7.3</td><td colspan="2">Where do you send the record?
Previous month reports are taken us by sister and I don’t know where is she giving or what she is doing with that. Dr. also takes register from us.</td></tr>
</table>

.

**7.4**	How often is the data sent?<br>Complete month report is given	
**8. Blood bank**		
**8.1**	Could you tell us about your previous NSSK / Neonatal Resuscitation Training?<br>If any person is coming here on correct time then we use to transfuse blood within 1 hour.	
**9. Training process**		
**9.1**	Could you tell us about your previous NSSK / Neonatal Resuscitation Training?	
	Time (month / year)	2012
	place	Chacha Nehru
	Duration (in days)	15
**9.2**	Who conducts the workshop? Who prepares roster for workshop/training and how it is notified? How it is monitored?<br>That is done by Staff, he use to tell to us that when and where we have to go for training.	
**9.3**	What did you like the most in the training?	
**9.4**	What did you dislike the most in the training?<br>Mine was very bad, we didn't got any residence to stay, Dr. was very strict, we all got troubled full 15 days. We didn't have any proper facility maybe that was the first batch of training that's why so many problems we faced.	
**9.5**	What was the training methodology used (Lectures/ Hands-on / Practical's)?<br>Projector was used	
**9.6**	How did you like meeting / interacting with the trainers?<br>IT was ok Dr.was the trainer, the training was mostly of observation nothing much.	
**9.7**	What was their level of knowledge / skills?	
	How was your conversation with him	
**9.8**	What are the opportunities and mechanisms currently in place/adopted to retain the skills of Nurses/ANMs/Doctors?<br>We people should get trainings as we don't have any knowledge whatever we do here we know that much only nothing extra.	
**9.9**	What challenges do you have with the skills of nurses and the support of staff in delivery rooms, perinatal wards and newborn care units? In your opinion, how can this be controlled?	
**9.10**	How the training related to care during delivery and newborn period can be further improved?	
**9.11**	Have you been to a skill lab set up in your district?	
**9.12**	What are the good things about this skill lab?	
**9.13**	What are the challenges related to skill lab?	
**9.14**	In your opinion, how many health staffs might have used or visited the skill labs?	
**9.15**	Did somebody advise or persuade you to attend the skill lab?	
**9.16**	How does the Skill Lab help in Neonatal Resuscitation and Neonatal Care?	
**10. Referral services**		
**10.1**	In what situation usually the newborns/mothers (pregnant/recently delivered) are referred to the next level of healthcare?<br>We refer in the case that jaundice is more than 20 baby is lethargic, LBW like such cases we refer and respiratory problem is there since long time or coming here from private hospital without crying. We try here first for improvement but If the child is on seizure then we only tell them to take forwards. If the condition is not in our control then we refer	
**10.2**	Where the newborns/ pregnant women/ mothers are usually referred, what is the usual mode of transportation and how long does it takes to reach the next level health facility in your area?<br>PGI. Rohtak	
**10.3**	What facilitation is done from facility side for referral and what difficulties/challenges do you face while transporting the sick newborn and mother to next level? *(Probe: monetary/logistics)*<br>1 hour it takes to reach there. Earlier it use to take longer time but now the condition of the road is	

.

	good so we refer there itself.
**10.4**	What are the challenges faced related to referral transport experienced by this facility and how are they handled?<br>This I don't know as my duty is mostly here in ward and such problems I have never seen.
**11. Logistics**	
**11.1**	Are you familiar with any scarcity / irregular supply of medicines and / or supplies required for care during delivery and newborn in the last one year? What were the reasons for this deficiency and how were these conditions managed?
**11.2**	How frequently the families/ parents asked to procure drugs from outside/ store?<br>Actually every medicines are available here itself so we don't prescribe any medicines to buy from outside.
**11.3**	What are the supervisory mechanisms in place at present for maternal and newborn care services?
	Who supervises
	What is the frequency of supervisory visits
	Is any feedback/report provided usually after the supervision?
	What actions are taken after last supervisory visit?
**11.4**	Please let us know about the last supervisory visit to the facility related to maternal and newborn care services?
	Who came for last supervisory visit?<br>That is told by Dr only that the condition of the baby is good for discharge or not.
	How long ago the supervisory visit took place?
	What all components were observed?<br>First he sees all the condition that whether it is improved or not. Other than that he used to check the respiration, feeding, weight etc.
	What feedback was given and what actions were taken?
**12. others**	
**12.1**	How wide do the events in discharge / record slip?<br>In that sir use to write the weight, heart rate , medicine , follow up
**12.2**	In your view, what are the perceived barriers between families in the use of public health services for newborns of pregnant women?<br>How can these obstacles be overcome?
**12.3**	How much additional effort is needed to reduce the neonatal mortality rate in your area?
**12.4**	According to you, what is the meaning of quality?<br>Good work is quality or if the work is beneficial for others then that is quality
**12.5**	According to you, what are the issues affecting the quality (quality) of health services?<br>Here the number of beds is very less due to which we have to face lots of problem due to adjusting which leads to arguments with the attendant.<br>The micropore available here is very bad as it doesn't stick properly on the cannula.
**12.6**	What can you do to improve the quality of the health services?<br>Gown should be available for the mother. As the KMC room is very far from here some staff should take child for KMC from here to the room. The mother here is too unhygienic due to which we also sometimes hesitate to give the child to her but we can't do anything. Now they all came inside SNCU which creates bad smell inside the SNCU which should be controlled. 1 class 4 staff is very much needed in the evening shifts.
**12.7**	Did any of your relatives, friends or acquaintances ever availed health services at this hospital? If not, any reason?<br>Yes they use to come

.

ID: 2108101		
**1.1 Type of Health Facility: DH ,REWARI,**	**1.2 Designation: Medical officer**	
**2. General**		
**2.1**	How long have you been working in this health facility? (months/years)	April 2017
**2.2**	Total months/years of service	It's been 3 months
**2.3**	What are your current roles and responsibility with respect to maternal and neonatal care?<br>Mine is OPD of paeds, then SNCU duty and we use to see children upto the age of 0- 18	
**2.4**	How many deliveries and resuscitations of newborns have you attended in last 1 month?	
	A. No. of deliveries attended in last 1 month	
	B. No. of newborn resuscitations attended in last 1 month<br>I don't know the counting of it but yes we do many	
**3. Service Delivery**		
**3.1**	In routine practice, which health staff performs the following services?	
	**Services**	**Staff performing the services**
	Delivery without complication	Nurse
	Delivery with complication/ high risk delivery	Doctor & nurse
	Caesarean section	
	Newborn care at birth	Nurse & Doctor
	Sick newborn care	Nurse & Doctor
	Breastfeeding support	Nurse
**3.2**	What are the challenges faced by you and your colleagues for delivering the desired mother and newborn care services?	
	**Challenges faced**	**How do you manage these challenges**
	Infrastructure	The structure here is good but the problem here is that Both SNCU & OPD are at different places
	Equipment The main equipments such as CPAP & ventilator is not available here	We refer such cases
	Drugs and supplies No challenges	
	Support services Unavailability of guard & class 4 for evening & night shifts	It is managed by the staff nurse & available Dr. only during evening & night shifts
	Other	
**3.3**	What challenges do you face while delivering essential newborn care services and how do you manage these?	
	**Challenges faced**	**How do you manage these challenges**
	Care at delivery<br>No challenges	
	Care in the ward<br>No challenges	
	Care of sick newborns Sometimes when there is rush then it feels like there should be 2 staff nurses appointed per shifts	What manage we are doing the way we can
**3.4**	What challenges do you face while delivery of pregnant women?	
	**Challenges faced**	**How do you manage these challenges**
	Delivery without complication	NA
	Delivery with complication	NA
	Caesarean section	NA
	Referred cases with complication	NA
**3.5**	How long usually the mothers stay at the facility after the delivery?	
	Normal Delivery	NA
	Caesarean Delivery	NA
**4. Manpower**		

**4.1**	How many positions of doctors are lying vacant in your health facility?<br>I can tell you about SNCU only that presently there is 1 vacant post for Dr as recently 1 MO has left	
**4.2**	If there is a shortage of manpower who addresses the issue so that is does not hinders routine work?<br>We all adjust amongst each other	
**4.3**	Do you have adequate staff inside labor room, ANC clinic and SNCU's?<br>There is limited staff in the SNCU like Class 4, Guard and Sweeper are not available in the evening & night shifts	
**4.4**	What happens if a particular department is having more flow of patients? Is there any flexibility in assigning inter departmental responsibilities among staff?<br>In that case if we see any baby whose condition is improving then we discharge.	
**4.5**	What is the mechanism of taking leave and who sanctions it?<br>Our leaves are sanctioned by Dr.	
**5. Duty Roster**		
**5.1**	Who prepares the duty roster for you?<br>RMO Dr.	
**5.2**	Who follows up the prepared roster so that the shifts are routinely changed?<br>Dr.	
**5.3**	How many Medical Officers are posted at one time in your department? What is the pattern of shift?<br>Only one in morning, evening and night	
**5.4**	Do you have flexibility in changing the shifts?<br>Yes there is difficulty in that	
**5.5**	How do you manage when you have double shifts?<br>We don't have any double shifts	
**6. Infrastructure**		
**6.1**	Do you have space to accommodate changes inside the department?<br>Yes there is adequate space but it's my request to make some changes here	
**6.2**	Do you have enough beds to accommodate increased number of patients?<br>No, even phototherapy machines are less, if more babies are admitted then they are admitted like 3 babies on 1 bed.	
**6.3**	Is their regular power supply and clean water for drinking? Any substitute available in case of power cut or irregular water supply?<br>Yes we don't face any issues of electricity & water supply.	
**7. Training /Skills**		
**7.1**	How many of the total staff are trained for MCH services?<br>Don't know	
**7.2**	Is there any pre job posting training for newly joined staff?<br>Yes Dr. use to give trainings	
**7.3**	Is there any on- job training for the staff?<br>Yes	
**7.4**	Please let us know about the last training on attended by you?<br>I didn't attend any such sort of trainings	
	Timing (Month/Year)	
	Place	
	Duration (in days)	
	What did you like the most in the training?	
	What did you dislike the most in the training?	
	What was the training methodology used (Lectures/ Hands-on / Practical's)?	
	Who conducts the workshop? Who prepares roster for workshop/training and how it is notified? How it is monitored?	
**7.5**	What are the opportunities and mechanisms currently in place/adopted to retain the skills of	

	Nurses/ANMs/Doctors?<br>Now I don't have any such knowledge of that
**7.6**	How the training related to care during delivery and newborn period can be further improved?<br>There should be regular trainings and if new equipments are availed here then we also can learn new things.
**7.7**	Have you ever visited/attended the skill labs operational in your district? Where it was conducted? Who conducted it? What was the time duration of skill lab training?<br>No, I don't know about it
**7.8**	What are the good things about this skill lab?<br>NA
**7.9**	What are the challenges related to skill lab?<br>NA
**7.10**	In your opinion, how many health staffs might have used or visited the skill labs?<br>NA
**7.11**	Did somebody advise or persuade you to attend the skill lab?<br>NA
**8. Referral services**	
**8.1**	In what situation usually the newborns/mothers (pregnant/recently delivered) are referred to the next level of healthcare?<br>Here most of the cases that come are of jaundice cases and other cases such as LBW, Respiratory distress are referred
**8.2**	Where the newborns/ pregnant women/ mothers are usually referred, what is the usual mode of transportation and how long does it takes to reach the next level health facility in your area?<br>PGI Rohtak from here ambulance use to go and it hardly takes 1 hour to reach there
**8.3**	What facilitation is done from facility side for referral and what difficulties/challenges do you face while transporting the sick newborn and mother to next level? *(Probe: monetary/logistics)*<br>I don't have any idea about it as I never faced any challenges in it.
**8.4**	What are the challenges faced related to referral transport experienced by this facility and how are they handled?<br>No we didn't face any such issues at least in my duty hours I didn't faced any.
**9. Logistics**	
**9.1**	Are you aware of any shortage/irregular supply of drugs and/or supplies needed for care during delivery and newborn period in the last one year? What were the reasons for this shortage and how these situations were managed?<br>Its been 3 months that I have joined here and in these 3 months I have never seen any excessive shortage
**9.2**	How frequently the families/ parents asked to procure drugs from outside/ store?<br>It happens rarely, in case if there is any medicine that has to be given for any particular problem and if it is not available here then we advice them to take from outside.
**9.3**	How many equipments essential for management of delivery or newborn care are out of order at this moment?<br>Presently now I think 1 phototherapy machine is not functional rest all the machines are functional.
**9.4**	What is the usual mechanism of repair and maintenance of these equipments? *(probe: who is responsible and what is the duration of repair*)<br>That all are managed by the staff nurse
**9.5**	What are the supervisory mechanisms in place at present for maternal and newborn care services?
	Who supervises<br>Its done by us or Dr.
	What is the frequency of supervisory visits<br>In the morning sir use to 1-2 rounds and in the evening shifts I will be there or any other MO
	Is any feedback/report provided usually after the supervision?

	Yes we use to tell the family members about the present condition of the child
	What actions are taken after last supervisory visit?
**9.6**	Please let us know about the last supervisory visit to the facility related to maternal and newborn care services?
	Who came for last supervisory visit?<br>Today it was done by me
	How long ago the supervisory visit took place?<br>Morning sir has done
	What all components were observed?<br>Like for the condition of jaundice we see blood reports, sucking reflex, feeding etc we check
	What feedback was given and what actions were taken?<br>If the child is completely fine then we tell for discharge or if the condition is not good then we tell about the refer
**10. Perceptions regarding Quality of care**	
**10.1**	According to you, what is the meaning of quality?<br>Quality means better care, better facilities like we see in private hospitals
**10.2**	According to you, what are the issues that affect the quality of health services?<br>If I tell you about SNCU then here there are no good equipments available here and evening and night staff are limited.
**10.3**	What can you do to improve the quality of the health services?<br>First I will provide duty to the staff and the most important is to control the crowd here
**10.4**	Did any of your relatives, friends or acquaintances ever availed health services at this hospital? If not, any reason?<br>Yes they do there is no issue in that
**11. Others**	
**11.1**	If any shortage of blood and how is it tackled?
**11.2**	Do you arrange blood donation camps on facility basis?

ID: 2108102			
**1 Type of Health Facility- DH, REWARI**			**1.2 Designation: staff nurse**
**2. General**			
**2.1**	How long have you been working in this health facility? (months/years)-since may 2017		
**2.2**	Total months/years of service -1 month		
**2.3**	What are your current roles and responsibility with respect to maternal and neonatal care?<br>I am looking after the SNCU as well as pediatric ward here I am taking care of their medicines, blood transfusion etc.		
**2.4**	WHow many deliveries and resuscitations of newborns have you attended in last 1 month?		
	A. No. of deliveries attended in last 1 month		I am not conducting delivery
	B. No. of newborn resuscitations attended in last 1 month		I have just joined that's why not a single one yet.
**2.5**	Who did you receive the training from:		

	Area	Training name	Year
A	Care during delivery (S.B.A.)	SBA	2014 January
B	Neonatal Resuscitation Program (In RP / NSS)	IMNCI	2010
C	Sick Newborn Care (FBNC)	NIl	Nil

3. Service Delivery

3.1 What are the challenges faced by you and your colleagues for delivering the desired mother and newborn care services?

Challenges faced

	mother care	newborn care
Infrastructure	NA	Actually I didn't faced any such challenges yet
Equipment	NA	Essential equipments are all available here, there is no such difficulty here
Drugs and supplies	NA	Only those medicines that are available here prescribed.
Support services	NA	I don't know yet about it.
Other	NA	No Problem

3.2 What challenges do you face while delivering essential newborn care services and how do you manage these?

**Challenges faced**	**How do you manage these challenges**
Delivery without complication	NA
Delivery with complication	I do whatever advises are given by the Dr. Rest I have few experience also that's why I use to manage myself also.
Caesarean section	No

3.3 What challenges do you face while delivery of pregnant women?

**Challenges faced**	**How do you manage these challenges**
Delivery without complication	NA
Delivery with complication	NA
Caesarean section	NA
Referred cases with complication	NA

3.4 How long usually the mothers stay at the facility after the delivery?

Normal Delivery	48 hours
Caesarean Delivery	I don't know

4. Manpower

4.1 How many posts of Staff Nurse / ANM are vacant in your health facility?
Here there is no vacant position moreover extra staff are hired here. Like now 3 post were vacant

	but 6 were hired
**4.2**	What difficulties do you face in providing mother and newborn care services to existing employees? (Doctors, nurses and other staff)<br>No there is no problem. The only thing is only 1 nurse is on duty and it becomes here only responsibility.
**4.3**	What is the mechanism of taking leave and who sanctions it?<br>No training is given to me yet here.
**5. Duty Roster**	
**5.1**	Who prepares the duty roster for you?<br>Staff roster is made by nursing sister
**5.2**	Do you have flexibility in changing the shifts?<br>Only in the case of emergency otherwise it is very difficult to change the duty or I can say It is not changed also.
**5.3**	How do you manage when you have double shifts?<br>Only night shift is done that we call as double shift otherwise we have 6 hours of duty.
**5.4**	What is the procedure for taking leave and who approves it?<br>If we require only 1-2 days of leave then sister manages it but if we require more number of days of leave we have to write one application and forward it to MS.
**5.5**	Who prepares rosters for emergency / regular service?<br>Nursing sister
**6. infrastructure**	
**6.1**	Do you have space to accommodate changes inside the department?<br>There is no space the available space is already occupied
**6.2**	Do you have enough beds to accommodate increased number of patients?<br>No in that case we admit on bed 2-3 patients
**6.3**	Is their regular power supply and clean water for drinking? Any substitute available in case of power cut or irregular water supply?<br>Yes water and electricity is available here 24 hours .Even if it goes we have generator here.
**7. Data management**	
**7.1**	How do you record data?<br>We use to do all entry on the register itself. We have different registers for different cases like Thalasemia register, Refer register, admission & discharge register etc.
**7.2**	How do you maintain a register?
**7.3**	Where do you send the record?<br>Every month there is one person in data entry who takes record and does the entry in the system.
**7.4**	How often is the data sent?<br>Monthly
**8. Blood bank**	
**8.1**	How long does it take for a needy person to get blood?<br>30 mins
**9. Training /Skills**	
**9.1**	Could you tell us about your previous NSSK / Neonatal Resuscitation Training?

	Time (month / year)	2010
	place	DH
	Duration (in days)	2 days

**9.2**	Who conducts the workshop? Who prepares roster for workshop/training and how it is notified? How it is monitored?<br>There is one particular person in the CMO office he use to make
**9.3**	What did you like the most in the training?

.

	We got to learn many things there, trainers were very good.
**9.4**	What did you dislike the most in the training?<br>Only one thing the trainer use to address us again and again as Nurse-Nurse. Whereas in the govt policy it is written sister ji. For that reason I had argument also that you can call me by my name rather than again saying nurse.
**9.5**	What was the training methodology used (Lectures/ Hands-on / Practical's)?<br>Dummy & projectors were used.
**9.6**	How did you like meeting / interacting with the trainers?<br>It was very good
**9.7**	What was their level of knowledge / skills? — Good
	How was your conversation with him — Good
**9.8**	What are the opportunities and mechanisms currently in place/adopted to retain the skills of Nurses/ANMs/Doctors?<br>Warning should be given to nurse/Doctor as here no person have fear for anything as no matter what all things they are doing it wrong no actions are taken on them.
**9.9**	What challenges do you have with the skills of nurses and the support of staff in delivery rooms, perinatal wards and newborn care units? In your opinion, how can this be controlled?<br>Don't know
**9.10**	How the training related to care during delivery and newborn period can be further improved?<br>There should be training for us regularly. Here it has been years that people have got any training.
**9.11**	Have you been to a skill lab set up in your district?<br>NO
**9.12**	What are the good things about this skill lab?<br>NA
**9.13**	What are the challenges related to skill lab?<br>NA
**9.14**	In your opinion, how many health staffs might have used or visited the skill labs?<br>NA
**9.15**	Did somebody advise or persuade you to attend the skill lab?<br>NA
**9.16**	How does the Skill Lab help in Neonatal Resuscitation and Neonatal Care?<br>NA
**10. Referral services**	
**10.1**	In what situation usually the newborns/mothers (pregnant/recently delivered) are referred to the next level of healthcare?<br>When the condition of the child is very bad as here only basic facilities are available to provide to the child.
**10.2**	Where the newborns/ pregnant women/ mothers are usually referred, what is the usual mode of transportation and how long does it takes to reach the next level health facility in your area?<br>PGI Rohtak maximum it takes 1 hour as the roads are good to reach
**10.3**	What facilitation is done from facility side for referral and what difficulties/challenges do you face while transporting the sick newborn and mother to next level? *(Probe: monetary/logistics)*<br>We have good ambulance here and In the ambulance EMP person also accompany for the referring cases
**10.4**	What are the challenges faced related to referral transport experienced by this facility and how are they handled?<br>If there is any strike or the ambulance is not available then in that case people have to hire there own private vehicle
**11. logistics**	

.

**11.1**	Are you familiar with any scarcity / irregular supply of medicines and / or supplies required for care during delivery and newborn in the last one year? What were the reasons for this deficiency and how were these conditions managed?<br>Meptal P sometimes are prescribed which is not available here. In that case patient has to buy from outside
**11.2**	How frequently the families/ parents asked to procure drugs from outside/ store?<br>Meptal P is prescribed very rarely otherwise the medicine available here are only prescribed.
**11.3**	What are the supervisory mechanisms in place at present for maternal and newborn care services?
	Who supervises<br>Nurse alone are doing
	What is the frequency of supervisory visits<br>We provide immediately like if Dr. prescribed for blood transfusion or IV fluid transfusion we start immediately.
	Is any feedback/report provided usually after the supervision?<br>Yes then only that can be done
	What actions are taken after last supervisory visit?<br>That is known only by Dr. only as we do as we are instructed
**11.4**	Please let us know about the last supervisory visit to the facility related to maternal and newborn care services?
	Who came for last supervisory visit?<br>Dr. only tell about the discharge
	How long ago the supervisory visit took place?<br>It is done immediately
	What all components were observed?<br>Just vital signs, and the problems with which the person is admitted we check whether that is improved or not.
	What feedback was given and what actions were taken?<br>We tell them to look after the diet and the date of follow up is given.
**12. others**	
**12.1**	How wide do the events in discharge / record slip?<br>Medicines and vital signs.
**12.2**	In your view, what are the perceived barriers between families in the use of public health services for newborns of pregnant women?<br>Now that I don't know much<br>How can these obstacles be overcome?
**12.3**	How much additional effort is needed to reduce the neonatal mortality rate in your area?<br>There should be timely ANC Check up
**12.4**	According to you, what is the meaning of quality?<br>According to me quality in healthcare means to provide procedure wise treatment to the patients.
**12.5**	According to you, what are the issues affecting the quality (quality) of health services?<br>No I don't see any problem
**12.6**	What can you do to improve the quality of the health services?<br>If everyone works together then quality of work will increase as it will improve the work. Actually what happens now is Doctor is sometimes available and sometimes not and when he comes he just give order and go and nurses have to do all the work. Due to excessive work load she sometimes forget some work also.<br>The presently joined fresher Dr needs good supervision a they leave the ward and roam around and in due course if anything wrong happens then nurses have to listen to the scoldings.

.

**12.7**	Did any of your relatives, friends or acquaintances ever availed health services at this hospital? If not, any reason?<br>Yes they use to come.

.

ID: 2108103	
**1 Type of Health Facility rewari, dh**	**1.2 Designation: staff nurse**

2. General

**2.1**	How long have you been working in this health facility? (months/years)-since 16 feb 2013
**2.2**	Total months/years of service -4.5 years
**2.3**	What are your current roles and responsibility with respect to maternal and neonatal care?<br>I don't have any much of the responsibility on the newborn. Most f the responsibility is for the Pregnant mother Like to conduct delivery, provide medication, Conduct ANC, and sometimes do resuscitation for the babies and to make report also.
**2.4**	How many deliveries and resuscitations of newborns have you attended in last 1 month?

2.4.1 No. of deliveries attended in last 1 month<br>Presently I am posted in HRPU si last 1 month I didn't conducted any delivery otherwise I use to conduct in my duty 3-4 deliveries	
2.4.2 No. of newborn resuscitations attended in last 1 month<br>Not even a single one.	

2.5 Who did you receive the training from:

	Area	Training name	Year
A	Care during delivery (S.B.A.)	S.B.A	Nov, 2015
B	Neonatal Resuscitation Program (In RP / NSS)		I didn't had any training for that
C	Sick Newborn Care (FBNC)		No

3. Service Delivery

3.1 What are the challenges faced by you and your colleagues for delivering the desired mother and newborn care services?

Challenges faced

	**Mother care**	**Newborn care**
Infrastructure	Yes one problem here is that Doctor and Nurses are all sitting in same OPD due to which the OPD gets crowded	
Equipment	No issue in this	
Drugs and supplies	Medicines are all available there is no particular issue in that	
Support services	Yeah one issue in this is that we are not getting extra nurses in case of any necessity or for help due to which workload is doubled	
Other	Rest what else I should say everything Is fine only.	

3.2 What challenges do you face while delivering essential newborn care services and how do you manage these?

**Challenges faced**	**How do you manage these challenges**
Care at delivery	Didn't face any difficulty yet
Care in the ward	Sometimes we need to admit 2-3 mothers on one single bed.
Care of sick newborns	NA

3.3 What challenges do you face while delivery of pregnant women?

**Challenges faced**	**How do you manage these challenges**
Delivery without complication	Relatives of the mother start getting on to us after 24 hours of delivery that we want to go home.
Delivery with complication	NR
Caesarean section	NR
Referred cases with complication	Sometimes there is no availability of the ambulance

3.4 How long usually the mothers stay at the facility after the delivery?

Normal Delivery	48 hours

	Caesarean Delivery	4 days
**4. Manpower**		
**4.1**	How many posts of Staff Nurse / ANM are vacant in your health facility?<br>Don't know about it there were 10 post vacant in RCH all the 10 are filled rest of that I don't know	
**4.2**	What difficulties do you face in providing mother and newborn care services to existing employees? (Doctors, nurses and other staff)<br>Actually not facing any such problem	
**4.3**	What is the mechanism of taking leave and who sanctions it?<br>Yes we people only train the fresher's by giving them duty during the morning shift and with seniors like that	
**5. Duty Roster**		
**5.1**	Who prepares the duty roster for you?<br>Duty roster is made by nursing sister	
**5.2**	Do you have flexibility in changing the shifts?<br>Yes we use to adjust amongst ourselves and inform nursing sister	
**5.3**	How do you manage when you have double shifts?<br>For us the double shift is the night shift itself , rest I don't face any problem in this	
**5.4**	What is the procedure for taking leave and who approves it?<br>If we need leave we get CL the only thing we need to do is to inform the nursing sister about it, which can be done telephonically.	
**5.5**	Who prepares rosters for emergency / regular service?<br>No there is no system like this	
**6. infrastructure**		
**6.1**	Do you have space to accommodate changes inside the department?<br>Yes there is adequate space over here the only thing needed is to use wisely for the improvement.	
**6.2**	Do you have enough beds to accommodate increased number of patients?<br>No there is no adequate beds available. In one bed we use to adjust 2-3 mothers at a time. Sometimes 4 deliveries come together in that case we have to deliver one mother by lying her on the floor as we have only 3 delivery beds.	
**6.3**	Is their regular power supply and clean water for drinking? Any substitute available in case of power cut or irregular water supply?<br>We have generators and also invertors so we don't have any problem in that.	
**7. Data management**		
**7.1**	How do you record data?<br>We use to do daily entry on the registers.	
**7.2**	How do you maintain a register?<br>We have 7-8 registers so we do daily entries in that, that itself is one big task	
**7.3**	Where do you send the record?<br>I use to send monthly records to who is the HMIS person	
**7.4**	How often is the data sent?<br>Every monthly reports	
**8. Blood bank**		
**8.1**	How long does it take for a needy person to get blood?<br>It takes 1 hour	
**9. Training /Skills**		
**9.1**	Could you tell us about your previous NSSK / Neonatal Resuscitation Training?	
	Time (month / year)	
	place	
	Duration (in days)	
**9.2**	Who conducts the workshop? Who prepares roster for workshop/training and how it is notified?	

.

	How it is monitored?<br>There is one in the CMO office he use make the Training roster for all.	
**9.3**	What did you like the most in the training?<br>Now it’s already been so many years I don’t remember anything.	
**9.4**	What did you dislike the most in the training?<br>Everything was good only I don’t remember that anything was not.	
**9.5**	What was the training methodology used (Lectures/ Hands-on / Practical’s)?<br>For the training they used the projector	
**9.6**	How did you like meeting / interacting with the trainers?<br>It was good that they taught very nicely	
**9.7**	What was their level of knowledge / skills?	It was very good
	How was your conversation with him	It was ok
**9.8**	What are the opportunities and mechanisms currently in place/adopted to retain the skills of Nurses/ANMs/Doctors?<br>There should be training for all on the regular basis	
**9.9**	What challenges do you have with the skills of nurses and the support of staff in delivery rooms, perinatal wards and newborn care units? In your opinion, how can this be controlled?<br>Don’t know	
**9.10**	How the training related to care during delivery and newborn period can be further improved?<br>Now what can I say, what I want is some way to control the crowd	
**9.11**	Have you been to a skill lab set up in your district?<br>I don’t have any knowledge about this	
**9.12**	What are the good things about this skill lab?<br>NA	
**9.13**	What are the challenges related to skill lab?<br>NA	
**9.14**	In your opinion, how many health staffs might have used or visited the skill labs?<br>NA	
**9.15**	Did somebody advise or persuade you to attend the skill lab?<br>NA	
**9.16**	How does the Skill Lab help in Neonatal Resuscitation and Neonatal Care?<br>NA	
**10. Referral services**		
**10.1**	In what situation usually the newborns/mothers (pregnant/recently delivered) are referred to the next level of healthcare?<br>We refer in the case of PIH, Cord prolapsed, severe anemia like that.	
**10.2**	Where the newborns/ pregnant women/ mothers are usually referred, what is the usual mode of transportation and how long does it takes to reach the next level health facility in your area?<br>We use to give option sometimes like PGI Rohtak or Safdarjung Delhi for both places it takes 1 hour	
**10.3**	What facilitation is done from facility side for referral and what difficulties/challenges do you face while transporting the sick newborn and mother to next level? *(Probe: monetary/logistics)*<br>There is all facilities in the ambulance. there is one EMP staff accompanied during referral	
**10.4**	What are the challenges faced related to referral transport experienced by this facility and how are they handled?<br>If there is no ambulance that time it causes a problem. The patient needs to wait or they need to be referred to the private	
**11. logistics**		

.

**11.1**	Are you familiar with any scarcity / irregular supply of medicines and / or supplies required for care during delivery and newborn in the last one year? What were the reasons for this deficiency and how were these conditions managed?<br>No I don't remember anything like that
**11.2**	How frequently the families/ parents asked to procure drugs from outside/ store?<br>Nothing like that happened in front of me
**11.3**	What are the supervisory mechanisms in place at present for maternal and newborn care services?
	Who supervises<br>Everything is done by the nurses itself
	What is the frequency of supervisory visits<br>Immediately
	Is any feedback/report provided usually after the supervision?<br>Yes why not
	What actions are taken after last supervisory visit?<br>Yes Doctor after checking only and seeing the reports finalize for further actions.
**11.4**	Please let us know about the last supervisory visit to the facility related to maternal and newborn care services?
	Who came for last supervisory visit?<br>Doctor does the final check up after that only they tell finally what to do.
	How long ago the supervisory visit took place?<br>It is done immediately
	What all components were observed?<br>We see vitals and check the problem with which the mother came here that it is rectified or not.
	What feedback was given and what actions were taken?<br>Now that I don't know
**12.** Others	
**12.1**	How wide do the events in discharge / record slip?<br>On the discharge slip time, date, baby's information, vital signs, HB, ANC etc are written on it.
**12.2**	In your view, what are the perceived barriers between families in the use of public health services for newborns of pregnant women?<br>Don't know<br>How can these obstacles be overcome?
**12.3**	How much additional effort is needed to reduce the neonatal mortality rate in your area?<br>Here there is no such cases of maternal deaths only 1 case occurred last year, other than that All the pregnant mother should come for ANC check timely to the facility
**12.4**	How much additional effort is needed to reduce the neonatal mortality rate in your area?
**12.5**	According to you, what are the issues affecting the quality (quality) of health services?
**12.6**	What can you do to improve the quality of the health services?
**12.7**	Did any of your relatives, friends or acquaintances ever availed health services at this hospital? If not, any reason?

.

ID: 2108105		
1 Type of Health Facility dh, Rewari		1.2 Designation: staff nurse

2. General

**2.1**	How long have you been working in this health facility? (months/years)-since may 2015
**2.2**	Total months/years of service -2 years
**2.3**	What are your current roles and responsibility with respect to maternal and neonatal care?<br>I don't have any responsibilities of Newborn as they are managed by the SNCU staff. I am here for pregnant women for Conducting delivery admissions, discharge, referral etc are seen here.
**2.4**	How many deliveries and resuscitations of newborns have you attended in last 1 month

2.4.1 No. of deliveries attended in last 1 month	20-25
2.4.2 No. of newborn resuscitations attended in last 1 month	Nil

2.5 Who did you receive the training from:

	Area	Training name	Year
A	Care during delivery (S.B.A.)	SBA	2016
B	Neonatal Resuscitation Program (In RP / NSS)	No	
C	Sick Newborn Care (FBNC)		

3. Service Delivery

3.1 What are the challenges faced by you and your colleagues for delivering the desired mother and newborn care services?

	challenges faced	
	**Mother care**	**Newborn care**
Infrastructure	Here due to overcrowding it is difficult to control the mother & their relatives	
Equipment	No there is no difficulty in that	
Drugs and supplies	Here only those medicines are prescribed that is available over here.	
Support services	No these all things here are good	
Other	Here one problem is that whenever mother is coming for ANC checkup most of the mothers are not bringing their old card due to which it becomes difficult to record	

3.2 What challenges do you face while delivering essential newborn care services and how do you manage these?

Challenges faced	How do you manage these challenges
Care at delivery	No there is no issues in this yes one problem comes when at a time 4-5 deliveries come which causes a lot of problems.
Care in the ward	We admit on one bed 2 mothers and discharge the one who is ok.
Care of sick newborns	

3.3 What challenges do you face while delivery of pregnant women?

Challenges faced	How do you manage these challenges
Delivery without complication	
Delivery with complication	
Caesarean section	
Referred cases with complication	

3.4 How long usually the mothers stay at the facility after the delivery?

Normal Delivery	48 hours

	Caesarean Delivery	3-4 days
**4. Manpower**		
**4.1**	How many posts of Staff Nurse / ANM are vacant in your health facility?<br>I heard that there are 2 post vacant but I am not sure about it as it is heard that 2 staff from RCH are transferred but I am not sure about it.	
**4.2**	What difficulties do you face in providing mother and newborn care services to existing employees? (Doctors, nurses and other staff)	
**4.3**	What is the mechanism of taking leave and who sanctions it?<br>Yes fresher staffs are given morning duty along with a senior staff who trains the new comer.	
**5. Duty Roster**		
**5.1**	Who prepares the duty roster for you?<br>Our Nursing sister	
**5.2**	Do you have flexibility in changing the shifts?<br>Yes it can be done in case of any emergency	
**5.3**	How do you manage when you have double shifts?<br>There is no double shifts only night shifts are of 12 ours	
**5.4**	What is the procedure for taking leave and who approves it?<br>We just need to inform nursing sister than we can get leave	
**5.5**	Who prepares rosters for emergency / regular service?<br>She only does but presently as she is sick matron is doing it.	
**6. Infrastructure**		
**6.1**	Do you have space to accommodate changes inside the department?<br>No I can't see any vacant space here	
**6.2**	Do you have enough beds to accommodate increased number of patients?<br>No there is not. Although currently patients are less those are admitted. There are few months in which there is increased no of cases du to which we have to give 1 bed to 3 mothers as we are helpless and they adjust amongst themselves	
**6.3**	Is their regular power supply and clean water for drinking? Any substitute available in case of power cut or irregular water supply?<br>Yes there is no difficulty	
**7. Data management**		
**7.1**	How do you record data?<br>Here there are 7-8 registers we do entry in these registers.	
**7.2**	How do you maintain a register?	
**7.3**	Where do you send the record?<br>For that we make a monthly report and then hand it to Nursing sister	
**7.4**	How often is the data sent?<br>Once every month	
**8. Blood bank**		
**8.1**	How long does it take for a needy person to get blood?<br>It takes 1 hour as sometimes the blood is too cold to transfuse	
**9. Training /Skills**		
**9.1**	Could you tell us about your previous NSSK / Neonatal Resuscitation Training?	
	Time (month / year)	
	place	
	Duration (in days)	
**9.2**	Who conducts the workshop? Who prepares roster for workshop/training and how it is notified? How it is monitored?<br>We get it from CMO office	
**9.3**	What did you like the most in the training?	

.

	There are many things like I got 1 week of leave from the facility plus we got money for per day training and also got to learn new things	
**9.4**	What did you dislike the most in the training?<br>There was nothing like that	
**9.5**	What was the training methodology used (Lectures/ Hands-on / Practical's)?<br>It was done using the projector	
**9.6**	How did you like meeting / interacting with the trainers?<br>I didn't talk to him like that.	
**9.7**	What was their level of knowledge / skills?	It was good
	How was your conversation with him	
**9.8**	What are the opportunities and mechanisms currently in place/adopted to retain the skills of Nurses/ANMs/Doctors?<br>Work load should be reduced and we should get trainings regularly.	
**9.9**	What challenges do you have with the skills of nurses and the support of staff in delivery rooms, perinatal wards and newborn care units? In your opinion, how can this be controlled?	
**9.10**	How the training related to care during delivery and newborn period can be further improved?<br>I don't know about it I think you only know things better.	
**9.11**	Have you been to a skill lab set up in your district?<br>Don't know	
**9.12**	What are the good things about this skill lab?	
**9.13**	What are the challenges related to skill lab?	
**9.14**	In your opinion, how many health staffs might have used or visited the skill labs?	
**9.15**	Did somebody advise or persuade you to attend the skill lab?	
**9.16**	How does the Skill Lab help in Neonatal Resuscitation and Neonatal Care?	
**10. Referral services**		
**10.1**	In what situation usually the newborns/mothers (pregnant/recently delivered) are referred to the next level of healthcare?<br>If the level of HB is too low or there is complication in delivery then only we refer	
**10.2**	Where the newborns/ pregnant women/ mothers are usually referred, what is the usual mode of transportation and how long does it takes to reach the next level health facility in your area?<br>PGI Rohtak or safdarjung in our ambulance and it takes around 1 hour to reach	
**10.3**	What facilitation is done from facility side for referral and what difficulties/challenges do you face while transporting the sick newborn and mother to next level? *(Probe: monetary/logistics)*<br>EMT staff use to go along with them and if sometimes ambulance is not available then they use to go in private vehicle.	
**10.4**	What are the challenges faced related to referral transport experienced by this facility and how are they handled?<br>We use to tell them to wait or hire any private vehicle. Actually I don't know much about it.	
**11. Logistics**		
**11.1**	Are you familiar with any scarcity / irregular supply of medicines and / or supplies required for care during delivery and newborn in the last one year? What were the reasons for this deficiency and how were these conditions managed?<br>No I don't know anything about it.	
**11.2**	How frequently the families/ parents asked to procure drugs from outside/ store?<br>Very rarely it happens	
**11.3**	What are the supervisory mechanisms in place at present for maternal and newborn care services?	
	Who supervises<br>There is LMO she only does	
	What is the frequency of supervisory visits<br>We do immediately as soon as mother reaches to us.	

.

	Is any feedback/report provided usually after the supervision?<br>Yes of course otherwise how will we know
	What actions are taken after last supervisory visit?<br>Yes
**11.4**	Please let us know about the last supervisory visit to the facility related to maternal and newborn care services?
	Who came for last supervisory visit?<br>LMO or the nurse available for duty
	How long ago the supervisory visit took place?<br>It don't take much time
	What all components were observed?<br>B.P, temperature and the condition of the mother that's it.
	What feedback was given and what actions were taken?
**12. others**	
**12.1**	How wide do the events in discharge / record slip?
**12.2**	In your view, what are the perceived barriers between families in the use of public health services for newborns of pregnant women?<br>How can these obstacles be overcome?
**12.3**	How much additional effort is needed to reduce the neonatal mortality rate in your area?
**12.4**	According to you, what is the meaning of quality?<br>According to me quality means to provide care on timely manner and whatever the problem with which mother or other patients come here should face minimal problems.
**12.5**	According to you, what are the issues affecting the quality (quality) of health services?<br>The biggest problem here is the crowd which gets out of control many times. Secondly there is only one sitting area for both Doctors as well as nurse.
**12.6**	What can you do to improve the quality of the health services?<br>Now what can I say, first of all I would like to have a guard who is capable to manage the crowd. Second there should be training for nurses and our salary should be increased as since last 3 years I am getting 10,000 only.
**12.7**	Did any of your relatives, friends or acquaintances ever availed health services at this hospital? If not, any reason?<br>Yes they use to come whenever it is necessary

.

ID: 2108106

**1 Type of Health Facility DH, REWARI**	**1.2 Designation: STAFF NURSE**

2. General

**2.1**	How long have you been working in this health facility? (months/years)-since 18/10/2009
**2.2**	Total months/years of service -8 years
**2.3**	How many deliveries and resuscitations of newborns have you attended in last 1 month?<br>Every responsibilities that is needed for babies I am doing here like in SNCU from the time of admission till the time of discharge , if there is no doctor available here I use to do refer also as the saving of child life is very much important
**2.4**	How many deliveries and resuscitations of newborns have you attended in last 1 month?

**2.4**	2.4.1 No. of deliveries attended in last 1 month	NA (SNCU staff not conducting delivery)
	2.4.2 No. of newborn resuscitations attended in last 1 month	4-5 in last one month don't know about rest of the staff

2.5 Who did you receive the training from:

	Area	Training name	Year
A	Care during delivery (S.B.A.)	No training at all	
B	Neonatal Resuscitation Program (In RP / NSS)	2 days of training of NSSK	It was done before 2012 after that I didn't got any training.
C	Sick Newborn Care (FBNC)	No	

3. Service Delivery

3.1 What are the challenges faced by you and your colleagues for delivering the desired mother and newborn care services?

challenges faced		
	**Mother care**	**Newborn care**
Infrastructure	NA	Everything is fine there is no difficulty we are facing related to infrastructure
Equipment	NA	No problem
Drugs and supplies	NA	Medicines are all available here , there is no need for taking medicines from outside
Support services	NA	Everything is fine here I don't face any problem
Other	NA	The only problem is there is only 1 staff in SNCU which is sometimes challenging

3.2 What challenges do you face while delivering essential newborn care services and how do you manage these?

**Challenges faced**	**How do you manage these challenges**
Care at delivery	I have conducted delivery way back in the past now a days I don't do so don't know much about it.
Care in the ward	I don't work in ward
Care of sick newborns	There is only 1 staff nurse that is the only problem here

3.3 What challenges do you face while delivery of pregnant women?

**Challenges faced**	**How do you manage these challenges**
Delivery without complication	NA
Delivery with complication	NA
Caesarean section	NA
Referred cases with complication	NA

3.4 How long usually the mothers stay at the facility after the delivery?

Normal Delivery	NA
Caesarean Delivery	NA

**4. Manpower**	
**4.1**	How many posts of Staff Nurse / ANM are vacant in your health facility?<br>No I don't know about it.
**4.2**	What difficulties do you face in providing mother and newborn care services to existing employees? (Doctors, nurses and other staff)<br>Doctors are not here sometimes like now that's why there is some issues in handling the work. Other than that everything is fine
**4.3**	What is the mechanism of taking leave and who sanctions it?<br>Yes Dr. use to give complete training.
**5. Duty Roster**	
**5.1**	Who prepares the duty roster for you?<br>Nursing sister
**5.2**	Do you have flexibility in changing the shifts?<br>Yes but it is adjusted in the case of utmost emergency, otherwise it is very difficult.
**5.3**	How do you manage when you have double shifts?<br>Double shift night shift only otherwise our duty hour is of 6 hours.
**5.4**	What is the procedure for taking leave and who approves it?<br>For leave we have to write an application and forward to MS, then he only confirms about the leave.
**5.5**	Who prepares rosters for emergency / regular service?<br>sister only makes. Actually such type of problems never occurred
**6. Infrastructure**	
**6.1**	Do you have space to accommodate changes inside the department?<br>Yes the space that you can see over here this much space we have. Here according to the space you can make necessary changes.
**6.2**	Do you have enough beds to accommodate increased number of patients?<br>Yes in the step down unit we have 3 beds there we admit the sick child first there then we discharge the newborns who are getting better.
**6.3**	Is their regular power supply and clean water for drinking? Any substitute available in case of power cut or irregular water supply?<br>Yes for the electricity and water we have no issues.
**7. Data management**	
**7.1**	How do you record data?<br>We make in the registers the way I am doing now.
**7.2**	How do you maintain a register?<br>We use to make daily records in this register and before going I use to give the record to the data entry operator over here that makes entry into the system.
**7.3**	Where do you send the record?<br>I use to give to the data entry operator and sometimes nursing sister use to come and ask then I give to her also.
**7.4**	How often is the data sent?<br>After every shift we use to give to the data entry operator and for evening and night shift duty reports are given in the morning.
**8. Blood bank**	
**8.1**	How long does it take for a needy person to get blood?<br>Here no blood transfusion is given, for babies we are providing blood transfusion in the room no.8
**9. Training /Skills**	
**9.1**	Could you tell us about your previous NSSK / Neonatal Resuscitation Training?

	Time (month / year)	2012
	place	Trauma centre
	Duration (in days)	2 days

.

**9.2**	Who conducts the workshop? Who prepares roster for workshop/training and how it is notified? How it is monitored?<br>Yes they use to make, there is one person in the CMO office he use to make the training roster.	
**9.3**	What did you like the most in the training?<br>The best thing I like is that I got 2 days leave from here.	
**9.4**	What did you dislike the most in the training?<br>No there was nothing like that, training was good only.	
**9.5**	What was the training methodology used (Lectures/ Hands-on / Practical's)?<br>For the training they used Ambu bag, dummy and projector.	
**9.6**	How did you like meeting / interacting with the trainers?<br>I like it very much got to learn many things from the training.	
**9.7**	What was their level of knowledge / skills?	It was very good.
	How was your conversation with him	Good
**9.8**	What are the opportunities and mechanisms currently in place/adopted to retain the skills of Nurses/ANMs/Doctors?<br>This I don't know	
**9.9**	What challenges do you have with the skills of nurses and the support of staff in delivery rooms, perinatal wards and newborn care units? In your opinion, how can this be controlled?<br>I don't know anything like that.	
**9.10**	How the training related to care during delivery and newborn period can be further improved?<br>Everything is good here I don't think there is any need for the change here	
**9.11**	Have you been to a skill lab set up in your district?<br>No I don't know anything about that.	
**9.12**	What are the good things about this skill lab?<br>NA	
**9.13**	What are the challenges related to skill lab?<br>NA	
**9.14**	In your opinion, how many health staffs might have used or visited the skill labs?<br>NA	
**9.15**	Did somebody advise or persuade you to attend the skill lab?<br>NA	
**9.16**	How does the Skill Lab help in Neonatal Resuscitation and Neonatal Care?<br>NA	
**10. Referral services**		
**10.1**	In what situation usually the newborns/mothers (pregnant/recently delivered) are referred to the next level of healthcare?<br>If for the newborns there is a need for the use of CPAP & ventilator. Because here only basic care is given nothing more than that.	
**10.2**	Where the newborns/ pregnant women/ mothers are usually referred, what is the usual mode of transportation and how long does it takes to reach the next level health facility in your area?<br>From here every cases are referred to Rohtak in ambulance. It takes 1 hour to reach Rohtak	
**10.3**	रे What facilitation is done from facility side for referral and what difficulties/challenges do you face while transporting the sick newborn and mother to next level? *(Probe: monetary/logistics)*<br>Ambulance is provided from here. EMT staff is going along in ambulance. In the ambulance all the basic care equipments are available. There is no problem in it.	
**10.4**	What are the challenges faced related to referral transport experienced by this facility and how are they handled?<br>There is separate person for that I don't know much about it.	
**11. Logistics**		

.

**11.1**	Are you familiar with any scarcity / irregular supply of medicines and / or supplies required for care during delivery and newborn in the last one year? What were the reasons for this deficiency and how were these conditions managed?<br>No nothing like that happened in front of me. After all Doctors are only prescribing those medicines that are available in the pharmacy.
**11.2**	How frequently the families/ parents asked to procure drugs from outside/ store?<br>Never
**11.3**	What are the supervisory mechanisms in place at present for maternal and newborn care services?
	Who supervises<br>We only do no one else does.
	What is the frequency of supervisory visits<br>Immediately
	Is any feedback/report provided usually after the supervision?<br>Yes doctor only give advice after seeing the report. We just follow the advice given by the Dr.
	What actions are taken after last supervisory visit?<br>We just discharge
**11.4**	Please let us know about the last supervisory visit to the facility related to maternal and newborn care services?
	Who came for last supervisory visit?<br>We only do no one else.
	How long ago the supervisory visit took place?<br>We do it immediately. We don't give much time to the babies who are fine.
	What all components were observed?<br>We use to see the heart rate, respiration, temperature, baby crying, breastfeeding or not, this all things we see.
	What feedback was given and what actions were taken?<br>If everything is fine we use to inform the Dr. then Discharge is made accordingly.
**12. others**	
**12.1**	How wide do the events in discharge / record slip?<br>Not much just the necessary things are written.
**12.2**	In your view, what are the perceived barriers between families in the use of public health services for newborns of pregnant women?<br>Don't know<br>How can these obstacles be overcome?<br>I don't know about it.
**12.3**	How much additional effort is needed to reduce the neonatal mortality rate in your area?<br>No here nothing like that happen nor I have seen so what can I tell you about this.
**12.4**	According to you, what is the meaning of quality?<br>Quality means providing good care
**12.5**	According to you, what are the issues affecting the quality (quality) of health services?<br>There is no shortcomings.
**12.6**	What can you do to improve the quality of the health services?<br>There should be recruitment of more nursing staff, we are not getting complete leave which we should get, Dr. should be available all the time here in this area.
**12.7**	Did any of your relatives, friends or acquaintances ever availed health services at this hospital? If not, any reason?<br>Yes they use to come

.

ID: 2108301	
AREA DH, Reawri	DESIGNATION : class IV
**S.No.**	**Questions**
**1.**	**General Information**
1.1	Are you an employee of the hospital or are you contracted for the same?<br>No I am on contract basis
1.2	How many classrooms are there in this hospital?<br>Here in this department there are 5 class 4 staff in which 3 remain during the morning shift 1 in the evening and 1 in the night.
**2.**	**Specific Information**
2.1	Please tell us about the duty of duty and how many people are present in the cross<br>In the duty 3 are in the morning shift 1 in the evening and 1 in the night.
2.2	Your responsibility is only of one department, of all the other departments of the hospital?<br>Only in this area in the rest of the area I only use to go if there is so much of an emergency.
2.3	Explain your responsibilities<br>Our responsibility here is dusting, transfer of files, Autoclaving, to bring the medicine stock, call the patients inside; sometimes we use to help the nurses as well.
2.4	Do you have a duty even during the night shift?<br>Yes we have duty on rotation.
2.5	Tell us which places in this hospital do you take care of cleanliness?<br>The area of the room no 22, ANC/PNC entry till the entry of SNCU we use to clean.
2.6	Tell us about your family's success?<br>During Morning and evening we use to do rest we use to clean when it is not clean.
2.7	What media do you use for cleaning many types of goods?<br>We use to use Duster, surf, scrubber etc.
2.8	Does anyone check your work?<br>Yes Dr. use to do and our supervisor also use to come sometimes for checking
**3.**	**Disposal**
3.1	How you are you dispose the waste?<br>Waste we dispose according to the color codes.
3.2	Do you use color coding systems for waste disposal? Detailed<br>Yes we use to do according to that only like in the black we dump general waste, in the red plastic waste, in the yellow blood soiled waste and in the blue sharp objects like ampoules and other glass objects.
3.3	Is there is any vehicle for pickup the waste?<br>Yes it uses to come twice a day.
3.4	If yes, then how many times is it come in the hospital?<br>It comes daily twice.
3.5	Do you burn the waste room? If so where<br>We use to do it earlier but nowadays the burning method is not practiced here.
**4.**	**Issues/Suggestions**
4.1	tell us how other staff members treat you in hospital<br>The behavior of the staff is good we never face any such problems.
4.2	tell us how patients treat you in the hospital<br>Patients are also good but sometimes 1-2 patients come with whom arguments happen
4.3	tell us what difficulty you face during your work<br>As now there is too much of crowding controlling them is very much difficult.

4.4	Want to change the way you do for better service?<br>Now what can I say about that you people only know better than me.
**4.5**	What do you mean by quality?<br>Quality means to provide good care to the patients so that when they go from here they are happy.
**4.6**	According to you, what are the issues affecting the quality of health services?<br>In this area I can say that the availability of guards, beds are also less in no are the biggest issue other than that overcrowding of the area.
**4.7**	What can you do to improve the quality of health services?<br>According to me there should be different OPD for Doctor and nurses now as they both are sitting in same place all the patients come inside even PNC mother's relatives due to which controlling them all becomes a major task.
**4.8**	Have your relatives, friends or acquaintances ever taken advantage of the health services of this hospital? If not, any reason?<br>Yes.

ID: 2108302	
**AREA dh, Reawri**	**DESIGNATION : class IV**
**S.No.**	**Questions**
**1.**	**General Information**
1.1	Are you an employee of the hospital or are you contracted for the same?<br>I am on contract basis in this hospital
1.2	How many class 4 are there in this hospital?<br>I don't know about complete hospital but in this SNCU I am the only class 4 working
**2.**	**Specific Information**
2.1	Please tell us about the duty of duty and how many people are present in the cross<br>There is only one shift in the morning and being the only class 4 in SNCU I am the only person available.
2.2	Your responsibility is only of one department, of all the other departments of the hospital?<br>Only of the SNCU but in case of any emergency I had to got to room no. 8(OPD) and R.No 22.
2.3	Explain your responsibilities<br>My responsibility here is of cleanliness, dusting, I have to look for the entry of mother and the child relative in the SNCU, sometimes has to take files from here to OPD or I have to bring the files from other places.
2.4	Do you have a duty even during the night shift?<br>No I don't have any other shift than morning shifts
2.5	Tell us which places in this hospital do you take care of cleanliness?<br>Only in the SNCU complete area dusting I have to manage
2.6	What media do you use for cleaning many types of goods?<br>We use to use Duster, surf, scrubber etc.I do daily in the morning and sometimes I use to do dusting of the equipments before going from the facility
2.7	What media do you use for cleaning many types of goods?<br>Wet cloth, surf, bleaching powder is the products that are used for cleaning and dusting.
2.8	Does anyone check your work??<br>Yes our supervisor he uses to do rounds for check.
**3.**	**Disposal**
3.1	How you are you dispose the waste?<br>I don't know about it as such work is done by the sweeper of the area.
3.2	Do you use color coding systems for waste disposal? Detailed<br>Yes we use to manage according to the color coding only.
3.3	There is any vehicle for pickup the waste?<br>Yes in a day 3 times vehicle to collect the waste use to come.
3.4	If yes, then how many times is it come in the hospital?<br>Daily 3 times
3.5	Do you burn the waste room? If so where<br>I don't know about it.
**4.**	**Issues/Suggestions**
4.1	tell us how other staff members treat you in hospital<br>That is always good I never faced any problem
4.2	tell us how patients treat you in the hospital<br>Patients relatives behavior is not good many a times. As I have to see the gate also due to which I had fights with the relatives.

4.3	tell us what difficulty you face during your work<br>There is nothing like that
4.4	Want to change the way you do for better service?<br>There should be some sitting area for the public as there is too much of crowding here.
**4.5**	What do you mean by quality?<br>It means there should be cleanliness and good care should be given
**4.6**	According to you, what are the issues affecting the quality of health services?<br>Here the biggest problem is relatives are coming inside the sncu even after telling them not to enter the SNCU. There is no guard here to manage people, how can I myself handle such crowd who are not listening to me.
**4.7**	What can you do to improve the quality of health services?<br>Here the first thing to be done is the availability of a regular guard who can manage the crowd. Rest of the things can be managed easily.
**4.8**	Have your relatives, friends or acquaintances ever taken advantage of the health services of this hospital? If not, any reason?<br>Yes they use to come, where else we will go.

ID: 2208101			
**1 Type of Health Facility fru 1, rewari**			**1.2 Designation: staff nurse**
**2. General**			
**2.1**	How long have you been working in this health facility? (months/years)- since may 2007		
**2.2**	Total months/years of service - 10 years		
**2.3**	What are your current roles and responsibility with respect to maternal and neonatal care?<br>After delivery first of all we do breast feeding, provide medicine to mother,we take care of the cleanliness, we see whether before delievery bed is clean or not, check the B.PP and temperature of the mother, after the delivery we assist moher for gurping the baby and keep the baby with the mother.		
**2.4**	How many deliveries and resuscitations of newborns have you attended in last 1 month?		
	No. of deliveries attended in last 1 month<br>I don't remember this much but around 10 deliveries I use to do		
	2.4.2 No. of newborn resuscitations attended in last 1 month<br>Last month only 1 after that we referred the child		
**2.5**	Who did you receive the training from:		

	Area	Training name	Year
A	Care during delivery (S.B.A.)	Induction training for 14 days	2015
B	Neonatal Resuscitation Program (In RP / NSS)	NSSK	2012
C	Sick Newborn Care (FBNC)	No I didn't had	

3. Service Delivery

3.1 What are the challenges faced by you and your colleagues for delivering the desired mother and newborn care services?

Challenges faced

	**Mother care**	**Newborn care**
Infrastructure	There is no challenges in this	
Equipment	Every equipments are available	Every equipments are available
Drugs and supplies	We get prior notification about it, so there is no shortage in it	
Support services	Only 1 sweeper is available in the morning, and no sweeper in the evening and night shift so what we do is as the sweeper is staying nearby we call him if any cleanliness is required	
Other	Overall there is no problem, but there is no LMO, Pediatrician or class iv which is very much required here	

3.2 What challenges do you face while delivering essential newborn care services and how do you manage these?

**Challenges faced**	**How do you manage these challenges**
Care at delivery	We arrange the labor room properly first like check the suctioning machine, labor bed, oxygen, radiant warmer such things we arrange first. Then we provide emotional support to the mother.
Care in the ward	There is a problem with attendant like they don't go from the patient bed side and start lying down on the vacant beds.
Care of sick newborns	

3.3 What challenges do you face while delivery of pregnant women?

**Challenges faced**	**How do you manage these challenges**
Delivery without complication	Sweeper is not available so after the delivery it takes time to clean up.

	Delivery with complication	We use to refer
	Caesarean section	We use to refer
	Referred cases with complication	I don't know about it
**3.4**	How long usually the mothers stay at the facility after the delivery?	
	Normal Delivery	48 hours
	Caesarean Delivery	
**4. Manpower**		
**4.1**	How many posts of Staff Nurse / ANM are vacant in your health facility?<br>dont know about the nurse	
**4.2**	What difficulties do you face in providing mother and newborn care services to existing employees? (Doctors, nurses and other staff)<br>I already told about this	
**4.3**	What is the mechanism of taking leave and who sanctions it?<br>Actually the senior nurses use to train them. Also if there is any training in rewari we use to send them<br>According to the new batches formed.	
**5. Duty Roster**		
**5.1**	Who prepares the duty roster for you?<br>Nursing sister	
**5.2**	Do you have flexibility in changing the shifts??<br>We use to adjust amongst us and inform to the nursing sister	
**5.3**	How do you manage when you have double shifts?<br>It comes very rarely like in the case of strike in NHM other than that there is no double shifts.	
**5.4**	What is the procedure for taking leave and who approves it?<br>We use to give an application to the nursing sister and she approves for the leave.	
**5.5**	Who prepares rosters for emergency / regular service?<br>There is no separate roster for emergency	
**6. Infrastructure**		
**6.1**	Do you have space to accommodate changes inside the department?<br>There is ample space available that you can even start a medical college here	
**6.2**	Do you have enough beds to accommodate increased number of patients?<br>Beds are not adequate we use to admit on 1 bed 2-3 patients sometimes.	
**6.3**	Is their regular power supply and clean water for drinking? Any substitute available in case of power cut or irregular water supply?<br>Here we have generator and invertor so we don't face any problem in this.	
**7. Data management**		
**7.1**	How do you record data?<br>We use to record the data on registers.	
**7.2**	How do you maintain a register?<br>Here we have separate registers, so we use to do entry according to it.	
**7.3**	Where do you send the record?<br>Nursing sister use to manage that, she give the record to data entry operator (sachin) and he use to do entry in his system.	
**7.4**	How often is the data sent?<br>She use to send the data on a monthly basis	
**8. Blood bank**		
**8.1**	How long does it take for a needy person to get blood?<br>Mostly we use to refer blood transfusion cases. We actually did blood transfusion here last year	

.

	once. In that case we send our ambulance to rewari from where we issued the blood as the mother was very weak and could not go for the district and ambulance was not available here at that time. It was brought here by using proper cold chain method and it took around 35 minutes to get blood here.	
**9. Training /Skills**		
**9.1**	Could you tell us about your previous NSSK / Neonatal Resuscitation Training?	
	Time (month / year)	2012
	place	Rewari
	Duration (in days)	2
**9.2**	Who conducts the workshop? Who prepares roster for workshop/training and how it is notified? How it is monitored?<br>the data entry operator use to keep such records	
**9.3**	What did you like the most in the training?<br>We got to learn many new things there.	
**9.4**	What did you dislike the most in the training?<br>The food they provided was very bad other than this everything was good.	
**9.5**	What was the training methodology used (Lectures/ Hands-on / Practical's)?<br>They provided trainings on the dummy and on the board	
**9.6**	How did you like meeting / interacting with the trainers?<br>It was good actually the trainer was our duty doctor itself so there was not much of a problem	
**9.7**	What was their level of knowledge / skills?	It was good
	How was your conversation with him	
**9.8**	What are the opportunities and mechanisms currently in place/adopted to retain the skills of Nurses/ANMs/Doctors?	
**9.9**	What challenges do you have with the skills of nurses and the support of staff in delivery rooms, perinatal wards and newborn care units? In your opinion, how can this be controlled?<br>Already told	
**9.10**	How the training related to care during delivery and newborn period can be further improved?<br>Everything same as told above I don't know what else new to say	
**9.11**	Have you been to a skill lab set up in your district?	
**9.12**	What are the good things about this skill lab?<br>Actually skill lab is only there in Faridabad and I never went there neither I got chance to go also	
**9.13**	What are the challenges related to skill lab?<br>NA	
**9.14**	In your opinion, how many health staffs might have used or visited the skill labs?<br>NA	
**9.15**	Did somebody advise or persuade you to attend the skill lab?<br>NA	
**9.16**	How does the Skill Lab help in Neonatal Resuscitation and Neonatal Care?<br>NA	
**10. Referral services**		
**10.1**	in what situation usually the newborns/mothers (pregnant/recently delivered) are referred to the next level of healthcare?<br>Here only normal cases are seen. If the mother is anemic or in case of ultrasound if there is any problem seen we don't take risk and we immediately refer to the higher facility	
**10.2**	Where the newborns/ pregnant women/ mothers are usually referred, what is the usual mode of transportation and how long does it takes to reach the next level health facility in your area?<br>From here we normally refer to DH rewari in the ambulance provided from this facility. From here it takes maximum 30 minutes.	

.

**10.3**	What facilitation is done from facility side for referral and what difficulties/challenges do you face while transporting the sick newborn and mother to next level? *(Probe: monetary/logistics)*<br>There is no such challenge the only problem that sometimes comes is if the ambulance is not available at that time for that they have to wait then for ambulance to come
**10.4**	What are the challenges faced related to referral transport experienced by this facility and how are they handled?<br>I never saw any such challenges which cause problem
**11. Logistics**	
**11.1**	Are you familiar with any scarcity / irregular supply of medicines and / or supplies required for care during delivery and newborn in the last one year? What were the reasons for this deficiency and how were these conditions managed?<br>Yes medicines are all written which are available here itself and even if it is not available then they have to buy medicine from outside which happens very rarely.
**11.2**	How frequently the families/ parents asked to procure drugs from outside/ store?<br>Very rarely they have to take medicine from outside mostly they get all medicine
**11.3**	What are the supervisory mechanisms in place at present for maternal and newborn care services?
	Who supervises<br>We only do take care of everything and doctor just prescribe what all medicines to be given
	What is the frequency of supervisory visits<br>Immediately when patient arrives in the facility
	Is any feedback/report provided usually after the supervision?<br>Yes doctor advice what all test to be done, then after seeing the result then he tells what to do further
	What actions are taken after last supervisory visit?<br>After seeing the blood report then he decide what to do further. If the patient condition is taken care in this facility itself then we admit here else we refer to the DH rewari.
**11.4**	Please let us know about the last supervisory visit to the facility related to maternal and newborn care services?
	Who came for last supervisory visit?<br>We only inform the doctor about the condition of the mother.
	How long ago the supervisory visit took place?<br>This is difficult to say as it differs patient to patient
	What all components were observed?<br>We use to check the blood pressure, hemoglobin and whether she is able to walk or not.
	What feedback was given and what actions were taken?<br>After seeing then we decide to for discharging or if there is no signs of improvement we refer to the DH rewari
**12. others**	
**12.1**	How wide do the events in discharge / record slip?<br>In that only Blood pressure, pulses, HB are mentioned.
**12.2**	In your view, what are the perceived barriers between families in the use of public health services for newborns of pregnant women?<br>Here the biggest problem is the unavailability of 1 pediatrician, LMO as there is very risky in referring the serious cases to the higher facility<br>How can these obstacles be overcome?<br>Atleast the specialist doctor should be available during the morning shift
**12.3**	How much additional effort is needed to reduce the neonatal mortality rate in your area?<br>Like I already said earlier here facilities should be developed, doctors should be given here so that we can take care here itself without the need of referring.

**12.4**	According to you, what is the meaning of quality?
**12.5**	According to you, what are the issues affecting the quality (quality) of health services?<br>Meaning of quality is providing good care , cleanliness in the labor room or ward these all are quality.
**12.6**	What can you do to improve the quality of the health services?<br>Here as I told earlier there is a need for the staff like pediatrician, LMO, class 4, sweeper etc other than this we are managing somehow
**12.7**	Did any of your relatives, friends or acquaintances ever availed health services at this hospital? If not, any reason?<br>We should get staff here then the rest of the things will be improved automatically.

.

ID: 2208102		
**1 Type of Health Facility fru 1, rewari**		**1.2 Designation: staff nurse**
**2. General**		
**2.1**	How long have you been working in this health facility? (months/years)- since 2008	
**2.2**	Total months/years of service- Total 9 years of work	
**2.3**	What are your current roles and responsibility with respect to maternal and neonatal care?<br>What should I say, whatever you are seeing here like complete responsibility of mother and newborn. Our responsibility is starting from the time of delivery till the birth of child.	
**2.4**	How many deliveries and resuscitations of newborns have you attended in last 1 month?	
	2.4.1 No. of deliveries attended in last 1 month<br>In a month sometimes 10-15	
	2.4.2 No. of newborn resuscitations attended in last 1 month<br>Actually there are different staff for this from NBSU but still I have done 2-3.	
**2.5**	Who did you receive the training from:	

	Area	Training name	Year
A	Care during delivery (S.B.A.)	SBA	2013
B	Neonatal Resuscitation Program (In RP / NSS)	nil	
C	Sick Newborn Care (FBNC)	nil	

3. Service Delivery

3.1 What are the challenges faced by you and your colleagues for delivering the desired mother and newborn care services?

Challenges faced		
	**Mother care**	**Newborn care**
Infrastructure	No till now I didn't face any problem in this	
Equipment	Yes we have less equipments due to which we use to refer the mother	
Drugs and supplies	There is no challenges in this till now. Actually I don't know much about this	
Support services	Yes we are facing staff shortage I this, like unavailability of sweeper. Due to which cleaning is not done sometimes.	
Other	Yes there is no system here . we need to look after all the general cases as well as the delivery cases.	

3.2 What challenges do you face while delivering essential newborn care services and how do you manage these?

Challenges faced	How do you manage these challenges
Care at delivery	Actually we use to recommend the mother and relatives to stay after delivery for 2 days, if they are staying then we provide medicine and other care. But in most of the cases they insist in leaving the facility next day itself
Care in the ward	No problem in providing care in the ward
Care of sick newborns	We don't provide any care here as no doctor is available

3.3 What challenges do you face while delivery of pregnant women?

Challenges faced	How do you manage these challenges
Delivery without complication	There are no LMO available in the CHC so even for a minute problem we use to refer the mother and due to which we are unavailable to learn anything new.
Delivery with	All cases are referred

	complication	
	Caesarean section	All cases are referred
	Referred cases with complication	Don’t have any challenges as we use to refer and ambulance takes them.
**3.4**	How long usually the mothers stay at the facility after the delivery?	
	Normal Delivery	48 hours
	Caesarean Delivery	NA
**4. Manpower**		
**4.1**	How many posts of Staff Nurse / ANM are vacant in your health facility?<br>Don’t know about this.	
**4.2**	What difficulties do you face in providing mother and newborn care services to existing employees? (Doctors, nurses and other staff)<br>NR.	
**4.3**	What is the mechanism of taking leave and who sanctions it?<br>Yes we only use to train them by giving them duty on the morning hours	
**5. Duty Roster**		
**5.1**	Who prepares the duty roster for you?<br>Nursing sister	
**5.2**	Do you have flexibility in changing the shifts?<br>Yes it can be done, we use to adjust amongst ourselves	
**5.3**	How do you manage when you have double shifts?<br>There are no double shifts only night shift is considered as double shift	
**5.4**	What is the procedure for taking leave and who approves it?<br>Nursing sister use to provide us leave	
**5.5**	Who prepares rosters for emergency / regular service?<br>NA	
**6. Infrastructure**		
**6.1**	Do you have space to accommodate changes inside the department?<br>Yes we have lot of space here, so much that a new building can also be built here.	
**6.2**	Do you have enough beds to accommodate increased number of patients?<br>No, usually we never have so many patients here, even if patients come here in excess then we admit like 2-3 patient on 1 bed.	
**6.3**	Is their regular power supply and clean water for drinking? Any substitute available in case of power cut or irregular water supply?<br>Yes there is no issue in this	
**7. Data management**		
**7.1**	How do you record data?<br>We use to write it on the register	
**7.2**	How do you maintain a register?	
**7.3**	Where do you send the record?<br>Nursing sister use to send the monthly report to the SMO office.	
**7.4**	How often is the data sent??<br>Monthly reports are made	
**8. Blood bank**		
**8.1**	How long does it take for a needy person to get blood?<br>We use to refer the blood transfusion to the higher facility	
**9. Training /Skills**		
**9.1**	Could you tell us about your previous NSSK / Neonatal Resuscitation Training?	
	Time (month / year)	

	place	
	Duration (in days)	
**9.2**	Who conducts the workshop? Who prepares roster for workshop/training and how it is notified? How it is monitored?<br>There is one staff in office he use to make all such records	
**9.3**	What did you like the most in the training?<br>Actually not remembering anything now as the training was done many years back. Training was good and we got to learn many new things	
**9.4**	What did you dislike the most in the training?<br>Not remembering anything like that now.	
**9.5**	What was the training methodology used (Lectures/ Hands-on / Practical's)?<br>They use to ask everyone one by one, gave written exam, they used projector also.	
**9.6**	How did you like meeting / interacting with the trainers?<br>No I didn't talk to anyone	
**9.7**	What was their level of knowledge / skills?	It was good
	How was your conversation with him	
**9.8**	What are the opportunities and mechanisms currently in place/adopted to retain the skills of Nurses/ANMs/Doctors?<br>There should be frequent training for nurses so that nurses can handle minor complications rather than referring to the higher facility	
**9.9**	What challenges do you have with the skills of nurses and the support of staff in delivery rooms, perinatal wards and newborn care units? In your opinion, how can this be controlled?<br>NA	
**9.10**	How the training related to care during delivery and newborn period can be further improved?<br>There should be training at least once in a 6 months	
**9.11**	Have you been to a skill lab set up in your district?<br>Don't know about it, actually here there is no skill lab. The only skill lab is there in Faridabad.	
**9.12**	What are the good things about this skill lab?<br>NA	
**9.13**	What are the challenges related to skill lab?<br>NA	
**9.14**	In your opinion, how many health staffs might have used or visited the skill labs?<br>NA	
**9.15**	Did somebody advise or persuade you to attend the skill lab?<br>NA	
**9.16**	How does the Skill Lab help in Neonatal Resuscitation and Neonatal Care?<br>NA	
**10. Referral services**		
**10.1**	In what situation usually the newborns/mothers (pregnant/recently delivered) are referred to the next level of healthcare?<br>Here only normal delivery cases are seen. If the mother is anemic or there is any complication in the ultrasound then the mother is immediately referred to the District hospital.	
**10.2**	Where the newborns/ pregnant women/ mothers are usually referred, what is the usual mode of transportation and how long does it takes to reach the next level health facility in your area?<br>We use to refer to the district hospital only	
**10.3**	What facilitation is done from facility side for referral and what difficulties/challenges do you face while transporting the sick newborn and mother to next level? *(Probe: monetary/logistics)*<br>No during my time I have never faced any such situation	
**10.4**	What are the challenges faced related to referral transport experienced by this facility and how are they handled?	

.

11. Logistics	
**11.1**	Are you familiar with any scarcity / irregular supply of medicines and / or supplies required for care during delivery and newborn in the last one year? What were the reasons for this deficiency and how were these conditions managed?<br>No never saw anything like that, even if it happens then we tell them to take medicine from outside which they buy also.
**11.2**	How frequently the families/ parents asked to procure drugs from outside/ store?<br>Very rarely anything like this happens here
**11.3**	What are the supervisory mechanisms in place at present for maternal and newborn care services?
	Who supervises<br>We only do whatever we have to do.
	What is the frequency of supervisory visits<br>Immediately we use to take care. We start the drip or any medicine as prescribed.
	Is any feedback/report provided usually after the supervision?<br>Yes for discharge we use to do like this only.
	What actions are taken after last supervisory visit?<br>We use to see whether the person need to be discharged or referred
**11.4**	Please let us know about the last supervisory visit to the facility related to maternal and newborn care services?
	Who came for last supervisory visit?<br>Inform MO about it.
	How long ago the supervisory visit took place?
	What all components were observed?<br>We use to see that whatever problem she was admitted here is improved or not.
	What feedback was given and what actions were taken?<br>We use to see whether the person need to be discharged or referred
**12. others**	
**12.1**	How wide do the events in discharge / record slip?<br>We use to rake in writing about the discharge from Doctor (MO)
**12.2**	In your view, what are the perceived barriers between families in the use of public health services for newborns of pregnant women?<br>How can these obstacles be overcome?
**12.3**	How much additional effort is needed to reduce the neonatal mortality rate in your area?<br>Training of staff nurses is very much important as there is no doctor available here, so if any case comes we use to refer them which ultimately take extra time. So if staff is well trained we can provide basic care so that we can save the life of mother and child.
**12.4**	According to you, what is the meaning of quality?<br>According to me quality means to provide good facility along with proper treatment and care to the patient.
**12.5**	According to you, what are the issues affecting the quality (quality) of health services?<br>The biggest problem here is the unavailability of child specialist and one LMO due to which we are referring the cases forward
**12.6**	What can you do to improve the quality of the health services?<br>The one thing is that we need doctor here<br>Second to provide training to the nursing staff due to which they could learn something new.
**12.7**	Did any of your relatives, friends or acquaintances ever availed health services at this hospital? If not, any reason?<br>Yes we use to provide only basic care here as limited facility is available

.

ID: 2208301	
**AREA fru 1, Rewari**	**DESIGNATION : class IV**
**S.No.**	**Questions**
**1.**	**General Information**
1.1	Are you an employee of the hospital or are you contracted for the same?<br>Sir I am not here as a permanent staff of the hospital I am here as a contractual basis appointed here by a contactor
1.2	How many classrooms are there in this hospital?<br>Presently I am the only one class 4 staff of the facility earlier there was one more but recently he got retired
**2.**	**Specific Information**
2.1	Please tell us about the duty of duty and how many people are present in the cross<br>Presently the duty is made according to the morning shift only.
2.2	Your responsibility is only of one department, of all the other departments of the hospital?<br>Everywhere I am the only staff available wherever I am called I go. I don't have any fixed duty.
2.3	Explain your responsibilities<br>My duty here is of SMO office duty, if any stock is to be brought, if any file is to be transferred. Sometimes I have to work as a washer man also, all the bed sheets here are washed by me only. I also have to tie the bandage for the patients.
2.4	Do you have a duty even during the night shift?<br>No currently class 4 staff duty are not given at night.
2.5	Tell us which places in this hospital do you take care of cleanliness?<br>For cleaning one sweeper is there. If in case sweeper is not available then nurse call me to do cleaning as well, actually very rarely it happens like that. I am mostly doing only dusting work that too wherever necessary.
2.6	Tell us about your family's success?
2.7	What media do you use for cleaning many types of goods?<br>We use to use Duster, surf, scrubber etc.Wet cloth, surf, bleaching powder is the products that are used for cleaning and dusting.
2.8	Does anyone check your work?<br>No there is no one as such to monitor my work here.
**3.**	**Disposal**
3.1	How you are you dispose the waste?<br>I don't know about it.
3.2	Do you use color coding systems for waste disposal? Detailed<br>Yes it is made over here but I don't know much about it.
3.3	Is there any vehicle for pickup the waste?<br>Yes vehicle use to come and it collects waste from outside itself.
3.4	If yes, then how many times is it come in the hospital?<br>1-2 times daily hey use to come to collect waste.
3.5	Do you burn the waste room? If so where<br>No waste here are burned all the waste are dumped in the vehicle.
**4.**	**Issues/Suggestions**
4.1	tell us how other staff members treat you in hospital<br>It is good only. No one use to create problem here for me.
4.2	tell us how patients treat you in the hospital<br>It is ok only sometimes when relatives use to come shouting against the staff then I have to

	interrupt.
4.3	tell us what difficulty you face during your work<br>There are lots of problem here. Now you only tell me only 1 class 4 staff how much work will he do, sometime someone is calling me then some other time someone else. My salary is limited only neither it has increased but I am all time working only. If there is 2 more class 4 staff here then the work can be divided and no much burden will be there on one person. Now the complete night shift work is left pending for me in the morning when I come.
4.4	Want to change the way you do for better service?<br>The most necessary thing here is there should be more recruitment of the class 4 staff so that work can be divided amongst all equally. Now bedsheets are also washed by me. As new rule is passed that daily bedsheets has to be changed according to the color due to which I have to wash daily
**4.5**	What do you mean by quality?<br>Don't know
**4.6**	According to you, what are the issues affecting the quality of health services?<br>Staff is limited.
**4.7**	What can you do to improve the quality of health services?<br>Recruiting of the staff
**4.8**	Have your relatives, friends or acquaintances ever taken advantage of the health services of this hospital? If not, any reason?<br>Yes they use to come.

ID: 2308101			
**1 Type of Health Facility : fru 2, rewari**			**1.2 Designation: staff nurse**
**2. General**			
**2.1**	How long have you been working in this health facility? (months/years)- since 3/7/2014		
**2.2**	Total months/years of service- 3 years		
**2.3**	What are your current roles and responsibility with respect to maternal and neonatal care?<br>My responsibilities here are in OPD, Casality, delivery and NBSU also		
**2.4**	How many deliveries and resuscitations of newborns have you attended in last 1 month?		
	2.4. No. of deliveries attended in last 1 month<br>In last 1 month I have assisted in 7 deliveries		
	2.4.2 No. of newborn resuscitations attended in last 1 month<br>I have done 7 newborn babies resuscitation after doing the delivery of the mother		
**2.5**	Who did you receive the training from:		

	Area	Training name	Year
A	Care during delivery (S.B.A.)	NA	
B	Neonatal Resuscitation Program<br>(In RP / NSS)	NSSK	2016
C	Sick Newborn Care (FBNC)	FBNC	2014

3. दिए Service Delivery

3.1 What are the challenges faced by you and your colleagues for delivering the desired mother and newborn care services?

Challenges faced		
	**Mother care**	**Newborn care**
Infrastructure		Here there is nothing for the NBSU to properly function
Equipment	Here what I feel is equipments are very less and sometimes the equipments are not properly sterilized	
Drugs and supplies	Here there are no challenges related to medicines as all the available medicines are only prescribed here.	
Support services	The primary problem of this facility is the unavailability of a lady doctor here in the facility. The second problem is the availability of at least 1 permanent doctor. Other problems here that we face are the unavailability of Class IV and Sweeper too.	
Other	Class IV is only available during the night shift there is no class IV for the day shifts.	

3.2 What challenges do you face while delivering essential newborn care services and how do you manage these?

**Challenges faced**	**How do you manage these challenges**
Care at delivery	Here food is not provided in the facility therefore no patients are admitted here to provide the daily care.
Care in the ward	Here food is not provided in the facility therefore no patients are admitted here to provide the daily care.
Care of sick newborns	Newborn are not admitted in the facility

3.3 What challenges do you face while delivery of pregnant women?

**Challenges faced**	**How do you manage these challenges**
Delivery without complication	Unavailability of a lady doctor
Delivery with complication	Referred

	Caesarean section	Referred
	Referred cases with complication	I don’t know about this
**3.4**	how long usually the mothers stay at the facility after the delivery?	
	Normal Delivery	According to the rule it is 48 hours but here the mother stays for only 4-5 hours after that they go back to their home.
	Caesarean Delivery	Referred
**4. Manpower**		
**4.1**	How many posts of Staff Nurse / ANM are vacant in your health facility?<br>I don’t know anything about this; you can ask SMO regarding this he can help you out.	
**4.2**	What difficulties do you face in providing mother and newborn care services to existing employees? (Doctors, nurses and other staff)<br>Here there is no doctor in the facility, at one shift only 1 nurse is posted who has to take care of all the work. There is no class IV and no sweeper; you only tell how we will adjust.	
**4.3**	What is the mechanism of taking leave and who sanctions it?<br>No there is nothing like that. If any new staff is appointed then she is given only morning shifts or sometimes with the senior staff.	
**5. Duty Roster**		
**5.1**	Who prepares the duty roster for you?<br>SMO sir is only making roster for us.	
**5.2**	Do you have flexibility in changing the shifts?<br>It can be adjusted but it can create several problems because after exchanging the duty if that person didn’t do the duty properly or leave the facility early then the letter is made in my name only not her. As duty is not exchanged on paper.	
**5.3**	How do you manage when you have double shifts?<br>There is no double shift duty other than night	
**5.4**	छु What is the procedure for taking leave and who approves it?<br>Taking a leave is a very difficult task. I have joined here 3 years back and I never got a single leave. Now on Saturday I require a day leave due to some urgent work, let’s see that I will get or not.	
**5.5**	Who prepares rosters for emergency / regular service?<br>SMO sir	
**6. Infrastructure**		
**6.1**	Do you have space to accommodate changes inside the department?<br>Space is more than enough here but staff is also required here to work.	
**6.2**	Do you have enough beds to accommodate increased number of patients?<br>Here very rarely patients come in excess whoever comes they go back after few hours. So that is not a problem over here.	
**6.3**	Is their regular power supply and clean water for drinking? Any substitute available in case of power cut or irregular water supply?<br>Electricity problem is too much over here. During night no bulb is working outside the facility every bulb is damaged. Due to such darkness it is very dangerous to work during the night shifts.	
**7. I Data management**		
**7.1**	How do you record data?<br>We do entry in register itself	
**7.2**	How do you maintain a register?<br>There is one permanent staff here she only does handle such registers	
**7.3**	Where do you send the record?<br>That she only can say as I don’t know about it much.	
**7.4**	How often is the data sent?<br>Don’t know	

**8. Blood bank**		
**8.1**	How long does it take for a needy person to get blood?<br>Here no cases of blood transfusion is managed all such cases are referred	
**9. Training /Skills**		
**9.1**	Could you tell us about your previous NSSK / Neonatal Resuscitation Training?	
	Time (month / year)	2012
	place	Rewari District hospital
	Duration (in days)	
**9.2**	Who conducts the workshop? Who prepares roster for workshop/training and how it is notified? How it is monitored?<br>SMO sir use to make all the staff rosters	
**9.3**	What did you like the most in the training?<br>Got to learn many new things in the training	
**9.4**	What did you dislike the most in the training?<br>I didn't felt anything like that	
**9.5**	What was the training methodology used (Lectures/ Hands-on / Practical's)?<br>Training was given on dummy and sir use to call one by one and makes us do the procedure on the dummy.	
**9.6**	How did you like meeting / interacting with the trainers?<br>It was good training was given by our facility doctor only.	
**9.7**	What was their level of knowledge / skills?	It was good
	How was your conversation with him	
**9.8**	What are the opportunities and mechanisms currently in place/adopted to retain the skills of Nurses/ANMs/Doctors?<br>Don't know	
**9.9**	What challenges do you have with the skills of nurses and the support of staff in delivery rooms, perinatal wards and newborn care units? In your opinion, how can this be controlled?<br>Here atleast there should be one LMO who can manage all the cases here	
**9.10**	How the training related to care during delivery and newborn period can be further improved?	
**9.11**	Have you been to a skill lab set up in your district?<br>I don't know about it	
**9.12**	What are the good things about this skill lab?<br>NA	
**9.13**	What are the challenges related to skill lab?<br>NA	
**9.14**	In your opinion, how many health staffs might have used or visited the skill labs?<br>NA	
**9.15**	Did somebody advise or persuade you to attend the skill lab?<br>NA	
**9.16**	How does the Skill Lab help in Neonatal Resuscitation and Neonatal Care?<br>NA	
**10. Referral services**		
**10.1**	In what situation usually the newborns/mothers (pregnant/recently delivered) are referred to the next level of healthcare?<br>Here only normal cases are taken care of, other than that even a minute problem patients are referred.	
**10.2**	Where the newborns/ pregnant women/ mothers are usually referred, what is the usual mode of transportation and how long does it takes to reach the next level health facility in your area?<br>We are referring to DH Rewari . We are from here in the ambulance. From here it takes 45 to 60 minutes to reach the referred facility	

.

**10.3**	What facilitation is done from facility side for referral and what difficulties/challenges do you face while transporting the sick newborn and mother to next level? *(Probe: monetary/logistics)*<br>His I don't know
**10.4**	What are the challenges faced related to referral transport experienced by this facility and how are they handled?<br>Don't know
**11. Logistics**	
**11.1**	Are you familiar with any scarcity / irregular supply of medicines and / or supplies required for care during delivery and newborn in the last one year? What were the reasons for this deficiency and how were these conditions managed?
**11.2**	How frequently the families/ parents asked to procure drugs from outside/ store?<br>Here that medicines are only prescribed that is available in the facility other than that no other medicines are prescribed
**11.3**	What are the supervisory mechanisms in place at present for maternal and newborn care services?
	Who supervises
	What is the frequency of supervisory visits
	Is any feedback/report provided usually after the supervision?
	What actions are taken after last supervisory visit?
**11.4**	Please let us know about the last supervisory visit to the facility related to maternal and newborn care services?
	Who came for last supervisory visit?
	How long ago the supervisory visit took place?
	What all components were observed?
	What feedback was given and what actions were taken?
**12. others**	
**12.1**	How wide do the events in discharge / record slip?
**12.2**	In your view, what are the perceived barriers between families in the use of public health services for newborns of pregnant women?<br>How can these obstacles be overcome?
**12.3**	How much additional effort is needed to reduce the neonatal mortality rate in your area?
**12.4**	According to you, what is the meaning of quality?<br>Don't know what quality is.
**12.5**	According to you, what are the issues affecting the quality (quality) of health services?<br>Here the biggest problem is there is no sweeper because of which here there is no cleanliness. There is one sweeper whose tantrums are so high that he is something big over here. Here outside grass started growing up due to which so many mosquitoes and animals are here at the facility, the condition here s worst and it is the beginning of rainy season still the condition is worst. Here you see so many space is there but no work is done on it.
**12.6**	What can you do to improve the quality of the health services?<br>If only these problems are sorted out then there we can see improvement.
**12.7**	Did any of your relatives, friends or acquaintances ever availed health services at this hospital? If not, any reason?<br>Only for medicines purpose they are using services here but other than that they use to go to DH Rewari as no doctor is available here.

ID: 3108101			
**1.1 Type of Health Facility: DH, JHAJJAR**		**1.2 Designation: Medical officer**	
**2. General**			
**2.1**	How long have you been working in this health facility? (months/years)		11/16
**2.2**	Total months/years of service		8 months
**2.3**	What are your current roles and responsibility with respect to maternal and neonatal care?<br>• Care in ward, ANC, FTP, emergency obs/gynae<br>• Entrying and filling case sheets<br>• Taking rounds in wards		
**2.4**	How many deliveries and resuscitations of newborns have you attended in last 1 month?		
	A. No. of deliveries attended in last 1 month	20	
	B. No. of newborn resuscitations attended in last 1 month	3-4	
**3. Service Delivery**			
**3.1**	In routine practice, which health staff performs the following services?		
	**Services**	**Staff performing the services**	
	Delivery without complication	S/N, Doctor	
	Delivery with complication/ high risk delivery	Doctor, S/N	
	Caesarean section	Doctor	
	Newborn care at birth	Doctor, S/N	
	Sick newborn care	Pediatrician	
	Breastfeeding support	Doctors	
**3.2**	What are the challenges faced by you and your colleagues for delivering the desired mother and newborn care services?		
	**Challenges faced**	**How do you manage these challenges**	
	Infrastructure It is full of challenges. There are so many deliveries in line and no trays are ready. Patients which are uninvestigated with anemia visit with labor pain and are treated just like that.		
	Equipment	If autoclaving is not possible, equipment are washed in bleaching solution and then used. Class 4 washes them and keeps it ready.	
	Drugs and supplies		
	Support services	Staff should be increased. Also there is a problem of security. Female security guard is required.	
	Other		
**3.3**	What challenges do you face while delivering essential newborn care services and how do you manage these?		
	**Challenges faced**	**How do you manage these challenges**	
	Care at delivery There are no specialists in evening and night. So if we don't know what to do, we refer the patients at night. Hb tests are required at night, but are not possible.		
	Care in the ward Beds are less in the wards. Ratio is far too less. Even the sheets are very dirty. They don't wash it properly. There is no ventilation in the wards.		
	Care of sick newborns Pediatrician handles the cases. We don't interfere.		
**3.4**	What challenges do you face while delivery of pregnant women?		
	**Challenges faced**	**How do you manage these challenges**	
	Delivery without complication<br>If there is any tear we manage them ourselves.	Referred	

	But if we sense any risk, we refer the patient immediately.	
	Delivery with complication<br>Twins cases referred from PHCs, where the first is delivered normally and the second is breached. This is a major challenge.	We are obliged to refer them.
	Caesarean section	
	Referred cases with complication	It is difficult to convince them. They feel we could have referred them before hand, so it is bit challenging.
**3.5**	How long usually the mothers stay at the facility after the delivery?	
	Normal Delivery	48hrs
	Caesarean Delivery	4-8days

**4. Manpower**	
**4.1**	How many positions of doctors are lying vacant in your health facility?<br>There are very few.
**4.2**	If there is a shortage of manpower who addresses the issue so that is does not hinders routine work?<br>Doctor, class 4 and staff are there. At night class 4 is not available. Their work is usually divided. Sweeper is available but still less.
**4.3**	Do you have adequate staff inside labor room, ANC clinic and SNCU's?<br>3-4 staffs are required. They oversee duty at LR, ANC and ward and are required there. So it is a burden on them.
**4.4**	What happens if a particular department is having more flow of patients? Is there any flexibility in assigning inter departmental responsibilities among staff?<br>Class 4 help in deliveries. There is too much burden then.
**4.5**	What is the mechanism of taking leave and who sanctions it?<br>We don't take leaves. We don't get leaves. We adjust among ourselves.
**5. Duty Roster**	
**5.1**	Who prepares the duty roster for you?<br>Administrator does it for us.
**5.2**	Who follows up the prepared roster so that the shifts are routinely changed?<br>LR in charge does it.
**5.3**	How many Medical Officers are posted at one time in your department? What is the pattern of shift?<br>One in every shift. Morning and evening shifts from 8am to 5pm and 5pm to 8am respectively.
**5.4**	Do you have flexibility in changing the shifts?<br>Yes. It is adjustable.
**5.5**	How do you manage when you have double shifts?<br>Nobody tells us to do double shifts, but rather we choose to do it so that we can get extra off.
**6. Infrastructure**	
**6.1**	Do you have space to accommodate changes inside the department?<br>Doctor's room.
**6.2**	Do you have enough beds to accommodate increased number of patients?<br>We accommodate 2-3 in one bed. It is quite common.
**6.3**	Is their regular power supply and clean water for drinking? Any substitute available in case of power cut or irregular water supply?<br>It is irregular. We make use of generator. We bring drinking water from home. There is no separate cooler as such.
**7. Training /Skills**	
**7.1**	How many of the total staffs are trained for MCH services?<br>Everyone is not trained. 70% of them are trained in SBA. But not trained in IMNCI though.

**7.2**	Is there any pre job posting training for newly joined staff?<br>Nothing as such.	
**7.3**	Is there any on- job training for the staff?<br>We join first, and then we do it after joining here. There is induction, but nobody attends. So we left the second day itself.	
**7.4**	Please let us know about the last training on attended by you?<br>Induction training, sanitation practices and FMNCI	
	Timing (Month/Year)	2016
	Place	Jhajjar
	Duration (in days)	1day
	What did you like the most in the training?	
	What did you dislike the most in the training?<br>Not trained regarding delivery.	
	What was the training methodology used (Lectures/ Hands-on / Practical's)?<br>There were more of lectures and Power point slides. and less of practical. They used to teach on dummies.	
	Who conducts the workshop? Who prepares roster for workshop/training and how it is notified? How it is monitored?<br>DTO prepares the roster. BDO oversees whether the trainees attend and the lectures takes place or not.	
**7.5**	What are the opportunities and mechanisms currently in place/adopted to retain the skills of Nurses/ANMs/Doctors?	
**7.6**	How the training related to care during delivery and newborn period can be further improved?	
**7.7**	Have you ever visited/attended the skill labs operational in your district? Where it was conducted? Who conducted it? What was the time duration of skill lab training?<br>There are no skill labs here. There is one at Rohtak. Doctors are mostly sent to Panchkula or here itself at Jhajjar GH. Otherwise we train ourselves.	
**7.8**	What are the good things about this skill lab?	
**7.9**	What are the challenges related to skill lab?	
**7.10**	In your opinion, how many health staffs might have used or visited the skill labs?<br>None	
**7.11**	Did somebody advise or persuade you to attend the skill lab?<br>No	
**8. Referral services**		
**8.1**	In what situation usually the newborns/mothers (pregnant/recently delivered) are referred to the next level of healthcare?<br>We send them to Rohtak PGI. But it depends on them where they want to go. Ambulance is not available at all times.	
**8.2**	Where the newborns/ pregnant women/ mothers are usually referred, what is the usual mode of transportation and how long does it takes to reach the next level health facility in your area?<br>PGI Rohtak is 30 mins away from here.	
**8.3**	What facilitation is done from facility side for referral and what difficulties/challenges do you face while transporting the sick newborn and mother to next level? *(Probe: monetary/logistics)*<br>It is difficult to convince the patients and their attendants for referral. EMT and ambulance fall short at night when there are 3-4 referrals.	
**8.4**	What are the challenges faced related to referral transport experienced by this facility and how are they handled?<br>Equipment in ambulance is not even clean. The EMTs are not even trained. S/N accompanies the patient if required.	
**9. Logistics**		
**9.1**	Are you aware of any shortage/irregular supply of drugs and/or supplies needed for care during	

	delivery and newborn period in the last one year? What were the reasons for this shortage and how these situations were managed?<br>If not available anywhere, we purchase from outside. Gloves got over recently; we had to purchase it from outside. 3 pairs are required per patient.
**9.2**	How frequently the families/ parents asked to procure drugs from outside/ store?<br>We ask the patient to buy from outside. Once in a month some or the other drug falls short. S/N is responsible for keeping an eye over the supply. But if she fails to do so, drugs are bought from outside.
**9.3**	How many equipments essential for management of delivery or newborn care are out of order at this moment?<br>There are very few as such. Nonfunctional keep lying around. BP apparatus gets faulty usually. Thermometer put under armpit is used for different patient in their mouths. they don't even clean it with spirit. We usually remind them to do it.
**9.4**	What is the usual mechanism of repair and maintenance of these equipments? *(probe: who is responsible and what is the duration of repair*)<br>We ask Nursing I/C, she replaces it but it takes lot of time.
**9.5**	What are the supervisory mechanisms in place at present for maternal and newborn care services?
	Who supervises<br>MS, Dr Kanika on duty staff. There are charge registers. They keep an eye on that. We supervise on them.
	What is the frequency of supervisory visits<br>Daily routine
	Is any feedback/report provided usually after the supervision?<br>No feedbacks
	What actions are taken after last supervisory visit?<br>Letters are given daily, but it is usually for the students or someone who is new. They only come to check whether S/N is on duty or not.
**9.6**	Please let us know about the last supervisory visit to the facility related to maternal and newborn care services?
	Who came for last supervisory visit?
	How long ago the supervisory visit took place?
	What all components were observed?
	What feedback was given and what actions were taken?
**10. Perceptions regarding Quality of care**	
**10.1**	According to you, what is the meaning of quality?<br>Job satisfaction
**10.2**	According to you, what are the issues that affect the quality of health services?<br>Lack of interest now, feels misrepresented. Even the class 4 don't listen
**10.3**	What can you do to improve the quality of the health services?<br>Nothing would change on our say
**10.4**	Did any of your relatives, friends or acquaintances ever availed health services at this hospital? If not, any reason?<br>Yes, Mother and father do come to visit doctors here. Drugs and tests we do it from outside.
**11. Others**	
**11.1**	If any shortage of blood and how is it tackled?<br>Referred in severe cases
**11.2**	Do you arrange blood donation camps on facility basis?<br>Yes they do happen

ID: 3108102		
**1.1 Type of Health Facility: DH, JAHAJJAR**		**1.2 Designation: MO**

2. General

**2.1**	How long have you been working in this health facility? (months/years)	07/14
**2.2**	Total months/years of service	3 years

2.3 What are your current roles and responsibility with respect to maternal and neonatal care?

- Patient Care- Delivery, OPD, Ward, counseling parents, counseling for defects
- OPD register, case sheets completion, follow up card, checkups.

2.4 How many deliveries and resuscitations of newborns have you attended in last 1 month?

A. No. of deliveries attended in last 1 month	30-35
B. No. of newborn resuscitations attended in last 1 month	15

3. Service Delivery

3.1 In routine practice, which health staff performs the following services?

Services	Staff performing the services
Delivery without complication	Staff, on call doctor
Delivery with complication/ high risk delivery	Paed/Doctor or on-call
Caesarean section	Paed/Doctors
Newborn care at birth	Staff, Paed/Dr SNCU on call
Sick newborn care	Paed/Doctor, Staff
Breastfeeding support	Counselors, staff, doctors

3.2 What are the challenges faced by you and your colleagues for delivering the desired mother and newborn care services?

Challenges faced	How do you manage these challenges
Infrastructure Pigeon nest in False ceiling, MO room AC not functional, Inborn/Out born not separate	Complained but no action taken yet.
Equipment CPAP, Ventilator not available	Patients are referred
Drugs and supplies Antibiotics not available Normal drugs are present. So to clear stock they ask us to clear them. So we ask them to buy from outside as there is variety, in case of emergency.	
Support services Culture sensitivity is not done. There is an issue of security at night too.	Drugs are given blindly.
Other Disinfectant for cleaning purposes are not supplied within 15days	They just give 1 litre. There should be a specific amount to it.

3.3 What challenges do you face while delivering essential newborn care services and how do you manage these?

Challenges faced	How do you manage these challenges
Care at delivery Staffs are trained on paper but are under performing. Their performance is not up to the mark.	
Care in the ward Room is not ventilated no temperature regulation. AC should be present there.	We are just making use of it without any change.
Care of sick newborns Staff cant resuscitate or even change cannula or apply ET tube	We perform these activities. They just support us.

3.4 What challenges do you face while delivery of pregnant women?

Challenges faced	How do you manage these challenges
Delivery without complication	

	Delivery with complication	
	Caesarean section	
	Referred cases with complication	
**3.5**	How long usually the mothers stay at the facility after the delivery?	
	Normal Delivery	
	Caesarean Delivery	
**4. Manpower**		
**4.1**	How many positions of doctors are lying vacant in your health facility?<br>There is shortage only when we take leave. Otherwise it is manageable	
**4.2**	If there is a shortage of manpower who addresses the issue so that is does not hinders routine work?<br>Only during leave.	
**4.3**	Do you have adequate staff inside labor room, ANC clinic and SNCU's?<br>Manageable	
**4.4**	What happens if a particular department is having more flow of patients? Is there any flexibility in assigning inter departmental responsibilities among staff?<br>Pediatrician does the rounds. But on call is not much available.	
**4.5**	What is the mechanism of taking leave and who sanctions it?<br>We adjust among ourselves	
**5. Duty Roster**		
**5.1**	Who prepares the duty roster for you?<br>Pediatrician, S/N in-charge	
**5.2**	Who follows up the prepared roster so that the shifts are routinely changed?<br>Evening, night and then off for the MO's and Pediatrician has morning shifts and weekly offs.	
**5.3**	How many Medical Officers are posted at one time in your department? What is the pattern of shift?<br>There is one always present in one shift	
**5.4**	Do you have flexibility in changing the shifts?<br>We manage ourselves	
**5.5**	How do you manage when you have double shifts?<br>We rest in the doctors room	
**6. Infrastructure**		
**6.1**	Do you have space to accommodate changes inside the department?<br>Doctor room	
**6.2**	Do you have enough beds to accommodate increased number of patients?	
**6.3**	Is their regular power supply and clean water for drinking? Any substitute available in case of power cut or irregular water supply?<br>There are power cuts. The generator does not take sufficient load. Even the warmer doesn't function during such power cuts in winter. There is no issue of drinking water as such.	
**7. Training /Skills**		
**7.1**	How many of the total staff are trained for MCH services?<br>Some staffs are not trained	
**7.2**	Is there any pre job posting training for newly joined staff?<br>Yes, there is.	
**7.3**	Is there any on- job training for the staff?<br>No, there is no such training.	
**7.4**	Please let us know about the last training on attended by you?<br>No, not even one training.	
	Timing (Month/Year)	
	Place	
	Duration (in days)	

	What did you like the most in the training?
	What did you dislike the most in the training?
	What was the training methodology used (Lectures/ Hands-on / Practical's)?
	Who conducts the workshop? Who prepares roster for workshop/training and how it is notified? How it is monitored?
**7.5**	What are the opportunities and mechanisms currently in place/adopted to retain the skills of Nurses/ANMs/Doctors?<br>Regular proper training should be conducted. If the trainees are not receptive, nothing can be done.
**7.6**	How the training related to care during delivery and newborn period can be further improved?<br>We can have training sessions here too.
**7.7**	Have you ever visited/attended the skill labs operational in your district? Where it was conducted? Who conducted it? What was the time duration of skill lab training?<br>There are such skill labs, but not here.
**7.8**	What are the good things about this skill lab?
**7.9**	What are the challenges related to skill lab?
**7.10**	In your opinion, how many health staffs might have used or visited the skill labs?
**7.11**	Did somebody advise or persuade you to attend the skill lab?
**8. Referral services**	
**8.1**	In what situation usually the newborns/mothers (pregnant/recently delivered) are referred to the next level of healthcare?<br>Respiratory distress, LBW, sepsis, congenital heart diseases, pediatric surgery
**8.2**	Where the newborns/ pregnant women/ mothers are usually referred, what is the usual mode of transportation and how long does it takes to reach the next level health facility in your area?<br>PGI, Rohtak along with Ambulance and EMT
**8.3**	What facilitation is done from facility side for referral and what difficulties/challenges do you face while transporting the sick newborn and mother to next level? *(Probe: monetary/logistics)*<br>No radiant warmer in ambulance, cotton wrapped temperature management, not upto the mark trained EMT. Ambulance for neonates not present. Equipment in ambulance not sterilized
**8.4**	What are the challenges faced related to referral transport experienced by this facility and how are they handled?<br>Equipment required for stabilization or any other are provided from here. Staff is required to be trained for emergency care.
**9. Logistics**	
**9.1**	Are you aware of any shortage/irregular supply of drugs and/or supplies needed for care during delivery and newborn period in the last one year? What were the reasons for this shortage and how these situations were managed?<br>Antibiotics are not cleared sometimes in order to clear the stocks.
**9.2**	How frequently the families/ parents asked to procure drugs from outside/ store?<br>Yes we do ask the sometimes to buy from outside
**9.3**	How many equipments essential for management of delivery or newborn care are out of order at this moment?<br>Equipment is under standard, not up to the mark. AC is not functional.
**9.4**	What is the usual mechanism of repair and maintenance of these equipments? *(probe: who is responsible and what is the duration of repair*)<br>Biomedical engineer is called for by sister I/C
**9.5**	What are the supervisory mechanisms in place at present for maternal and newborn care services?
	Who supervises<br>Pediatrician and MS sir takes rounds
	What is the frequency of supervisory visits<br>They visit frequently
	Is any feedback/report provided usually after the supervision?

	What actions are taken after last supervisory visit?
**9.6**	Please let us know about the last supervisory visit to the facility related to maternal and newborn care services?
	Who came for last supervisory visit?
	How long ago the supervisory visit took place?
	What all components were observed?
	What feedback was given and what actions were taken?
**10. Perceptions regarding Quality of care**	
**10.1**	According to you, what is the meaning of quality?<br>Equipment, services and performance should be used in best possible way and should be upto the mark/ standard. Babies should not suffer because of our facility.
**10.2**	According to you, what are the issues that affect the quality of health services?<br>Training is not sufficient, Staff requires training.
**10.3**	What can you do to improve the quality of the health services?<br>Training, standardized equipment, interactive training, home level care should be improved. Referral should be improved.
**10.4**	Did any of your relatives, friends or acquaintances ever availed health services at this hospital? If not, any reason?
**11. Others**	
**11.**	If any shortage of blood and how is it tackled?
**11.**	Do you arrange blood donation camps on facility basis?

ID: 3108103		
**1.1 Type of Health Facility: DH, JHAJJAR**	**1.2 Designation: MO**	

**2. General**		
**2.1**	How long have you been working in this health facility? (months/years)	April/2016
**2.2**	Total months/years of service	1 n half years
**2.3**	What are your current roles and responsibility with respect to maternal and neonatal care?<br>• FTP patients, CS, tubectomy, post-delivery patient related to their examination and care<br>• Extra duty for X-ray reporting	
**2.4**	How many deliveries and resuscitations of newborns have you attended in last 1 month?	
	A. No. of deliveries attended in last 1 month	50-60
	B. No. of newborn resuscitations attended in last 1 month	2

3. Service Delivery

3.1 In routine practice, which health staff performs the following services?

Services	Staff performing the services
Delivery without complication	S/N, MO
Delivery with complication/ high risk delivery	MO
Caesarean section	Gynae
Newborn care at birth	Paed, S/N, Mo
Sick newborn care	Paed
Breastfeeding support	MO, S/N

3.2 What are the challenges faced by you and your colleagues for delivering the desired mother and newborn care services?

Challenges faced	How do you manage these challenges
Infrastructure Tables for accommodating deliveries are less when deliveries are lined up	Accommodated in PV room
Equipment	
Drugs and supplies	
Support services	
Other	

3.3 What challenges do you face while delivering essential newborn care services and how do you manage these?

Challenges faced	How do you manage these challenges
Care at delivery Counseling of patients pre and post. It is difficult to communicate with patients sometimes. Sometimes we tie them before delivery.	
Care in the ward They don't stay for 48hrs	They request for discharge
Care of sick newborns Staff is not trained, they don't know about the equipments and methods	It then becomes the responsibility of the MO

3.4 What challenges do you face while delivery of pregnant women?

Challenges faced	How do you manage these challenges
Delivery without complication Patients are uncooperative, gets difficult to explain	
Delivery with complication Drugs for PPH, eclampsia are not available Staff is scared to handle such cases Staff is less at night and evening	referred<br>Sole responsibility
Caesarean section Doctor does the delivery, so hardly	

	any complications	
	Referred cases with complication	
**3.5**	How long usually the mothers stay at the facility after the delivery?	
	Normal Delivery	48hrs
	Caesarean Delivery	4-8days
**4. Manpower**		
**4.1**	How many positions of doctors are lying vacant in your health facility?<br>Don't know	
**4.2**	If there is a shortage of manpower who addresses the issue so that is does not hinders routine work?<br>In-charge does extended duty. if there is complication we call for her help.	
**4.3**	Do you have adequate staff inside labor room, ANC clinic and SNCU's?	
**4.4**	What happens if a particular department is having more flow of patients? Is there any flexibility in assigning inter departmental responsibilities among staff?<br>There is nothing of that sort. We manage on our own.	
**4.5**	What is the mechanism of taking leave and who sanctions it?<br>20 CLs are available. In-charge provides with sign and then MS.	
**5. Duty Roster**		
**5.1**	Who prepares the duty roster for you?<br>In-charge	
**5.2**	Who follows up the prepared roster so that the shifts are routinely changed?<br>We mark the attendance. In-charge keeps an eye.	
**5.3**	How many Medical Officers are posted at one time in your department? What is the pattern of shift?<br>Day and night shift. One MO in each shift. Gynae does the CS in the morning.	
**5.4**	Do you have flexibility in changing the shifts?<br>We do it among ourselves	
**5.5**	How do you manage when you have double shifts?<br>We do 2 shifts. We just wait here. We have even done 24hrs. There is no other option.	
**6. Infrastructure**		
**6.1**	Do you have space to accommodate changes inside the department?<br>Nothing as such.	
**6.2**	Do you have enough beds to accommodate increased number of patients?<br>We shift the patient with baby. We put double FTP patients on one bed if there is much load.	
**6.3**	Is their regular power supply and clean water for drinking? Any substitute available in case of power cut or irregular water supply?<br>It depends. Even if the light goes, we use the generator.	
**7. Training /Skills**		
**7.1**	How many of the total staff are trained for MCH services?<br>Mostly trained	
**7.2**	Is there any pre job posting training for newly joined staff?<br>Yes there is. But I haven't myself been to such trainings.	
**7.3**	Is there any on- job training for the staff?<br>No there is no such training	
**7.4**	Please let us know about the last training on attended by you?<br>SBA 2016	
	Timing (Month/Year)	2016
	Place	Jhajjar
	Duration (in days)	15days
	What did you like the most in the training?<br>Topics on resuscitation.	

	What did you dislike the most in the training?<br>There should be more of such trainings
	What was the training methodology used (Lectures/ Hands-on / Practical's)?<br>Practical
	Who conducts the workshop? Who prepares roster for workshop/training and how it is notified? How it is monitored?<br>Administrator decides on whom to send
**7.5**	What are the opportunities and mechanisms currently in place/adopted to retain the skills of Nurses/ANMs/Doctors?<br>We teach the staff topics on delivery and resuscitation in routine
**7.6**	How the training related to care during delivery and newborn period can be further improved?<br>We could teach and have proper training sessions here. We could also have refreshers
**7.7**	Have you ever visited/attended the skill labs operational in your district? Where it was conducted? Who conducted it? What was the time duration of skill lab training?<br>No trainings have done in the past
**7.8**	What are the good things about this skill lab?
**7.9**	What are the challenges related to skill lab?
**7.10**	In your opinion, how many health staffs might have used or visited the skill labs?<br>No clue
**7.11**	Did somebody advise or persuade you to attend the skill lab?<br>No
**8. Referral services**	
**8.1**	In what situation usually the newborns/mothers (pregnant/recently delivered) are referred to the next level of healthcare?<br>PGI Rohtak
**8.2**	Where the newborns/ pregnant women/ mothers are usually referred, what is the usual mode of transportation and how long does it takes to reach the next level health facility in your area?<br>It takes 1 hour for the ambulance with EMT to reach there
**8.3**	What facilitation is done from facility side for referral and what difficulties/challenges do you face while transporting the sick newborn and mother to next level? *(Probe: monetary/logistics)*<br>There is lack of availability of vehicle, EMT at the time of need. We are in touch with them regularly.
**8.4**	What are the challenges faced related to referral transport experienced by this facility and how are they handled?<br>We guide them and send
**9. Logistics**	
**9.1**	Are you aware of any shortage/irregular supply of drugs and/or supplies needed for care during delivery and newborn period in the last one year? What were the reasons for this shortage and how these situations were managed?<br>Rarely does it happen. We ask from the store or else we ask them to buy from outside.
**9.2**	How frequently the families/ parents asked to procure drugs from outside/ store?<br>We inform them before asking to purchase from outside
**9.3**	How many equipments essential for management of delivery or newborn care are out of order at this moment?<br>We regularly check on the equipment to see whether they function or not.
**9.4**	What is the usual mechanism of repair and maintenance of these equipments? *(probe: who is responsible and what is the duration of repair*)<br>We ask Sister I/C to change them. I don't know who comes to check.
**9.5**	What are the supervisory mechanisms in place at present for maternal and newborn care services?
	Who supervises<br>We supervise the staff. Otherwise MS sir does rounds
	What is the frequency of supervisory visits

	Regularly
	Is any feedback/report provided usually after the supervision?<br>There is no external supervision. It's only on the admin level.
	What actions are taken after last supervisory visit?<br>There is nothing as such.
**9.6**	Please let us know about the last supervisory visit to the facility related to maternal and newborn care services?
	Who came for last supervisory visit?
	How long ago the supervisory visit took place?
	What all components were observed?
	What feedback was given and what actions were taken?
**10. Perceptions regarding Quality of care**	
**10.1**	According to you, what is the meaning of quality?<br>Patient shouldn't be affected by the services that we provided
**10.2**	According to you, what are the issues that affect the quality of health services?<br>There is nothing as such but patient sometimes lands up with sepsis.
**10.3**	What can you do to improve the quality of the health services?<br>We could train the Class 4, sweeper as sterilization is very irregular
**10.4**	Did any of your relatives, friends or acquaintances ever availed health services at this hospital? If not, any reason?<br>Yes
**11. Others**	
**11.1**	If any shortage of blood and how is it tackled?<br>No
**11.2**	Do you arrange blood donation camps on facility basis?<br>Regularly

ID : 3108201		
**1 Type of Health Facility DH, JHAJJAR**		**1.2 Designation: STAFF NURSE**
**2. General**		

**2.1**	How long have you been working in this health facility? (months/years)<br>SNCU- 4 Months, GH Hospital 1 and half years
**2.2**	Total months/years of service -7/2/14
**2.3**	What are your current roles and responsibility with respect to maternal and neonatal care?<br>We take over first assess how sick the baby is, not feeding time of the baby. We do all of this in front of the baby, We check the equipment, general instruments and drugs for its availability<br>We do the bedding, Prepare the bleaching solution, We perform the rounds with the Doctor
**2.4**	How many deliveries and resuscitations of newborns have you attended in last 1 month?

A. No. of deliveries attended in last 1 month	
B. No. of newborn resuscitations attended in last 1 month	0

2.5 Who did you receive the training from:

	Area	Training name	**YEAR**
A	Care during delivery (S.B.A.)	Yes	2015
B	Neonatal Resuscitation Program (In RP / NSS)	no	
C	Sick Newborn Care (FBNC)	No	

3. Service Delivery

3.1 What are the challenges faced by you and your colleagues for delivering the desired mother and newborn care services?

Challenges faced

	**Mother care**	**Newborn care**
Infrastructure	There is AC in one inborn but not on the outborn. There is no proper fumigation. Light cuts are frequent. We hand over the babies to the mothers at night. There is back up but it just powers only the lights. The light cuts happen daily.	
Equipment		
Drugs and supplies	Drugs are available. If not then they are bought from outside.	
Support services		
Other	Doctor is available 24/7 in a private setup. But we have to call the doctors here at the time of emergency. Sometimes they do come, sometime they don't. We often clash with the parents. They catch hold of us.	

3.2 What challenges do you face while delivering essential newborn care services and how do you manage these?

**Challenges faced**	**How do you manage these challenges**
Care at delivery	There should be training for it. I haven't been trained. There is lot to learn.
Care in the ward	We learn stuff from here and there. Calculating fluids is a challenge for me. I have never performed resuscitation

	Care of sick newborns	We admit the patients without the Doctor being present. But we are not able to take decisions on our own. We have to wait for the Doctor.
**3.3**	What challenges do you face while delivery of pregnant women?	
	**Challenges faced**	**How do you manage these challenges**
	Delivery without complication	
	Delivery with complication	
	Caesarean section	
	Referred cases with complication	
**3.4**	How long usually the mothers stay at the facility after the delivery?	
	Normal Delivery	
	Caesarean Delivery	
**4. Manpower**		
**4.1**	How many posts of Staff Nurse / ANM are vacant in your health facility?	
**4.2**	What difficulties do you face in providing mother and newborn care services to existing employees? (Doctors, nurses and other staff)	
**4.3**	What is the mechanism of taking leave and who sanctions it?	
**5. Duty Roster**		
**5.1**	Who prepares the duty roster for you?<br>S/N IC prepares it first using a pencil so that we could adjust among ourselves.	
**5.2**	Do you have flexibility in changing the shifts?<br>Not always	
**5.3**	How do you manage when you have double shifts?<br>We do it as our responsibility.	
**5.4**	What is the procedure for taking leave and who approves it?<br>We have to inform SN IC and MS sir	
**5.5**	Who prepares rosters for emergency / regular service?<br>No	
**6. Infrastructure**		
**6.1**	Do you have space to accommodate changes inside the department?<br>Yes but the door is not fixed. We make one person stand outside.	
**6.2**	Do you have enough beds to accommodate increased number of patients?<br>Mothers from LR are kept in LR itself. Then we have one KMC room, we adjust the mothers there. Or else we keep them 2 in 1 bed.	
**6.3**	Is their regular power supply and clean water for drinking? Any substitute available in case of power cut or irregular water supply?<br>No. We had arranged for water camper but we shut that down. Light cuts are daily. even after informing Nurse I/C the problem hasn't solved.	
**7. Data management**		
**7.1**	How do you record data?<br>We fill in the columns in case sheet of the patient file.	
**7.2**	How do you maintain a register?<br>Then we enter it in the Indoor register. We put in all the details from the case sheet. Every S/N has charge of different registers for instruments to medicines	
**7.3**	Where do you send the record?<br>We give the case sheets to the data operator. They then fill it in the computer system.	
**7.4**	How often is the data sent?<br>Monthly reporting and daily discharges are to be filled.	
**8. blood bank**		

**8.1**	How long does it take for a needy person to get blood?<br>I don't know. Mothers require blood. 2-3hrs.	
**9. Training /Skills**		
**9.1**	Could you tell us about your previous NSSK / Neonatal Resuscitation Training?	
	Time (month / year)	
	place	
	Duration (in days)	
**9.2**	Who conducts the workshop? Who prepares roster for workshop/training and how it is notified? How it is monitored?	
**9.3**	What did you like the most in the training?<br>Provide support and counseling in feeding the baby	
**9.4**	What did you dislike the most in the training?<br>Inserting cannula. It is not our job. It is the job of Duty Dr.	
**9.5**	What was the training methodology used (Lectures/ Hands-on / Practical's)?	
**9.6**	What was the training methodology used (Lectures/ Hands-on / Practical's)?	
**9.7**	What was their level of knowledge / skills?	**9.7**
	How was your conversation with him	
**9.8**	What are the opportunities and mechanisms currently in place/adopted to retain the skills of Nurses/ANMs/Doctors?	
**9.9**	What challenges do you have with the skills of nurses and the support of staff in delivery rooms, perinatal wards and newborn care units? In your opinion, how can this be controlled?	
**9.10**	How the training related to care during delivery and newborn period can be further improved?	
**9.11**	Have you been to a skill lab set up in your district?	
**9.12**	What are the good things about this skill lab?	
**9.13**	What are the challenges related to skill lab?	
**9.14**	In your opinion, how many health staffs might have used or visited the skill labs?	
**9.15**	Did somebody advise or persuade you to attend the skill lab?	
**9.16**	How does the Skill Lab help in Neonatal Resuscitation and Neonatal Care?	
**10. Referral services**		
**10.1**	Where the newborns/ pregnant women/ mothers are usually referred, what is the usual mode of transportation and how long does it takes to reach the next level health facility in your area?<br>The baby that is the most sick. Doctors have their own 'thinking'. Some sent early itself, or some wait. They don't take up the babies that we send from here because they don't have enough space with them.	
**10.2**	Where the newborns/ pregnant women/ mothers are usually referred, what is the usual mode of transportation and how long does it takes to reach the next level health facility in your area?<br>We send them from here but they don't take them in there. Even I've accompanied the babies there. Here they just want to remove the sick babies from their responsibility	
**10.3**	What facilitation is done from facility side for referral and what difficulties/challenges do you face while transporting the sick newborn and mother to next level? *(Probe: monetary/logistics)*<br>We are not able to provide them ambulance on time. Migrant families take time to be sent from here or reach here. The EMT or the driver takes the baby only till the emergency. If there is ASHA along with them, they make sure the doctor sees the patient. It is not the EMTs job.	
**10.4**	What are the challenges faced related to referral transport experienced by this facility and how are they handled?<br>We should be able to treat and provide care for the baby here itself. And not send them anywhere	

.

	else. Or else if have to send the baby, then train the EMT before sending.
**11. Logistics**	
**11.1**	Are you familiar with any scarcity / irregular supply of medicines and / or supplies required for care during delivery and newborn in the last one year? What were the reasons for this deficiency and how were these conditions managed?<br>It is alright, it is available.
**11.2**	How frequently the families/ parents asked to procure drugs from outside/ store?<br>If there is no antibiotic we get it from outside.
**11.3**	What are the supervisory mechanisms in place at present for maternal and newborn care services?
	Who supervises<br>I don't know
	What is the frequency of supervisory visits
	Is any feedback/report provided usually after the supervision?
	What actions are taken after last supervisory visit?
**11.4**	Please let us know about the last supervisory visit to the facility related to maternal and newborn care services?
	Who came for last supervisory visit?
	How long ago the supervisory visit took place?
	What all components were observed?
	What feedback was given and what actions were taken?
**12. others**	
**12.1**	How wide do the events in discharge / record slip?<br>We write the name and other details of medicine etc, follow up details
**12.2**	In your view, what are the perceived barriers between families in the use of public health services for newborns of pregnant women?<br>If the baby is alright then there is no issue, but if the baby has some complication the parents get agitated. If there has to be any improvement, bringing the babies becomes an issue. The parents don't even know where the SNCU is, the roam around searching for the SNCU. If we want to send the babies for feed, we keep searching for the mother. There is a need for announcement system.<br>How can these obstacles be overcome?
**12.3**	How much additional effort is needed to reduce the neonatal mortality rate in your area?<br>The staff should be informed and trained, then only something can happen.
**12.4**	According to you, what is the meaning of quality?<br>Perfection
**12.5**	According to you, what are the issues affecting the quality (quality) of health services?<br>If we do anything wrong, we are not told or explained. If we do anything wrong, doctors should tell us what is to be done.
**12.6**	What can you do to improve the quality of the health services?<br>Send the staff for training
**12.7**	Did any of your relatives, friends or acquaintances ever availed health services at this hospital? If not, any reason?<br>We usually accompany them or else we don't ask them to go alone

.

ID: 3108202	
1 Type of Health Facility DH, JHAJAJR	1.2 Designation: STAFF NURSE

2. General

**2.1**	How long have you been working in this health facility? (months/years)- 7/2014
**2.2**	Total months/years of service- 3 years
**2.3**	What are your current roles and responsibility with respect to maternal and neonatal care?<br>Over of baby is taken and over is given to next sister and explain about the condition of the baby and file is made, Baby takes notes, takes Respiratory rate, Spo2, what doctor write we take the sample of the baby, Baby care bedding, Make file do entries, Counselling the mother, Nursing notes, treatment given to the baby, Counselling of how to feed the baby
**2.4**	How many deliveries and resuscitations of newborns have you attended in last 1 month?

A. No. of deliveries attended in last 1 month	
B. No. of newborn resuscitations attended in last 1 month	40-50

2.5 Who did you receive the training from: no training done

	Area	Training name	Year
A	Care during delivery (S.B.A.)		
B	Neonatal Resuscitation Program (In RP / NSS)		
C	Sick Newborn Care (FBNC)		

3. Service Delivery

3.1 What are the challenges faced by you and your colleagues for delivering the desired mother and newborn care services?

Challenges faced

	**Mother care**	**Newborn care**
Infrastructure		There is no resting room<br>There is no drinking water
Equipment		
Drugs and supplies		
Support services		No security guard<br>Attendant fight at night
Other		

3.2 What challenges do you face while delivering essential newborn care services and how do you manage these?

**Challenges faced**	**How do you manage these challenges**
Care at delivery	If Doctor was not available than we have heavy load than there problem in handling
Care in the ward	
Care of sick newborns	We have to take decision by our own to take care to baby

3.3 What challenges do you face while delivery of pregnant women?

**Challenges faced**	**How do you manage these challenges**
Delivery without complication	No
Delivery with complication	
Caesarean section	
Referred cases with complication	

3.4 How long usually the mothers stay at the facility after the delivery?

Normal Delivery	

	Caesarean Delivery	
**4. Manpower**		
**4.1**	How many posts of Staff Nurse / ANM are vacant in your health facility?<br>Don't know ask from administration	
**4.2**	What difficulties do you face in providing mother and newborn care services to existing employees? (Doctors, nurses and other staff)<br>We not able to give services because doctors are not available	
**4.3**	What is the mechanism of taking leave and who sanctions it?<br>All are trained	
**5. Duty Roster**		
**5.1**	Who prepares the duty roster for you?<br>Nursing sister do	
**5.2**	Do you have flexibility in changing the shifts?<br>Yes we get	
**5.3**	How do you manage when you have double shifts?<br>No problem in double shift. Two are there in one shift	
**5.4**	What is the procedure for taking leave and who approves it?<br>Nursing incharge, SNCU Incharge , MS	
**5.5**	Who prepares rosters for emergency / regular service?<br>SISTER KNOW	
**6. Infrastructure**		
**6.1**	Do you have space to accommodate changes inside the department?<br>No Sit outside and look after that	
**6.2**	Do you have enough beds to accommodate increased number of patients?<br>Yes we have a separate feeding room and pediatric ward are also there	
**6.3**	Is their regular power supply and clean water for drinking? Any substitute available in case of power cut or irregular water supply?<br>Light problem is all over district. We have generator but on that AC doesn't work. There is a problem in water we bring from home	
**7. Data management**		
**7.1**	How do you record data?<br>File is made after the admission of the baby then we do the entry and after discharge we keep the file in records	
**7.2**	How do you maintain a register?<br>Indoor register, SNCU, Diagnosis, baby details register entry charge given to staff nurses	
**7.3**	Where do you send the record?<br>Report is given to clerk she makes monthly	
**7.4**	How often is the data sent?<br>Monthly report	
**8. Blood bank**		
**8.1**	How long does it take for a needy person to get blood?	
**9. Training /Skills NO**		
**9.1**	Could you tell us about your previous NSSK / Neonatal Resuscitation Training?	
	Time (month / year)	
	place	
	Duration (in days)	
**9.2**	Who conducts the workshop? Who prepares roster for workshop/training and how it is notified? How it is monitored?	
**9.3**	What did you like the most in the training?	

.

**9.4**	What did you dislike the most in the training?	
**9.5**	what was the training methodology used (Lectures/ Hands-on / Practical's)?	
**9.6**	How did you like meeting / interacting with the trainers?	
**9.7**	What was their level of knowledge / skills?	**9.7**
	How was your conversation with him	
**9.8**	What are the opportunities and mechanisms currently in place/adopted to retain the skills of Nurses/ANMs/Doctors?	
**9.9**	What challenges do you have with the skills of nurses and the support of staff in delivery rooms, perinatal wards and newborn care units? In your opinion, how can this be controlled?	
**9.10**	How the training related to care during delivery and newborn period can be further improved?	
**9.11**	Have you been to a skill lab set up in your district?	
**9.12**	What are the good things about this skill lab?	
**9.13**	What are the challenges related to skill lab?	
**9.14**	In your opinion, how many health staffs might have used or visited the skill labs?	
**9.15**	Did somebody advise or persuade you to attend the skill lab?	
**9.16**	How does the Skill Lab help in Neonatal Resuscitation and Neonatal Care?	
**10. Referral services**		
**10.1**	Where the newborns/ pregnant women/ mothers are usually referred, what is the usual mode of transportation and how long does it takes to reach the next level health facility in your area?<br>Explain baby problem to their parents and give details to patient. And we send the baby in ambulance we drop the baby till the ambulance	
**10.2**	Where the newborns/ pregnant women/ mothers are usually referred, what is the usual mode of transportation and how long does it takes to reach the next level health facility in your area?<br>PGI Rohtak 30-40 min EMT, driver are there in the ambulance	
**10.3**	What facilitation is done from facility side for referral and what difficulties/challenges do you face while transporting the sick newborn and mother to next level? *(Probe: monetary/logistics)*<br>Ambulance doesn't have everything so we send the baby after stabilizing	
**10.4**	What are the challenges faced related to referral transport experienced by this facility and how are they handled?<br>Providing services in ambulance and EMT should be trained	
**11. Logistics**		
**11.1**	Are you familiar with any scarcity / irregular supply of medicines and / or supplies required for care during delivery and newborn in the last one year? What were the reasons for this deficiency and how were these conditions managed?<br>No it doesn't happen	
**11.2**	How frequently the families/ parents asked to procure drugs from outside/ store?	
**11.3**	What are the supervisory mechanisms in place at present for maternal and newborn care services?	
	Who supervises<br>NHM Team	
	What is the frequency of supervisory visits<br>MS Sir, SNCU incharge comes round anytime	
	Is any feedback/report provided usually after the supervision?<br>Reporting is done of everything	
	What actions are taken after last supervisory visit?<br>I don't know	
**11.4**	Please let us know about the last supervisory visit to the facility related to maternal and newborn care services?	

.

	Who came for last supervisory visit?
	How long ago the supervisory visit took place?
	What all components were observed?
	What feedback was given and what actions were taken?
**12. others**	
**12.1**	How wide do the events in discharge / record slip?
**12.2**	In your view, what are the perceived barriers between families in the use of public health services for newborns of pregnant women?<br>Medicine should be available and provide services we can do which able to do<br>How can these obstacles be overcome?
**12.3**	How much additional effort is needed to reduce the neonatal mortality rate in your area?<br>medicines
**12.4**	According to you, what is the meaning of quality?<br>Do their duty properly and discuss the problem and speak the problem and don't hide anything is quality
**12.5**	According to you, what are the issues affecting the quality (quality) of health services?<br>Having all things which can make the work easy but work load is more and behavior is not good
**12.6**	What can you do to improve the quality of the health services?<br>Don't know. all have their own thinking
**12.7**	Did any of your relatives, friends or acquaintances ever availed health services at this hospital? If not, any reason?

.

ID: 3108204

1 Type of Health Facility: DH, JHAJJAR **1.2 Designation: STAFF NURSE**

2. General

**2.1**	How long have you been working in this health facility? (months/years)- 7/11
**2.2**	Total months/years of service - 6 YEARS
**2.3**	What are your current roles and responsibility with respect to maternal and neonatal care?<br>Look after LABOR ROOM WARD, Receive new patient, Make file of patient, Post ward nurses 4 in morning, 2 evening, 2 night, Patient send to examination room having 2 table. Waiting room having 6 table sometime 2 patient adjust in one table, Look after waiting patient in every 4 hours
**2.4**	How many deliveries and resuscitations of newborns have you attended in last 1 month?

A. No. of deliveries attended in last 1 month	
B. No. of newborn resuscitations attended in last 1 month	

2.5 Who did you receive the training from:

	Area	Training name	Year
A	Care during delivery (S.B.A.)	DH, JHAJJAR. 21 DAYS	2014
B	Neonatal Resuscitation Program (In RP / NSS)		2016
C	Sick Newborn Care (FBNC)		

3. Service Delivery

3.1 What are the challenges faced by you and your colleagues for delivering the desired mother and newborn care services?

Challenges faced

	**Mother care**	**Newborn care**
Infrastructure	All things are good	
Equipment	Cooler is not working properly. Please provide A.C in LR and all things should work properly	
Drugs and supplies	Sometimes medicine are taken from outside if not available we inform M.S Sir. He provide to us	
Support services	Migrated people having problem of food. Milk is given to them is cold so heating the milk is a problem to patient and gas cylinder is finished	
Other	Patient said to admit them then file is made after that they went back without informing	

3.2 What challenges do you face while delivering essential newborn care services and how do you manage these?

**Challenges faced**	**How do you manage these challenges**
Care at delivery	
Care in the ward	2-3 hours wait in L.R if there are patient load if normal delivery then patient shifted to ward
Care of sick newborns	Pediatrician come on call. He was always available

3.3 What challenges do you face while delivery of pregnant women?

**Challenges faced**	**How do you manage these challenges**
Delivery without complication	One nurse is available for patient and if the baby is week than baby is send to SNCU with class4. Baby have to stay in L.R
Delivery with complication	Staff nurse support breastfeeding
Caesarean section	No problem
Referred cases with complication	

3.4 how long usually the mothers stay at the facility after the delivery?

Normal Delivery	
Caesarean Delivery	

4. Manpower

**4.1**	How many posts of Staff Nurse / ANM are vacant in your health facility?<br>12 staff nurse	
**4.2**	What difficulties do you face in providing mother and newborn care services to existing employees? (Doctors, nurses and other staff)<br>All staff is good behavior is also good	
**4.3**	What is the mechanism of taking leave and who sanctions it?<br>12 staff	
**5. Duty Roster**		
**5.1**	Who prepares the duty roster for you?	
**5.2**	Do you have flexibility in changing the shifts?	
**5.3**	How do you manage when you have double shifts?	
**5.4**	What is the procedure for taking leave and who approves it?	
**5.5**	Who prepares rosters for emergency / regular service?	
**6. Infrastructure**		
**6.1**	Do you have space to accommodate changes inside the department?	
**6.2**	Do you have enough beds to accommodate increased number of patients?	
**6.3**	Is their regular power supply and clean water for drinking? Any substitute available in case of power cut or irregular water supply?<br>Water cooler is there but we don't drink that water we bring from home. Upper tank lid is closed have to open	
**7. Data management**		
**7.1**	How do you record data?	
**7.2**	How do you maintain a register?	
**7.3**	Where do you send the record?	
**7.4**	How often is the data sent?	
**8. Blood bank**		
**8.1**	How long does it take for a needy person to get blood?	
**9. Training /Skills**		
**9.1**	Could you tell us about your previous NSSK / Neonatal Resuscitation Training?	
	Time (month / year)	2012
	place	
	Duration (in days)	
**9.2**	Who conducts the workshop? Who prepares roster for workshop/training and how it is notified? How it is monitored?	
**9.3**	What did you like the most in the training?	
**9.4**	What did you dislike the most in the training?	
**9.5**	what was the training methodology used (Lectures/ Hands-on / Practical's)?	
**9.6**	How did you like meeting / interacting with the trainers?	
**9.7**	What was their level of knowledge / skills?	
	How was your conversation with him	
**9.8**	What are the opportunities and mechanisms currently in place/adopted to retain the skills of Nurses/ANMs/Doctors?	
**9.9**	What challenges do you have with the skills of nurses and the support of staff in delivery rooms, perinatal wards and newborn care units? In your opinion, how can this be controlled?	
**9.10**	How the training related to care during delivery and newborn period can be further improved?	
**9.11**	Have you been to a skill lab set up in your district?	
**9.12**	What are the good things about this skill lab?	
**9.13**	What are the challenges related to skill lab?	
**9.14**	In your opinion, how many health staffs might have used or visited the skill labs?	

**9.15**	Did somebody advise or persuade you to attend the skill lab?
**9.16**	How does the Skill Lab help in Neonatal Resuscitation and Neonatal Care?
**10. Referral services**	
**10.1**	In what situation usually the newborns/mothers (pregnant/recently delivered) are referred to the next level of healthcare?<br>Refer is done after taking advice by Dr.
**10.2**	Where the newborns/ pregnant women/ mothers are usually referred, what is the usual mode of transportation and how long does it takes to reach the next level health facility in your area?
**10.3**	What facilitation is done from facility side for referral and what difficulties/challenges do you face while transporting the sick newborn and mother to next level? *(Probe: monetary/logistics)*
**10.4**	What are the challenges faced related to referral transport experienced by this facility and how are they handled?
**11. Logistics**	
**11.1**	Are you familiar with any scarcity / irregular supply of medicines and / or supplies required for care during delivery and newborn in the last one year? What were the reasons for this deficiency and how were these conditions managed?
**11.2**	How frequently the families/ parents asked to procure drugs from outside/ store?
**11.3**	What are the supervisory mechanisms in place at present for maternal and newborn care services?
	Who supervises
	What is the frequency of supervisory visits
	Is any feedback/report provided usually after the supervision?
	What actions are taken after last supervisory visit?
**11.4**	Please let us know about the last supervisory visit to the facility related to maternal and newborn care services?
	Who came for last supervisory visit?
	How long ago the supervisory visit took place?
	What all components were observed?
	What feedback was given and what actions were taken?
**12. others**	
**12.1**	How wide do the events in discharge / record slip?<br>Before referral medicine is given to the patient
**12.2**	In your view, what are the perceived barriers between families in the use of public health services for newborns of pregnant women?<br>Patient is motivated during waiting time<br>How can these obstacles be overcome?
**12.3**	How much additional effort is needed to reduce the neonatal mortality rate in your area??
**12.4**	According to you, what is the meaning of quality?<br>Patient comfortable and satisfaction. Patient don't want to refer we talk politely to the patient they are happy
**12.5**	According to you, what are the issues affecting the quality (quality) of health services?<br>Patient are sometimes rude so we not able to provide quality. 2-3 staff nurses are rude.<br>Charges are not taken for treatment from patients. Government hospital is good for treatment
**12.6**	What can you do to improve the quality of the health services?<br>Patient sometime says but we explain them properly
**12.7**	Did any of your relatives, friends or acquaintances ever availed health services at this hospital? If not, any reason?<br>Some known patient comes again but they don't come when we are not present

.

ID: 3108206			
**1 Type of Health Facility DH, JHAJJAR**		**1.2 Designation: STAFF NURSE**	
**2. General**			
**2.1**	How long have you been working in this health facility? (months/years)- 08/2010		
**2.2**	Total months/years of service - 7 Years		
**2.3**	What are your current roles and responsibility with respect to maternal and neonatal care?<br>Give care to patient, How to give birth, How to take care of patient and child Bedding , delivery work all work done by us, Arranging equipment, Delivery written work, Maintaining delivery register, birth register, entry, intro register, PPUICD Register and doing entry		
**2.4**	How many deliveries and resuscitations of newborns have you attended in last 1 month?		
	A. No. of deliveries attended in last 1 month	40	
	B. No. of newborn resuscitations attended in last 1 month	0	
**2.5**	Who did you receive the training from:		

	Area	Training name	Year
A	Care during delivery (S.B.A.)	y	2013
B	Neonatal Resuscitation Program (In RP / NSS)	y	2014
C	Sick Newborn Care (FBNC)	N	

3. Service Delivery

3.1 What are the challenges faced by you and your colleagues for delivering the desired mother and newborn care services?

Challenges faced		
	**Mother care**	**Newborn care**
Infrastructure	NO	
Equipment	ALL THINGS ARE AVILABLE	
Drugs and supplies	All things are there	
Support services	All things can be done	
Other		

3.2 What challenges do you face while delivering essential newborn care services and how do you manage these?

**Challenges faced**	**How do you manage these challenges**
Care at delivery	No sir
Care in the ward	no
Care of sick newborns	NO

3.3 What challenges do you face while delivery of pregnant women?

**Challenges faced**	**How do you manage these challenges**
Delivery without complication	NO sir, we do everything
Delivery with complication	
Caesarean section	In work nothing is les other staffs are there they handle that
Referred cases with complication	

3.4 How long usually the mothers stay at the facility after the delivery?

Normal Delivery	48 hrs
Caesarean Delivery	4-8 days

4. Manpower

4.1 How many posts of Staff Nurse / ANM are vacant in your health facility?

	Staff are accurate
**4.2**	What difficulties do you face in providing mother and newborn care services to existing employees? (Doctors, nurses and other staff)<br>All are trained
**4.3**	What is the mechanism of taking leave and who sanctions it?
**5. Duty Roster**	
**5.1**	Who prepares the duty roster for you?<br>Nursing sister incharge
**5.2**	Do you have flexibility in changing the shifts?<br>Adjusting can be done
**5.3**	How do you manage when you have double shifts?<br>No it doesn't happen
**5.4**	What is the procedure for taking leave and who approves it?<br>IF we have to take CL then we have to give the application and off is written in the roaster
**5.5**	Who prepares rosters for emergency / regular service?<br>Generally it adjust with the shift nurses
**6. Infrastructure**	
**6.1**	Do you have space to accommodate changes inside the department?<br>No, we do in the AC ROOM
**6.2**	Do you have enough beds to accommodate increased number of patients?<br>ADJUST 2 in one bed
**6.3**	Is their regular power supply and clean water for drinking? Any substitute available in case of power cut or irregular water supply?<br>No in LR and not for staff also. Water is always available for Handwashing
**7. Data management**	
**7.1**	How do you record data?<br>Case sheet
**7.2**	How do you maintain a register?<br>Indoor receiving register, delivery, baby entry, indoor aur birth register, diet register, PPIUCD, Birth forms
**7.3**	Where do you send the record?<br>Staff having charge. monthly report send by her and monthly report incharge changes every 6 months
**7.4**	How often is the data sent?<br>In 10-15 days we write the report
**8. Blood bank**	
**8.1**	How long does it take for a needy person to get blood?<br>1-11/2 hr
**9. Training /Skills**	
**9.1**	Could you tell us about your previous NSSK / Neonatal Resuscitation Training?
	Time (month / year): 2014
	place: Jhajjar
	Duration (in days): 2 days
**9.2**	Who conducts the workshop? Who prepares roster for workshop/training and how it is notified? How it is monitored?<br>Nursing sister incharge how having training adjusting done by didi
**9.3**	What did you like the most in the training?<br>We learn things from training
**9.4**	What did you dislike the most in the training?<br>No It doesn't happen

.

**9.5**	what was the training methodology used (Lectures/ Hands-on / Practical's)?<br>Practical	
**9.6**	How did you like meeting / interacting with the trainers?<br>good	
**9.7**	What was their level of knowledge / skills?	
	How was your conversation with him	nice
**9.8**	What are the opportunities and mechanisms currently in place/adopted to retain the skills of Nurses/ANMs/Doctors?<br>Any time in morning shift training should be given or decision should be will help in increasing knowlegde	
**9.9**	What challenges do you have with the skills of nurses and the support of staff in delivery rooms, perinatal wards and newborn care units? In your opinion, how can this be controlled?<br>All are trained. All do their work properly	
**9.10**	How the training related to care during delivery and newborn period can be further improved?	
**9.11**	Have you been to a skill lab set up in your district?<br>NO, WE DON'T KNOW	
**9.12**	What are the challenges related t skill lab?	
**9.13**	In your opinion, how many health staffs might have used or visited the skill labs?	
**9.14**	Did somebody advise or persuade you to attend the skill lab?	
**9.15**	How does the Skill Lab help in Neonatal Resuscitation and Neonatal Care?	
**9.16**	What are the challenges related to skill lab?	
**10. Referral services**		
**10.1**	In what situation usually the newborns/mothers (pregnant/recently delivered) are referred to the next level of healthcare?<br>Refer done by doctor slip made by doctor, class4 go with us till the ambulance	
**10.2**	Where the newborns/ pregnant women/ mothers are usually referred, what is the usual mode of transportation and how long does it takes to reach the next level health facility in your area?<br>PGI Rhotak 1 hr	
**10.3**	What facilitation is done from facility side for referral and what difficulties/challenges do you face while transporting the sick newborn and mother to next level? *(Probe: monetary/logistics)*<br>EMT is there in the ambulance we explain to them there is no problem	
**10.4**	What are the challenges faced related to referral transport experienced by this facility and how are they handled?<br>EMT is trained and they handle the patient	
**11. Logistics**		
**11.1**	Are you familiar with any scarcity / irregular supply of medicines and / or supplies required for care during delivery and newborn in the last one year? What were the reasons for this deficiency and how were these conditions managed?<br>It was on stock	
**11.2**	How frequently the families/ parents asked to procure drugs from outside/ store?	
**11.3**	What are the supervisory mechanisms in place at present for maternal and newborn care services?	
	Who supervises<br>Dr., MO do that	
	What is the frequency of supervisory visits	
	Is any feedback/report provided usually after the supervision?	
	What feedback was given and what actions were taken?<br>I don't know	

.

**11.4**	Please let us know about the last supervisory visit to the facility related to maternal and newborn care services?
	Who came for last supervisory visit?
	How long ago the supervisory visit took place?
	What all components were observed?
	What feedback was given and what actions were taken?
**12. others**	
**12.1**	How wide do the events in discharge / record slip?<br>Fallow up, give medicine and tell about breast feeding
**12.2**	In your view, what are the perceived barriers between families in the use of public health services for newborns of pregnant women?<br>How can these obstacles be overcome?
**12.3**	How much additional effort is needed to reduce the neonatal mortality rate in your area?
**12.4**	According to you, what is the meaning of quality?<br>Someone have different quality and someone do not have. good behavior
**12.5**	According to you, what are the issues affecting the quality (quality) of health services?
**12.6**	What can you do to improve the quality of the health services?
**12.7**	Did any of your relatives, friends or acquaintances ever availed health services at this hospital? If not, any reason?<br>Yes we tell others

.

ID 3108301	
AREA DH, jhajjar	DESIGNATION : class IV
**S.No.**	**Questions**
**1.**	**General Information**
1.1	Are you an employee of the hospital or are you contracted for the same?<br>From 2015 I am in SNCU. The post has removed for one year now. I am contracted as Class 4 and sweeper.
1.2	How many class 4 are there in this hospital?<br>5 C4 and sweeper Morning 2 evening 1 and night 1
**2.**	**Specific Information**
2.1	Please tell us about the duty of duty and how many people are present in the cross<br>We just have to look over here. We get changed a lot. but im here now, after 6 months we will be changed.
2.2	Your responsibility is only of one department, of all the other departments of the hospital?<br>Only in SNCU
2.3	Explain your responsibilities<br>Cleaning, dusting of warmer and other equipment<br>Receive and send baby for feed. Search for patients, doctors. Bring beds for Doctors. All the work here we have to do. Check on cylinders for O2. Bring the cylinders and change them. Make tea for everyone.
2.4	Do you have a duty even during the night shift?<br>Yes, right from tomorrow.
2.5	Tell us which places in this hospital do you take care of cleanliness?<br>Complete SNCU, KMC room, Doctors room, store room<br>Inborn/outborn and changing room
2.6	Tell us about your family's success?<br>Daily morning we do the cleaning. And every evenings only inside the SNCU. we do it shift wise.
2.7	What media do you use for cleaning many types of goods?<br>We use to use Duster, surf, scrubber etc.We put the solution in 2 liter water and mop the whole place. We clean the Ambu bag. We wash the walls using surf. Autoclave the instruments. Clean the warmers with sterilium or spirit. The bedsheets are cleaned in bleach water.
2.8	Does anyone check your work?<br>Nobody watches over. They are just happy with themselves.
**3.**	**Disposal**
3.1	How you are you dispose the waste?<br>We do it separately. We put it in a bowl inside the SNCU. Then we separate it outside.
3.2	Do you use color coding systems for waste disposal?<br>Detailed Red is for plastic. Yellow for blood, bandage, blood sugar stix, Black for normal waste, Blue is for glass.
3.3	There is any vehicle for pickup the waste?<br>Black bag is put in municipal dump waste. we also have to remove the waste.
3.4	If yes, then how many times is it came in the hospital?<br>They come daily or alternate day
3.5	Do you burn the waste room? If so where<br>No we don’t burn in hospital

**4.**	**Issues/Suggestions**
4.1	tell us how other staff members treat you in hospital<br>The supervisor doesn't speak with us clearly. We have to speak in front of all male staff. They even threaten to remove us. SN, doctors are good to us
4.2	tell us how patients treat you in the hospital<br>Some patients are good to us some are not. They come of all types. Some even drink and come
4.3	tell us what difficulty you face during your work<br>We go down to put our attendance. On a day of holiday we have to keep searching for the supervisor.
4.4	Want to change the way you do for better service?<br>If the baby is serious, we get the doctor from emergency. It has happened with me thrice now. But we can't report this. It had happened once during Dr. duty. We are scared of the parents. Dr had to come and refer the baby. We don't report this. They say that the baby was received as such to them. It is important to tell them.
**4.5**	What do you mean by quality?<br>There is nothing good here. 90% is of no use here. We tell them it would be good for them if they get it done from outside.
**4.6**	According to you, what are the issues affecting the quality of health services?<br>No it won't happen here. Everybody has their own perspective. Different answers. They can't work together.
**4.7**	What can you do to improve the quality of health services?<br>Everyone will have to adjust.
**4.8**	Have your relatives, friends or acquaintances ever taken advantage of the health services of this hospital? If not, any reason?<br>No

ID 3108302	
AREA DH, JHAJJAR DESIGNATION : class IV	
**S.No.**	**Questions**
**1.**	**General Information**
1.1	Are you an employee of the hospital or are you contracted for the same?<br>Contracted for 1year
1.2	How many class 4 are there in this hospital?<br>The contractor can tell that
**2.**	**Specific Information**
2.1	Please tell us about the duty of duty and how many people are present in the cross<br>There is 1 in every shift. 1 in the morning and evening There is no one in the night
2.2	Your responsibility is only of one department, of all the other departments of the hospital?<br>No. only here
2.3	Explain your responsibilities<br>Autoclave, do the sheets, clean equipment, walls, do the bedding. Change sheets and send for cleaning.
2.4	Do you have a duty even during the night shift?<br>Morning and evening. From tomorrow there would be night shift, let see how it goes. We get 4 offs every month.
2.5	Tell us which places in this hospital do you take care of cleanliness?<br>We only do the LR. I just look after here.
2.6	Tell us about your family's success?<br>Morning and evening once. Instruments are autoclaved after every delivery
2.7	What media do you use for cleaning many types of goods?<br>We use to use Duster, surf, scruber etc. There are different trays. if there are 2-4 deliveries then there is no issue. We prepare the bleaching solution
2.8	Does anyone check your work?<br>Nurse IC looks after us.
**3.**	**Disposal**
3.1	How you are you dispose the waste?<br>We put the waste in different dustbins. According to their color.
3.2	Do you use color coding systems for waste disposal? Detailed<br>Red- Plastic, Blue- Glass, Black- Paper and yellow- Placenta. We know this by looking at the posters
3.3	There is any vehicle for pickup the waste?<br>We put the black in the municipal bin and the rest is collected by BMW
3.4	If yes, then how many times is it come in the hospital?<br>That sweeper would tell
3.5	Do you burn the waste room? If so where<br>We burn the black sometimes
**4.**	**Issues/Suggestions**
4.1	tell us how other staff members treat you in hospital<br>It is alright
4.2	tell us how patients treat you in the hospital<br>They all are fine. Just there are few
4.3	tell us what difficulty you face during your work<br>We never used to understand before. But no we are trained.

4.4	Want to change the way you do for better service?<br>We want it to be good. Cleaning should take place at night. We even drop the patient till the ambulance
**4.5**	What do you mean by quality?<br>We can control the patient. They can reduce the way they dirty the place. We can make them understand. They throw the waste outside.
**4.6**	According to you, what are the issues affecting the quality of health services?<br>So we tell them, we tell them daily. We can have posters to remind them.
**4.7**	What can you do to improve the quality of health services?<br>C4 should be more
**4.8**	Have your relatives, friends or acquaintances ever taken advantage of the health services of this hospital? If not, any reason?<br>Yes we do bring them

ID: 3208101	
**1.1 Type of Health Facility: SUB-DH**	**1.2 Designation: MO**

2. General

**2.1**	How long have you been working in this health facility? (months/years)	7/2004
**2.2**	Total months/years of service	13years
**2.3**	What are your current roles and responsibility with respect to maternal and neonatal care?<br>Apart from obs and gynae, im also LR incharge, administrative and patient care duties. I supervise the nursing sister to make sure of availability and arrangements.	
**2.4**	How many deliveries and resuscitations of newborns have you attended in last 1 month?	
	A. No. of deliveries attended in last 1 month	70-80
	B. No. of newborn resuscitations attended in last 1 month	3-4

3. Service Delivery

3.1 In routine practice, which health staff performs the following services?

Services	Staff performing the services
Delivery without complication	All deliveries are supervised
Delivery with complication/ high risk delivery	Medical officer
Caesarean section	Specialist
Newborn care at birth	Pediatrician on call
Sick newborn care	Pediatrician on call
Breastfeeding support	S/N

3.2 What are the challenges faced by you and your colleagues for delivering the desired mother and newborn care services?

Challenges faced	How do you manage these challenges
Infrastructure First and foremost non availability of blood.	We have to refer the patient and if it gets complicated then it is our head ache.
Equipment Tocograph and CTG machine	
Drugs and supplies Drugs are usually available	
Support services Segregation becomes an issue while handling waste	
Other We used to have private wards but not operated as there is shortage of staff and security	

3.3 What challenges do you face while delivering essential newborn care services and how do you manage these?

Challenges faced	How do you manage these challenges
Care at delivery Shortage of doctors in evening. If there is a problem, I have to come.	
Care in the ward There is no storage unit for blood. Blood loves proves to be disastrous. Patients are either referred or blood is arranged from Jhajjar.	
Care of sick newborns ICU is required for raised BP or shock. These two things are important and round the clock it should be available.	

3.4 What challenges do you face while delivery of pregnant women?

Challenges faced	How do you manage these challenges
Delivery without complication	We speak with the seniors and civil surgeons to solve this issue.
Delivery with complication	Patients are usually referred in cases where blood might be required Sometimes if patient can wait, the attendant is sent to Jhajjar.
Caesarean section	

	Referred cases with complication	
**3.5**	How long usually the mothers stay at the facility after the delivery?	
	Normal Delivery	48hrs
	Caesarean Delivery	8days
**4. Manpower**		
**4.1**	How many positions of doctors are lying vacant in your health facility?<br>I'm not sure about that	
**4.2**	If there is a shortage of manpower who addresses the issue so that is does not hinders routine work?<br>Civil surgeon or seniors are informed about it.	
**4.3**	Do you have adequate staff inside labor room, ANC clinic and SNCU's?<br>Shortage of C4 and there is no security person. Attendants do get aggressive	
**4.4**	What happens if a particular department is having more flow of patients? Is there any flexibility in assigning inter departmental responsibilities among staff?<br>No, there is no such arrangement. We manage on our own.	
**4.5**	What is the mechanism of taking leave and who sanctions it?<br>My leave is the hardest to take	
**5. Duty Roster**		
**5.1**	Who prepares the duty roster for you?<br>I prepare it myself	
**5.2**	Who follows up the prepared roster so that the shifts are routinely changed?<br>I keep an eye, I make the changes	
**5.3**	How many Medical Officers are posted at one time in your department? What is the pattern of shift?<br>1 in the morning and 1 at night. The morning shift is till 4-5pm	
**5.4**	Do you have flexibility in changing the shifts?<br>Yes we manage on our own	
**5.5**	How do you manage when you have double shifts?<br>If possible I take compensatory off. Not compulsory. If there is someone to take cover duty. I stay locally so I manage but I never go home on duty.	
**6. Infrastructure**		
**6.1**	Do you have space to accommodate changes inside the department?<br>There is space for me but not sufficient for staff. There is no place for them to change.	
**6.2**	Do you have enough beds to accommodate increased number of patients?<br>Previously we had post op ward. Now it's not functional. So now we send to the surgical building. The building was made for ICU but not in use.	
**6.3**	Is their regular power supply and clean water for drinking? Any substitute available in case of power cut or irregular water supply?<br>Water electricity is adequate but there is shortage sometimes. We have back up for the LR.	
**7. Training /Skills**		
**7.1**	How many of the total staff are trained for MCH services?<br>It depends to person to person. S/N is very good otherwise.	
**7.2**	Is there any pre job posting training for newly joined staff?<br>Disinfection practices, handling of sterilized equipments. They do not know about it.	
**7.3**	Is there any on- job training for the staff?<br>They don't practice it sometimes.	
**7.4**	Please let us know about the last training on attended by you?	
	Timing (Month/Year)	
	Place	
	Duration (in days)	
	What did you like the most in the training?	

	What did you dislike the most in the training?
	What was the training methodology used (Lectures/ Hands-on / Practical's)?
	Who conducts the workshop? Who prepares roster for workshop/training and how it is notified? How it is monitored?
**7.5**	What are the opportunities and mechanisms currently in place/adopted to retain the skills of Nurses/ANMs/Doctors?
**7.6**	How the training related to care during delivery and newborn period can be further improved?
**7.7**	Have you ever visited/attended the skill labs operational in your district? Where it was conducted? Who conducted it? What was the time duration of skill lab training?<br>Im not aware about it.
**7.8**	What are the good things about this skill lab?
**7.9**	What are the challenges related to skill lab?
**7.10**	In your opinion, how many health staffs might have used or visited the skill labs?
**7.11**	Did somebody advise or persuade you to attend the skill lab?
**8. Referral services**	
**8.1**	In what situation usually the newborns/mothers (pregnant/recently delivered) are referred to the next level of healthcare?<br>Patient is first assessed and stabilized. If required in cases such as consult the patient and arrange for ambulance
**8.2**	Where the newborns/ pregnant women/ mothers are usually referred, what is the usual mode of transportation and how long does it takes to reach the next level health facility in your area?<br>PGI Rohtak- 45min to 1hr
**8.3**	What facilitation is done from facility side for referral and what difficulties/challenges do you face while transporting the sick newborn and mother to next level? *(Probe: monetary/logistics)*<br>EMT is present, actually SN or pharmacist but they are not available. Required staff is less
**8.4**	What are the challenges faced related to referral transport experienced by this facility and how are they handled?<br>We usually refer the sick patients and they tend to get sicker. Actually the ambulances are not well equipped. And the personnel is not trained. We usually stable the patient and require we send an S/N along.
**9. Logistics**	
**9.1**	Are you aware of any shortage/irregular supply of drugs and/or supplies needed for care during delivery and newborn period in the last one year? What were the reasons for this shortage and how these situations were managed?<br>Drug supply is not an issue
**9.2**	How frequently the families/ parents asked to procure drugs from outside/ store?<br>Everything is managed
**9.3**	How many equipments essential for management of delivery or newborn care are out of order at this moment?<br>Baby cart- light is not working. Since feb, there is no bulb or some other problem. Digital weighing machine doesn't work
**9.4**	What is the usual mechanism of repair and maintenance of these equipments? *(probe: who is responsible and what is the duration of repair*)<br>Nursing sister calls the biomedical engineer who covers the whole district. I'm not much aware
**9.5**	What are the supervisory mechanisms in place at present for maternal and newborn care services?
	Who supervises<br>Admin
	What is the frequency of supervisory visits<br>NHM team visits quarterly
	Is any feedback/report provided usually after the supervision?
	What actions are taken after last supervisory visit?

**9.6**	Please let us know about the last supervisory visit to the facility related to maternal and newborn care services?
	Who came for last supervisory visit?
	How long ago the supervisory visit took place?
	What all components were observed?
	What feedback was given and what actions were taken?
**10. Perceptions regarding Quality of care**	
**10.1**	According to you, what is the meaning of quality?<br>Perfection- bedding, disinfection, equipment sterilization or perfect work
**10.2**	According to you, what are the issues that affect the quality of health services?<br>Workload is an issue
**10.3**	What can you do to improve the quality of the health services?<br>Everyone should take responsibility to maintain quality. it is not and individual effort but teamwork
**10.4**	Did any of your relatives, friends or acquaintances ever availed health services at this hospital? If not, any reason?<br>Yes
**11. Others**	
**11.1**	If any shortage of blood and how is it tackled?<br>Jhajjar GH
**11.2**	Do you arrange blood donation camps on facility basis?<br>No

ID : 3208202				
**1 Type of Health Facility: DH, JHAJAJR**			**1.2 Designation: STAFF NURSE**	
**2. General**				
**2.1**	How long have you been working in this health facility? (months/years)- December 12			
**2.2**	Total months/years of service- 4 years			
**2.3**	What are your current roles and responsibility with respect to maternal and neonatal care?<br>Check instrument, Check SNCU baby, check tag on baby, check baby is empty stomach so give to mother for feeding. Giving routine care to baby. Shift change giving over to next nurse. Provide fluid to the patient. File is filled and keeps records in the file. After discharge it was submitted and give discharge slip to the patient. It was filled by doctor. Details written on the Discharge slip			
**2.4**	How many deliveries and resuscitations of newborns have you attended in last 1 month?			
	A. No. of deliveries attended in last 1 month			
	B. No. of newborn resuscitations attended in last 1 month			1-2
**2.5**	Who did you receive the training from:			

	Area	Training name	Year
A	Care during delivery (S.B.A.)		
B	Neonatal Resuscitation Program (In RP / NSS)	IMNCI	2012
C	Sick Newborn Care (FBNC)	FBNC	2/2014

3. Service Delivery

3.1 What are the challenges faced by you and your colleagues for delivering the desired mother and newborn care services?

Challenges faced		
	**Mother care**	**Newborn care**
Infrastructure		ok
Equipment		Equipment are available and working but doctors are not there so we not able to do anything
Drugs and supplies		Test-serum bilirubin, s.calcium not done here have to be done from outside PCM drop have to bring from outside
Support services		Doctors are not there than resuscitation medicine we can't give. We can give suction and if we require the doctor we call him and resuscitation done by us
Other		Cpap, ventilator not available baby is refer and SNCU was made but doctors should be there

3.2 What challenges do you face while delivering essential newborn care services and how do you manage these?

Challenges faced	How do you manage these challenges
Care at delivery	
Care in the ward	
Care of sick newborns	

3.3 What challenges do you face while delivery of pregnant women?

Challenges faced	How do you manage these challenges
Delivery without complication	
Delivery with complication	

	Caesarean section	We need doctors. We take decision by our own put cannula and advice taken from doctor on phone
	Referred cases with complication	
**3.4**	How long usually the mothers stay at the facility after the delivery?	
	Normal Delivery	
	Caesarean Delivery	
**4. Manpower**		
**4.1**	How many posts of Staff Nurse / ANM are vacant in your health facility?<br>2 nurse	
**4.2**	What difficulties do you face in providing mother and newborn care services to existing employees? (Doctors, nurses and other staff)<br>Security is less than there was problem in night class 4 were available but what they can do	
**4.3**	What is the mechanism of taking leave and who sanctions it?	
**5. Duty Roster**		
**5.1**	Who prepares the duty roster for you?<br>Nurse incharge do	
**5.2**	Do you have flexibility in changing the shifts?<br>yes sister do that	
**5.3**	How do you manage when you have double shifts?<br>Sometime it happens.	
**5.4**	What is the procedure for taking leave and who approves it?<br>Staff is always available. there is no problem of staff	
**5.5**	Who prepares rosters for emergency / regular service?<br>Weekly off is there and we have CL AND NURSE INCHARGE DO THAT	
**6. Infrastructure**		
**6.1**	Do you have space to accommodate changes inside the department?<br>yes	
**6.2**	Do you have enough beds to accommodate increased number of patients?<br>Beds for children bt for mother we have to adjust 2 in 1 bed. We keep inborn and outborn in one place	
**6.3**	Is their regular power supply and clean water for drinking? Any substitute available in case of power cut or irregular water supply?<br>Ro is there. Firstly water is not clean but now it was clean so we drink now	
**7. Data management**		
**7.1**	How do you record data?<br>Discharge slip given to the Patient and one copy we attach in the file. File entry done by us	
**7.2**	How do you maintain a register?<br>Address entry done in the Register. Entry of Age, details like admit date, time discharge and how many admitted done in indoor register	
**7.3**	Where do you send the record?<br>counsellor feed on the computer. Computer operator comes from jhajjar weekly she do the entry from the register	
**7.4**	How often is the data sent?<br>Daily we have to do. Daily entry monthly entry can be done	
**8. Blood bank**		
**8.1**	How long does it take for a needy person to get blood?	
**9. Training /Skills**		
**9.1**	Could you tell us about your previous NSSK / Neonatal Resuscitation Training?	
	Time (month / year)	2012
	place	jhajjar

.

	Duration (in days)	2 days
**9.2**	Who conducts the workshop? Who prepares roster for workshop/training and how it is notified? How it is monitored?<br>Name come from jhajjar but sister incharge send the nurse	
**9.3**	What did you like the most in the training?<br>BF method, 2-3 years of job now we know but in starting we don't know breastfeeding method we should be trained	
**9.4**	What did you dislike the most in the training?<br>NO IT WAS NOT	
**9.5**	What was the training methodology used (Lectures/ Hands-on / Practical's)?<br>Very less Hand on training BF method , counselling	
**9.6**	How did you like meeting / interacting with the trainers?<br>Not good not bad	
**9.7**	What was their level of knowledge / skills?	pediatrician
	How was your conversation with him	good
**9.8**	What are the opportunities and mechanisms currently in place/adopted to retain the skills of Nurses/ANMs/Doctors?<br>Training should be given	
**9.9**	What challenges do you have with the skills of nurses and the support of staff in delivery rooms, perinatal wards and newborn care units? In your opinion, how can this be controlled?	
**9.10**	How the training related to care during delivery and newborn period can be further improved?	
**9.11**	Have you been to a skill lab set up in your district?<br>NO, WE DON'T KNOW	
**9.12**	What are the challenges related to skill lab?	
**9.13**	In your opinion, how many health staffs might have used or visited the skill labs?	
**9.14**	Did somebody advise or persuade you to attend the skill lab?	
**9.15**	How does the Skill Lab help in Neonatal Resuscitation and Neonatal Care?	
**9.16**	What are the challenges related to skill lab?	
**10. Referral services**		
**10.1**	In what situation usually the newborns/mothers (pregnant/recently delivered) are referred to the next level of healthcare?<br>Rohtak PGI We can only make slip for PGI Rohtak but it depend on the attender where take the patient	
**10.2**	Where the newborns/ pregnant women/ mothers are usually referred, what is the usual mode of transportation and how long does it takes to reach the next level health facility in your area?<br>Ambulance reaches in 45mins	
**10.3**	What facilitation is done from facility side for referral and what difficulties/challenges do you face while transporting the sick newborn and mother to next level? *(Probe: monetary/logistics)*<br>We don't send patient directly first we stabilize the patient but after this if something happens to patient we don't know	
**10.4**	What are the challenges faced related to referral transport experienced by this facility and how are they handled?<br>EMT was available. If there was training we inform them. We arrange the ambulance we don't know further	
**11. Logistics**		
**11.1**	Are you familiar with any scarcity / irregular supply of medicines and / or supplies required for care during delivery and newborn in the last one year? What were the reasons for this deficiency and how were these conditions managed?<br>No	

.

**11.2**	How frequently the families/ parents asked to procure drugs from outside/ store?<br>Not done since one month
**11.3**	What are the supervisory mechanisms in place at present for maternal and newborn care services?
	Who supervises<br>Never done before. Daily done by nurse incharge
	What is the frequency of supervisory visits
	Is any feedback/report provided usually after the supervision?
	What actions are taken after last supervisory visit?
**11.4**	Please let us know about the last supervisory visit to the facility related to maternal and newborn care services?
	Who came for last supervisory visit?
	How long ago the supervisory visit took place?
	What all components were observed?
	What feedback was given and what actions were taken?
**12. others**	
**12.1**	How wide do the events in discharge / record slip?<br>All Details are given where delivery is done
**12.2**	In your view, what are the perceived barriers between families in the use of public health services for newborns of pregnant women?<br>How can these obstacles be overcome?
**12.3**	How much additional effort is needed to reduce the neonatal mortality rate in your area?
**12.4**	According to you, what is the meaning of quality?<br>Quality inside every person, care given by others and how care is given
**12.5**	According to you, what are the issues affecting the quality (quality) of health services?<br>Language should be good
**12.6**	What can you do to improve the quality of the health services?<br>By improving routine work
**12.7**	Did any of your relatives, friends or acquaintances ever availed health services at this hospital? If not, any reason?<br>NO.

.

ID: 3208203					
1 Type of Health Facility SDH, JHAJJAR				1.2 Designation: STAFF NURSE	
**2. General**					
**2.1**	How long have you been working in this health facility? (months/years)- 10/2007				
**2.2**	Total months/years of service- 10 years				
**2.3**	What are your current roles and responsibility with respect to maternal and neonatal care?<br>Examination, care, referral, Fix work not there depend on the patient. We look at the patient and by looking at them we can tell the treatment. Some are very yellowish we need LT for them urgently we needed the report. It depends on the condition of the patient if the patient is severe than patient shifted to the P/V Room we can leave the patient outside the LT we only have basic test 24*7 we don't have time to do test of everyone we have shift for basic test from LT.<br>FHS, BP check is done and we do examination and take patient history and after that we call doctor than we refer the patient. It is Natural phenomenon nurses have to trained. Examine is done if it is HRP than doctor is called and pediatrician is called				
**2.4**	How many deliveries and resuscitations of newborns have you attended in last 1 month?				
	A. No. of deliveries attended in last 1 month			20-25	
	B. No. of newborn resuscitations attended in last 1 month			10-12	
**2.5**	Who did you receive the training from:				
	Area		Training name	Year	
	A	Care during delivery (S.B.A.)	y	2008	
	B	Neonatal Resuscitation Program (In RP / NSS)	IMNCI	2010	
	C	Sick Newborn Care (FBNC)	N		

**3. Service Delivery**			
**3.1**	What are the challenges faced by you and your colleagues for delivering the desired mother and newborn care services?		
	**Challenges faced**		
		**Mother care**	**Newborn care**
	Infrastructure	Alone delivery, prenatal, postnatal, preoperation, anemia, ANC, BLOOD transfusion	Cooler is not working since 3 month there is very hot
	Equipment		
	Drugs and supplies	Blood transfusion is not done here attendant take sample from LT and shifted to jhajjar. Blood bank is closed since 2 months	
	Support services	washing external Security guard is not there in night Counselling is done by nurses	Washing Should be internal
	Other		

**3.2**	What challenges do you face while delivery of pregnant women?	
	**Challenges faced**	**How do you manage these challenges**
	Care at delivery	Delivery done by staff nurse only doctors writes notes on the file .staff nurses do dietician , pediatrician work, data entry
	Care in the ward	C4 are not available so there work also done by us
	Care of sick newborns	Baby care is done by us only
**3.3**	what challenges do you face while delivery of pregnant women?	
	**Challenges faced**	**How do you manage these challenges**

	Delivery without complication	Dietician is not available we provide 1 kg milk and biscuit to the patient which is sufficient
	Delivery with complication	
	Caesarean section	
	Referred cases with complication	
**3.4**	How long usually the mothers stay at the facility after the delivery?	
	Normal Delivery	48 hrs
	Caesarean Delivery	7-8 hrs
**4. Manpower**		
**4.1**	How many posts of Staff Nurse / ANM are vacant in your health facility?<br>There are many girls but some nurses should be there for post operating patient	
**4.2**	What difficulties do you face in providing mother and newborn care services to existing employees? (Doctors, nurses and other staff)<br>First there was surgery patient shifted to upper surgery ward and having different staff	
**4.3**	What is the mechanism of taking leave and who sanctions it?<br>ANC, PRE OP, POST OP, ALL WORK HAVE TO BE DONE BY US if there are less sister then we call other sister from other depertment	
**5. Duty Roster**		
**5.1**	Who prepares the duty roster for you?<br>Nurse incharge do	
**5.2**	Do you have flexibility in changing the shifts?<br>We can excahnge	
**5.3**	How do you manage when you have double shifts?<br>We do our duty	
**5.4**	What is the procedure for taking leave and who approves it?<br>Staff nurse done nurse incharge. We adjust by ourself holiday have to inform nurse incharge	
**5.5**	Who prepares rosters for emergency / regular service?<br>Inform Sister incharge	
**6. Infrastructure**		
**6.1**	Do you have space to accommodate changes inside the department?<br>Eclampsia room is used	
**6.2**	Do you have enough beds to accommodate increased number of patients?<br>Post-operative ward is there if not then we shifted to surgery ward. Post-operative open but care should be given. Dressing should be done properly.	
**6.3**	Is their regular power supply and clean water for drinking? Any substitute available in case of power cut or irregular water supply?<br>Ro is there. Dirty water is coming don't know what have to do. Maintance should be done and due to unclean water it is smelling	
**7. Data management**		
**7.1**	How do you record data?<br>Delivery is done, finding, pantograph, nurse sheet, pedi. Notes we fill by us like baby footprint weight, time, mother sign, records maintain	
**7.2**	How do you maintain a register?<br>Delivery, anaemia, birth register, dietary, PPUICD, 10 register have to maintain after delivery. Sometime delivery is done but registered is not maintain. Sister became less sometimes. Having high load delivery takes time than records left if there is a complication than work is left and written work is more. Please register should be less	
**7.3**	Where do you send the record?	
**7.4**	How often is the data sent?	
**8. Blood bank**		

**8.1**	How long does it take for a needy person to get blood?<br>Arrange from jhajjar that takes 2-3 hrs	
**9. Training /Skills**		
**9.1**	Could you tell us about your previous NSSK / Neonatal Resuscitation Training?	
	Time (month / year)	Time (month / year)
	place	place
	Duration (in days)	Duration (in days)
**9.2**	Who conducts the workshop? Who prepares roster for workshop/training and how it is notified? How it is monitored?	
**9.3**	What did you like the most in the training?	
**9.4**	What did you dislike the most in the training?	
**9.5**	what was the training methodology used (Lectures/ Hands-on / Practical's)?	
**9.6**	How did you like meeting / interacting with the trainers?	
**9.7**	What was their level of knowledge / skills?	
	How was your conversation with him	
**9.8**	What are the opportunities and mechanisms currently in place/adopted to retain the skills of Nurses/ANMs/Doctors?	
**9.9**	What challenges do you have with the skills of nurses and the support of staff in delivery rooms, perinatal wards and newborn care units? In your opinion, how can this be controlled?	
**9.10**	How the training related to care during delivery and newborn period can be further improved?	
**9.11**	Have you been to a skill lab set up in your district?<br>NO , done on DUMMY but don't remember	
**9.12**	What are the good things about this skill lab?	
**9.13**	What are the challenges related to skill lab?	
**9.14**	In your opinion, how many health staffs might have used or visited the skill labs?	
**9.15**	Did somebody advise or persuade you to attend the skill lab?	
**9.16**	How does the Skill Lab help in Neonatal Resuscitation and Neonatal Care?	
**10. Referral services**		
**10.1**	In what situation usually the newborns/mothers (pregnant/recently delivered) are referred to the next level of healthcare?<br>Referral after asking doctors. Referral sheet is made. S/N give treatment which is possible and then refer.	
**10.2**	Where the newborns/ pregnant women/ mothers are usually referred, what is the usual mode of transportation and how long does it takes to reach the next level health facility in your area?<br>Refer to ROHTAK. EMT is there in ambulance it reaches in 40-45 mins	
**10.3**	₹ What facilitation is done from facility side for referral and what difficulties/challenges do you face while transporting the sick newborn and mother to next level? *(Probe: monetary/logistics)*<br>No problem we handle	
**10.4**	What are the challenges faced related to referral transport experienced by this facility and how are they handled?	
**11. Logistics**		
**11.1**	Are you familiar with any scarcity / irregular supply of medicines and / or supplies required for care during delivery and newborn in the last one year? What were the reasons for this deficiency and how were these conditions managed?	

.

**11.2**	How frequently the families/ parents asked to procure drugs from outside/ store?
**11.3**	What are the supervisory mechanisms in place at present for maternal and newborn care services?
	Who supervises<br>NRHM Team
	What is the frequency of supervisory visits<br>It doesn't happen in my duty time. In 5-6 months
	Is any feedback/report provided usually after the supervision?
	What actions are taken after last supervisory visit?
**11.4**	Please let us know about the last supervisory visit to the facility related to maternal and newborn care services?
	Who came for last supervisory visit?
	How long ago the supervisory visit took place?
	What all components were observed?
	What feedback was given and what actions were taken?
**12. others**	
**12.1**	How wide do the events in discharge / record slip?<br>INDOOR no. , name, address, time birth , sex, admission, vitamin k(sometime don't write) , EBF, fallow up advice is given
**12.2**	In your view, what are the perceived barriers between families in the use of public health services for newborns of pregnant women?<br>There is no problem. Outcome is good discharge healthy. After went home ASHA , ANM look after them<br>How can these obstacles be overcome?<br>3rd and 7th day visit for fallow up in that day what happen we don't know
**12.3**	How much additional effort is needed to reduce the neonatal mortality rate in your area?<br>Mother should come to ANC visit care is given to mothers, see high risk cases, selective list is there we check and then refer. Problem of blood storage unit, doctors and nurses are less so it is better to refer. Child doctors are less so we needed doctors. All depend on us
**12.4**	According to you, what is the meaning of quality?<br>Behavior, punctuality, honesty , optimistic
**12.5**	According to you, what are the issues affecting the quality (quality) of health services?<br>Training is done in one place but now training held in different places. They give the certificate but knowledge is not given fully
**12.6**	What can you do to improve the quality of the health services?<br>When girls come we train them they learn everything in training. Cleaning is not done properly it can be done if sweeper is available inside the ward
**12.7**	Did any of your relatives, friends or acquaintances ever availed health services at this hospital? If not, any reason?<br>Yes, people only come there where charges are not taken. Result is good and good treatment is given to the patient

.

ID : 3208204	
**1 Type of Health Facility SDH, JHAJJAR**	**1.2 Designation: STAFF NURSE**

2. General

**2.1**	How long have you been working in this health facility? (months/years)-03/2014
**2.2**	Total months/years of service -3 YEARS
**2.3**	What are your current roles and responsibility with respect to maternal and neonatal care?<br>Deal patients, take over from other nurse, take information regarding patient, taking history, investigation, lifesaving of patient, mother and child care, Records entry , medicine issue, maintain register and maintain records and fills
**2.4**	How many deliveries and resuscitations of newborns have you attended in last 1 month?

A. No. of deliveries attended in last 1 month	50-60
B. No. of newborn resuscitations attended in last 1 month	

2.5 Who did you receive the training from:

	Area	Training name	Year
A	Care during delivery (S.B.A.)		
B	Neonatal Resuscitation Program (In RP / NSS)		
C	Sick Newborn Care (FBNC)		

3. Service Delivery

3.1 What are the challenges faced by you and your colleagues for delivering the desired mother and newborn care services?

	Challenges faced	
	**Mother care**	**Newborn care**
Infrastructure		
Equipment		
Drugs and supplies		
Support services		
Other		

3.2 hat challenges do you face while delivery of pregnant women?

**Challenges faced**	**How do you manage these challenges**
Care at delivery	Migratory patients don't have any investigation
Care in the ward	staff have to do this work but now counselling done after the delivery
Care of sick newborns	

3.3 What challenges do you face while delivery of pregnant women?

**Challenges faced**	**How do you manage these challenges**
Delivery without complication	
Delivery with complication	
Caesarean section	At night less but blood bank is not functioning and no storage unit
Referred cases with complication	

3.4 how long usually the mothers stay at the facility after the delivery?

Normal Delivery	3 days
Caesarean Delivery	8 days

4. Manpower

4.1 How many posts of Staff Nurse / ANM are vacant in your health facility?
yes

**4.2**	What difficulties do you face in providing mother and newborn care services to existing employees? (Doctors, nurses and other staff)	
**4.3**	What is the mechanism of taking leave and who sanctions it?	
**5. Duty Roster**		
**5.1**	Who prepares the duty roster for you?<br>Nursing sister incharge	
**5.2**	Do you have flexibility in changing the shifts?<br>yes	
**5.3**	How do you manage when you have double shifts?<br>We share duties	
**5.4**	What is the procedure for taking leave and who approves it?<br>Give application for CL	
**5.5**	Who prepares rosters for emergency / regular service?<br>NO	
**6. Infrastructure**		
**6.1**	Do you have space to accommodate changes inside the department?<br>NO, BUT WE INFORM THEM MANY TIMES	
**6.2**	Do you have enough beds to accommodate increased number of patients?<br>There are wards in 2nd floor we shifted in that floor. PNC ward is there for the patients	
**6.3**	Is their regular power supply and clean water for drinking? Any substitute available in case of power cut or irregular water supply?<br>fine	
**7. Data management**		
**7.1**	How do you record data?	
**7.2**	How do you maintain a register?<br>Indoor to discharge we maintain records, delivery register, birth register, complication, IUCD Register, LR indoor register, MTP register, ANC Records	
**7.3**	Where do you send the record?<br>Monthly records central records feed	
**7.4**	How often is the data sent?<br>monthly	
**8. Blood bank**		
**8.1**	How long does it take for a needy person to get blood?<br>Attendant patient carries to jhajjar	
**9. Training /Skills never**		
**9.1**	Could you tell us about your previous NSSK / Neonatal Resuscitation Training?	
	Time (month / year)	Time (month / year)
	place	place
	Duration (in days)	Duration (in days)
**9.2**	Who conducts the workshop? Who prepares roster for workshop/training and how it is notified? How it is monitored?	
**9.3**	What did you like the most in the training?	
**9.4**	What did you dislike the most in the training?	
**9.5**	what was the training methodology used (Lectures/ Hands-on / Practical's)?	
**9.6**	How did you like meeting / interacting with the trainers?	
**9.7**	What was their level of knowledge / skills?	
	How was your conversation with him	
**9.8**	What are the opportunities and mechanisms currently in place/adopted to retain the skills of Nurses/ANMs/Doctors?	
**9.9**	What challenges do you have with the skills of nurses and the support of staff in delivery rooms,	

.

	perinatal wards and newborn care units? In your opinion, how can this be controlled?
**9.10**	How the training related to care during delivery and newborn period can be further improved?
**9.11**	Have you been to a skill lab set up in your district?
**9.12**	What are the good things about this skill lab?
**9.13**	What are the challenges related to skill lab?
**9.14**	In your opinion, how many health staffs might have used or visited the skill labs?
**9.15**	Did somebody advise or persuade you to attend the skill lab?
**9.16**	How does the Skill Lab help in Neonatal Resuscitation and Neonatal Care?
**10. Referral services**	
**10.1**	In what situation usually the newborns/mothers (pregnant/recently delivered) are referred to the next level of healthcare?<br>Rohtak
**10.2**	Where the newborns/ pregnant women/ mothers are usually referred, what is the usual mode of transportation and how long does it takes to reach the next level health facility in your area?<br>45 MIN
**10.3**	What facilitation is done from facility side for referral and what difficulties/challenges do you face while transporting the sick newborn and mother to next level? *(Probe: monetary/logistics)*<br>Caesarian go in shock, sister accompanied with EMT
**10.4**	What are the challenges faced related to referral transport experienced by this facility and how are they handled?<br>We stable the patient. Give Medicine
**11. Logistics**	
**11.1**	Are you familiar with any scarcity / irregular supply of medicines and / or supplies required for care during delivery and newborn in the last one year? What were the reasons for this deficiency and how were these conditions managed?<br>NO
**11.2**	How frequently the families/ parents asked to procure drugs from outside/ store?<br>NO
**11.3**	What are the supervisory mechanisms in place at present for maternal and newborn care services?
	Who supervises
	What is the frequency of supervisory visits
	Is any feedback/report provided usually after the supervision?
	What actions are taken after last supervisory visit?
**11.4**	Please let us know about the last supervisory visit to the facility related to maternal and newborn care services?
	Who came for last supervisory visit?
	How long ago the supervisory visit took place?
	What all components were observed?
	What feedback was given and what actions were taken?
**12. others**	
**12.1**	How wide do the events in discharge / record slip?<br>Information of patient, breast feeding, follow up and PPIUCD
**12.2**	In your view, what are the perceived barriers between families in the use of public health services for newborns of pregnant women?<br>How can these obstacles be overcome?
**12.3**	How much additional effort is needed to reduce the neonatal mortality rate in your area?
**12.4**	According to you, what is the meaning of quality?<br>Best care a and no problem to mother and child and infection prevention

.

**12.5**	According to you, what are the issues affecting the quality (quality) of health services?<br>Timely LR fumigate is not done , only fumigation is done after HIV patien
**12.6**	What can you do to improve the quality of the health services?<br>fumigation done in every 20days, LR should be like OT
**12.7**	Did any of your relatives, friends or acquaintances ever availed health services at this hospital? If not, any reason?<br>Yes we do

.

ID: 3208205	
**1 Type of Health Facility SDH, JHAJJAR**	**1.2 Designation: STAFF NURSE**

2. General

**2.1**	How long have you been working in this health facility? (months/years)- 2/2013
**2.2**	Total months/years of service- 4 Years
**2.3**	What are your current roles and responsibility with respect to maternal and neonatal care?<br>Give over to other nurse, Autoclave instruments, Check the solution , bleaching before the delivery Vital monitor, Fill Partogarph, Maintain register, Records, We do this in one shift
**2.4**	How many deliveries and resuscitations of newborns have you attended in last 1 month?

	A. No. of deliveries attended in last 1 month	50-55
	B. No. of newborn resuscitations attended in last 1 month	

2.5 Who did you receive the training from:

	Area	Training name	Year
A	Care during delivery (S.B.A.)	SBA	2014
B	Neonatal Resuscitation Program (In RP / NSS)	Nssk, twice in jhajjar	2014
C	Sick Newborn Care (FBNC)	FBNC	2015

3. Service Delivery

3.1 What are the challenges faced by you and your colleagues for delivering the desired mother and newborn care services?

Challenges faced

	**Mother care**	**Newborn care**
Infrastructure	ECLAMPSIA patient don't have the bed – the proper beds they needed	
Equipment	Only monitor is in L.R nothing is there in L.R. OT is having everything but L.R nothing. 1 Digital thermometer and 1 is lost.<br>Having Problem in arranging for B.T	
Drugs and supplies		
Support services		
Other	C4, security is not there. Problem in night there is no security	

3.2 What challenges do you face while delivering essential newborn care services and how do you manage these?

**Challenges faced**	**How do you manage these challenges**
Care at delivery	No beds for Patients. We have to shift the patient in every 6 hrs. patient have to wait , problem in motivating the patient
Care in the ward	
Care of sick newborns	

3.3 What challenges do you face while delivery of pregnant women?

**Challenges faced**	**How do you manage these challenges**
Delivery without complication	Work load is not too much. But arranging everything is more because of this there is problem in maintain the hygiene. In one shift around 10 deliveries can be held. Sometimes there 2 -2 deliveries at the same time then there was a problem
Delivery with complication	
Caesarean section	
Referred cases with complication	

**3.4**	How long usually the mothers stay at the facility after the delivery?	
	Normal Delivery	
	Caesarean Delivery	
**4. Manpower**		
**4.1**	How many posts of Staff Nurse / ANM are vacant in your health facility?<br>Salary is less of the staff and it doesn't come on time	
**4.2**	What difficulties do you face in providing mother and newborn care services to existing employees? (Doctors, nurses and other staff)<br>If someone wants to take off there will be interfere. There is difference in permanent and other	
**4.3**	What is the mechanism of taking leave and who sanctions it?<br>Salary issue don't come on time and post is temporary	
**5. Duty Roster**		
**5.1**	Who prepares the duty roster for you?<br>Sister incharge	
**5.2**	Do you have flexibility in changing the shifts?<br>yes	
**5.3**	How do you manage when you have double shifts?<br>Doesn't happen	
**5.4**	What is the procedure for taking leave and who approves it?<br>Sister incharge M.S sir	
**5.5**	Who prepares rosters for emergency / regular service?	
**6. Infrastructure**		
**6.1**	Do you have space to accommodate changes inside the department?<br>No rest in morning and afternoon but in night 30-1 hr we get rest but we don't have room for rest and no room for nurses to sit	
**6.2**	Do you have enough beds to accommodate increased number of patients?	
**6.3**	Is their regular power supply and clean water for drinking? Any substitute available in case of power cut or irregular water supply?<br>NO Difference for staff and patient, We get from OT room, RO is not working of L.R	
**7. Data management**		
**7.1**	How do you record data?	
**7.2**	How do you maintain a register?	
**7.3**	Where do you send the record?	
**7.4**	How often is the data sent?	
**8. Blood bank**		
**8.1**	How long does it take for a needy person to get blood?<br>Depend on blood group. They come full 3-4 hrs they don't have to do anything sometimes they have to wait for the blood	
**9. Training /Skills not done**		
**9.1**	What was their level of knowledge / skills?	
	How was your conversation with him	How was your conversation with him
	What was their level of knowledge / skills?	What was their level of knowledge / skills?
	How was your conversation with him	How was your conversation with him
**9.2**	What was their level of knowledge / skills?	
**9.3**	How was your conversation with him	
**9.4**	What was their level of knowledge / skills?	
**9.5**	How was your conversation with him	
**9.6**	What was their level of knowledge / skills?	

.

**9.7**	What was their level of knowledge / skills?	
	How was your conversation with him	
**9.8**	What are the opportunities and mechanisms currently in place/adopted to retain the skills of Nurses/ANMs/Doctors?<br>Sometimes don't know where and when the training going on. Inform morning staff and in next meeting we know about the training	
**9.9**	What challenges do you have with the skills of nurses and the support of staff in delivery rooms, perinatal wards and newborn care units? In your opinion, how can this be controlled?	
**9.10**	How the training related to care during delivery and newborn period can be further improved?	
**9.11**	Have you been to a skill lab set up in your district?<br>Don't know	
**9.12**	What are the good things about this skill lab?	
**9.13**	What are the challenges related to skill lab?	
**9.14**	In your opinion, how many health staffs might have used or visited the skill labs?	
**9.15**	Did somebody advise or persuade you to attend the skill lab?	
**9.16**	How does the Skill Lab help in Neonatal Resuscitation and Neonatal Care?	
**10. Referral services**		
**10.1**	In what situation usually the newborns/mothers (pregnant/recently delivered) are referred to the next level of healthcare?<br>If severe case is there then we send the patient and after calling the pediatric then we send the child	
**10.2**	Where the newborns/ pregnant women/ mothers are usually referred, what is the usual mode of transportation and how long does it takes to reach the next level health facility in your area?<br>Rohtak 45-1 hr	
**10.3**	What facilitation is done from facility side for referral and what difficulties/challenges do you face while transporting the sick newborn and mother to next level? *(Probe: monetary/logistics)*	
**10.4**	What are the challenges faced related to referral transport experienced by this facility and how are they handled?	
**11. Logistics**		
**11.1**	Are you familiar with any scarcity / irregular supply of medicines and / or supplies required for care during delivery and newborn in the last one year? What were the reasons for this deficiency and how were these conditions managed?	
**11.2**	How frequently the families/ parents asked to procure drugs from outside/ store?	
**11.3**	What are the supervisory mechanisms in place at present for maternal and newborn care services?	
	Who supervises<br>Staff nurse or M.S sir do that	
	What is the frequency of supervisory visits<br>From outside we don't know and someone come we don't know	
	Is any feedback/report provided usually after the supervision?	
	What actions are taken after last supervisory visit?	
**11.4**	Please let us know about the last supervisory visit to the facility related to maternal and newborn care services?	
	Who came for last supervisory visit?	
	How long ago the supervisory visit took place?	
	What all components were observed?	
	What feedback was given and what actions were taken?	
**12. others**		
**12.1**	How wide do the events in discharge / record slip?	
**12.2**	In your view, what are the perceived barriers between families in the use of public health services for newborns of pregnant women?<br>Lifesaving equipment are not there like Ventilator	

	How can these obstacles be overcome?<br>Arrangement can be done
**12.3**	How much additional effort is needed to reduce the neonatal mortality rate in your area?
**12.4**	According to you, what is the meaning of quality?<br>Aseptic should be everything. Patient should be good and fine then they have to be discharge
**12.5**	According to you, what are the issues affecting the quality (quality) of health services?<br>Knowledge is less, Sterilization have to be done by C4, time problem to handle the patient
**12.6**	What can you do to improve the quality of the health services?<br>There should be doctor, staff or c4 security all should be available then only work can be then
**12.7**	Did any of your relatives, friends or acquaintances ever availed health services at this hospital? If not, any reason?<br>Yes, we do that

ID: 3208206				
**1.1 Type of Health Facility : SDH , JHAJJAR**			**1.2 Designation: STAFF NURSE**	
**2. General**				
**2.1**	How long have you been working in this health facility? (months/years)- 7/2014			
**2.2**	Total months/years of service- 3 Years			
**2.3**	What are your current roles and responsibility with respect to maternal and neonatal care?<br>Take over from the sister, check instrument, check crash cart, Check bleaching solution, See medicine treatment, Check PV of patient, Inform sister about working of any instrument equipment, Give diet to the patient, Do entries, Instrument charge was under me			
**2.4**	How many deliveries and resuscitations of newborns have you attended in last 1 month?			
	A. No. of deliveries attended in last 1 month			50-60
	B. No. of newborn resuscitations attended in last 1 month			
**2.5**	Who did you receive the training from:			
	Area		Training name	**YEAR**
	A	Care during delivery (S.B.A.)	SBA	2016
	B	Neonatal Resuscitation Program (In RP / NSS)		
	C	Sick Newborn Care (FBNC)	FBNC	2013
**3. Service Delivery**				
**3.1**	What are the challenges faced by you and your colleagues for delivering the desired mother and newborn care services?			
	**Challenges faced**			
		**Mother care**		**Newborn care**
	Infrastructure	BT cannot be done properly don't how to do properly. Need in PPH		
	Equipment			
	Drugs and supplies	BT storage was there but now storage unit is closed		
	Support services			
	Other	There is no safety. There is no security. Patient don't talk properly. Patient don't talk properly. Safety issue is there		
**3.2**	What challenges do you face while delivering essential newborn care services and how do you manage these?			
	**Challenges faced**	**How do you manage these challenges**		
	Care at delivery	BT is required then first we check then we refer urgently to jhajjar. Patient is kept different ward in post op ward but now we kept in same ward. In one shift 2 nurses are there one in labor and other is in entry. Not able to give pre care to patient so it was a big problem		
	Care in the ward			
	Care of sick newborns			
**3.3**	What challenges do you face while delivery of pregnant women?			
	**Challenges faced**	**How do you manage these challenges**		
	Delivery without complication			
	Delivery with complication			
	Caesarean section	LSCS having different staff and keep patient in different ward but now we send the patient above ward and there no one can give care		
	Referred cases with complication			

**3.4**	How long usually the mothers stay at the facility after the delivery?	
	Normal Delivery	
	Caesarean Delivery	
**4. Manpower**		
**4.1**	How many posts of Staff Nurse / ANM are vacant in your health facility?<br>We don't know. You get information from administration. Operation patients need different care so we need different people for them	
**4.2**	What difficulties do you face in providing mother and newborn care services to existing employees? (Doctors, nurses and other staff)<br>ANM, Class 4, having the same salary what we have then what was the difference between them and us. Doctors salary is high. Behavior is different for permanent and different	
**4.3**	What is the mechanism of taking leave and who sanctions it?	
**5. Duty Roster**		
**5.1**	Who prepares the duty roster for you?<br>Sister in-charge	
**5.2**	Do you have flexibility in changing the shifts?<br>yes	
**5.3**	How do you manage when you have double shifts?<br>No it doesn't happen	
**5.4**	What is the procedure for taking leave and who approves it?<br>Sister in-charge and M.S sir	
**5.5**	Who prepares rosters for emergency / regular service?<br>Sister in-charge	
**6. Infrastructure**		
**6.1**	Do you have space to accommodate changes inside the department?<br>No changing room for sisters there is one small room but that room is dirty	
**6.2**	Do you have enough beds to accommodate increased number of patients?	
**6.3**	Is their regular power supply and clean water for drinking? Any substitute available in case of power cut or irregular water supply?	
**7. Data management**		
**7.1**	How do you record data?	
**7.2**	How do you maintain a register?	
**7.3**	Where do you send the record?	
**7.4**	How often is the data sent?	
**8. Blood bank**		
**8.1**	How long does it take for a needy person to get blood?<br>1 week before it takes 5-6 hr. patient get rude then we referred the patient	
**9. Training /Skills**		
**9.1**	Could you tell us about your previous NSSK / Neonatal Resuscitation Training?	
	Time (month / year)	NSSK 2014
	place	jhajjar
	Duration (in days)	2 days
**9.2**	Who conducts the workshop? Who prepares roster for workshop/training and how it is notified? How it is monitored?<br>In Roaster mark training sister in-charge do the roster entry	
**9.3**	What did you like the most in the training?<br>Dr. explain us properly we understand the things easily Training is done good	

.

**9.4**	What did you dislike the most in the training?<br>2 times of Training NSSK or don't give certificate. We only know about training there is no proof	
**9.5**	What was the training methodology used (Lectures/ Hands-on / Practical's)?<br>They give instruction book and practical is also then	
**9.6**	How did you like meeting / interacting with the trainers?<br>Explain us properly understand everything	
**9.7**	What was their level of knowledge / skills?	gynecologist
	How was your conversation with him	Only my meeting was there
**9.8**	What are the opportunities and mechanisms currently in place/adopted to retain the skills of Nurses/ANMs/Doctors?<br>Monthly meeting should be there and inform us about the training.<br>Tell us about new technology in the meeting	
**9.9**	What challenges do you have with the skills of nurses and the support of staff in delivery rooms, perinatal wards and newborn care units? In your opinion, how can this be controlled?Meeting should be held and give training in the starting of the month and if someone wants off than they take off in the end of the month	
**9.10**	How the training related to care during delivery and newborn period can be further improved?<br>Timely improve the knowledge than it will help in improve confidence and help in handle patient	
**9.11**	Have you been to a skill lab set up in your district?<br>Don't know	
**9.12**	What are the good things about this skill lab?	
**9.13**	What are the challenges related to skill lab?	
**9.14**	In your opinion, how many health staffs might have used or visited the skill labs?	
**9.15**	Did somebody advise or persuade you to attend the skill lab?	
**9.16**	How does the Skill Lab help in Neonatal Resuscitation and Neonatal Care?	
**10. Referral services**		
**10.1**	In what situation usually the newborns/mothers (pregnant/recently delivered) are referred to the next level of healthcare?<br>Yes, we also went with the patient in emergency case. Take patient with care in ambulance. Send patient in ventilator	
**10.2**	Where the newborns/ pregnant women/ mothers are usually referred, what is the usual mode of transportation and how long does it takes to reach the next level health facility in your area?<br>Rohtak 40-45 min	
**10.3**	What facilitation is done from facility side for referral and what difficulties/challenges do you face while transporting the sick newborn and mother to next level? *(Probe: monetary/logistics)*	
**10.4**	What are the challenges faced related to referral transport experienced by this facility and how are they handled?	
**11. Logistics**		
**11.1**	Are you familiar with any scarcity / irregular supply of medicines and / or supplies required for care during delivery and newborn in the last one year? What were the reasons for this deficiency and how were these conditions managed?	
**11.2**	How frequently the families/ parents asked to procure drugs from outside/ store?	
**11.3**	What are the supervisory mechanisms in place at present for maternal and newborn care services?	
	Who supervises	
	What is the frequency of supervisory visits	
	Is any feedback/report provided usually after the supervision?	
	What actions are taken after last supervisory visit?	
**11.4**	Please let us know about the last supervisory visit to the facility related to maternal and newborn care services?	
	Who came for last supervisory visit?	

.

	How long ago the supervisory visit took place?
	What all components were observed?
	What feedback was given and what actions were taken?
**12. OTHERS**	
**12.1**	How wide do the events in discharge / record slip?
**12.2**	In your view, what are the perceived barriers between families in the use of public health services for newborns of pregnant women?<br>How can these obstacles be overcome?
**12.3**	How much additional effort is needed to reduce the neonatal mortality rate in your area?
**12.4**	According to you, what is the meaning of quality?<br>All things should be hygienic and provide good services to everyone
**12.5**	According to you, what are the issues affecting the quality (quality) of health services?<br>Need Blood bank. New instrument should be there or provide knowledge to everyone
**12.6**	What can you do to improve the quality of the health services?<br>Yes we did
**12.7**	Did any of your relatives, friends or acquaintances ever availed health services at this hospital? If not, any reason?

ID: 3208207				
**1.1 Type of Health Facility-SDH, JHAJJAR**				**1.2 Designation: STAFF NURSE**
**2. General**				
**2.1**	How long have you been working in this health facility? (months/years)- 2/2009 PHC BERI			
**2.2**	Total months/years of service – 3 YEARS			
**2.3**	What are your current roles and responsibility with respect to maternal and neonatal care?<br>Giving in-charge to next nurse morning having over load after that was fine, Arrange bedding for patient, Cleaning, File complete, Doctors write on the file give that treatment to the patient<br>Collect sample			
**2.4**	How many deliveries and resuscitations of newborns have you attended in last 1 month?			
	A. No. of deliveries attended in last 1 month			
	B. No. of newborn resuscitations attended in last 1 month		1-2 Delivery + baby problem call sir he tell on the phone we do that	
**2.5**	Who did you receive the training from:			
	Area		Training name	Year
	A	Care during delivery (S.B.A.)		
	B	Neonatal Resuscitation Program (In RP / NSS)	Nssk , jhajjar	2010
	C	Sick Newborn Care (FBNC)	FBNC, DELHI	2015

3. Service Delivery

3.1 What are the challenges faced by you and your colleagues for delivering the desired mother and newborn care services?

Challenges faced		
	**Mother care**	**Newborn care**
Infrastructure		1. Phototherapy machine<br>2. O2 Cylinder
Equipment		
Drugs and supplies	Cal. potassium test is not done here they have to do from outside	Biochemistry test not done here<br>Drugs problem but if we inform it will come<br>Jaundice test also not done
Support services		Class 4 don't come and in evening class4 not avaliable
Other	Test sample collected from L.R they don't come to SNCU. There is problem in collecting sample. Lt don't collect sample	Lt having problem in collecting sample of the baby

3.2 What challenges do you face while delivering essential newborn care services and how do you manage these?

**Challenges faced**	**How do you manage these challenges**
Care at delivery	
Care in the ward	
Care of sick newborns	

3.3 What challenges do you face while delivery of pregnant women?

**Challenges faced**	**How do you manage these challenges**
Delivery without complication	

	Delivery with complication	
	Caesarean section	
	Referred cases with complication	
**3.4**	How long usually the mothers stay at the facility after the delivery?	
	Normal Delivery	
	Caesarean Delivery	
**4. Manpower**		
**4.1**	How many posts of Staff Nurse / ANM are vacant in your health facility??<br>L.R and SNCU nurse don't change shift because there will problem in handling SNCU baby. Need training before shifting. They thought baby are sleeping we are only sitting and talking but handing baby and giving treatment is big problem	
**4.2**	What difficulties do you face in providing mother and newborn care services to existing employees? (Doctors, nurses and other staff)	
**4.3**	What is the mechanism of taking leave and who sanctions it?	
**5. Duty Roster**		
**5.1**	Who prepares the duty roster for you?	
**5.2**	Do you have flexibility in changing the shifts?	
**5.3**	How do you manage when you have double shifts?	
**5.4**	What is the mechanism of taking leave and who sanctions it?<br>Nurse in-charge. Shift arrange by her don't change the shift	
**5.5**	What is the procedure for taking leave and who approves it?	
**6. Infrastructure**		
**6.1**	Do you have space to accommodate changes inside the department?<br>Store room we made it was not available	
**6.2**	Do you have enough beds to accommodate increased number of patients?<br>KMC is there we adjust patient in that room. adjust 2 patient in one bed	
**6.3**	Is their regular power supply and clean water for drinking? Any substitute available in case of power cut or irregular water supply?<br>First there was not there but now there is RO water	
**7. Data management**		
**7.1**	How do you record data?<br>Fill Case sheet. Fill indoor register all patient entry done	
**7.2**	How do you maintain a register?<br>Given to staff	
**7.3**	Where do you send the record?<br>Daily entry done and send	
**7.4**	How often is the data sent?	
**8. Blood bank**		
**8.1**	How long does it take for a needy person to get blood?	
**9. Training /Skills**		
**9.1**	Could you tell us about your previous NSSK / Neonatal Resuscitation Training?	
	Time (month / year)	
	place	jhajjar
	Duration (in days)	2 days

.

**9.2**	Who conducts the workshop? Who prepares roster for workshop/training and how it is notified? How it is monitored?<br>Nurse in-charge she arrange everything. Off adjust she only done that
**9.3**	What did you like the most in the training?<br>Not Good training they can tell many thing There was no special
**9.4**	What did you dislike the most in the training?<br>Practical is not done and so much crowd not understand properly
**9.5**	What was the training methodology used (Lectures/ Hands-on / Practical's)?<br>Dummy is there. Class training is given
**9.6**	How did you like meeting / interacting with the trainers?<br>Crowded so many people not able to see
**9.7**	What was their level of knowledge / skills? | pediatrician
	How was your conversation with him |
**9.8**	What are the opportunities and mechanisms currently in place/adopted to retain the skills of Nurses/ANMs/Doctors?<br>Training should be here it should be outside. Our doctor should only give training
**9.9**	What challenges do you have with the skills of nurses and the support of staff in delivery rooms, perinatal wards and newborn care units? In your opinion, how can this be controlled?
**9.10**	How the training related to care during delivery and newborn period can be further improved?
**9.11**	Have you been to a skill lab set up in your district?
**9.12**	What are the good things about this skill lab?
**9.13**	What are the challenges related to skill lab?
**9.14**	In your opinion, how many health staffs might have used or visited the skill labs?
**9.15**	Did somebody advise or persuade you to attend the skill lab?
**9.16**	How does the Skill Lab help in Neonatal Resuscitation and Neonatal Care?
**10. Referral services**	
**10.1**	In what situation usually the newborns/mothers (pregnant/recently delivered) are referred to the next level of healthcare?<br>rohtak
**10.2**	Where the newborns/ pregnant women/ mothers are usually referred, what is the usual mode of transportation and how long does it takes to reach the next level health facility in your area?<br>Baby IV is given and give instruction to the EMT if there is no EMT there we have to go with the patient
**10.3**	What facilitation is done from facility side for referral and what difficulties/challenges do you face while transporting the sick newborn and mother to next level? *(Probe: monetary/logistics)*If 2 sister are there then one is gone with the patient then there is problem single can handle. Problem in sunday
**10.4**	What are the challenges faced related to referral transport experienced by this facility and how are they handled?<br>EMT IS NOT AVALIABLE. Extra should be there and Sunday off should be given
**11. Logistics**	
**11.1**	Are you familiar with any scarcity / irregular supply of medicines and / or supplies required for care during delivery and newborn in the last one year? What were the reasons for this deficiency and how were these conditions managed?<br>No. There is available during emergency

.

**11.2**	How frequently the families/ parents asked to procure drugs from outside/ store?<br>NO. Medicine are available if not then we arrange that
**11.3**	What are the supervisory mechanisms in place at present for maternal and newborn care services?
	Who supervises<br>Sister in-charge.
	What is the frequency of supervisory visits<br>Register check is done
	Is any feedback/report provided usually after the supervision?
	What actions are taken after last supervisory visit?
**11.4**	Please let us know about the last supervisory visit to the facility related to maternal and newborn care services?
	Who came for last supervisory visit?
	How long ago the supervisory visit took place?
	What all components were observed?
	What feedback was given and what actions were taken?
**12. OTHERS**	
**12.1**	How wide do the events in discharge / record slip?<br>Slip is made. Counselling breastfeeding and antibiotics is given and explain them. Follow up of is done by staff
**12.2**	In your view, what are the perceived barriers between families in the use of public health services for newborns of pregnant women?<br>Class 4 is less. Less doctors<br>How can these obstacles be overcome?<br>Doctor should be provided and should giving training to all
**12.3**	How much additional effort is needed to reduce the neonatal mortality rate in your area?
**12.4**	According to you, what is the meaning of quality?<br>all things should be working properly if somethting is old than we cant give quality. this should be there if we need anything is should be working properly it not needed to check. all things should be working smoothly
**12.5**	According to you, what are the issues affecting the quality (quality) of health services?<br>O2 cylinder are available but needed class 4 to change cylinder and then we have to call above for class 4 and sometime they say we are not able to change
**12.6**	What can you do to improve the quality of the health services?<br>Doctor should be available There is a demand of doctor. Doctor t come for round for checkup but doctor should be there
**12.7**	Did any of your relatives, friends or acquaintances ever availed health services at this hospital? If not, any reason?<br>We are not from here but bring my children here for any treatment

ID: 3208301	
AREA DH, jhajjar	DESIGNATION : class IV
**S.No.**	**Questions**
**1.**	**General Information**
1.1	Are you an employee of the hospital or are you contracted for the same?<br>Contracted for 1 year. The contract changes after every 3-6 months
1.2	How many class 4 are there in this hospital?
**2.**	**Specific Information**
2.1	Please tell us about the duty of duty and how many people are present in the cross<br>2 in the morning and 1 in the night. Inside LR only in the morning.
2.2	Your responsibility is only of one department, of all the other departments of the hospital?<br>The whole of LR is my responsibility
2.3	Explain your responsibilities<br>To room doctor room and other rooms. To do autoclaving. If there is any case, I have to be with the staff. I also prepare the bleaching solution.
2.4	Do you have a duty even during the night shift?<br>No there are 4 offs for everybody. Every 6 months the posting changes. Everyone is trained. The sweeper cleans the equipment at night. The sweeper is a trained dai
2.5	Tell us which places in this hospital do you take care of cleanliness?<br>Labour room, doctor room and examination room.
2.6	Tell us when and when do you clean the premises?<br>After every case the equipment is to be cleaned. We dust the place once every morning
2.7	What media do you use for cleaning many types of goods?<br>We put the equipment in the bleaching solution and clean it. 3 spoons in 1 litre of water. We put it in the solution for a while and separate the water.
2.8	Does anyone check your work?
**3.**	**Disposal**
3.1	How you are you dispose the waste?<br>Sweeper does this work. We do put the waste. During dusting we come across some waste.
3.2	Do you use color coding systems for waste disposal?<br>Red- plastic, yellow- ladies waste, cotton, blue- glass, black- paper
3.3	There is any vehicle for pickup the waste?<br>There are color coded bins in every room.
3.4	If yes, then how many times is it came in the hospital?<br>That I don't know
3.5	Do you burn the waste room? If so where<br>We keep the placenta bin separate. They take it away in a vehicle. We don't burn here.
**4.**	**Issues/Suggestions**
4.1	tell us how other staff members treat you in hospital<br>It is alright
4.2	tell us how patients treat you in the hospital<br>There is no problem
4.3	tell us what difficulty you face during your work<br>We don't get the salary in time. We do our jobs. The staff is less too.
4.4	Want to change the way you do for better service?<br>Sister would tell you that.

**4.5**	What do you mean by quality?<br>C4 should be more. It will help to take care of LR completely. I don't know what else can be done to improve.
**4.6**	According to you, what are the issues affecting the quality of health services?<br>There should be improvement. That sister can tell
**4.7**	What can you do to improve the quality of health services?
**4.8**	Have your relatives, friends or acquaintances ever taken advantage of the health services of this hospital? If not, any reason?<br>No. we do tell them. but they don't know what happens here. My mother in law got her eye checked up here.

ID 3208302	
**AREA DH, jhajjar**	**DESIGNATION : class IV**
**S.No.**	**Questions**
**1.**	**General Information**
1.1	Are you an employee of the hospital or are you contracted for the same?<br>Contracted for 6 months. We don't even know who our supervisor is. We don't know what is written on the contract
1.2	How many class 4 are there in this hospital?<br>There are 3 C4 and 1 sweeper in the morning. 1 in the morning and 1 in night shift.
**2.**	**Specific Information**
2.1	Please tell us about the duty of duty and how many people are present in the cross<br>Only one.
2.2	Your responsibility is only of one department, of all the other departments of the hospital?
2.3	explain your responsibilities<br>Clean the instruments and then sit at the gate. We stop if someone enters. We also send or receive the babies for feed.
2.4	Do you have a duty even during the night shift?<br>Yes
2.5	Tell us which places in this hospital do you take care of cleanliness?<br>Bedding, sheets are changed. The washer man takes out the old sheets.<br>Right from the SNCU to the pediatric ward.
2.6	Tell us about your family's success?<br>Once in the morning and once in every shift thereafter.
2.7	What media do you use for cleaning many types of goods?<br>We use surf to clean the tiles. Ezee spray for the glasses. Inside the facility we use solution for instrument and warmer so that it doesn't smell, using a cloth.
2.8	Does anyone check your work?<br>No the nursing sister tells us.
**3.**	**Disposal**
3.1	How you are you dispose the waste?<br>The sweeper picks it up. We put it in color coded bags.
3.2	Do you use color coding systems for waste disposal? Detailed<br>Black is for normal. Red- plastic, Yellow- for blood, Blue for glass. All about this Dr. tells us. We wear gloves before doing it.
3.3	There is any vehicle for pickup the waste?<br>Sweeper takes it out. Then they take it away.
3.4	If yes, then how many times is it come in the hospital?
3.5	Do you burn the waste room? If so where<br>No
**4.**	**Issues/Suggestions**
4.1	tell us how other staff members treat you in hospital<br>It is really good.
4.2	tell us how patients treat you in the hospital<br>Happens sometimes, they tell us sometimes.
4.3	tell us what difficulty you face during your work<br>I have no issues as such. I do my job. I do it on time. I try my best to do what they say. I don't feel the workload is too much.

4.4	Want to change the way you do for better service?<br>We distribute the work among ourselves in different shifts. If we get the salary on time that would be more than enough. Some nice contractor that comes, then it would be good. Nobody comes ahead in fear.
**4.5**	What do you mean by quality?<br>If someone does something wrong they would call out wrong to them. If someone does well, they'll say well.
**4.6**	According to you, what are the issues affecting the quality of health services?<br>It's all good according to me.
**4.7**	What can you do to improve the quality of health services?
**4.8**	Have your relatives, friends or acquaintances ever taken advantage of the health services of this hospital? If not, any reason?

ID: 3308201

1 Type of Health Facility: FRU 2, JHAJJAR 1.2 **Designation: STAFF NURSE**

2. General

**2.1**	How long have you been working in this health facility? (months/years)- 2 YEAR BACK
**2.2**	Total months/years of service - PHC 9 YEAR+ 2 YEAR CHC
**2.3**	What are your current roles and responsibility with respect to maternal and neonatal care?<br>Check ANC Patient, Indoor records, OPD – injection, PPUICD, INJECTION, B.P measure, Sterilization, cleaning by supporting staff after delivery, Refer to jhajjar, Ambulance calling
**2.4**	How many deliveries and resuscitations of newborns have you attended in last 1 month?

A. No. of deliveries attended in last 1 month	6
B. No. of newborn resuscitations attended in last 1 month	No (last month)

2.5 Who did you receive the training from:

	Area	Training name	**YEAR**
A	Care during delivery (S.B.A.)	SBI, BIOWASTE, PPUICD	JAN 2015, DEC 2014
B	Neonatal Resuscitation Program(In RP / NSS)	NSSK	2016
C	Sick Newborn Care (FBNC)		2014

3. Service Delivery

3.1 What are the challenges faced by you and your colleagues for delivering the desired mother and newborn care services?

Challenges faced

	**Mother care**	**Newborn care**
Infrastructure	All things are good	
Equipment		
Drugs and supplies		
Support services		
Other		

3.2 What challenges do you face while delivering essential newborn care services and how do you manage these?

**Challenges faced**	**How do you manage these challenges**
Care at delivery- MO IS NOT AVALIABLE	Refer to jhajjar
Care in the ward - Patient don't clean properly	Sweeper clean everytime
Care of sick newborns Doctor not available and patient parents not treat properly	Refer to jhajjar

3.3 What challenges do you face while delivery of pregnant women?

**Challenges faced**	**How do you manage these challenges**
Delivery without complication normal we can handle easly	We can handle, management can be done and first treatment given to them
Delivery with complication	REFER TO JHAJJAR
Caesarean section	Refer to jhajjar
Referred cases with complication	Call ambulance and if there is an emergency then we also go with the patient

**3.4**	How long usually the mothers stay at the facility after the delivery?	
	Normal Delivery	3 days 24-36 hr
	Caesarean Delivery	
**4. Manpower**		
**4.1**	How many posts of Staff Nurse / ANM are vacant in your health facility?<br>Permanent 5-6. 2 required in emergency	
**4.2**	What difficulties do you face in providing mother and newborn care services to existing employees? (Doctors, nurses and other staff)<br>Normal we can do easily but high risk cases are refer to jhajjar	
**4.3**	What is the mechanism of taking leave and who sanctions it?<br>jhajjar	
**5. Duty Roster**		
**5.1**	Who prepares the duty roster for you?<br>We only do that	
**5.2**	Do you have flexibility in changing the shifts?<br>We only do that	
**5.3**	How do you manage when you have double shifts?<br>We handle easily as OPD is closed in evening to it was easy to handle and delivery cases are less in number during this time	
**5.4**	What is the procedure for taking leave and who approves it?<br>Doctor	
**5.5**	Who prepares rosters for emergency / regular service?<br>We only make but doctor look after that	
**6. Infrastructure**		
**6.1**	Do you have space to accommodate changes inside the department?<br>All good but make SNCU properly	
**6.2**	Do you have enough beds to accommodate increased number of patients?<br>We have enough bed and patients are less	
**6.3**	Is their regular power supply and clean water for drinking? Any substitute available in case of power cut or irregular water supply?<br>invertor are available and 2 aqua Gard are there	
**7. Data management**		
**7.1**	How do you record data?<br>Different register are there for different data and Handle shift wise nurses	
**7.2**	How do you maintain a register?<br>Handover to other staff, fill monthly performer	
**7.3**	Where do you send the record?<br>Available in Almira, monthly performer is filled and given information to ASSISSTANT	
**7.4**	How often is the data sent?<br>Monthly	
**8. Blood bank**		
**8.1**	How long does it take for a needy person to get blood?<br>Refer to jhajjar	
**9. Training /Skills**		
**9.1**	Could you tell us about your previous NSSK / Neonatal Resuscitation Training?	
	Time (month / year)	
	place	
	Duration (in days)	

.

**9.2**	Who conducts the workshop? Who prepares roster for workshop/training and how it is notified? How it is monitored?<br>Doctor	
**9.3**	What did you like the most in the training?<br>Practical demonstration	
**9.4**	What did you dislike the most in the training?<br>All is good	
**9.5**	What was the training methodology used (Lectures/ Hands-on / Practical's)?<br>Lecture and practical	
**9.6**	How did you like meeting / interacting with the trainers?<br>Doctor and team all were good	
**9.7**	What was their level of knowledge / skills?good	We understand everything
	How was your conversation with him<br>Good, nice	Talk politely talk nicely, good behaviour
**9.8**	What are the opportunities and mechanisms currently in place/adopted to retain the skills of Nurses/ANMs/Doctors?<br>Training only but since when I am posted here no training was held	
**9.9**	What challenges do you have with the skills of nurses and the support of staff in delivery rooms, perinatal wards and newborn care units? In your opinion, how can this be controlled?<br>Doctors are not available we handle by our self if not handle than refer to jhajjar	
**9.10**	How the training related to care during delivery and newborn period can be further improved?<br>Provide MO, Gynae and Pediatrician. SNCU- provide nurses and doctors	
**9.11**	Have you been to a skill lab set up in your district?<br>Not here	
**9.12**	What are the good things about this skill lab?	
**9.13**	What are the challenges related to skill lab?	
**9.14**	In your opinion, how many health staffs might have used or visited the skill labs?	
**9.15**	Did somebody advise or persuade you to attend the skill lab?	
**9.16**	How does the Skill Lab help in Neonatal Resuscitation and Neonatal Care?	
**10. Referral services**		
**10.1**	In what situation usually the newborns/mothers (pregnant/recently delivered) are referred to the next level of healthcare?<br>Baby don't cry immediately, cyanosis, respiratory distress,<br>Mother – PPH, Fatigue , LSCS, obstructed labor, anemia, hypertensive, low height patient, No progress labor	
**10.2**	Where the newborns/ pregnant women/ mothers are usually referred, what is the usual mode of transportation and how long does it takes to reach the next level health facility in your area?<br>Jhajjar- call AMBULANCE , OPD slip and reached in 20-30 min	
**10.3**	What facilitation is done from facility side for referral and what difficulties/challenges do you face while transporting the sick newborn and mother to next level? *(Probe: monetary/logistics)*<br>FIRST AID, OXYGEN, BAG MASK, ventilation, Some are cooperative and some are not cooperative	
**10.4**	What are the challenges faced related to referral transport experienced by this facility and how are they handled?<br>No problem	
**11. Logistics**		
**11.1**	Are you familiar with any scarcity / irregular supply of medicines and / or supplies required for care during delivery and newborn in the last one year? What were the reasons for this deficiency and how were these conditions managed?<br>For emergency medicine are available	

.

**11.2**	How frequently the families/ parents asked to procure drugs from outside/ store?<br>All are available
**11.3**	What are the supervisory mechanisms in place at present for maternal and newborn care services?
	Who supervises<br>Ourself and doctor
	What is the frequency of supervisory visits?<br>1-2 times
	Is any feedback/report provided usually after the supervision?<br>yes
	What actions are taken after last supervisory visit?<br>Treatment given to patient if required emergency treatment refer to jhajjar
**11.4**	Please let us know about the last supervisory visit to the facility related to maternal and newborn care services?
	Who came for last supervisory visit?<br>CMO JHAJJAR
	How long ago the supervisory visit took place?<br>1-2 HR
	What all components were observed?<br>LOOK AFTER EVERYTHING
	What feedback was given and what actions were taken?<br>Maintain register
**12. OTHERS**	
**12.1**	How wide do the events in discharge / record slip?<br>Before referral medicine is given to the patient, ANTIBIOTICS, HB, BP, Immunization,<br>Baby height, weight time of birth written
**12.2**	In your view, what are the perceived barriers between families in the use of public health services for newborns of pregnant women?<br>Security guard are there they handle if any problem taken place but they understand us also<br>How can these obstacles be overcome?
**12.3**	How much additional effort is needed to reduce the neonatal mortality rate in your area?<br>Staff nurse and doctors
**12.4**	According to you, what is the meaning of quality?<br>Responsibility do properly and come on time on duty
**12.5**	According to you, what are the issues affecting the quality (quality) of health services?<br>Wecanhandlethingsbutsometimesomepeoplecomewhodon'tunderstandandarerudeproblem<br>to handle them
**12.6**	What can you do to improve the quality of the health services?<br>Duty do properly come on time, staff cooperation, counselor should be there for attender
**12.7**	Did any of your relatives, friends or acquaintances ever availed health services at this hospital? If not, any reason?<br>Yes they here only

1. **ID : 1168101**		
**1.1 Type of Health Facility: District hospital, FARIDABAD**	**1.2 Designation: Medical officer**	
**2. General**		
**2.1**	How long have you been working in this health facility? (months/years)	
**2.2**	Total months/years of service	
**2.3**	What are your current roles and responsibility with respect to maternal and neonatal care?<br>I am working as gynecologist. I supervise normal deliveries and conduct LSCS.	
**3**	What do you mean by quality in the care provided by you at this hospital?<br>Quality of care means sterilization. Patient should not get hospital acquired infections. It refers to cleanliness and regular monitoring of services.	
**4**	In your opinion what all have been done to improve the quality of care?<br>Staff is more responsible than earlier. Cleanliness is improved. Infection control practices are now followed.	
**5**	What do you know about the quality of care project undertaken/ implemented at your hospital?<br>I know about the details of activities conducted by QOC teams such as CFA of patients attending ANC and LR. Improvisation of hand hygiene practices is done by the QOC team which was really helpful for the staff.	
**6**	What all changes did you see at the hospital?<br>There are changes in biomedical waste management. Now we always alert the staff to follow the new BMW guidelines. Training sessions are being regularly conducted for the staff. Regular visits of officers are being done on routine basis.	
**7**	What all skill building efforts were done at this facility/ hospital? How that helped you?<br>Training sessions are being regularly conducted for the staff. In previous visit of NQAS, staff was not aware about eclampsia but this time, the knowledge of staff was updated and they answered all the questions asked by the assessor. I think these changes are because of regular trainings provided to them.	
**8**	What all changes do mean see in the record keeping /case sheet maintenance for the patients?<br>Yes, there are huge changes in record keeping. Earlier the staff was reluctant but now the scenario has been changed. The staff follow and maintain all the records and keep the records updated.	
**9**	What all changes do you see in the disinfection practices in your unit?<br>Overall cleanliness and hand hygiene practices have been improved. But instruments are sometimes not been sterilized. We always have to do constant supervision to maintain that.	
**10**	In your opinion how these changes would help the patients and you?<br>Both patients and doctors are benefited with these changes. Patients are more satisfied and for us, it is good to see that rate of secondary infection has been reduced.	
**11**	What more could have done to improve the quality of care for patients at this hospital?<br>Sterilization of instruments should be constantly monitored. Quality improvement system should be continued. Guards should be more attentive.	
**12**	Has there been any change in the supervision and monitoring at this hospital?<br>Yes, there is lot of changes now. Earlier, we used to take things for granted but now everything is changed. We regularly monitor vitals of patient like BP, pulse, FHR and keep the updated record.	
**13**	How have the quality of care project team helped you and other members/ staffs at this hospital?<br>QOC team is very helpful. Due to constant presence of your team, chances of disinfection are improved. Skills of staff have been improved due to regular training sessions. Record keeping system has been improved because of regular monitoring by the QOC team and discussing the gaps found while evaluation.	
**14**	What all challenges did you experience with the quality of care project team?<br>No, I didn't find any challenge with your presence.	
**15**	After the quality of care project finishes how the quality improvement efforts can be continued?<br>These improvements should be sustained. People should be sensitized about how and why we do infection control practices. One dedicated person should be appointed to supervise all the quality things and another person shall supervise that person. Daily visits of the concerned person should be done.	
**16**	Any other comments	

Name of Interviewer______________________ Signature________________

1. **ID : 1168102**		
**1.1 Type of Health Facility: District hospital , FBD**	**1.2 Designation: Medical officer**	
**2. General**		
**2.1**	How long have you been working in this health facility? (months/years)	8 years
**2.2**	Total months/years of service	18 years
**2.3**	What are your current roles and responsibility with respect to maternal and neonatal care?<br>I am appointed here for NICU through NHM. I also look for administrative work, do rounds at causality, pediatric ward, attend C/S and OPD.	
**3**	What do you mean by quality in the care provided by you at this hospital?<br>Quality means timely and immediate care. Medicines and supplies should be regularly available in the department.	
**4**	In your opinion what all have been done to improve the quality of care?<br>Cleanliness is improved in the department but sometimes due to shortage of staff, it is difficult for us to give immediate care.	
**5**	What do you know about the quality of care project undertaken/ implemented at your hospital?<br>Your team come and takes data. Basically you check patient handling techniques by doctor and staff nurse. You observe record keeping and do supervision to find the gaps in the processes and later discuss the problems and gaps with us to find the solution.	
**6**	What all changes did you see at the hospital?<br>There is improvement in hand washing practices and record keeping. Staff is now sensitized and aware about the infection control practices.	
**7**	What all skill building efforts were done at this facility/ hospital? How that helped you?<br>Yes, regular trainings were conducted in the department but only 50% staff was covered. The staff which received training was benefited from these skill building efforts.	
**8**	What all changes do mean see in the record keeping /case sheet maintenance for the patients?<br>Record keeping and file maintenance is much more improved now. The shortcomings were discussed in the training sessions and we gradually improved the record maintenance system.	
**9**	What all changes do you see in the disinfection practices in your unit?<br>Huge changes are seen in disinfection practices. Hand washing practice is followed by all staff and patient's attendants. Cleanliness is improved and fumigation is done on regular intervals.	
**10**	In your opinion how these changes would help the patients and you?<br>Outcome of patient have been improved as less number of sepsis cases are seen now.	
**11**	What more could have done to improve the quality of care for patients at this hospital?<br>Regular supply of materials should be available. Due to this, many patients have to face problem as we ask them to buy from outside.	
**12**	Has there been any change in the supervision and monitoring at this hospital?<br>Yes, state teams and other senior officials are regularly visiting our department now.	
**13**	How have the quality of care project team helped you and other members/ staffs at this hospital?<br>QOC team is very helpful in improving infection control practices and record maintenance.	
**14**	What all challenges did you experience with the quality of care project team?<br>No, I didn't had any challenge while working with your team.	
**15**	After the quality of care project finishes how the quality improvement efforts can be continued?<br>A designated staff should be appointed for quality improvement.	
**16**	Any other comments	

Name of Interviewer______________________ Signature________________

1. **ID: 1168103**		
**1.1 Type of Health Facility : District hospital, FBD**		**1.2 Designation: Medical officer**
**2. General**		
**2.1**	How long have you been working in this health facility? (months/years)	15 years
**2.2**	Total months/years of service	32 years
**2.3**	What are your current roles and responsibility with respect to maternal and neonatal care?<br>I am working as gynecologist. I check all the ANC patients at OPD.	
**3**	What do you mean by quality in the care provided by you at this hospital?<br>Quality of care means to provide best health services in time to all the patients. Emergency patients should be given priority.	
**4**	In your opinion what all have been done to improve the quality of care?<br>Total time for checkup is reduced for the patients especially for those coming in third trimester and are high risk. High risk category are now been segregated and given priority in checkup. Time duration for registration is also reduced by creating a separate queue for high risk patients.	
**5**	What do you know about the quality of care project undertaken/ implemented at your hospital?<br>Quality of care project is associated with improving health services for pregnant women and newborn. Many changes have been implemented under this project. ANC records are now up to date and time taken for pregnant women for checkup is also reduced to almost half by making priority queue for high risk patients.	
**6**	What all changes did you see at the hospital?<br>Cleanliness is improved in the hospital. Waiting time for OPD visit and registration have been reduced. High risk patients are segregated in the OPD and the cards are stamped for priority checkup. Counseling is also given to women coming for ANC checkup.	
**7**	What all skill building efforts were done at this facility/ hospital? How that helped you?<br>Trainings have been given to the staff regarding counseling, infection control and clinical components are also been revised to all the staff members.	
**8**	What all changes do mean see in the record keeping /case sheet maintenance for the patients?<br>Records are now updated regularly. ANC patients are now registered in the facility and high risk patients are segregated. Records for counseling is also updated regularly.	
**9**	What all changes do you see in the disinfection practices in your unit?<br>Infection control practices are very improved. OPD area is very clean then earlier. Hand washing practices are maintained by all the staff members. We get regular supply of soap and handrub now.	
**10**	In your opinion how these changes would help the patients and you?<br>These changes are very helpful to the patients and doctors. High risk patients are benefitted most by these changes. Now they don't have to wait for long hours to get their routine checkup done. Infection control practices avoid patients and us to get contaminated with bacterial or any other secondary infection.	
**11**	What more could have done to improve the quality of care for patients at this hospital?<br>Colour coded OPD cards should be printed. It will be easier to identify high risk patients in the queue. 1 more doctor is required at the OPD.	
**12**	Has there been any change in the supervision and monitoring at this hospital?<br>Yes, now many teams visit the hospital for supervision. Due to nqas, laqshay and kayakalp guidelines, CMO and PMO are also making regular visits.	
**13**	How have the quality of care project team helped you and other members/ staffs at this hospital?<br>QOC team is very helpful. Due to constant presence of your team, hand washing practices have been improved. Staff nurse sometimes help us in data entry of high risk patients otherwise it would have been very difficult to complete everything alone. The team finds out the gaps and implemented solutions which are very helpful to us. Regular discussion of the gaps identified was discussed with the staff and they were motivated to perform better in future.	
**14**	What all challenges did you experience with the quality of care project team?<br>No, I didn't find any challenge with your presence.	
**15**	After the quality of care project finishes how the quality improvement efforts can be continued?<br>People should be sensitized about how and why we need to improve the quality of health services. Quality manager and hospital administration department should be given this charge to continue the processes.	
**16**	Any other comments	

Name of Interviewer______________________ Signature________________

1. **ID : 1168201**		
**1.1 Type of Health Facility : DISTRICT HOSPITAL, FBD**	**1.2 Designation : staff nurse**	
**2. General**		
**2.1**	How long have you been working in this health facility? (months/years)	5 years
**2.2**	Total months/years of service	6 years
**2.3**	What are your current roles and responsibility with respect to maternal and neonatal care?<br>I am responsible for record maintenance of delivery and birth, monitor the patient, assist during delivery and any other work related to labor room.	
**3**	What do you mean by quality in the care provided by you at this hospital?<br>Quality means cleanliness in the hospital. It means to provide good treatment to the patient and patient should be happy while leaving the hospital at the time of discharge.	
**4**	In your opinion what all have been done to improve the quality of care?<br>There are many things which are improved like cleanliness and improvement in record keeping. We attended regular trainings for infection control practices and hand hygiene. We also got refreshment training for clinical side which was untouched since we joined. These training sessions should be conducted on regular basis.	
**5**	What do you know about the quality of care project undertaken/ implemented at your hospital?<br>Record maintenance is improved by regular monitoring of the team. We also get regular trainings under this project. The team also takes feedback from the patients when they are going back to their home.	
**6**	What all changes did you see at the hospital?<br>There are many changes like cleanliness, improved record maintenance and improvement in hand washing practices. We get regular supply of hand rub and posters are present on walls to remind us if we forget to wash our hands.	
**7**	What all skill building efforts were done at this facility/ hospital? How that helped you?<br>Regular training sessions were conducted which improved our knowledge and expertise.<br>It helped me in revising and brushing up of my knowledge as well. These trainings are very useful to keep in touch with the latest developments in patient care.	
**8**	What all changes do mean see in the record keeping /case sheet maintenance for the patients?<br>The system of record maintenance has been changed. All the case sheets are maintained and updated now.<br>Some new registers are implemented in the department. Although it increased our workload but it is now very easy to track details of the patients. We now know about the importance of record keeping.	
**9**	What all changes do you see in the disinfection practices in your unit?<br>Hand washing practices are improved due to regular monitoring and availability of hand rub. Trainings provided to staff related to disinfection practices were very helpful. Fumigation is done on regular basis and instruments are kept in delivery trays after sterilization.	
**10**	In your opinion how these changes would help the patients and you?<br>Earlier patients used to complain about secondary infection. Now the percentage is very less. We are also working in an aseptic environment.	
**11**	What more could have done to improve the quality of care for patients at this hospital?<br>There should be adequate number of staff. Single register should be maintained to keep all the records and details of the patient.	
**12**	Has there been any change in the supervision and monitoring at this hospital?<br>Yes, monitoring and supervision more than earlier. Nursing sister and doctors are constantly watching our activities. We are also monitoring vitals of the patients at regular intervals.	
**13**	How have the quality of care project team helped you and other members/ staffs at this hospital?<br>Your team was helpful for us. We got to know about many new things through you. The team helped us in knowing our gaps and helped in finding solutions for that. We got to know about importance of documentation because of you. Regular discussions of the gaps helped us in improving our work.	
**14**	What all challenges did you experience with the quality of care project team?<br>No, I didn’t had any challenges with you or your team.	
**15**	After the quality of care project finishes how the quality improvement efforts can be continued?<br>These processes should be continued. I am not sure who will do this but you should talk to our seniors to carry on these activities on regular basis in future.	
**16**	Any other comments	

Name of Interviewer______________________ Signature________________

1. **ID : 1168202**		
**1.1 Type of Health Facility: District hospital, FBD**		**1.2 Designation:** Staff Nurse
**2. General**		
**2.1**	How long have you been working in this health facility? (months/years)	5 years
**2.2**	Total months/years of service	6 years
**2.3**	What are your current roles and responsibility with respect to maternal and neonatal care?<br>My duties include medicine charge, assessment of babies, baby care, feeding assessment, admission and discharge filing of baby, monitoring of vitals to provide emergency care like resuscitation to the babies.	
**3**	What do you mean by quality in the care provided by you at this hospital?<br>Quality means infection control practices in the department and staff should get regular trainings to increase the work skills.	
**4**	In your opinion what all have been done to improve the quality of care?<br>There are many things changed like regular and good training sessions for us, timely fumigation of SNCU and good overall cleaning of the hospital. There are changes in documentation of records and now all records are updated. KMC have been started and proper hand hygiene practices are being followed.	
**5**	What do you know about the quality of care project undertaken/ implemented at your hospital?<br>We got to learn many things through this project. All the above mentioned changes were initiated by the QOC project.	
**6**	What all changes did you see at the hospital?<br>Overall cleanliness of the hospital is improved. We have all equipment's available and functional.	
**7**	What all skill building efforts were done at this facility/ hospital? How that helped you?<br>Yes, many training were conducted in the facility in past 1 year like infection prevention, improve baby care, clinical topics like hypothermia and hyperthermia. They were very helpful in upgrading our knowledge and skills.	
**8**	What all changes do mean see in the record keeping /case sheet maintenance for the patients?<br>Changes are done like proper charting of vitals, complete follow up records, weight at admission and discharge is mentioned in all case files, counseling and KMC records are maintained. Now we know about the importance of documentation in government set up. Earlier we used to skip certain things in case files.	
**9**	What all changes do you see in the disinfection practices in your unit?<br>Proper hand hygiene practices are followed, three bucket system is available for cleaning, regular fumigation is done in the department, and warmers are now cleaned on daily basis.	
**10**	In your opinion how these changes would help the patients and you?<br>Babies have minimum chances of getting secondary infection. Discharge rate has been increased in past few months.<br>These changes are good for us as well because we also have minimum chance of getting infections.	
**11**	What more could have done to improve the quality of care for patients at this hospital?<br>Yes, some more changes are required like entry of attendants should be restricted and mothers should be encouraged for hand washing practice at home.	
**12**	Has there been any change in the supervision and monitoring at this hospital?<br>Yes, supervision visits are increased now. Our in charge visit regularly to check the entries in the register. NQAS team also visited few months back.	
**13**	How have the quality of care project team helped you and other members/ staffs at this hospital?<br>QOC team was very helpful to us. We were guided about maintaining records and importance of record keeping and timely upgrading. Infection control and hand washing practices were encouraged by the QOC team. Weekly trainings were also very helpful. KMC set up is improved and all records of KMC are now being entered and updated.	
**14**	What all challenges did you experience with the quality of care project team?<br>No, I didn't have any problem. Everything was fine.	
**15**	After the quality of care project finishes how the quality improvement efforts can be continued?<br>We got to know about many new things because of you and your team. We were really benefitted with the activities and processes. These efforts should be continuous. You should come back and continue here only.	
**16**	Any other comments	

Name of Interviewer______________________ Signature_______________

1. **ID: 1168401**		
**1.1 Type of Health Facility : District Hospital , FBD**	**1.2 Designation in : SISTER INCHARGE**	
**2. General**		
**2.1**	How long have you been working in this health facility? (months/years)	10 years
**2.2**	Total months/years of service	30 years
**2.3**	What are your current roles and responsibility with respect to maternal and neonatal care?<br>I am nursing sister here so I supervise all staff nurse, check daily entries in register, check bedding and assure that bed sheets are being changed regularly. I also listen to problems of the patients. I make indent for medicines and prepare daily and monthly reports as well. I maintain all records and registers of patient's data as well as of staff.	
**3**	What do you mean by quality in the care provided by you at this hospital?<br>Quality is not only about medicines and treatment. It also includes clean surrounding and bio medical waste segregation according to the guidelines. Adequate health care providers are also needed for good quality of services.	
**4**	In your opinion what all have been done to improve the quality of care?<br>There are many things which are improved like cleanliness and improvement in record keeping. It also increased our work load but we are getting used to it.	
**5**	What do you know about the quality of care project undertaken/ implemented at your hospital?<br>The QOC project is implemented by your team is running since last one and half year. Record maintenance is improved by regular monitoring of the team. We also get regular trainings under this project.	
**6**	What all changes did you see at the hospital?<br>There are many changes like cleanliness, improved record maintenance, and improvement in hand washing practices.	
**7**	What all skill building efforts were done at this facility/ hospital? How that helped you?<br>Yes, regular training sessions are being conducted in the department which improved the knowledge and expertise of the staff.<br>It helped me in revising and brushing up of my knowledge as well. The last training which I attended was 5 years back hence these refreshment trainings are very helpful.	
**8**	What all changes do mean see in the record keeping /case sheet maintenance for the patients?<br>The system of record maintenance has been changed. It is better than earlier. All the case sheets are maintained and updated now. We are now using delivery and birth register to maintain all delivery related records.	
**9**	What all changes do you see in the disinfection practices in your unit?<br>Hand washing practices are improved due to regular monitoring and availability of handrub and soap. Fumigation is done on regular basis now and bedsheets are also changed on daily basis.	
**10**	In your opinion how these changes would help the patients and you?<br>Earlier patients used to complain about sepsis and episiotomy. At present, the number of patients with sepsis and secondary infection has been reduced.	
**11**	What more could have done to improve the quality of care for patients at this hospital?<br>There should be adequate number of staff. It is difficult to manage quality with handful of staff.	
**12**	Has there been any change in the supervision and monitoring at this hospital?<br>Yes, monitoring and supervision is improved now. I myself do regular supervision and doctors are also doing regular supervision visits to monitor the services.	
**13**	How have the quality of care project team helped you and other members/ staffs at this hospital?<br>Yes, your team was helpful as you came for the good reasons but your staff never assisted me as they were always busy with data collection. Earlier I thought that your staff will strengthen the shortage of staff at the labor room.	
**14**	What all challenges did you experience with the quality of care project team?<br>No, I didn't had any challenges with you or your team.	
**15**	After the quality of care project finishes how the quality improvement efforts can be continued?<br>A designated person should be appointed to check all the activities.	
**16**	Any other comments	

Name of Interviewer_____________________ Signature_______________

ID: 1168301		
**1.1 Type of Health Facility: District hospital, fbd**	**1.2 Designation : class 4**	
**2. General**		
**2.1**	How long have you been working in this health facility? (months/years)	5 years
**2.2**	Total months/years of service	6 years
**2.3**	What are your current roles and responsibility with respect to maternal and neonatal care?<br>My roles and responsibilities include dusting, cleaning of labor room and maternity ward, shifting of patients from labor room to wards and changing clothes of the patients.	
**3**	What do you mean by quality in the care provided by you at this hospital?<br>For me quality means adequate number of staff, regular supply of medicines and equipments. There should be adequate amount of medicines and cotton gauge etc.	
**4**	In your opinion what all have been done to improve the quality of care?<br>Cleanliness is improved in the department. I am not sure about other improvements.	
**5**	What do you know about the quality of care project undertaken/ implemented at your hospital?<br>You people are here to help and guide us. I received training on infection control and hand washing.	
**6**	What all changes did you see at the hospital?<br>Ro is installed in labor room complex for clean drinking water. Earlier it was not there. Color coded dustbins are also available in the wards and labor room. New labor tables are available for delivery. Earlier, all window glasses were broken but now new glasses are fixed in the wards.	
**7**	What all skill building efforts were done at this facility/ hospital? How that helped you?<br>Yes, I received regular training in the hospital. It helped me in many ways like now I know how to protect myself. Now I use gloves and mask while cleaning.	
**8**	What all changes do mean see in the record keeping /case sheet maintenance for the patients?<br>I don't know as I am not responsible for record maintenance.	
**9**	What all changes do you see in the disinfection practices in your unit?<br>Yes, the disinfection practices are improved now. Now we get regular supply of hand rub and hand wash.	
**10**	In your opinion how these changes would help the patients and you?<br>Now the patients get discharge on time. There are less cases of sepsis now.	
**11**	What more could have done to improve the quality of care for patients at this hospital?<br>Improvement is dependent on patients as well like dustbins are available at the designated areas but still some patient's throw garbage near the bed or inside the wards only.	
**12**	Has there been any change in the supervision and monitoring at this hospital?<br>Yes, now doctors and pmo do regular visits and they check the cleanliness of labor rooms and wards.	
**13**	How have the quality of care project team helped you and other members/ staffs at this hospital?<br>Qoc team is very helpful. If we have any problem, we can discuss with you and we get solutions as well.	
**14**	What all challenges did you experience with the quality of care project team?<br>No, I didn't had any challenge with your team.	
**15**	After the quality of care project finishes how the quality improvement efforts can be continued?<br>No one from the hospital will work on improving the quality. Some people or team from outside will have to come for this purpose.	
**16**	Any other comments	

Name of Interviewer______________________ Signature________________

1. **ID: 1168302**		
**1.1 Type of Health Facility: District hospital , fbd**	**1.2 Designation: class 4**	
**2. General**		
**2.1**	How long have you been working in this health facility? (months/years)	5 years
**2.2**	Total months/years of service	9 years
**2.3**	What are your current roles and responsibility with respect to maternal and neonatal care?<br>I am responsible for cleaning and dusting of SNCU. I also clean the radiant warmers after the baby is discharged.	
**3**	What do you mean by quality in the care provided by you at this hospital?<br>For me, quality means clean environment. There should be someone to regularly monitor the cleaning.	
**4**	In your opinion what all have been done to improve the quality of care?<br>Fumigation is done regularly. All the equipment's are cleaned and checked regularly. Proper cleaning is done in SNCU in timely and regular manner.	
**5**	What do you know about the quality of care project undertaken/ implemented at your hospital?<br>QOC team is here to check the quality and to check all the records.	
**6**	What all changes did you see at the hospital?<br>Earlier death rate was very high but now it has been reduced. I think it is because of good cleanliness in the SNCU.	
**7**	What all skill building efforts were done at this facility/ hospital? How that helped you?<br>Yes, I got training on hand hygiene and bio medical waste management. It helped me in many ways. Now I clean my hands before and after doing any work in the department. I also learned about importance of self-cleanliness.	
**8**	What all changes do mean see in the record keeping /case sheet maintenance for the patients?<br>I do not have any work related to record keeping.	
**9**	What all changes do you see in the disinfection practices in your unit?<br>Fumigation is done regularly. We clean all equipment's on daily basis. We also do timely cleaning of oxygen mask, suction tips etc.	
**10**	In your opinion how these changes would help the patients and you?<br>These changes are very helpful for us and for patients as well. Staff and patient will be prevented from any infection if we keep cleanliness in the department.	
**11**	What more could have done to improve the quality of care for patients at this hospital?<br>There is shortage of supply. We do not get material for cleaning regularly. Sometimes we do not get phenyl to clean the floors.	
**12**	Has there been any change in the supervision and monitoring at this hospital?<br>It is done more frequently than before. Earlier less attention was paid on our work but now doctors and senior officers keep regular visits to monitor our work.	
**13**	How have the quality of care project team helped you and other members/ staffs at this hospital?<br>QOC team helped us to know about all right and wrong practices. QOC team also motivated us and gave training about infection control practices.	
**14**	What all challenges did you experience with the quality of care project team?<br>I didn't face any challenge while working with the team.	
**15**	After the quality of care project finishes how the quality improvement efforts can be continued?<br>Doctor should get the charge to continue the processes.	
**16**	Any other comments	

Name of Interviewer______________________ Signature________________

1. **ID : 1168501**		
**1.1 Type of Health Facility : DISTRICT HOSPITAL, FBD**		**1.2 Designation: Quality manager**
**2. General**		
**2.1**	How long have you been working in this health facility? (months/years)	1.6 years
**2.2**	Total months/years of service	3 years
**2.3**	What are your current roles and responsibility with respect to maternal and neonatal care?<br>As a district quality manager, I do periodic visit to the facilities, conduct assessments, check NQAS, kayakalp and lakshya checklist and share the findings with HOD's, PMO and state officials.	
**3**	What do you mean by quality in the care provided by you at this hospital?<br>Quality is about input and output process. It means to provide quality of care to patients according to the SOPs. It also means to receive good level of patient satisfaction.	
**4**	In your opinion what all have been done to improve the quality of care?<br>To improve quality of care, infection control practices have been improved. Apart from that, regular trainings have been given to the staff. We identified the gaps and implemented solutions within given time.	
**5**	What do you know about the quality of care project undertaken/ implemented at your hospital?<br>Quality of care project is being undertaken by INCLEN in collaboration with WHO. The program mainly focuses on labor room, ANC OPD and SNCU by using quality tools. They give training to the staff and also improve the processes.	
**6**	What all changes did you see at the hospital?<br>Up gradation of knowledge and training of staff. Improved hand hygiene and infection control practices. Change in patient's behavior and attitude. Patients are now more satisfied than before.	
**7**	What all skill building efforts were done at this facility/ hospital? How that helped you?<br>Periodic trainings have been given to the staff which includes clinical and non-clinical sections. Staff has been introduced to the quality improvement processes and checklists.<br>It helped me to integrate the staff in a better way for data collection and improved patient care.	
**8**	What all changes do mean see in the record keeping /case sheet maintenance for the patients?<br>Record keeping part has been improved. Now the records are kept in confidential manner and all the records are complete and updated on time e.g. details of partograph, initial assessment and medicine chart.	
**9**	What all changes do you see in the disinfection practices in your unit?<br>Disinfection practices have been improved as well. Hand hygiene practice is improved among staff. Instruments are now regularly sterilized. Regular cleaning of department is in routine process now. Fumigation is done in timely manner.	
**10**	In your opinion how these changes would help the patients and you?<br>These changes will definitely help the patients. Patients go back to home with more satisfaction. They are now treated with respect and dignity and good quality of treatment is being delivered to them. As DQM, these changes will improve the quality outcomes.	
**11**	What more could have done to improve the quality of care for patients at this hospital?<br>For further improvement, continuous good practice should be ensured in the facility and apart from quality assurance, more focus shall be given for quality improvement.	
**12**	Has there been any change in the supervision and monitoring at this hospital?<br>Supervision and monitoring have been improved which includes collection of proper data from all departments, proper survey process.	
**13**	How have the quality of care project team helped you and other members/ staffs at this hospital?<br>QOC team has provided a valuable assistance in maintaining data during NQAS, Kayakalp and Laqshya assessment and also helped in providing refreshment/ training sessions at the facility for the staff.	
**14**	What all challenges did you experience with the quality of care project team?<br>No such challenges were faced while working with the team. Everyone was very cooperative.	
**15**	After the quality of care project finishes how the quality improvement efforts can be continued?<br>The transition of practices started by QOC are discussed with higher authorities and the improvement processes will be continued with the help of HOD and other staff members.	
**16**	Any other comments<br>Wish you all the best.	

Name of Interviewer______________________ Signature________________

1. **ID : 1268101**		
**1.1 Type of Health Facility: FRU 1 , FBD**	**1.2 Designation: Medical officer**	
**2. General**		
**2.1**	How long have you been working in this health facility? (months/years)	8 years
**2.2**	Total months/years of service	10 years
**2.3**	What are your current roles and responsibility with respect to maternal and neonatal care?<br>I am working as a gynecologist here. I do cesareans, monitor normal deliveries and attend pregnant women for ANC checkups.	
**3**	What do you mean by quality in the care provided by you at this hospital?<br>Quality means good hygiene, sanitation and most important thing is patient satisfaction.	
**4**	In your opinion what all have been done to improve the quality of care?<br>Many things are improved now such as hygiene and sanitation, infrastructure is better than earlier. Patients are more satisfied than earlier. Infection control practices are improved.	
**5**	What do you know about the quality of care project undertaken/ implemented at your hospital?<br>QOC project is related to improve infection control services, to check record maintenance system and to know about patient satisfaction rate.	
**6**	What all changes did you see at the hospital?<br>White wash is done. More emphasis is paid on record maintenance. Staff is motivated to provide good quality of health services.	
**7**	What all skill building efforts were done at this facility/ hospital? How that helped you?<br>Training sessions are being conducted on regular basis now. It has improved knowledge level of the staff.	
**8**	What all changes do mean see in the record keeping /case sheet maintenance for the patients?<br>Record maintenance system is much better than earlier. Files are fully completed and partographs are completed with full explanation.	
**9**	What all changes do you see in the disinfection practices in your unit?<br>Huge changes are seen in infection control practices. Staff is regularly washing their hands before and after any procedure. We also check the staff if they are following the infection prevention practices or not.	
**10**	In your opinion how these changes would help the patients and you?<br>Sepsis cases are reduced now. Overall post-delivery complications has been reduced now. Hence it is good for patients.	
**11**	What more could have done to improve the quality of care for patients at this hospital?<br>I would suggest that you people should continue this project. Many changes are being done with your presence. Manpower issues should be highlighted.	
**12**	Has there been any change in the supervision and monitoring at this hospital?<br>Quality consultants from district and state do regular visits. Nodal officers and DIO is also visiting the facility on regular basis.	
**13**	How have the quality of care project team helped you and other members/ staffs at this hospital?<br>QOC team was very much helpful. Everything has been improved. Knowledge of the staff is improved by regular training sessions.	
**14**	What all challenges did you experience with the quality of care project team?<br>No, we were comfortable with your team and didn’t face any challenge.	
**15**	After the quality of care project finishes how the quality improvement efforts can be continued?<br>A designated staff should be appointed for this activity only. High risk cases should be marked by them and gaps should be analyzed by them on routine basis.	
**16**	Any other comments	

Name of Interviewer_____________________ Signature_______________

1. **ID: 1268201**		
**1.1 Type of Health Facility: DH, REWARI**	**1.2 Designation: staff nurse**	
**2. General**		
**2.1**	How long have you been working in this health facility? (months/years)	6 YEARS
**2.2**	Total months/years of service	6 years
**2.3**	What are your current roles and responsibility with respect to maternal and neonatal care?<br>Delivery and BP and record keeping	
**3**	What do you mean by quality in the care provided by you at this hospital?<br>In Facility doing well	
**4**	In your opinion what all have been done to improve the quality of care?<br>In few year back we are doing doing cleaning auto calving BMW and patient facility	
**5**	What do you know about the quality of care project undertaken/ implemented at your hospital?<br>ANC,PNC record keeping in time of discharge we are taking to the patient	
**6**	What all changes did you see at the hospital?<br>In this facility very much change very much change in BMW quality is improve in private wards improve of cleaning .	
**7**	What all skill building efforts were done at this facility/ hospital? How that helped you?<br>BMW	
**8**	What all changes do mean see in the record keeping /case sheet maintenance for the patients?<br>NOW BETTER she follow proper record maintaining she is proper fill pantograph also	
**9**	What all changes do you see in the disinfection practices in your unit?<br>She follow proper step of 5 step of Hand wash and doing autoclave also	
**10**	In your opinion how these changes would help the patients and you?<br>Follow infection steps we are provide home free environment they are not miss our home that's why we are refer and after that we are doing LAMA.	
**11**	What more could have done to improve the quality of care for patients at this hospital?<br>Please provide glauj, gown and cover also.	
**12**	Has there been any change in the supervision and monitoring at this hospital?<br>Many changes after that regular checking of medicine and dustbin and proper supply	
**13**	How have the quality of care project team helped you and other members/ staffs at this hospital?<br>Yes they are doing lots of help because crowd iis very high and they people help lot of us they are doing proper help of Delivery and PNC they are very help full.	
**14**	What all challenges did you experience with the quality of care project team?<br>No problem	
**15**	After the quality of care project finishes how the quality improvement efforts can be continued?<br>We are know this project is over and then she help lot they are prepare report of ANC and PNC also we are not maintain quality.	
**16**	Any other comments	

Name of Interviewer______________________ Signature________________

1. **ID: 1268201**		
**1.1 Type of Health Facility : FRU 1, FBD**	**1.2 Designation: Staff nurse**	
**2. General**		
**2.1**	How long have you been working in this health facility? (months/years)	5 years
**2.2**	Total months/years of service	5 years
**2.3**	What are your current roles and responsibility with respect to maternal and neonatal care?	
**3**	What do you mean by quality in the care provided by you at this hospital?<br>Quality means following infection control practices.	
**4**	In your opinion what all have been done to improve the quality of care?<br>Hand washing and bio medical waste segregation are improved. Mother are educated about care of newborn.	
**5**	What do you know about the quality of care project undertaken/ implemented at your hospital?<br>QOC project is related to skill building and providing training to the staff. It also includes monitoring of record keeping system in the hospital. Basically, main motive of the project is to improve the quality of health services in the hospital.	
**6**	What all changes did you see at the hospital?<br>Many changes are done like cleanliness in the hospital, improvement in record maintenance, solution of infrastructural issues, white wash of the facility.	
**7**	What all skill building efforts were done at this facility/ hospital? How that helped you?<br>My knowledge is improved by the weekly training sessions. Way of working is changed now and we are working in better way now.	
**8**	What all changes do mean see in the record keeping /case sheet maintenance for the patients?<br>There are many changes. Case sheets are now complete. We write all the details of the patients.	
**9**	What all changes do you see in the disinfection practices in your unit?<br>Disinfection practices are very much improved. Hand washing is encouraged and is being practice in routine.	
**10**	In your opinion how these changes would help the patients and you?<br>By these changes, patients are getting discharge early and they are now more satisfied and happy. Chances of getting infection is reduced.	
**11**	What more could have done to improve the quality of care for patients at this hospital?<br>No idea. I think most of the things are done by now. It is important to continue the same system in long run.	
**12**	Has there been any change in the supervision and monitoring at this hospital?<br>Yes, supervision and monitoring is improved. Doctor staff and other district level officers visit the facility on regular basis.	
**13**	How have the quality of care project team helped you and other members/ staffs at this hospital?<br>The system is not simplified and changed because of qoc team. Your team pointed out the gaps and suggested us the need of changes in the processes. Hence it was very helpful.	
**14**	What all challenges did you experience with the quality of care project team?<br>No, we didn't face any challenge.	
**15**	After the quality of care project finishes how the quality improvement efforts can be continued?<br>One dedicated person should be appointed for this work only.	
**16**	Any other comments	

Name of Interviewer______________________ Signature________________

1. **ID 1268202**		
**1.1 Type of Health Facility: DH, Rewari**	**1.2 Designation: Class 4**	
**2. General**		
**2.1**	How long have you been working in this health facility? (months/years)	6 years
**2.2**	Total months/years of service	6 years
**2.3**	What are your current roles and responsibility with respect to maternal and neonatal care?<br>Drug, cleaning, moping in delivery	
**3**	What do you mean by quality in the care provided by you at this hospital?	
**4**	In your opinion what all have been done to improve the quality of care?	
**5**	What do you know about the quality of care project undertaken/ implemented at your hospital?<br>Write on paper and don't know who they wright	
**6**	What all changes did you see at the hospital?<br>Yes all challenges , cleaning in ward is increased certain staff room is separated	
**7**	What all skill building efforts were done at this facility/ hospital? How that helped you?<br>Training of hand hygiene is important.	
**8**	What all changes do mean see in the record keeping /case sheet maintenance for the patients?<br>Don't know	
**9**	What all changes do you see in the disinfection practices in your unit?<br>Yes cleaning all-time 3 time all day	
**10**	In your opinion how these changes would help the patients and you?<br>Patient is happy because of cleaning patient come here because poor people are come here and take free treatment.	
**11**	What more could have done to improve the quality of care for patients at this hospital?<br>We are available	
**12**	Has there been any change in the supervision and monitoring at this hospital?<br>Sister , doctor clock in	
**13**	How have the quality of care project team helped you and other members/ staffs at this hospital?<br>Tell all the things cleaning and hand hygiene	
**14**	What all challenges did you experience with the quality of care project team?<br>No challenge	
**15**	After the quality of care project finishes how the quality improvement efforts can be continued?<br>Sister in charge	
**16**	Any other comments	

Name of Interviewer_____________________ Signature_______________

1. **ID : 1268301**		
**1.1 Type of Health Facility: FRU 1, FBD**	**1.2 Designation: class 4**	
**2. General**		
**2.1**	How long have you been working in this health facility? (months/years)	8 years
**2.2**	Total months/years of service	8 years
**2.3**	What are your current roles and responsibility with respect to maternal and neonatal care?<br>My daily job includes cleaning and dusting of labor room and maternity ward, changing bed sheets, assisting patients in daily care.	
**3**	What do you mean by quality in the care provided by you at this hospital?<br>For me quality means availability of good healthcare services to the patients and adequate staff in the hospital.	
**4**	In your opinion what all have been done to improve the quality of care?<br>Many things have been done. Cleanliness is good now. Clean water is available for the patients. Floor tiles are installed in the maternity ward.	
**5**	What do you know about the quality of care project undertaken/ implemented at your hospital?<br>This project is about improving quality of treatment for the patients. Your team come here and checks the case records. You give us trainings and also check the cleanliness of the hospital.	
**6**	What all changes did you see at the hospital?<br>Hospital is much cleaner than before. White wash is done and floor tiles are installed in the ward. Clean drinking water is available for the patients.	
**7**	What all skill building efforts were done at this facility/ hospital? How that helped you?<br>Weekly trainings were very good. We know more about bio medical waste segregation and infection control practices. It was helpful in gaining knowledge and skills.	
**8**	What all changes do mean see in the record keeping /case sheet maintenance for the patients?<br>I don’t know as I am not responsible for record maintenance.	
**9**	What all changes do you see in the disinfection practices in your unit?<br>Cleanliness is good now. Fumigation is done on regular basis. We get phenyl and bacciloid for cleaning the floors. We now segregate all the bio medical waste properly according to the color coding and also disinfect the bed sheets before sending it for cleaning.	
**10**	In your opinion how these changes would help the patients and you?<br>Patients are now very happy and satisfied with the treatment. They make less complaint now. These changes are good for both of us. We will also be safe from any infections and if patients are satisfied then we also get mental satisfaction.	
**11**	What more could have done to improve the quality of care for patients at this hospital?<br>First of all our salaries should be increase. Supplies should be regular. We should not have to wait for phenyl and other things which are needed for cleaning. Although supplies are now better but still sometimes we face shortage.	
**12**	Has there been any change in the supervision and monitoring at this hospital?<br>Yes, doctor staff do regular rounds to check the cleaning. Cmo and team from panchkula also visit the hospital regularly.	
**13**	How have the quality of care project team helped you and other members/ staffs at this hospital?<br>Yes, your team was very helpful. We get trainings from you and also get to know about new and better ways to do work. We can discuss our problems and issues with you so that you can speak to higher authorities regarding our problems.	
**14**	What all challenges did you experience with the quality of care project team?<br>No, I didn’t had any challenge with your team.	
**15**	After the quality of care project finishes how the quality improvement efforts can be continued?<br>Someone should be given the responsibility to continue these activities. Doctor staff can decide better.	
**16**	Any other comments	

Name of Interviewer______________________ Signature________________

1. **ID : 1268401**		
**1.1 Type of Health Facility ; FRU1, FBD**	**1.2 Designation in** Nursing In charge	
**2. General**		
**2.1**	How long have you been working in this health facility? (months/years)	19 years
**2.2**	Total months/years of service	23 years
**2.3**	What are your current roles and responsibility with respect to maternal and neonatal care?<br>I make duty roaster, managing the department, arrangements, checking file maintain the flies, monitoring supervision.	
**3**	What do you mean by quality in the care provided by you at this hospital?<br>To give all facilities to the patient so that they get best treatment and care.	
**4**	In your opinion what all have been done to improve the quality of care?<br>The facility is cleaned, records are up to date, and trainings have been given to staff regarding infection control and on other important clinical topics to gain the knowledge of all staff.	
**5**	What do you know about the quality of care project undertaken/ implemented at your hospital?<br>The project includes monitoring of processes and checking of files, check the record maintenance system, training to the staff,	
**6**	What all changes did you see at the hospital?<br>Record maintenance has been improved, staff is been monitored and check if they are doing their duties properly or not. Cleanliness of the hospital is improved, regular trainings and meetings are been conducted to discuss any gaps in the facility or any issues with the staff, solutions are implemented within time.	
**7**	What all skill building efforts were done at this facility/ hospital? How that helped you?<br>Various training sessions have been conducted for the staff on weekly basis. It helped the staff in gaining knowledge and expertise.	
**8**	What all changes do mean see in the record keeping /case sheet maintenance for the patients?<br>All records and registers are completed well on time. Case sheets are filled completely. Partographs are complete. Discharge slips are also been filled completely.	
**9**	What all changes do you see in the disinfection practices in your unit?<br>Hand hygiene practices have been improved, instruments are regularly sterilized and labor room is fumigated on weekly basis. Class 4 are regularly cleaning the assigned area and overall cleanliness of the facility is improved.	
**10**	In your opinion how these changes would help the patients and you?<br>This will help in reducing the cases of secondary infection. Patients will be more happy and satisfied.	
**11**	What more could have done to improve the quality of care for patients at this hospital?<br>Staff is inadequate. Workload is more hence we should get at least 3 more staff nurse.	
**12**	Has there been any change in the supervision and monitoring at this hospital?<br>District quality manager is making regular visits to monitor the services. NQAS team also visited the facility for assessment.	
**13**	How have the quality of care project team helped you and other members/ staffs at this hospital?<br>Regular monitoring of the team helped us in improving the gaps. We get to know about importance of completing records. Trainings conducted by the team was also very helpful. Staff is now more confident and motivated.	
**14**	What all challenges did you experience with the quality of care project team?<br>I never faced any challenge while working with your team.	
**15**	After the quality of care project finishes how the quality improvement efforts can be continued?<br>1 dedicated staff to be assigned the duty of maintaining the processes. They can be either from the hospital or any new position shall be open.	
**16**	Any other comments	

Name of Interviewer______________________ Signature________________

1. **ID :1368101**		
**1.1 Type of Health Facility : FRU 2, FBD**	**1.2 Designation : Medical officer**	
**2. General**		
**2.1**	How long have you been working in this health facility? (months/years)	2 years
**2.2**	Total months/years of service	8 years
**2.3**	What are your current roles and responsibility with respect to maternal and neonatal care?<br>I am working as anesthetist. My responsibility is to monitor and performing C/S.	
**3**	What do you mean by quality in the care provided by you at this hospital?<br>Quality of care means giving a better infection free treatment.	
**4**	In your opinion what all have been done to improve the quality of care?<br>Sterilization, hand hygiene practices, fumigation has been improved in past months.	
**5**	What do you know about the quality of care project undertaken/ implemented at your hospital?<br>I do not have much idea as I went on maternity leave for 6 months in between. Nut I know that your team gave trainings to the staff and also monitored the records of labor room.	
**6**	What all changes did you see at the hospital?<br>There are changes in biomedical waste management. Regular visits of officers are being done on routine basis. Overall cleanliness in hospital is improved.	
**7**	What all skill building efforts were done at this facility/ hospital? How that helped you?<br>Training of class iv for hand hygiene practices had shown great changes. Other training sessions for the staff were also good.	
**8**	What all changes do mean see in the record keeping /case sheet maintenance for the patients?<br>Yes, there are huge changes in record keeping. The staff follows and maintains all the records and keeps the records updated. We have a checklist system for fumigation and autoclaving of the instruments.	
**9**	What all changes do you see in the disinfection practices in your unit?<br>Overall cleanliness and hand hygiene practices have been improved. Fumigation is done routinely.	
**10**	In your opinion how these changes would help the patients and you?<br>Both patients and doctors are benefited with these changes. Sepsis is reduced in our facility. Patient care is done in better way.	
**11**	What more could have done to improve the quality of care for patients at this hospital?<br>Swab culture should be performed in the facility on regular basis. Then only we will get to know about the effect of following disinfection practices. Supply of material and equipment’s should be on regular basis.	
**12**	Has there been any change in the supervision and monitoring at this hospital?<br>Yes, there is lot of changes now. More supervision visits are been done in the hospital.	
**13**	How have the quality of care project team helped you and other members/ staffs at this hospital?<br>QOC team is very helpful. The staff from QOC team used to discuss the gaps in the records. You also helped us in finding solutions for the infrastructural issues. Now white washing of the facility has been started which we were demanding since last 3-4 years but you helped us by discussing the issue with CMO.	
**14**	What all challenges did you experience with the quality of care project team?<br>No, I didn’t find any challenge with your presence.	
**15**	After the quality of care project finishes how the quality improvement efforts can be continued?<br>These efforts should continue. We should make a unit which will constantly review the system and processes on quarterly basis.	
**16**	Any other comments	

Name of Interviewer______________________ Signature________________

1. **ID: 1368301**		
**1.1 Type of Health Facility: FRU 3, FBD**	**1.2 Designation: class 4**	
**2. General**		
**2.1**	How long have you been working in this health facility? (months/years)	11 years
**2.2**	Total months/years of service	13 years
**2.3**	What are your current roles and responsibility with respect to maternal and neonatal care?<br>Cleaning and dusting of maternity ward, labor room and surrounding area. Shifting of patients, changing bed sheets.	
**3**	What do you mean by quality in the care provided by you at this hospital?<br>Quality means cleanliness and all patient should get treatment on time.	
**4**	In your opinion what all have been done to improve the quality of care?<br>Many things have been done now. White wash is done and renovation of hospital is under process. Cleanliness is better than before.	
**5**	What do you know about the quality of care project undertaken/ implemented at your hospital?<br>This project is related to improving our hospital. We get suggestions to improve our work and we also get trainings to improve our knowledge.	
**6**	What all changes did you see at the hospital?<br>No blockage of hose pipes, building is maintained, better cleanliness, patients are satisfied.	
**7**	What all skill building efforts were done at this facility/ hospital? How that helped you?<br>We get trainings on weekly basis. These sessions helped us to know about recent changes in guidelines and also helped us in gaining knowledge.	
**8**	What all changes do mean see in the record keeping /case sheet maintenance for the patients?<br>I do not have any work related to record keeping. We just have to sign on a given format after routine cleaning.	
**9**	What all changes do you see in the disinfection practices in your unit?<br>Fumigation is done more frequently; entry of attendants is restricted in labor room, all the instruments are sterilized daily, hand washing practices are improved.	
**10**	In your opinion how these changes would help the patients and you?<br>This will help in controlling any chances of infection from patients to us or to patients through us.	
**11**	What more could have done to improve the quality of care for patients at this hospital?<br>We should get regular supply of phenyl. Sometimes there are no medicines or even cotton in the hospital. Patients have to suffer in that case.	
**12**	Has there been any change in the supervision and monitoring at this hospital?<br>Yes, our incharge come for supervision on regular basis. In past couple of months, several teams from cmo office and panchkula also visited the hospital for supervision. Madam (Nursing sister) is always present to monitor the work done by us. We have to maintain a daily log sheet also.	
**13**	How have the quality of care project team helped you and other members/ staffs at this hospital?<br>Yes, we get so many trainings by you which helped us in gaining knowledge.	
**14**	What all challenges did you experience with the quality of care project team?<br>I never had any challenge.	
**15**	After the quality of care project finishes how the quality improvement efforts can be continued?<br>These efforts should continue. It is better for the patients. I don't know who will do it. Someone from outside have to come because we already have so many things to do and staff is less.	
**16**	Any other comments	

Name of Interviewer______________________ Signature________________

1. **ID : 1368401**		
**1.1 Type of Health Facility: FRU 2 FBD**	**1.2 Designation:** Nursing In charge	
**2. General**		
**2.1**	How long have you been working in this health facility? (months/years)	5 YEARS
**2.2**	Total months/years of service	15 YEARS
**2.3**	What are your current roles and responsibility with respect to maternal and neonatal care?	
**3**	What do you mean by quality in the care provided by you at this hospital?<br>Quality means infection control practices. Patient should get timely treatment and emergencies should be handled well on time.	
**4**	In your opinion what all have been done to improve the quality of care?<br>Many things have been done. Record maintenance is improved. Staff behavior is changed and good towards the patient. Cleanliness and disinfection practices are very much improved. Patients are more satisfied than before.	
**5**	What do you know about the quality of care project undertaken/ implemented at your hospital?<br>QOC team provides training to the staff and do monitoring of our work. QOC team also suggested many changes to improve the system such as use of 7 delivery trays at the labor room.	
**6**	What all changes did you see at the hospital?<br>Infrastructure changes have been done. White washing of the facility is done now. Record keeping system is improved. High risk pregnancies are been marked and high risk pregnant women's are given priorities at the ANC OPD.	
**7**	What all skill building efforts were done at this facility/ hospital? How that helped you?<br>Trainings are done at district level and facility level. QOC team is also involved with the weekly trainings which helped the staff to brush up their knowledge.	
**8**	What all changes do mean see in the record keeping /case sheet maintenance for the patients?<br>Record maintenance had been improved. All details of the patients are available in the files. Partograph is filled for all the patients. Monitoring of files is also done on routine basis to check whether the records are updated or not.	
**9**	What all changes do you see in the disinfection practices in your unit?<br>Now we use disposables like shoe cover and head caps in labor room. Hand washing practices are improved. Regular fumigation of labor room is done on routine basis. Overall cleanliness of the facility is improved. We are now maintaining OT's guidelines for sterilization in labor room.	
**10**	In your opinion how these changes would help the patients and you?<br>Rate of secondary infection is less among patients and hence they are more happy and satisfied. We get mental satisfaction at the end of the day.	
**11**	What more could have done to improve the quality of care for patients at this hospital?<br>More emphasis shall be given to biomedical waste segregation. A security guard should be appointed for the facility. Generator is also needed for regular power supply in summers.	
**12**	Has there been any change in the supervision and monitoring at this hospital?<br>Yes, supervisions and monitoring are being done regularly. Teams are also visiting the facility for assessment of the services.	
**13**	How have the quality of care project team helped you and other members/ staffs at this hospital?<br>QOC team was very helpful for us. They helped us in OPD by segregating high risk patients. QOC team also motivated and encouraged the staff to provide quality health care services to the patients. They helped us in following hand washing practices. Record maintenance system is improved because of pointing the gaps and incomplete records done by our staff. It was indeed very helpful for us.	
**14**	What all challenges did you experience with the quality of care project team?<br>QOC team was very helpful. We didn't face any challenges while working.	
**15**	After the quality of care project finishes how the quality improvement efforts can be continued?<br>These activities should be continued. We do not have sufficient staff to continue this. Hence, either you or any dedicated staff should be permanently appointed at the facility to continue this work. It will be good if these processes will continue.	
**16**	Any other comments	

Name of Interviewer______________________ Signature________________

1. **ID: 2168101**		
**1.1 Type of Health Facility: DH, Rewari**	**1.2 Designation: Medical officer**	
**2. General**		
**2.1**	How long have you been working in this health facility? (months/years)	7 years
**2.2**	Total months/years of service	25 years
**2.3**	What are your current roles and responsibility with respect to maternal and neonatal care?<br>In enter the patient in facility and then back to home and OT also include	
**3**	What do you mean by quality in the care provided by you at this hospital?<br>Quality maintenance and minimize death and enter people to monitoring hand wash facility.	
**4**	In your opinion what all have been done to improve the quality of care?<br>Very important is the main problem is supply not provide sope in 10 days no soap available in facility present time we are seeing overall changes patient are happy is better before some years in this time records are up to date in this time	
**5**	What do you know about the quality of care project undertaken/ implemented at your hospital?<br>Work is very good 2 staff are doing proper monitoring if find any problem in facility so we are solve and discus also in staff also	
**6**	What all changes did you see at the hospital?<br>In 1 week if any problem to this ups and down are held in facility and some very much improve data very much if any problem we discus weekly and we are improve	
**7**	What all skill building efforts were done at this facility/ hospital? How that helped you?<br>Hand washing, Brest feeding are very helpful training are very useful our staff and these trainings are very useful for patients also in our infection control very much controlled.	
**8**	What all changes do mean see in the record keeping /case sheet maintenance for the patients?<br>Very much better for after few years in 3 years people are maintaining records properly.	
**9**	What all changes do you see in the disinfection practices in your unit?<br>We are doing proper hand wash and sterilization this is very helpful for our work	
**10**	In your opinion how these changes would help the patients and you?<br>This is very helpful for patient life breast feeding and other things	
**11**	What more could have done to improve the quality of care for patients at this hospital?<br>Most important thing is medicine and other item provide time. sweeper is not posted then we faces many problem<br>Staff is not sufficient in this facility medicine supply is not regular then we face many problem	
**12**	Has there been any change in the supervision and monitoring at this hospital?<br>Yes in this facility change many things staff is not monitoring regular BP, Temperature but now doing regularly now in monitor PV, Temperature, BP they are regular wrote in reports and maintain records properly.	
**13**	How have the quality of care project team helped you and other members/ staffs at this hospital?<br>Yes people are provide lots of support if any mistake she monitor regularly and discus with staff<br>Then we are held meeting and doing discussion.	
**14**	What all challenges did you experience with the quality of care project team?<br>Thy people are coming outside then people are not interact properly but this time everything is ok now.	
**15**	After the quality of care project finishes how the quality improvement efforts can be continued?<br>The project is finish then we are looking proper changes in this project continue regularly in after 3 months start new project in this facility.	
**16**	Any other comments	

Name of Interviewer______________________ Signature________________

1. **ID:2168401**		
AREA : FRU 1, **REWARI**	Designation: Class 4	
**2. General**		
**2.1**	How long have you been working in this health facility? (months/years)	13 year
**2.2**	Total months/years of service	25 years
**2.3**	What are your current roles and responsibility with respect to maternal and neonatal care?<br>Gyne in charge	
**3**	What do you mean by quality in the care provided by you at this hospital?<br>Hygiene aspects maintenance post-natal and patient satisfy	
**4**	In your opinion what all have been done to improve the quality of care?<br>Regular feedback quickly	
**5**	What do you know about the quality of care project undertaken/ implemented at your hospital?<br>Weekly meetings	
**6**	What all changes did you see at the hospital?<br>Record keeping patient	
**7**	What all skill building efforts were done at this facility/ hospital? How that helped you?<br>Patient provide proper training this is very important.	
**8**	What all changes do mean see in the record keeping /case sheet maintenance for the patients?<br>Data sheet maintenance partograph and PV improved records of mystery imposed now work smartly imposed.	
**9**	What all changes do you see in the disinfection practices in your unit?<br>Regularly ongoing process	
**10**	In your opinion how these changes would help the patients and you?	
**11**	What more could have done to improve the quality of care for patients at this hospital?<br>Scope is always in this work also	
**12**	Has there been any change in the supervision and monitoring at this hospital?<br>We all do	
**13**	How have the quality of care project team helped you and other members/ staffs at this hospital?<br>We are known and regular feedback	
**14**	What all challenges did you experience with the quality of care project team?<br>No challenges as such	
**15**	After the quality of care project finishes how the quality improvement efforts can be continued?<br>**Coming sister will do aspects sister in charge**	
**16**	Any other comments	

Name of Interviewer______________________ Signature________________

1. **ID :** **3168101**			
**1.1 Type of Health Facility: District hospital**		**1.2 Designation: Medical In charge**	
**2. General**			
**2.1**	How long have you been working in this health facility? (months/years)		2 years
**2.2**	Total months/years of service		2 years
**2.3**	What are your current roles and responsibility with respect to maternal and neonatal care?<br>Head of the department of pediatric and SNCU ward. Handle patients in pediatric and SNCU ward and treat the new born babies inborn and out born.		
**3**	What do you mean by quality in the care provided by you at this hospital?<br>Provide all facility to the patient and care to the patient give treatment on time		
**4**	In your opinion what all have been done to improve the quality of care?<br>Proper care was given to the patient, Give treatment on time, patient satisfy, proper management. if important to refer than refer the patient on time and give emergency treatment to the patient		
**5**	What do you know about the quality of care project undertaken/ implemented at your hospital?<br>Yes, I know nurse come to check the files and tell mistakes to the duty nurse which helps in improving file management and daily check cleanliness and hygienic maintain and ask patient there satisfaction and if any gaps then fill the gaps . monitoring the files		
**6**	What all changes did you see at the hospital?<br>Yes changes seen in the documentation and cleanliness improved and SNCU KMC improved and improved in entry on KMC details and improve in missing data of mother on files was improved		
**7**	What all skill building efforts were done at this facility/ hospital? How that helped you?<br>Training were given in routine bases which helps in improving skill of the nurses and training help in improving their knowledge in proper way and can able to gain knowledge in a proper way		
**8**	What all changes do mean see in the record keeping /case sheet maintenance for the patients?<br>Documentation of the patient information is improved and maintaining of the files improved. Some gaps was there was fulfilled by your information. Discharge slip provided to the discharged patient. Improved mothers information documentation		
**9**	What all changes do you see in the disinfection practices in your unit?<br>It is improved. Cleanliness maintained. Improved in mothers hygienic maintaince		
**10**	In your opinion how these changes would help the patients and you?<br>Patient satisfaction was improved. patient discharged happily with cleanliness and bed cleanness		
**11**	What more could have done to improve the quality of care for patients at this hospital?<br>Provide fumigation machine in the hospital		
**12**	Has there been any change in the supervision and monitoring at this hospital?<br>Quality nurse was made which takes daily round supervise the things and provide equipment to the department		
**13**	How have the quality of care project team helped you and other members/ staffs at this hospital?<br>Help in record keeping and managing the data, training the nurses for improve their knowledge and apply there in proper way		
**14**	What all challenges did you experience with the quality of care project team?<br>No challenges faced		
**15**	After the quality of care project finishes how the quality improvement efforts can be continued?<br>It will continue by the staff or sister in charge		
**16**	Any other comments<br><br>No comment		

Name of Interviewer______________________ Signature________________

1. **ID: 3168102**		
**1.1 Type of Health Facility: District hospital**	**1.2 Designation : medical officer**	
**2. General**		
**2.1**	How long have you been working in this health facility? (months/years)	2 years
**2.2**	Total months/years of service	3 years
**2.3**	What are your current roles and responsibility with respect to maternal and neonatal care?<br>Conduct labour deliveries , counselling the patient, OPD of the pregnant women, advice treatment and medicine to the patient, post natal care , newborn care	
**3**	What do you mean by quality in the care provided by you at this hospital?<br>Provide proper care to the patient, give all facility to the patient and care to the patient. Timely treatment. Hygienic free treatment to all patient and our self also	
**4**	In your opinion what all have been done to improve the quality of care?<br>Maintain hygienic and unhygienic free treatment to the patient	
**5**	What do you know about the quality of care project undertaken/ implemented at your hospital?<br>Yes, I know nurse come to check the files and tell mistakes to the duty nurse which helps in improving file management. Weekly round and check partogarh and APGAR Score and train nurses and check knowledge of nurses	
**6**	What all changes did you see at the hospital?<br>Yes changes seen in the documentation and improve in entering of the patient in labour room	
**7**	What all skill building efforts were done at this facility/ hospital? How that helped you?<br>Training were given in routine bases which helps in improving skill of the nurses	
**8**	What all changes do mean see in the record keeping /case sheet maintenance for the patients?<br>Documentation of the patient information is improved and maintaining of the files improved. And instrument complaint register was stared and was well maintained	
**9**	What all changes do you see in the disinfection practices in your unit?<br>Daily routine round was started in each of the department which helps in disposing the waste and there is improvement in hand hygiene	
**10**	In your opinion how these changes would help the patients and you?<br>Patient satisfaction was improved. patient discharged happily	
**11**	What more could have done to improve the quality of care for patients at this hospital?<br>Gynae should come and handle the high risk patient	
**12**	Has there been any change in the supervision and monitoring at this hospital?<br>Daily round and improve the disinfection practices helps in supervision and monitoring improvement	
**13**	How have the quality of care project team helped you and other members/ staffs at this hospital?<br>Help in record keeping and managing the data , cleanliness improves , improved hygienic practices, help in gaining knowledge	
**14**	What all challenges did you experience with the quality of care project team?<br>No problem	
**15**	After the quality of care project finishes how the quality improvement efforts can be continued?<br>Gynae will come and see will see	
**16**	Any other comments<br>It should be continue	

Name of Interviewer______________________ Signature________________

ID : 3168201		
**1.1 Type of Health Facility : DISTRICT HOSPITAL**	**1.2 DESIGNATION : STAFF NURSE**	
**2. General**		
**2.1**	How long have you been working in this health facility? (months/years)	4 YEARS
**2.2**	Total months/years of service	24 years
**2.3**	What are your current roles and responsibility with respect to maternal and neonatal care?<br>Sister in charge make duty roaster, managing the department, arrangements, checking file maintain the flies, monitoring supervision. All over departments , RTI	
**3**	What do you mean by quality in the care provided by you at this hospital?<br>People come to instructional delivery and went happily. Cleanliness hygienic treatment should be given to the patient	
**4**	In your opinion what all have been done to improve the quality of care?<br>Management should be done properly and infection free area	
**5**	What do you know about the quality of care project undertaken/ implemented at your hospital?<br>Files checking , management , patient satisfaction , patient in out timing , training was checking by the nurses mentor she daily come and tell the details and missing things and we correct it	
**6**	What all changes did you see at the hospital?<br>Files improved and complete partograph started	
**7**	What all skill building efforts were done at this facility/ hospital? How that helped you?<br>Training helped in skill improvement and applied their knowledge in skill	
**8**	What all changes do mean see in the record keeping /case sheet maintenance for the patients?<br>Improved in record keeping and files arrangement	
**9**	What all changes do you see in the disinfection practices in your unit?<br>Hand washing improved, cleanliness improved and disposal of waste was started properly	
**10**	In your opinion how these changes would help the patients and you?<br>Start a change in patient satisfaction	
**11**	What more could have done to improve the quality of care for patients at this hospital?<br>All good	
**12**	Has there been any change in the supervision and monitoring at this hospital?<br>Quality manger improve their visit and supervise the things and maintain the cleanness	
**13**	How have the quality of care project team helped you and other members/ staffs at this hospital?<br>Daily see the files help in missing data and help in missing data	
**14**	What all challenges did you experience with the quality of care project team?<br>All good experiences and no problem. First I thing some files will be missed but it not happened	
**15**	After the quality of care project finishes how the quality improvement efforts can be continued?<br>Yes I will do it but it should not finish it should be continue if some supervisor will be there than it will very helpful.	
**16**	Any other comments<br><br>All good you helped a lot in doing a work in proper way so it should be continue	

Name of Interviewer______________________ Signature________________

1. **ID : 3168202**		
**1.1 Type of Health Facility : DISTRICT HOSPITAL**	**1.2 Designation: sister in charge**	
**2. General**		
**2.1**	How long have you been working in this health facility? (months/years)	7 YEARS
**2.2**	Total months/years of service	30 YEARS
**2.3**	What are your current roles and responsibility with respect to maternal and neonatal care?<br>Sister in charge make duty roaster, managing the department, arrangements, checking file maintain the flies, monitoring supervision.	
**3**	What do you mean by quality in the care provided by you at this hospital?<br>Provide facilities to the patient and patient discharge happily	
**4**	In your opinion what all have been done to improve the quality of care?<br>Files management improved and clean the department infection free area	
**5**	What do you know about the quality of care project undertaken/ implemented at your hospital?<br>Files checking , training , monitoring, cleanness , maintaining of register , disposing waste in proper way improved	
**6**	What all changes did you see at the hospital?<br>Files maintained properly and hand hygiene increase , cleaning of the department , AC facility improved and sister gowns was provided and patient gowns was given for each and every patient for breast feeding	
**7**	What all skill building efforts were done at this facility/ hospital? How that helped you?<br>Training helped in skill improvement	
**8**	What all changes do mean see in the record keeping /case sheet maintenance for the patients?<br>Well maintained and gaps we overcome	
**9**	What all changes do you see in the disinfection practices in your unit?<br>Yes hand hygiene , nurses are doing disinfection practices , class 4 a lean properly and maintain the disinfection practices and autoclaving also started	
**10**	In your opinion how these changes would help the patients and you?<br>Patient are very satisfied	
**11**	What more could have done to improve the quality of care for patients at this hospital?<br>Staff is less increase them and fumigation machine is not well	
**12**	Has there been any change in the supervision and monitoring at this hospital?<br>Quality manger improve their visit and supervise the things properly regularly see the files	
**13**	How have the quality of care project team helped you and other members/ staffs at this hospital?<br>Daily see the files help in missing data and help in doctors report filling , hygienic maintain , cleanliness improved KMC department improved	
**14**	What all challenges did you experience with the quality of care project team?<br>All good experiences and no problem	
**15**	After the quality of care project finishes how the quality improvement efforts can be continued?<br>Duty of 1 nurses and class 4 for cleanness	
**16**	Any other comments<br><br>All good	

Name of Interviewer______________________ Signature________________

1. **ID : 3168301**		
**1.1 Type of Health Facility: District hospital**	**1.2 Designation: NURSE**	
**2. General**		
**2.1**	How long have you been working in this health facility? (months/years)	7 YEARS
**2.2**	Total months/years of service	7 years
**2.3**	What are your current roles and responsibility with respect to maternal and neonatal care?<br>Normal delivery, patient handling , treatment given to mothers , handling after post delivery	
**3**	What do you mean by quality in the care provided by you at this hospital?<br>Give care to all patient and all patients should be satisfied	
**4**	In your opinion what all have been done to improve the quality of care?<br>File management, file completion, food quality improved, cleaning of all area improved	
**5**	What do you know about the quality of care project undertaken/ implemented at your hospital?<br>Files complete check, management of the files, patient files completion, register maintain, ask discharged patient satisfaction	
**6**	What all changes did you see at the hospital?<br>Files improved and complete partograph started	
**7**	What all skill building efforts were done at this facility/ hospital? How that helped you?<br>Training helped in skill improvement and applied their knowledge in skill	
**8**	What all changes do mean see in the record keeping /case sheet maintenance for the patients?<br>Improved in record keeping and files arrangement and records keeping and cleaning hygienic maintained in the department	
**9**	What all changes do you see in the disinfection practices in your unit?<br>Sweeper clean the area and autoclave done 3 times by class 4	
**10**	In your opinion how these changes would help the patients and you?<br>Start a change in patient satisfaction and patient attendant is also happy	
**11**	What more could have done to improve the quality of care for patients at this hospital?<br>All good	
**12**	Has there been any change in the supervision and monitoring at this hospital?<br>Sister in charge supervise everything properly and all checked properly and start daily round of sister quality in charge.	
**13**	How have the quality of care project team helped you and other members/ staffs at this hospital?<br>Improving in files and give training to the nurses and ask the patient satisfaction daily check nurses hand washing technique	
**14**	What all challenges did you experience with the quality of care project team?<br>All good experiences and no problem.	
**15**	After the quality of care project finishes how the quality improvement efforts can be continued?<br>Duty will be given	
**16**	Any other comments<br><br>All good	

Name of Interviewer______________________ Signature_______________

1. **ID : 3168302**		
**1.1 Type of Health Facility : District hospital**	**1.2 Designation : Nurse**	
**2. General**		
**2.1**	How long have you been working in this health facility? (months/years)	2 YEARS
**2.2**	Total months/years of service	10 years
**2.3**	What are your current roles and responsibility with respect to maternal and neonatal care?<br>Normal delivery, patient handling , treatment given to mothers , handling after post-delivery, iron sucrose , entries in files	
**3**	What do you mean by quality in the care provided by you at this hospital?<br>Give treatment to the patient with care	
**4**	In your opinion what all have been done to improve the quality of care?<br>Used best techniques, safety of our self and to the patient and by maintaining hygienic	
**5**	What do you know about the quality of care project undertaken/ implemented at your hospital?<br>Yes I know do file checking daily, ask patient about their view about the treatment and hygienic check and give training on different topics	
**6**	What all changes did you see at the hospital?<br>Hand washing and autoclaving is improved and arrange autoclaved instrument is improved	
**7**	What all skill building efforts were done at this facility/ hospital? How that helped you?<br>Training helped in improve our work	
**8**	What all changes do mean see in the record keeping /case sheet maintenance for the patients?<br>Yes record keeping , files managed and any lack of documentation tell to us and we fill the files properly after that	
**9**	What all changes do you see in the disinfection practices in your unit?<br>Daily round which helps in disinfection practices	
**10**	In your opinion how these changes would help the patients and you?<br>Patient discharge properly don't take lama and went happy satisfied	
**11**	What more could have done to improve the quality of care for patients at this hospital?<br>Training is given we applied that technique for improving	
**12**	Has there been any change in the supervision and monitoring at this hospital?<br>Monitoring and supervision done daily in the morning , ward is also maintained	
**13**	How have the quality of care project team helped you and other members/ staffs at this hospital?<br>Yes u helped in improving in files and disinfections practices	
**14**	What all challenges did you experience with the quality of care project team?<br>There was no problem faced all helped a lot in our documentation	
**15**	After the quality of care project finishes how the quality improvement efforts can be continued?<br>Sister in charge help in everything	
**16**	Any other comments<br><br>Best project	

Name of Interviewer______________________ Signature________________

1. **ID: 3168303**		
**1.1 Type of Health Facility : District hospital**	**1.2 Designation :staff nurse**	
**2. General**		
**2.1**	How long have you been working in this health facility? (months/years)	6 YEARS
**2.2**	Total months/years of service	6 YEARS
**2.3**	What are your current roles and responsibility with respect to maternal and neonatal care?<br>Sister managing the department, patient handling , patient treatment, handling of new born babies , in absence of doctors arrange everything , bedding sheet arrange	
**3**	What do you mean by quality in the care provided by you at this hospital?<br>Provide facilities to the patient infection free treatment and handle each and every patient	
**4**	In your opinion what all have been done to improve the quality of care?<br>Files management improved and clean the department infection free area	
**5**	What do you know about the quality of care project undertaken/ implemented at your hospital?<br>Files checking , training , monitoring, cleanliness , maintaining of register , disposing waste in proper way improved, infection check	
**6**	What all changes did you see at the hospital?<br>Files maintained properly and hand hygiene increase , cleaning of the department , AC facility improved and sister gowns was provided and patient gowns was given for each and every patient for breast feeding	
**7**	What all skill building efforts were done at this facility/ hospital? How that helped you?<br>Training given by staff sir, help in improving the skill	
**8**	What all changes do mean see in the record keeping /case sheet maintenance for the patients?<br>Well maintained and gaps we overcome changes are seen in file record keeping	
**9**	What all changes do you see in the disinfection practices in your unit?<br>Gloves was well used for cleaning and other process also , cleaning done 3 times daily, hand washing all 6 steps fallowed at every step	
**10**	In your opinion how these changes would help the patients and you?<br>Patient are very satisfied 9 out of 10	
**11**	What more could have done to improve the quality of care for patients at this hospital?<br>All improved other done by time to time	
**12**	Has there been any change in the supervision and monitoring at this hospital?<br>Quality manger improve their visit and supervise the things properly regularly see the files	
**13**	How have the quality of care project team helped you and other members/ staffs at this hospital?<br>Daily see the files help in missing data and help in doctors report filling , hygienic maintain , cleanliness improved KMC department improved and continues training given	
**14**	What all challenges did you experience with the quality of care project team?<br>All good experiences and no problem	
**15**	After the quality of care project finishes how the quality improvement efforts can be continued?<br>Check by sister in charge	
**16**	Any other comments<br><br>All good no comment	

Name of Interviewer______________________ Signature________________

1. **ID : 3168304**		
**1.1 Type of Health Facility :District hospital**	**1.2 Designation : Nurse**	
**2. General**		
**2.1**	How long have you been working in this health facility? (months/years)	3.6YEARS
**2.2**	Total months/years of service	4.6 YEARS
**2.3**	What are your current roles and responsibility with respect to maternal and neonatal care?<br>Patient treatment neonatal care, patient handling, patient care, counselling of mother for feeding	
**3**	What do you mean by quality in the care provided by you at this hospital?<br>Verbally understand the patient and give treatment timely	
**4**	In your opinion what all have been done to improve the quality of care?<br>Give counselling. Tell them about the patient condition. Tell about patient hygiene and tell mothers personal hygiene	
**5**	What do you know about the quality of care project undertaken/ implemented at your hospital?<br>Admission in- out daily and ask about their satisfaction level, give training and file management and arrangement of files and documentation of files then in proper way and observe the hygienic maintains in the department by nurses and mothers of the babies	
**6**	What all changes did you see at the hospital?<br>Files maintained properly and hand hygiene increase, cleaning of the department, fall ceiling is cleaned now and gowns head caps given to each nurses to wear of infection free area.	
**7**	What all skill building efforts were done at this facility/ hospital? How that helped you?<br>Training given by staff sir, help in improving the skill	
**8**	What all changes do mean see in the record keeping /case sheet maintenance for the patients?<br>Well maintained and gaps we overcome changes are seen in file record keeping	
**9**	What all changes do you see in the disinfection practices in your unit?<br>Improvement in cleaning area , hand hygiene washing techniques improved, gowns and cap was start provided to the nurses which helps in protection of themselves as well as to patients	
**10**	In your opinion how these changes would help the patients and you?<br>Yes , recovering fast	
**11**	What more could have done to improve the quality of care for patients at this hospital?<br>No	
**12**	Has there been any change in the supervision and monitoring at this hospital?<br>Quality manger improve their visit and supervise the things properly	
**13**	How have the quality of care project team helped you and other members/ staffs at this hospital?<br>By giving training helps in improving knowledge and by tell the missing data helps in record keeping and observation the things we can see improve in hygiene maintain in the department.	
**14**	What all challenges did you experience with the quality of care project team?<br>All good experiences and no problem	
**15**	After the quality of care project finishes how the quality improvement efforts can be continued?<br>It will be continue	
**16**	Any other comments<br><br>All good no comment	

Name of Interviewer______________________ Signature________________

1. **ID :3168401**		
**1.1 Type of Health Facility : District hospital**	**1.2 Designation: Class IV**	
**2. General**		
**2.1**	How long have you been working in this health facility? (months/years)	3 YEARS
**2.2**	Total months/years of service	3 YEARS
**2.3**	What are your current roles and responsibility with respect to maternal and neonatal care?<br>Cleaning the area, floor, sometimes helps in the delivery, instrument cleaning autoclaving , disposing waste , enema placed , cleaning fans , cutting gauge piece, and autoclave them and pads autoclave , prepare delivery tray	
**3**	What do you mean by quality in the care provided by you at this hospital?<br>Cleaning all area and provide infection free area	
**4**	In your opinion what all have been done to improve the quality of care?<br>Provide unhygienic care to the patient	
**5**	What do you know about the quality of care project undertaken/ implemented at your hospital?<br>Yes , I know daily come and observe the things and tell about maintaining hygiene and tell following hand washing technique	
**6**	What all changes did you see at the hospital?<br>Yes, improved in labour room, improving in hygiene, started removing outside sleepers outside the room. More hand rubs are provide and phenyl provided on time to time	
**7**	What all skill building efforts were done at this facility/ hospital? How that helped you?<br>Training given is helpful in hand washing and clean the area	
**8**	What all changes do mean see in the record keeping /case sheet maintenance for the patients?<br>Register in started for sending clothes and bed sheet to drycleaner. And cleaning checklist is started	
**9**	What all changes do you see in the disinfection practices in your unit?<br>Yes we cleaning all time and wear gloves while washing	
**10**	In your opinion how these changes would help the patients and you?<br>Yes are patient satisfaction	
**11**	What more could have done to improve the quality of care for patients at this hospital?<br>Cleaning all the things	
**12**	Has there been any change in the supervision and monitoring at this hospital?<br>Yes monitoring all the things buy sister in charge and clean all the things	
**13**	How have the quality of care project team helped you and other members/ staffs at this hospital?<br>By observing the things day to day bases and training given and monitoring the things	
**14**	What all challenges did you experience with the quality of care project team?<br>Good experience	
15	After the quality of care project finishes how the quality improvement efforts can be continued?<br>Duty roaster and daily checklist will make and do the things	
**16**	Any other comments<br>No comments	

Name of Interviewer______________________ Signature_______________

1. **ID :3168402**		
**1.1 Type of Health Facility : District Hospital**		
**DESIGNATION : CLASS IV**		
**2. General**		
**2.1**	How long have you been working in this health facility? (months/years)	5years
**2.2**	Total months/years of service	7 years
**2.3**	What are your current roles and responsibility with respect to maternal and neonatal care?<br>Cleaning the area, floor, instrument cleaning autoclaving , disposing waste , cleaning fans , cutting gauge piece, and autoclave them and pads autoclave ,. Cleaning table of nursing counter cylinder opening give cylinder fill not fill. Give babies to the parents. Changing bed sheets of babies	
**3**	What do you mean by quality in the care provided by you at this hospital?<br>Cleaning all area and providing proper care hygienic free care to the patient	
**4**	In your opinion what all have been done to improve the quality of care?<br>Provide unhygienic care to the patient	
**5**	What do you know about the quality of care project undertaken/ implemented at your hospital?<br>Yes , I know daily come and observe the things and tell about maintaining hygiene and tell following hand washing technique	
**6**	What all changes did you see at the hospital?<br>Yes, improved in SNCU CLEANING, improving in hygiene, started removing outside sleepers outside the room. More hand rubs are provide and phenyl provided on time to time	
**7**	What all skill building efforts were done at this facility/ hospital? How that helped you?<br>Training given is helpful in hand washing and clean the area which help in remembering 6 steps technique properly	
**8**	What all changes do mean see in the record keeping /case sheet maintenance for the patients?<br>Register in started for sending clothes and bed sheet to drycleaner. And cleaning checklist is started	
**9**	What all changes do you see in the disinfection practices in your unit?<br>Yes we cleaning all time and wear gloves while washing and phenyl is provided on time and new mob and provide new bucket and cloths for cleaning(mob)	
**10**	In your opinion how these changes would help the patients and you?<br>Yes are patient satisfaction	
**11**	What more could have done to improve the quality of care for patients at this hospital?<br>Cleaning all the things	
**12**	Has there been any change in the supervision and monitoring at this hospital?<br>Yes monitoring all the things by sister in charge	
**13**	How have the quality of care project team helped you and other members/ staffs at this hospital?<br>By observing the things day to day bases and training given and monitoring the things	
**14**	What all challenges did you experience with the quality of care project team?<br>No problem	
15	After the quality of care project finishes how the quality improvement efforts can be continued?<br>Duty roaster and daily checklist will make and do the things	
**16**	Any other comments<br>No comments	

Name of Interviewer______________________ Signature________________

ID: 3168501		
**1.1 Type of Health Facility :DISTRICT HOSPITAL**	**1.2 Designation : Quality Manager**	
**2. General**		
**2.1**	How long have you been working in this health facility? (months/years)	2 YEARS
**2.2**	Total months/years of service	4 YEARS
**2.3**	What are your current roles and responsibility with respect to maternal and neonatal care?<br>In charge of quality manger and quality assurance	
**3**	What do you mean by quality in the care provided by you at this hospital?<br>Provide proper care to the patient and hygienic treatment to the patient	
**4**	In your opinion what all have been done to improve the quality of care?<br>Many things have been done but because of proper building and place it is not able to done properly	
**5**	What do you know about the quality of care project undertaken/ implemented at your hospital?<br>Yes I know that the project is going on quality of care from the Organization INCLEN covering labour room, SNCU and ANC it's having different cycle and tell changes cycle wise the project was going on with lakshay. And monthly training was given to the nurses and daily observation of files records are seen.	
**6**	What all changes did you see at the hospital?<br>In the starting some changes were seen and some hand hygiene maintain was seen	
**7**	What all skill building efforts were done at this facility/ hospital? How that helped you?<br>Training help nurses to get knowledge and improve in their work.	
**8**	What all changes do mean see in the record keeping /case sheet maintenance for the patients?<br>Partograph and APGAR score entry in files and documentation was improved	
**9**	What all changes do you see in the disinfection practices in your unit?<br>Little improved. Removing shoes outside labor room and SNCU can be seen and gloves used was improved	
**10**	In your opinion how these changes would help the patients and you?<br>Patient satisfaction cannot tell	
**11**	What more could have done to improve the quality of care for patients at this hospital?<br>Infrastructure will improve then only it can improved	
**12**	Has there been any change in the supervision and monitoring at this hospital?<br>Daily round started and monitoring round taken and tell daily see the things and can be than in a proper way	
**13**	How have the quality of care project team helped you and other members/ staffs at this hospital?<br>They there work some gaps was there they was tell by your staff and started to fill those gaps and fill in documentation and make the hospital in better way	
**14**	What all challenges did you experience with the quality of care project team?<br>No problem	
**15**	After the quality of care project finishes how the quality improvement efforts can be continued?<br>Some can be done by me and others will be done by HOD and sister in charge.	
**16**	Any other comments<br>Good project it should be continue	

Name of Interviewer______________________ Signature________________

1. **ID :3268101**		
**1.1 Type of Health Facility : SUB DISTRICT HOSPITAL**	**1.2 DESIGNATION** Gynecologist	
**2. General**		
**2.1**	How long have you been working in this health facility? (months/years)	8 YEARS
**2.2**	Total months/years of service	24 YEARS
**2.3**	What are your current roles and responsibility with respect to maternal and neonatal care?<br>head of the department OF labour room , handle caesarian section, patient handling, Manage high risk patients OPD and give treatment to the patient operative services , counseling to the patient, family planning	
**3**	What do you mean by quality in the care provided by you at this hospital?<br>Provide proper care to the patient , give all facility to the patient and care to the patient by which patient get satisfied	
**4**	In your opinion what all have been done to improve the quality of care?<br>PPC Room is established for family planning. Established different OPD of HRP unit. Postnatal ward started, 24*7 services provided to the postnatal ward patient	
**5**	What do you know about the quality of care project undertaken/ implemented at your hospital?<br>Yes , I know nurse come to check the files and tell mistakes to the duty nurse which helps in improving file management and daily check cleanliness and hygienic maintain and ask patient there satisfaction and if any gaps then fill the gaps	
**6**	What all changes did you see at the hospital?<br>Yes changes seen in the documentation and all the changes are in the positive directions	
**7**	What all skill building efforts were done at this facility/ hospital? How that helped you?<br>Training were given in routine bases which helps in improving skill of the nurses and training help in improving their knowledge in proper way and can able to gain knowledge in a proper way	
**8**	What all changes do mean see in the record keeping /case sheet maintenance for the patients?<br>Documentation of the patient information is improved and maintaining of the files improved. some gaps was their was fulfilled by your information	
**9**	What all changes do you see in the disinfection practices in your unit?<br>Daily routine round was started in each of the department which helps in disposing the waste and there is improvement in hand hygiene	
**10**	In your opinion how these changes would help the patients and you?<br>Patient satisfaction was improved. patient discharged happily with cleanliness and by increase in doctors visit	
**11**	What more could have done to improve the quality of care for patients at this hospital?<br>Need specialized man power	
**12**	Has there been any change in the supervision and monitoring at this hospital?<br>Quality nurse was made which takes daily round and which monitor the things and inform the HOD of the department and improve the disinfection practices of the department	
**13**	How have the quality of care project team helped you and other members/ staffs at this hospital?<br>Help in record keeping and managing the data , cleanliness improves , improved hygienic practices, help in gaining knowledge	
**14**	What all challenges did you experience with the quality of care project team?<br>No challenges faced	
**15**	After the quality of care project finishes how the quality improvement efforts can be continued?<br>It will continue by the staff or sister in charge but this activities should be done time to time	
**16**	Any other comments<br>Activities should be done time to time and project should come that facility will improved in better way	

Name of Interviewer______________________ Signature________________

1. **ID:3268102**		
**1.1 Type of Health Facility : sub district hospital**	**1.2 Designation:** Pediatrician	
**2. General**		
**2.1**	How long have you been working in this health facility? (months/years)	2 years
**2.2**	Total months/years of service	2 years
**2.3**	What are your current roles and responsibility with respect to maternal and neonatal care?<br>Head of the department of pediatric and SNCU ward. handle patients in pediatric and SNCU ward and sometime in the OPD of PAEDIATRIC ward and treat the new born babies and help in new born resuscitation	
**3**	What do you mean by quality in the care provided by you at this hospital?<br>Provide proper care to the patient , give all facility to the patient and care to the patient give treatment on time	
**4**	In your opinion what all have been done to improve the quality of care?<br>Proper care was given to the patient, discharge slip provided to the discharge patient. Give treatment on time	
**5**	What do you know about the quality of care project undertaken/ implemented at your hospital?<br>Yes , I know nurse come to check the files and tell mistakes to the duty nurse which helps in improving file management and daily check cleanliness and hygienic maintain and ask patient there satisfaction and if any gaps then fill the gaps	
**6**	What all changes did you see at the hospital?<br>Yes changes seen in the documentation and cleanliness improved and SNCU KMC improved and counselling improved	
**7**	What all skill building efforts were done at this facility/ hospital? How that helped you?<br>Training were given in routine bases which helps in improving skill of the nurses and training help in improving their knowledge in proper way and can able to gain knowledge in a proper way	
**8**	What all changes do mean see in the record keeping /case sheet maintenance for the patients?<br>Documentation of the patient information is improved and maintaining of the files improved. Some gaps was there was fulfilled by your information. Discharge slip provided to the discharged patient. Improved mothers information documentation	
**9**	What all changes do you see in the disinfection practices in your unit?<br>Daily routine round was started in each of the department which helps in disposing the waste and there is improvement in hand hygiene. Supply on hand rub improved and taps waters supply improved outside the SNCU.	
**10**	In your opinion how these changes would help the patients and you?<br>Patient satisfaction was improved. patient discharged happily with cleanliness and by increase in doctors visit but there is less place for mother whose child is admit	
**11**	What more could have done to improve the quality of care for patients at this hospital?<br>Provide proper instrument and continues check of bmr and check instrument	
**12**	Has there been any change in the supervision and monitoring at this hospital?<br>Quality nurse was made which takes daily round and which monitor the things and inform the HOD of the department and improve the disinfection practices	
**13**	How have the quality of care project team helped you and other members/ staffs at this hospital?<br>Help in record keeping and managing the data , cleanliness improves , improved hygienic practices, help in gaining knowledge	
**14**	What all challenges did you experience with the quality of care project team?<br>No challenges faced	
**15**	After the quality of care project finishes how the quality improvement efforts can be continued?<br>It will continue by the staff or sister in charge	
**16**	Any other comments<br>No comment	

Name of Interviewer______________________ Signature________________

1.**ID : 3268201**		
**1.1 Type of Health Facility: SUB DISTRICT HOSPITAL**	**1.2 Designation: sister incharge**	
**2. General**		
**2.1**	How long have you been working in this health facility? (months/years)	1 years
**2.2**	Total months/years of service	21 years
**2.3**	What are your current roles and responsibility with respect to maternal and neonatal care?<br>Sister in charge make duty roaster, managing the department, arrangements, checking file maintain the flies, monitoring supervision. Data collection, give duties to the nurses.	
**3**	What do you mean by quality in the care provided by you at this hospital?<br>Handle patient timely, infection free	
**4**	In your opinion what all have been done to improve the quality of care?<br>clean the department infection free area, improve files, data improve	
**5**	What do you know about the quality of care project undertaken/ implemented at your hospital?<br>Files checking , training, monitoring, cleanness, maintaining of register, disposing waste in proper way improved ask patient satisfaction and training provide to the nurse	
**6**	What all changes did you see at the hospital?<br>Files maintained properly and hand hygiene increase , manage the register and maintain the family planning register and arrange the trays in a proper way and increase in autoclaving in a proper way	
**7**	What all skill building efforts were done at this facility/ hospital? How that helped you?<br>Training helped in skill improvement	
**8**	What all changes do mean see in the record keeping /case sheet maintenance for the patients?<br>Register maintain and improve in files and maintain the high risk patients register and there records and refers register	
**9**	What all changes do you see in the disinfection practices in your unit?<br>Yes hand hygiene , disposing waste technique is improved	
**10**	In your opinion how these changes would help the patients and you?<br>Patient are very satisfied	
**11**	What more could have done to improve the quality of care for patients at this hospital?<br>Staff is less increase them and nurse improve their behavior and improve their work on time and take the sign in consent forms and addhar register and in birth register	
**12**	Has there been any change in the supervision and monitoring at this hospital?<br>Quality manger improve their visit and supervise the things properly and inform the head of the department	
**13**	How have the quality of care project team helped you and other members/ staffs at this hospital?<br>Daily see the files help in missing data and help in doctors report filling , hygienic maintain , cleanliness improved and posters and helping improving the skills	
**14**	What all challenges did you experience with the quality of care project team?<br>All good experiences and no problem	
**15**	After the quality of care project finishes how the quality improvement efforts can be continued?<br>Duty of 1 nurses and class 4 for cleanliness	
**16**	Any other comments<br><br>All good	

Name of Interviewer_____________________ Signature_______________

1. **ID : 3268202**		
**1.1 Type of Health Facility : SUB DISTRICT HOSPITAL**		**1.2 Designation: sister in charge**
**2. General**		
**2.1**	How long have you been working in this health facility? (months/years)	5-6 months
**2.2**	Total months/years of service	18 years
**2.3**	What are your current roles and responsibility with respect to maternal and neonatal care?<br>Sister in charge make duty roaster, managing the department, data collect, monitoring supervision	
**3**	What do you mean by quality in the care provided by you at this hospital?<br>Give proper services to the patients	
**4**	In your opinion what all have been done to improve the quality of care?<br>Training on time to time	
**5**	What do you know about the quality of care project undertaken/ implemented at your hospital?<br>Files checking, training, monitoring, cleanliness, maintaining of register	
**6**	What all changes did you see at the hospital?<br>Files maintained properly and hand hygiene increase , cleaning of the department , arrange of Almira and keeping the record in Almira and maintain the medicine arrangement	
**7**	What all skill building efforts were done at this facility/ hospital? How that helped you?<br>Training helped in skill improvement	
**8**	What all changes do mean see in the record keeping /case sheet maintenance for the patients?<br>Well maintained and gaps overcome discharge slips was improved and started attached in the files also mother info start filling	
**9**	What all changes do you see in the disinfection practices in your unit?<br>Yes hand hygiene , nurses are doing disinfection practices , class 4 clean properly and maintain the disinfection practices and autoclaving also started	
**10**	In your opinion how these changes would help the patients and you?<br>Patient are very satisfied but less place for mothers whose child is admit	
**11**	What more could have done to improve the quality of care for patients at this hospital?<br>Place for mother and pediatric ward should be different from SNCU, NEBULIZERS should be put in OPD not in pediatric ward	
**12**	Has there been any change in the supervision and monitoring at this hospital?<br>Quality manger improve their visit and supervise the things properly regularly see the files	
**13**	How have the quality of care project team helped you and other members/ staffs at this hospital?<br>Daily see the files help in missing data and help in doctors report filling , hygienic maintain , cleanliness improved KMC department improved	
**14**	What all challenges did you experience with the quality of care project team?<br>All good experiences and no problem	
**15**	After the quality of care project finishes how the quality improvement efforts can be continued?<br>Duty of 1 nurses will see	
**16**	Any other comments	

Name of Interviewer______________________ Signature________________

1. **ID : 3268301**		
**1.1 Type of Health Facility : SUB DISTRICT HOSPITAL**	**1.2 Designation : Staff Nurse**	
**2. General**		
**2.1**	How long have you been working in this health facility? (months/years)	4 YEARS
**2.2**	Total months/years of service	5 years
**2.3**	What are your current roles and responsibility with respect to maternal and neonatal care?<br>Labour room conduct deliveries, give care and treatment after delivery, tell breast feeding initiation, newborn care, files records	
**3**	What do you mean by quality in the care provided by you at this hospital?<br>Better management of files, timely give treatment to the patient	
**4**	In your opinion what all have been done to improve the quality of care?<br>Best care, safety of patients as well as to us, education of family planning, hygienic maintain	
**5**	What do you know about the quality of care project undertaken/ implemented at your hospital?<br>Yes I know do file management, surveillance, supervision, kin observation, infection control	
**6**	What all changes did you see at the hospital?<br>Firstly all things was ignored but now all improvement can be seen, all arrangements improved , improvement in disposal of things	
**7**	What all skill building efforts were done at this facility/ hospital? How that helped you?<br>Training helped in skill improved	
**8**	What all changes do mean see in the record keeping /case sheet maintenance for the patients?<br>Yes record keeping maintained properly, all arrangement is done properly , instrument arranged properly	
**9**	What all changes do you see in the disinfection practices in your unit?<br>Yes, disinfection practices is fallowed all changes can be seen in ward, labour room, PNC room. I hand hygienic practices improved, over all cleanness improved , disposal of things improved	
**10**	In your opinion how these changes would help the patients and you?<br>Patient satisfaction is improved. By hygienic improved which will be helpful to the patient as well as to us.	
**11**	What more could have done to improve the quality of care for patients at this hospital?<br>Sister in charges should change if not change than she should be calm and polite. Safety of nurses should be improved	
**12**	Has there been any change in the supervision and monitoring at this hospital?<br>Daily round started which helps a lot in monitoring and supervise the things properly. It should be continue.	
**13**	How have the quality of care project team helped you and other members/ staffs at this hospital?<br>Supervision and monitoring the things and observe the things which helps in do the work in proper way and timely.<br>Managing and treating the patient timely	
**14**	What all challenges did you experience with the quality of care project team?<br>There was no problem faced.	
**15**	After the quality of care project finishes how the quality improvement efforts can be continued?<br>Duty of staff will see everything and sister in charge will check	
**16**	Any other comments<br><br>Medicine set up is done properly	

Name of Interviewer______________________ Signature________________

1. **ID : 3268302**		
**1.1 Type of Health Facility: SUB-DISTRICT HOSPITAL**	**1.2 Designation: Staff nurse**	
**2. General**		
**2.1**	How long have you been working in this health facility? (months/years)	6 YEARS
**2.2**	Total months/years of service	9 years
**2.3**	What are your current roles and responsibility with respect to maternal and neonatal care?<br>Normal delivery, patient handling, treatment given to mothers, handling after post-delivery, iron sucrose, entries in files, post natal care, post operation care, in charge of medicines	
**3**	What do you mean by quality in the care provided by you at this hospital?<br>Infection free treatment to the patient , satisfy the patient	
**4**	In your opinion what all have been done to improve the quality of care?<br>Used best techniques , safety of our self and to the patient and by maintaining hygienic	
**5**	What do you know about the quality of care project undertaken/ implemented at your hospital?<br>Yes I know do file checking daily, files arrangements of files , cleanliness , observation patients , satisfaction seen	
**6**	What all changes did you see at the hospital?<br>Yes very improved in files and cleanliness and arrangements of medicine draws and labeling the rows of medicines	
**7**	What all skill building efforts were done at this facility/ hospital? How that helped you?<br>Training helped in improve our work	
**8**	What all changes do mean see in the record keeping /case sheet maintenance for the patients?<br>Yes record keeping , files managed and data collection and information of patient was documented properly	
**9**	What all changes do you see in the disinfection practices in your unit?<br>Yes, disinfection practices is fallowed all changes can be seen in ward, labour room, PNC room. In all area disinfection practices was fallowed	
**10**	In your opinion how these changes would help the patients and you?<br>Patient satisfaction is improved. By hygienic improved which will be helpful to the patient as well as to us. Proper disposing of the waste is improved which help in maintain hygienic in the area and which help to us also	
**11**	What more could have done to improve the quality of care for patients at this hospital?<br>All improved and other which is remaining will improved by time to time	
**12**	Has there been any change in the supervision and monitoring at this hospital?<br>Disposal of waste material improved and all staff understand their duty and weekly training is started which help in maintaining hygiene in the department. Staff understand their duty. Daily round started which helps a lot in monitoring and supervise the things properly	
**13**	How have the quality of care project team helped you and other members/ staffs at this hospital?<br>Yes , a lot in files improvement, files documentation, started high risk pregnant women register in department which helps in getting know about the total number of patient come of high risk in the department	
**14**	What all challenges did you experience with the quality of care project team?<br>There was no problem faced	
**15**	After the quality of care project finishes how the quality improvement efforts can be continued?<br>Duty of staff will see everything and sister in charge will see and then send the files	
**16**	Any other comments<br>No comment	

Name of Interviewer______________________ Signature________________

1. **ID :3268303**		
**1.1 Type of Health Facility : District Hospital**	**1.2 Designation : staff nurse**	
**2. General**		
**2.1**	How long have you been working in this health facility? (months/years)	7 YEARS
**2.2**	Total months/years of service	9 years
**2.3**	What are your current roles and responsibility with respect to maternal and neonatal care?<br>Monthly report, medicine in charge, patient handling, resuscitation, counselling, KMC and neonatal care to the patient. and handle pediatric ward also	
**3**	What do you mean by quality in the care provided by you at this hospital?<br>Infection free treatment to the patient, satisfy the patient and breast feeding counselling which is very for new born	
**4**	In your opinion what all have been done to improve the quality of care?<br>Used best techniques, safety of our self and to the patient and by maintaining hygienic. improve files and knowledge improve by which we give proper care to the patient	
**5**	What do you know about the quality of care project undertaken/ implemented at your hospital?<br>Yes I know do file checking daily, files arrangements of files, cleanliness, observation patients, satisfaction seen	
**6**	What all changes did you see at the hospital?<br>Files data was arranged in a proper way, files entry improved, start giving discharge slip and because doctors present timely given treatment to the patient	
**7**	What all skill building efforts were done at this facility/ hospital? How that helped you?<br>Training helped in improve our work, knowledge improved which helps in handling patient in a better way	
**8**	What all changes do mean see in the record keeping /case sheet maintenance for the patients?<br>Yes record keeping, files managed and data collection and information of patient mother was entered properly.	
**9**	What all changes do you see in the disinfection practices in your unit?<br>Yes, disinfection practices is fallowed all changes can be seen in ward, daily cleaning 3 times was done and weekly cleaning of the floors done and monthly fumigation started.. Improved in hand washing of nurses, doctors and patients increased.	
**10**	In your opinion how these changes would help the patients and you?<br>Patient satisfaction is improved. By hygienic improved which will be helpful to the patient as well as to us. Proper disposing of the waste is improved which help in maintain hygienic in the area and which help to us also.	
**11**	What more could have done to improve the quality of care for patients at this hospital?<br>Doctors should be available on time<br>All improved and other which is remaining will improved by time to time	
**12**	Has there been any change in the supervision and monitoring at this hospital?<br>Disposal of waste material improved. Daily round started which helps a lot in monitoring and supervise the things properly and advice class 4 for cleaning and round helps in seeing the expiry date of the medicines	
**13**	How have the quality of care project team helped you and other members/ staffs at this hospital?<br>Yes, a lot in files improvement, files documentation, discharge slips, bio-waste is done properly and oxygen cylinder utilization check properly.	
**14**	What all challenges did you experience with the quality of care project team?<br>There was no problem faced	
**15**	After the quality of care project finishes how the quality improvement efforts can be continued?<br>Continue with duty staff nurses with sister in charge	
**16**	Any other comments<br>It is Continue process	

Name of Interviewer______________________ Signature________________

1. **ID : 3268304**		
**1.1 Type of Health Facility : SUB DISTRICT HOSPITAL**	**1.2 Designation : Nurse**	
**2. General**		
**2.1**	How long have you been working in this health facility? (months/years)	3year
**2.2**	Total months/years of service	10 years
**2.3**	What are your current roles and responsibility with respect to maternal and neonatal care?<br>Handle paedtric and SNCU department, patient handling , counselling of the patient	
**3**	What do you mean by quality in the care provided by you at this hospital?<br>Give facilities to the patient increase moral supports to the patients	
**4**	In your opinion what all have been done to improve the quality of care?<br>Give moral supports	
**5**	What do you know about the quality of care project undertaken/ implemented at your hospital?<br>Files checking, training, monitoring, cleanliness, maintaining of register. Daily observations	
**6**	What all changes did you see at the hospital?<br>Files maintained properly and hand hygiene increase , cleaning of the department , arrange of Almira and keeping the record in Almira and maintain the medicine arrangement	
**7**	What all skill building efforts were done at this facility/ hospital? How that helped you?<br>Training helped in skill improvement applying all knowledge	
**8**	What all changes do mean see in the record keeping /case sheet maintenance for the patients?<br>Improving in maintaining files records and keeping in a proper way.	
**9**	What all changes do you see in the disinfection practices in your unit?<br>Yes hand hygiene , nurses are doing disinfection practices , class 4 clean properly and maintain the disinfection practices and autoclaving also started properly timely	
**10**	In your opinion how these changes would help the patients and you?<br>Patient are very satisfied	
**11**	What more could have done to improve the quality of care for patients at this hospital?<br>Give all efforts	
**12**	Has there been any change in the supervision and monitoring at this hospital?<br>Quality manger improve their visit and supervise the things properly	
**13**	How have the quality of care project team helped you and other members/ staffs at this hospital?<br>Daily see the files help in missing data and help in doctors report filling, hygienic maintain, cleanliness improved. training to us so that we apply knowledge on a better way	
**14**	What all challenges did you experience with the quality of care project team?<br>All good experiences and no problem	
**15**	After the quality of care project finishes how the quality improvement efforts can be continued?<br>Already improved a lot others continues with time. Nurses should take punctual responsibility	
**16**	Any other comments<br><br>No comment	

Name of Interviewer_____________________ Signature_______________

1. **ID : 3268401**		
**1.1 Type of Health Facility : SUB DISTRICT HOSPITAL**		**1.2 Respondent Qualification : CLASS 1V**
**2. General**		
**2.1**	How long have you been working in this health facility? (months/years)	4 months
**2.2**	Total months/years of service	4 months
**2.3**	What are your current roles and responsibility with respect to maternal and neonatal care?<br>Cleaning the area, floor, sometimes helps in the delivery, instrument cleaning autoclaving , disposing waste , enema placed , cleaning fans , cutting gauge piece, and autoclave them and pads autoclave , prepare delivery tray. Cleaning table of nursing counter	
**3**	What do you mean by quality in the care provided by you at this hospital?<br>Cleaning all area and provide infection free area	
**4**	In your opinion what all have been done to improve the quality of care?<br>Provide unhygienic care to the patient	
**5**	What do you know about the quality of care project undertaken/ implemented at your hospital?<br>Yes , I know daily come and observe the things and tell about maintaining hygiene and tell following hand washing technique	
**6**	What all changes did you see at the hospital?<br>Yes, improved in labour room, improving in hygiene, started removing outside sleepers outside the room. More hand rubs are provide and phenyl provided on time to time	
**7**	What all skill building efforts were done at this facility/ hospital? How that helped you?<br>Training given is helpful in hand washing and clean the area	
**8**	What all changes do mean see in the record keeping /case sheet maintenance for the patients?<br>Register in started for sending clothes and bed sheet to drycleaner. And cleaning checklist is started	
**9**	What all changes do you see in the disinfection practices in your unit?<br>Yes we cleaning all time and wear gloves while washing and phenyl is provided on time and new mob and provide new bucket and cloths for cleaning(mob)	
**10**	In your opinion how these changes would help the patients and you?<br>Yes are patient satisfaction	
**11**	What more could have done to improve the quality of care for patients at this hospital?<br>Cleaning all the things	
**12**	Has there been any change in the supervision and monitoring at this hospital?<br>Yes monitoring all the things by sister in charge	
**13**	How have the quality of care project team helped you and other members/ staffs at this hospital?<br>By observing the things day to day bases and training given and monitoring the things	
**14**	What all challenges did you experience with the quality of care project team?<br>No problem	
15	After the quality of care project finishes how the quality improvement efforts can be continued?<br>Duty roaster and daily checklist will make and do the things	
**16**	Any other comments<br>No comments	

Name of Interviewer______________________ Signature________________

1. **ID:**	**3268402**	
**1.1 Type of Health Facility: Sub-district hospital**	**1.2 Respondent Qualification : Class IV**	
**2. General**		
**2.1**	How long have you been working in this health facility? (months/years)	5years
**2.2**	Total months/years of service	5years
**2.3**	What are your current roles and responsibility with respect to maternal and neonatal care?<br>Sweeper cum class 4 cum attendant , patient nebulizing , cylinder change, dusting, cleaning, bed sheet change, patient call for breast feeding	
**3**	What do you mean by quality in the care provided by you at this hospital?<br>Cleaning all area and providing proper care and stop infection	
**4**	In your opinion what all have been done to improve the quality of care?<br>Provide unhygienic care to the patient by proper cleaning and hand washing	
**5**	What do you know about the quality of care project undertaken/ implemented at your hospital?<br>Yes , I know daily come and observe the things and tell about maintaining hygiene and tell following hand washing technique	
**6**	What all changes did you see at the hospital?<br>Yes, improved in SNCU cleaning, improving in hygiene, started removing outside sleepers outside the room. More hand rubs are provide and phenyl provided on time to time new mob is provided	
**7**	What all skill building efforts were done at this facility/ hospital? How that helped you?<br>Training given is helpful in hand washing and clean the area which help in remembering 6 steps technique properly	
**8**	What all changes do mean see in the record keeping /case sheet maintenance for the patients?<br>Register in started for sending clothes and bed sheet to drycleaner. And cleaning checklist is started	
**9**	What all changes do you see in the disinfection practices in your unit?<br>Yes we cleaning all time and wear gloves while washing and phenyl is provided on time and new mob provided	
**10**	In your opinion how these changes would help the patients and you?<br>Yes are patient satisfaction	
**11**	What more could have done to improve the quality of care for patients at this hospital?<br>Cleaning all the things	
**12**	Has there been any change in the supervision and monitoring at this hospital?<br>Yes monitoring all the things by sister in charge	
**13**	How have the quality of care project team helped you and other members/ staffs at this hospital?<br>By observing the things day to day bases and training given and monitoring the things and helped in telling the right way how to do the work in a proper way	
**14**	What all challenges did you experience with the quality of care project team?<br>No problem. All good	
15	After the quality of care project finishes how the quality improvement efforts can be continued?<br>checklist will make and do the things	
**16**	Any other comments<br>After doctors came all the things are much better	

Name of Interviewer______________________ Signature________________

1. **ID: 3368301**		
**1.1 Type of Health Facility : FRU 2**	**1.2 Designation : Nurse**	
**2. General**		
**2.1**	How long have you been working in this health facility? (months/years)	5YEARS
**2.2**	Total months/years of service	10 years
**2.3**	What are your current roles and responsibility with respect to maternal and neonatal care?<br>Normal delivery, patient handling, treatment given to mothers, give medicine to mothers and new born handling after post-delivery, iron sucrose , entries in files. ANC OPD also handle by me.	
**3**	What do you mean by quality in the care provided by you at this hospital?<br>Give treatment to the patient with care and timely	
**4**	In your opinion what all have been done to improve the quality of care?<br>Provided LMO Doctors 24hrs and staff nurses and provide canteen for patient and establish emergency department	
**5**	What do you know about the quality of care project undertaken/ implemented at your hospital?<br>Yes I know do file checking tell file documentation, ask patient about their view about the treatment and hygienic check and give training on different topics	
**6**	What all changes did you see at the hospital?<br>Hand washing and hygiene maintain in the hospital started and files maintain stated or improved also.	
**7**	What all skill building efforts were done at this facility/ hospital? How that helped you?<br>Training helped in improve our work and should be given time to time	
**8**	What all changes do mean see in the record keeping /case sheet maintenance for the patients?<br>Yes record keeping, files managed and any lack of documentation tell to us and we fill the files properly and documentation done properly. first we left many columns but after your help we start documenting each and every thing	
**9**	What all changes do you see in the disinfection practices in your unit?<br>Hand hygienic improved … everything taken be carefully for patient and for our self also	
**10**	In your opinion how these changes would help the patients and you?<br>Patient discharge properly don’t take lama and went happy satisfied	
**11**	What more could have done to improve the quality of care for patients at this hospital?<br>Medicine should be give good quality and medicine should be provide from the facility to each and every patient and doctors should available on time to time	
**12**	Has there been any change in the supervision and monitoring at this hospital?<br>Doctors should be available 24hrs by which supervision can be done properly	
**13**	How have the quality of care project team helped you and other members/ staffs at this hospital?<br>Yes u helped in improving in files and disinfections practices but please send the request at high level of requirements of doctors and nurses	
**14**	What all challenges did you experience with the quality of care project team?<br>There was no problem , we are happy by your work	
**15**	After the quality of care project finishes how the quality improvement efforts can be continued?<br>Improvement is required it should be continue and it will be continue by staff	
**16**	Any other comments<br><br>Project should run	

Name of Interviewer______________________ Signature________________
