# Supplementary material for: Improving quality of care for pregnancy, perinatal and newborn care at district and sub-district public health facilities in three districts of Haryana, India: An Implementation study
Source: PLoS One. 2021 Jul 23;16(7):e0254781. doi: 10.1371/journal.pone.0254781 (PMC8301676; doi:10.1371/journal.pone.0254781)
Supplement: S3 File — (PDF) [file pone.0254781.s020.pdf]

UNIQUE ID

Implementation Research on management of Possible Serious Bacterial Infection in young infants where referral is not feasible

<u>DH Assessment tool</u>

**SECTION I: Assessment Information**<br>(write the number of correct answer inside the box)	
I. Instrument Number:	II. Team Member Code:
III. Parcel ID :	IV. Unique facility identification:
V Block Name: ……………………………	VI Village Name: ………………………………
VII Location /Add……………………………	VIII Date of Survey [ ][ ][ ][ ] 2 0 1 7
IX Start Time:	X End Time:

Guidelines for interviewers

- Greet the hospital authority and staffs in respective areas/facilities and introduce yourself.
- Explain the purpose of the assessment.
- Please introduce each question separately and record the response.
- For some section, the suppository documents may be needed, please collect and append, if necessary.
- Marking the response.

SECTION-A: GENERAL INFORMATION

1. Facility Identification

S. no	Question	Response	Comments
1.1	Name of facility:……………………		
1.2	Is the facilty easily accessible?	☐ Yes ☐ No	
1.3	Is the approach road to the facility metalled ?	☐ Yes ☐ No	
1.4	Is the condition of approach road is good?		
1.5	What is the locality where this facility located:<br>(Multiple options can be possible)	Residential area ☐<br>Business/market area ☐<br>Institutional area ☐<br>Far from residential/business area ☐<br>Any other ☐<br>*If other, mention:*	
1.6	Does the facility have a transport facility for referral of mothers and newborns?	☐ Yes ☐ No	

2. General Infrastructure

Q. No.	Question	Response	Comments
2.1	Is this facility open 24 x 7 for the public?	☐ Yes ☐ No	
2.2	**Whether the facility has an enquiry/Help desk?**	☐ Yes ☐ No	
2.3	**Whether the citizen charter is displayed at prominent place in this facility?**	☐ Yes ☐ No	
2.4	**Does the health facility have a separate emergency department?**	☐ Yes ☐ No	
2.5	**Is it open 24x7?**		
2.6	**If not, what hours is it open?**	**________Hrs**	
2.7	Does the health facility have separate labour room?	☐ Yes ☐ No	
2.8	Is the labour room open 24 hours	☐ Yes ☐ No	
2.9	If not then for how many hours a day does it open	_______ hrs	
2.10	Does the health facility have an Intensive care Unit (ICU) for managing critically ill obstetric patients	☐ Yes ☐ No	
2.11	Does the health facility have a ward (maternity ward) for admitting obstetrics patients	☐ Yes ☐ No	
2.12	Is the obstetrical outpatient department separate from the adult outpatient department?	☐ Yes ☐ No	
2.13	At what time does the obstetrical outpatient department open and close?	Opens - ☐☐:☐☐ AM/PM<br>Closes - ☐☐:☐☐ AM/PM	
2.14	Does the health facility have an operation theatre?	☐ Yes ☐ No	

**2.15**	If yes, is the operation theatre available 24 hours?	☐ Yes ☐ No	
**2.16**	If not, what hours is it open?	__________ Hrs	
**2.17**	**If not functional, what is the reason?**		
**2.18**	Does the health facility provide zero expense treatment to the BPL cardholders	☐ Yes ☐ No	
**2.19**	Does this facility have a Functional Blood Bank?	☐ Yes ☐ No	
**2.20**	Does the Blood bank have facility of blood collection and storage?	☐ Yes ☐ No	
**2.21**	Does the Blood Bank have facility for Blood Components separation?	☐ Yes ☐ No	
**2.22**	Does the facility have provisions of Janani Suraksha Yojana (JSY) and monetory incentives are given to the patients?	☐ Yes ☐ No	
**2.23**	Does the facility have provisions of Janani Shishu Suraksha Karyakram (JSSK)- Free drugs, diagnostics, diet, consumables given to pregnant female and newborns	☐ Yes ☐ No	
**2.24**	Number of beneficiaries in last 3 months	☐☐☐☐	
**2.25**	Does the facility have entitlements under JSY & JSSK displayed prominently in the hospital	☐ Yes ☐ No	
**2.26**	Does the facility have Central Sterilization Supply Department (CSSD)?	☐ Yes ☐ No	
**2.27**	Does the facility provide diets to admitted patients during day and night?	☐ Yes ☐ No	
**2.28**	Does the facility have displayed IEC / BCC materials/ for educating patients & visitors regarding pregnancy and/or postnatal care?	☐ Yes ☐ No	
**2.29**	Does the facility provide and update the "Mother and Child Protection Card" for care of mothers and neonates?	☐ Yes ☐ No	
**2.30**	Does the hospital have a functional RKS with its meeting held at prescribed interval and minutes of meeting are recorded?	☐ Yes ☐ No	
**2.31**	Is there a separate functioning fridge available for drugs or vaccines in labour room and pharmacy?	☐ Yes ☐ No	
**2.32**	Is there a complaints box on the hospital premises or a formal way patients can communicate with the hospital?	☐ Yes ☐ No	
**2.33**	Is there appropriate mechanism in place to contact duty doctor? (On telephone/stays nearby)	☐ Yes ☐ No	
**Does this facility have the following services?**			
**2.35**	**Functional Integrated counseling and testing center (ICTC)**	☐ Yes ☐ No	

**2.36**	**Functional Antiretroviral therapy(ART)**	☐ Yes ☐ No	
**2.37**	**Functional Directly observed treatment short course(DOTS) Center**	☐ Yes ☐ No	
**2.38**	Is the stretcher facility available?	☐ Yes ☐ No	
**2.39**	Is the wheel chair facility available?	☐ Yes ☐ No	
**2.40**	Is ramp access available?	☐ Yes ☐ No	
**2.41**	Whether directions and signage's present?	☐ Yes ☐ No	
**2.42**	Is there adequate separate waiting area for attendants of admitted patients?	☐ Yes ☐ No	
**(i)**	**Electricity**		
**2.43**	Does the facilities have 24 x 7 initerrupted electricity supply?	☐ Yes ☐ No	
**2.44**	Is the power cut predictable?	☐ Yes ☐ No	
**2.45**	What is the average hour for power cut in a day?	______hrs/day	
**2.46**	Does the facilities have backup source for the uninterrupted supply of electrical power?	☐ Yes ☐ No	
**2.47**	Did you have uninterupted power supply during the last one month?	☐ Yes ☐ No	
**2.48**	If NO , how many days?	☐☐ Days	
**(ii)**	**Water supply**		
**2.49**	Does this facility have 24 x 7 water supply for functions ?	☐ Yes ☐ No	
**2.50**	Did you have uninterupted water supply during the last one month?	☐ Yes ☐ No	
**2.51**	If No, how many days?	☐☐ Days	
**2.52**	Does the facility have appropriate and functional hand washing facility in the patient care area?	☐ Yes ☐ No	
**2.53**	Does the water supply in the facility is supported with overhead water tank and pump?	☐ Yes ☐ No	
**(iii)**	**Cleanliness**		
**2.54**	Is there Separate functional Public Utilities for Male and Female in waiting area ?	☐ Yes ☐ No	
**2.55**	Is the overall cleaniness of the toilets good in waiting area?	☐ Yes ☐ No	
**2.56**	Is the overall cleaniness of the waiting area good?	☐ Yes ☐ No	

3. Staffing

(i) Availability of staff in the facility

SL No	Type of staff	A. Sanctioned number	B. In-position number	C. Number During any shift on weekday			D. On holiday	E. Any deficiency in (OT/LR/Ward/OPD (Yes/No)
				M	E	N		
3.1.	Chief medical officer							
3.2.	Medical officers							
	Paediatrician							
	Obstetrician							
	General surgeon							
	Medicine specialist							
	Orthopaedic							
	ENT							
	Dentist							
	Other specialists							
3.3.	Doctors							
3.4.	Staff nurse							
3.5.	Physiotherapist							
3.6.	Storekeeper							
3.7.	Radiographer							
3.8.	Dietician							
3.9.	Counsellor							
3.10.	O.T technician							
3.11.	CSSD Asstt.							
3.12.	Pharmacist							
3.13.	Laboratory Technician							
3.14.	CC & Vaccine Logistic Assistant							
3.15.	Dental technician							

4. **Hospital health statistics**

(i) Maternal health statistics

Q. No.	Questions	Response	
		Last year	Last 3 months
4.1	Number of deliveries (total)		
4.2	Number of live births		
4.3	Number of still births (total)		
4.4	Number of MTPs (total)		
4.5	Number of spontaneous abortions		
4.6	Number of septic abortions		
4.7	Number of Caesarean section deliveries		
4.8	Number of Referrals received for maternal cases		
4.9	Number of maternal deaths in hospital		
4.10	Three major causes of maternal deaths? (Specify reasons and numbers due to the cause in last 1 year)	____________(No:)<br>____________(No:)<br>____________(No:)	____________(No:)<br>____________(No:)<br>____________(No:)

(ii) Newborn Health Statistics:

S. No.	Questions	Last Year	Last 3 months
4.11	Number of low birth weight newborn babies (<2500g)		
4.12	Number of pre-term deliveries<37completed weeks		
4.13	Number of babies who required resuscitation (bag and mask ventilation)		
4.14	Number of neonatal deaths in the hospital		
4.15	Number of perinatal deaths (number of still births plus neonatal deaths in hospital)		
4.16	Number of Referrals received for neonatal cases		
4.17	Three major causes of neonatal deaths? (Specify reasons and numbers due to cause in last 1 year)	____________(No:)<br>____________(No:)<br>____________(No:)	____________(No:)<br>____________(No:)<br>____________(No:)
4.18	Three major causes of neonatal surgeries conducted? (Give reasons and numbers due to cause in last 1 year)	____________(No:)<br>____________(No:)<br>____________(No:)	____________(No:)<br>____________(No:)<br>____________(No:)

(iii) Child Health Statistics

Collect the information from registers (for last one month calendar).

S. No	Age Group	OPD Visits (A)		Emergency Visits (B)		Admission (D)		Deaths (E)			Referrals (F)	Age Specific Fatality Rate G)
		M	F	M	F	M	F	M	F	Reasons		
**4.19**	0-28 days											
**4.20**	1-12 months											
**4.21**	1-5 Years											
**4.22**	5-12 years											
**4.23**	Total											

5. Health Information System and Medical Records

(i) Health Information System

S.No.	Items	Presence/ Absence	Comment
**5.1**	Existence and use of a computer-based **(Record Maintainance)** information system on patient flow (admissions, outpatients, etc.)	☐ Yes ☐ No	
**5.2**	Existence and use of a computer-based information system on important medical indicators	☐ Yes ☐ No	
**5.3**	Existence and use of paper-based information system on patient flow (admissions, outpatients, etc.), if computer-based unavailable	☐ Yes ☐ No	
**5.4**	Existence and use of paper-based information system on important medical indicators, if computer-based unavailable	☐ Yes ☐ No	

(ii) Medical Records

Q. No.	Items in Labour room	Response	Comment
**5.5**	Are case records clear and legible?	☐ Yes ☐ No	
**5.6**	Are records dated?	☐ Yes ☐ No	
**5.7**	Are all admissions and discharge diagnoses clearly written in the notes?	☐ Yes ☐ No	
**5.8**	Are all drugs and treatments clearly identifiable in nurses/hospital records?	☐ Yes ☐ No	
**5.9**	Is information from previous admissions available to staff providing care to mothers, neonates and children?	☐ Yes ☐ No	
**5.10**	Is information from antenatal records available to staff providing care during labour?	☐ Yes ☐ No	
**5.11**	Are previous admission/ case records available to staff providing care during postpartum period?	☐ Yes ☐ No	

6. Hospital Administration

S. No.	Standards and Criterion	Response	Comments
**(i) Availability of adequate and updated treatment guidelines**			
**6.1.**	Guidelines for common conditions are available, wall charts, or job aids.	☐ Yes ☐ No	
**6.2.**	Recommended antibiotics for common infections and essential drugs list are available.	☐ Yes ☐ No	
**6.3.**	New-born resuscitation is described in wall charts	☐ Yes ☐ No	
**6.4.**	Management of Obstetric complications is described in wall charts	☐ Yes ☐ No	
**(ii) Availability of Essentials**			
**6.5.**	Essential drugs are always available and free	☐ Yes ☐ No	
**6.6.**	Essential equipment is available and functioning	☐ Yes ☐ No	
**6.7.**	Essential lab tests are available and delivered timely	☐ Yes ☐ No	
**6.8.**	Transport for referral is available	☐ Yes ☐ No	

**Summary Score –General Information**					
a. Total responses with YES/NO options ☐☐		b. Total Yes ☐☐		c. Score ☐☐.☐☐	
d. Scale (Circle)	**5** (95-100%)	**4** (75-94%)	**3** (51-75%)	**2** (26-50%)	**1** **(< 25%)**

Section B: Labour room

7. General infrastructure- Labour Room

S.no	Question	Response	Comments
**7.1.**	Is the facility has a labour cum delivery room available?	☐ Yes ☐ No	
**7.2.**	Is it open 24 hours	☐ Yes ☐ No	
**7.3.**	If not then for how many hours a day does it open	______ hrs	
**7.4.**	On which floor is the labour room situated	☐	
**7.5.**	Is there availability of stretcher, ramps for the easy accessibility to labour room	☐ Yes ☐ No	
**7.6.**	Is there availability of lifts if the labour room is situated on the higher floor	☐ Yes ☐ No	
**7.7.**	Does the facility have waiting room for patients?	☐ Yes ☐ No	
**7.8.**	**Is there a newborn care corner within Labour room?**	☐ Yes ☐ No	
**7.9.**	**Is a fire extinguisher installed anywhere in the labor room complex?**	☐ Yes ☐ No	
**7.10.**	**Is there a clear signage at entrance of the facility (near registration counter) to direct patients to LR?**	☐ Yes ☐ No	
**7.11.**	**Are names of Doctors, Nurses and ANMs on duty in LR displayed outside the LR?**	☐ Yes ☐ No	
**7.12.**	**Is the electricity connection same as with the**	☐ Yes ☐ No	

	**other part of the hospital???**		
**(i)**	**Electricity**		
**7.13.**	Does the facilities have 24 x 7 uninterrupted electricity supply?	☐ Yes ☐ No	
**7.14.**	Is the power cut predictable?	☐ Yes ☐ No	
**7.15.**	What is the average hour for power cut in a day?	________hours	
**7.16.**	Does the facilities have backup source for the uninterrupted supply of electrical power?	☐ Yes ☐ No	
**7.17.**	Did you have uninterupted power supply (atleast for 12 hours) during the last one month?	☐ Yes ☐ No	
**7.18.**	If NO , how many days?	☐☐ Days	
**(ii)**	**Water supply**		
**7.19.**	Does this facility have 24 x 7 water supply for functions ?	☐ Yes ☐ No	
**7.20.**	What is the main source of water of this facility?		
**7.21.**	Does this facility have uninturrupted water supply for last one month ?	☐ Yes ☐ No	
**7.22.**	If No, how many days?	☐☐	

(iii) Service delivery

**S.no**	**Question**	**Response**	**Comments**
**7.23.**	Who usually conducts the normal deliveries?	a) Specialist (Obstetrician) ☐<br>b) Doctor ☐<br>c) Staff Nurse/ ANM ☐<br>d) Other Staff ☐	
**7.24.**	Who usually conducts the high risk deliveries?	a) Specialist (Obstetrician) ☐<br>b) Doctor ☐<br>c) Staff Nurse/ ANM ☐<br>d) Other Staff ☐<br>e) NA (tick N/A if facility does not conduct caesarean section) ☐	
**7.25.**	Who usually conducts the Caesarean Section?	a) Specialist (Obstetrician) ☐<br>b) Doctor ☐<br>c) Staff Nurse/ ANM ☐<br>d) Other Staff ☐<br>e) NA (tick N/A if facility does not conduct caesarean section) ☐	
**7.26.**	Who usually conducts the operative vaginal deliveries like forceps and vacuum?	a) Specialist (Obstetrician) ☐<br>b) Doctor ☐<br>c) Staff Nurse/ ANM ☐<br>d) Other Staff ☐<br>e) NA (tick N/A if facility does not conduct caesarean section) ☐	
**7.27.**	MO provides the first contact care to the pregnant females?	☐ Yes ☐ No	
**7.28.**	Staff Nurse provides the first contact care to the pregnant females?	☐ Yes ☐ No	
**7.29.**	Has the first contact care staff received training in SBA/BEmOC?	☐ Yes ☐ No	

8. **Equipments- Labour Room**

(i) **Maternal care**

Q. No.	Equipment/ Supplies	Availability	Number	Functionality (Numbers)	Comments
**8.1.**	Normal Delivery Kit	☐ Yes ☐ No			
**8.2.**	Episiotomy kit	☐ Yes ☐ No			
**8.3.**	Forceps Delivery Kit	☐ Yes ☐ No			
**8.4.**	Vacuum extractor Malastrom	☐ Yes ☐ No			
**8.5.**	BP Apparatus & Stethoscope	☐ Yes ☐ No			
**8.6.**	Carditocograph	☐ Yes ☐ No			
**8.7.**	Fetal Doppler	☐ Yes ☐ No			
**8.8.**	Emergency Tray	☐ Yes ☐ No			
**8.9.**	Oxygen source, delivery system	☐ Yes ☐ No			
**8.10.**	Autoclaving /sterilization unit	☐ Yes ☐ No			
**8.11.**	Resuscitation Kit for adults	☐ Yes ☐ No			
**8.12.**	laryngoscope	☐ Yes ☐ No			
**8.13.**	Diathermy machine	☐ Yes ☐ No			
**8.14.**	Shadow less lamps	☐ Yes ☐ No			
**8.15.**	Standard Surgical Set	☐ Yes ☐ No			
**8.16.**	IUD Insertion Kit	☐ Yes ☐ No			
**8.17.**	Suction Apparatus	☐ Yes ☐ No			
**8.18.**	Table Operation, Hydraulic	☐ Yes ☐ No			
**8.19.**	Trolley for patients	☐ Yes ☐ No			
**8.20.**	Color Doppler Ultrasound	☐ Yes ☐ No			
**8.21.**	ECG machine	☐ Yes ☐ No			
**8.22.**	Cardiac monitors	☐ Yes ☐ No			
**8.23.**	Partograph charts	☐ Yes ☐ No			

**Newborn Care**					
**8.24.**	Resuscitation Kit	☐ Yes ☐ No			
**8.25.**	Radiant warmers	☐ Yes ☐ No			
**8.26.**	Pulse Oximeter	☐ Yes ☐ No			
**8.27.**	Weighing scales for children	☐ Yes ☐ No			
**8.28.**	Infantometer	☐ Yes ☐ No			
**8.29.**	Light examination, mobile	☐ Yes ☐ No			
**8.30.**	Thermometers	☐ Yes ☐ No			
**8.31.**	Pediatric Laryngoscope set & cells	☐ Yes ☐ No			
**8.32.**	Flow meters and humidifiers for oxygen	☐ Yes ☐ No			
**8.33.**	Self-inflating bags (0250 ml, 500 ml	☐ Yes ☐ No			
**8.34.**	Glucometer	☐ Yes ☐ No			
**8.35.**	Lamp, ultra- violet	☐ Yes ☐ No			
**8.36.**	Nebulizers	☐ Yes ☐ No			
**8.37.**	Fetal Doppler	☐ Yes ☐ No			
**8.38.**	Stethoscope	☐ Yes ☐ No			
**8.39.**	Suction Equipment (Catheter 6,8, 10 FG)	☐ Yes ☐ No			
**8.40.**	Oxygen Catheter 8 F, Oxygen cylinder	☐ Yes ☐ No			
**8.41.**	Nasal Prongs	☐ Yes ☐ No			
**8.42.**	Nasal Catheters	☐ Yes ☐ No			
**8.43.**	Infant size mask	☐ Yes ☐ No			
**8.44.**	NG tubes (8,10, 12FG)	☐ Yes ☐ No			
**8.45.**	IV sets with chambers	☐ Yes ☐ No			
**8.46.**	Mucus extractor 20 ml	☐ Yes ☐ No			
**8.47.**	IV cannulas(24G, 26G)	☐ Yes ☐ No			
**8.48.**	Glucostix/ multistix	☐ Yes ☐ No			

8.49.	Feeding tube, CH07, L40 cm	☐ Yes ☐ No			
8.50.	Sterile gloves	☐ Yes ☐ No			
8.51.	Disinfectant, 20% Chlorhexidine	☐ Yes ☐ No			

9. DRUGS & CONSUMABLES AND VACCINES

(Methodology: observation, Record review, Provider interaction in the Labour room & Medical store)

Availability of Labour Room Trays *(Observation)*

S.NO	Labour Room Trays	Availability	Total Number of Trays	Functionality (Numbers)	Comments
9.1	**Delivery Tray**	☐ Yes ☐ No			
9.2	**Episiotomy Tray**	☐ Yes ☐ No			
9.3	**Baby Tray**	☐ Yes ☐ No			
9.4	**Medicine Tray**	☐ Yes ☐ No			
9.5	**Emergency Drug Tray**	☐ Yes ☐ No			
9.6	**MVA/ EVA tray**	☐ Yes ☐ No			
9.7	**PPIUCD Tray**	☐ Yes ☐ No			

S.NO	Labour Room Trays				
9.1	Delivery tray	Available	Designated tray	Functional	Comments
9.1.1	Scissors	☐ Yes ☐ No	☐ Yes ☐ No	☐ Yes ☐ No	
9.1.2	Artery forceps	☐ Yes ☐ No	☐ Yes ☐ No	☐ Yes ☐ No	
9.1.3	Sponge holding forceps	☐ Yes ☐ No	☐ Yes ☐ No	☐ Yes ☐ No	
9.1.4	Speculum	☐ Yes ☐ No	☐ Yes ☐ No	☐ Yes ☐ No	
9.1.5	Urinary catheter	☐ Yes ☐ No	☐ Yes ☐ No	☐ Yes ☐ No	
9.1.6	BP blade / surgical blade for cutting cord	☐ Yes ☐ No	☐ Yes ☐ No	☐ Yes ☐ No	
9.1.7	Bowl for antiseptic solution	☐ Yes ☐ No	☐ Yes ☐ No	☐ Yes ☐ No	
9.1.8	kidney tray	☐ Yes ☐ No	☐ Yes ☐ No	☐ Yes ☐ No	
9.1.9	Gauze pieces; cotton swabs	☐ Yes ☐ No	☐ Yes ☐ No	☐ Yes ☐ No	
9.1.10	Sanitary pads	☐ Yes ☐ No	☐ Yes ☐ No	☐ Yes ☐ No	

9.1.11	Gloves	☐ Yes ☐ No	☐ Yes ☐ No	☐ Yes ☐ No	
**9.2**	**Episiotomy Tray**	**Available**	**Designated tray**	**Functional**	**Comment**
**9.2.1**	Inj. Xylocaine 2%	☐ Yes ☐ No	☐ Yes ☐ No	☐ Yes ☐ No	
**9.2.2**	10 ml disposable syringe with needle,	☐ Yes ☐ No	☐ Yes ☐ No	☐ Yes ☐ No	
**9.2.3**	Episiotomy scissor	☐ Yes ☐ No	☐ Yes ☐ No	☐ Yes ☐ No	
**9.2.4**	Kidney tray	☐ Yes ☐ No	☐ Yes ☐ No	☐ Yes ☐ No	
**9.2.5**	Artery forceps	☐ Yes ☐ No	☐ Yes ☐ No	☐ Yes ☐ No	
**9.2.6**	Allis forceps	☐ Yes ☐ No	☐ Yes ☐ No	☐ Yes ☐ No	
**9.2.7**	Sponge holding forceps	☐ Yes ☐ No	☐ Yes ☐ No	☐ Yes ☐ No	
**9.2.8**	Toothed forceps	☐ Yes ☐ No	☐ Yes ☐ No	☐ Yes ☐ No	
**9.2.9**	Needle holder	☐ Yes ☐ No	☐ Yes ☐ No	☐ Yes ☐ No	
**9.2.10**	Needle (round body and cutting)	☐ Yes ☐ No	☐ Yes ☐ No	☐ Yes ☐ No	
**9.2.11**	Chromic catgut no. 0	☐ Yes ☐ No	☐ Yes ☐ No	☐ Yes ☐ No	
**9.2.12**	Gauze pieces	☐ Yes ☐ No	☐ Yes ☐ No	☐ Yes ☐ No	
**9.2.13**	Cotton swabs	☐ Yes ☐ No	☐ Yes ☐ No	☐ Yes ☐ No	
**9.2.14**	Antiseptic lotion	☐ Yes ☐ No	☐ Yes ☐ No	☐ Yes ☐ No	
**9.2.15**	Thumb forceps	☐ Yes ☐ No	☐ Yes ☐ No	☐ Yes ☐ No	
**9.2.16**	Gloves	☐ Yes ☐ No	☐ Yes ☐ No	☐ Yes ☐ No	
**9.3**	**Baby Tray**	**Available**	**Designated tray**	**Functional**	**Comment**
**9.3.1**	Two pre-warmed towels/sheets for wrapping the baby,	☐ Yes ☐ No	☐ Yes ☐ No	☐ Yes ☐ No	
**9.3.2**	Cotton swabs,	☐ Yes ☐ No	☐ Yes ☐ No	☐ Yes ☐ No	
**9.3.3**	Mucus extractor,	☐ Yes ☐ No	☐ Yes ☐ No	☐ Yes ☐ No	
**9.3.4**	Bag & mask,	☐ Yes ☐ No	☐ Yes ☐ No	☐ Yes ☐ No	

**9.3.5**	Sterilized thread for cord/cord clamp,	☐ Yes ☐ No	☐ Yes ☐ No	☐ Yes ☐ No	
**9.3.6**	Nasogastric tube and gloves	☐ Yes ☐ No	☐ Yes ☐ No	☐ Yes ☐ No	
**9.3.7**	Inj. Vitamin K,	☐ Yes ☐ No	☐ Yes ☐ No	☐ Yes ☐ No	
**9.3.8**	Needle and syringe	☐ Yes ☐ No	☐ Yes ☐ No	☐ Yes ☐ No	
**9.4**	**Medicine tray**	**Available**	**Designated tray**	**Functional**	**Comment**
**9.4.1**	Inj. Oxytocin (to be kept in fridge)	☐ Yes ☐ No	☐ Yes ☐ No	☐ Yes ☐ No	
**9.4.2**	Inj. Oxytocin 10 IU	☐ Yes ☐ No	☐ Yes ☐ No	☐ Yes ☐ No	
**9.4.3**	Inj. Gentamycin	☐ Yes ☐ No	☐ Yes ☐ No	☐ Yes ☐ No	
**9.4.4**	Inj. Betamethason	☐ Yes ☐ No	☐ Yes ☐ No	☐ Yes ☐ No	
**9.4.5**	Inj. Hydrazaline	☐ Yes ☐ No	☐ Yes ☐ No	☐ Yes ☐ No	
**9.4.6**	Inj Vit K	☐ Yes ☐ No	☐ Yes ☐ No	☐ Yes ☐ No	
**9.4.7**	Tab Nefidepin	☐ Yes ☐ No	☐ Yes ☐ No	☐ Yes ☐ No	
**9.4.8**	Tab Methyldopa	☐ Yes ☐ No	☐ Yes ☐ No	☐ Yes ☐ No	
**9.4.9**	Tab Metronidazole 400 mg	☐ Yes ☐ No	☐ Yes ☐ No	☐ Yes ☐ No	
**9.4.10**	Tab Paracetamol	☐ Yes ☐ No	☐ Yes ☐ No	☐ Yes ☐ No	
**9.4.11**	Tab Ibuprofen	☐ Yes ☐ No	☐ Yes ☐ No	☐ Yes ☐ No	
**9.4.12**	Tab B complex	☐ Yes ☐ No	☐ Yes ☐ No	☐ Yes ☐ No	
**9.4.13**	Tab. Misoprostol 200 mg	☐ Yes ☐ No	☐ Yes ☐ No	☐ Yes ☐ No	
**9.4.14**	Cap Ampicillin 500 mg	☐ Yes ☐ No	☐ Yes ☐ No	☐ Yes ☐ No	
**9.4.15**	IV fluids	☐ Yes ☐ No	☐ Yes ☐ No	☐ Yes ☐ No	
**9.4.16**	Ringer lactate	☐ Yes ☐ No	☐ Yes ☐ No	☐ Yes ☐ No	
**9.4.17**	Normal Saline	☐ Yes ☐ No	☐ Yes ☐ No	☐ Yes ☐ No	
**9.4.18**	Magnifying glass.	☐ Yes ☐ No	☐ Yes ☐ No	☐ Yes ☐ No	
**9.5**	**Emergency Drug Tray**	**Available**	**Designated tray**	**Functional**	**Comment**
**9.5.1**	Inj. Oxytocin (to be kept in fridge)	☐ Yes ☐ No	☐ Yes ☐ No	☐ Yes ☐ No	
**9.5.2**	Inj. Magsulf 50%	☐ Yes ☐ No	☐ Yes ☐ No	☐ Yes ☐ No	
**9.5.3**	Inj. Calcium gluconate-10%	☐ Yes ☐ No	☐ Yes ☐ No	☐ Yes ☐ No	
**9.5.4**	Inj. Dexamethasone	☐ Yes ☐ No	☐ Yes ☐ No	☐ Yes ☐ No	

**9.5.5**	Inj. Ampicillin	☐ Yes ☐ No	☐ Yes ☐ No	☐ Yes ☐ No	
**9.5.6**	Inj. Gentamicin	☐ Yes ☐ No	☐ Yes ☐ No	☐ Yes ☐ No	
**9.5.7**	Inj. Metronidazole	☐ Yes ☐ No	☐ Yes ☐ No	☐ Yes ☐ No	
**9.5.8**	Inj. Lignocaine-2%	☐ Yes ☐ No	☐ Yes ☐ No	☐ Yes ☐ No	
**9.5.9**	Inj. Adrenaline	☐ Yes ☐ No	☐ Yes ☐ No	☐ Yes ☐ No	
**9.5.10**	Inj. Hydrocortisone Succinate	☐ Yes ☐ No	☐ Yes ☐ No	☐ Yes ☐ No	
**9.5.11**	Inj. Diazepam	☐ Yes ☐ No	☐ Yes ☐ No	☐ Yes ☐ No	
**9.5.12**	Inj. Pheneramine maleate	☐ Yes ☐ No	☐ Yes ☐ No	☐ Yes ☐ No	
**9.5.13**	Inj. Carboprost	☐ Yes ☐ No	☐ Yes ☐ No	☐ Yes ☐ No	
**9.5.14**	Inj. Fortwin	☐ Yes ☐ No	☐ Yes ☐ No	☐ Yes ☐ No	
**9.5.15**	Inj. Phenergan	☐ Yes ☐ No	☐ Yes ☐ No	☐ Yes ☐ No	
**9.5.16**	Ringer lactate	☐ Yes ☐ No	☐ Yes ☐ No	☐ Yes ☐ No	
**9.5.17**	Normal saline	☐ Yes ☐ No	☐ Yes ☐ No	☐ Yes ☐ No	
**9.5.18**	Inj. Betamexthazon	☐ Yes ☐ No	☐ Yes ☐ No	☐ Yes ☐ No	
**9.5.19**	Inj. Hydrazaline	☐ Yes ☐ No	☐ Yes ☐ No	☐ Yes ☐ No	
**9.5.20**	Tab Nefidepin	☐ Yes ☐ No	☐ Yes ☐ No	☐ Yes ☐ No	
**9.5.21**	Tab Methyldopa	☐ Yes ☐ No	☐ Yes ☐ No	☐ Yes ☐ No	
**9.5.22**	IV sets with 16-gauge needle at least two,	☐ Yes ☐ No	☐ Yes ☐ No	☐ Yes ☐ No	
**9.5.23**	Controlled suction catheter,	☐ Yes ☐ No	☐ Yes ☐ No	☐ Yes ☐ No	
**9.5.24**	Mouth gag,	☐ Yes ☐ No	☐ Yes ☐ No	☐ Yes ☐ No	
**9.5.25**	IV Canula,	☐ Yes ☐ No	☐ Yes ☐ No	☐ Yes ☐ No	
**9.5.26**	Vials for drug collection Ceftriaxone (3rd generation cephalosporins) - For L3 facility	☐ Yes ☐ No	☐ Yes ☐ No	☐ Yes ☐ No	
**9.6**	**MVA/ EVA tray**	**Available**	**Designated tray**	**Functional**	**Comment**
**9.6.1**	Gloves	☐ Yes ☐ No	☐ Yes ☐ No	☐ Yes ☐ No	
**9.6.2**	Speculum	☐ Yes ☐ No	☐ Yes ☐ No	☐ Yes ☐ No	
**9.6.3**	Anterior vaginal wall retractor	☐ Yes ☐ No	☐ Yes ☐ No	☐ Yes ☐ No	

**9.6.4**	Posterior vaginal wall retractor	☐ Yes ☐ No	☐ Yes ☐ No	☐ Yes ☐ No	
**9.6.5**	Sponge holding forceps	☐ Yes ☐ No	☐ Yes ☐ No	☐ Yes ☐ No	
**9.6.6**	MVA syringe and cannulas	☐ Yes ☐ No	☐ Yes ☐ No	☐ Yes ☐ No	
**9.6.7**	MTP cannulas	☐ Yes ☐ No	☐ Yes ☐ No	☐ Yes ☐ No	
**9.6.8**	Small bowl of antiseptic lotion	☐ Yes ☐ No	☐ Yes ☐ No	☐ Yes ☐ No	
**9.6.9**	Sanitary pads	☐ Yes ☐ No	☐ Yes ☐ No	☐ Yes ☐ No	
**9.6.10**	Pads /cotton swabs	☐ Yes ☐ No	☐ Yes ☐ No	☐ Yes ☐ No	
**9.6.11**	Disposable syringe and needle	☐ Yes ☐ No	☐ Yes ☐ No	☐ Yes ☐ No	
**9.6.12**	Misoprostol tablet	☐ Yes ☐ No	☐ Yes ☐ No	☐ Yes ☐ No	
**9.6.13**	Sterilized gauze/pads	☐ Yes ☐ No	☐ Yes ☐ No	☐ Yes ☐ No	
**9.6.14**	Urinary catheter	☐ Yes ☐ No	☐ Yes ☐ No	☐ Yes ☐ No	
**9.7**	**PPIUCD tray**	**Available**	**Designated tray**	**Functional**	**Comments**
**9.7.1**	PPIUCD insertion forceps	☐ Yes ☐ No	☐ Yes ☐ No	☐ Yes ☐ No	
**9.7.2**	Sims speculum	☐ Yes ☐ No	☐ Yes ☐ No	☐ Yes ☐ No	
**9.7.3**	Sponge holding forceps	☐ Yes ☐ No	☐ Yes ☐ No	☐ Yes ☐ No	
**9.7.4**	Cu IUCD 380A in sterile pack	☐ Yes ☐ No	☐ Yes ☐ No	☐ Yes ☐ No	
**9.7.5**	Cu IUCD 375 in sterile pack	☐ Yes ☐ No	☐ Yes ☐ No	☐ Yes ☐ No	

10. Staffing (Labour Room)

(i) Availability of staffs in labour room

SL No	Type of staff	Sanctioned number	In-position number	Number during any shift on weekdays			On holiday	Any deficiency in any area (OT/IL.R/Ward /OPD (Yes/No)
				M	E	N		
10.1	Doctor (Gynaecologist)							
10.2	Medical officers							
10.3	Staff nurse							
10.4	ANM							
10.5	Multi-skilled Group D worker							
10.6	Sanitary worker							
10.7	Watchman							
10.8								
10.9								
10.10								

S.No	(ii). Some Specific details of Staffs (For Labor Room)											
10.11						Training						
	Staff	a. Posted since (MM/YY)	b.Full time avaiable in facility	c. Residential	d.If no, Distance from residence	e. SBA	f.BEmOC	g.NSSK	h.MTP	i.RTI/STI	j.IUCD	k.Any other training
10.11.1	Designation<br>Name	☐☐☐☐	YES ☐<br>NO ☐	YES ☐<br>NO ☐	__.__<br>KMs	YES ☐<br>NO ☐	YES ☐<br>NO ☐	YES ☐<br>NO ☐	YES ☐<br>NO ☐	YES ☐<br>NO ☐	YES ☐<br>NO ☐	
10.11.2	Designation<br>Name	☐☐☐☐	YES ☐<br>NO ☐	YES ☐<br>NO ☐	__.__<br>KMs	YES ☐<br>NO ☐	YES ☐<br>NO ☐	YES ☐<br>NO ☐	YES ☐<br>NO ☐	YES ☐<br>NO ☐	YES ☐<br>NO ☐	
10.11.3	Designation<br>Name	☐☐☐☐	YES ☐<br>NO ☐	YES ☐<br>NO ☐	__.__<br>KMs	YES ☐<br>NO ☐	YES ☐<br>NO ☐	YES ☐<br>NO ☐	YES ☐<br>NO ☐	YES ☐<br>NO ☐	YES ☐<br>NO ☐	
10.11.4	Designation<br>Name	☐☐☐☐	YES ☐<br>NO ☐	YES ☐<br>NO ☐	__.__<br>KMs	YES ☐<br>NO ☐	YES ☐<br>NO ☐	YES ☐<br>NO ☐	YES ☐<br>NO ☐	YES ☐<br>NO ☐	YES ☐<br>NO ☐	
10.11.5	Designation<br>Name	☐☐☐☐	YES ☐<br>NO ☐	YES ☐<br>NO ☐	__.__<br>KMs	YES ☐<br>NO ☐	YES ☐<br>NO ☐	YES ☐<br>NO ☐	YES ☐<br>NO ☐	YES ☐<br>NO ☐	YES ☐<br>NO ☐	
10.11.6	Designation<br>Name	☐☐☐☐	YES ☐<br>NO ☐	YES ☐<br>NO ☐	__.__<br>KMs	YES ☐<br>NO ☐	YES ☐<br>NO ☐	YES ☐<br>NO ☐	YES ☐<br>NO ☐	YES ☐<br>NO ☐	YES ☐<br>NO ☐	
10.11.7	Designation<br>Name	☐☐☐☐	YES ☐<br>NO ☐	YES ☐<br>NO ☐	__.__<br>KMs	YES ☐<br>NO ☐	YES ☐<br>NO ☐	YES ☐<br>NO ☐	YES ☐<br>NO ☐	YES ☐<br>NO ☐	YES ☐<br>NO ☐	
10.11.8	Designation<br>Name	☐☐☐☐	YES ☐<br>NO ☐	YES ☐<br>NO ☐	__.__<br>KMs	YES ☐<br>NO ☐	YES ☐<br>NO ☐	YES ☐<br>NO ☐	YES ☐<br>NO ☐	YES ☐<br>NO ☐	YES ☐<br>NO ☐	

11. Infection control practices- Labour Room

(i) Hand washing

Q. no.	Items in Facility	Labour room	Comments
11.1	Hand washing stations are well organized and equipped?	☐ Yes ☐ No	
11.2	Is there availability of running water?	☐ Yes ☐ No	
11.3	Are there soap and/or disinfectant available f or hand washing?	☐ Yes ☐ No	
11.4	Written protocols on hygiene for hands are available near scrub area/ hand wash area?	☐ Yes ☐ No	
**(ii)**	**Use of gloves**		
11.5	Sterile gloves are used for conducting delivery?	☐ Yes ☐ No	
11.6	Gloves are disposed after used according to the waste management protocol	☐ Yes ☐ No	
**(iii)**	**Infection control Practices**		
11.7	Is there a routine disinfection of the labour room?	☐ Yes ☐ No	
11.8	Caps, masks and shoe covers are routinely used by staff	☐ Yes ☐ No	
11.9	There is an infection control policy for visitors in the hospital, that may require restriction of access to patients	☐ Yes ☐ No	
11.10	Is waste disposal and segregation done as per guidelines?	☐ Yes ☐ No	
11.11	Availability of elbow operated taps	☐ Yes ☐ No	
11.12	Heavy duty gloves and gum boats for housekeeping staff	☐ Yes ☐ No	
11.13	Personal protective kit for delivering HIV patients	☐ Yes ☐ No	

12. Case management- Labour Room

(i) Care throughout labour

S.no	Questions	Response	Comments
12.1	There is at least one skilled service provider present throughout labor and birth.	☐ Yes ☐ No	
12.2	A supportive, encouraging atmosphere for birth	☐ Yes ☐ No	
12.3	Partogram is used consistently and for decision making	☐ Yes ☐ No	
**(ii)**	**Care during first stage of labour**		
12.4	Temperature and blood pressure are monitored 4 hourly and pulse on an hourly basis	☐ Yes ☐ No	
12.5	PV is done 4 hourly in active phase	☐ Yes ☐ No	
12.6	Progress of labor is assessed correctly and appropriate actions taken	☐ Yes ☐ No	
**(iii)**	**Care during second stage of labour**		
12.7	Episiotomy is NOT routinely performed	☐ Yes ☐ No	
12.8	Local anaesthesia is given if episiotomy is performed	☐ Yes ☐ No	
12.9	10 IU Oxytocin IM are given to the mother immediately after delivery	☐ Yes ☐ No	
**(iv)**	**Care during third stage of labour**		
12.10	controlled cord traction practiced for placenta delivery	☐ Yes ☐ No	
12.11	Delayed Cord Clamping done	☐ Yes ☐ No	
12.12	Placenta and membranes are checked for completeness	☐ Yes ☐ No	
**(v)**	**Care of mother immediately after delivery**		
12.13	Blood Pressure monitored regularly	☐ Yes ☐ No	

**12.14**	Temperature monitored regularly	☐ Yes ☐ No	
**12.15**	Vaginal bleeding monitored regularly	☐ Yes ☐ No	
**12.16**	Pulse monitored regularly	☐ Yes ☐ No	
**12.17**	Contraction of uterus monitored regularly	☐ Yes ☐ No	
**(vi)**	**Post-Partum Haemorrhage**		
**12.18**	Guidelines and a written protocol for preventing and managing PPH is available, known and used by skilled birth attendants	☐ Yes ☐ No	
**12.19**	Blood can be obtained without delay 24 hours a day	☐ Yes ☐ No	
**(vii)**	**Management of pre-term labour**		
**12.20**	Do you manage pregnant women in preterm labor here?	☐ Yes ☐ No	
**12.21**	Do you give antenatal steroids to such cases?	☐ Yes ☐ No	
**12.22**	Guidelines and written protocol with indications of antenatal steroid are available, known and used	☐ Yes ☐ No	
**(viii)**	**Management of Caesarean Section**		
**12.23**	Anesthesiologist, Obstetrician and operation theatre staff are available	☐ Yes ☐ No	
**12.24**	An operation theatre is 24 hours/7 days fully ready for caesarean sections	☐ Yes ☐ No	
**12.25**	Stored blood is readily available in case blood transfusion needed	☐ Yes ☐ No	
**12.26**	Cases for caesarean section are reviewed before carrying out the procedure by operating obstetrician	☐ Yes ☐ No	
**12.27**	Guidelines and written protocol (or checklist) for caesarean section is available, known and used by staff	☐ Yes ☐ No	
**12.28**	The woman´s vital signs are monitored throughout the procedure and recorded	☐ Yes ☐ No	
**12.29**	After recovery from anesthesia, the vital signs are monitored closely and recorded	☐ Yes ☐ No	
**(ix)**	**Management of unsatisfactory progress of labour**		
**12.30**	Unsatisfactory progress of labor is diagnosed using partograph	☐ Yes ☐ No	
**12.31**	The patient is re-hydrated	☐ Yes ☐ No	
**12.32**	Uterine contractions assessed on partograph	☐ Yes ☐ No	
**12.33**	Assessed for cephalo-pelvic disproportion	☐ Yes ☐ No	
**12.34**	Caesarean section is performed if ACTION line on Partograph is crossed	☐ Yes ☐ No	
**(x)**	**Management of severe anemia in pregnancy**		
**12.35**	Haemoglobin test is routinely done for all pregnant women attending for delivery	☐ Yes ☐ No	
**12.36**	Guidelines and a written protocol for management of anemia is available, known and routinely used by staff.	☐ Yes ☐ No	
**12.37**	All pregnant women with POG 30-36 weeks with severe anemia Hb<7 are to be given Iron Sucrose infusion	☐ Yes ☐ No	
**12.38**	All pregnant women with PoG >36 weeks with severe anemia Hb<7 are to be given blood transfusion	☐ Yes ☐ No	
**(xi)**	**Management of Postpartum Haemorrhage**		
**12.39**	Rapid infusion of RL/ NS 1litre over 15-20 min given	☐ Yes ☐ No	
**12.40**	Oxygen inhalation by mask	☐ Yes ☐ No	
**12.41**	Bladder catheterization, measurement of urine output	☐ Yes ☐ No	
**12.42**	Inj Oxytocin 10 IU IM and start Inj Oxytocin infusion 20 IU in 500 ml RL @ 40-60 drops/ min given	☐ Yes ☐ No	

**12.43**	Vitals monitored every 15 minutes	☐ Yes ☐ No	
**12.44**	Cause of PPH determined & specific management done	☐ Yes ☐ No	
**12.45**	Bimanual Compression of Uterus given	☐ Yes ☐ No	
**(xii)**	**Management of Hypertension in pregnancy**		
**12.46**	Guidelines& written protocol for management of pregnant female with hypertension are used	☐ Yes ☐ No	
**12.47**	BP is regularly monitored and appropriate classification of HT done	☐ Yes ☐ No	
**12.48**	Urine protein checked	☐ Yes ☐ No	
**12.49**	In cases of severe pre-eclampsia/ eclampsia, Magnesium Sulphate is given- 5 g IM in alternate buttocks every four hours for 24 hours after birth/ last convulsion	☐ Yes ☐ No	
**12.50**	Women with pre-eclampsia or gestational hypertension at term, induction of labour is recommended.	☐ Yes ☐ No	
**(xiii)**	**Management protocol during pregnancy and delivery**		
**12.51**	Standard Treatment Protocol/ guidelines are available and is used by the health care staff for the management of obstetric complications and delivery	☐ Yes ☐ No	
**12.52**	Is job description of hospital staff (doctors, nurses, paramedical) defined and communicated	☐ Yes ☐ No	
**12.53**	Is the duty roster of hospital staff (doctors, nurses, paramedical) prepared, updated and communicated	☐ Yes ☐ No	
**12.54**	Labor ward teams use childbirth checklist	☐ Yes ☐ No	
**12.55**	Guidelines and a written protocol for delivery are available, known and used by staff	☐ Yes ☐ No	
**12.56**	Appropriate admission history is taken and recorded in case sheet	☐ Yes ☐ No	

13. Newborn care at birth

S. No.	Questions	Response	Comments
**(i)**	**Neonatal resuscitation**		
**13.1**	Is there a separate area defined for neonatal resuscitation	☐ Yes ☐ No	
**13.2**	Written guidelines for resuscitation and care of the new born are available and documented	☐ Yes ☐ No	
**13.3**	Is there a doctor available 24 hours who is trained in NRP	☐ Yes ☐ No	
**13.4**	Is there a staff nurse available 24 hours who is trained in NRP	☐ Yes ☐ No	
**13.5**	Are the equipment for resuscitation available	☐ Yes ☐ No	
**13.6**	Are all the equipment functional	☐ Yes ☐ No	
**13.7**	Are there job aids for NRP displayed at NBSU	☐ Yes ☐ No	
**(ii)**	**Routine Neonatal care (NBCC)**		
**13.8**	APGAR Score is documented for all	☐ Yes ☐ No	
**13.9**	Kangaroo Mother Care is encouraged for LBW newborns	☐ Yes ☐ No	
**(iii)**	**Early and exclusive breast feeding and skin contact is ensured**		
**13.10**	Skin to skin contact with mother initiated within the first ½hour	☐ Yes ☐ No	

**13.11**	Breast feeding started within 1 hour of birth	☐ Yes ☐ No	
**13.12**	There is no promotion of infant formula on the ward or distributed to mothers	☐ Yes ☐ No	
**13.13**	Mothers stay with their infants in the same room day and night	☐ Yes ☐ No	
**(iv)**	**Babies are kept Warm, vaccination and immunization given**		
**13.14**	Newborns are kept in a warm room, with no draught	☐ Yes ☐ No	
**13.15**	Newborns are cleaned with dry/warm cloth, no bathing or washing given in first 24 hours	☐ Yes ☐ No	
**13.16**	Body temperature is monitored regularly	☐ Yes ☐ No	
**13.17**	Vitamin K prophylaxis at birth given for all newborns	☐ Yes ☐ No	
**13.18**	Immunizations are given (Zero OPV, Hep B and BCG) for all newborns	☐ Yes ☐ No	

14. Display of Protocols in LR (appropriate for the level of facility) - Are the following protocols displayed

**S. No**	**Display of Protocols in LR**	**Response**	**Comments**
**14.1**	Simplified Partograph	☐ Yes ☐ No	
**14.2**	Vaginal bleeding before 20 weeks	☐ Yes ☐ No	
**14.3**	Vaginal bleeding after 20 weeks	☐ Yes ☐ No	
**14.4**	Management of PPH	☐ Yes ☐ No	
**14.5**	Eclampsia	☐ Yes ☐ No	
**14.6**	AMTSL	☐ Yes ☐ No	
**14.7**	New born resuscitation	☐ Yes ☐ No	
**14.8**	Kangaroo Mother Care	☐ Yes ☐ No	
**14.9**	Breastfeeding	☐ Yes ☐ No	
**14.10**	Hand washing	☐ Yes ☐ No	
**14.11**	Preparation of 1 litre bleaching soln.	☐ Yes ☐ No	
**14.12**	Infection prevention	☐ Yes ☐ No	
**14.13**	Processing of used items	☐ Yes ☐ No	
**14.14**	LR Sterilization	☐ Yes ☐ No	
**14.15**	Management of atonic PPH	☐ Yes ☐ No	
**14.16**	Whether JSY entitlements displayed (wall painting/banner etc) in admission/OPD area?	☐ Yes ☐ No	

**14.17**	Whether JSSK entitlements displayed (wall painting/banner etc) in admission/OPD area?	☐ Yes ☐ No	
**14.18**	Whether Referral Transport Details displayed (wall painting/banner etc) in admission/OPD area?	☐ Yes ☐ No	

15. Documentation & Records

Available and Updated for last 3 calendar months *(Methodology: Direct Observation)*

S.no	Records / Documents	Available	Updated	Comments
15.1	**Labour Room Register**	☐Yes ☐ No	☐Yes ☐ No	
15.2	**Partograph in case sheets**	☐Yes ☐ No	☐Yes ☐ No	
15.3	**Referral Register (In)**	☐Yes ☐ No	☐Yes ☐ No	
15.4	**Referral Register (Out)**	☐Yes ☐ No	☐Yes ☐ No	
15.5	**Referral Slip**	☐Yes ☐ No	☐Yes ☐ No	
15.6	**Maternal death record Register**	☐Yes ☐ No	☐Yes ☐ No	
15.7	**Discharge Register**	☐Yes ☐ No	☐Yes ☐ No	
15.8	**Discharge Slip**	☐Yes ☐ No	☐Yes ☐ No	
15.9	**PPIUCD Register**	☐Yes ☐ No	☐Yes ☐ No	
15.10	**LR sterilization Register**	☐Yes ☐ No	☐Yes ☐ No	
15.11	**Handing over-taking over Register**	☐Yes ☐ No	☐Yes ☐ No	
15.12	**PNC Register**	☐Yes ☐ No	☐Yes ☐ No	
15.13	**FP register**	☐Yes ☐ No	☐Yes ☐ No	

**Summary Score –Labor room**					
a. Total responses with YES/NO options ☐☐		b. Total Yes ☐☐		c. Score ☐☐.☐☐	
d.Scale (Circle) ☐☐	**5** (95-100%)	**4** (75-94%)	**3** (51-75%)	**2** (26-50%)	**1** **(< 25%)**

SECTION C: OPERATION THEATRE

16. OT Infrastructure

S.no	Question	Response	Comments
16.1	Does the facility have operation theatre (OT)	☐ Yes ☐ No	
16.2	If not, what are the issues?		
16.3	Are surgeries carried out at present?	☐ Yes ☐ No	
16.4	If operation theatre is present and surgeries are not carried out, what is the reason?	*Non-availability of doctors* ☐<br>*Lack of equipment* ☐<br>*Poor condition of facility* ☐<br>*No power supply* ☐<br>*Other* ☐<br>*N/A* ☐<br>*Specify other*	
16.5	Is Operation theatre used for obstetric / gynecological/ cesarean section purpose	☐ Yes ☐ No	
16.6	Does the facility conduct surgery in children?	☐ Yes ☐ No	
16.7	Does the facility conduct surgery in newborns?	☐ Yes ☐ No	
16.8	Does the facility have minor OT / dressing room	☐ Yes ☐ No	
16.9	Does the facility has protocols for handling equipments	☐ Yes ☐ No	
16.10	Does the facility has procedures manual for Infection prevention and control	☐ Yes ☐ No	
	**(i) Cleanliness**		
16.11	Is there separate functional Public Utilities for Male and Female near or within the OT area?	☐ Yes ☐ No	
16.12	Is the overall cleaniness of the toiltes good?	☐ Yes ☐ No	
16.13	Is the overall cleaninees of OT good?	☐ Yes ☐ No	
16.14	Does the facility has appropriate and functional hand washing facility in the patient care area?	☐ Yes ☐ No	
	**(ii) Waste management**		
16.15	Do you have system for segregation of waste?	☐ Yes ☐ No	
16.16	Are there color coded bins available for waste segregation?	☐ Yes ☐ No	
16.17	Is there display of instructions for management of BMW	☐ Yes ☐ No	

**Summary Score –Operation theatre**					
a. Total responses with YES/NO options ☐☐		b. Total Yes ☐☐		c.Score ☐☐.☐☐	
d. Scale (Circle)	**5** (95-100%)	**4** (75-94%)	**3** (51-75%)	**2** (26-50%)	**1** **(< 25%)**

Section –D: Ward

17. Maternal ward

General infrastructure- Maternal ward

S.no	Questions	Response	Comments
17.1	No of bed in female ward	☐ Yes ☐ No	
17.2	Does the facility have waiting room for attendants?	☐ Yes ☐ No	
17.3	Does the health facility have separate beds for admitting newborns?	☐ Yes ☐ No	
17.4	How many beds are available for newborns?		
**(i)**	**Electricity**		
17.5	Does the facilities have 24 x 7 initerrupted electricity supply?	☐ Yes ☐ No	
17.6	Does the facilities have backup source for the uninterrupted supply of electrical power?	☐ Yes ☐ No	
17.7	Did you have uninterupted power supply during the last one month?	☐ Yes ☐ No	
17.8	If NO , how many days?	_______Days	
**(ii)**	**Water supply**		
17.9	Does this facility have 24 x 7 water supply for functions ?	☐ Yes ☐ No	
17.10	Did you have uninterupted water supply during the last one month?	☐ Yes ☐ No	
**(iii)**	**Cleanliness**		
17.11	Are functional clean toilets available for patients?	☐ Yes ☐ No	
17.12	Are the toilets for health care providers available at wards?	☐ Yes ☐ No	
17.13	Is the overall cleaniness of the ward good?	☐ Yes ☐ No	
17.14	Does the facility has appropriate and functional hand washing facility in the patient care area?	☐ Yes ☐ No	
**(iv)**	**Services**		
17.15	The most seriously ill infants are cared for in a section near the nursing station for direct observation	☐ Yes ☐ No	
17.16	Is there emergency management area near ward	☐ Yes ☐ No	
17.17	Is there a heat source in the ward	☐ Yes ☐ No	
17.18	Minimum 48 hours of stay after delivery is followed for all deliveries is recommended	☐ Yes ☐ No	
17.19	Sick new-borns are kept in a separate unit or room	☐ Yes ☐ No	
17.20	Mothers of sick new-borns are rooming in with their babies.	☐ Yes ☐ No	

18. Staffing- Maternal ward

(i) Availability of staff (maternal ward)

SL No	Type of staff	Sanctioned number	In-position number	Number During any shift on weekdays			On holiday	Any deficiency in any area (OT/IL.R/Ward /OPD
				M	E	N		
18.1	Doctor (Obstetrician)							
18.2	Medical officer							
18.3	Staff nurse							
18.4	ANM							
18.5	Nursing assistant							
18.6	Multi-skilled Group D worker							
18.7	Sanitary worker							
18.8	Sweeper							
18.9	Aya							
18.10	Watchman							
18.11								
18.12								
18.13								
18.14								

S.No	Specific details of staff					Maternity Ward						
							Training					
18.15	Staff	a. Posted since (MM/YY)	b.Full time avaiable in facility	c. Residential	d.If no, Distance from residence	e.SBA/ BEmonc	f. BEmONC	g.NSSK	h.MTP	i.RTI/STI	j.IUCD	k. Any other Training
18.15.1	Designation<br>Name	☐☐☐☐	YES ☐<br>NO ☐	YES ☐<br>NO ☐	__.__ KMs	YES ☐<br>NO ☐	YES ☐<br>NO ☐	YES ☐<br>NO ☐	YES ☐<br>NO ☐	YES ☐<br>NO ☐	YES ☐<br>NO ☐	
18.15.2	Designation<br>Name	☐☐☐☐	YES ☐<br>NO ☐	YES ☐<br>NO ☐	__.__ KMs	YES ☐<br>NO ☐	YES ☐<br>NO ☐	YES ☐<br>NO ☐	YES ☐<br>NO ☐	YES ☐<br>NO ☐	YES ☐<br>NO ☐	
18.15.3	Designation<br>Name	☐☐☐☐	YES ☐<br>NO ☐	YES ☐<br>NO ☐	__.__ KMs	YES ☐<br>NO ☐	YES ☐<br>NO ☐	YES ☐<br>NO ☐	YES ☐<br>NO ☐	YES ☐<br>NO ☐	YES ☐<br>NO ☐	
18.15.4	Designation<br>Name	☐☐☐☐	YES ☐<br>NO ☐	YES ☐<br>NO ☐	__.__ KMs	YES ☐<br>NO ☐	YES ☐<br>NO ☐	YES ☐<br>NO ☐	YES ☐<br>NO ☐	YES ☐<br>NO ☐	YES ☐<br>NO ☐	
18.15.5	Designation<br>Name	☐☐☐☐	YES ☐<br>NO ☐	YES ☐<br>NO ☐	__.__ KMs	YES ☐<br>NO ☐	YES ☐<br>NO ☐	YES ☐<br>NO ☐	YES ☐<br>NO ☐	YES ☐<br>NO ☐	YES ☐<br>NO ☐	
18.15.6	Designation<br>Name	☐☐☐☐	YES ☐<br>NO ☐	YES ☐<br>NO ☐	__.__ KMs	YES ☐<br>NO ☐	YES ☐<br>NO ☐	YES ☐<br>NO ☐	YES ☐<br>NO ☐	YES ☐<br>NO ☐	YES ☐<br>NO ☐	
18.15.7	Designation<br>Name	☐☐☐☐	YES ☐<br>NO ☐	YES ☐<br>NO ☐	__.__ KMs	YES ☐<br>NO ☐	YES ☐<br>NO ☐	YES ☐<br>NO ☐	YES ☐<br>NO ☐	YES ☐<br>NO ☐	YES ☐<br>NO ☐	
18.15.8	Designation<br>Name	☐☐☐☐	YES ☐<br>NO ☐	YES ☐<br>NO ☐	__.__ KMs	YES ☐<br>NO ☐	YES ☐<br>NO ☐	YES ☐<br>NO ☐	YES ☐<br>NO ☐	YES ☐<br>NO ☐	YES ☐<br>NO ☐	

19. Paediatric Ward/ SNCU

Infrastructure- Paediatric Ward

S. No.	Question	Response	Comments
**19.1**	How many beds does the ward have?	☐ Yes ☐ No	
**19.2**	How many patients are currently admitted	☐ Yes ☐ No	
**19.3**	Which age group admitted to the Pediatric ward?		
**19.4**	Are beds safe and well maintained?	☐ Yes ☐ No	
**19.5**	Are mattresses present on the beds	☐ Yes ☐ No	
**19.6**	Do patients receive clean bed linen?	☐ Yes ☐ No	
**19.7**	Are beds clean?	☐ Yes ☐ No	
**19.8**	Is there an emergency management area near the ward	☐ Yes ☐ No	
**19.9**	Is there any temperature regulatory mechanism present in the Pediatric ward	☐ Yes ☐ No	
**19.10**	Are there installed with mesh windows available for use of patients	☐ Yes ☐ No	
**(i)**	**Cleanliness**		
**19.11**	There are sufficient and adequate toilets which are easily accessible near the Pediatric ward	☐ Yes ☐ No	
**19.12**	Mothers have access to running water and to an appropriate space, near the ward, to wash themselves and their child.	☐ Yes ☐ No	
**19.13**	Mothers have access to a washing facility, in order to wash her and her child's clothes.	☐ Yes ☐ No	
**19.14**	Staff has access to hand washing facilities The ward is kept clean and dangerous items are inaccessible for children	☐ Yes ☐ No	

**(ii)**	**Standards**		
**19.15**	Children are kept in a separate ward or separate area of a ward.	☐ Yes ☐ No	
**19.16**	Severely ill children are kept apart from adults in wards such as for infectious diseases or intensive care.	☐ Yes ☐ No	
**19.17**	Children with surgical conditions are atleast kept in a separate room, with staff aware of the special needs for children such as feeding and warmth	☐ Yes ☐ No	
**19.18**	Arrangements are made to meet these needs.	☐ Yes ☐ No	
**19.19**	In cold climates, the ward has an efficient and safe heat source	☐ Yes ☐ No	
**19.20**	Mothers of sick newborns are rooming in with their babies, and have adequate facilities.	☐ Yes ☐ No	

20. Staffing – Paediatric Ward

(i) Availability of staff (Newborn ward)

SL No	Type of staff	Sanctioned number	In-position number	Number During any shift on weekdays			On holiday	Any deficiency in any area (OT/IL.R/Ward/OPD (Yes/No)
				M	E	N		
**20.1**	Medical officer							
**20.2**	Doctor (paediatrician)							
**20.3**	Staff nurse							
**20.4**	ANM							
**20.5**	Nurse Assistant							
**20.6**	Multi-skilled Group D worker							
**20.7**	Sanitary worker							
**21.8**	Sweeper							
**22.9**	Watchman							
**20.10**								

S.No	Specific details of staff (Pediatric Ward)										
20.11	Staff	a. Posted since (MM/YY)	b.Full time avaiable in facility	c. Residential	d.If no, Distance from residence	Training					
						e.IMNCI	f.NSSK	g.HBNC	h.PSBI	i.RBSK	j. Any other Training
20.11.1	Designation<br>Name	☐☐☐☐	YES ☐<br>NO ☐	YES ☐<br>NO ☐	__.__KMs	YES ☐<br>NO ☐	YES ☐<br>NO ☐	YES ☐<br>NO ☐	YES ☐<br>NO ☐	YES ☐<br>NO ☐	
20.11.2	Designation<br>Name	☐☐☐☐	YES ☐<br>NO ☐	YES ☐<br>NO ☐	__.__KMs	YES ☐<br>NO ☐	YES ☐<br>NO ☐	YES ☐<br>NO ☐	YES ☐<br>NO ☐	YES ☐<br>NO ☐	
20.11.3	Designation<br>Name	☐☐☐☐	YES ☐<br>NO ☐	YES ☐<br>NO ☐	__.__KMs	YES ☐<br>NO ☐	YES ☐<br>NO ☐	YES ☐<br>NO ☐	YES ☐<br>NO ☐	YES ☐<br>NO ☐	
20.11.4	Designation<br>Name	☐☐☐☐	YES ☐<br>NO ☐	YES ☐<br>NO ☐	__.__ KMs	YES ☐<br>NO ☐	YES ☐<br>NO ☐	YES ☐<br>NO ☐	YES ☐<br>NO ☐	YES ☐<br>NO ☐	
20.11.5	Designation<br>Name	☐☐☐☐	YES ☐<br>NO ☐	YES ☐<br>NO ☐	__.__ KMs	YES ☐<br>NO ☐	YES ☐<br>NO ☐	YES ☐<br>NO ☐	YES ☐<br>NO ☐	YES ☐<br>NO ☐	
20.11.6	Designation<br>Name	☐☐☐☐	YES ☐<br>NO ☐	YES ☐<br>NO ☐	__.__ KMs	YES ☐<br>NO ☐	YES ☐<br>NO ☐	YES ☐<br>NO ☐	YES ☐<br>NO ☐	YES ☐<br>NO ☐	
20.11.7	Designation<br>Name	☐☐☐☐	YES ☐<br>NO ☐	YES ☐<br>NO ☐	__.__ KMs	YES ☐<br>NO ☐	YES ☐<br>NO ☐	YES ☐<br>NO ☐	YES ☐<br>NO ☐	YES ☐<br>NO ☐	
20.11.8	Designation<br>Name	☐☐☐☐	YES ☐<br>NO ☐	YES ☐<br>NO ☐	__.__ KMs	YES ☐<br>NO ☐	YES ☐<br>NO ☐	YES ☐<br>NO ☐	YES ☐<br>NO ☐	YES ☐<br>NO ☐	

Summary Score – WARD					
a. Total responses with YES/NO options ☐☐		b. Total Yes ☐☐		c. Score ☐☐ . ☐☐	
d.Scale (Circle)	**5** (95-100%)	**4** (75-94%)	**3** (51-75%)	**2** (26-50%)	**1** **(< 25%)**

Section-E: SNCU

21. Standards- SNCU

General Infrastructure

S. No	Standards and Criterion	Response	Comments
21.1	Availability of functional SNCU	☐ Yes ☐ No	
21.2	Pediatrician/ MO trained in SNCU and adequate Staff Nurses	☐ Yes ☐ No	
21.3	SNCU with 12 beds 12-bed unit (plus 4 beds for step-down area)	☐ Yes ☐ No	
21.4	Provision of Kangaroo mother care is available	☐ Yes ☐ No	
21.5	24 hours services are provided	☐ Yes ☐ No	
21.6	Management of low birth weight infants <1800 gm and preterm done	☐ Yes ☐ No	
21.7	Management of sick newborns done	☐ Yes ☐ No	
21.8	Resuscitation of asphyxiated newborns done	☐ Yes ☐ No	
21.9	Prevention of infection, management of newborn sepsis	☐ Yes ☐ No	
21.10	Phototherapy for new born given	☐ Yes ☐ No	
21.11	Screening of New born for birth defects done	☐ Yes ☐ No	

22. Case management of sick new-born

S. No	Standards and Criterion	Response	Comments
(i)	**Appropriate diagnosis and treatment of neonatal sepsis**		
22.1	Neonatal sepsis cases are admitted and treated	☐ Yes ☐ No	
22.2	Blood sugar checked in all sick neonates	☐ Yes ☐ No	
22.3	Sepsis screen (TLC, DLC, CRP) done in suspected sepsis	☐ Yes ☐ No	
22.4	Appropriate antibiotics given as per guidelines	☐ Yes ☐ No	
22.5	The response to treatment is monitored	☐ Yes ☐ No	
(ii)	**Specific feeding needs of sick young infants and those with low birth weight**		
22.6	LBW babies are given breast milk	☐ Yes ☐ No	
22.7	Blood sugar checked periodically	☐ Yes ☐ No	
22.8	Frequent feedings (atleast 8 times per day) are provided to LBW- babies and intake is monitored	☐ Yes ☐ No	
22.9	If neonate is unable to feed expressed breast milk is given by cup and spoon or fed by orogastric tube	☐ Yes ☐ No	
22.10	In LBW-babies, heat loss is minimized by kangaroo mother care	☐ Yes ☐ No	
22.11	If IV-fluids are given, they are recorded and monitored	☐ Yes ☐ No	
(iii)	**Recognition and management of jaundice**		
22.12	Serum bilirubin estimations are done	☐ Yes ☐ No	

**22.13**	Phototherapy and guidelines when to use it are available	☐ Yes ☐ No	
**22.14**	There are guidelines when to recommend a baby who requires exchange transfusion	☐ Yes ☐ No	
**22.15**	Are mothers and/or family members allowed inside the SNCU?	☐ Yes ☐ No	
**22.16**	Are the mothers/family members encouraged to participate in general care of the newborn?	☐ Yes ☐ No	
**22.17**	Are the mothers/family members informed about the status of the newborn atleast daily?	☐ Yes ☐ No	

1. **Staffing- SNCU**

(i) Availability of staff (SNCU)

S. No	Type of staff	Sanctioned number	In-position number	Number During any shift on weekdays			On holiday	Any deficiency in any area (OT/IL.R/Ward /OPD (Yes/No)
				M	E	N		
**23.1**	Medical officer							
**23.2**	Doctor (paediatrician)							
**23.3**	Staff nurse							
**23.4**	ANM							
**23.5**	CC & Vaccine Logistic Assistant							
**23.6**	Multi-skilled Group D worker							
**23.7**	Sanitary worker							
**23.8**	Watchman							
**23.9**								
**23.10**								

S.No	Specific details of staff (SNCU)										
						Training					
23.11	Staff	a. Posted since (MM/YY)	b.Full time avaiable in facility	c. Residential	d.If no, Distance from residence	e.IMNCI	f.NSSK	g.HBNC	h.PSBI	i.RBSK	j. Any other Training
23.11.1	Designation<br>Name	☐☐☐☐	YES ☐<br>NO ☐	YES ☐<br>NO ☐	__.__ KMs	YES ☐<br>NO ☐	YES ☐<br>NO ☐	YES ☐<br>NO ☐	YES ☐<br>NO ☐	YES ☐<br>NO ☐	
23.11.2	Designation<br>Name	☐☐☐☐	YES ☐<br>NO ☐	YES ☐<br>NO ☐	__.__ KMs	YES ☐<br>NO ☐	YES ☐<br>NO ☐	YES ☐<br>NO ☐	YES ☐<br>NO ☐	YES ☐<br>NO ☐	
23.11.3	Designation<br>Name	☐☐☐☐	YES ☐<br>NO ☐	YES ☐<br>NO ☐	__.__ KMs	YES ☐<br>NO ☐	YES ☐<br>NO ☐	YES ☐<br>NO ☐	YES ☐<br>NO ☐	YES ☐<br>NO ☐	
23.11.4	Designation<br>Name	☐☐☐☐	YES ☐<br>NO ☐	YES ☐<br>NO ☐	__.__ KMs	YES ☐<br>NO ☐	YES ☐<br>NO ☐	YES ☐<br>NO ☐	YES ☐<br>NO ☐	YES ☐<br>NO ☐	
23.11.5	Designation<br>Name	☐☐☐☐	YES ☐<br>NO ☐	YES ☐<br>NO ☐	__.__ KMs	YES ☐<br>NO ☐	YES ☐<br>NO ☐	YES ☐<br>NO ☐	YES ☐<br>NO ☐	YES ☐<br>NO ☐	
23.11.6	Designation<br>Name	☐☐☐☐	YES ☐<br>NO ☐	YES ☐<br>NO ☐	__.__ KMs	YES ☐<br>NO ☐	YES ☐<br>NO ☐	YES ☐<br>NO ☐	YES ☐<br>NO ☐	YES ☐<br>NO ☐	
23.11.7	Designation<br>Name	☐☐☐☐	YES ☐<br>NO ☐	YES ☐<br>NO ☐	__.__ KMs	YES ☐<br>NO ☐	YES ☐<br>NO ☐	YES ☐<br>NO ☐	YES ☐<br>NO ☐	YES ☐<br>NO ☐	
23.11.8	Designation<br>Name	☐☐☐☐	YES ☐<br>NO ☐	YES ☐<br>NO ☐	__.__ KMs	YES ☐<br>NO ☐	YES ☐<br>NO ☐	YES ☐<br>NO ☐	YES ☐<br>NO ☐	YES ☐<br>NO ☐	

2. Equipments- SNCU

S. No	Equipment/ Supplies	Availability	Number	Functionality (Number)	Comments
**24.1**	Phototherapy Unit	☐ Yes ☐ No			
**24.2**	Emergency Resuscitation Kit-Baby	☐ Yes ☐ No			
**24.3**	Oxygen Concentrator	☐ Yes ☐ No			
**24.4**	Radiant Warmer	☐ Yes ☐ No			
**24.5**	Room Warmer	☐ Yes ☐ No			
**24.6**	Pulse Oxymeter	☐ Yes ☐ No			
**24.7**	Nebulizer	☐ Yes ☐ No			
**24.8**	Weighing machine infant	☐ Yes ☐ No			
**24.9**	CPAP Machine	☐ Yes ☐ No			
**24.10**	Stethoscope	☐ Yes ☐ No			
**24.11**	Suction Machine	☐ Yes ☐ No			
**24.12**	Hub cutters	☐ Yes ☐ No			
**24.13**	Infantometer	☐ Yes ☐ No			
**24.14**	Light examination, mobile	☐ Yes ☐ No			
**24.15**	Thermometers	☐ Yes ☐ No			
**24.16**	Pediatric Laryngoscope set & cells	☐ Yes ☐ No			
**24.17**	Flow meters and humidifiers for oxygen	☐ Yes ☐ No			
**24.18**	Self-inflating bags (0250 ml, 500 ml	☐ Yes ☐ No			
**24.19**	Glucometer	☐ Yes ☐ No			
**24.20**	Lamp, ultra- violet	☐ Yes ☐ No			
**24.21**	Fetal Doppler	☐ Yes ☐ No			
**24.22**	Oxygen Catheter 8 F, Oxygen cylinder	☐ Yes ☐ No			
**24.23**	Nasal Prongs	☐ Yes ☐ No			
**24.24**	Nasal Catheters	☐ Yes ☐ No			
**24.25**	Infant size mask	☐ Yes ☐ No			
**24.26**	NG tubes (8,10, 12FG)	☐ Yes ☐ No			

**24.27**	IV sets with chambers	☐ Yes ☐ No			
**24.28**	Mucus extractor 20 ml	☐ Yes ☐ No			
**24.29**	IV cannulas(24G, 26G)	☐ Yes ☐ No			
**24.30**	Glucostix/ multistix	☐ Yes ☐ No			
**24.31**	Feeding tube, CH07, L40 cm	☐ Yes ☐ No			
**24.32**	Sterile gloves	☐ Yes ☐ No			
**24.33**	Disinfectant, 20% Chlorhexidine	☐ Yes ☐ No			

**Summary Score –SNCU**					
a. Total responses with YES/NO options ☐☐		b. Total Yes ☐☐	c. Score ☐☐.☐☐		
d.Scale (Circle)	**5** **(**95-100%)	**4** **(**75-94%)	**3** **(**51-75%)	**2** **(**26-50%)	**1** **(< 25%)**

SECTION F: - OUT PATIENT DEPARTMENT

3. Infrastructure – Out Patient Department

S.No	Question	Response	Comment
**(i)**	**Paediatric OPD**		
**25.1**	Is the facility runs a separate pediatric outpatient department?	☐ Yes ☐ No	
**25.2**	At what time does the outpatient department open and close?	Mon- Fri ☐☐ : ☐☐ to ☐☐ : ☐☐<br>Sat ☐☐ : ☐☐ to ☐☐ : ☐☐<br>Sun & Holidays ☐☐ : ☐☐ to ☐☐ : ☐☐	
**25.3**	Does the health facility have a separate emergency area for newborn services?	☐ Yes ☐ No	
**25.4**	Is it open 24 hours	☐ Yes ☐ No	
**25.5**	If not then for how many hours a day does it open	________Hrs	
**25.6**	MO provides the first contact care to the pediatric patient?	☐ Yes ☐ No	
**25.7**	Has the first contact care staff received training in resuscitation/FBNC/NSSK/ F-IMNCI	☐ Yes ☐ No	
**(ii)**	**OBS & GYNAE OPD**		
**25.8**	Is the obstetrical outpatient separate from the Adult outpatient department?	☐ Yes ☐ No	
**25.9**	At what time does the outpatient department open and close?	Mon- Fri ☐☐ : ☐☐ to ☐☐ : ☐☐<br>Sat ☐☐ : ☐☐ to ☐☐ : ☐☐<br>Sun & Holidays ☐☐ : ☐☐ to ☐☐ : ☐☐	
**25.10**	MO provides the first contact care to the patient?	☐ Yes ☐ No	
**25.11**	Has the first contact care staff received training in SBA/BEmOnc/High risk pregnancy care)	☐ Yes ☐ No	
**25.12**	Suggestion/ Complaint Box avaiable in the OPD	☐ Yes ☐ No	
**25.13**	Does this OPD have separate drug dispensing unit.	☐ Yes ☐ No	
**25.14**	Does the facility have registration counter	☐ Yes ☐ No	
**(iii)**	**Cleanliness**		
**25.15**	Separate functional Public Utilities for Male and Female ?	☐ Yes ☐ No	
**25.16**	Is the overall cleaniness of the toilets good?	☐ Yes ☐ No	
**25.17**	Is the overall cleaniness of the OPD room good?	☐ Yes ☐ No	

**25.18**	Is the overall cleaniness of the OPDs good?(Including the waiting area)	☐ Yes ☐ No	
**25.19**	Does the facility has appropriate and functional hand washing facility in the patient care area?	☐ Yes ☐ No	
**25.20**	Does the OPD have running water facility	☐ Yes ☐ No	
**25.21**	Does the water supply in OPD is supported with overhead water tank and pump?	☐ Yes ☐ No	
**(iV)**	**Waste management**		
**25.22**	Do you have system for segregation of waste?	☐ Yes ☐ No	
**25.23**	Are there color coded bins for segregation of waste?	☐ Yes ☐ No	
**25.24**	Is there display of instructions for management of BMW	☐ Yes ☐ No	

4. Staffing at OPD

Availability of staff (OPD)

**S. No**	**Type of staff**	**Sanctioned number**	**In-position number**	**Number During any shift on weekdays**			**On holiday**	**Any deficiency in any area (OT/IL.R/Ward/ OPD (Yes/No)**
				**M**	**E**	**N**		
**26.1**	Doctor (obstetrician)							
**26.2**	Doctor (paediatrician)							
**26.3**	Staff nurse							
**26.4**	ANM							
**26.5**	Nursing Assisstant							
**26.6**	Multi-skilled Group D worker							
**26.7**	Sanitary worker							
**26.8**								
**26.9**								
**26.10**								

S.No	Specific details of staff (OPD)														
26.11	Staff	a. Posted since (MM/YY)	b.Full time avaiable in facility	c. Residential	d.If no, Distance from residence	Training									
						Mother				Newborn					
						e.SBA BEmOC	f.MTP	g.RTI/STI	h.IUCD	i.IMNCI	j. NSSK	k. HBNC	L.PSBI	M. RBSK	N. Any other training
26.11.1	Designation<br>Name	☐☐☐☐	YES ☐<br>NO ☐	YES ☐<br>NO ☐	__.__ KMs	YES ☐<br>NO ☐	YES ☐<br>NO ☐	YES ☐<br>NO ☐	YES ☐<br>NO ☐	YES ☐<br>NO ☐	YES ☐<br>NO ☐	YES ☐<br>NO ☐	YES ☐<br>NO ☐	YES ☐<br>NO ☐	
26.11.2	Designation<br>Name	☐☐☐☐	YES ☐<br>NO ☐	YES ☐<br>NO ☐	__.__ KMs	YES ☐<br>NO ☐	YES ☐<br>NO ☐	YES ☐<br>NO ☐	YES ☐<br>NO ☐	YES ☐<br>NO ☐	YES ☐<br>NO ☐	YES ☐<br>NO ☐	YES ☐<br>NO ☐	YES ☐<br>NO ☐	
26.11.3	Designation<br>Name	☐☐☐☐	YES ☐<br>NO ☐	YES ☐<br>NO ☐	__.__ KMs	YES ☐<br>NO ☐	YES ☐<br>NO ☐	YES ☐<br>NO ☐	YES ☐<br>NO ☐	YES ☐<br>NO ☐	YES ☐<br>NO ☐	YES ☐<br>NO ☐	YES ☐<br>NO ☐	YES ☐<br>NO ☐	
26.11.4	Designation<br>Name	☐☐☐☐	YES ☐<br>NO ☐	YES ☐<br>NO ☐	__.__ KMs	YES ☐<br>NO ☐	YES ☐<br>NO ☐	YES ☐<br>NO ☐	YES ☐<br>NO ☐	YES ☐<br>NO ☐	YES ☐<br>NO ☐	YES ☐<br>NO ☐	YES ☐<br>NO ☐	YES ☐<br>NO ☐	
26.11.5	Designation<br>Name	☐☐☐☐	YES ☐<br>NO ☐	YES ☐<br>NO ☐	__.__ KMs	YES ☐<br>NO ☐	YES ☐<br>NO ☐	YES ☐<br>NO ☐	YES ☐<br>NO ☐	YES ☐<br>NO ☐	YES ☐<br>NO ☐	YES ☐<br>NO ☐	YES ☐<br>NO ☐	YES ☐<br>NO ☐	
26.11.6	Designation<br>Name	☐☐☐☐	YES ☐<br>NO ☐	YES ☐<br>NO ☐	__.__ KMs	YES ☐<br>NO ☐	YES ☐<br>NO ☐	YES ☐<br>NO ☐	YES ☐<br>NO ☐	YES ☐<br>NO ☐	YES ☐<br>NO ☐	YES ☐<br>NO ☐	YES ☐<br>NO ☐	YES ☐<br>NO ☐	
26.11.7	Designation<br>Name	☐☐☐☐	YES ☐<br>NO ☐	YES ☐<br>NO ☐	__.__ KMs	YES ☐<br>NO ☐	YES ☐<br>NO ☐	YES ☐<br>NO ☐	YES ☐<br>NO ☐	YES ☐<br>NO ☐	YES ☐<br>NO ☐	YES ☐<br>NO ☐	YES ☐<br>NO ☐	YES ☐<br>NO ☐	
26.11.8	Designation<br>Name	☐☐☐☐	YES ☐<br>NO ☐	YES ☐<br>NO ☐	__.__ KMs	YES ☐<br>NO ☐	YES ☐<br>NO ☐	YES ☐<br>NO ☐	YES ☐<br>NO ☐	YES ☐<br>NO ☐	YES ☐<br>NO ☐	YES ☐<br>NO ☐	YES ☐<br>NO ☐	YES ☐<br>NO ☐	

Summary Score –Out-patient department					
b. Total responses with YES/NO options	☐☐	b. Total Yes	☐☐	c.Score	☐☐ ☐☐
d.Scale (Circle)	**5** (95-100%)	**4** (75-94%)	**3** (51-75%)	**2** (26-50%)	**1** **(< 25%)**

Section-G: Laboratory services

5. Laboratory Tests

S. No.	Name of Test	Availability	Available 24X7	Time to get results	Comments
27.1	Blood Glucose	☐ Yes ☐ No	☐ Yes ☐ No		
27.2	Glucose Tolerance Test	☐ Yes ☐ No	☐ Yes ☐ No		
27.3	Hemoglobin	☐ Yes ☐ No	☐ Yes ☐ No		
27.4	Peripheral blood smear	☐ Yes ☐ No	☐ Yes ☐ No		
27.5	Serum bilirubin	☐ Yes ☐ No	☐ Yes ☐ No		
27.6	Blood grouping, Cross matching, Rh typing	☐ Yes ☐ No	☐ Yes ☐ No		
27.7	Urine analysis for sugar, albumin	☐ Yes ☐ No	☐ Yes ☐ No		
27.8	VDRL	☐ Yes ☐ No	☐ Yes ☐ No		
27.8	Blood urea, cholesterol	☐ Yes ☐ No	☐ Yes ☐ No		
27.9	Elisa test for HIV, HBsAg, HCV, malaria	☐ Yes ☐ No	☐ Yes ☐ No		

28. Laboratory standards

S. No.	Standards and Criterion	Response	Comments
28.1	Essential lab tests (blood glucose, hemoglobin, hematocrit) are available all the time and their results delivered in a timely fashion to the ward emergency area.	☐ Yes ☐ No	
28.2	Tests are available free of cost to patients	☐ Yes ☐ No	
28.3	Tests for emergency cases are given priority	☐ Yes ☐ No	
28.4	The laboratory is situated such that it has easy access to IPD as well as OPD patients.	☐ Yes ☐ No	
28.5	There is a separate and demarcated areas for sample collection	☐ Yes ☐ No	
28.6	There is a separate area for sample processing	☐ Yes ☐ No	
28.7	There is a separate and demarcated areas for report distribution/ collection.	☐ Yes ☐ No	

Summary Score –Laboratory					
a. Total responses with YES/NO options ☐☐			b. Total Yes ☐☐	c. Score ☐☐ ☐☐	
d. Scale (Circle)	**5** (95-100%)	**4** (75-94%)	**3** (51-75%)	**2** (26-50%)	**1** (< 25%)

Section-H: Pharmacy

29. Drugs

S. No.	Drugs	Availability	Comment	S. No	Drugs	Availability	Comment
**Maternal Care**							
29.1	Cap Amoxycillin	☐ Yes ☐ No		29.20	Inj Thiopentone	☐ Yes ☐ No	
29.2	Tab Metronidazole	☐ Yes ☐ No		29.21	Inj Bupivacaine	☐ Yes ☐ No	
29.3	Tab Misprostol	☐ Yes ☐ No		29.22	Inj Ampicillin	☐ Yes ☐ No	
29.4	Tab Nifedipine	☐ Yes ☐ No		29.23	Inj Gentamycin	☐ Yes ☐ No	
29.5	Tab Ibuprofen	☐ Yes ☐ No		29.24	Inj Dexmethasone	☐ Yes ☐ No	
29.6	Tab Norfloxacin	☐ Yes ☐ No		29.25	Inj Hydrocortisone	☐ Yes ☐ No	
29.7	Tab Fluconazole	☐ Yes ☐ No		29.26	Inj Paracetamol	☐ Yes ☐ No	
29.8	Tab Paracetamol	☐ Yes ☐ No		29.27	Inj Magnesium sulphate	☐ Yes ☐ No	
29.9	Tab Methyl Dopa	☐ Yes ☐ No		29.28	Inj Oxytocin	☐ Yes ☐ No	
29.10	Tab Labetolol	☐ Yes ☐ No		29.29	Inj Insulin	☐ Yes ☐ No	
29.11	Tab. Medroxy Progestrone Acetate	☐ Yes ☐ No		29.30	Inj Aminophylline	☐ Yes ☐ No	
29.12	Tab. Clotrimazole (Vag)	☐ Yes ☐ No		29.31	Inj Hydroxy Projesterone	☐ Yes ☐ No	
29.13	Tab Clotrimazole + Clindamicin (Vag)	☐ Yes ☐ No		29.32	Inj Methyl Ergometrine	☐ Yes ☐ No	
29.14	Inj Xylocaine	☐ Yes ☐ No		29.33	Inj. Dilantin Sodium	☐ Yes ☐ No	
29.15	Inj Betamethasone	☐ Yes ☐ No		29.34	Inj diazepam	☐ Yes ☐ No	
29.16	Inj Hydralazine	☐ Yes ☐ No		29.35	inj Pheniramine maleate	☐ Yes ☐ No	
29.17	Inj Atropine	☐ Yes ☐ No		29.36	Inj Corboprost	☐ Yes ☐ No	
29.18	Tab Doxycycline	☐ Yes ☐ No		29.37	Tab Iron FA	☐ Yes ☐ No	
29.19	Inj Lignocaine	☐ Yes ☐ No					
**Newborn care**							
29.38	Pediatric Maintenance Fluid (Isolyte P)	☐ Yes ☐ No		29.53	Combined mineral and vitamins	☐ Yes ☐ No	
29.39	Syp Amoxycillin-Clavulanic	☐ Yes ☐ No		29.54	Amoxycillin-Clavulanic Suspension	☐ Yes ☐ No	
29.40	Glucose 10% IV	☐ Yes ☐ No		29.55	Inj Vit K (IM)	☐ Yes ☐ No	
29.41	Glucose 20/25% IV	☐ Yes ☐ No		29.56	Inj Adrenaline	☐ Yes ☐ No	
29.42	Normal Saline	☐ Yes ☐ No		29.57	BCG Vaccine	☐ Yes ☐ No	

29.43	Inj Phenobarbital	☐ Yes ☐ No		29.58	Hep B vaccine	☐ Yes ☐ No	
29.44	Ampicillin/Amoxycillin	☐ Yes ☐ No		29.59	OPV Vaccine	☐ Yes ☐ No	
29.45	Inj Benzyl Penicillin	☐ Yes ☐ No		29.60	Inj Calcium gluconate	☐ Yes ☐ No	
29.46	Inj Cloxacillin	☐ Yes ☐ No		29.61	Tab Oral Zinc	☐ Yes ☐ No	
29.47	Inj Gentamycin	☐ Yes ☐ No		29.62	ORS	☐ Yes ☐ No	
29.48	Inj Amikacin	☐ Yes ☐ No		29.63	Inj. Cefotaxime	☐ Yes ☐ No	
29.49	Inj Dopamine/ Dobutamine	☐ Yes ☐ No		29.64	Ringer Lactate IVF	☐ Yes ☐ No	
29.50	Syp. Iron/FA	☐ Yes ☐ No		29.65	Tab Ciprofloxacin	☐ Yes ☐ No	
29.51	Inj Lasix	☐ Yes ☐ No		29.66	Inj Naloxone	☐ Yes ☐ No	
29.52	Salbutamol nebusol solution	☐ Yes ☐ No					

30. Standards- Pharmacy

Essential Drugs			
**S. No**	**Question**	**Response**	**Comments**
**30.1**	Is there drug inventory register?	☐ Yes ☐ No	
**30.2**	Is the drug inventory register up-to-date?	☐ Yes ☐ No	
**30.3**	Is there any mechanism to ensure that expiry drugs are not distributed?	☐ Yes ☐ No	
**30.4**	An essential drug list exists in the hospital	☐ Yes ☐ No	
**30.5**	Are adequate quantity of drugs in essential drug list available	☐ Yes ☐ No	
**30.6**	Oldest drugs are used first	☐ Yes ☐ No	
**30.7**	The facility ensures that drugs which are available at Pharmacy and or wards are prescribed to patients as priority	☐ Yes ☐ No	
**30.8**	Antibiotic policy exists and revised as per sensitivity pattern	☐ Yes ☐ No	

Final facility assessment score

		Score	Rating	Improvement Scale (Circle one)
	**General**			Performance to be sustained(Rating 5)……………………………………………1<br>Needs improvement in some areas (Rating 3 and 4)…………….…………2<br>Improvement needed in many areas (Rating 1 or 2)…………………………3
	**Labour Room**			Performance to be sustained(Rating 5)……………………………………………1<br>Needs improvement in some areas (Rating 3 and 4)…………….…………2<br>Improvement needed in many areas (Rating 1 or 2)…………………………3
	**WARD**			Performance to be sustained(Rating 5)……………………………………………1<br>Needs improvement in some areas (Rating 3 and 4)…………….…………2<br>Improvement needed in many areas (Rating 1 or 2)…………………………3
	**SNCU**			Performance to be sustained(Rating 5)……………………………………………1<br>Needs improvement in some areas (Rating 3 and 4)…………….…………2<br>Improvement needed in many areas (Rating 1 or 2)…………………………3
	**OPD**			Performance to be sustained(Rating 5)……………………………………………1<br>Needs improvement in some areas (Rating 3 and 4)…………….…………2<br>Improvement needed in many areas (Rating 1 or 2)…………………………3
	**LABORATORY**			Performance to be sustained(Rating 5)……………………………………………1<br>Needs improvement in some areas (Rating 3 and 4)…………….…………2<br>Improvement needed in many areas (Rating 1 or 2)…………………………3
	**PHARMACY**			Performance to be sustained(Rating 5)……………………………………………1<br>Needs improvement in some areas (Rating 3 and 4)…………….…………2<br>Improvement needed in many areas (Rating 1 or 2)…………………………3

End of assessment tool

Main Strengths

……

……

Main Weaknesses

……

……

Suggestions for improvement

……

……

UNIQUE ID [ ]

Implementation Research on management of Possible Serious Bacterial Infection in young infants where referral is not feasible

FRU Assessment tool

**SECTION I: Assessment Information**<br>(write the number of correct answer inside the box)	
I. Instrument Number: [ ][ ][ ][ ]	II. Team Member Code: [ ][ ]
III. Parcel ID : [ ][ ][ ][ ]	IV. Unique facility identification: [ ][ ][ ][ ]
V Block Name: ……………………………	VI Village Name: ………………………………
VII Location /Add……………………………	VIII Date of Survey [ ][ ][ ][ ][2][0][1][7]
IX Start Time:	X End Time:

Guidelines for interviewers

- Greet the hospital authority and staffs in respective areas/facilities and introduce yourself.
- Explain the purpose of the assessment.
- Please introduce each question separately and record the response.
- For some section, the suppository documents may be needed, please collect and append, if necessary.
- Marking the response.

SECTION-A: GENERAL INFORMATION

1. Facility Identification

S. no	Question	Response	Comments
1.1	Name of facility:……………………		
1.2	Is the facilty easily accessible?	☐ Yes ☐ No	
1.3	Is the approach road to the facility metalled ?	☐ Yes ☐ No	
1.4	Is the condition of approach road is good?		
1.5	What is the locality where this facility located:<br>(Multiple options can be possible)	Residential area ☐<br>Business/market area ☐<br>Institutional area ☐<br>Far from residential/business area ☐<br>Any other ☐<br>*If other, mention:*	
1.6	Does the facility have a transport facility for referral of mothers and newborns?	☐ Yes ☐ No	

2. General Infrastructure

Q. No.	Question	Response	Comments
2.1	Is this facility open 24 x 7 for the public?	☐ Yes ☐ No	
2.2	Whether the facility has an enquiry/Help desk?	☐ Yes ☐ No	
2.3	Whether the citizen charter is displayed at prominent place in this facility?	☐ Yes ☐ No	
2.4	Does the health facility have a separate emergency department?	☐ Yes ☐ No	
2.5	Is it open 24x7?		
2.6	If not, what hours is it open?	________**Hrs**	
2.7	Does the health facility have separate labour room?	☐ Yes ☐ No	
2.8	Is the labour room open 24 hours	☐ Yes ☐ No	
2.9	If not then for how many hours a day does it open	_______ hrs	
2.10	Does the health facility have an Intensive care Unit (ICU) for managing critically ill obstetric patients	☐ Yes ☐ No	
2.11	Does the health facility have a ward (maternity ward) for admitting obstetrics patients	☐ Yes ☐ No	
2.12	Is the obstetrical outpatient department separate from the adult outpatient department?	☐ Yes ☐ No	
2.13	At what time does the obstetrical outpatient department open and close?	Opens - ☐☐:☐☐ AM/PM<br>Closes - ☐☐:☐☐ AM/PM	
2.14	Does the health facility have an operation theatre?	☐ Yes ☐ No	
2.15	If yes, is the operation theatre available 24 hours?	☐ Yes ☐ No	

**2.16**	If not, what hours is it open?	__________ Hrs	
**2.17**	**If not functional, what is the reason?**		
**2.18**	Does the health facility provide zero expense treatment to the BPL cardholders	☐ Yes ☐ No	
**2.19**	Does this facility have a Functional Blood Bank?	☐ Yes ☐ No	
**2.20**	Does the Blood bank have facility of blood collection and storage?	☐ Yes ☐ No	
**2.21**	Does the Blood Bank have facility for Blood Components separation?	☐ Yes ☐ No	
**2.22**	Does the facility have provisions of Janani Suraksha Yojana (JSY) and monetory incentives are given to the patients?	☐ Yes ☐ No	
**2.23**	Does the facility have provisions of Janani Shishu Suraksha Karyakram (JSSK)- Free drugs, diagnostics, diet, consumables given to pregnant female and newborns	☐ Yes ☐ No	
**2.24**	Number of beneficiaries in last 3 months	☐☐☐☐	
**2.25**	Does the facility have entitlements under JSY & JSSK displayed prominently in the hospital	☐ Yes ☐ No	
**2.26**	Does the facility have Central Sterilization Supply Department (CSSD)?	☐ Yes ☐ No	
**2.27**	Does the facility provide diets to admitted patients during day and night according to the nutritional requirements?	☐ Yes ☐ No	
**2.28**	Does the facility have displayed IEC / BCC materials/ for educating patients & visitors regarding pregnancy and/or postnatal care?	☐ Yes ☐ No	
**2.29**	Does the facility provide and update the "Mother and Child Protection Card" for care of mothers and neonates?	☐ Yes ☐ No	
**2.30**	Does the hospital have a functional RKS with its meeting held at prescribed interval and minutes of meeting are recorded?	☐ Yes ☐ No	
**2.31**	Is there a separate functioning fridge available for drugs or vaccines in labour room and pharmacy?	☐ Yes ☐ No	
**2.32**	Is there a complaints box on the hospital premises or a formal way patients can communicate with the hospital?	☐ Yes ☐ No	
**2.33**	Is there appropriate mechanism in place to contact duty doctor? (On telephone/stays nearby)	☐ Yes ☐ No	
**Does this facility have the following services?**			
**2.35**	Functional Integrated counseling and testing center (ICTC)	☐ Yes ☐ No	
**2.36**	Functional Antiretroviral therapy(ART)	☐ Yes ☐ No	

**2.37**	Functional Directly observed treatment short course(DOTS) Center	☐ Yes ☐ No	
**2.38**	Is the stretcher facility available?	☐ Yes ☐ No	
**2.39**	Is the wheel chair facility available?	☐ Yes ☐ No	
**2.40**	Is ramp access available?	☐ Yes ☐ No	
**2.41**	Whether directions and signage's present?	☐ Yes ☐ No	
**2.42**	Is there adequate separate waiting area for attendants of admitted patients?	☐ Yes ☐ No	
**(i)**	**Electricity**		
**2.43**	Does the facilities have 24 x 7 initerrupted electricity supply?	☐ Yes ☐ No	
**2.44**	Is the power cut predictable?	☐ Yes ☐ No	
**2.45**	What is the average hour for power cut in a day?	_______hrs/day	
**2.46**	Does the facilities have backup source for the uninterrupted supply of electrical power?	☐ Yes ☐ No	
**2.47**	Did you have uninterupted power supply during the last one month?	☐ Yes ☐ No	
**2.48**	If NO , how many days?	D[illegible] ☐☐	
**(ii)**	**Water supply**		
**2.49**	Does this facility have 24 x 7 water supply for functions ?	☐ Yes ☐ No	
**2.50**	Did you have uninterupted water supply during the last one month?	☐ Yes ☐ No	
**2.51**	If No, how many days?	Days ☐☐	
**2.52**	Does the facility have appropriate and functional hand washing facility in the patient care area?	☐ Yes ☐ No	
**2.53**	Does the water supply in the facility is supported with overhead water tank and pump?	☐ Yes ☐ No	
**(iii)**	**Cleanliness**		
**2.54**	Is there Separate functional Public Utilities for Male and Female in waiting area ?	☐ Yes ☐ No	
**2.55**	Is the overall cleaniness of the toilets good in waiting area?	☐ Yes ☐ No	
**2.56**	Is the overall cleaniness of the waiting area good?	☐ Yes ☐ No	

3. Staffing

(i) Availability of staff in the facility

SL No	Type of staff	A. Sanctioned number	B. In-position number	C. Number During any shift on weekday			D. On holiday	E. Any deficiency in (OT/LR/Ward/OPD (Yes/No)
				M	E	N		
**3.1.**	Chief medical officer							
**3.2.**	**Medical officers**							
	Paediatrician							
	Obstetrician							
	General surgeon							
	Medicine specialist							
	Orthopaedic							
	ENT							
	Dentist							
	Other specialists							
**3.3.**	Doctors							
**3.4.**	Staff nurse							
**3.5.**	Physiotherapist							
**3.6.**	Storekeeper							
**3.7.**	Radiographer							
**3.8.**	Dietician							
**3.9.**	Counsellor							
**3.10.**	O.T technician							
**3.11.**	CSSD Asstt.							
**3.12.**	Pharmacist							
**3.13.**	Laboratory Technician							
**3.14.**	CC & Vaccine Logistic Assistant							
**3.15.**	Dental technician							

4. **Hospital health statistics**

(i) **Maternal health statistics**

Q. No.	Questions	Response	
		**Last year**	**Last 3 months**
**4.1**	Number of deliveries (total)		
**4.2**	Number of live births		
**4.3**	Number of still births (total)		
**4.4**	Number of MTPs (total)		
**4.5**	Number of spontaneous abortions		
**4.6**	Number of septic abortions		
**4.7**	Number of Caesarean section deliveries		
**4.8**	Number of Referrals received for maternal cases		
**4.9**	Number of maternal deaths in hospital		
**4.10**	Three major causes of maternal deaths? (Specify reasons and numbers due to the cause in last 1 year)	______ (No:) ______ (No:) ______ (No:)	______ (No:) ______ (No:) ______ (No:)

(ii) **Newborn Health Statistics:**

S. No.	Questions	Last Year	Last 3 months
**4.11**	Number of low birth weight newborn babies (<2500g)		
**4.12**	Number of pre-term deliveries<37completed weeks		
**4.13**	Number of babies who required resuscitation (bag and mask ventilation)		
**4.14**	Number of neonatal deaths in the hospital		
**4.15**	Number of perinatal deaths (number of still births plus neonatal deaths in hospital)		
**4.16**	Number of Referrals received for neonatal cases		
**4.17**	Three major causes of neonatal deaths? (Specify reasons and numbers due to cause in last 1 year)	______ (No:) ______ (No:) ______ (No:)	______ (No:) ______ (No:) ______ (No:)
**4.18**	Three major causes of neonatal surgeries conducted? (Give reasons and numbers due to cause in last 1 year)	______ (No:) ______ (No:) ______ (No:)	______ (No:) ______ (No:) ______ (No:)

(iii) Child Health Statistics

Collect the information from registers (for last one month calendar).

S. No	Age Group	OPD Visits (A)		Emergency Visits (B)		Admission (D)		Deaths (E)			Referrals (F)	Age Specific Fatality Rate G)
		M	F	M	F	M	F	M	F	Reasons		
**4.19**	0-28 days											
**4.20**	1-12 months											
**4.21**	1-5 Years											
**4.22**	5-12 years											
**4.23**	Total											

5. Health Information System and Medical Records

(i) Health Information System

S.No.	Items	Presence/ Absence	Comment
**5.1**	Existence and use of a computer-based **(Record Maintainance)**information system on patient flow (admissions, outpatients, etc.)	☐ Yes ☐ No	
**5.2**	Existence and use of a computer-based information system on important medical indicators	☐ Yes ☐ No	
**5.3**	Existence and use of paper-based information system on patient flow (admissions, outpatients, etc.), if computer-based unavailable	☐ Yes ☐ No	
**5.4**	Existence and use of paper-based information system on important medical indicators, if computer-based unavailable	☐ Yes ☐ No	

(ii) Medical Records

Q. No.	Items in Labour room	Response	Comment
**5.5**	Are case records clear and legible?	☐ Yes ☐ No	
**5.6**	Are records dated?	☐ Yes ☐ No	
**5.7**	Are all admissions and discharge diagnoses clearly written in the notes?	☐ Yes ☐ No	
**5.8**	Are all drugs and treatments clearly identifiable in nurses/hospital records?	☐ Yes ☐ No	
**5.9**	Is information from previous admissions available to staff providing care to mothers, neonates and children?	☐ Yes ☐ No	
**5.10**	Is information from antenatal records available to staff providing care during labour?	☐ Yes ☐ No	
**5.11**	Are previous admission/ case records available to staff providing care during postpartum period?	☐ Yes ☐ No	

6. Hospital Administration

S. No.	Standards and Criterion	Response	Comments
(i) Availability of adequate and updated treatment guidelines			
6.1.	Guidelines for common conditions are available, wall charts, or job aids.	☐ Yes ☐ No	
6.2.	Recommended antibiotics for common infections and essential drugs list are available.	☐ Yes ☐ No	
6.3.	New-born resuscitation is described in wall charts	☐ Yes ☐ No	
6.4.	Management of Obstetric complications is described in wall charts	☐ Yes ☐ No	
(ii) Availability of Essentials			
6.5.	Essential drugs are always available and free	☐ Yes ☐ No	
6.6.	Essential equipment is available and functioning	☐ Yes ☐ No	
6.7.	Essential lab tests are available and delivered timely	☐ Yes ☐ No	
6.8.	Transport for referral is available	☐ Yes ☐ No	

Summary Score –General Information					
a. Total responses with YES/NO options ☐☐		b. Total Yes ☐☐		c. Score ☐☐.☐☐	
d. Scale (Circle)	**5** (95-100%)	**4** (75-94%)	**3** (51-75%)	**2** (26-50%)	**1** (< 25%)

Section B: Labour room

7. General infrastructure- Labour Room

S.no	Question	Response	Comments
**7.1.**	Is the facility has a labour cum delivery room available?	☐ Yes ☐ No	
**7.2.**	Is it open 24 hours	☐ Yes ☐ No	
**7.3.**	If not then for how many hours a day does it open	_______ hrs	
**7.4.**	On which floor is the labour room situated	☐	
**7.5.**	Is there availability of stretcher, ramps for the easy accessibility to labour room	☐ Yes ☐ No	
**7.6.**	Is there availability of lifts if the labour room is situated on the higher floor	☐ Yes ☐ No	
**7.7.**	Does the facility have waiting room for patients?	☐ Yes ☐ No	
**7.8.**	Is there a newborn care corner within Labour room?	☐ Yes ☐ No	
**7.9.**	Is a fire extinguisher installed anywhere in the labor room complex?	☐ Yes ☐ No	
**7.10.**	Is there a clear signage at entrance of the facility (near registration counter) to direct patients to LR?	☐ Yes ☐ No	
**7.11.**	Are names of Doctors, Nurses and ANMs on duty in LR displayed outside the LR?	☐ Yes ☐ No	
**7.12.**	Is the electricity connection same as with the other part of the hospital???	☐ Yes ☐ No	
**(i)**	**Electricity**		
**7.13.**	Does the facilities have 24 x 7 uninterrupted electricity supply?	☐ Yes ☐ No	
**7.14.**	Is the power cut predictable?	☐ Yes ☐ No	
**7.15.**	What is the average hour for power cut in a day?		

		________hours	
**7.16.**	Does the facilities have backup source for the uninterrupted supply of electrical power?	☐ Yes ☐ No	
**7.17.**	Did you have uninterupted power supply (atleast for 12 hours) during the last one month?	☐ Yes ☐ No	
**7.18.**	If NO , how many days?	Days ☐☐	
**(ii)**	**Water supply**		
**7.19.**	Does this facility have 24 x 7 water supply for functions ?	☐ Yes ☐ No	
**7.20.**	What is the main source of water of this facility?		
**7.21.**	Does this facility have uninturrupted water supply for last one month ?	☐ Yes ☐ No	
**7.22.**	If No, how many days?	☐☐	

(iii) Service delivery

S.no	Question	Response	Comments
**7.23.**	Who usually conducts the normal deliveries?	a) Specialist (Obstetrician) ☐<br>b) Doctor ☐<br>c) Staff Nurse/ ANM ☐<br>d) Other Staff ☐	
**7.24.**	Who usually conducts the high risk deliveries?	a) Specialist (Obstetrician) ☐<br>b) Doctor ☐<br>c) Staff Nurse/ ANM ☐<br>d) Other Staff ☐<br>e) NA (tick N/A if facility does not conduct caesarean section) ☐	
**7.25.**	Who usually conducts the Caesarean Section?	a) Specialist (Obstetrician) ☐<br>b) Doctor ☐<br>c) Staff Nurse/ ANM ☐<br>d) Other Staff ☐<br>e) NA (tick N/A if facility does not conduct caesarean section) ☐	
**7.26.**	Who usually conducts the operative vaginal deliveries like forceps and vacuum?	a) Specialist (Obstetrician) ☐<br>b) Doctor ☐<br>c) Staff Nurse/ ANM ☐<br>d) Other Staff ☐<br>e) NA (tick N/A if facility does not conduct caesarean section) ☐	
**7.27.**	MO provides the first contact care to the pregnant females?	☐ Yes ☐ No	
**7.28.**	Staff Nurse provides the first contact care to the pregnant females?	☐ Yes ☐ No	
**7.29.**	Has the first contact care staff received training in SBA/BEmOC?	☐ Yes ☐ No	

8. Equipments- Labour Room

(i) Maternal care

Q. No.	Equipment/ Supplies	Availability	Number	Functionality (Numbers)	Comments
**8.1.**	Normal Delivery Kit	☐ Yes ☐ No			
**8.2.**	Episiotomy kit	☐ Yes ☐ No			

**8.3.**	Forceps Delivery Kit	☐ Yes ☐ No			
**8.4.**	Vacuum extractor Malastrom	☐ Yes ☐ No			
**8.5.**	BP Apparatus & Stethoscope	☐ Yes ☐ No			
**8.6.**	Carditocograph	☐ Yes ☐ No			
**8.7.**	Fetal Doppler	☐ Yes ☐ No			
**8.8.**	Emergency Tray	☐ Yes ☐ No			
**8.9.**	Oxygen source, delivery system	☐ Yes ☐ No			
**8.10.**	Autoclaving /sterilization unit	☐ Yes ☐ No			
**8.11.**	Resuscitation Kit for adults	☐ Yes ☐ No			
**8.12.**	laryngoscope	☐ Yes ☐ No			
**8.13.**	Diathermy machine	☐ Yes ☐ No			
**8.14.**	Shadow less lamps	☐ Yes ☐ No			
**8.15.**	Standard Surgical Set	☐ Yes ☐ No			
**8.16.**	IUD Insertion Kit	☐ Yes ☐ No			
**8.17.**	Suction Apparatus	☐ Yes ☐ No			
**8.18.**	Table Operation, Hydraulic	☐ Yes ☐ No			
**8.19.**	Trolley for patients	☐ Yes ☐ No			
**8.20.**	Color Doppler Ultrasound	☐ Yes ☐ No			
**8.21.**	ECG machine	☐ Yes ☐ No			
**8.22.**	Cardiac monitors	☐ Yes ☐ No			
**8.23.**	Partograph charts	☐ Yes ☐ No			
**Newborn Care**					
**8.24.**	Resuscitation Kit	☐ Yes ☐ No			
**8.25.**	Radiant warmers	☐ Yes ☐ No			
**8.26.**	Pulse Oximeter	☐ Yes ☐ No			
**8.27.**	Weighing scales for children	☐ Yes ☐ No			

**8.28.**	Infantometer	☐ Yes ☐ No			
**8.29.**	Light examination, mobile	☐ Yes ☐ No			
**8.30.**	Thermometers	☐ Yes ☐ No			
**8.31.**	Pediatric Laryngoscope set & cells	☐ Yes ☐ No			
**8.32.**	Flow meters and humidifiers for oxygen	☐ Yes ☐ No			
**8.33.**	Self-inflating bags (0250 ml, 500 ml	☐ Yes ☐ No			
**8.34.**	Glucometer	☐ Yes ☐ No			
**8.35.**	Lamp, ultra- violet	☐ Yes ☐ No			
**8.36.**	Nebulizers	☐ Yes ☐ No			
**8.37.**	Fetal Doppler	☐ Yes ☐ No			
**8.38.**	Stethoscope	☐ Yes ☐ No			
**8.39.**	Suction Equipment (Catheter 6,8, 10 FG)	☐ Yes ☐ No			
**8.40.**	Oxygen Catheter 8 F, Oxygen cylinder	☐ Yes ☐ No			
**8.41.**	Nasal Prongs	☐ Yes ☐ No			
**8.42.**	Nasal Catheters	☐ Yes ☐ No			
**8.43.**	Infant size mask	☐ Yes ☐ No			
**8.44.**	NG tubes (8,10, 12FG)	☐ Yes ☐ No			
**8.45.**	IV sets with chambers	☐ Yes ☐ No			
**8.46.**	Mucus extractor 20 ml	☐ Yes ☐ No			
**8.47.**	IV cannulas(24G, 26G)	☐ Yes ☐ No			
**8.48.**	Glucostix/ multistix	☐ Yes ☐ No			
**8.49.**	Feeding tube, CH07, L40 cm	☐ Yes ☐ No			
**8.50.**	Sterile gloves	☐ Yes ☐ No			
**8.51.**	Disinfectant, 20% Chlorhexidine	☐ Yes ☐ No			

9. DRUGS & CONSUMABLES AND VACCINES

(Methodology: observation, Record review, Provider interaction in the Labour room & Medical store)

Availability of Labour Room Trays *(Observation)*

S.NO	Labour Room Trays	Availability	Total Number of Trays	Functionality (Numbers)	Comments
9.1	**Delivery Tray**	☐ Yes ☐ No			
9.2	**Episiotomy Tray**	☐ Yes ☐ No			
9.3	**Baby Tray**	☐ Yes ☐ No			
9.4	**Medicine Tray**	☐ Yes ☐ No			
9.5	**Emergency Drug Tray**	☐ Yes ☐ No			
9.6	**MVA/ EVA tray**	☐ Yes ☐ No			
9.7	**PPIUCD Tray**	☐ Yes ☐ No			

S.NO	Labour Room Trays				
**9.1**	**Delivery tray**	**Available**	**Designated tray**	**Functional**	**Comments**
**9.1.1**	Scissors	☐ Yes ☐ No	☐ Yes ☐ No	☐ Yes ☐ No	
**9.1.2**	Artery forceps	☐ Yes ☐ No	☐ Yes ☐ No	☐ Yes ☐ No	
**9.1.3**	Sponge holding forceps	☐ Yes ☐ No	☐ Yes ☐ No	☐ Yes ☐ No	
**9.1.4**	Speculum	☐ Yes ☐ No	☐ Yes ☐ No	☐ Yes ☐ No	
**9.1.5**	Urinary catheter	☐ Yes ☐ No	☐ Yes ☐ No	☐ Yes ☐ No	
**9.1.6**	BP blade / surgical blade for cutting cord	☐ Yes ☐ No	☐ Yes ☐ No	☐ Yes ☐ No	
**9.1.7**	Bowl for antiseptic solution	☐ Yes ☐ No	☐ Yes ☐ No	☐ Yes ☐ No	
**9.1.8**	kidney tray	☐ Yes ☐ No	☐ Yes ☐ No	☐ Yes ☐ No	
**9.1.9**	Gauze pieces; cotton swabs	☐ Yes ☐ No	☐ Yes ☐ No	☐ Yes ☐ No	
**9.1.10**	Sanitary pads	☐ Yes ☐ No	☐ Yes ☐ No	☐ Yes ☐ No	
**9.1.11**	Gloves	☐ Yes ☐ No	☐ Yes ☐ No	☐ Yes ☐ No	
**9.2**	**Episiotomy Tray**	**Available**	**Designated tray**	**Functional**	**Comment**
**9.2.1**	Inj. Xylocaine 2%	☐ Yes ☐ No	☐ Yes ☐ No	☐ Yes ☐ No	

**9.2.2**	10 ml disposable syringe with needle,	☐ Yes ☐ No	☐ Yes ☐ No	☐ Yes ☐ No	
**9.2.3**	Episiotomy scissor	☐ Yes ☐ No	☐ Yes ☐ No	☐ Yes ☐ No	
**9.2.4**	Kidney tray	☐ Yes ☐ No	☐ Yes ☐ No	☐ Yes ☐ No	
**9.2.5**	Artery forceps	☐ Yes ☐ No	☐ Yes ☐ No	☐ Yes ☐ No	
**9.2.6**	Allis forceps	☐ Yes ☐ No	☐ Yes ☐ No	☐ Yes ☐ No	
**9.2.7**	Sponge holding forceps	☐ Yes ☐ No	☐ Yes ☐ No	☐ Yes ☐ No	
**9.2.8**	Toothed forceps	☐ Yes ☐ No	☐ Yes ☐ No	☐ Yes ☐ No	
**9.2.9**	Needle holder	☐ Yes ☐ No	☐ Yes ☐ No	☐ Yes ☐ No	
**9.2.10**	Needle (round body and cutting)	☐ Yes ☐ No	☐ Yes ☐ No	☐ Yes ☐ No	
**9.2.11**	Chromic catgut no. 0	☐ Yes ☐ No	☐ Yes ☐ No	☐ Yes ☐ No	
**9.2.12**	Gauze pieces	☐ Yes ☐ No	☐ Yes ☐ No	☐ Yes ☐ No	
**9.2.13**	Cotton swabs	☐ Yes ☐ No	☐ Yes ☐ No	☐ Yes ☐ No	
**9.2.14**	Antiseptic lotion	☐ Yes ☐ No	☐ Yes ☐ No	☐ Yes ☐ No	
**9.2.15**	Thumb forceps	☐ Yes ☐ No	☐ Yes ☐ No	☐ Yes ☐ No	
**9.2.16**	Gloves	☐ Yes ☐ No	☐ Yes ☐ No	☐ Yes ☐ No	
**9.3**	**Baby Tray**	**Available**	**Designated tray**	**Functional**	**Comment**
**9.3.1**	Two pre-warmed towels/sheets for wrapping the baby,	☐ Yes ☐ No	☐ Yes ☐ No	☐ Yes ☐ No	
**9.3.2**	Cotton swabs,	☐ Yes ☐ No	☐ Yes ☐ No	☐ Yes ☐ No	
**9.3.3**	Mucus extractor,	☐ Yes ☐ No	☐ Yes ☐ No	☐ Yes ☐ No	
**9.3.4**	Bag & mask,	☐ Yes ☐ No	☐ Yes ☐ No	☐ Yes ☐ No	
**9.3.5**	Sterilized thread for cord/cord clamp,	☐ Yes ☐ No	☐ Yes ☐ No	☐ Yes ☐ No	
**9.3.6**	Nasogastric tube and gloves	☐ Yes ☐ No	☐ Yes ☐ No	☐ Yes ☐ No	

**9.3.7**	Inj. Vitamin K,	☐ Yes ☐ No	☐ Yes ☐ No	☐ Yes ☐ No	
**9.3.8**	Needle and syringe	☐ Yes ☐ No	☐ Yes ☐ No	☐ Yes ☐ No	
**9.4**	**Medicine tray**	**Available**	**Designated tray**	**Functional**	**Comment**
**9.4.1**	Inj. Oxytocin (to be kept in fridge)	☐ Yes ☐ No	☐ Yes ☐ No	☐ Yes ☐ No	
**9.4.2**	Inj. Oxytocin 10 IU	☐ Yes ☐ No	☐ Yes ☐ No	☐ Yes ☐ No	
**9.4.3**	Inj. Gentamycin	☐ Yes ☐ No	☐ Yes ☐ No	☐ Yes ☐ No	
**9.4.4**	Inj. Betamethason	☐ Yes ☐ No	☐ Yes ☐ No	☐ Yes ☐ No	
**9.4.5**	Inj. Hydrazaline	☐ Yes ☐ No	☐ Yes ☐ No	☐ Yes ☐ No	
**9.4.6**	InjVit K	☐ Yes ☐ No	☐ Yes ☐ No	☐ Yes ☐ No	
**9.4.7**	Tab Nefidepin	☐ Yes ☐ No	☐ Yes ☐ No	☐ Yes ☐ No	
**9.4.8**	Tab Methyldopa	☐ Yes ☐ No	☐ Yes ☐ No	☐ Yes ☐ No	
**9.4.9**	Tab Metronidazole 400 mg	☐ Yes ☐ No	☐ Yes ☐ No	☐ Yes ☐ No	
**9.4.10**	Tab Paracetamol	☐ Yes ☐ No	☐ Yes ☐ No	☐ Yes ☐ No	
**9.4.11**	Tab Ibuprofen	☐ Yes ☐ No	☐ Yes ☐ No	☐ Yes ☐ No	
**9.4.12**	Tab B complex	☐ Yes ☐ No	☐ Yes ☐ No	☐ Yes ☐ No	
**9.4.13**	Tab. Misoprostol 200 mg	☐ Yes ☐ No	☐ Yes ☐ No	☐ Yes ☐ No	
**9.4.14**	Cap Ampicillin 500 mg	☐ Yes ☐ No	☐ Yes ☐ No	☐ Yes ☐ No	
**9.4.15**	IV fluids	☐ Yes ☐ No	☐ Yes ☐ No	☐ Yes ☐ No	
**9.4.16**	Ringer lactate	☐ Yes ☐ No	☐ Yes ☐ No	☐ Yes ☐ No	
**9.4.17**	Normal Saline	☐ Yes ☐ No	☐ Yes ☐ No	☐ Yes ☐ No	
**9.4.18**	Magnifying glass.	☐ Yes ☐ No	☐ Yes ☐ No	☐ Yes ☐ No	
**9.5**	**Emergency Drug Tray**	**Available**	**Designated tray**	**Functional**	**Comment**
**9.5.1**	Inj. Oxytocin (to be kept in fridge)	☐ Yes ☐ No	☐ Yes ☐ No	☐ Yes ☐ No	
**9.5.2**	Inj. Magsulf 50%	☐ Yes ☐ No	☐ Yes ☐ No	☐ Yes ☐ No	
**9.5.3**	Inj. Calcium gluconate-10%	☐ Yes ☐ No	☐ Yes ☐ No	☐ Yes ☐ No	
**9.5.4**	Inj. Dexamethasone	☐ Yes ☐ No	☐ Yes ☐ No	☐ Yes ☐ No	
**9.5.5**	Inj. Ampicillin	☐ Yes ☐ No	☐ Yes ☐ No	☐ Yes ☐ No	
**9.5.6**	Inj. Gentamicin	☐ Yes ☐ No	☐ Yes ☐ No	☐ Yes ☐ No	
**9.5.7**	Inj. Metronidazole	☐ Yes ☐ No	☐ Yes ☐ No	☐ Yes ☐ No	

**9.5.8**	Inj. Lignocaine-2%	☐ Yes ☐ No	☐ Yes ☐ No	☐ Yes ☐ No	
**9.5.9**	Inj. Adrenaline	☐ Yes ☐ No	☐ Yes ☐ No	☐ Yes ☐ No	
**9.5.10**	Inj. Hydrocortisone Succinate	☐ Yes ☐ No	☐ Yes ☐ No	☐ Yes ☐ No	
**9.5.11**	Inj. Diazepam	☐ Yes ☐ No	☐ Yes ☐ No	☐ Yes ☐ No	
**9.5.12**	Inj. Pheneramine maleate	☐ Yes ☐ No	☐ Yes ☐ No	☐ Yes ☐ No	
**9.5.13**	Inj. Carboprost	☐ Yes ☐ No	☐ Yes ☐ No	☐ Yes ☐ No	
**9.5.14**	Inj. Fortwin	☐ Yes ☐ No	☐ Yes ☐ No	☐ Yes ☐ No	
**9.5.15**	Inj. Phenergan	☐ Yes ☐ No	☐ Yes ☐ No	☐ Yes ☐ No	
**9.5.16**	Ringer lactate	☐ Yes ☐ No	☐ Yes ☐ No	☐ Yes ☐ No	
**9.5.17**	Normal saline	☐ Yes ☐ No	☐ Yes ☐ No	☐ Yes ☐ No	
**9.5.18**	Inj. Betamexthazon	☐ Yes ☐ No	☐ Yes ☐ No	☐ Yes ☐ No	
**9.5.19**	Inj. Hydrazaline	☐ Yes ☐ No	☐ Yes ☐ No	☐ Yes ☐ No	
**9.5.20**	Tab Nefidepin	☐ Yes ☐ No	☐ Yes ☐ No	☐ Yes ☐ No	
**9.5.21**	Tab Methyldopa	☐ Yes ☐ No	☐ Yes ☐ No	☐ Yes ☐ No	
**9.5.22**	IV sets with 16-gauge needle at least two,	☐ Yes ☐ No	☐ Yes ☐ No	☐ Yes ☐ No	
**9.5.23**	Controlled suction catheter,	☐ Yes ☐ No	☐ Yes ☐ No	☐ Yes ☐ No	
**9.5.24**	Mouth gag,	☐ Yes ☐ No	☐ Yes ☐ No	☐ Yes ☐ No	
**9.5.25**	IV Canula,	☐ Yes ☐ No	☐ Yes ☐ No	☐ Yes ☐ No	
**9.5.26**	Vials for drug collection Ceftriaxone (3rd generation cephalosporins) - For L3 facility	☐ Yes ☐ No	☐ Yes ☐ No	☐ Yes ☐ No	
**9.6**	**MVA/ EVA tray**	**Available**	**Designated tray**	**Functional**	**Comment**
**9.6.1**	Gloves	☐ Yes ☐ No	☐ Yes ☐ No	☐ Yes ☐ No	
**9.6.2**	Speculum	☐ Yes ☐ No	☐ Yes ☐ No	☐ Yes ☐ No	
**9.6.3**	Anterior vaginal wall retractor	☐ Yes ☐ No	☐ Yes ☐ No	☐ Yes ☐ No	
**9.6.4**	Posterior vaginal wall retractor	☐ Yes ☐ No	☐ Yes ☐ No	☐ Yes ☐ No	
**9.6.5**	Sponge holding forceps	☐ Yes ☐ No	☐ Yes ☐ No	☐ Yes ☐ No	
**9.6.6**	MVA syringe and cannulas	☐ Yes ☐ No	☐ Yes ☐ No	☐ Yes ☐ No	

**9.6.7**	MTP cannulas	☐ Yes ☐ No	☐ Yes ☐ No	☐ Yes ☐ No	
**9.6.8**	Small bowl of antiseptic lotion	☐ Yes ☐ No	☐ Yes ☐ No	☐ Yes ☐ No	
**9.6.9**	Sanitary pads	☐ Yes ☐ No	☐ Yes ☐ No	☐ Yes ☐ No	
**9.6.10**	Pads /cotton swabs	☐ Yes ☐ No	☐ Yes ☐ No	☐ Yes ☐ No	
**9.6.11**	Disposable syringe and needle	☐ Yes ☐ No	☐ Yes ☐ No	☐ Yes ☐ No	
**9.6.12**	Misoprostol tablet	☐ Yes ☐ No	☐ Yes ☐ No	☐ Yes ☐ No	
**9.6.13**	Sterilized gauze/pads	☐ Yes ☐ No	☐ Yes ☐ No	☐ Yes ☐ No	
**9.6.14**	Urinary catheter	☐ Yes ☐ No	☐ Yes ☐ No	☐ Yes ☐ No	
**9.7**	**PPIUCD tray**	**Available**	**Designated tray**	**Functional**	**Comments**
**9.7.1**	PPIUCD insertion forceps	☐ Yes ☐ No	☐ Yes ☐ No	☐ Yes ☐ No	
**9.7.2**	Sims speculum	☐ Yes ☐ No	☐ Yes ☐ No	☐ Yes ☐ No	
**9.7.3**	Sponge holding forceps	☐ Yes ☐ No	☐ Yes ☐ No	☐ Yes ☐ No	
**9.7.4**	Cu IUCD 380A in sterile pack	☐ Yes ☐ No	☐ Yes ☐ No	☐ Yes ☐ No	
**9.7.5**	Cu IUCD 375 in sterile pack	☐ Yes ☐ No	☐ Yes ☐ No	☐ Yes ☐ No	

10. Staffing (Labour Room)

(i) Availability of staffs in labour room

**SL No**	**Type of staff**	**Sanctioned number**	**In-position number**	**Number during any shift on weekdays**			**On holiday**	**Any deficiency in any area (OT/IL.R/Ward /OPD (Yes/No)**
				**M**	**E**	**N**		
**10.1**	Doctor (Gynaecologist)							
**10.2**	Medical officers							
**10.3**	Staff nurse							
**10.4**	ANM							
**10.5**	Multi-skilled Group D worker							
**10.6**	Sanitary worker							
**10.7**	Watchman							
**10.8**								
**10.9**								
**10.10**								

S.No	(ii). Some Specific details of Staffs (For Labor Room)											
10.11	Staff	a. Posted since (MM/YY)	b.Full time avaiable in facility	c. Residential	d.If no, Distance from residence	Training						
						e. SBA	f.BEmOC	g.NSSK	h.MTP	i.RTI/STI	j.IUCD	k.Any other training
10.11.1	Designation<br>Name	☐☐☐☐	YES ☐ NO ☐	YES ☐ NO ☐	_._ KMs	YES ☐ NO ☐	YES ☐ NO ☐	YES ☐ NO ☐	YES ☐ NO ☐	YES ☐ NO ☐	YES ☐ NO ☐	
10.11.2	Designation<br>Name	☐☐☐☐	YES ☐ NO ☐	YES ☐ NO ☐	_._ KMs	YES ☐ NO ☐	YES ☐ NO ☐	YES ☐ NO ☐	YES ☐ NO ☐	YES ☐ NO ☐	YES ☐ NO ☐	
10.11.3	Designation<br>Name	☐☐☐☐	YES ☐ NO ☐	YES ☐ NO ☐	_._ KMs	YES ☐ NO ☐	YES ☐ NO ☐	YES ☐ NO ☐	YES ☐ NO ☐	YES ☐ NO ☐	YES ☐ NO ☐	
10.11.4	Designation<br>Name	☐☐☐☐	YES ☐ NO ☐	YES ☐ NO ☐	_._ KMs	YES ☐ NO ☐	YES ☐ NO ☐	YES ☐ NO ☐	YES ☐ NO ☐	YES ☐ NO ☐	YES ☐ NO ☐	
10.11.5	Designation<br>Name	☐☐☐☐	YES ☐ NO ☐	YES ☐ NO ☐	_._ KMs	YES ☐ NO ☐	YES ☐ NO ☐	YES ☐ NO ☐	YES ☐ NO ☐	YES ☐ NO ☐	YES ☐ NO ☐	
10.11.6	Designation<br>Name	☐☐☐☐	YES ☐ NO ☐	YES ☐ NO ☐	_._ KMs	YES ☐ NO ☐	YES ☐ NO ☐	YES ☐ NO ☐	YES ☐ NO ☐	YES ☐ NO ☐	YES ☐ NO ☐	
10.11.7	Designation<br>Name	☐☐☐☐	YES ☐ NO ☐	YES ☐ NO ☐	_._ KMs	YES ☐ NO ☐	YES ☐ NO ☐	YES ☐ NO ☐	YES ☐ NO ☐	YES ☐ NO ☐	YES ☐ NO ☐	
10.11.8	Designation<br>Name	☐☐☐☐	YES ☐ NO ☐	YES ☐ NO ☐	_._ KMs	YES ☐ NO ☐	YES ☐ NO ☐	YES ☐ NO ☐	YES ☐ NO ☐	YES ☐ NO ☐	YES ☐ NO ☐	

11. Infection control practices- Labour Room

(i) Hand washing

Q. no.	Items in Facility	Labour room	Comments
11.1	Hand washing stations are well organized and equipped?	☐ Yes ☐ No	
11.2	Is there availability of running water?	☐ Yes ☐ No	
11.3	Are there soap and/or disinfectant available f or hand washing?	☐ Yes ☐ No	
11.4	Written protocols on hygiene for hands are available near scrub area/ hand wash area?	☐ Yes ☐ No	
**(ii)**	**Use of gloves**		
11.5	Sterile gloves are used for conducting delivery?	☐ Yes ☐ No	
11.6	Gloves are disposed after used according to the waste management protocol	☐ Yes ☐ No	
**(iii)**	**Infection control Practices**		
11.7	Is there a routine disinfection of the labour room?	☐ Yes ☐ No	
11.8	Caps, masks and shoe covers are routinely used by staff	☐ Yes ☐ No	
11.9	There is an infection control policy for visitors in the hospital, that may require restriction of access to patients	☐ Yes ☐ No	
11.10	Is waste disposal and segregation done as per guidelines?	☐ Yes ☐ No	
11.11	Availability of elbow operated taps	☐ Yes ☐ No	
11.12	Heavy duty gloves and gum boats for housekeeping staff	☐ Yes ☐ No	
11.13	Personal protective kit for delivering HIV patients	☐ Yes ☐ No	

12. Case management- Labour Room

(i) Care throughout labour

S.no	Questions	Response	Comments
12.1	There is at least one skilled service provider present throughout labor and birth.	☐ Yes ☐ No	
12.2	A supportive, encouraging atmosphere for birth	☐ Yes ☐ No	
12.3	Partogram is used consistently and for decision making	☐ Yes ☐ No	
**(ii)**	**Care during first stage of labour**		
12.4	Temperature and blood pressure are monitored 4 hourly and pulse on an hourly basis	☐ Yes ☐ No	
12.5	PV is done 4 hourly in active phase	☐ Yes ☐ No	
12.6	Progress of labor is assessed correctly and appropriate actions taken	☐ Yes ☐ No	
**(iii)**	**Care during second stage of labour**		
12.7	Episiotomy is NOT routinely performed	☐ Yes ☐ No	
12.8	Local anaesthesia is given if episiotomy is performed	☐ Yes ☐ No	
12.9	10 IU Oxytocin IM are given to the mother immediately after delivery	☐ Yes ☐ No	
**(iv)**	**Care during third stage of labour**		
12.10	controlled cord traction practiced for placenta delivery	☐ Yes ☐ No	
12.11	Delayed Cord Clamping done	☐ Yes ☐ No	
12.12	Placenta and membranes are checked for completeness	☐ Yes ☐ No	
**(v)**	**Care of mother immediately after delivery**		
12.13	Blood Pressure monitored regularly	☐ Yes ☐ No	

**12.14**	Temperature monitored regularly	☐ Yes ☐ No	
**12.15**	Vaginal bleeding monitored regularly	☐ Yes ☐ No	
**12.16**	Pulse monitored regularly	☐ Yes ☐ No	
**12.17**	Contraction of uterus monitored regularly	☐ Yes ☐ No	
**(vi)**	**Post-Partum Haemorrhage**		
**12.18**	Guidelines and a written protocol for preventing and managing PPH is available, known and used by skilled birth attendants	☐ Yes ☐ No	
**12.19**	Blood can be obtained without delay 24 hours a day	☐ Yes ☐ No	
**(vii)**	**Management of pre-term labour**		
**12.20**	Do you manage pregnant women in preterm labor here?	☐ Yes ☐ No	
**12.21**	Do you give antenatal steroids to such cases?	☐ Yes ☐ No	
**12.22**	Guidelines and written protocol with indications of antenatal steroid are available, known and used	☐ Yes ☐ No	
**(viii)**	**Management of Caesarean Section**		
**12.23**	Anesthesiologist, Obstetrician and operation theatre staff are available	☐ Yes ☐ No	
**12.24**	An operation theatre is 24 hours/7 days fully ready for caesarean sections	☐ Yes ☐ No	
**12.25**	Stored blood is readily available in case blood transfusion needed	☐ Yes ☐ No	
**12.26**	Cases for caesarean section are reviewed before carrying out the procedure by operating obstetrician	☐ Yes ☐ No	
**12.27**	Guidelines and written protocol (or checklist) for caesarean section is available, known and used by staff	☐ Yes ☐ No	
**12.28**	The woman´s vital signs are monitored throughout the procedure and recorded	☐ Yes ☐ No	
**12.29**	After recovery from anesthesia, the vital signs are monitored closely and recorded	☐ Yes ☐ No	
**(ix)**	**Management of unsatisfactory progress of labour**		
**12.30**	Unsatisfactory progress of labor is diagnosed using partograph	☐ Yes ☐ No	
**12.31**	The patient is re-hydrated	☐ Yes ☐ No	
**12.32**	Uterine contractions assessed on partograph	☐ Yes ☐ No	
**12.33**	Assessed for cephalo-pelvic disproportion	☐ Yes ☐ No	
**12.34**	Caesarean section is performed if ACTION line on Partograph is crossed	☐ Yes ☐ No	
**(x)**	**Management of severe anemia in pregnancy**		
**12.35**	Haemoglobin test is routinely done for all pregnant women attending for delivery	☐ Yes ☐ No	
**12.36**	Guidelines and a written protocol for management of anemia is available, known and routinely used by staff.	☐ Yes ☐ No	
**12.37**	All pregnant women with POG 30-36 weeks with severe anemia Hb<7 are to be given Iron Sucrose infusion	☐ Yes ☐ No	
**12.38**	All pregnant women with PoG>36 weeks with severe anemia Hb<7 are to be given blood transfusion	☐ Yes ☐ No	
**(xi)**	**Management of Postpartum Haemorrhage**		
**12.39**	Rapid infusion of RL/ NS 1litre over 15-20 min given	☐ Yes ☐ No	
**12.40**	Oxygen inhalation by mask	☐ Yes ☐ No	
**12.41**	Bladder catheterization, measurement of urine output	☐ Yes ☐ No	
**12.42**	Inj Oxytocin 10 IU IM and start Inj Oxytocin infusion 20 IU in 500 ml RL @ 40-60 drops/ min given	☐ Yes ☐ No	

**12.43**	Vitals monitored every 15 minutes	☐ Yes ☐ No	
**12.44**	Cause of PPH determined & specific management done	☐ Yes ☐ No	
**12.45**	Bimanual Compression of Uterus given	☐ Yes ☐ No	
**(xii)**	**Management of Hypertension in pregnancy**		
**12.46**	Guidelines& written protocol for management of pregnant female with hypertension are used	☐ Yes ☐ No	
**12.47**	BP is regularly monitored and appropriate classification of HT done	☐ Yes ☐ No	
**12.48**	Urine protein checked	☐ Yes ☐ No	
**12.49**	In cases of severe pre-eclampsia/ eclampsia, Magnesium Sulphate is given- 5 g IM in alternate buttocks every four hours for 24 hours after birth/ last convulsion	☐ Yes ☐ No	
**12.50**	Women with pre-eclampsia or gestational hypertension at term, induction of labour is recommended.	☐ Yes ☐ No	
**(xiii)**	**Management protocol during pregnancy and delivery**		
**12.51**	Standard Treatment Protocol/ guidelines are available and is used by the health care staff for the management of obstetric complications and delivery	☐ Yes ☐ No	
**12.52**	Is job description of hospital staff (doctors, nurses, paramedical) defined and communicated	☐ Yes ☐ No	
**12.53**	Is the duty roster of hospital staff (doctors, nurses, paramedical) prepared, updated and communicated	☐ Yes ☐ No	
**12.54**	Labor ward teams use childbirth checklist	☐ Yes ☐ No	
**12.55**	Guidelines and a written protocol for delivery are available, known and used by staff	☐ Yes ☐ No	
**12.56**	Appropriate admission history is taken and recorded in case sheet	☐ Yes ☐ No	
**(xiv)**	**Antenatal administration of corticosteroids**		
**12.57**	If less than 34 weeks gestation, corticosteroids are given to mother to improve foetal lung maturity & chances of neonatal survival:<br>- Dexamethasone 6 mg IM, four doses 12 hours apart	☐ Yes ☐ No	
**(xv)**	**Management of – Infections in pregnancy**		
**12.58**	Adequate antibiotics are given in case of preterm pre labour rupture of membranes	☐ Yes ☐ No	
**12.59**	Urine M/E done to screen for asymptomatic bacteriruria	☐ Yes ☐ No	
**12.60**	Appropriate antibiotics are given to women with infection	☐ Yes ☐ No	

13. Newborn care at birth

S. No.	Questions	Response	Comments
**(i)**	**Neonatal resuscitation**		
**13.1**	Is there a separate area defined for neonatal resuscitation	☐ Yes ☐ No	
**13.2**	Written guidelines for resuscitation and care of the new born are available and documented	☐ Yes ☐ No	
**13.3**	Is there a doctor available 24 hours who is trained in NRP	☐ Yes ☐ No	
**13.4**	Is there a staff nurse available 24 hours who is trained in NRP	☐ Yes ☐ No	
**13.5**	Are the equipment for resuscitation available	☐ Yes ☐ No	
**13.6**	Are all the equipment functional	☐ Yes ☐ No	

**13.7**	Are there job aids for NRP displayed at NBSU	☐ Yes ☐ No	
**(ii)**	**Routine Neonatal care (NBCC)**		
**13.8**	APGAR Score is documented for all	☐ Yes ☐ No	
**13.9**	Kangaroo Mother Care is encouraged for LBW newborns	☐ Yes ☐ No	
**(iii)**	**Early and exclusive breast feeding and skin contact is ensured**		
**13.10**	Skin to skin contact with mother initiated within the first ½hour	☐ Yes ☐ No	
**13.11**	Breast feeding started within 1 hour of birth	☐ Yes ☐ No	
**13.12**	There is no promotion of infant formula on the ward or distributed to mothers	☐ Yes ☐ No	
**13.13**	Mothers stay with their infants in the same room day and night	☐ Yes ☐ No	
**(iv)**	**Babies are kept Warm, vaccination and immunization given**		
**13.14**	Newborns are kept in a warm room, with no draught	☐ Yes ☐ No	
**13.15**	Newborns are cleaned with dry/warm cloth, no bathing or washing given in first 24 hours	☐ Yes ☐ No	
**13.16**	Body temperature is monitored regularly	☐ Yes ☐ No	
**13.17**	Vitamin K prophylaxis at birth given for all newborns	☐ Yes ☐ No	
**13.18**	Immunizations are given (Zero OPV, Hep B and BCG) for all newborns	☐ Yes ☐ No	

14. Display of Protocols in LR (appropriate for the level of facility) - Are the following protocols displayed

**S. No**	**Display of Protocols in LR**	**Response**	**Comments**
**14.1**	Simplified Partograph	☐ Yes ☐ No	
**14.2**	Vaginal bleeding before 20 weeks	☐ Yes ☐ No	
**14.3**	Vaginal bleeding after 20 weeks	☐ Yes ☐ No	
**14.4**	Management of PPH	☐ Yes ☐ No	
**14.5**	Eclampsia	☐ Yes ☐ No	
**14.6**	AMTSL	☐ Yes ☐ No	
**14.7**	New born resuscitation	☐ Yes ☐ No	
**14.8**	Kangaroo Mother Care	☐ Yes ☐ No	
**14.9**	Breastfeeding	☐ Yes ☐ No	
**14.10**	Hand washing	☐ Yes ☐ No	
**14.11**	Preparation of 1 litre bleaching soln.	☐ Yes ☐ No	

**14.12**	Infection prevention	☐ Yes ☐ No	
**14.13**	Processing of used items	☐ Yes ☐ No	
**14.14**	LR Sterilization	☐ Yes ☐ No	
**14.15**	Management of atonic PPH	☐ Yes ☐ No	
**14.16**	Whether JSY entitlements displayed (wall painting/banner etc) in admission/OPD area?	☐ Yes ☐ No	
**14.17**	Whether JSSK entitlements displayed (wall painting/banner etc) in admission/OPD area?	☐ Yes ☐ No	
**14.18**	Whether Referral Transport Details displayed (wall painting/banner etc) in admission/OPD area?	☐ Yes ☐ No	

15. Documentation & Records

Available and Updated for last 3 calendar months*(Methodology: Direct Observation)*

S.no	Records / Documents	Available	Updated	Comments
15.1	**Labour Room Register**	☐Yes ☐ No	☐Yes ☐ No	
15.2	**Partograph in case sheets**	☐Yes ☐ No	☐Yes ☐ No	
15.3	**Referral Register (In)**	☐Yes ☐ No	☐Yes ☐ No	
15.4	**Referral Register (Out)**	☐Yes ☐ No	☐Yes ☐ No	
15.5	**Referral Slip**	☐Yes ☐ No	☐Yes ☐ No	
15.6	**Maternal death record Register**	☐Yes ☐ No	☐Yes ☐ No	
15.7	**Discharge Register**	☐Yes ☐ No	☐Yes ☐ No	
15.8	**Discharge Slip**	☐Yes ☐ No	☐Yes ☐ No	
15.9	**PPIUCD Register**	☐Yes ☐ No	☐Yes ☐ No	
15.10	**LR sterilization Register**	☐Yes ☐ No	☐Yes ☐ No	
15.11	**Handing over-taking over Register**	☐Yes ☐ No	☐Yes ☐ No	
15.12	**PNC Register**	☐Yes ☐ No	☐Yes ☐ No	
15.13	**FP register**	☐Yes ☐ No	☐Yes ☐ No	

Summary Score –Labor room					
a. Total responses with YES/NO options ☐☐		b. Total Yes ☐☐		c. Score ☐☐.☐☐	
d.Scale (Circle) ☐☐	**5** **(**95-100%)	**4** **(**75-94%)	**3** **(**51-75%)	**2** **(**26-50%)	**1** **(< 25%)**

SECTION C: OPERATION THEATRE

16. OT Infrastructure

S.no	Question	Response	Comments
**16.1**	Does the facility have operation theatre (OT)	☐ Yes ☐ No	
**16.2**	If not, what are the issues?		
**16.3**	Are surgeries carried out at present?	☐ Yes ☐ No	
**16.4**	If operation theatre is present and surgeries are not carried out, what is the reason?	*Non-availability of doctors* ☐<br>*Lack of equipment* ☐<br>*Poor condition of facility* ☐<br>*No power supply* ☐<br>*Other* ☐<br>*N/A* ☐<br>*Specify other*	
**16.5**	Is Operation theatre used for obstetric / gynecological/ cesarean section purpose	☐ Yes ☐ No	
**16.6**	Does the facility conduct surgery in children?	☐ Yes ☐ No	
**16.7**	Does the facility conduct surgery in newborns?	☐ Yes ☐ No	
**16.8**	Does the facility have minor OT / dressing room	☐ Yes ☐ No	
**16.9**	Does the facility has protocols for handling equipments	☐ Yes ☐ No	
**16.10**	Does the facility has procedures manual for Infection prevention and control	☐ Yes ☐ No	
	**(i) Cleanliness**		
**16.11**	Is there separate functional Public Utilities for Male and Female near or within the OT area?	☐ Yes ☐ No	
**16.12**	Is the overall cleaniness of the toiltes good?	☐ Yes ☐ No	
**16.13**	Is the overall cleaninees of OT good?	☐ Yes ☐ No	
**16.14**	Does the facility has appropriate and functional hand washing facility in the patient care area?	☐ Yes ☐ No	
	**(ii) Waste management**		
**16.15**	Do you have system for segregation of waste?	☐ Yes ☐ No	
**16.16**	Are there color coded bins available for waste segregation?	☐ Yes ☐ No	
**16.17**	Is there display of instructions for management of BMW	☐ Yes ☐ No	

Summary Score –Operation theatre					
a. Total responses with YES/NO options ☐☐		b. Total Yes ☐☐		c.Score ☐☐.☐☐	
d.Scale (Circle)	**5** **(**95-100%)	**4** **(**75-94%)	**3** **(**51-75%)	**2** **(**26-50%)	**1** **(< 25%)**

Section –D: Ward

17. Maternal ward

General infrastructure- Maternal ward

S.no	Questions	Response	Comments
17.1	No of bed in female ward	☐ Yes ☐ No	
17.2	Does the facility have waiting room for attendants?	☐ Yes ☐ No	
17.3	Does the health facility have separate beds for admitting newborns?	☐ Yes ☐ No	
17.4	How many beds are available for newborns?		
(i)	**Electricity**		
17.5	Does the facilities have 24 x 7 initerrupted electricity supply?	☐ Yes ☐ No	
17.6	Does the facilities have backup source for the uninterrupted supply of electrical power?	☐ Yes ☐ No	
17.7	Did you have uninterupted power supply during the last one month?	☐ Yes ☐ No	
17.8	If NO , how many days?	_______Days	
(ii)	**Water supply**		
17.9	Does this facility have 24 x 7 water supply for functions ?	☐ Yes ☐ No	
17.10	Did you have uninterupted water supply during the last one month?	☐ Yes ☐ No	
(iii)	**Cleanliness**		
17.11	Are functional clean toilets available for patients?	☐ Yes ☐ No	
17.12	Are the toilets for health care providers available at wards?	☐ Yes ☐ No	
17.13	Is the overall cleaniness of the ward good?	☐ Yes ☐ No	
17.14	Does the facility has appropriate and functional hand washing facility in the patient care area?	☐ Yes ☐ No	
(iv)	**Services**		
17.15	The most seriously ill infants are cared for in a section near the nursing station for direct observation	☐ Yes ☐ No	
17.16	Is there emergency management area near ward	☐ Yes ☐ No	
17.17	Is there a heat source in the ward	☐ Yes ☐ No	
17.18	Minimum 48 hours of stay after delivery is followed for all deliveries is recommended	☐ Yes ☐ No	
17.19	Sick new-bornsare kept in a separate unit or room	☐ Yes ☐ No	
17.20	Mothers of sick new-bornsare rooming in with their babies.	☐ Yes ☐ No	

18. Staffing- Maternal ward

(i) Availability of staff (maternal ward)

SL No	Type of staff	Sanctioned number	In-position number	Number During any shift on weekdays			On holiday	Any deficiency in any area (OT/IL.R/Ward /OPD
				M	E	N		
**18.1**	Doctor (Obstetrician)							
**18.2**	Medical officer							
**18.3**	Staff nurse							
**18.4**	ANM							
**18.5**	Nursing assistant							
**18.6**	Multi-skilled Group D worker							
**18.7**	Sanitary worker							
**18.8**	Sweeper							
**18.9**	Aya							
**18.10**	Watchman							
**18.11**								
**18.12**								
**18.13**								
**18.14**								

S.No	Specific details of staff					Maternity Ward						
							Training					
18.15	Staff	a. Posted since (MM/YY)	b.Full time avaiable in facility	c. Residential	d.If no, Distance from residence	e.SBA/ BEmonc	f. BEmONC	g.NSSK	h.MTP	i.RTI/STI	j.IUCD	k. Any other Training
18.15.1	Designation<br>Name	☐☐☐☐	YES ☐<br>NO ☐	YES ☐<br>NO ☐	__.__ KMs	YES ☐<br>NO ☐	YES ☐<br>NO ☐	YES ☐<br>NO ☐	YES ☐<br>NO ☐	YES ☐<br>NO ☐	YES ☐<br>NO ☐	
18.15.2	Designation<br>Name	☐☐☐☐	YES ☐<br>NO ☐	YES ☐<br>NO ☐	__.__ KMs	YES ☐<br>NO ☐	YES ☐<br>NO ☐	YES ☐<br>NO ☐	YES ☐<br>NO ☐	YES ☐<br>NO ☐	YES ☐<br>NO ☐	
18.15.3	Designation<br>Name	☐☐☐☐	YES ☐<br>NO ☐	YES ☐<br>NO ☐	__.__ KMs	YES ☐<br>NO ☐	YES ☐<br>NO ☐	YES ☐<br>NO ☐	YES ☐<br>NO ☐	YES ☐<br>NO ☐	YES ☐<br>NO ☐	
18.15.4	Designation<br>Name	☐☐☐☐	YES ☐<br>NO ☐	YES ☐<br>NO ☐	__.__ KMs	YES ☐<br>NO ☐	YES ☐<br>NO ☐	YES ☐<br>NO ☐	YES ☐<br>NO ☐	YES ☐<br>NO ☐	YES ☐<br>NO ☐	
18.15.5	Designation<br>Name	☐☐☐☐	YES ☐<br>NO ☐	YES ☐<br>NO ☐	__.__ KMs	YES ☐<br>NO ☐	YES ☐<br>NO ☐	YES ☐<br>NO ☐	YES ☐<br>NO ☐	YES ☐<br>NO ☐	YES ☐<br>NO ☐	
18.15.6	Designation<br>Name	☐☐☐☐	YES ☐<br>NO ☐	YES ☐<br>NO ☐	__.__ KMs	YES ☐<br>NO ☐	YES ☐<br>NO ☐	YES ☐<br>NO ☐	YES ☐<br>NO ☐	YES ☐<br>NO ☐	YES ☐<br>NO ☐	
18.15.7	Designation<br>Name	☐☐☐☐	YES ☐<br>NO ☐	YES ☐<br>NO ☐	__.__ KMs	YES ☐<br>NO ☐	YES ☐<br>NO ☐	YES ☐<br>NO ☐	YES ☐<br>NO ☐	YES ☐<br>NO ☐	YES ☐<br>NO ☐	
18.15.8	Designation<br>Name	☐☐☐☐	YES ☐<br>NO ☐	YES ☐<br>NO ☐	__.__ KMs	YES ☐<br>NO ☐	YES ☐<br>NO ☐	YES ☐<br>NO ☐	YES ☐<br>NO ☐	YES ☐<br>NO ☐	YES ☐<br>NO ☐	

19. Paediatric ward

Infrastructure- Paediatric Ward

S. No.	Question	Response	Comments
**19.1**	How many beds does the ward have?	☐ Yes ☐ No	
**19.2**	How many patients are currently admitted	☐ Yes ☐ No	
**19.3**	Which age group admitted to the Pediatric ward?		
**19.4**	Are beds safe and well maintained?	☐ Yes ☐ No	
**19.5**	Are mattresses present on the beds	☐ Yes ☐ No	
**19.6**	Do patients receive clean bed linen?	☐ Yes ☐ No	
**19.7**	Are beds clean?	☐ Yes ☐ No	
**19.8**	Is there an emergency management area near the ward	☐ Yes ☐ No	
**19.9**	Is there any temperature regulatory mechanism present in the Pediatric ward	☐ Yes ☐ No	
**19.10**	Are there installed with mesh windows available for use of patients	☐ Yes ☐ No	
**(i)**	**Cleanliness**		
**19.11**	There are sufficient and adequate toilets which are easily accessible near the Pediatric ward	☐ Yes ☐ No	
**19.12**	Mothers have access to running water and to an appropriate space, near the ward, to wash themselves and their child.	☐ Yes ☐ No	
**19.13**	Mothers have access to a washing facility, in order to wash her and her child's clothes.	☐ Yes ☐ No	
**19.14**	Staff has access to hand washing facilities The ward is kept clean and dangerous items are inaccessible for children	☐ Yes ☐ No	

**(ii)**	**Standards**		
**19.15**	Children are kept in a separate ward or separate area of a ward.	☐ Yes ☐ No	
**19.16**	Severely ill children are kept apart from adults in wards such as for infectious diseases or intensive care.	☐ Yes ☐ No	
**19.17**	Children with surgical conditions are atleastkept in a separate room, with staff aware of the special needs for children such as feeding and warmth	☐ Yes ☐ No	
**19.18**	Arrangements are made to meet these needs.	☐ Yes ☐ No	
**19.19**	In cold climates, the ward has an efficient and safe heat source	☐ Yes ☐ No	
**19.20**	Mothers of sick newborns are rooming in with their babies, and have adequate facilities.	☐ Yes ☐ No	

20. Staffing – Paediatric Ward

(i) Availability of staff (Newborn ward)

SL No	Type of staff	Sanctioned number	In-position number	Number During any shift on weekdays			On holiday	Any deficiency in any area (OT/IL.R/Ward/OPD (Yes/No)
				M	E	N		
**20.1**	Medical officer							
**20.2**	Doctor (paediatrician)							
**20.3**	Staff nurse							
**20.4**	ANM							
**20.5**	Nurse Assistant							
**20.6**	Multi-skilled Group D worker							
**20.7**	Sanitary worker							
**21.8**	Sweeper							
**22.9**	Watchman							
**20.10**								

S.No	Specific details of staff (Pediatric Ward)										
						Training					
20.11	Staff	a. Posted since (MM/YY)	b.Full time avaiable in facility	c. Residential	d.If no, Distance from residence	e.IMNCI	f.NSSK	g.HBNC	h.PSBI	i.RBSK	j. Any other Training
20.11.1	Designation<br>Name		YES ☐<br>NO ☐	YES ☐<br>NO ☐	__.__KMs	YES ☐<br>NO ☐	YES ☐<br>NO ☐	YES ☐<br>NO ☐	YES ☐<br>NO ☐	YES ☐<br>NO ☐	
20.11.2	Designation<br>Name		YES ☐<br>NO ☐	YES ☐<br>NO ☐	__.__KMs	YES ☐<br>NO ☐	YES ☐<br>NO ☐	YES ☐<br>NO ☐	YES ☐<br>NO ☐	YES ☐<br>NO ☐	
20.11.3	Designation<br>Name		YES ☐<br>NO ☐	YES ☐<br>NO ☐	__.__KMs	YES ☐<br>NO ☐	YES ☐<br>NO ☐	YES ☐<br>NO ☐	YES ☐<br>NO ☐	YES ☐<br>NO ☐	
20.11.4	Designation<br>Name		YES ☐<br>NO ☐	YES ☐<br>NO ☐	__.__ KMs	YES ☐<br>NO ☐	YES ☐<br>NO ☐	YES ☐<br>NO ☐	YES ☐<br>NO ☐	YES ☐<br>NO ☐	
20.11.5	Designation<br>Name		YES ☐<br>NO ☐	YES ☐<br>NO ☐	__.__ KMs	YES ☐<br>NO ☐	YES ☐<br>NO ☐	YES ☐<br>NO ☐	YES ☐<br>NO ☐	YES ☐<br>NO ☐	
20.11.6	Designation<br>Name		YES ☐<br>NO ☐	YES ☐<br>NO ☐	__.__ KMs	YES ☐<br>NO ☐	YES ☐<br>NO ☐	YES ☐<br>NO ☐	YES ☐<br>NO ☐	YES ☐<br>NO ☐	
20.11.7	Designation<br>Name		YES ☐<br>NO ☐	YES ☐<br>NO ☐	__.__ KMs	YES ☐<br>NO ☐	YES ☐<br>NO ☐	YES ☐<br>NO ☐	YES ☐<br>NO ☐	YES ☐<br>NO ☐	
20.11.8	Designation<br>Name		YES ☐<br>NO ☐	YES ☐<br>NO ☐	__.__ KMs	YES ☐<br>NO ☐	YES ☐<br>NO ☐	YES ☐<br>NO ☐	YES ☐<br>NO ☐	YES ☐<br>NO ☐	

Summary Score – WARD					
a. Total responses with YES/NO options ☐☐		b. Total Yes ☐☐		c. Score ☐☐.☐☐	
d.Scale (Circle)	**5** (95-100%)	**4** (75-94%)	**3** (51-75%)	**2** (26-50%)	**1** **(< 25%)**

Section-E: NBSU

21. Standards- NBSU
General Infrastructure

S. No	Standards and Criterion	Response	Comments
21.1	Availability of functional NBSU	☐ Yes ☐ No	
21.2	Pediatrician/ MO trained in NBSU and adequate Staff Nurses	☐ Yes ☐ No	
21.3	SNCU with 12 beds 12-bed unit (plus 4 beds for step-down area)	☐ Yes ☐ No	
21.4	Provision of Kangaroo mother care is available	☐ Yes ☐ No	
21.5	24 hours services are provided	☐ Yes ☐ No	
21.6	Management of low birth weight infants <1800 gm and preterm done	☐ Yes ☐ No	
21.7	Management of sick newborns done	☐ Yes ☐ No	
21.8	Resuscitation of asphyxiated newborns done	☐ Yes ☐ No	
21.9	Prevention of infection, management of newborn sepsis	☐ Yes ☐ No	
21.10	Phototherapy for new born given	☐ Yes ☐ No	
21.11	Screening of New born for birth defects done	☐ Yes ☐ No	

22. Case management of sick new-born

S. No	Standards and Criterion	Response	Comments
**(i)**	**Appropriate diagnosis and treatment of neonatal sepsis**		
22.1	Neonatal sepsis cases are admitted and treated	☐ Yes ☐ No	
22.2	Blood sugar checked in all sick neonates	☐ Yes ☐ No	
22.3	Sepsis screen (TLC, DLC, CRP) done in suspected sepsis	☐ Yes ☐ No	
22.4	Appropriate antibiotics given as per guidelines	☐ Yes ☐ No	
22.5	The response to treatment is monitored	☐ Yes ☐ No	
**(ii)**	**Specific feeding needs of sick young infants and those with low birth weight**		
22.6	LBW babies are given breast milk	☐ Yes ☐ No	
22.7	Blood sugar checked periodically	☐ Yes ☐ No	
22.8	Frequent feedings (atleast 8 times per day) are provided to LBW- babies and intake is monitored	☐ Yes ☐ No	
22.9	If neonate is unable to feed expressed breast milk is given by cup and spoon or fed by orogastric tube	☐ Yes ☐ No	
22.10	In LBW-babies, heat loss is minimized by kangaroo mother care	☐ Yes ☐ No	
22.11	If IV-fluids are given, they are recorded and monitored	☐ Yes ☐ No	
**(iii)**	**Recognition and management of jaundice**		
22.12	Serum bilirubin estimations are done	☐ Yes ☐ No	

22.13	Phototherapy and guidelines when to use it are available	☐ Yes ☐ No	
22.14	There are guidelines when to recommend a baby who requires exchange transfusion	☐ Yes ☐ No	
22.15	Are mothers and/or family members allowed inside the NBSU?	☐ Yes ☐ No	
22.16	Are the mothers/family members encouraged to participate in general care of the newborn?	☐ Yes ☐ No	
22.17	Are the mothers/family members informed about the status of the newborn atleast daily?	☐ Yes ☐ No	

1. **Staffing- NBSU**

(i) **Availability of staff (NBSU)**

S. No	Type of staff	Sanctioned number	In-position number	Number During any shift on weekdays			On holiday	Any deficiency in any area (OT/IL.R/Ward /OPD (Yes/No)
				M	E	N		
23.1	Medical officer							
23.2	Doctor (paediatrician)							
23.3	Staff nurse							
23.4	ANM							
23.5	CC & Vaccine Logistic Assistant							
23.6	Multi-skilled Group D worker							
23.7	Sanitary worker							
23.8	Watchman							
23.9								
23.10								

S.No	Specific details of staff (NBSU)										
23.11	Staff	a. Posted since (MM/YY)	b.Full time avaiable in facility	c. Residential	d.If no, Distance from residence	Training					
						e.IMNCI	f.NSSK	g.HBNC	h.PSBI	i.RBSK	j. Any other Training
23.11.1	Designation<br>Name	☐☐☐☐	YES ☐<br>NO ☐	YES ☐<br>NO ☐	__.__ KMs	YES ☐<br>NO ☐	YES ☐<br>NO ☐	YES ☐<br>NO ☐	YES ☐<br>NO ☐	YES ☐<br>NO ☐	
23.11.2	Designation<br>Name	☐☐☐☐	YES ☐<br>NO ☐	YES ☐<br>NO ☐	__.__ KMs	YES ☐<br>NO ☐	YES ☐<br>NO ☐	YES ☐<br>NO ☐	YES ☐<br>NO ☐	YES ☐<br>NO ☐	
23.11.3	Designation<br>Name	☐☐☐☐	YES ☐<br>NO ☐	YES ☐<br>NO ☐	__.__ KMs	YES ☐<br>NO ☐	YES ☐<br>NO ☐	YES ☐<br>NO ☐	YES ☐<br>NO ☐	YES ☐<br>NO ☐	
23.11.4	Designation<br>Name	☐☐☐☐	YES ☐<br>NO ☐	YES ☐<br>NO ☐	__.__ KMs	YES ☐<br>NO ☐	YES ☐<br>NO ☐	YES ☐<br>NO ☐	YES ☐<br>NO ☐	YES ☐<br>NO ☐	
23.11.5	Designation<br>Name	☐☐☐☐	YES ☐<br>NO ☐	YES ☐<br>NO ☐	__.__ KMs	YES ☐<br>NO ☐	YES ☐<br>NO ☐	YES ☐<br>NO ☐	YES ☐<br>NO ☐	YES ☐<br>NO ☐	
23.11.6	Designation<br>Name	☐☐☐☐	YES ☐<br>NO ☐	YES ☐<br>NO ☐	__.__ KMs	YES ☐<br>NO ☐	YES ☐<br>NO ☐	YES ☐<br>NO ☐	YES ☐<br>NO ☐	YES ☐<br>NO ☐	
23.11.7	Designation<br>Name	☐☐☐☐	YES ☐<br>NO ☐	YES ☐<br>NO ☐	__.__ KMs	YES ☐<br>NO ☐	YES ☐<br>NO ☐	YES ☐<br>NO ☐	YES ☐<br>NO ☐	YES ☐<br>NO ☐	
23.11.8	Designation<br>Name	☐☐☐☐	YES ☐<br>NO ☐	YES ☐<br>NO ☐	__.__ KMs	YES ☐<br>NO ☐	YES ☐<br>NO ☐	YES ☐<br>NO ☐	YES ☐<br>NO ☐	YES ☐<br>NO ☐	

2. **Equipments- NBSU**

S. No	Equipment/ Supplies	Availability	Number	Functionality (Number)	Comments
**24.1**	Phototherapy Unit	☐ Yes ☐ No			
**24.2**	Emergency Resuscitation Kit-Baby	☐ Yes ☐ No			
**24.3**	Oxygen Concentrator	☐ Yes ☐ No			
**24.4**	Radiant Warmer	☐ Yes ☐ No			
**24.5**	Room Warmer	☐ Yes ☐ No			
**24.6**	Pulse Oxymeter	☐ Yes ☐ No			
**24.7**	Nebulizer	☐ Yes ☐ No			
**24.8**	Weighing machine infant	☐ Yes ☐ No			
**24.9**	CPAP Machine	☐ Yes ☐ No			
**24.10**	Stethoscope	☐ Yes ☐ No			
**24.11**	Suction Machine	☐ Yes ☐ No			
**24.12**	Hub cutters	☐ Yes ☐ No			
**24.13**	Infantometer	☐ Yes ☐ No			
**24.14**	Light examination, mobile	☐ Yes ☐ No			
**24.15**	Thermometers	☐ Yes ☐ No			
**24.16**	Pediatric Laryngoscope set & cells	☐ Yes ☐ No			
**24.17**	Flow meters and humidifiers for oxygen	☐ Yes ☐ No			
**24.18**	Self-inflating bags (0250 ml, 500 ml	☐ Yes ☐ No			
**24.19**	Glucometer	☐ Yes ☐ No			
**24.20**	Lamp, ultra- violet	☐ Yes ☐ No			
**24.21**	Fetal Doppler	☐ Yes ☐ No			
**24.22**	Oxygen Catheter 8 F, Oxygen cylinder	☐ Yes ☐ No			
**24.23**	Nasal Prongs	☐ Yes ☐ No			
**24.24**	Nasal Catheters	☐ Yes ☐ No			
**24.25**	Infant size mask	☐ Yes ☐ No			
**24.26**	NG tubes (8,10, 12FG)	☐ Yes ☐ No			

**24.27**	IV sets with chambers	☐ Yes ☐ No			
**24.28**	Mucus extractor 20 ml	☐ Yes ☐ No			
**24.29**	IV cannulas(24G, 26G)	☐ Yes ☐ No			
**24.30**	Glucostix/ multistix	☐ Yes ☐ No			
**24.31**	Feeding tube, CH07, L40 cm	☐ Yes ☐ No			
**24.32**	Sterile gloves	☐ Yes ☐ No			
**24.33**	Disinfectant, 20% Chlorhexidine	☐ Yes ☐ No			

**Summary Score –NBSU**					
a. Total responses with YES/NO options ☐☐			b. Total Yes ☐☐	c. Score ☐☐.☐☐	
d.Scale (Circle)	**5** **(**95-100%)	**4** **(**75-94%)	**3** **(**51-75%)	**2** **(**26-50%)	**1** **(< 25%)**

SECTION F: -OUT PATIENT DEPARTMENT

3. Infrastructure – Out Patient Department

S.No	Question	Response	Comment
**(i)**	**Paediatric OPD**		
**25.1**	Is the facility runs a separate pediatric outpatient department?	☐ Yes ☐ No	
**25.2**	At what time does the outpatient department open and close?	Mon- Fri ☐☐ : ☐☐ to ☐☐ : ☐☐<br>Sat ☐☐ : ☐☐ to ☐☐ : ☐☐<br>Sun & Holidays ☐☐ : ☐☐ to ☐☐ : ☐☐	
**25.3**	Does the health facility have a separate emergency area for newborn services?	☐ Yes ☐ No	
**25.4**	Is it open 24 hours	☐ Yes ☐ No	
**25.5**	If not then for how many hours a day does it open	________Hrs	
**25.6**	MO provides the first contact care to the pediatric patient?	☐ Yes ☐ No	
**25.7**	Has the first contact care staff received training in resuscitation/FBNC/NSSK/ F-IMNCI	☐ Yes ☐ No	
**(ii)**	**OBS & GYNAE OPD**		
**25.8**	Is the obstetrical outpatient separate from the Adult outpatient department?	☐ Yes ☐ No	
**25.9**	At what time does the outpatient department open and close?	Mon- Fri ☐☐ : ☐☐ to ☐☐ : ☐☐<br>Sat ☐☐ : ☐☐ to ☐☐ : ☐☐<br>Sun & Holidays ☐☐ : ☐☐ to ☐☐ : ☐☐	
**25.10**	MO provides the first contact care to the patient?	☐ Yes ☐ No	
**25.11**	Has the first contact care staff received training in SBA/BEmOnc/High risk pregnancy care)	☐ Yes ☐ No	
**25.12**	Suggestion/ Complaint Box avaiable in the OPD	☐ Yes ☐ No	
**25.13**	Does this OPD have separate drug dispensing unit.	☐ Yes ☐ No	
**25.14**	Does the facility have registration counter	☐ Yes ☐ No	
**(iii)**	**Cleanliness**		
**25.15**	Separate functional Public Utilities for Male and Female ?	☐ Yes ☐ No	
**25.16**	Is the overall cleaniness of the toilets good?	☐ Yes ☐ No	
**25.17**	Is the overall cleaniness of the OPD room good?	☐ Yes ☐ No	

**25.18**	Is the overall cleaniness of the OPDs good?(Including the waiting area)	☐ Yes ☐ No	
**25.19**	Does the facility has appropriate and functional hand washing facility in the patient care area?	☐ Yes ☐ No	
**25.20**	Does the OPD have running water facility	☐ Yes ☐ No	
**25.21**	Does the water supply in OPD is supported with overhead water tank and pump?	☐ Yes ☐ No	
**(iV)**	**Waste management**		
**25.22**	Do you have system for segregation of waste?	☐ Yes ☐ No	
**25.23**	Are there color coded bins for segregation of waste?	☐ Yes ☐ No	
**25.24**	Is there display of instructions for management of BMW	☐ Yes ☐ No	

4. Staffing at OPD

Availability of staff(OPD)

S. No	Type of staff	Sanctioned number	In-position number	Number During any shift on weekdays			On holiday	Any deficiency in any area (OT/IL.R/Ward/ OPD (Yes/No)
				M	E	N		
**26.1**	Doctor (obstetrician)							
**26.2**	Doctor (paediatrician)							
**26.3**	Staff nurse							
**26.4**	ANM							
**26.5**	Nursing Assisstant							
**26.6**	Multi-skilled Group D worker							
**26.7**	Sanitary worker							
**26.8**								
**26.9**								
**26.10**								

S.No	Specific details of staff (OPD)														
26.11	Staff	a. Posted since (MM/YY)	b.Full time avaiable in facility	c. Residential	d.If no, Distance from residence	Training									
						Mother				Newborn					
						e.SBA BEmOC	f.MTP	g.RTI/STI	h.IUCD	i.IMNCI	j. NSSK	k. HBNC	L.PSBI	M. RBSK	N. Any other training
26.11.1	Designation<br>Name	☐☐ ☐☐	YES ☐<br>NO ☐	YES ☐<br>NO ☐	__.__ KMs	YES ☐<br>NO ☐	YES ☐<br>NO ☐	YES ☐<br>NO ☐	YES ☐<br>NO ☐	YES ☐<br>NO ☐	YES ☐<br>NO ☐	YES ☐<br>NO ☐	YES ☐<br>NO ☐	YES ☐<br>NO ☐	
26.11.2	Designation<br>Name	☐☐ ☐☐	YES ☐<br>NO ☐	YES ☐<br>NO ☐	__.__ KMs	YES ☐<br>NO ☐	YES ☐<br>NO ☐	YES ☐<br>NO ☐	YES ☐<br>NO ☐	YES ☐<br>NO ☐	YES ☐<br>NO ☐	YES ☐<br>NO ☐	YES ☐<br>NO ☐	YES ☐<br>NO ☐	
26.11.3	Designation<br>Name	☐☐ ☐☐	YES ☐<br>NO ☐	YES ☐<br>NO ☐	__.__ KMs	YES ☐<br>NO ☐	YES ☐<br>NO ☐	YES ☐<br>NO ☐	YES ☐<br>NO ☐	YES ☐<br>NO ☐	YES ☐<br>NO ☐	YES ☐<br>NO ☐	YES ☐<br>NO ☐	YES ☐<br>NO ☐	
26.11.4	Designation<br>Name	☐☐ ☐☐	YES ☐<br>NO ☐	YES ☐<br>NO ☐	__.__ KMs	YES ☐<br>NO ☐	YES ☐<br>NO ☐	YES ☐<br>NO ☐	YES ☐<br>NO ☐	YES ☐<br>NO ☐	YES ☐<br>NO ☐	YES ☐<br>NO ☐	YES ☐<br>NO ☐	YES ☐<br>NO ☐	
26.11.5	Designation<br>Name	☐☐ ☐☐	YES ☐<br>NO ☐	YES ☐<br>NO ☐	__.__ KMs	YES ☐<br>NO ☐	YES ☐<br>NO ☐	YES ☐<br>NO ☐	YES ☐<br>NO ☐	YES ☐<br>NO ☐	YES ☐<br>NO ☐	YES ☐<br>NO ☐	YES ☐<br>NO ☐	YES ☐<br>NO ☐	
26.11.6	Designation<br>Name	☐☐ ☐☐	YES ☐<br>NO ☐	YES ☐<br>NO ☐	__.__ KMs	YES ☐<br>NO ☐	YES ☐<br>NO ☐	YES ☐<br>NO ☐	YES ☐<br>NO ☐	YES ☐<br>NO ☐	YES ☐<br>NO ☐	YES ☐<br>NO ☐	YES ☐<br>NO ☐	YES ☐<br>NO ☐	
26.11.7	Designation<br>Name	☐☐ ☐☐	YES ☐<br>NO ☐	YES ☐<br>NO ☐	__.__ KMs	YES ☐<br>NO ☐	YES ☐<br>NO ☐	YES ☐<br>NO ☐	YES ☐<br>NO ☐	YES ☐<br>NO ☐	YES ☐<br>NO ☐	YES ☐<br>NO ☐	YES ☐<br>NO ☐	YES ☐<br>NO ☐	
26.11.8	Designation<br>Name	☐☐ ☐☐	YES ☐<br>NO ☐	YES ☐<br>NO ☐	__.__ KMs	YES ☐<br>NO ☐	YES ☐<br>NO ☐	YES ☐<br>NO ☐	YES ☐<br>NO ☐	YES ☐<br>NO ☐	YES ☐<br>NO ☐	YES ☐<br>NO ☐	YES ☐<br>NO ☐	YES ☐<br>NO ☐	

Summary Score –Out-patient department					
b. Total responses with YES/NO options	☐☐	b. Total Yes	☐☐	c.Score	☐☐ ☐☐
d.Scale (Circle)	**5** (95-100%)	**4** (75-94%)	**3** (51-75%)	**2** (26-50%)	**1** **(< 25%)**

Section-G: Laboratory services

5. **Laboratory Tests**

S. No.	Name of Test	Availability	Available 24X7	Time to get results	Comments
**27.1**	Blood Glucose	☐ Yes ☐ No	☐ Yes ☐ No		
**27.2**	Glucose Tolerance Test	☐ Yes ☐ No	☐ Yes ☐ No		
**27.3**	Hemoglobin	☐ Yes ☐ No	☐ Yes ☐ No		
**27.4**	Peripheral blood smear	☐ Yes ☐ No	☐ Yes ☐ No		
**27.5**	Serum bilirubin	☐ Yes ☐ No	☐ Yes ☐ No		
**27.6**	Blood grouping, Cross matching, Rh typing	☐ Yes ☐ No	☐ Yes ☐ No		
**27.7**	Urine analysis for sugar, albumin	☐ Yes ☐ No	☐ Yes ☐ No		
**27.8**	VDRL	☐ Yes ☐ No	☐ Yes ☐ No		
**27.8**	Blood urea, cholesterol	☐ Yes ☐ No	☐ Yes ☐ No		
**27.9**	Elisa test for HIV, HBsAg, HCV, malaria	☐ Yes ☐ No	☐ Yes ☐ No		

28. Laboratory standards

S. No.	Standards and Criterion	Response	Comments
**28.1**	Essential lab tests (blood glucose, hemoglobin, hematocrit) are available all the time and their results delivered in a timely fashion to the ward emergency area.	☐ Yes ☐ No	
**28.2**	Tests are available free of cost to patients	☐ Yes ☐ No	
**28.3**	Tests for emergency cases are given priority	☐ Yes ☐ No	
**28.4**	The laboratory is situated such that it has easy access to IPD as well as OPD patients.	☐ Yes ☐ No	
**28.5**	There is a separate and demarcated areas for sample collection	☐ Yes ☐ No	
**28.6**	There is a separate area for sample processing	☐ Yes ☐ No	
**28.7**	There is a separate and demarcated areas for report distribution/ collection.	☐ Yes ☐ No	

**Summary Score –Laboratory**					
a. Total responses with YES/NO options ☐☐			b. Total Yes ☐☐	c. Score ☐☐ ☐☐	
d. Scale (Circle)	**5** (95-100%)	**4** (75-94%)	**3** (51-75%)	**2** (26-50%)	**1** (< 25%)

Section-H: Pharmacy

29. Drugs

S. No.	Drugs	Availability	Comment	S. No	Drugs	Availability	Comment
**Maternal Care**							
29.1	Cap Amoxycillin	☐ Yes ☐ No		29.20	InjThiopentone	☐ Yes ☐ No	
29.2	Tab Metronidazole	☐ Yes ☐ No		29.21	Inj Bupivacaine	☐ Yes ☐ No	
29.3	Tab Misprostol	☐ Yes ☐ No		29.22	Inj Ampicillin	☐ Yes ☐ No	
29.4	Tab Nifedipine	☐ Yes ☐ No		29.23	Inj Gentamycin	☐ Yes ☐ No	
29.5	Tab Ibuprofen	☐ Yes ☐ No		29.24	InjDexmethasone	☐ Yes ☐ No	
29.6	Tab Norfloxacin	☐ Yes ☐ No		29.25	Inj Hydrocortisone	☐ Yes ☐ No	
29.7	Tab Fluconazole	☐ Yes ☐ No		29.26	Inj Paracetamol	☐ Yes ☐ No	
29.8	Tab Paracetamol	☐ Yes ☐ No		29.27	Inj Magnesium sulphate	☐ Yes ☐ No	
29.9	Tab Methyl Dopa	☐ Yes ☐ No		29.28	Inj Oxytocin	☐ Yes ☐ No	
29.10	Tab Labetolol	☐ Yes ☐ No		29.29	Inj Insulin	☐ Yes ☐ No	
29.11	Tab. MedroxyProgestrone Acetate	☐ Yes ☐ No		29.30	Inj Aminophylline	☐ Yes ☐ No	
29.12	Tab. Clotrimazole (Vag)	☐ Yes ☐ No		29.31	InjHydroxyProjesterone	☐ Yes ☐ No	
29.13	Tab Clotrimazole + Clindamicin (Vag)	☐ Yes ☐ No		29.32	Inj Methyl Ergometrine	☐ Yes ☐ No	
29.14	Inj Xylocaine	☐ Yes ☐ No		29.33	Inj. Dilantin Sodium	☐ Yes ☐ No	
29.15	Inj Betamethasone	☐ Yes ☐ No		29.34	Inj diazepam	☐ Yes ☐ No	
29.16	Inj Hydralazine	☐ Yes ☐ No		29.35	injPheniramine maleate	☐ Yes ☐ No	
29.17	Inj Atropine	☐ Yes ☐ No		29.36	InjCorboprost	☐ Yes ☐ No	
29.18	Tab Doxycycline	☐ Yes ☐ No		29.37	Tab Iron FA	☐ Yes ☐ No	
29.19	Inj Lignocaine	☐ Yes ☐ No					
**Newborn care**							
29.38	Pediatric Maintenance Fluid (Isolyte P)	☐ Yes ☐ No		29.53	Combined mineral and vitamins	☐ Yes ☐ No	
29.39	SypAmoxycillin-Clavulanic	☐ Yes ☐ No		29.54	Amoxycillin-Clavulanic Suspension	☐ Yes ☐ No	
29.40	Glucose 10% IV	☐ Yes ☐ No		29.55	InjVit K (IM)	☐ Yes ☐ No	
29.41	Glucose 20/25% IV	☐ Yes ☐ No		29.56	Inj Adrenaline	☐ Yes ☐ No	
29.42	Normal Saline	☐ Yes ☐ No		29.57	BCG Vaccine	☐ Yes ☐ No	

**29.43**	Inj Phenobarbital	☐ Yes ☐ No		**29.58**	Hep B vaccine	☐ Yes ☐ No	
**29.44**	Ampicillin/Amoxycillin	☐ Yes ☐ No		**29.59**	OPV Vaccine	☐ Yes ☐ No	
**29.45**	Inj Benzyl Penicillin	☐ Yes ☐ No		**29.60**	Inj Calcium gluconate	☐ Yes ☐ No	
**29.46**	InjCloxacillin	☐ Yes ☐ No		**29.61**	Tab Oral Zinc	☐ Yes ☐ No	
**29.47**	Inj Gentamycin	☐ Yes ☐ No		**29.62**	ORS	☐ Yes ☐ No	
**29.48**	Inj Amikacin	☐ Yes ☐ No		**29.63**	Inj. Cefotaxime	☐ Yes ☐ No	
**29.49**	Inj Dopamine/ Dobutamine	☐ Yes ☐ No		**29.64**	Ringer Lactate IVF	☐ Yes ☐ No	
**29.50**	Syp. Iron/FA	☐ Yes ☐ No		**29.65**	Tab Ciprofloxacin	☐ Yes ☐ No	
**29.51**	Inj Lasix	☐ Yes ☐ No		**29.66**	Inj Naloxone	☐ Yes ☐ No	
**29.52**	Salbutamol nebusol solution	☐ Yes ☐ No					

30. Standards- Pharmacy

**Essential Drugs**			
**S. No**	**Question**	**Response**	**Comments**
**30.1**	Is there drug inventory register?	☐ Yes ☐ No	
**30.2**	Is the drug inventory register up-to-date?	☐ Yes ☐ No	
**30.3**	Is there any mechanism to ensure that expiry drugs are not distributed?	☐ Yes ☐ No	
**30.4**	An essential drug list exists in the hospital	☐ Yes ☐ No	
**30.5**	Are adequate quantity of drugs in essential drug list available	☐ Yes ☐ No	
**30.6**	Oldest drugs are used first	☐ Yes ☐ No	
**30.7**	The facility ensures that drugs which are available at Pharmacy and or wards are prescribed to patients as priority	☐ Yes ☐ No	
**30.8**	Antibiotic policy exists and revised as per sensitivity pattern	☐ Yes ☐ No	

Final facility assessment score

		Score	Rating	Improvement Scale (Circle one)
	**General**			Performance to be sustained(Rating 5)……………………………………………1<br>Needs improvement in some areas (Rating 3 and 4)……………….…………2<br>Improvement needed in many areas (Rating 1 or 2)…………………………3
	**Labour Room**			Performance to be sustained(Rating 5)……………………………………………1<br>Needs improvement in some areas (Rating 3 and 4)……………….…………2<br>Improvement needed in many areas (Rating 1 or 2)…………………………3
	**WARD**			Performance to be sustained(Rating 5)……………………………………………1<br>Needs improvement in some areas (Rating 3 and 4)……………….…………2<br>Improvement needed in many areas (Rating 1 or 2)…………………………3
	**NBSU**			Performance to be sustained(Rating 5)……………………………………………1<br>Needs improvement in some areas (Rating 3 and 4)……………….…………2<br>Improvement needed in many areas (Rating 1 or 2)…………………………3
	**OPD**			Performance to be sustained(Rating 5)……………………………………………1<br>Needs improvement in some areas (Rating 3 and 4)……………….…………2<br>Improvement needed in many areas (Rating 1 or 2)…………………………3
	**LABORATORY**			Performance to be sustained(Rating 5)……………………………………………1<br>Needs improvement in some areas (Rating 3 and 4)……………….…………2<br>Improvement needed in many areas (Rating 1 or 2)…………………………3
	**PHARMACY**			Performance to be sustained(Rating 5)……………………………………………1<br>Needs improvement in some areas (Rating 3 and 4)……………….…………2<br>Improvement needed in many areas (Rating 1 or 2)…………………………3

End of assessment tool

Main Strengths

……

……

Main Weaknesses

……

……

Suggestions for improvement

……

……

UNIQUE ID:

Improving Quality of Care for Mother and Newborn at District Hospitals and FRU's of 3 Districts of Haryana

PATIENT SATISFACTION QUESTIONNAIRE
WOMEN ATTENDING FOR ANTENATAL CARE

Name and Address of the facility: ____

____

Name and Address of the patient: ____

____

Consent given: ____

Date of Interview: ____

Start time: ____

End time: ____

Interviewed by: ____

Instructions:

- *Greet the patient and introduce yourself.*
- *Explain the patient why you are taking up this questionnaire.*
- *Take the consent and then start the questions.*
- *If you do not get satisfactory response, promptly please repeat the question and probe wherever necessary.*
- *If a patient does not agree to respond, say thanks to them for giving you the time.*
- *Please introduce each question separately and record the response.*
- *The elicited information will be treated as confidential and don't discuss or share the responses with any third/outside person.*
- ***The data will be recorded using the TABLET.***

		**Response**
**1**	**Personal Details**	
**1.1**	What is your age?	
**1.2**	What is your Education status?	
	**1-Illitrate 2- Primary school 3- High School 4- Graduate 5-Post graduate**	
**1.3**	What is your occupation?	
	**1-Housewife 2- Service 3- Business 4. Labor 5. others**	
**1.4**	How far away do you live from the hospital?	**_____ Km**
**1.5**	How long did it take to reach the hospital?	__ Hrs __ Min
**1.6**	**Pregnancy Information:**	
**1.6.1**	☐ Gravida ☐ Para ☐ Live birth ☐ Still b[illegible]h ☐ Ab[illegible]tion ☐ No of living children	
**1.6.2**	If G2 or more did you came for ANC visits here ? **1-Yes 0- No**	
**1.7**	Where did you deliver during your past pregnancies? *(Skip, if 1st pregnancy)*	
	**1-Home 2- This hospital 3- Any other hospital**	
**1.7.1**	Have you been here for earlier ANC checkup during this pregnancy? **1-Yes 0- No**	
**1.7.2**	What is the total number of times you came for ANC checkup?	
**2**	**General Details**	
**2.1**	Have you reached hospital after visiting multiple facilities? **1-Yes 0- No**	
**2.2**	Did you get a free transport to reach the facility (from home/ another health facility to this facility/ hospital)? **1-Yes 0- No**	
**2.3**	Did ANM/ ASHA arrange the transport? **1-Yes 0- No**	
**2.4**	Are you aware that pregnant women and mother of newborn are entitled for free drop back to home? **1-Yes 0- No**	
**2.5**	Did ASHA accompany you till facility? **1-Yes 0- No**	
**3**	**Experience at Admission**	
**3.1**	Were you able to locate the ANC OPD easily on your first visit **1-Yes 0- No**	
**3.2**	Are you satisfied with the care given in OPD? **1-Yes 0- No**	
**4**	**Attitude/ Behavior of the Staff**	
**4.1**	How was the attitude and behavior of the staff, at the reception, when you reached the facility?	
	**1- Helpful & Polite 2- Bad at times but acceptable 3- Rude, unhelpful, Not acceptable**	
**4.2**	Was the adequate information displayed at the reception/ registration counter?	
	**1- Yes 2- Somewhat adequate 3- Not at all clear**	
**4.3**	How prompt was the registration process?	
	**1- Very Prompt 2- Neither prompt nor delayed 3- Delayed**	
**4.4**	When you arrived at the hospital, how long did you have to wait before a nurse/doctor examined you?	
	**1- Immediately 2- Between 15-30 minutes 3- More than 30 minutes**	
**4.5**	Was a family member allowed to stay in the same room with you during the examination? **1-Yes 0- No**	
**4.6**	Did the health workers explain what they were doing and what would happen next? **1-Yes 0- No**	
**4.7**	Were you satisfied with the privacy and confidentiality that you got in the OPD? **1-Yes 0- No**	

1- Very Satisfied 2- Neither satisfied nor dissatisfied 3- Dissatisfied			
4.8	Did the nurse/doctor spent adequate time in examining/ discussing with you and your problem?	1-Yes 0- No	
4.9	Did the nurse/doctor explain you about care and precaution during pregnancy?	1-Yes 0- No	
4.10	Did the nurse/doctor explain about the preparation for delivery and where to deliver/when to come/ preparation etc?	1-Yes 0- No	
**5**	**Cleanliness/ Hygiene**		
5.1	How was the general cleanliness/ hygiene of the building, corridor and premises		
1- Very clean & hygienic 2- Not so clean but acceptable 3- Very poor & unhygienic			
5.2	How was the cleanliness of OPD Chamber/ Waiting area		
1- Very clean & hygienic 2- Not so clean but acceptable 3- Very poor & unhygienic			
5.3	Did you use the toilet during this visit?	1-Yes 0- No	
	**If yes, please comment about:**		
5.3.1	Availability of water	1-Yes 0- No 8. Don't Know	
5.3.2	Cleanliness of water	1-Yes 0- No 8.Don't know	
5.3.3	Hand washing facility in toilet	1-Yes 0- No 8.Don't know	
**6.**	**Basic Amenities**		
6.1	Please tell us about the light & fan availability:		
6.1.1	Light	1-Yes 0- No 8. Don't Know	
6.1.2	Fan/ Ventilation of the waiting area	1-Yes 0- No 8.Don't know	
6.1.3	Fan/ Ventilation of the OPD chamber	1-Yes 0- No 8.Don't know	
**7.**	**Out of Pocket Expenses**		
7.1	Did you make any unofficial/ informal payments?	1-Yes 0- No	
7.2	Did you pay for any medications/ any other out of pocket expenses? (Please specify)	1-Yes 0- No	
7.3	Did you pay for diagnostic facilities (Laboratory, Radiology, Ultrasound and specialized investigation etc)	1-Yes 0- No	
**8.**	**High Risk Case (PIH/GDM/BOH/Anemia)**		
8.1	Have you been informed about any high risk factor/problem identified during this pregnancy	1-Yes 0- No	
8.2	Do you know about the danger signs during pregnancy when you should come to hospital?	1-Yes 0- No	
**9**	**Quality of Service Delivery at the facility**		
9.1	Was the doctor's availability during day time adequate?	1-Yes 0- No 8.Don't know	
9.2	Was the nurse's availability during day time adequate?	1-Yes 0- No 8.Don't know	
9.3	When you had important question to ask a doctor/nurse, did you get answers that you could understand?		
1- Yes, always 2- I had no need to ask 3- No			
9.4	Did you understand what the doctor/nurse explained to you?		
1- Yes, very clear 2- Somewhat clear 3- Not at all clear			
9.5	How was the behavior of doctor and nurses with you?		
1- Helpful & Polite 2- Bad at times but acceptable 3- Rude, unhelpful, Not acceptable			

**9.6**	Overall, did you feel you were treated with respect and dignity while you were in hospital?	**1-Yes 0- No**	
**9.7**	Were you satisfied by the care and treatment given at the health facility?		
**1- Very Satisfied 2- somewhat satisfied 3- Dissatisfied**			
**9.8**	Would you recommend other family members to come to this hospital?	**1-Yes 0- No**	
**9.9**	Do you have any other comments or suggestions on how care can be improved for mothers and babies in this hospital?		

UNIQUE ID:

Improving Quality of Care for Mother and Newborn at District Hospitals and FRU's of 3 Districts of Haryana

PATIENT SATISFACTION QUESTIONNAIRE
RECENTLY DELIVERED WOMEN (POSTNATAL CARE)

Name and Address of the facility: ____

____

Name and Address of the patient: ____

____

Date of Hospitalization: ____________________ **Date of Discharge:** ____________________

Consent given: ____

Date of Interview: ____

Start time: ____

End time: ____

Interviewed by: ____

Instructions:

- *Greet the patient and introduce yourself.*
- *Explain the patient why you are taking up this questionnaire.*
- *Take the consent and then start the questions.*
- *If you do not get satisfactory response, promptly please repeat the question and probe wherever necessary.*
- *If a patient does not agree to respond, say thanks to them for giving you the time.*
- *Please introduce each question separately and record the response.*
- *The elicited information will be treated as confidential and don't discuss or share the responses with any third/outside person.*
- ***The data will be recorded using the TABLET.***

		Response
**1**	**Personal Details**	
**1.1**	What is your age? (For mother)<br>Relation with the patient? (For family member)	
**1.2**	What is your Education status?	
**1-Illitrate 2- Primary school 3- High School 4-Secondary School 5-Under graduate 6-Post graduate**		
**1.3**	What is your occupation?	
	**1-Housewife 2- Service 3- Business 4.Labor 5. Others**	
**1.4**	**Pregnancy Info:**	
**1.4.1**	☐ Gravida ☐ Para ☐ Live birth ☐ Still b[illegible]th ☐ Ab[illegible]tion ☐ No of living children	
**1.5**	If G2 /more, were your previous children born here?	**1-Yes 0- No**
**1.6**	Have you been here for any other illness?	**1-Yes 0- No**
**1.7**	Length of stay in hospital	**_______ Days**
**1.8**	How far away do you live from the hospital?	**___ Km**
**1.9**	How long did it take to reach the hospital?	__ Hrs __ Min
**2**	**General Details**	
**2.1**	Have you been referred to this facility- from other facility/Family or self/Private doctor?	**1-Yes 0- No**
**2.2**	Did you get a free transport to reach the facility (from home/ another health facility to this facility/ hospital)?	**1-Yes 0- No**
**2.3**	Have you reached hospital after visiting multiple facilities?	**1-Yes 0- No**
**2.4**	Did ANM/ ASHA arrange the transport?	**1-Yes 0- No**
**2.5**	Did ASHA accompany you till facility?	**1-Yes 0- No**
**2.6**	Did you attend antenatal care during your pregnancy?	**1-Yes 0- No**
**2.7**	If yes, where did you attend antenatal appointments?	
**3**	**Experience at admission**	
**3.1**	Did you/your patient get admitted through OPD?	**1-Yes 0- No**
**3.2**	If yes, was the person at registration/ in OPD was helpful to you?	**1-Yes 0- No**
**3.3**	Did your patient get admitted through Emergency?	**1-Yes 0- No**
**3.4**	If yes, was the person at registration/ in emergency helpful to you?	**1-Yes 0- No**
**3.5**	Was wheel chair/ stretcher available for you?	**1-Yes 0- No**
**3.6**	Were you able to locate the ward/ labour room easily (location & sign boards)?	**1-Yes 0- No**
**4**	**Attitude/ Behavior of the Staff**	
**4.1**	How was the attitude and behavior of the staff, at the reception, when you reached the facility?	
	**1- Helpful & Polite 2- Bad at times but acceptable 3- Rude, unhelpful, Not acceptable**	
**4.2**	Was the adequate information displayed at the reception/ registration counter?	
	**1- Yes 2- Somewhat adequate 3- Not at all clear**	
**4.3**	How prompt was the registration process?	
	**1- Very Prompt 2- Average 3- Delayed**	

5	Care in Labor		
5.1	How long did you have to wait before a health worker examined you, after your arrival at the hospital?		
**1- Immediately 2- Between 15-30 minutes 3- More than 30 minutes**			
5.2	Did the health workers explain what they were doing and what would happen next?	**1-Yes 0- No**	
5.3	Overall, did you feel comfortable in the environment where you were laboring?		
**1- Very comfortable 2- Somewhat comfortable 3- Very uncomfortable**			
5.4	During your labor, did the staff supported you ensuring privacy	**1-Yes 0- No**	
5.5	During your labor, did the staff verbally encouraged or reassured you?	**1-Yes 0- No**	
5.6	What was the mode of delivery?		
**1-Vaginally 2- Caesarean 3- Forceps/Vacuum**			
5.7	How was the attitude and behavior of the staff during labor and delivery?		
**1- Helpful & Polite 2- Bad at times but acceptable 3- Rude, unhelpful, Not acceptable**			
5.8	Were the family members allowed to stay in the same room with the patient during the initial examination?	**1-Yes 0- No**	
5.9	Were the family members allowed to stay in the room with you all the time during labor?	**1-Yes 0- No**	
5.10	Overall, are you happy with the support you received from the health professionals during labor and delivery?	**1-Yes 0- No**	
**6**	**Care after Delivery**		
6.1	Immediately after birth of the baby, did anybody tell you about the status of the baby and sex?	**1-Yes 0- No**	
6.2	Was your baby placed on your chest/ abdomen immediately after the birth?	**1-Yes 0- No**	
6.3	Did anyone (Nurse/ Other staff) explain/ support you in initiating & giving breastfeeding?	**1-Yes 0- No**	
6.4	Were you and your baby examined regularly (at least daily) during your stay at hospital?		
**1- Yes 2- Occasional Visits 3- Not at all regular**			
6.5	Who examined you and your baby mostly?		
6.6	Did any doctor visit you during your stay at hospital?	**1-Yes 0- No**	
6.7	Did health staff discuss contraception/ family planning with you during your stay?	**1-Yes 0- No**	
6.8	Now you are getting discharge, have you been explained about the care for yourself and baby to be taken at home?	**1-Yes 0- No**	
6.9	Do you know about the danger signs for the baby and yourself and when to come back/go to hospital?	**1-Yes 0- No**	
6.10	Have you received the discharge sheet explaining the medicines and follow-up?	**1-Yes 0- No**	
**7**	**Cleanliness/ Hygiene**		
7.1	How were the general cleanliness/ hygiene of the building, corridor and premises?		
**1- Very clean & hygienic 2- Not so clean but acceptable 3- Very poor & unhygienic**			
7.2	How was the cleanliness inside patient wards/rooms?		

1- Very clean & hygienic 2- Not so clean but acceptable 3- Very poor & unhygienic			
7.3	Did you get clean bed-sheets? How frequently did the hospital staff used to change bed-sheets?	1-Yes 0- No	
7.4	How was the cleanliness inside labor ward/room?		
1- Very clean & hygienic 2- Not so clean but acceptable 3- Very poor & unhygienic			
7.5	Is the running water and hand washing facilities available in toilets?	1-Yes 0- No	
**8**	**Privacy/ Basic Amenities**		
8.1	Were you satisfied with privacy and confidentiality that you got in the OPD/wards/labor room?		
1- Very Satisfied 2- Not satisfied but acceptable 3- Dissatisfied			
8.2	Is 24/7 safe and clean supply of drinking water available in the hospital?	1-Yes 0- No	
8.3	Whether free diet was provided during the stay in hospital?	1-Yes 0- No	
8.4	Was quality and quantity of food adequate and satisfactory (If food is provided by the health facility)?		
1- Adequate 2- Just appropriate 3- Not at all adequate			
8.5	Do you find the amount of space/ bed provided for the mother and baby to stay adequate?	1-Yes 0- No	
8.6	Do the availability of toilet/hand washing facility appropriate?	1-Yes 0- No 8. Don't Know	
8.7	Do you find the toilets well maintained and clean?	1-Yes 0- No 8.Don't Know	
8.8	Do you find the lights and fans in the ward functional?	1-Yes 0- No 8.Don't Know	
8.9	Is there provision of shelter and cafeteria for care takers/ attendants of patients?	1-Yes 0- No 8. Don't Know	
**9**	**Out of Pocket expenditure**		
9.1	Did you make any unofficial/ informal payments?	1-Yes 0- No	
9.2	Did you pay for any medications/ any other out of pocket expenses? (Please specify)	1-Yes 0- No	
9.3	Did you pay for diagnostic facilities (Laboratory, Radiology, Ultrasound and specialized investigation etc)	1-Yes 0- No	
**10**	**Quality of Service Delivery at the facility**		
10.1	Whether shelter for night available for your family members within the premises?	1-Yes 0- No	
10.2	How frequently did doctor and nurses visit for checkup?		
1- More than 2 visits 2- Just 1 visit 3- Rarely visited			
10.3	How much time did doctor spent on you?		
1- More than 10 minutes 2- Between 5-10 minutes 3- Less than 5 minutes			
10.4	Was the instructions during consultation and discharge were clear?		
1- Yes, very clear 2- Somewhat clear 3- Not at all clear			
10.5	How was the behavior of doctor and nurses with the patient and baby?		
1- Helpful & Polite 2- Bad at times but acceptable 3- Rude, unhelpful, Not acceptable			
10.6	When you had important question to ask a doctor/nurse, did you get answers that you could understand?		

**1- Yes, always 2- I had no need to ask 3- No**			
**11**	**Discharge from Hospital**		
**11.1**	Do you feel the condition of the mother is good for discharge?	**1-Yes 0- No 8. Don't Know**	
**11.2**	Do you feel the condition of the baby is good for discharge?	**1-Yes 0- No 8.Don't Know**	
**11.3**	Are you sent home on medicines?	**1-Yes 0- No 8.Don't Know**	
**11.4**	Do you understand properly how to give the medicines?	**1-Yes 0- No**	
**12**	**Overall Satisfaction**		
**12.1**	Overall, did you feel you were treated with respect and dignity while you were in hospital?	**1-Yes 0- No**	
**12.2**	Would you like to return to this health facility for your subsequent health needs?	**1-Yes 0- No**	
**12.3**	Would you like to recommend this hospital to others?	**1-Yes 0- No**	
**12.4**	Were you satisfied by the care and treatment given at the health facility?		
**1- Very Satisfied 2- Somewhat satisfied 3- Dissatisfied**			
**12.5**	Do you have any other comments or suggestions on how care can be improved for mothers and babies in this hospital?		

UNIQUE ID:

Improving Quality of Care for Mother and Newborn at District Hospitals and FRU's of 3 Districts of Haryana

PATIENT SATISFACTION QUESTIONNAIRE
SICK NEWBORN CARE

Name and Address of the facility: ____

____

Name and Address of the patient: ____

____

Date of Hospitalization: ____________________ **Date of Discharge:** ____________________

Consent given: ____

Date of Interview: ____

Start time: ____

End time: ____

Interviewed by: ____

Instructions:

- *Greet the patient and introduce yourself.*
- *Explain the patient why you are taking up this questionnaire.*
- *Take the consent and then start the questions.*
- *If you do not get satisfactory response, promptly please repeat the question and probe wherever necessary.*
- *If a patient does not agree to respond, say thanks to them for giving you the time.*
- *Please introduce each question separately and record the response.*
- *The elicited information will be treated as confidential and don't discuss or share the responses with any third/outside person.*
- ***The data will be recorded using the TABLET.***

		Response	
**1**	**General Details**		
**1.1**	How old is your baby?		
**1.2**	Is your child born in this same hospital?	**1-Yes 0- No**	
**1.3**	If, not then where?		
	1. At home 2. Any other hospital 3. Others		
**1.4**	Date of admission in this hospital?	___/___/____	
**1.5**	Length of stay	______ Days	
**1.6**	How far away do you live from the hospital?	_____ **Km**	
**1.7**	How long did it take to reach the hospital?	__ Hrs __ Min	
**1.8**	Have you been referred to this facility- from other facility/Family or self/Private doctor?	**1-Yes 0- No**	
**1.9**	Did you get a free transport to reach the facility (from home/ another health facility to this facility/ hospital)?	**1-Yes 0- No**	
**1.10**	Did you attend antenatal care during your pregnancy?	**1-Yes 0- No**	
**1.11**	Where did you attend antenatal appointments?		
**1.12**	Place of delivery		
**1.13**	Mode of delivery		
**1.14**	When did you bring your child first at this hospital?		
**1.15**	Did the ANM/ASHA arrange the ambulance/ transport for you?	**1-Yes 0- No**	
**1.16**	Were you referred to this hospital?	**1-Yes 0- No**	
**1.17**	If yes, who referred the mother to this hospital (Designation of person)		
**1.18**	Have you reached hospital after visiting multiple facilities	**1-Yes 0- No**	
**2**	**Experience at Admission**		
**2.1**	Did your child got admitted through OPD?	**1-Yes 0- No**	
**2.2**	If yes, was the person at registration helpful to you?	**1-Yes 0- No**	
**2.3**	Did your child got admitted through Emergency?	**1-Yes 0- No**	
**2.4**	If yes, was the person at registration/ in emergency helpful to you?	**1-Yes 0- No**	
**2.5**	Did the ward attendant/support staff assist you for reaching to ward/ SNCU/NICU?	**1-Yes 0- No**	
**2.6**	Were you able to locate the ward/ SNCU/NICU easily (location & sign boards)?	**1-Yes 0- No**	
**3**	**Attitude/ Behavior of the Staff**		
**3.1**	How was the attitude and behavior of the staff, at the reception, when you reached the facility?		
	**1- Helpful & Polite 2- Bad at times but acceptable 3- Rude, unhelpful, Not acceptable**		
**3.2**	Was the adequate information displayed at the reception/ registration counter?		
	**1- Yes 2- Somewhat adequate 3- Not at all clear**		
**3.3**	How prompt was the registration process?		
	**1- Very Prompt 2- Neither prompt nor delayed 3- Delayed**		
**4**	**Initial Assessment**		
**4.1**	When you arrived at the hospital, how long did you have to wait before a nurse/doctor examined your baby?		
	**1- Immediately 2- Between 15-30 minutes 3- More than 30 minutes**		

**4.2**	Was a family member allowed to stay in the same room with you during the examination?	**1-Yes 0- No**	
**4.3**	Did the health workers explain what they were doing and what would happen next?	**1-Yes 0- No**	
**4.4**	Overall, are you happy with the support you received from the health professionals?	**1-Yes 0- No**	
**1- Very Satisfied 2- Neither satisfied nor dissatisfied 3- Dissatisfied**			
**5**	**Cleanliness/ Hygiene**		
**5.1**	How was the general cleanliness/ hygiene of the building, corridor and premises		
**1- Very clean & hygienic 2- Not so clean but acceptable 3-Very poor & unhygienic 8. Don't Know**			
**5.2**	How was the cleanliness of the area where you(mother)/ family member were staying in the hospital?		
**1- Very clean & hygienic 2- Not so clean but acceptable 3- Very poor & unhygienic 8. Don't Know**			
**6**	**Privacy/ Basic Amenities**		
**6.1**	Were you satisfied with privacy and confidentiality that you got in the wards?		
**1- Very Satisfied 2- Neither satisfied nor dissatisfied 3- Dissatisfied**			
**6.2**	Is 24/7 safe and clean supply of drinking water available in the hospital?	**1-Yes 0- No 8. Don't Know**	
**6.3**	Do the availability of toilet/hand washing facility appropriate?	**1-Yes 0- No 8. Don't Know**	
**6.4**	Do you find the toilets well maintained and clean?	**1-Yes 0- No 8. Don't Know**	
**6.5**	Are there provision of shelter and cafeteria for care takers/ attendants of patients?	**1-Yes 0- No 8. Don't Know**	
**7**	**Out of Pocket Expenses**		
**7.1**	Did you make any unofficial/ informal payments?	**1-Yes 0- No**	
**7.2**	Did you pay for any medications/ any other out of pocket expenses? (Please specify)	**1-Yes 0- No**	
**7.3**	Did you pay for diagnostic facilities (Laboratory, Radiology, Ultrasound and specialized investigation etc)	**1-Yes 0- No**	
**8**	**Quality of Service Delivery at the facility**		
**8.1**	Was the doctor's availability during day time adequate?	**1-Yes 0- No**	
**8.2**	Was the nurse's availability during day time adequate?	**1-Yes 0- No**	
**8.3**	How frequently did doctor and nurses visit for checkup?		
**1- More than 2 visits 2- Just 1 visit 3- Rarely visited**			
**8.4**	How much time did doctor spent on your baby?		
**1- More than 10 minutes 2- Between 5-10 minutes 3- Less than 5 minutes**			
**8.5**	Did the doctor explain/ update you about the status of your child?	**1-Yes 0- No**	
**8.6**	Did nurse give you some guidance and/or support for child care?	**1-Yes 0- No**	
**8.7**	Did you learn anything new on how to keep your child healthy while	**1-Yes 0- No**	

	in the hospital?		
8.8	Was the instructions/information during consultation were clear?		
	**1- Yes, very clear 2- Somewhat clear 3- Not at all clear**		
8.9	When you had important question to ask a doctor/nurse, did you get answers that you could understand**?**		
	**1- Yes, always 2- I had no need to ask 3- No**		
**9**	**Discharge from hospital**		
**9.1**	Do you feel the condition of the child is good for discharge?	**1-Yes 0- No 8. Don't Know**	
**9.2**	Did you receive a discharge sheet explaining the events and problem and follow up?	**1-Yes 0- No**	
**9.3**	Have you been explained about the care at home (medicines/ when to come)?	**1-Yes 0- No**	
**9.4**	Do you know about the danger signs of the child?	**1-Yes 0- No**	
**9.5**	Are you sent home on medicines?	**1-Yes 0- No 8. Don't Know**	
**9.6**	Do you understand properly how to give the medicines?	**1-Yes 0- No**	
**9.7**	Did the doctor/staff tell you where to go for follow-up visit?	**1-Yes 0- No**	
**10**	**Overall Satisfaction**		
**10.1**	Overall, did you feel you were treated with respect and dignity while you were in hospital?	**1-Yes 0- No**	
**10.2**	Were you satisfied by the care and treatment given at the health facility?		
	**1- Very Satisfied 2- somewhat satisfied 3- Dissatisfied**		
**10.3**	Would you like to return to this health facility for your subsequent health needs?	**1-Yes 0- No**	
**10.4**	Would you recommend other family members to come to this hospital?	**1-Yes 0- No**	
**10.5**	Do you have any other comments or suggestions on how care can be improved for mothers and babies in this hospital?		

UNIQUE ID:

Improving Quality of Care for Mother and Newborn at District Hospitals and FRU's of 3 Districts of Harvana

CASE RECORD REVIEW FOR ANTENATAL CHECKUP

Name and Address of the Facility: ______________________________

Name and Address of the Patient: ______________________________

Date of Data Collection: ____

Start time: ____________________ **End time:** ______________________

Collected by: ____

S.No.	Case Record	Response (mark appropriate box)	Documented (Please give details)
**1.**	**GENERAL INFORMATION**		
1.1.	MCTS No.		
1.2.	Name of the Patient	☐ Yes ☐ No	
1.3.	Age *(in years)*	☐ Yes ☐ No	
1.4.	Education	☐ Yes ☐ No	
	☐ Illiterate ☐ Primary School ☐ High School ☐ Graduate ☐ Postgraduate		
1.5.	Name of the Husband	☐ Yes ☐ No	
1.6.	Mobile No.	☐ Yes ☐ No	
1.7.	Address	☐ Yes ☐ No	
1.8.	No. of present ANC visit	☐ Yes ☐ No	
**2.**	**HISTORY OF PREGNANCY**		
2.1.	Date of LMP *(DD/MM/YYYY)*	☐ Yes ☐ No	☐☐ ☐☐ ☐☐☐☐
2.2.	EDD *(DD/MM/YYYY)*	☐ Yes ☐ No	☐☐ ☐☐ ☐☐☐☐
2.3.	**Pregnancy status** *(Please fill in the numbers in relevant box)*	☐ Yes ☐ No	Previous birth defects ☐<br>Previous C-section ☐
	☐ Gravida ☐ Para ☐ Live births ☐ Still births ☐ Abortions ☐ No. of living children		
2.4.	Last Delivery *(DD/MM/YYYY)*	☐ Yes ☐ No	☐☐ ☐☐ ☐☐☐☐
2.5.	Place of first ANC checkup	☐ Yes ☐ No	
	☐ SC ☐ PHC ☐ DH ☐ Private Nursing home ☐ Medical college		
2.6.	Total ANC visits till now	☐ Yes ☐ No	

3.	GENERAL EXAMINATION (Please check the ANC card and write values if mentioned)				
Parameter		Current visit		Last visit	
		Checked	Mentioned	Checked	Mentioned
a	Pallor	☐ Yes ☐ No	☐ Not mentioned	☐ Yes ☐ No	☐ Not mentioned
b	Pulse Rate	☐ Yes ☐ No	☐ Not mentioned	☐ Yes ☐ No	☐ Not mentioned
c	Blood Pressure	☐ Yes ☐ No	☐ Not mentioned	☐ Yes ☐ No	☐ Not mentioned
d	Weight *(in kgs)*	☐ Yes ☐ No	☐ Not mentioned	☐ Yes ☐ No	☐ Not mentioned
e	Edema	☐ Yes ☐ No	☐ Not mentioned	☐ Yes ☐ No	☐ Not mentioned

4.	ABDOMINAL EXAMINATION				
		Current visit		Last visit	
		Checked	Mentioned	Checked	Mentioned
a	Fundal height	☐ Yes ☐ No	☐ Not mentioned	☐ Yes ☐ No	☐ Not mentioned
b	Foetal lie and presentation	☐ Yes ☐ No	☐ Not mentioned	☐ Yes ☐ No	☐ Not mentioned
c	Foetal heart rate	☐ Yes ☐ No	☐ Not mentioned	☐ Yes ☐ No	☐ Not mentioned
d	Foetal movements	☐ Yes ☐ No	☐ Not mentioned	☐ Yes ☐ No	☐ Not mentioned
e	Multiple pregnancies?	☐ Yes ☐ No	If yes, how many?<br>☐ Not mentioned		

5.	INVESTIGATIONS				
		Current visit		Last visit	
		Advised	Done	Advised	Done
a	Hemoglobin	☐ Yes ☐ No	☐ Yes ☐ No<br>Value:	☐ Yes ☐ No	☐ Yes ☐ No<br>Value:
b	Urine sugar	☐ Yes ☐ No	☐ Yes ☐ No<br>Value:	☐ Yes ☐ No	☐ Yes ☐ No<br>Value:
c	Urine protein	☐ Yes ☐ No	☐ Yes ☐ No<br>Value:	☐ Yes ☐ No	☐ Yes ☐ No<br>Value:
d	USG Abdomen	☐ Yes ☐ No	☐ Yes ☐ No<br>☐ Normal<br>☐ Abnormal	☐ Yes ☐ No	☐ Yes ☐ No<br>☐ Normal<br>☐ Abnormal
e	Blood group, Rh factor	☐ Yes ☐ No	☐ Yes ☐ No	☐ Yes ☐ No	☐ Yes ☐ No
f	VDRL	☐ Yes ☐ No	☐ Yes ☐ No<br>☐ Positive<br>☐ Negative	☐ Yes ☐ No	☐ Yes ☐ No<br>☐ Positive<br>☐ Negative
g	HIV	☐ Yes ☐ No	☐ Yes ☐ No<br>☐ Positive<br>☐ Negative	☐ Yes ☐ No	☐ Yes ☐ No<br>☐ Positive<br>☐ Negative
h	Urine Pregnancy test	☐ Yes ☐ No	☐ Yes ☐ No<br>☐ Positive<br>☐ Negative	☐ Yes ☐ No	☐ Yes ☐ No<br>☐ Positive<br>☐ Negative

6.	INTERVENTIONS				
		Current visit		Last visit	
		Given	Mentioned	Given	Mentioned
a	IFA tablets given	☐ Yes ☐ No	☐ Yes ☐ No	☐ Yes ☐ No	☐ Yes ☐ No
b	TT injections	☐ Yes ☐ No	☐ Yes ☐ No	☐ Yes ☐ No	☐ Yes ☐ No

7.	COUNSELLING		
		Mentioned	Done
a	Recognizing and preparing for danger signs (complication readiness)	☐ Yes ☐ No	☐ Yes ☐ No
b	Planning and preparing for birth (birth preparedness)	☐ Yes ☐ No	☐ Yes ☐ No
c	Diet and rest	☐ Yes ☐ No	☐ Yes ☐ No
d	Breastfeeding	☐ Yes ☐ No	☐ Yes ☐ No
e	Sex during pregnancy	☐ Yes ☐ No	☐ Yes ☐ No
f	Domestic violence	☐ Yes ☐ No	☐ Yes ☐ No

UNIQUE ID:

Improving Quality of Care for Mother and Newborn at District Hospitals and FRU's of 3 Districts of Haryana

CASE RECORD REVIEW FOR RECENTLY DELIVERED WOMEN

Name and Address of the facility: ________________________________

Name and Address of the Patient: _________________________________

Date of Data Collection: ____

Start time: ____

End time:____

Collected by: ____

Instructions:

1. *Please write code 1 or 0 for 'yes' or 'no' respectively if the detail is documented or not documented (if you find it in case sheet)*
2. *If documented, proceed to next column, and fill in the value where required or fill code for the appropriate option.*
3. *Please follow* ***24 hours format*** *for filling time.*
4. *Please do the review for those women who have been discharged.*
5. *Fill multiple codes where applicable, in sequence (if any).*

S.No.	Case Record	If Documented? (Please code 1 or 0 for 'yes' or 'no')	Documented *(please give details)*
**1.**	**GENERAL INFORMATION**		
1.1.	Inpatient ID	☐ Yes ☐ No	
1.2.	Name of the mother	☐ Yes ☐ No	
1.3.	Age *(in years)*	☐ Yes ☐ No	
**2.**	**ADMISSION/DISCHARGE INFORMATION**		
2.1.	Date of Admission *(DD/MM/YYYY)*	☐ Yes ☐ No	☐☐ ☐☐ ☐☐☐☐
2.2.	Time of Admission *(Hours: mins)*	☐ Yes ☐ No	☐☐ ☐☐
2.3.	Diagnosis on admission	☐ Yes ☐ No	
2.4.	Date of Discharge *(DD/MM/YYYY)*	☐ Yes ☐ No	☐☐ ☐☐ ☐☐☐☐
2.5.	Time of Discharge *(Hours: mins)*	☐ Yes ☐ No	☐☐ ☐☐
**3.**	**VITAL SIGNS ON ADMISSION *(Please mention values/status/ measurement, if documented)***		
3.1.	Blood Pressure	☐ Yes ☐ No	
3.2.	Pulse Rate	☐ Yes ☐ No	
3.3.	Temperature	☐ Yes ☐ No	
3.4.	Pallor/ Hb	☐ Yes ☐ No	
3.5.	Blood Group, Rh Factor	☐ Yes ☐ No	
3.6.	HIV	☐ Yes ☐ No	☐ Positive ☐ Negative
**4.**	**MATERNAL HISTORY**		
4.1.	**Pregnancy status** *(Please fill in the numbers in relevant box)*	☐ Yes ☐ No	Previous birth defects ☐<br>Previous C-section ☐
	☐ Gravida ☐ Para ☐ Live births	☐ Still births ☐ Abortions	☐ No. of living children
4.2.	Past Medical illness<br>*(Please mention, if any)*	☐ Yes ☐ No	
**5.**	**DETAILS OF MONITORING AND DELIVERY**		
5.1.	Foetal heart rate	☐ Yes ☐ No	
5.2.	Partograph	☐ Yes ☐ No	☐ Complete ☐ Incomplete
5.3.	Vaginal examination	☐ Yes ☐ No	
5.4.	Mode of delivery	☐ Yes ☐ No	☐ Vaginal<br>☐ Assisted<br>☐ C-section
	(If option 1, go to Q.5.4.e.)		
5.4.a.	If assisted delivery, indication?	☐ Yes ☐ No	
5.4.b.	If assisted delivery, mode of delivery?	☐ Yes ☐ No	☐ Forceps ☐ Ventouse ☐ Others
5.4.c.	If C-section, indication?	☐ Yes ☐ No	
5.4.d.	If C-section, Type of anesthesia	☐ Yes ☐ No	
5.4.e.	Episiotomy done?	☐ Yes ☐ No	
5.5.	Delivered by	☐ Yes ☐ No	☐ Doctor ☐ Nurse ☐ Others

**6.**	**DETAILS OF THE BABY**		
6.1.	Date of Delivery *(DD/MM/YYYY)*	☐ Yes ☐ No	☐☐ - ☐☐ - ☐☐☐☐
6.2.	Time of delivery *(Hours: mins)*	☐ Yes ☐ No	☐☐ ☐☐
6.3.	Sex of the baby	☐ Yes ☐ No	☐ Male ☐ Female
6.4.	Status of the baby	☐ Yes ☐ No	☐ Live Birth ☐ Still birth
6.5.	Apgar score	☐ Yes ☐ No	At 1 min:<br>At 5 min
6.6.	Resuscitation done?	☐ Yes ☐ No	
6.7.	Any complication?	☐ Yes ☐ No	☐ Yes ☐ No<br>*(Please specify)*
6.8.	Medication given (name & dose)	☐ Yes ☐ No	
**7.**	**HOSPITAL COURSE *(Please fill multiple codes where needed)***		
7.1	Total days of hospital stay	☐ Yes ☐ No<br>हाँ<br>ना	
7.2.	Daily notes/ progress reports	☐ Yes ☐ No	
7.2.a.	If yes, filled for how many days		
7.3.	Complications during stay	☐ Yes ☐ No	☐ Monitoring ☐ Medication<br>*(Please specify)*
7.4.	Outcome	☐ Yes ☐ No	☐ Improved<br>☐ Deteriorated<br>☐ Referred<br>☐ LAMA<br>☐ Death
**8.**	**DISCHARGE/ REFERRAL**		
8.1.	Status of mother	☐ Yes ☐ No	
8.2.	Status of baby	☐ Yes ☐ No	
8.3.	Discharge/Referral note	☐ Yes ☐ No	*(Please write if any specific advice written)*
8.4.	Danger signs explained	☐ Yes ☐ No	
**9.**	**OTHER INFORMATION**		
9.1.	Any blood transfusion<br>(to mother or to baby)	☐ Yes ☐ No	☐ Yes ☐ No<br>*(Please specify)*
9.2.	Any other complication	☐ Yes ☐ No	☐ Yes ☐ No<br>*(Please specify)*

UNIQUE ID:

Improving Quality of Care for Mother and Newborn at District Hospitals and FRU's of 3 Districts of Haryana

CASE RECORD REVIEW FOR SICK NEWBORN CARE UNIT

Name and Address of the facility: ________________________________

Name and Address of the Patient (रोगी का नाम एवं पता): ________________________________

Date of Data Collection: ________________________________

Start time: ________________________________

End time: ________________________________

Collected by: ________________________________

Instructions (दिशानिर्देश) :

1. *Please write code 1 or 0 for 'yes' or 'no' respectively if the detail is documented or not documented (if you find it in case sheet)*
2. *If documented, proceed to next column, and fill in the value where required or fill code for the appropriate option.*
3. *Please follow* ***24 hours format*** *for filling time.*
4. *Please do the review for those women who have been discharged.*
5. *Fill multiple codes where applicable, in sequence (if any).*

S.No	Case Record	If Documented? (Please code 1 or 0 for 'yes' or 'no')	Documented *(Please give details)*
**1.**	**GENERAL INFORMATION**		
1.1.	Inpatient ID/ SNCU regn. No	☐ Yes ☐ No	
1.2.	Age *(Days: Hours)*	☐ Yes ☐ No	☐☐ ☐☐
1.3.	Sex	☐ Yes ☐ No	☐ Male ☐ Female
1.4.	Name of the mother	☐ Yes ☐ No	
1.5.	Date of birth *(DD/MM/YYYY)*	☐ Yes ☐ No	☐☐ ☐☐ ☐☐☐☐
1.6.	Time of birth *(Hours: mins)*	☐ Yes ☐ No	☐☐ ☐☐
**2.**	**ADMISSION/DISCHARGE INFORMATION**		
2.1.	Date of Admission *(DD/MM/YYYY)*	☐ Yes ☐ No	☐☐ ☐☐ ☐☐☐☐
2.2.	Time of Admission *(Hours: mins)*	☐ Yes ☐ No	☐☐ ☐☐
2.3.	Diagnosis on admission	☐ Yes ☐ No	
2.4.	Date of Discharge *(DD/MM/YYYY)*	☐ Yes ☐ No	☐☐ ☐☐ ☐☐☐☐
2.5.	Time of Discharge *(Hours: mins)*	☐ Yes ☐ No	☐☐ ☐☐
**3.**	**DELIVERY INFORMATION**		
3.1.	Place of delivery	☐ Yes ☐ No	
3.2.	Mode of delivery	☐ Yes ☐ No	☐ Vaginal<br>☐ Assisted<br>☐ C-section
3.2.a	If C-section or Assisted, indication?	☐ Yes ☐ No	
3.3.	Maternal Problem *(Please specify)*	☐ Yes ☐ No	
3.4.	Birth weight *(in kgs)*	☐ Yes ☐ No	
3.5.	Apgar score	☐ Yes ☐ No	At 1 min<br>At 5 min
3.6.	Resuscitation done?	☐ Yes ☐ No	
3.7.	Period of Gestation *(MM:DD)*	☐ Yes ☐ No	☐☐ ☐☐
**4.**	**HISTORY OF PRESENT PREGNANCY**		
4.1.	LMP/EDD *(DD/MM/YYYY)*	☐ Yes ☐ No	☐☐ ☐☐ ☐☐☐☐
4.2.	ANC Details	☐ Yes ☐ No	
4.3.	Any pregnancy induced complication(e.g. PIH, Diabetes, APH, maternal fever) *(Please specify)*	☐ Yes ☐ No	

5.	CLINICAL DETAILS		
5.1.	Presenting complaint<br>*(Please specify)*	☐ Yes ☐ No<br>हाँ ना	
5.2.	Vital signs on admission	☐ Yes ☐ No<br>हाँ ना	Heart rate (हृदय गति) ________________<br>Pulse (नब्ज़)<br>________________________<br>Temperature (तापमान) ________________
5.3.	Major Congenital Malformation	☐ Yes ☐ No<br>हाँ ना	
5.4.	Weight at admission	☐ Yes ☐ No<br>हाँ ना	
5.5.	Head circumference	☐ Yes ☐ No<br>हाँ ना	
5.6.	If breastfeeding?	☐ Yes ☐ No<br>हाँ ना	
**6.**	**HOSPITAL COURSE**		
6.1.	Total days of hospital stay	☐ Yes ☐ No	
6.2.	Daily notes/ progress reports	☐ Yes ☐ No	
6.2.a.	If yes, filled for how many days		
6.3.	Medications given (name and dose)	☐ Yes ☐ No	
6.4.	Investigations (as per need)	☐ Yes ☐ No	
6.5.	Complications during stay	☐ Yes ☐ No	☐ Monitoring ☐ Medication<br>*(Please specify)*
6.6.	Outcome	☐ Yes ☐ No	☐ Improved<br>☐ Deteriorated<br>☐ Referred<br>☐ LAMA<br>☐ Death
**7.**	**DISCHARGE/ REFERRAL**		
7.1.	Status of baby	☐ Yes ☐ No	
7.2.	Weight at discharge	☐ Yes ☐ No	
7.3.	Discharge/Referral note<br>*(Please write if any specific advice written)*	☐ Yes ☐ No	

UNIQUE ID:

Improving Quality of Care for Mother and Newborn at District Hospitals and FRU's of 3 Districts of Haryana

CLIENT FLOW ANALYSIS
ANTENATAL CLINIC

Name and Address of the facility: ______________________________

Name and Address of the patient: ______________________________

Date of Observation: ______________________________

Start time of Observation: ______________________________

End time of Observation: ______________________________

Observed by: ______________________________

Instructions:

- *Introduce the patient about the research and the importance of this process and take a proper consent before proceeding.*
- *Tell the patient that he will be followed for all his proceedings in the hospital until his exit.*
- *Use the tablet to record the timings of entry and exit of the patient from each department in the hospital.*
- *Enter the observation start and end time precisely.*
- *Please add remarks for elaborating the observations for delays, etc.*
- *Please inform the patient that any of the information will be kept confidential and it will be used for further improving the hospital procedures.*
- *Appreciate the patient for her cooperation and thank her for the same.*

GENERAL INFORMATION	PREGNANCY INFORMATION
Name of the husband:	LMP: EDD:
Age of the mother:	Gestation period:
Distance from home:	No. of ANC visits:
Accompanied by:	G ☐ P ☐ L ☐ S ☐ A ☐
Purpose of the Visit:	ANC Records:
Consent given:	Medications:

Any Other Information:

Place	Start time	Service provider<br>1. Doctor<br>2. Nurse/ ANM<br>3. Supporting staff<br>4. Receptionist<br>5. Others	Patient/ family	Event/ activity	End time
**Any comments:**					

UNIQUE ID:

Improving Quality of Care for Mother and Newborn at District Hospitals and FRU's of 3 Districts of Haryana

CLIENT FLOW ANALYSIS
LABOUR ROOM

Name and Address of the facility: ___

Name and Address of the patient: ___

Date of Observation: ___

Start time of Observation: ___

End time of Observation: ___

Collected by: ___

Instructions:

- *Introduce the patient about the research and the importance of this process and take a proper consent before proceeding.*
- *Tell the patient that he will be followed for all his proceedings in the hospital until his exit.*
- *Use the tablet to record the timings of entry and exit of the patient from each department in the hospital.*
- *Enter the observation start and end time precisely.*
- *Please add remarks for elaborating the observations for delays, etc.*
- *Please inform the patient that any of the information will be kept confidential and it will be used for further improving the hospital procedures.*
- *Appreciate the patient for her cooperation and thank her for the same.*

GENERAL INFORMATION	PREGNANCY INFORMATION
Name of the husband:	LMP: EDD:
Age of the mother:	Gestation period:
Distance from home:	No. of ANC visits:
Accompanied by:	G ☐ P ☐ L ☐ A ☐ S ☐
Purpose of the Visit:	ANC Records:
Consent given:	Onset of labor:

***<u>Other information's</u>*:**

Place	Start time	Service provider<br>1. Doctor<br>2. Nurse/ ANM<br>3. Supporting staff<br>4. Receptionist<br>5. Others	Patient/ family	Event/ activity	End time
**Any comments:**					

UNIQUE ID:

Improving Quality of Care for Mother and Newborn at District Hospitals and FRU's of 3 Districts of Haryana

KNOWLEDGE ASSESSMENT OF PROVIDERS FOR MATERNAL CARE

Name and Address of the facility: ____

Commencing Time: ______________________ **Concluding Time:** ______________________

Date of Assessment: ____

Assessed by: ____

Health Functionary: ____

{1. Medical Officer 2. Obstetrician/ Gynecologist 3. Ayush Doctor 4. Nurse 5. Any other (please specify)}

<u>Instructions for the observer undertaking the knowledge assessment</u>

1. *At each health facility, the designated health functionary is to be selected for knowledge assessment.*
2. *The doctor/health care provider managing delivery and neonatal resuscitation will be considered for knowledge assessment.*
3. *If more than one person is available at the same time, the senior is to be chosen.*
4. *Do not make any judgments/comments/gesture while making the assessment.*
5. *The data collected is to be treated as confidential and not to be communicated to any other health official/functionary.*
6. *Explain in prior to the assessee about where and how the answers are to be filled in the form.*

S.No.	Questions	Responses
**Sec 1**	**GENERAL INFORMATION**	
1.	Name of the Health Provider	
2	Total number of months and years in service	
3	When and where did you receive last training on….?<br>*(Skip to Section 2 if don't know)*	Don't Know ☐
3.1	Pregnancy Care	
3.1.a	Place	
3.1.b	Date *(MM/YYYY)*	
3.2	Newborn Resuscitation	
3.2.a	Place *(MM/YYYY)*	
3.2.b	Date	

**Sec 2**	**KNOWLEDGE ASSESSMENT OF MATERNAL SERVICES**	
**S.No.**	**Statement**	**Response**
**1.**	**True or False**<br>*(Please put a tick mark (✓)in front of true and (X) in front of false statement)*	
1.1	Severe pre-eclampsia is defined as<br>"1. BP is ≥ 140/90 to 160/110 along with Proteinuria (Uristix test by dip stick test - Trace/ 1+/2+) and symptoms (headache/ blurring vision/ epigastric pain/ Oliguria/ pulmonary edema/ abnormal edema over hands/ face abdomen and vulva)- OR<br>2- BP is ≥ 160/110 with Proteinuria (3+/4+) with/out symptoms"	☐
1.2	Headache, Blurring of vision, epigastric pain, Oliguria, pulmonary edema, abnormal edema over hands, face abdomen and vulva are danger signs of Pregnancy Induced hypertension.	☐
1.3	Maternal sepsis cannot be prevented by limiting procedures in the birth canal such as PV examination.	☐
1.4	Pregnant woman with Gestational Diabetes Mellitus and their offspring are at increased risk of developing Type II Diabetes mellitus in later life. They should be counseled for healthy lifestyle and behavior, particularly role of diet & exercise.	☐
1.5	Pregnant women with Gestational Diabetes Mellitus should deliver at home since complications during delivery are rare.	☐
1.6	Anemia is defined as Hemoglobin level < 11 gm% in pregnancy or immediate post partum period.	☐
1.7	The indications for forceps delivery include- Foetal distress, Maternal distress or Prophylactic - cardiac disease, severe pre eclampsia / eclampsia, anemia, etc.	☐
1.8	The pre-requisites for conducting vacuum delivery include- Vertex presentation, Term foetus, No CPD, Ruptured membranes, Cervix fully dilated, Station at or below +1	☐
1.9	Insulin therapy is the accepted medical management of 'Pregnant woman with gestational diabetes mellitus' not controlled by Medical Nutrition therapy for 2 weeks.	☐
1.10	The most common characteristic signs and symptoms of diabetic pregnant female include: tremors of hands, sweating, palpitations, hunger, easy fatigability, headache, mood changes, irritability, low attentiveness, and tingling sensation around the mouth/lips or any other abnormal feeling.	☐

2.	**Choose the Correct Answer**<br>*(Please write the correct option in the box provided in response column)*	
2.1	Eclampsia is diagnosed when-<br>a. Convulsions are present in a pregnant female along with diastolic blood pressure ≥ 90 mm Hg and or systolic BP ≥140 mm Hg after 20 weeks; and Proteinuria 2+ or more.<br>b. Two readings of diastolic blood pressure 90 mm Hg and systolic BP 140-160 mm Hg 4 hours apart after 20 weeks of pregnancy.<br>c. BP is ≥ 160/110 with Proteinuria (3+/4+) with/out symptoms.<br>d. BP is ≥ 140/90 to 160/110 along with Proteinuria.	☐
2.2	Which of the following is diagnosed in the antenatal female by using 75 gm Oral Glucose Tolerance Test (OGTT) irrespective of last meal with a threshold value of Plasma glucose >140 mg/dl?<br>a. Pregnancy Induced Hypertension.<br>b. Pre-eclampsia.<br>c. Gestational Diabetes Mellitus (GDM).<br>d. Pre-diabetes.	☐
2.3	Mild to moderate anemia is treated by-<br>a. Iron and folic acid tablets (500 mg elemental iron + 0.5 mg folic acid) twice daily.<br>b. Iron and folic acid tablets (200 mg elemental iron + 0.5 mg folic acid) twice daily.<br>c. Iron and folic acid tablets (100 mg elemental iron + 1 mg folic acid) twice daily.<br>d. Iron and folic acid tablets (100 mg elemental iron + 0.5 mg folic acid) twice daily.	☐
2.4	Contraindications for forceps delivery are as follows-<br>a. Contracted pelvis.<br>b. Incomplete dilatation of cervix.<br>c. Malpresentation.<br>d. All the above.	☐
2.5	The indications for vacuum delivery include-<br>a. Maternal distress, Prophylactic - cardiac disease, severe pre-eclampsia / eclampsia, anemia, etc. or Failure of descent or rotation of head.<br>b. Contracted pelvis, incomplete dilatation of cervix, Malpresentation.<br>c. None of the above.<br>d. Options A & B.	☐
2.6	Which of the following are indications of antenatal corticosteroids?<br>a. True labour pains.<br>b. Conditions leading to imminent delivery. E.g. Ante partum hemorrhage, preterm premature rupture of membranes, severe pre-eclampsia/ eclampsia.<br>c. Frank chorioamniotis presenting with fever, lower abdominal pain, foul smelling vaginal discharge, tender uterus, maternal and foetal tachycardia.<br>d. Sepsis in pregnancy	☐

3.	**Multiple Choice Questions**<br>*(Please write the correct options (multiple) in response column)*	
3.1	Which of the following are features of true labour pains?-<br>a. Regular and predictable<br>b. Irregular<br>c. First felt in lower back, then lower abdomen<br>d. No cervical changes<br>e. Confined to lower abdomen<br>f. Not relieved by rest<br>g. Intensity, frequency, duration does not increase with time<br>h. Accompanied by cervical changes<br>i. Intensity, frequency and duration rises with time<br>j. Show absent<br>k. 'Show' present- blood stained mucus<br>l. Relieved by rest	
3.2	Which of the following are indications for starting antibiotics in pregnancy?<br>a. Fever (Temperature above 38^0 C/ 100.5^0 F)<br>b. Foul smelling vaginal discharge<br>c. Prolonged labour> 24 hours<br>d. Normal Labour<br>e. Obstructed labour<br>f. Planned Caesarean section<br>g. Lower abdominal tenderness after delivery<br>h. After removal of placenta<br>i. Manual removal of placenta<br>j. Blood pressure more than 140/90 mm Hg.<br>k. Preterm Prelabor rupture of membranes (before 37 weeks)<br>l. After normal delivery<br>m. Prolonged rupture of membranes – more than 12 hours without labour pains or more than 18 hours with labour pain	
**4.**	**Fill in the response column with correct stage of labour**	
4.1	**Characteristic features**	**Stages of Labour**
4.1.1	Full dilatation of cervix to delivery of baby includes-<br>a. Full cervical dilatation<br>b. Bulging thinned out perineum<br>c. Gaping anus and vagina<br>d. Head visible at perineum	Stage___
4.1.2	Onset of labour pains to dilatation of cervix-<br>Part 1- Cervix dilatation < 4cms;<br>≤ 2 contractions/10 mins<br>Part 2- Cervix dilation ≥4cms;<br>≥3 contractions/10 mins lasting >40 sec;<br>Rate of contraction ≥1cm/hour	Stage___
4.1.3.	For 2 hours after delivery of placenta	Stage___
4.1.4	From delivery of baby to delivery of placenta	Stage___

4.2	**Management**	**Stage of Labour**
4.2.1	Review of condition of mother and baby	Stage___
4.2.2	Performing AMTSL	Stage___
4.2.3	Monitoring of Labour with partograph	Stage___
4.2.4	Preparation of birth of baby	Stage___

**5.**	**Fill in the blanks**<br>*(Please fill in the correct answer (s) for blanks in the response column)*<br>*(Fill in multiple blanks in responses as a, b, c, d……..)*	
5.1	Pregnancy-induced hypertension is defined as "Two readings of diastolic BP _______ and systolic BP _______ 4 hours apart after 20 weeks gestation and no Proteinuria"	
5.2	_______ can be used for controlling blood pressure in pregnancy. In severe cases, _______can be used.	
5.3	Gestational diabetes mellitus (GDM) can be easily controlled by diet (Medical Nutrition Therapy) and exercise. Only in few women in whom blood glucose is not controlled by diet, _______are required.	
5.4	Anemia is classified based on _______as mild (10-11gm %), moderate (7-10 gm %), severe (4 - 7gm %), and very severe (<4 gm %).	
5.5	The pre-requites for conducting a forceps delivery include-<br>a. Pelvis should be _______<br>b. Cervix should be _______<br>c. Position should be _______<br>d. Station of head should be _______<br>e. Membranes should be _______<br>f. Bladder and rectum should be _______<br>g. On PA examination -_______	
5.6	Contra indications for vacuum delivery are:<br>a. _______<br>b. _______<br>c. _______<br>d. _______	
5.7	In women with mild pre-eclampsia or mild gestational hypertension at term (after 37 weeks), _______is recommended.	
5.8	The antibiotics recommended during sepsis in pregnancy include the following? Write their correct doses and frequency?<br>a. Ampicillin _______oral or intravenous<br>b. Metronidazole _______ oral or 500 mg 8 hourly intravenous<br>c. Gentamicin _______ intramuscularly	
5.9	Testing for GDM is recommended _______during Antenatal natal period. The first testing should be done during _______in pregnancy.<br>The second testing should be done _______of pregnancy if the first test is negative. There should be at least 4 weeks gap between the two tests.	
5.10	Medical Nutrition Therapy recommended for initial treatment of GDM for 2 weeks includes the following recommendations for carbohydrates, proteins and fats-<br>a. Addition of _______above the adult requirement is recommended	

	during second and third trimester.<br>b. _______Carbohydrate serving at each major meal and 1–2 carbohydrate serving at each snack. (One serve= approximately 15 grams of carbohydrate)<br>c. Saturated fat intake should be _______% of total calories and dietary cholesterol should be less than 300mg/d.<br>d. Additional _______of protein is recommended to allow for fetal growth. At least 3 serving of protein foods are advised every day to meet the increased demand.<br>e. High _______ foods may help control blood sugar.	

Thank you for giving your precious time.

Observer's Name ______________________

Signature ____________________________

UNIQUE ID:

Improving Quality of Care for Mother and Newborn at District Hospitals and FRU's of 3 Districts of Haryana

KNOWLEDGE ASSESSMENT OF NURSES – LABOUR ROOM

**1.1**	**District**		**1.2**	**Date**	
**1.3**	**Facility type**	1-District Hospital<br>2- FRU-CHC<br>3-CHC	**1.4**	**Health functionary**	1-Staff nurse<br>2- LHV-<br>3- ANM<br>4- Any other (specify
**1.5**	**Facility name**		**1.6**	**Facility area**	1-Labour room<br>2- Female/PNC ward<br>3- SNCU/NBSU<br>3- ANC OPD<br>4- Any other (specify)
**1.7**	**Total years in service**		**1.8**	**Duration of posting in the area**	
**1.9**	**Last SBA training**		**1.10**	**Last training on newborn care**	
**1.11**	**Last delivery attended on (date)**		**1.12**	**Last newborn resuscitation done on (date)**	

Instructions for Health Staff for filling the format

1. Kindly read all the questions and carefully respond as indicated for specific question.
2. You can take your time while reading the questions and ask the quality team member if there is any confusion.
3. Please put a tick mark (√) in front of correct response.

S.No.	Question
2.1	What is usually given to the delivering women before delivery of placenta to prevent haemorrhage?<br>a. Oxytocin<br>b. Misoprostol<br>c. Vitamin K<br>d. Nothing
2.2	If you have a woman in labour with a BP of 160/110, protein in urine and having convulsions, what is the **ONE key drug of choice**?<br>a. Diazepam<br>b. Calcium gluconate<br>c. Magnesium<br>d. Phenytoin
2.3	How do you massage uterus after delivery?<br>a. Continuously till bleeding stops<br>b. Every 15 minutes till the uterus is contracted well<br>c. Every 10 minutes till the uterus is contracted well<br>d. Every 5 minutes till the uterus is contracted well
2.4	In a recently delivered women the uterus is well contracted, placenta is expelled complete, but the bleeding is continuing. **What is the likely possibility**?<br>a. Atonic uterus<br>b. Uterine rupture<br>c. Cervical trauma<br>d. Retained placenta
2.5	Features of fetal distress include all **EXCEPT**.<br>a. Foetal heart lower rate <120 per minute<br>b. Foetal heart upper rate >160 per minute<br>c. Loss of fetal movement<br>d. No uterine contraction
2.6	Monitoring of the recently delivered women include all **EXCEPT**<br>a. Heart rate<br>b. Blood Pressure<br>c. Vaginal Bleeding<br>d. Uterine contraction
2.7	How long the wrapped sterile (autoclaved) instruments in delivery kit can be used?<br>a. 3 days after autoclave<br>b. 5 days after autoclave<br>c. 7 days after autoclave<br>d. 1 day after autoclave
3.1	Before the birth of the baby the radiant warmer should be kept switched on preferably _________minutes before expected time of birth.<br>a. 15 minutes<br>b. 20 minutes<br>c. 1/2 hour<br>d. d. 1hour
3.2	Normal axillary temperature of newborn should be between ___^{0}C to ___^{0}C<br>a. 36^0C - 37.5^0C<br>b. 36.5^0C - 37.5^0C<br>c. 36^0C - 37^0C<br>d. 36^0C - 38^0C

3.3	Umbilical cord should be cut ____________ (time) after birth with sterile scissors.<br>a. Immediately<br>b. After 1 minutes<br>c. After 2-3 minutes<br>d. After 5 minutes
3.4	After cutting the cord what should be applied on it?<br>a. Spirit<br>b. Gentian violet<br>c. Betadine solution<br>d. Nothing
3.5	Baby bath should be given to remove the vernix after/time<br>a. bath not essential at all<br>b. should be bathed within 1 hour after birth<br>c. should be bathed at least after 6 hours after birth<br>d. should be bathed after 24 hours after birth
3.6	Newborn babies are to be given the vaccines after birth.<br>a. BCG<br>b. OPV<br>c. Hepatis B<br>d. All of above
3.7	After delivery when do you advise the mother for breastfeeding the baby?<br>a. Immediately after delivery<br>b. After mother shifted to ward/ bed<br>c. After 2 hours<br>d. After baby passes urine and stool
3.8	Which drug should be given to mothers with preterm labour?<br>a. Dexamethasone<br>b. Ampicillin<br>c. Tetanus<br>d. Oxytocin
3.9	**Please check the statement and mark True or False**<br>If not crying at birth, there is no activity and the baby is cyanosed, the baby is probably dead and there is no need to initiate ventilation.<br>a. True<br>b. False
3.10	If newborn is not crying and meconium is present how would you do suction<br>a. mouth-→ nose-→trachea<br>b. trachea-→ nose-→mouth<br>c. mouth-→ nose<br>a. d. nose-→ mouth →trachea
3.11	_____ size suction catheter is required for meconium suctioning from mouth and nose in term newborn.<br>b. 10 F<br>c. 12 F<br>d. 8 F<br>e. 14 F

3.12	If a term baby (born at 9 completed months) is not breathing at birth even after initial steps he/she can be resuscitated with bag and mask ventilation using<br>a. room air, even if oxygen is not available<br>b. oxygen but no reservoir<br>c. oxygen with reservoir<br>d. room air with reservoir
3.13	Bag and mask ventilation is to be initiated when heart rate is less than _______ per minute<br>a. 100<br>b. 120<br>c. 60<br>d. 80
3.14	Bag and mask ventilation is usually given at rate .... per minute.<br>a. 30-40<br>b. 40-60<br>c. 60-80<br>d. 50-70
3.15	Hand washing involves ____ steps and done for _____ seconds.<br>a. 6 steps and 120 seconds<br>b. 5 steps and 60 seconds<br>c. 6 steps and 60 seconds<br>d. 5 steps and 120 seconds

4. Please plot the partograph for a women in labour using the following information
Name- L; age- 25 years; Primigravida

	Time	Cervical dilatation	Head descent	Fetal heart rate (beats/min)	Membrane/ amniotic fluid	Uterine contractions (duration)
4.1	10:00 AM	5	2	140	Membrane intact	3 (50 sec)
4.2	2:00 PM	7	2	130	Membrane ruptured	2 (35 sec)
4.3	4:00 PM	9	1	155	Clear	3 (45 sec)

Please use the partograph attached for marking the response.

UNIQUE ID:

Improving Quality of Care for Mother and Newborn at District Hospitals and FRU's of 3 Districts of Haryana

In Depth Interview
Medical Officer In charge

**District:**	**Block:**
**Name of the Facility:**	
**Date of Interview/ Observation**	☐☐☐☐☐☐☐☐
**Start time**	☐☐ ☐☐ (HH: MM)
**End time**	☐☐ ☐☐ (HH: MM)

Guidelines for interviewers

- Greet the patient and introduce yourself.
- Explain the patient why you are taking up this questionnaire.
- Take the consent and then start the questions.
- If you do not get satisfactory response, promptly please repeat the question and probe wherever necessary.
- If a patient does not agree to respond, say thanks to them for giving you the time.
- Please introduce each question separately and record the response.
- The elicited information will be treated as confidential and don't discuss or share the responses with any third/outside person.

1. **Government Health Facility:(Please tick ✓ the appropriate health facility and qualification)**			
**1.1 Type of Health Facility**		**1.2 Respondent Qualification**	
District Hospital	☐	MBBS	☐
CHC	☐	MD/ MS	☐
Block PHC	☐	BAMS/BHMS	☐
24x7 PHC	☐	**1.3 Designation in**	
PHC	☐	MO I/C	☐
Dispensary	☐	Medical Officer	☐
Sub-center	☐	Specialist (Pediatrician, Gynecologist)	☐
Any other; (Specify…………….)	☐	AYUSH doctor	☐
		Others(Specify)	☐

**2. General**		
**2.1**	How long have you been working in this health facility? (months/years)	
**2.2**	Total months/years of service	
**2.3**	What are your current roles and responsibility with respect to maternal and neonatal care?	
**2.4**	How many deliveries and resuscitations of newborns have you attended in last 1 month?	
	A. No. of deliveries attended in last 1 month	
	B. No. of newborn resuscitations attended in last 1 month	

**3. Service Delivery**		
**3.1**	In routine practice, which health staff performs the following services?	
	**Services**	**Staff performing the services**
	Delivery without complication	
	Delivery with complication/ high risk delivery	
	Caesarean section	
	Newborn care at birth	
	Sick newborn care	
	Breastfeeding support	

**3.2**	What are the challenges faced by you and your colleagues for delivering the desired mother and newborn care services?	
	**Challenges faced**	**How do you manage these challenges**
	Infrastructure	
	Equipment	
	Drugs and supplies	
	Support services	
	Other	
**3.3**	What challenges do you face while delivering essential newborn care services and how do you manage these?	
	**Challenges faced**	**How do you manage these challenges**
	Care at delivery	
	Care in the ward	
	Care of sick newborns	
**3.4**	What challenges do you face while delivery of pregnant women?	
	**Challenges faced**	**How do you manage these challenges**
	Delivery without complication	
	Delivery with complication	
	Caesarean section	
	Referred cases with complication	

**3.5**	How long usually the mothers stay at the facility after the delivery?	
	Normal Delivery	
	Caesarean Delivery	
**4. Manpower**		
**4.1**	How many positions of doctors are lying vacant in your health facility?	
**4.2**	If there is a shortage of manpower who addresses the issue so that is does not hinders routine work?	
**4.3**	Do you have adequate staff inside labor room, ANC clinic and SNCU's?	
**4.4**	What happens if a particular department is having more flow of patients? Is there any flexibility in assigning inter departmental responsibilities among staff?	
**4.5**	What is the mechanism of taking leave and who sanctions it?	
**5. Duty Roster**		
**5.1**	Who prepares the duty roster for you?	
**5.2**	Who follows up the prepared roster so that the shifts are routinely changed?	
**5.3**	How many Medical Officers are posted at one time in your department? What is the pattern of shift?	

**5.4**	Do you have flexibility in changing the shifts?
**5.5**	How do you manage when you have double shifts?
**6. Infrastructure**	
**6.1**	Do you have space to accommodate changes inside the department?
**6.2**	Do you have enough beds to accommodate increased number of patients?
**6.3**	Is their regular power supply and clean water for drinking? Any substitute available in case of power cut or irregular water supply?
**7. Training /Skills**	
**7.1**	How many of the total staff are trained for MCH services?
**7.2**	Is there any pre job posting training for newly joined staff?
**7.3**	Is there any on- job training for the staff?
**7.4**	Please let us know about the last training on attended by you?

Timing (Month/Year)	
Place	
Duration (in days)	

What did you like the most in the training?

	What did you dislike the most in the training?
	What was the training methodology used (Lectures/ Hands-on / Practical's)?
	Who conducts the workshop? Who prepares roster for workshop/training and how it is notified? How it is monitored?
**7.5**	What are the opportunities and mechanisms currently in place/adopted to retain the skills of Nurses/ANMs/Doctors?
**7.6**	How the training related to care during delivery and newborn period can be further improved?
**8. Referral services**	
**8.1**	In what situation usually the newborns/mothers (pregnant/recently delivered) are referred to the next level of healthcare?
**8.2**	Where the newborns/ pregnant women/ mothers are usually referred, what is the usual mode of transportation and how long does it takes to reach the next level health facility in your area?

**8.3**	What facilitation is done from facility side for referral and what difficulties/challenges do you face while transporting the sick newborn and mother to next level? *(Probe: monetary/logistics)*
**8.4**	What are the challenges faced related to referral transport experienced by this facility and how are they handled?
**9. Logistics**	
**9.1**	Are you aware of any shortage/irregular supply of drugs and/or supplies needed for care during delivery and newborn period in the last one year? What were the reasons for this shortage and how these situations were managed?
**9.2**	How frequently the families/ parents asked to procure drugs from outside/ store?
**9.3**	How many equipments essential for management of delivery or newborn care are out of order at this moment?
**9.4**	What is the usual mechanism of repair and maintenance of these equipments? *(probe: who is responsible and what is the duration of repair*)

**9.5**	What are the supervisory mechanisms in place at present for maternal and newborn care services?
	Who supervises
	What is the frequency of supervisory visits
	Is any feedback/report provided usually after the supervision?
	What actions are taken after last supervisory visit?
**9.6**	Please let us know about the last supervisory visit to the facility related to maternal and newborn care services?
	Who came for last supervisory visit?
	How long ago the supervisory visit took place?
	What all components were observed?
	What feedback was given and what actions were taken?
**10. Perceptions regarding Quality of care**	
**10.1**	According to you, what is the meaning of quality?
**10.2**	According to you, what are the issues that affect the quality of health services?
**10.3**	What can you do to improve the quality of the health services?

**10.4**	Did any of your relatives, friends or acquaintances ever availed health services at this hospital? If not, any reason?
**11. Others**	
**11.1**	If any shortage of blood and how is it tackled?
**11.2**	Do you arrange blood donation camps on facility basis?

**12**	*Interviewer's Observation*<br>1. Attitude of the respondent<br>a. Very co-operative<br>b. Co-operative<br>c. Non co-operative

Thank the respondent.

Name of Interviewer______________________ Signature________________

UNIQUE ID:

Improving Quality of Care for Mother and Newborn at District Hospitals and FRU's of 3 Districts of Haryana

In Depth Interview – Nurse (Baseline)

**District:**	**Block:**
**Name of the Facility:**	
**Date of Interview/ Observation**	☐☐☐☐☐☐☐☐
**Start time**	☐☐ ☐☐ (HH: MM)
**End time**	☐☐ ☐☐ (HH: MM)

Guidelines for interviewers

- Greet the patient and introduce yourself.
- Explain the patient why you are taking up this questionnaire.
- Take the consent and then start the questions.
- If you do not get satisfactory response, promptly please repeat the question and probe wherever necessary.
- If a patient does not agree to respond, say thanks to them for giving you the time.
- Please introduce each question separately and record the response.
- The elicited information will be treated as confidential and don't discuss or share the responses with any third/outside person.

1. **ID**	
**1 Type of Health Facility**	
1.2 **Designation:**	
**2. 2. General**	
**2.1**	How long have you been working in this health facility? (months/years)
**2.2**	Total months/years of service
**2.3**	What are your current roles and responsibility with respect to maternal and neonatal care?
**2.4**	How many deliveries and resuscitations of newborns have you attended in last 1 month?

**2.4**	A. No. of deliveries attended in last 1 month	
	B. No. of newborn resuscitations attended in last 1 month	

**2.5**	Who did you receive the training from:			
	Area		Training name	**Year**
	A	Care during delivery (S.B.A.)		
	B	Neonatal Resuscitation Program		
	C	Sick Newborn Care (FBNC)		

3. Service Delivery

**3.1**	What are the challenges faced by you and your colleagues for delivering the desired mother and newborn care services?		
	**Challenges faced**		
		**Mother care**	**Newborn care**
	Infrastructure		
	Equipment		
	Drugs and supplies		
	Support services		
	Other		

**3.2**	What challenges do you face while delivering essential newborn care services and how do you manage these?	
	**Challenges faced**	**How do you manage these challenges**
	Care at delivery	
	Care in the ward	
	Care of sick newborns	

**3.3**	What challenges do you face while delivery of pregnant women?	
	**Challenges faced**	**How do you manage these challenges**
	Delivery without complication	
	Delivery with complication	
	Caesarean section	
	Referred cases with complication	

**3.4**	How long usually the mothers stay at the facility after the delivery?	
	Normal Delivery	
	Caesarean Delivery	
**4. Manpower**		
**4.1**	How many posts of Staff Nurse / ANM are vacant in your health facility?	
**4.2**	What difficulties do you face in providing mother and newborn care services to existing employees? (Doctors, nurses and other staff)	
**4.3**	What is the mechanism of taking leave and who sanctions it?	
**5. Duty Roster**		
**5.1**	Who prepares the duty roster for you?	
**5.2**	Do you have flexibility in changing the shifts?	
**5.3**	How do you manage when you have double shifts?	
**5.4**	What is the procedure for taking leave and who approves it?	
**5.5**	Who prepares rosters for emergency / regular service?	
**6. Infrastructure**		
**6.1**	Do you have space to accommodate changes inside the department?	
**6.2**	Do you have enough beds to accommodate increased number of patients?	
**6.3**	Is their regular power supply and clean water for drinking? Any substitute available in case of power cut or irregular water supply?	
**7. Data management**		
**7.1**	How do you record data?	
**7.2**	How do you maintain a register?	
**7.3**	Where do you send the record?	
**7.4**	How often is the data sent?	
**8. Blood bank**		
**8.1**	How long does it take for a needy person to get blood?	
**9. Training /Skills**		
**9.1**	Could you tell us about your previous NSSK / Neonatal Resuscitation Training?	
	Time (month / year)	
	place	
	Duration (in days)	
**9.2**	Who conducts the workshop? Who prepares roster for workshop/training and how it is notified? How it is monitored?	
**9.3**	What did you like the most in the training?	
**9.4**	What did you dislike the most in the training?	
**9.5**	What was the training methodology used (Lectures/ Hands-on / Practical’s)?	
**9.6**	How did you like meeting / interacting with the trainers?	

.

**9.7**	What was their level of knowledge / skills?	
	How was your conversation with him?	
**9.8**	What are the opportunities and mechanisms currently in place/adopted to retain the skills of Nurses/ANMs/Doctors?	
**9.9**	What challenges do you have with the skills of nurses and the support of staff in delivery rooms, perinatal wards and newborn care units? In your opinion, how can this be controlled?	
**9.10**	How the training related to care during delivery and newborn period can be further improved?	
**9.11**	Have you been to a skill lab set up in your district?	
**9.12**	What are the good things about this skill lab?	
**9.13**	What are the challenges related to skill lab?	
**9.14**	In your opinion, how many health staffs might have used or visited the skill labs?	
**9.15**	Did somebody advise or persuade you to attend the skill lab?	
**9.16**	How does the Skill Lab help in Neonatal Resuscitation and Neonatal Care?	
**10. Referral services**		
**10.1**	In what situation usually the newborns/mothers (pregnant/recently delivered) are referred to the next level of healthcare?	
**10.2**	Where the newborns/ pregnant women/ mothers are usually referred, what is the usual mode of transportation and how long does it takes to reach the next level health facility in your area?	
**10.3**	What facilitation is done from facility side for referral and what difficulties/challenges do you face while transporting the sick newborn and mother to next level? *(Probe: monetary/logistics)*	
**10.4**	What are the challenges faced related to referral transport experienced by this facility and how are they handled?	
**11. Logistics**		
**11.1**	Are you familiar with any scarcity / irregular supply of medicines and / or supplies required for care during delivery and newborn in the last one year? What were the reasons for this deficiency and how were these conditions managed?	
**11.2**	How frequently the families/ parents asked to procure drugs from outside/ store?	
**11.3**	What are the supervisory mechanisms in place at present for maternal and newborn care services?	
	Who supervises	
	What is the frequency of supervisory visits	
	Is any feedback/report provided usually after the supervision?	
	What actions are taken after last supervisory visit?	
**11.4**	Please let us know about the last supervisory visit to the facility related to maternal and newborn care services?	
	Who came for last supervisory visit?	
	How long ago the supervisory visit took place?	
	What all components were observed?	
	What feedback was given and what actions were taken?	
**12. others**		
**12.1**	In your view, what are the perceived barriers between families in the use of public health	

	services for newborns of pregnant women?
**12.2**	In your view, what are the perceived barriers between families in the use of public health services for newborns of pregnant women?
**12.3**	How much additional effort is needed to reduce the neonatal mortality rate in your area?
**12.4**	According to you, what is the meaning of quality?
**12.5**	What can you do to improve the quality of the health services?
**12.6**	What can you do to improve the quality of the health services?
**12.7**	Did any of your relatives, friends or acquaintances ever availed health services at this hospital? If not, any reason?

Thank the respondent.

Name of Interviewer______________________ Signature________________

.

UNIQUE ID:

Improving Quality of Care for Mother and Newborn at District Hospitals and FRU's of 3 Districts of Haryana

In Depth Interview – Class IV Worker (Baseline)

**District:**	**Block**
**Name of the Facility:**	
**Date of Interview/ Observation**	☐☐☐☐☐☐☐☐
**Start time**	☐☐ ☐☐ (HH: MM)
**End time**	☐☐ ☐☐ (HH: MM)

Guidelines for interviewers

- Greet the patient and introduce yourself.
- Explain the patient why you are taking up this questionnaire.
- Take the consent and then start the questions.
- If you do not get satisfactory response, promptly please repeat the question and probe wherever necessary.
- If a patient does not agree to respond, say thanks to them for giving you the time.
- Please introduce each question separately and record the response.
- The elicited information will be treated as confidential and don't discuss or share the responses with any third/outside person.

S.No.	Questions
**1.**	**General Information**
1.1	Are you a staff of this hospital or hired on contract?
1.2	Are you provided with residence from hospital?
1.3	How many class IV worker are posted here in this hospital?
**2.**	**Specific Information**
2.1	Please tell us in details about the duty shifts and person present in each shift?
2.2	Do you have responsibility of just one department or all the departments of this hospital?
2.3	Tell us about your job responsibilities.
2.4	Do you have duty at night shift?
2.5	Tell us about the places you clean in this hospital?
2.6	Tell us how frequently you clean the premises?
2.7	What media you use for cleaning different things?
2.8	Does anyone monitors your work?
**3.**	**Disposal**
3.1	How to you dispose the waste?
3.2	Do you follow color coding system for waste disposal? Please tell us elaborately
3.3	Tell us if there is a pick up van facility for garbage?
3.4	If yes, how frequently this van visits the hospital in a week?
3.5	If you burn any waste or garbage? If yes, tell us where do you do that?
**4.**	**Issues/Suggestions**
4.1	Tell us about the behaviour of staff with you
4.2	Tell us about the behaviour of patients with you.
4.3	Tell us about the difficulties you face in your duty.
4.4	What kind of changes you suggest for better service delivery?

Thank the respondent.

Name of Interviewer______________________ Signature________________

UNIQUE ID:

Improving Quality of Care for Mother and Newborn at District Hospitals and FRU's of 3 Districts of Haryana

In Depth Interview (End line)
Medical Officer

**District:**	**Block:**
**Name of the Facility:**	
**Date of Interview/ Observation**	☐☐☐☐☐☐☐☐
**Start time**	☐☐ ☐☐ (HH: MM)
**End time**	☐☐ ☐☐ (HH: MM)

Guidelines for interviewers

- Greet the patient and introduce yourself.
- Explain the patient why you are taking up this questionnaire.
- Take the consent and then start the questions.
- If you do not get satisfactory response, promptly please repeat the question and probe wherever necessary.
- If a patient does not agree to respond, say thanks to them for giving you the time.
- Please introduce each question separately and record the response.
- The elicited information will be treated as confidential and don't discuss or share the responses with any third/outside person.

1. **Government Health Facility:(Please tick ✓ the appropriate health facility and qualification)**			
**1.1 Type of Health Facility**		**1.2 Respondent Qualification**	
District Hospital	☐	MBBS	☐
CHC	☐	MD/ MS	☐
Block PHC	☐	BAMS/BHMS	☐
24x7 PHC	☐	**1.3 Designation in**	
PHC	☐	MO I/C	☐
Dispensary	☐	Medical Officer	☐
Sub-center	☐	Specialist (Pediatrician, Gynecologist)	☐
Any other; (Specify…………….)	☐	AYUSH doctor	☐
		Others(Specify)	☐

**2. General**		
**2.1**	How long have you been working in this health facility? (months/years)	
**2.2**	Total months/years of service	
**2.3**	What are your current roles and responsibility with respect to maternal and neonatal care?	
**3**	What do you mean by quality in the care provided by you at this hospital?	
**4**	In your opinion what all have been done to improve the quality of care?	
**5**	What do you know about the quality of care project undertaken/ implemented at your hospital?	
**6**	What all changes did you see at the hospital?	
**7**	What all skill building efforts were done at this facility/ hospital? How that helped you?	
**8**	What all changes do mean see in the record keeping /case sheet maintenance for the patients?	
**9**	What all changes do you see in the disinfection practices in your unit?	
**10**	In your opinion how these changes would help the patients and you?	
**11**	What more could have done to improve the quality of care for patients at this hospital?	
**12**	Has there been any change in the supervision and monitoring at this hospital?	
**13**	How have the quality of care project team helped you and other members/ staffs at this hospital?	
**14**	What all challenges did you experience with the quality of care project team?	
**15**	After the quality of care project finishes how the quality improvement efforts can be continued?	
**16**	Any other comments	

Name of Interviewer______________________ Signature________________

**UNIQUE ID:**

Improving Quality of Care for Mother and Newborn at District Hospitals and FRU's of 3 Districts of Haryana

In Depth Interview (End line)
Nurse

**District:**	**Block:**
**Name of the Facility:**	
**Date of Interview/ Observation**	☐☐☐☐☐☐☐☐
**Start time**	☐☐ ☐☐ (HH: MM)
**End time**	☐☐ ☐☐ (HH: MM)

Guidelines for interviewers

- Greet the patient and introduce yourself.
- Explain the patient why you are taking up this questionnaire.
- Take the consent and then start the questions.
- If you do not get satisfactory response, promptly please repeat the question and probe wherever necessary.
- If a patient does not agree to respond, say thanks to them for giving you the time.
- Please introduce each question separately and record the response.
- The elicited information will be treated as confidential and don't discuss or share the responses with any third/outside person.

1. **Government Health Facility:(Please tick ✓ the appropriate health facility and qualification)**			
**1.1 Type of Health Facility**		**1.2 Respondent Qualification**	
District Hospital	☐	MBBS	☐
CHC	☐	MD/ MS	☐
Block PHC	☐	BAMS/BHMS	☐
24x7 PHC	☐	**1.3 Designation in**	
PHC	☐	MO I/C	☐
Dispensary	☐	Medical Officer	☐
Sub-center	☐	Specialist (Pediatrician, Gynecologist)	☐
Any other; (Specify…………….)	☐	AYUSH doctor	☐
		Others(Specify)	☐

**2. General**		
**2.1**	How long have you been working in this health facility? (months/years)	
**2.2**	Total months/years of service	
**2.3**	What are your current roles and responsibility with respect to maternal and neonatal care?	
**3**	What do you mean by quality in the care provided by you at this hospital?	
**4**	In your opinion what all have been done to improve the quality of care?	
**5**	What do you know about the quality of care project undertaken/ implemented at your hospital?	
**6**	What all changes did you see at the hospital?	
**7**	What all skill building efforts were done at this facility/ hospital? How that helped you?	
**8**	What all changes do mean see in the record keeping /case sheet maintenance for the patients?	
**9**	What all changes do you see in the disinfection practices in your unit?	
**10**	In your opinion how these changes would help the patients and you?	
**11**	What more could have done to improve the quality of care for patients at this hospital?	
**12**	Has there been any change in the supervision and monitoring at this hospital?	
**13**	How have the quality of care project team helped you and other members/ staffs at this hospital?	
**14**	What all challenges did you experience with the quality of care project team?	
**15**	After the quality of care project finishes how the quality improvement efforts can be continued?	
**16**	Any other comments	

Name of Interviewer______________________ Signature________________

UNIQUE ID:

Improving Quality of Care for Mother and Newborn at District Hospitals and FRU's of 3 Districts of Haryana

In Depth Interview (End line)
Class IV Worker

**District:**	**Block:**
**Name of the Facility:**	
**Date of Interview/ Observation**	☐☐☐☐☐☐☐☐
**Start time**	☐☐ ☐☐ (HH: MM)
**End time**	☐☐ ☐☐ (HH: MM)

Guidelines for interviewers

- Greet the patient and introduce yourself.
- Explain the patient why you are taking up this questionnaire.
- Take the consent and then start the questions.
- If you do not get satisfactory response, promptly please repeat the question and probe wherever necessary.
- If a patient does not agree to respond, say thanks to them for giving you the time.
- Please introduce each question separately and record the response.
- The elicited information will be treated as confidential and don't discuss or share the responses with any third/outside person.

1. **Government Health Facility:(Please tick ✓ the appropriate health facility and qualification)**			
**1.1 Type of Health Facility**		**1.2 Respondent Qualification**	
District Hospital	☐	MBBS	☐
CHC	☐	MD/ MS	☐
Block PHC	☐	BAMS/BHMS	☐
24x7 PHC	☐	**1.3 Designation in**	
PHC	☐	MO I/C	☐
Dispensary	☐	Medical Officer	☐
Sub-center	☐	Specialist (Pediatrician, Gynecologist)	☐
Any other; (Specify…………….)	☐	AYUSH doctor	☐
		Others(Specify)	☐

**2. General**		
**2.1**	How long have you been working in this health facility? (months/years)	
**2.2**	Total months/years of service	
**2.3**	What are your current roles and responsibility with respect to maternal and neonatal care?	
**3**	What do you mean by quality in the care provided by you at this hospital?	
**4**	In your opinion what all have been done to improve the quality of care?	
**5**	What do you know about the quality of care project undertaken/ implemented at your hospital?	
**6**	What all changes did you see at the hospital?	
**7**	What all skill building efforts were done at this facility/ hospital? How that helped you?	
**8**	What all changes do mean see in the record keeping /case sheet maintenance for the patients?	
**9**	What all changes do you see in the disinfection practices in your unit?	
**10**	In your opinion how these changes would help the patients and you?	
**11**	What more could have done to improve the quality of care for patients at this hospital?	
**12**	Has there been any change in the supervision and monitoring at this hospital?	
**13**	How have the quality of care project team helped you and other members/ staffs at this hospital?	
**14**	What all challenges did you experience with the quality of care project team?	
**15**	After the quality of care project finishes how the quality improvement efforts can be continued?	
**16**	Any other comments	

Name of Interviewer______________________ Signature________________

UNIQUE ID:

Improving Quality of Care for Mother and Newborn at District Hospitals and FRU's of 3 Districts of Haryana

In Depth Interview (End line)
Quality Manager

District:	Block:
Name of the Facility:	
Date of Interview/ Observation	☐☐☐☐☐☐☐☐
Start time	☐☐ ☐☐ (HH: MM)
End time	☐☐ ☐☐ (HH: MM)

Guidelines for interviewers

- Greet the patient and introduce yourself.
- Explain the patient why you are taking up this questionnaire.
- Take the consent and then start the questions.
- If you do not get satisfactory response, promptly please repeat the question and probe wherever necessary.
- If a patient does not agree to respond, say thanks to them for giving you the time.
- Please introduce each question separately and record the response.
- The elicited information will be treated as confidential and don't discuss or share the responses with any third/outside person.

1. **Government Health Facility:(Please tick ✓ the appropriate health facility and qualification)**			
**1.1 Type of Health Facility**		**1.2 Respondent Qualification**	
District Hospital	☐	MBBS	☐
CHC	☐	MD/ MS	☐
Block PHC	☐	BAMS/BHMS	☐
24x7 PHC	☐	**1.3 Designation in**	
PHC	☐	MO I/C	☐
Dispensary	☐	Medical Officer	☐
Sub-center	☐	Specialist (Pediatrician, Gynecologist)	☐
Any other; (Specify…………….)	☐	AYUSH doctor	☐
		Others(Specify)	☐

**2. General**		
**2.1**	How long have you been working in this health facility? (months/years)	
**2.2**	Total months/years of service	
**2.3**	What are your current roles and responsibility with respect to maternal and neonatal care?	
**3**	What do you mean by quality in the care provided by you at this hospital?	
**4**	In your opinion what all have been done to improve the quality of care?	
**5**	What do you know about the quality of care project undertaken/ implemented at your hospital?	
**6**	What all changes did you see at the hospital?	
**7**	What all skill building efforts were done at this facility/ hospital? How that helped you?	
**8**	What all changes do mean see in the record keeping /case sheet maintenance for the patients?	
**9**	What all changes do you see in the disinfection practices in your unit?	
**10**	In your opinion how these changes would help the patients and you?	
**11**	What more could have done to improve the quality of care for patients at this hospital?	
**12**	Has there been any change in the supervision and monitoring at this hospital?	
**13**	How have the quality of care project team helped you and other members/ staffs at this hospital?	
**14**	What all challenges did you experience with the quality of care project team?	
**15**	After the quality of care project finishes how the quality improvement efforts can be continued?	
**16**	Any other comments	

Name of Interviewer______________________ Signature________________
