# Supplementary material for: Improving quality of care for pregnancy, perinatal and newborn care at district and sub-district public health facilities in three districts of Haryana, India: An Implementation study
Source: PLoS One. 2021 Jul 23;16(7):e0254781. doi: 10.1371/journal.pone.0254781 (PMC8301676; doi:10.1371/journal.pone.0254781)
Supplement: S1 Appendix — (DOCX) [file pone.0254781.s021.docx]

**Title: Improving quality of care for pregnancy, perinatal and newborn care at district and sub-district public health facilities in three districts of Haryana, India: An Implementation study**

**S1 Appendix: Supplementary information**

**Index**

| Sl.no | Document description | Page no. |
| --- | --- | --- |
| 1 | Table S1: Organisation of the public health system in India and the maternal and newborn services | 2 |
| 2 | Table S2: Demographic and health profile of Haryana and study districts | 4 |
| 3 | Figure S1: Theory of change and logic model for the Quality Improvement in maternal and newborn care | 6 |
| 3 | Table S3: Quality management teams at the facilities in the districts and their compositions | 7 |
| 4 | Table S4: List of topics for self-learning and facilitated learning during weekly meetings | 9 |
| 5 | Table S5: Outcome Indicators for the impact of quality improvement and frequency of data collection | 11 |
| 6 | Table S6: Data collected related to the quality improvement interventions in the study districts | 13 |
| 7 | Table S7: The quality gaps identified and resolved during the intervention period for each district | 14 |
| 8 | Table S8: Key quality gaps observed at the facilities in the districts during formative research phase | 16 |
| 9 | Table S9: Changes in the infrastructure, manpower and processes at the hospitals in the three districts | 19 |
| 10 | Table S10: Changes in the quality of case record documentation at the hospitals in the three districts | 20 |
| 11 | Table S11: Changes in the patient satisfaction status at the hospitals in the three districts | 21 |
| 12 | Table S12: Time spent (median and IQR in minutes) in minutes by pregnant women in antenatal clinics in Faridabad district | 23 |
| 13 | Table S13: Change in knowledge and skill status of the care providers in labour room and sick newborn care units | 24 |

Table S1: Organisation of the public health system in India and the maternal and newborn services^(1-3)^

| Facility ther referral hospitalsof diseases  tion lements | Facilities and services related to maternal and newborn care |
| --- | --- |
| Tertiary care facilities (Medical colleges and other referral hospital) | - Comprehensive and advanced care for all types of diseases - Variable bed strength, doctor (including specialists and super-specialists) and paramedical staffs - Comprehensive pregnancy and childbirth care, comprehensive newborn care including critical care, child health, immunization, nutrition, family planning, adolescent health, care for communicable diseases, non-communicable diseases, mental health, cancer, oral health, elderly care and 24x7 emergency care - Referral for multiple districts and states |
| District hospital (DH)* | - 1,000,000-6,000,000 population - Outpatients and in-patient services (100-500 beds), labour room, operation theatres, laboratory, radiology, blood bank and ambulance - 32-68 doctors (19-40 specialists including >1 obstetrician and paediatrician) and 76-325 nursing and paramedical staffs - Pregnancy and childbirth (including caesarean section), postnatal care, essential newborn care, newborn illness care (SNCU 12-24 beds), child health, immunization and nutrition, family planning, adolescent health, communicable disease care and surveillance, non-communicable disease, mental health, oral health program implementation, elderly care and 24x7 emergency care - Referral, supervision and monitoring for the whole district |
| Sub-district hospital (SDH)* | - 500,000-600,000 population - Outpatients and in-patient services (31-100 beds), labour room, operation theatre, laboratory, radiology facility, blood bank/storage and ambulance - 20-30 doctors (10 specialists including obstetrician and paediatrician) and 45-75 nursing and paramedical staffs - Pregnancy and childbirth (including caesarean section), postnatal care, essential newborn care, newborn illness care (SNCU 6-12 beds), child health, immunization and nutrition, family planning, adolescent health, communicable disease care and surveillance, non-communicable disease, mental health, oral health program implementation, elderly care and 24x7 emergency care - First referral unit for multiple PHCs and CHCs |
| First referral unit (FRU)* | - 120,000 population (80,000 in hilly/tribal areas) - Outpatients and in-patient services (30 beds), labour room, operation theatre, laboratory, x-ray facility, blood storage and ambulance - 11 doctors (4-5 specialists including obstetrician and paediatrician) and 35-41 nursing and paramedical staffs - Pregnancy and childbirth (including caesarean section), postnatal care, essential newborn care, newborn illness care (NBSU), child health, immunization and nutrition, family planning, adolescent health, communicable disease care and surveillance, non-communicable disease, mental health, oral health program implementation, elderly care and 24x7 emergency care - Referral, supervision and monitoring of 4-5 PHCs |
| Community Health Centre (CHC) | - 120,000 population (80,000 in hilly/tribal areas) - Outpatients and in-patient services (30 beds) with labour room, operation theatre, laboratory, x-ray facility, blood storage (may) and ambulance - 11 doctors (4-5 specialists including obstetrician and paediatrician) and 35-41 nursing and paramedical staffs - Pregnancy and childbirth (including caesarean section), postnatal care, essential newborn care, newborn illness detection and referral, child health, immunization and nutrition, family planning, adolescent health, communicable disease care and surveillance, non-communicable disease, mental health, oral health program implementation, elderly care and emergency care (24 hours) - Referral, supervision and monitoring of 4-5 PHCs |
| Primary Health Centre (PHC) | - 30,000-50,000 population (20,000-25,000 in hilly/tribal areas) - Outpatients and in-patient services (6 beds) with labour room, laboratory, x-ray facility and ambulance - 1-3 doctors and 12-19 nursing and paramedical staffs - Pregnancy and childbirth (including assisted deliveries), postnatal care, essential newborn care, illness detection and referral, child health, immunization and nutrition, family planning, adolescent health, communicable disease care and surveillance, non-communicable disease, mental health, oral health program implementation, elderly care and emergency care (24 hours) - Supervision and monitoring of 5 Sub health centres |
| Health and Wellness Centre (HWC)^(2)^ | - 5000-20000 population - Outpatients and outreach services (no inpatient service) - 2-3 MPW for rural and one MPW per 10000 population (urban) - Pregnancy and childbirth, essential newborn care, illness detection and referral, child health, immunization and nutrition, family planning, adolescent health, communicable disease care and surveillance, non-communicable disease, mental health and oral health program implementation, elderly care and emergency care - Supervision and monitoring of 4-5 ASHAs by each MPW |
| Sub Health Centre (SCH) | - One per 5000 populations (3000 in hilly/tribal/desert area) - Outpatients and outreach services (no inpatient service) - 1-2 Auxillary Nurse and Mid-wife (ANM) - Antenatal care, deliveries (if needed), essential newborn care, immunization, illness detection and referral, family planning, adolescent health, disease surveillance and control of epidemics and non-communicable disease program implementation - Supervision and monitoring of 5 ASHAs |
| Community level | - 1 ASHA per 1000 (rural) or 2500 (urban) population - Mobilize pregnant women for antenatal care and institutional delivery, home visits for newborn care, mobilise for immunization, assist in referral, dispensing drugs, contraception and supplements |

*Note: * The facilities included in the current study were FRUs, SDH and DHs.*

*ASHA: Accredited Social Health Activist; MPW: Multipurpose worker; NBSU: Newborn stabilisation unit; SNCU: Sick newborn care unit-NBSU,*

**References**

1. Ministry of Health and Family Welfare, Government of India. Indian Public Health Standards (IPHS) for Sub-centres, Primary Health Centres (PHCs), Community Health Centres (CHCs), Sub-District and District Hospitals. 2012. Available from: <https://nhm.gov.in/index1.php?lang=1&level=2&sublinkid=971&lid=154#:~:text=IPHS%20are%20a%20set%20of,especially%20for%20Non%2DCommunicable%20Diseases>.
2. Ministry of Health and Family Welfare. Ayushman Bharat. Comprehensive Primary Health Care through Health and Wellness Centers- Operational Guidelines. Government of India; 2018. Available from: <https://ab-hwc.nhp.gov.in/download/document/45a4ab64b74ab124cfd853ec9a0127e4.pdf>
3. Ministry of Health and Family Welfare, Government of India. Infrastructure. Health System Strengthening, National Health Mission. Available from: <https://nhm.gov.in/index1.php?lang=1&level=2&sublinkid=1220&lid=190>

Table S2: Demographic and health profile of Haryana and study districts

|  | Haryana | | | | India |
| --- | --- | --- | --- | --- | --- |
| Parameters | Faridabad | Rewari | Jhajjar | State |  |
| *Population demography* |  |  |  |  |  |
| Total population, millions (2011)^a^ | 1.8 | 0.90 | 0.95 | 25.35 | 1,210 |
| Population density (2011)^a^ | 2442 | 565 | 523 | 573 | 382 |
| Rural population (%) (2011)^a^ | 20.5 | 74 | 74.6 | 651. | 68.8 |
| Scheduled caste population (%) (2011)^a^ | 12.3 | 20 | 17.8 | 19.3 | 16.6 |
| Female literacy (%) (2011)^a^ | 73.8 | 69.6 | 70.7 | 65.9 | 65.5 |
| *Socioeconomic indicator* |  |  |  |  |  |
| Per capita income, Indian Rupees/ US$* (2017-18)^b^ | - | - |  | 178,890 /2702 | 103,870 /1569 |
| Below poverty line population (%) (2011-12)^c^ | - | - |  | 11.16 | 21.92 |
| *Maternal and child health indicators* |  |  |  |  |  |
| Pregnant women with full antenatal check-ups (%) (2015-16)^d^ | 49.3 | 45.7 | 28.5 | 49.3 | 66.4 |
| Pregnant women protected against tetanus (%) (2015-16)^d^ | 93 | 91.2 | 80.8 | 93.0 | 89.9 |
| Institutional delivery (%) (2015-16)^d^ | 80.6 | 90.2 | 90.6 | 80.6 | 88.7 |
| Institutional delivery at public health facilities (%) (2015-16)^d^ | 46.8 | 69.2 | 50.7 | 46.3 | 46.2 |
| Caesarean section deliveries (%) (2015-16) ^d^ | 13.6 | 12.8 | 14.5 | 13.6 | 28.2 |
| Children breastfed within one hour (%) (2015-16)^d^ | 38.3 | 41.2 | 36.7 | 38.3 | 42.8 |
| Infant mortality rate (2017)^e^ | - | - | - | 30 | 33 |
| Neonatal mortality rate (2017)^f^ | - | - | - | 22 | 23.7 |
| Maternal mortality ratio (2015-17)^g^ | - | - | - | 98 | 122 |
| *Public Health Facilities* |  |  |  |  |  |
| Community Health Centres ^h^ | 4 | 5 | 6 | 128 | 5,624 |
| First Referral Units ^h^ | 2 | 2 | 2 | 20 | 3,057 |
| Primary Health Centres ^h^ | 16 | 21 | 27 | 529 | 25,743 |
| Sub Health Centres ^h^ | 58 | 112 | 126 | 2,650 | 158,417 |

*Notes: a Census of India 2011, Registrar General of India* (1)

*b Economic Survey of Haryana (2017-18)* (2)

*c Reserve Bank of India (2011-12)* (3)

*d National Family Health Survey, India (2015-16)* (4)

*e SRS Bulletin (2017), Sample Registration System, Registrar General, India* (5)

*f^^^ UN Inter-agency Group for Child Mortality Estimation (*[*www.childmortality.org*](http://www.childmortality.org)*.)*(6)

*g Special Bulletin on Maternal Mortality in India (2015-17), Registrar General, India* (7)

*h Infrastructure, Health Department, Government of Haryana* (8)

** Average US $ to INR 66.2 for 2017-18*

*Population density: number of persons per square kilometre area*

*Infant mortality rate per 1000 live births; Maternal mortality ratio- number of maternal deaths per 100000 live births; Neonatal mortality rate per 1000 live births*

**References**

1. Registrar General of India. Census of India 2011 [Internet]. Government of India; [cited 2020 Mar 28]. Available from: http://censusindia.gov.in/2011-Common/CensusData2011.html

2. Department of Economic and Statistical Analysis, Haryana. Economic Survey of Haryana (2017-18) [Internet]. Government of Haryana; 2018 [cited 2020 Mar 28]. Available from: http://esaharyana.gov.in/Portals/0/ES%202017-18%20English.pdf

3. Reserve Bank of India. Table 162 : Number and Percentage of Population Below Poverty Line [Internet]. Reserve Bank of India; 2017 [cited 2020 Mar 28]. Available from: https://www.rbi.org.in/scripts/PublicationsView.aspx?id=17937

4. International Institute for Population Sciences. Fact Sheets for Key Indicators, National Family Health Survey-4, India (2015-16) [Internet]. International Institute for Population Sciences; [cited 2020 Mar 28]. Available from: http://rchiips.org/NFHS/factsheet_NFHS-4.shtml

5. Registrar General of India. SRS Bulletin. Sample Registration System, 2017, Vol. 52, No.1 [Internet]. Registrar General of India, Government of India; 2917. Available from: http://censusindia.gov.in/vital_statistics/SRS_Bulletins/SRS_Bulletin-Rate-2017-_May_2019.pdf

6. UN Inter-agency Group for Child Mortality Estimation. Estimates Developed by the UN Inter-agency Group for Child Mortality Estimation (UNICEF, WHO, World Bank, UN DESA Population Division) [Internet]. [cited 2020 Mar 21]. Available from: https://data.worldbank.org/indicator/SH.DYN.NMRT?locations=IN

7. Registrar General of India. Special Bulletin on Maternal Mortality in India 2015-17, Sample Registration System November 2019 [Internet]. Registrar General of India, Government of India; 2019. Available from: http://censusindia.gov.in/vital_statistics/SRS_Bulletins/MMR_Bulletin-2015-17.pdf

8. Department of Health and Family Welfare. Infrastructure, Health Department, Government of [Internet]. Government of Haryana; [cited 2020 Mar 28]. Available from: http://haryanahealth.nic.in/Infrastructure.html

Figure S1: Theory of change and logic model for improving quality of care for the mothers and newborns


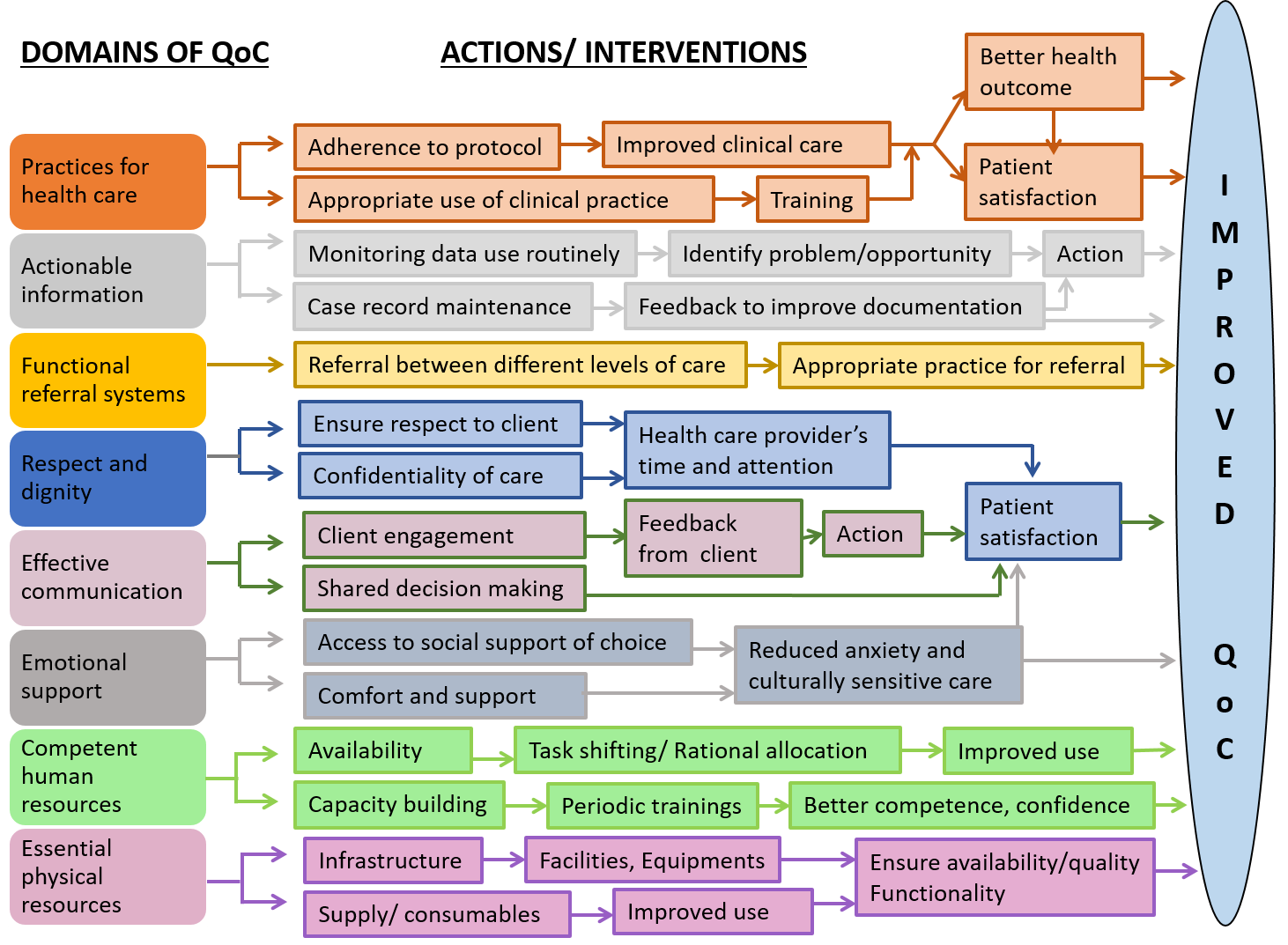


*Reference: Das MK, Arora NK, Dalpath S, Kumar S, Qazi SA, Bahl R. Improving quality of care for perinatal and newborn care at district and subdistrict hospitals in Haryana, India: Implementation research protocol. J Adv Nurs. 2018 Dec; 74(12):2904–11.*

Table S3: Quality management (QM) teams at the facilities in the districts and their compositions

| Members Designation | No. | Members Designation | No. |
| --- | --- | --- | --- |
| *1. Faridabad district* | | | |
| *1.1 Faridabad District QM Committee (n=10)* | | | |
| Chief Medical Officer  Medical Superintendent of DH  Medical Officer In-charge FRU 1  Medical Officer In-charge FRU 2  External QI Project Investigator | 1  1  1  1  1 | RCH Officer  District Quality Monitor  HOD- Obstetrics  HOD-Pediatrics  State Child Health Consultant | 1  1  1  1  1 |
| *1.2. Faridabad District Hospital QM Committee (n=12)* | | | |
| Principal Medical Officer  Hospital Administration Medical Officers  District Quality Monitor  External QI Project Investigator  External QI Project District Coordinator | 1  2  1  1  1 | Surgery Specialist  HOD-Obstetrics  HOD-Pediatrics  Nursing Superintendent  Staff Nurse (LR)- Quality & Infection Control | 1  1  1  1  1  1 |
| *1.3. Faridabad FRU 1 QM Committee (n=7)* | | | |
| Medical Officer in-charge & Obstetrician  Paediatrician  Dental Surgeon  External QI Project District Coordinator | 1  1  1  1 | Nursing In-charge  Pharmacist  Lab Technician | 1  1  1 |
| *1.4 Faridabad FRU 2 QM Committee (n=7)* | | | |
| Medical Officer in-charge  Obstetrician  Paediatrician  External QI Project District Coordinator | 1  1  1  1 | Nursing In-charge  Pharmacist  Lab Technician | 1  1  1 |
| *2. Rewari district* | | | |
| *2.1 Rewari District QM Committee (n=10)* | | | |
| Chief Medical Officer  Medical Superintendent of DH  Medical Officer In-charge FRU 1  Medical Officer In-charge FRU 2  External QI Project Investigator | 1  1  1  1  1 | RCH Officer  District Quality Monitor  Obstetrician  Paediatrician  State Child Health Consultant | 1  1  1  1  1 |
| *2.2. Rewari District Hospital QM Committee (n=7)* | | | |
| Medical Superintendent  District Quality Monitor  External QI Project Investigator  External QI Project District Coordinator | 1  1  1  1 | HOD-Obstetrics  HOD-Pediatrics  Nursing Superintendent | 1  1  1 |
| *2.3. Rewari FRU 1 QM Committee (n=5)* | | | |
| Medical Officer in-charge  External QI Project District Coordinator | 1  1 | Staff nurses  Pharmacist | 2  1 |
| *2.4. Rewari FRU 2 QM Committee (n=4)* | | | |
| Medical Officer in-charge  External QI Project District Coordinator | 1  1 | Staff nurse  Pharmacist | 1  1 |

| *3. Jhajjar district* | | | |
| --- | --- | --- | --- |
| *3.1 Jhajjar District QM Committee (n=10)* | | | |
| Chief Medical Officer  Medical Superintendent of DH  Medical Officer In-charge SDH  Medical Officer In-charge FRU 2  External QI Project Investigator | 1  1  1  1  1 | RCH Officer  District Quality Monitor  Obstetrician  Paediatrician  State Child Health Consultant | 1  1  1  1  1 |
| *3.2. Jhajjar District Hospital QM Committee (n=12)* | | | |
| Medical Superintendent  Medical Officer- Quality in-charge  District Quality Monitor  HOD-Obstetrics  External QI Project Investigator  External QI Project District Coordinator | 1  1  1  1  1  1 | HOD-Pediatrics  Nursing Superintendent  Nurse- Quality management  Nurse- infection control  Nurse in-charge- LR  Nurse in-charge- SNCU | 1  1  1  1  1  1 |
| *3.3. Jhajjar SDH QM Committee (n=12)* | | | |
| Medical Superintendent  Medical Officer- Hospital administration  Medical Officer- Quality in-charge  HOD-Obstetrics  External QI Project Investigator  External QI Project District Coordinator | 1  1  1  1  1  1 | HOD-Pediatrics  Nursing Superintendent  Nurse- Quality management  Nurse- infection control  Nurse in-charge- LR  Nurse in-charge- SNCU | 1  1  1  1  1  1 |
| *3.4. Jhajjar FRU 2 QM Committee (n=4)* | | | |
| Medical Officer in-charge  External QI Project District Coordinator | 1  1 | Medical officer  Staff nurse | 1  1 |

*Note: DH: District hospital; FRU: First referral unit; HOD: Head of department; LR: Labour room; QI: Quality improvement; QM: Quality management; RCH: Reproductive and child health; SDH: Sub-district hospital; SNCU: Sick newborn care unit;*

Table S4: List of topics for self-learning and facilitated learning during weekly meetings

| Sl no | Topic | Types of resource materials |
| --- | --- | --- |
| *1* | *Section 1. Essential newborn care* |  |
| 1.1 | Care of normal newborn at birth and postpartum period | Reading material, Job aid, Checklist, Video |
| 1.2 | Essential newborn care | Reading material, Job aid, Checklist |
| 1.3 | Hypothermia and thermal control | Reading material, Job aid |
| 1.4 | Breastfeeding | Reading material, Job aid, Checklist, Poster, Video |
| 1.5 | Neonatal Resuscitation | Reading material, Job aid, Video |
| *2* | *Section 2: Sick newborn care* |  |
| 2.1 | Emergency triage assessment and treatment | Reading material, Job aid, Checklist, Video |
| 2.2 | Management of low birth weight and preterm newborn | Reading material, Job aid |
|  | Hypothermia and Kangaroo mother care | Reading material, Job aid, Video |
| 2.3 | Neonatal jaundice | Reading material, Job aid |
| 2.4 | Management of hypoglycaemia | Reading material, Job aid, Video |
| 2.5 | Neonatal sepsis | Reading material, Job aid, Video |
| 2.6 | Neonatal shock and fluid management | Reading material, Job aid |
| 2.7 | Respiratory distress in newborn | Reading material, Job aid, Video |
| 2.8 | Neonatal seizures | Reading material, Job aid, Video |
| 2.9 | Post resuscitation management of an asphyxiated neonate | Reading material, Job aid |
| 2.10 | Anaemia & bleeding in neonates | Reading material |
| 2.11 | Fluid management | Reading material |
| 2.12 | Neonatal transport | Reading material |
| 2.13 | Follow up of high risk newborns | Reading material |
| 2.14 | Infection prevention and control in SNCU, hand Hygiene, disinfection | Reading materials, Job aid, Poster, Video |
| *3* | *Section 3: Obstetric care* |  |
| 3.1 | Normal labour and delivery: Part 1- stages, assessment and monitoring including partograph | Reading materials, Guidelines, Checklist, Poster |
| 3.2 | Normal labour and delivery: Part 2- normal delivery, empowering birth companion | Reading materials, Guidelines, Checklist, Poster |
| 3.3 | Active management of third stage of labour | Reading materials, Guidelines, Checklist, Poster |
| 3.4 | Preterm labour: identification and management | Reading materials, Guidelines, Poster |
| 3.5 | Prolonged labour: Identification and management | Reading materials, Guidelines, Checklist, Poster |
| 3.6 | Assisted delivery: Forceps and vacuum assisted delivery | Reading materials, Guidelines, Videos |
| 3.7 | Caesarean section: when to consider and care after CS delivery | Reading materials, Guidelines, Checklist |
| 3.8 | Newborn resuscitation | Reading materials, Guidelines, Videos |
| 3.9 | Infection prevention and control in LR, hand hygiene, disinfection | Reading materials, Job aid, Poster, Video |
| *4* | *Section 4: Postpartum care* |  |
| 4.1 | Postpartum care- Part 1: Mother- assessments of mother, care and monitoring | Reading materials, Guidelines, Checklist, Videos |
| 4.2 | Postpartum care- Part 2: Newborn- Assessments of newborn, temperature maintenance, family planning counselling, danger signs | Reading materials, Guidelines, Checklist, Videos |
| 4.3 | Postpartum care- Part 3: Supporting breastfeeding | Reading material, Job aid, Checklist, Poster, Video |
| 4.4 | Puerperal sepsis: Identification management and prevention | Reading materials, Guidelines, Checklist |
| 4.5 | Postpartum haemorrhage: identification and management | Reading materials, Guidelines, Checklist, Videos |
| 4.6 | Counselling of mother at discharge |  |
| *5* | *Section 5: Antenatal care* |  |
| 5.1 | ANC, EDD, abdominal examination, laboratory investigations, counselling, birth planning & screening for high risk pregnancy | Reading materials, Job aid, ANC card, Poster |
| 5.2 | PIH, detection, monitoring, management of eclampsia including Inj. MgSO_4_ | Reading materials, Job aid, ANC card, Poster |
| 5.3 | Gestational diabetes: Identification, management and counselling | Reading materials, Job aid |
| 5.4 | Anaemia: Identification, management and prevention | Reading materials, Job aid |
| 5.5 | Abortion and antepartum haemorrhage: Identification, management and counselling | Reading materials, Job aid |
| *6* | *Section 6: General* |  |
| 6.1 | Handwashing and infection control training for all support workers | Reading materials, Poster, Video |
| 6.2 | Biomedical waste management | Reading materials, Poster, Video |
| 6.3 | Equipment handling and maintenance demonstration | Demonstration and hands-on practice |
| 6.4 | Communication | Reading material, Job aid, Video |
| *7* | *Section 7: Quality Improvement* |  |
| 7.1 | Quality management system- Session 1: Principles and components | Reading material, Job aid, Discussion |
| 7.2 | Quality management system- Session 2: Documentation, monitoring and sustenance | Reading material, Job aid, Discussion |

*Note: ANC: Antenatal care; CS: Caesarean section; EDD: Expected date of delivery; LR: Labour room; MgSO_4_: Magnesium Sulfate; PIH: Pregnancy induced hypertension*

Table S5: Outcome Indicators for the impact of quality improvement and frequency of the data collection

| Sl.no. | Indicators | Data Source | Baseline | Quarterly | Endline |
| --- | --- | --- | --- | --- | --- |
|  | *Primary Outcome Indicators* |  |  |  |  |
| *1* | *Obstetric care (records)* |  |  |  |  |
| 1.1 | PIH cases detected (%) | CRR, Register review | X | X | X |
| 1.2 | MagSulf given to PIH cases (%) |  | X | X | X |
| 1.3 | Oxytocin given after delivery (%) |  | X | X | X |
| 1.4 | Prolonged labour cases (%) |  | X | X | X |
| 1.5 | Still births (%) |  | X | X | X |
| 1.6 | Severe systemic infection (%) |  | X | X | X |
| 1.7 | Maternal deaths (%) |  | X | X | X |
| *2* | *Newborn care at birth (observed)* |  |  |  |  |
| 2.1 | Immediate drying (%) | DO | X | X | X |
| 2.2 | Skin-to-skin contact (%) |  | X | X | X |
| 2.3 | Delayed cord clamping (%) |  | X | X | X |
| 2.4 | BF initiation (%) |  | X | X | X |
| *3* | *Antenatal care (observation)* |  |  |  |  |
| 3.1 | HRP cases identified (%) | CRR, DO | X | X | X |
| 3.2 | ANC counselling & birth planning (%) |  | X | X | X |
| *4* | *Sick newborn care (records)* |  |  |  |  |
| 4.1 | Total deaths (%) | Hospital data,  CRR | X | X | X |
| 4.2.1 | Deaths- weight >2500gms (%) |  | X | X | X |
| 4.2.2 | Deaths- weight <2500gms (%) |  | X | X | X |
| *5* | *Availability of services (review)* |  |  |  |  |
| 5.1 | Bag & mask, oxygen- LR/PNW/SNCUs (%) | DO, Stock register review | X | X | X |
| 5.2 | Medicines-obstetrics & neonates (%) |  | X | X | X |
| *6* | *Disinfection practice (observation)* |  |  |  |  |
| 6.1 | Soap & water in LR and SNCUs (%) | DO | X | X | X |
| 6.2 | Hand rub in LR and SNCU (%) |  | X | X | X |
| *7* | *Patient satisfaction (interview)* |  |  |  |  |
| 7.1 | Women who delivered (%) | Survey- mothers/  family | X | X | X |
| 7.2 | Women attending ANC (%) |  | X | X | X |
| 7.3 | Mothers of sick newborns (%) |  | X | X | X |
| *B* | *Secondary Outcome Indicators* |  |  |  |  |
| *8* | *Obstetric patient service delivery* |  |  |  |  |
| 8.1 | Total number of deliveries (n) | Hospital data,  CRR | X | X | X |
| 8.2 | Vaginal delivery (%) |  | X | X | X |
| 8.3 | Caesarean section (%) |  | X | X | X |
| 8.4 | Stillbirth (%) |  | X | X | X |
| 8.5 | Referred (%) |  | X | X | X |
| 8.6 | Antenatal clinical attendance (n) |  | X | X | X |
| 8.7 | High risk pregnancies (%) |  | X | X | X |
| *9* | *Newborn patient service delivery* |  |  |  |  |
| 9.1 | Total admissions (n) | Hospital data,  CRR | X | X | X |
| 9.2 | Inborn (%) |  | X | X | X |
| 9.3 | Discharge (%) |  | X | X | X |
| 9.4 | Referred/LAMA (%) |  | X | X | X |
| *10* | *Time spent in ANC facility* |  |  |  |  |
| 10.1 | ANC- time to 1^st^ contact with nurse (min) | DO | X | X | X |
| 10.2 | ANC- Total time spent (min) |  | X | X | X |
| *11* | *Time for response in LR* |  |  |  |  |
| 11.1 | Time to 1^st^ contact (min) |  |  |  |  |
| *12* | *Time for response in SNCU* |  |  |  |  |
| 12.1 | Time to 1^st^ contact (min) |  |  |  |  |

*Note: BL: Baseline; EL: Endline; LR: Labour room; PNW: Postnatal ward; ANC: Antenatal care; SNCU: Sick newborn care unit; BF: Breastfeeding; HRP: high risk pregnancy; PIH: Pregnancy induced hypertension; MagSulf: Magnesium Sulfate injection; LAMA: Left against medical advice; CRR: Case record review; DO: Direct observation*

Table S6: Data collected related to the quality improvement interventions in the study districts

| Sl.No | Data collected | Quality Improvement Cycles | | | | | | Total |
| --- | --- | --- | --- | --- | --- | --- | --- | --- |
|  |  | First | Second | Third | Fourth | Fifth | Sixth |  |
| A | Quantitative data collected |  |  |  |  |  |  |  |
| *1* | *Faridabad district* |  |  |  |  |  |  |  |
| 1.1 | Facility assessment (n)* | 3+12 | 3+12 | 3+12 | 3+12 | 3+12 | 3+12 | 18+72 |
| 1.2 | Case record review (n) | 666 | 921 | 857 | 544 | 620 | 637 | 4439 |
| 1.3 | Patient satisfaction status (n) | 401 | 665 | 649 | 422 | 678 | 483 | 3453 |
| 1.4 | Observation of care at birth (n) | 0 | 0 | 132 | 181 | 206 | 188 | 707 |
| 1.5 | Hand hygiene observations (n) | 0 | 0 | 1646 | 1911 | 1201 | 728 | 5486 |
| 1.6 | Patient flow analysis (n) | 171 | 226 | 227 | 185 | 188 | 157 | 1265 |
| 1.7 | Knowledge assessment (n) | 0 | 0 | 0 | 0 | 0 | 20 | 48 |
| *2* | *Rewari district* |  |  |  |  |  |  |  |
| 2.1 | Facility assessment (n)* | 3+12 | 3+12 | 3+12 | 3+12 | 3+12 | 3+12 | 18+72 |
| 2.2 | Case record review (n) | 719 | 811 | 735 | 742 | 785 | 908 | 4934 |
| 2.3 | Patient satisfaction status (n) | 399 | 597 | 519 | 439 | 746 | 590 | 3434 |
| 2.4 | Observation of care at birth (n) | 0 | 0 | 43 | 161 | 180 | 232 | 616 |
| 2.5 | Hand hygiene observations (n) | 0 | 0 | 630 | 1774 | 2200 | 2192 | 6796 |
| 2.6 | Patient flow analysis (n) | 120 | 130 | 117 | 98 | 96 | 98 | 735 |
| 2.7 | Knowledge assessment (n) | 0 | 0 | 0 | 0 | 0 | 32 | 58 |
| *3* | *Jhajjar district* |  |  |  |  |  |  |  |
| 3.1 | Facility assessment (n)* | 3+12 | 3+12 | 3+12 | 3+12 | 3+12 | 3+12 | 18+72 |
| 3.2 | Case record review (n) | 651 | 1057 | 877 | 759 | 770 | 861 | 5171 |
| 3.3 | Patient satisfaction status (n) | 330 | 353 | 354 | 377 | 395 | 424 | 2371 |
| 3.4 | Observation of care at birth (n) | 0 | 0 | 105 | 310 | 331 | 347 | 1093 |
| 3.5 | Hand hygiene observations (n) | 0 | 0 | 424 | 534 | 700 | 882 | 2540 |
| 3.6 | Patient flow analysis (n) | 151 | 138 | 150 | 129 | 141 | 141 | 953 |
| 3.7 | Knowledge assessment (n) | 0 | 0 | 0 | 0 | 0 | 22 | 43 |
| *4* | *Pooled (all three districts)* |  |  |  |  |  |  |  |
| 4.1 | Facility assessment (n)* | 9+36 | 9+36 | 9+36 | 9+36 | 9+36 | 9+36 | 54+216 |
| 4.2 | Case record review (n) | 2036 | 2789 | 2469 | 2045 | 2175 | 2406 | 14544 |
| 4.3 | Patient satisfaction status (n) | 1130 | 1615 | 1522 | 1238 | 1819 | 1497 | 9258 |
| 4.4 | Observation of care at birth (n) | 0 | 0 | 280 | 652 | 717 | 767 | 2416 |
| 4.5 | Hand hygiene observations (n) | 0 | 0 | 2700 | 4219 | 4101 | 3802 | 14822 |
| 4.6 | Patient flow analysis (n) | 442 | 494 | 494 | 412 | 425 | 396 | 2953 |
| 4.7 | Knowledge assessment (n) | 0 | 0 | 0 | 0 | 0 | 74 | 149 |
| B | Qualitative data collected |  |  |  |  |  |  |  |
| 5 | In-depth interviews |  |  |  |  |  |  |  |
| 5.1 | Faridabad | 28 | - | - | - | - | 27 | 55 |
| 5.2 | Rewari | 25 | - | - | - | - | 24 | 49 |
| 5.3 | Jhajjar | 29 | - | - | - | - | 28 | 57 |
|  | Pooled | 82 | - | - | - | - | 79 | 161 |

*Note: First cycle was the baseline period.*

** The facility assessments: n1+n2; n1: quarterly detailed assessments and n2: weekly rapid assessments in the labour room, antenatal clinics and sick newborn units.*

Table S7: The quality gaps identified and resolved during the intervention period for each district

| Sl no | Nature of the quality gaps | Items checked, n | Faridabad | | Rewari | | Jhajjar | |
| --- | --- | --- | --- | --- | --- | --- | --- | --- |
|  |  |  | Gaps identified, n (%)* | Gaps resolved n (%)** | Gaps identified, n (%)* | Gaps resolved n (%)** | Gaps identified, n (%)* | Gaps resolved n (%)** |
| *1* | *General Infrastructure and Systems* |  |  |  |  |  |  |  |
| 1.1 | Layout | 99 | 3 (9) | 2 (67) | 9 (27) | 4 (44) | 8 (24) | 3 (38) |
| 1.2 | Infrastructure (general) | 225 | 10 (13) | 7 (70) | 27 (36) | 6 (22) | 17 (23) | 13 (76) |
| 1.3 | Maternal care services | 117 | 7 (18) | 5 (71) | 16 (41) | 5 (31) | 15 (38) | 8 (53) |
| 1.4 | Newborn and child care | 90 | 13 (43) | 6 (46) | 18 (60) | 5 (28) | 17 (57) | 4 (24) |
| 1.5 | Staffing | 9 | 3 (100) | 0 (0) | 3 (100) | 0 (0) | 3 (100) | 0 (0) |
| 1.6 | Information & records | 99 | 4 (12) | 0 (0) | 7 (21) | 3 43) | 6 (18) | 5 (83) |
| 1.7 | Essential drugs & blood storage | 18 | 2 (33) | 0 (0) | 3 (50) | 1 (33) | 5 (83) | 1 (20) |
| 1.8 | Pharmacy and drugs | 72 | 3 (13) | 0 (0) | 6 (25) | 3 (50) | 6 (25) | 4 (67) |
| 1.9 | Laboratory services | 153 | 11 (22) | 10 (91) | 13 (25) | 4 (31) | 16 (31) | 13 (81) |
| 1.10 | Guidelines & auditing | 171 | 5 (9) | 3 (60) | 7 (12) | 4 (57) | 7 (12) | 5 (71) |
| 1.11 | Supportive Care | 108 | 8 (22) | 3 (38) | 5 (14) | 3 (60) | 10 (28) | 9 (90) |
| *2* | *Maternal Health Services* |  |  |  |  |  |  |  |
| 2.1 | Infrastructure | 171 | 18 (32) | 12 (67) | 26 (46) | 9 (35) | 22 (39) | 11 (50) |
| 2.2. | Equipment | 207 | 12 (17) | 7 (58) | 21 (30) | 10 (48) | 19 (28) | 19 (100) |
| 2.3 | Staff availability | 9 | 2 (67) | 0 (0) | 3 (100) | 0 (0) | 3 (100) | 0 (0) |
| 2.4 | Care in maternity wards | 54 | 6 (33) | 6 (100) | 5 (28) | 2 (40) | 6 (33) | 5 (83) |
| 2.5 | Case management | 414 | 7 (5) | 6 (86) | 25 (18) | 6 (24) | 23 (17) | 13 (57) |
| 2.6 | Monitoring & follow-up | 216 | 5 (7) | 4 (80) | 16 (22) | 5 (31) | 22 (31) | 22 (100) |
| 2.7 | Infection control | 108 | 5 (14) | 4 (80) | 11 (31) | 8 (73) | 5 (14) | 5 (100) |
| *3* | *Newborn Health Services* |  |  |  |  |  |  |  |
| 3.1 | Infrastructure | 297 | 29 (29) | 25 (86) | 53 (54) | 7 (13) | 39 (39) | 17 (44) |
| 3.2 | Equipment | 297 | 19 (19) | 18 (95) | 21 (21) | 1 (5) | 39 (39) | 17 (44) |
| 3.3 | Staff availability | 9 | 3 (100) | 0 (0) | 3 (100) | 0 (0) | 3 (100) | 0 (0) |
| 3.4 | Case management | 153 | 25 (49) | 23 (92) | 28 (55) | 7 (25) | 20 (39) | 7 (35) |
| 3.5 | Monitoring & follow-up | 63 | 3 (14) | 3 (100) | 13 (62) | 7 (54) | 6 (29) | 3 (50) |
| 3.6 | Newborn care at birth | 162 | 6 (11) | 5 (83) | 9 (17) | 5 (56) | 13 (24) | 7 (54) |
| *4* | *Skill of health providers* |  |  |  |  |  |  |  |
| 4.1 | Knowledge & skills-LR | 36 | 5 (40) | 2 (50) | 6 (48) | 5 (83) | 5 (42) | 3 (50) |
| 4.2 | Knowledge & skills-SNCU | 63 | 10 (46) | 4 (43) | 11 (51) | 8 (78) | 10 (46) | 5 (48) |
| *5* | *Patient satisfaction* |  |  |  |  |  |  |  |
| 5.1 | Patient satisfaction -LR | 108 | 5 (13) | 1 (31) | 10 (27) | 2.52 (26) | 8 (21) | 2 (29) |
| 5.2 | Patient satisfaction- ANC | 81 | 6 (22) | 1 (18) | 4 (13) | 0.54 (15) | 3 (12) | 1 (33) |
| 5.3 | Patient satisfaction- SNCU | 90 | 5 (16) | 2 (31) | 4 (13) | 1 (23) | 4 (13) | 0.3 (8) |
| *6* | *Clinical case record documentation* |  |  |  |  |  |  |  |
| 6.1 | Clinical case record-LR | 72 | 9 (37) | 1 (8) | 9 (37) | 3 (38) | 6 (23) | 3 (52) |
| 6.2 | Clinical case record-ANC | 63 | 8 (36) | 1 (14) | 12 (56) | 6 (54) | 8 (37) | 4 (51) |
| 6.3 | Clinical case record-SNCU | 63 | 3 (14) | 0.4 (14) | 2 (8) | 1 (88) | 6 (29) | 3 (45) |
| *7* | *Grand total* | 3972 | 268 (20) | 171 (64) | 409 (31) | 139 (34) | 393 (30) | 220 (56) |

*Note: ANC: Antenatal clinic; LR: Labour room; SNCU: Sick newborn care unit*

** The percentage estimated out of the total items checked, ** The percentage estimated out of the gaps identified*

Table S8: Key quality gaps observed at the facilities in the districts during formative research phase

| Sl.no | Areas | Activities done/Facilitated |
| --- | --- | --- |
| *1* | *Faridabad district* | |
| 1.1 | Gaps in facility assessment | - District hospital labour room: air conditioner not functional, safe drinking water not available, poor status of the toilets, bed sheet not changed daily, no specified waiting area for the family members and attendants - District hospital SNCU: heating facility for baby feed preparation not available, sitting facility for doctors/nurses not adequate, breastfeeding room not operational, problem in water supply for handwashing area and drainage challenge - FRUs: safe drinking water availability and maintenance problem, signage for various areas not available, diet for patients not regularly available, power backup challenge, no changing room for nurses |
| 1.2 | Gaps in clinical care practices | - District hospital labour room: Inadequate documentation of the clinical case records and registers, partograph documentation infrequent and incomplete, detailed delivery and birth register not used. - District hospital SNCU: Inadequate clinical case record documentation - FRU: partograph documentation infrequent and incomplete, poor counselling of mothers at discharge |
| 1.3 | Gaps in infection control | - District hospital labour room: autoclave facility and practice not organised, no routine schedule for equipment sterilization, hand washing facility in labour room not functional - District hospital SNCU: water source is common with other ward- creating challenge for uninterrupted water supply, water drainage challenge from wash basin - FRUs: equipment trays inadequate, autoclave not functional |
| 1.4 | Gaps in supervision & monitoring | - District hospital SNCU: No schedule for monitoring and supervision - FRUs: no schedule for monitoring and supervision |
| 1.5 | Gaps in skill and knowledge | - District hospital Sick newborn care unit: Inadequate knowledge and skills in essential newborn care, resuscitation, sick newborn care, waste management - FRUs: inadequate knowledge in partograph use, high risk pregnancy management, emergency obstetric case management, breastfeeding, essential newborn care, waste management, and infection control |
| *2* | *Rewari district* | |
| 2.2 | Gaps in facility assessment | - District hospital labour room: The signage and boards missing, intrapartum and postpartum case management protocols not displayed - District hospital SNCU: Crowding inside the unit for sample collection - FRUs: rregistration for antenatal check-up of low risk and high risk pregnant women same causing higher waiting time |
| 2.3 | Clinical care practices | - District hospital SNCU: inadequate documentation of case records - FRU: inadequate partograph use and APGAR recording, poor counselling at discharge, no antenatal ultrasound service |
| 2.4 | Infection control | - District hospital labour room: autoclave not functional - FRUs: autoclave not functional, inadequate segregated waste management |
| 2.5 | Supervision & Monitoring | - District hospital SNCU: no schedule for supervision and monitoring - FRUs: no schedule for supervision and monitoring |
| 2.6 | Skill building | - District hospital labour room: poor knowledge and skill on essential newborn care, resuscitation, partograph, safe child birth checklist use - District hospital SNCU: poor knowledge and skill on essential newborn care, resuscitation, breastfeeding and alternate feeding, sick newborn management, waste management - FRUs: poor knowledge and skill on partograph use, APGAR, child birth checklist, newborn resuscitation, breastfeeding, infection control |
| *3* | *Jhajjar district* | |
| 3.1 | Gaps in facility assessment | - District hospital labour room: Shortage of equipment and instrument, intrapartum and postpartum case management protocols not displayed - District hospital SNCU: ceiling full of pigeon droppings, no privacy in KMC area, poor security for SNCU and KMC area - Sub-district hospital labour room: shortage of equipment and instrument - SDH SNCU: Security guards for SNCU and KMC ward, curtains for privacy in KMC ward |
| 3.2 | Clinical care practices | - District hospital labour room: Poor partograph documentation, poor counselling at discharge - District hospital SNCU: poor and incomplete case documentation and clinical record keeping - Sub-district hospital SNCU: poor and incomplete case documentation and clinical record keeping |
| 3.3 | Infection control | - District hospital labour room: autoclave not functional, no schedule - Sub-district hospital labour room: no disinfection schedule and tracking |
| 3.4 | Supervision & Monitoring | - District hospital SNCU: no schedule for supervision and monitoring - Sub-district hospital SNCU: no schedule for supervision and monitoring |
| 3.5 | Skill building | - District hospital labour room: poor knowledge on essential newborn care, resuscitation - District hospital SNCU: poor knowledge on sick newborn care, resuscitation, breastfeeding, waste management - FRU: poor knowledge and skill of partograph use, newborn resuscitation, breastfeeding, sick newborn identification, infection control |
| *4* | *Generic observations* | |
| 4.1 | Orientation about Quality of Care | - The orientation and understanding of quality of care were limited among the health care providers across all levels, support team members and the administrators. - But most administrators and quality team members were aware of the quality improvement efforts by Government. |
| 4.2 | Willingness for Quality of Care | - Majority of the service providers (doctors and nurses) had apprehension and hesitation about the quality improvement effort. Their concerns were regarding: - The purpose, components, and activities; - Perceived that these would add to work load (documentation, processes), as there was no cross check of records, no schedule/monitoring, documentation; and - Several of the specialists/doctors had apprehension and response that “it cannot be done”; - The FRU teams had enthusiasm, as they expected to gain in knowledge and practice skills through the quality improvement process. |
| 4.3 | Willingness of the administrator | - Willingness of the administrator for quality improvement was evident. - They were willing to facilitate and support the Quality Improvement team for the implementation of desired activities. - They were open to receive the findings and expressed willingness to address the gaps as per the administrative and financial procedures. |
| 4.4 | Administrative challenges | - - Some administrative and staff related conflicts between health services and National Health Mission for SNCU was observed.   - Poor timeline for payment to vendors for services/repair etc., which challenged the regular service availability.   - On the face “no shortage of funds” but release of fund and related procedures were challenge for the service providers. |
| 4.5 | Counselling of patients | - Counselling in the postnatal wards, at discharge and antenatal clinics was a challenge as no dedicated person was allocated and no protocol followed. |
| 4.6 | Manpower shortage | - Manpower shortage was universal and critical constraint. - Relocation of staffs on adhoc basis between departments and units were being done to meet the challenges. - Several staffs were contractual in nature. |

*Note: SNCU: Sick newborn care unit; FRU: First referral unit; KMC: Kangaroo mother care; APGAR: appearance (colour), pulse (heart rate), grimace (reflexes), activity (muscle tone), and respiration*

Table S9: Changes in the infrastructure, manpower and processes at the hospitals in the three districts

| Sl. No | Health services components | Faridabad | | | | | | | | Rewari | | | | | | | | Jhajjar | | | | | | | |
| --- | --- | --- | --- | --- | --- | --- | --- | --- | --- | --- | --- | --- | --- | --- | --- | --- | --- | --- | --- | --- | --- | --- | --- | --- | --- |
|  |  | District hospital | | FRU-1 | | FRU-2 | | District Pooled | | District hospital | | FRU 1 | | FRU 2 | | District Pooled | | District hospital | | FRU1/SDH | | FRU 2 | | District Pooled | |
|  |  | Base-line | End-line | Base-line | End-line | Base-line | End-line | Base-line | End-line | Base-line | End-line | Base-line | End-line | Base-line | End-line | Base-line | End-line | Base-line | End-line | Base-line | End-line | Base-line | End-line | Base-line | End-line |
| *A* | *General Health Services, n* | 12 | 12 | 12 | 12 | 12 | 12 | 12 | 12 | 12 | 12 | 12 | 12 | 12 | 12 | 12 | 12 | 12 | 12 | 12 | 12 | 12 | 12 | 12 | 12 |
| A1 | Layout (%) | 100 | 100 | 82 | 91 | 91 | 91 | 91 | 94 | 91 | 91 | 73 | 82 | 73 | 82 | 79 | 85 | 100 | 100 | 100 | 100 | 25 | 50 | 75 | 83 |
| A2 | Infrastructure (general) (%) | 86 | 89 | 96 | 100 | 92 | 100 | 91 | 96 | 86 | 86 | 68 | 71 | 64 | 71 | 73 | 76 | 92 | 96 | 75 | 96 | 50 | 90 | 72 | 94 |
| A3 | Maternal care services (%) | 85 | 92 | 85 | 92 | 92 | 100 | 87 | 95 | 69 | 85 | 38 | 38 | 54 | 78 | 54 | 67 | 77 | 92 | 77 | 100 | 46 | 62 | 67 | 85 |
| A4 | Newborn & child care (%) | 70 | 80 | 50 | 70 | 63 | 100 | 61 | 83 | 50 | 80 | 35 | 50 | 20 | 45 | 35 | 58 | 60 | 90 | 70 | 80 | NA | NA | 43 | 57 |
| A5 | Staffing (%) | 56 | 67 | 89 | 89 | 84 | 63 | 76 | 73 | 66 | 78 | 78 | 63 | 72 | 86 | 72 | 76 | 87 | 87 | 84 | 84 | 35 | 50 | 69 | 74 |
| A6 | Information & records (%) | 100 | 100 | 82 | 82 | 82 | 82 | 88 | 88 | 100 | 100 | 73 | 88 | 65 | 73 | 79 | 87 | 82 | 100 | 100 | 100 | 64 | 100 | 82 | 100 |
| A7 | Blood bank/storage (%) | 100 | 100 | 0 | 0 | 0 | 0 | 33 | 33 | 75 | 100 | 0 | 0 | 0 | 0 | 25 | 33 | 75 | 100 | 25 | 25 | 0 | 0 | 33 | 42 |
| A8 | Pharmacy (%) | 74 | 95 | 94 | 95 | 74 | 91 | 81 | 94 | 86 | 92 | 86 | 95 | 77 | 84 | 83 | 90 | 86 | 92 | 86 | 95 | 77 | 84 | 83 | 90 |
| A9 | Laboratory services (%) | 90 | 100 | 71 | 100 | 76 | 100 | 79 | 100 | 72 | 80 | 68 | 88 | 50 | 67 | 63 | 78 | 71 | 94 | 82 | 100 | 71 | 88 | 75 | 94 |
| A10 | Guidelines and auditing (%) | 95 | 100 | 100 | 100 | 95 | 100 | 97 | 100 | 95 | 100 | 50 | 75 | 50 | 75 | 65 | 83 | 100 | 100 | 100 | 100 | 63 | 91 | 88 | 97 |
| A11 | Supportive care (%) | 83 | 100 | 67 | 75 | 75 | 75 | 75 | 83 | 83 | 100 | 50 | 75 | 50 | 75 | 61 | 83 | 100 | 100 | 67 | 100 | 83 | 89 | 83 | 96 |
|  | Sub-total general health | 85 | 93 | 74 | 81 | 75 | 82 | 78 | 85 | 79 | 90 | 56 | 66 | 52 | 67 | 63 | 74 | 85 | 96 | 79 | 89 | 47 | 64 | 70 | 83 |
| *B* | *Maternal Health Services, n* | 12 | 12 | 12 | 12 | 12 | 12 | 12 | 12 | 12 | 12 | 12 | 12 | 12 | 12 | 12 | 12 | 12 | 12 | 12 | 12 | 12 | 12 | 12 | 12 |
| B1 | Infrastructure (%) | 84 | 95 | 82 | 88 | 71 | 94 | 79 | 92 | 68 | 79 | 47 | 79 | 58 | 74 | 58 | 77 | 68 | 95 | 68 | 95 | 42 | 58 | 59 | 83 |
| B2 | Equipment (%) | 83 | 91 | 65 | 95 | 74 | 87 | 74 | 91 | 78 | 94 | 91 | 100 | 52 | 61 | 74 | 85 | 75 | 100 | 91 | 100 | 75 | 100 | 80 | 100 |
| B3 | Staff availability (%) | 81 | 74 | 75 | 75 | 89 | 89 | 82 | 79 | 76 | 86 | 78 | 80 | 72 | 73 | 75 | 80 | 98 | 98 | 86 | 88 | 45 | 66 | 76 | 84 |
| B4 | Care in maternity wards (%) | 50 | 100 | 83 | 100 | 83 | 100 | 72 | 100 | 67 | 89 | 50 | 92 | 50 | 89 | 56 | 90 | 33 | 100 | 100 | 100 | 50 | 65 | 61 | 88 |
| B5 | Case management (%) | 100 | 100 | 96 | 98 | 96 | 98 | 97 | 99 | 91 | 92 | 95 | 100 | 59 | 70 | 82 | 87 | 100 | 75 | 100 | 100 | 100 | 100 | 100 | 92 |
| B6 | Monitoring & follow-up (%) | 100 | 100 | 96 | 100 | 92 | 100 | 96 | 100 | 96 | 94 | 79 | 79 | 71 | 82 | 82 | 85 | 83 | 100 | 60 | 100 | 63 | 100 | 69 | 100 |
| B7 | Infection control | 83 | 100 | 92 | 100 | 83 | 100 | 86 | 100 | 92 | 100 | 83 | 100 | 42 | 88 | 72 | 96 | 100 | 100 | 92 | 100 | 100 | 100 | 97 | 100 |
|  | Sub-total maternal health | 83 | 94 | 84 | 94 | 84 | 95 | 84 | 94 | 81 | 91 | 75 | 90 | 58 | 77 | 71 | 86 | 80 | 95 | 85 | 98 | 68 | 84 | 78 | 92 |
| *C* | *Newborn Health Services, n* | 12 | 12 | 12 | 12 | 12 | 12 | 12 | 12 | 12 | 12 | 12 | 12 | 12 | 12 | 12 | 12 | 12 | 12 | 12 | 12 | 12 | 12 | 12 | 12 |
| C1 | Infrastructure (%) | 88 | 100 | 73 | 100 | 70 | 100 | 77 | 100 | 73 | 85 | 45 | 58 | 30 | 30 | 49 | 58 | 67 | 100 | 94 | 100 | 0 | 0 | 64 | 77 |
| C2 | Equipment (%) | 100 | 100 | 85 | 97 | 75 | 100 | 87 | 99 | 94 | 94 | 79 | 79 | 67 | 67 | 80 | 80 | 75 | 100 | 91 | 100 | 0 | 0 | 66 | 78 |
| C3 | Staff availability (%) | 68 | 91 | 0 | 0 | 0 | 0 | 23 | 30 | 66 | 86 | 0 | 0 | 0 | 0 | 22 | 29 | 50 | 83 | 86 | 86 | 0 | 0 | 45 | 56 |
| C4 | Case management (%) | 94 | 100 | 82 | 100 | 71 | 94 | 82 | 98 | 94 | 100 | 57 | 51 | 22 | 22 | 58 | 58 | 67 | 100 | 100 | 100 | 0 | 0 | 63 | 74 |
| C5 | Monitoring & follow-up (%) | 100 | 100 | 100 | 100 | 71 | 100 | 90 | 100 | 68 | 100 | 51 | 52 | 28 | 56 | 49 | 69 | 100 | 100 | 100 | 100 | 0 | 0 | 76 | 78 |
| C6 | Newborn care at birth (%) | 100 | 100 | 83 | 100 | 89 | 100 | 91 | 100 | 83 | 100 | 94 | 100 | 78 | 78 | 85 | 93 | 83 | 100 | 94 | 100 | 0 | 0 | 67 | 83 |
|  | Sub-total newborn health | 92 | 99 | 71 | 83 | 63 | 82 | 75 | 88 | 80 | 94 | 54 | 57 | 38 | 42 | 57 | 64 | 74 | 97 | 94 | 98 | 23 | 28 | 64 | 74 |

*Note: FRU: First referral unit SDH: Sub-district hospital*

Table S10: Changes in the quality of case record documentation at the hospitals in the three districts

| Sl  no | Components assessed | Faridabad | | | | | | | | Rewari | | | | | | | | Jhajjar | | | | | | | |
| --- | --- | --- | --- | --- | --- | --- | --- | --- | --- | --- | --- | --- | --- | --- | --- | --- | --- | --- | --- | --- | --- | --- | --- | --- | --- |
|  |  | District hospital | | FRU-1 | | FRU-2 | | District Pooled | | District hospital | | FRU 1 | | FRU 2 | | District Pooled | | District hospital | | FRU1/SDH | | FRU 2 | | District Pooled | |
|  |  | Base-line | End-line | Base-line | End-line | Base-line | End-line | Base-line | End-line | Base-line | End-line | Base-line | End-line | Base-line | End-line | Base-line | End-line | Base-line | End-line | Base-line | End-line | Base-line | End-line | Base-line | End-line |
| *1* | Labour room and postnatal wards, n | 169 | 178 | 79 | 97 | 79 | 56 | 327 | 361 | 210 | 234 | 79 | 111 | 75 | 81 | 364 | 426 | 289 | 366 | 79 | 75 | 57 | 57 | 425 | 498 |
| 1.1 | General information (%) | 100 | 100 | 100 | 100 | 100 | 100 | 100 | 100 | 100 | 100 | 99 | 100 | 100 | 100 | 100 | 100 | 100 | 100 | 100 | 100 | 100 | 100 | 100 | 100 |
| 1.2 | Pregnancy history (%) | 76 | 85 | 89 | **95** | 84 | **98** | 83 | **93** | 76 | **85** | 65 | **92** | 72 | **95** | 71 | **91** | 95 | 99 | 87 | **93** | 80 | **93** | 87 | **95** |
| 1.3 | Vitals (%) | 67 | **99** | 83 | **98** | 65 | **99** | 72 | **99** | 67 | **99** | 67 | **80** | 65 | **98** | 66 | **92** | 93 | **99** | 84 | **90** | 64 | **90** | 80 | **93** |
| 1.4 | Maternal history (%) | 51 | **99** | 24 | **100** | 40 | **100** | 38 | **100** | 31 | **99** | 45 | **55** | 31 | **85** | 36 | **80** | 89 | 91 | 48 | **78** | 64 | **95** | 67 | **88** |
| 1.5 | Labour and delivery (%) | 75 | **87** | 83 | **94** | 71 | **100** | 76 | **94** | 75 | **87** | 88 | **99** | 88 | **91** | 84 | **92** | 90 | **100** | 90 | 95 | 70 | **90** | 83 | **95** |
| 1.6 | Details of baby (%) | 28 | **99** | 25 | **99** | 20 | **99** | 24 | **99** | 28 | **99** | 41 | **50** | 27 | **76** | 32 | **75** | 67 | **75** | 47 | **75** | 77 | **98** | 64 | **83** |
| 1.7 | Hospital course (%) | 40 | **96** | 57 | **97** | 38 | **74** | 45 | **89** | 40 | **96** | 48 | **51** | 54 | **76** | 47 | **74** | 73 | 78 | 65 | **82** | 79 | 80 | 72 | **80** |
| 1.8 | Discharge/ Referral (%) | 68 | **100** | 63 | **100** | 54 | **100** | 62 | **100** | 68 | **100** | 59 | **75** | 77 | **82** | 68 | **86** | 50 | **75** | 74 | 74 | 50 | **70** | 58 | **73** |
|  | Pooled- labour room and postnatal ward (%) | 63 | **96** | 66 | **98** | 59 | **96** | 63 | **97** | 61 | **96** | 64 | **75** | 64 | **88** | 63 | **86** | 82 | **90** | 74 | **86** | 73 | **90** | 76 | **89** |
| ***2*** | Antenatal check-up, n | 113 | 116 | 41 | 39 | 39 | 38 | 193 | 193 | 178 | 183 | 91 | 84 | 24 | 24 | 293 | 291 | 172 | 180 | 48 | 43 | 12 | 12 | 232 | 235 |
| 2.1 | Age of patient (%) | 100 | 100 | 100 | 100 | 100 | 100 | 100 | 100 | 100 | 100 | 100 | 100 | 100 | 100 | 100 | 100 | 100 | 100 | 100 | 100 | 100 | 100 | 100 | 100 |
| 2.2 | Pregnancy history (%) | 94 | 99 | 70 | **100** | 87 | **100** | 84 | **100** | 73 | 85 | 26 | **66** | 33 | **69** | 44 | **73** | 93 | 97 | 90 | 96 | 100 | 100 | 94 | 98 |
| 2.3 | Vitals (%) | 22 | **100** | 50 | **100** | 44 | **100** | 39 | **100** | 29 | **70** | 38 | **50** | 25 | **40** | 31 | **53** | 38 | **62** | 75 | **84** | 40 | **56** | 51 | **67** |
| 2.4 | Abdomen examination (%) | 56 | **100** | 67 | **100** | 38 | **100** | 54 | **100** | 25 | **60** | 12 | **45** | 32 | **64** | 23 | **56** | 25 | **60** | 20 | **57** | 25 | **45** | 23 | **54** |
| 2.5 | Investigation (%) | 62 | **100** | 64 | **100** | 44 | **100** | 57 | **100** | 61 | **76** | 61 | **79** | 39 | **77** | 54 | **77** | 86 | **95** | 65 | **82** | 96 | **94** | 82 | **90** |
| 2.6 | Medication (%) | 62 | **98** | 98 | 100 | 95 | 100 | 85 | **99** | 53 | **78** | 42 | **75** | 38 | **96** | 44 | **83** | 85 | **95** | 50 | **98** | 75 | **90** | 70 | **94** |
| 2.7 | Counselling (%) | 11 | **65** | 40 | **67** | 40 | **67** | 30 | **66** | 10 | **45** | 20 | **35** | 0 | **50** | 10 | **43** | 17 | **73** | 0 | **54** | 50 | **60** | 22 | **62** |
|  | Pooled- Antenatal check-up (%) | 58 | **95** | 70 | **95** | 64 | **95** | 64 | **95** | 50 | **73** | 43 | **64** | 38 | **71** | 44 | **69** | 63 | **83** | 57 | **82** | 69 | **78** | 63 | **81** |
| *3* | Sick newborn care unit, n | 237 | 255 | - | - | - | - | 237 | 255 | 257 | 245 | - | - | - | - | 257 | 245 | 174 | 159 | 122 | 125 | - | - | 296 | 284 |
| 3.1 | General information (%) | 92 | **100** | - | - | - | - | 92 | 100 | 91 | 95 | - | - | - | - | 91 | 95 | 99 | 100 | 85 | 98 | - | - | 92 | 99 |
| 3.2 | Admission/discharge information (%) | 94 | 98 | - | - | - | - | 94 | 98 | 99 | 99 | - | - | - | - | 99 | 99 | 97 | 94 | 81 | 93 | - | - | 89 | 94 |
| 3.3 | Delivery information (%) | 89 | 90 | - | - | - | - | 89 | 90 | 28 | **75** | - | - | - | - | 28 | **75** | 87 | 95 | 40 | **77** | - | - | 64 | **86** |
| 3.4 | History (%) | 27 | **94** | - | - | - | - | 27 | **94** | 58 | **85** | - | - | - | - | 58 | **85** | 84 | **94** | 12 | **44** | - | - | 48 | **69** |
| 3.5 | Examination and clinical details (%) | 58 | **100** | - | - | - | - | 58 | **100** | 66 | **88** | - | - | - | - | 66 | **88** | 85 | **95** | 37 | **64** | - | - | 61 | **80** |
| 3.6 | Hospital course (%) | 97 | **100** | - | - | - | - | 97 | 100 | 96 | 93 | - | - | - | - | 96 | 93 | 94 | **100** | 80 | 91 | - | - | 87 | **96** |
| 3.7 | Discharge/Referral information (%) | 86 | **98** | - | - | - | - | 86 | **98** | 92 | 93 | - | - | - | - | 92 | 93 | 64 | **84** | 51 | **84** | - | - | 58 | **84** |
|  | Pooled- Sick newborn care unit (%) | 78 | **97** | - | - | - | - | 78 | **97** | 76 | **90** | - | - | - | - | 76 | **90** | 87 | **95** | 55 | **79** | - | - | 71 | **87** |

*Note: The figures in bold indicate the change is statistically significant (p <0.05); FRU: First referral unit; SDH: Sub-district hospital*

Table S11: Changes in the patient satisfaction status at the hospitals in the three districts

| Sl. No | Components assessed | Faridabad | | | | | | | | Rewari | | | | | | | | Jhajjar | | | | | | | |
| --- | --- | --- | --- | --- | --- | --- | --- | --- | --- | --- | --- | --- | --- | --- | --- | --- | --- | --- | --- | --- | --- | --- | --- | --- | --- |
|  |  | District hospital | | FRU-1 | | FRU-2 | | Pooled | | District hospital | | FRU-1 | | FRU-2 | | Pooled | | District hospital | | FRU-1/SDH | | FRU-2 | | Pooled | |
|  |  | Base-line | End-line | Base-line | End-line | Base-line | End-line | Base-line | End-line | Base-line | End-line | Base-line | End-line | Base-line | End-line | Base-line | End-line | Base-line | End-line | Base-line | End-line | Base-line | End-line | Base-line | End-line |
| *1* | *Labour rooms (LR) and postnatal wards, n* | 133 | 123 | 15 | 38 | 16 | 17 | 164 | 178 | 149 | 133 | 67 | 123 | 14 | 13 | 230 | 269 | 94 | 84 | 59 | 66 | 14 | 29 | 167 | 179 |
| 1.1 | Assistance in registration (%) | 93 | 96 | 80 | **100** | 100 | 100 | 91 | 99 | 93 | 100 | 70 | **95** | 72 | **90** | 78 | **95** | 87 | **100** | 100 | 100 | 100 | 100 | 96 | 100 |
| 1.2 | Accessibility (%) | 90 | 91 | 87 | **100** | 100 | 100 | 92 | 97 | 90 | **99** | 65 | **100** | 52 | **95** | 69 | **98** | 98 | 100 | 85 | **100** | 100 | 100 | 94 | 100 |
| 1.3 | Admission process (%) | 81 | **100** | 60 | **100** | 100 | 100 | 80 | **100** | 72 | **100** | 73 | **95** | 75 | **85** | 73 | **93** | 86 | **95** | 85 | **97** | 85 | 90 | 85 | **94** |
| 1.4 | Wait for nurse/doctor (%) | 81 | **100** | 33 | **100** | 100 | 100 | 71 | **100** | 57 | **83** | 85 | **100** | 80 | **92** | 74 | **92** | 88 | **96** | 67 | **90** | 75 | **90** | 77 | **92** |
| 1.5 | Comfort Level (%) | 85 | **98** | 84 | **99** | 100 | 100 | 90 | **99** | 94 | 97 | 69 | **97** | 71 | **94** | 78 | **96** | 87 | **97** | 81 | **90** | 40 | **70** | 69 | **86** |
| 1.6 | Care after delivery (%) | 77 | **88** | 78 | **94** | 75 | **85** | 77 | **89** | 96 | 100 | 68 | **100** | 69 | **85** | 78 | **95** | 95 | 100 | 80 | **98** | 80 | **90** | 85 | **96** |
| 1.7 | Behavior of staffs (%) | 94 | 96 | 93 | 100 | 100 | 100 | 96 | 99 | 86 | **96** | 55 | **100** | 50 | **89** | 64 | **95** | 90 | **99** | 96 | 100 | 90 | **100** | 92 | 100 |
| 1.8 | Cleanliness (%) | 60 | **84** | 65 | **75** | 48 | **75** | 58 | **78** | 94 | 99 | 83 | **100** | 50 | **95** | 76 | **98** | 50 | **69** | 100 | 100 | 25 | **100** | 58 | **90** |
| 1.9 | Diet supplied (%) | 99 | 100 | 93 | **100** | 100 | 100 | 97 | 100 | 51 | **80** | 49 | **50** | 50 | 50 | 50 | **60** | 73 | **100** | 33 | **95** | 65 | **100** | 57 | **98** |
| 1.10 | Other facilities (%) | 96 | 91 | 96 | 91 | 100 | 100 | 97 | 94 | 70 | **95** | 65 | **100** | 74 | **87** | 70 | **94** | 48 | **61** | 48 | **55** | 50 | **90** | 49 | **69** |
| 1.11 | No payment made (%) | 100 | 100 | 100 | 100 | 100 | 100 | 100 | 100 | 96 | 96 | 78 | **100** | 100 | 100 | 91 | 99 | 100 | 100 | 100 | 100 | 100 | 100 | 100 | 100 |
| 1.12 | Discharge process (%) | 87 | **96** | 77 | **100** | 100 | 100 | 88 | **99** | 72 | **90** | 72 | **100** | 69 | **90** | 71 | **93** | 92 | **100** | 99 | 100 | 50 | **95** | 80 | **98** |
|  | Pooled (%) | 87 | **95** | 79 | **97** | 94 | 97 | 87 | **96** | 81 | **95** | 69 | **95** | 68 | **88** | 73 | **93** | 83 | **93** | 81 | **94** | 72 | **94** | 79 | **94** |
| ***2*** | *Antenatal clinics, n* | 122 | 158 | 77 | 69 | 48 | 44 | 247 | 271 | 149 | 133 | 108 | 93 | 29 | 13 | 286 | 269 | 84 | 94 | 49 | 56 | 14 | 19 | 147 | 169 |
| 2.1 | Accessibility (%) | 65 | **95** | 64 | **98** | 91 | **100** | 73 | **98** | 83 | **91** | 100 | 100 | 98 | 99 | 94 | 97 | 95 | 97 | 96 | 98 | 98 | 99 | 96 | 98 |
| 2.2 | Time taken (%) | 40 | **69** | 85 | 90 | 41 | **92** | 55 | **84** | 75 | **85** | 99 | 100 | 100 | 100 | 91 | 95 | 51 | **67** | 50 | **70** | 100 | 100 | 67 | **79** |
| 2.3 | Comfortable (%) | 81 | **99** | 70 | **100** | 75 | **100** | 75 | **100** | 97 | 100 | 100 | 100 | 96 | 100 | 98 | 100 | 94 | 100 | 100 | 100 | 96 | 100 | 97 | 100 |
| 2.4 | Staff attitude (%) | 82 | **99** | 67 | **100** | 99 | 100 | 83 | **100** | 82 | **92** | 88 | **95** | 99 | 100 | 90 | 96 | 95 | 100 | 99 | 100 | 99 | 100 | 98 | 100 |
| 2.5 | General cleanliness (%) | 62 | 62 | 70 | **100** | 82 | **100** | 71 | **87** | 83 | **100** | 74 | **100** | 86 | **94** | 81 | **98** | 87 | **100** | 100 | 100 | 86 | **94** | 91 | 98 |
| 2.6 | Toilet cleanliness (%) | 65 | **87** | 79 | **100** | 80 | **100** | 75 | **96** | 44 | **95** | 50 | **100** | 45 | **87** | 46 | **94** | 44 | **100** | 85 | **92** | 45 | **87** | 58 | **93** |
| 2.7 | Other facilities (%) | 99 | 99 | 89 | **100** | 66 | **100** | 85 | **100** | 74 | **100** | 91 | **100** | 100 | 100 | 88 | **100** | 86 | **100** | 100 | 100 | 100 | 100 | 95 | 100 |
| 2.8 | No payment made (%) | 99 | 100 | 100 | 100 | 100 | 100 | 100 | 100 | 100 | 100 | 100 | 100 | 100 | 100 | 100 | 100 | 100 | 100 | 100 | 100 | 100 | 100 | 100 | 100 |
| 2.9 | HCP’s care and response (%) | 59 | **100** | 91 | **100** | 100 | 100 | 83 | **100** | 97 | 100 | 100 | 100 | 78 | 100 | 92 | 100 | 99 | 99 | 99 | 99 | 90 | 90 | 96 | 96 |
|  | Pooled (%) | 72 | **90** | 79 | **99** | 82 | **99** | 78 | **96** | 82 | **96** | 89 | **99** | 89 | **98** | 87 | **98** | 83 | **96** | 92 | 95 | 90 | **97** | 88 | **96** |
| *3* | *Sick newborn care (SNCU), n* | 70 | 84 |  |  |  |  | 70 | 84 | 73 | 82 |  |  |  |  | 73 | 82 | 39 | 34 | 20 | 47 |  |  | 59 | 81 |
| 3.1 | Accessibility (%) | 62 | **94** |  |  |  |  | 62 | **94** | 88 | 89 |  |  |  |  | 88 | **89** | 97 | 100 | 71 | **99** |  |  | 84 | **100** |
| 3.2 | Response at admission (%) | 96 | 100 |  |  |  |  | 96 | 100 | 88 | **98** |  |  |  |  | 88 | **98** | 92 | **100** | 95 | 97 |  |  | 94 | 99 |
| 3.3 | Care in SNCU (%) | 88 | **98** |  |  |  |  | 88 | **98** | 90 | 90 |  |  |  |  | 90 | 90 | 56 | **100** | 95 | 96 |  |  | 76 | **98** |
| 3.4 | General cleanliness (%) | 59 | **81** |  |  |  |  | 59 | **81** | 60 | 100 |  |  |  |  | 60 | **100** | 64 | **100** | 100 | 100 |  |  | 82 | **100** |
| 3.5 | Drinking water (%) | 84 | **94** |  |  |  |  | 84 | **94** | 100 | 100 |  |  |  |  | 100 | 100 | 97 | 100 | 75 | **99** |  |  | 86 | **100** |
| 3.6 | Clean toilets (%) | 90 | **100** |  |  |  |  | 90 | **100** | 100 | 100 |  |  |  |  | 100 | 100 | 85 | **100** | 100 | 100 |  |  | 93 | 100 |
| 3.7 | Shelter for caretakers (%) | 75 | **87** |  |  |  |  | 75 | **87** | 43 | **88** |  |  |  |  | 43 | **88** | 96 | 100 | 100 | 100 |  |  | 98 | 100 |
| 3.8 | No payment made (%) | 98 | 98 |  |  |  |  | 98 | 98 | 100 | 100 |  |  |  |  | 100 | 100 | 71 | **100** | 96 | 100 |  |  | 84 | **100** |
| 3.9 | Services by doctors (%) | 93 | **100** |  |  |  |  | 93 | 100 | 100 | 100 |  |  |  |  | 100 | 100 | 98 | 98 | 100 | 100 |  |  | 99 | 99 |
| 3.10 | Discharge process (%) | 97 | 100 |  |  |  |  | 97 | 100 | 97 | 100 |  |  |  |  | 97 | 100 | 77 | 82 | 67 | **100** |  |  | 72 | **91** |
|  | Pooled (%) | 84 | **95** |  |  |  |  | 84 | **95** | 87 | **97** |  |  |  |  | 87 | **97** | 83 | **98** | 90 | **99** |  |  | 87 | **99** |

*Note: The figures in bold indicate the change is statistically significant (p <0.05); FRU: First referral unit; SDH: Sub-district hospital; SNCU: Sick newborn care unit*

Table S12: Time spent (median and IQR in minutes) in minutes by pregnant women in antenatal clinics in Faridabad district

| Parameters | Faridabad | | | | Rewari | | | | Jhajjar | | | |
| --- | --- | --- | --- | --- | --- | --- | --- | --- | --- | --- | --- | --- |
|  | Non-high risk pregnant women  median time (IQR) | | High risk pregnant women  median time (IQR) | | Non-high risk pregnant women  median time (IQR) | | High risk pregnant women  median time (IQR) | | Non-high risk pregnant women  median time (IQR) | | High risk pregnant women  median time (IQR) | |
|  | Baseline | Endline | Baseline | Endline | Baseline | Endline | Baseline | Endline | Baseline | Endline | Baseline | Endline |
| 1. District hospital, observations, n | 57 | 22 | 18 | 15 | 26 | 15 | 18 | 13 | 24 | 14 | 38 | 25 |
| 1.1 Time for registration (minutes) | 24  (14-47) | 15  (12-18)† | 31  (16-39) | 17  (14-19)† | 1  (1-1) | 1  (1-2) | 1  (1-2) | 1  (1-1) | 10  (5-20) | 6  (5-7)† | 5  (5-16) | 6  (5-7)* |
| 1.2 Time to 1st contact-Nurse (minutes) | 87  (59-157) | 34  (29-39)† | 104  (84-148) | 48  (32-55)† | 3.5  (2-11) | 12  (12-14)† | 10  (4-10) | 8  (3-61)† | 7  (5-10) | 6  (5-8)* | 4.5  (3-6) | 4  (3.5-6) |
| 1.3. Time to 1st contact-Doctor (minutes) | 4  (3-6) | 9  (4-13)† | 3  (2-5) | 8  (7-12)† | 3  (2-4) | 10  (6-15)† | 2  (2-2) | 4  (1-6)† | 15  (8-22) | 14  (8-20) | 14.5  (8-19) | 7  (2.5-14)† |
| 1.4. Total time taken (minutes) | 142  (87-198) | 100  (76-113)† | 126  (101-162) | 107  (67-120)† | 107  (65-211) | 74  (70-171)* | 150  (111-245) | 70  (60-111)† | 50  (35-74) | 78  (62-110)† | 41  (30-51) | 33  (21-46)† |
| 2. FRU 1, observations, n | 16 | 16 | 8 | 19 | 15 | 13 | 6 | 10 | 18 | 14 | 12 | 17 |
| 2.1 Time for registration (minutes) | 18 (5-22) | 16 (15-19) | 9.5 (6-23) | 12 (10-14) | 2 (1-2) | 2 (1-3) | 2 (2-2) | 2 (2-2) | 10 (5-15) | 5 (0-12)† | 0 | 0 |
| 2.2. Time to 1st contact-Nurse (minutes) | 68  (42-93) | 50  (46-59)† | 18  (11.5-54) | 34  (33-46)† | 6  (2-15) | 6  (3-12) | 0 | 6  (5-7) | 8  (4-15) | 7  (4-15) | 2  (1-2.5) | 5  (5-7.5)† |
| 2.3. Time to 1st contact-Doctor (minutes) | 2  (2-3) | 3  (3-5) | 3.5  (2-4.5) | 3  (2-4) | 9.5  (3-75) | 16  (6-28) | 52  (38-55) | 8  (6-17)† | 9  (9-11) | 14  (12-19)† | 11  (10-13) | 40  (15-80)† |
| 2.4. Total time taken (minutes) | 95  (52-145) | 72  (64-86)† | 31  (22-70) | 53  (44-58)† | 101  (64-181) | 123  (123-183)* | 85  (80-90) | 32  (26-43)† | 62  (38-102) | 92  (75-137)† | 73  (40-121) | 125  (90-155)† |
| 3. FRU 2, observations, n | 11 | 17 | 5 | 16 | 6 | 10 | 12 | 11 | 14 | 15 | 11 | 13 |
| - 1. Time for registration (minutes) | 4 (3-15) | 16 (10-20)† | 13 (10-16) | 8 (5-10)† | 1.5 (1-2) | 2 (1-3) | 2 (1-4) | 2 (1-3) | 0 (0-30) | 3 (2-5)† | 10 (7-15) | 11 (9-14) |
| 3.2. Time to 1st contact-Nurse(minutes) | 40  (18-70) | 35  (29-37)* | 73  (65-83) | 22  (11-38)† | 5  (3-10) | 10.5  (4-17)* | 5  (2-8) | 7  (5-9)† | 20  (5-35) | 5  (3-9)† | 5  (3-7) | 2  (1-3)† |
| 3.3. Time to 1st contact-Doctor (minutes) | 3  (2-5) | 5  (3-7)† | 12  (11-13) | 7.5  (6-8)† | 5.5  (2-49) | 4  (2-8)* | 55  (42-62) | 9  (6-11)† | 10  (8-11) | 20  (12-32)† | 10  (8-12) | 3  (2-4)† |
| 3.4. Total time taken (minutes) | 79  (43-88) | 56  (46-61) † | 123  (113-133) | 52  (34-63)† | 87  (60-101) | 60  (30-96)* | 90  (70-112) | 48  (34-60)† | 135  (70-195) | 40  (25-80)† | 75  (55-90) | 72  (62-82) |

*Notes: * Indicate the change is statistically significant (p<0.05);* † *Indicate the change is statistically significant (p<0.01)*

Table S13: Change in knowledge and skill status of the care providers in labour room and sick newborn care units

| Sl.no | Domains | Faridabad | | Rewari | | Jhajjar | | Pooled | |
| --- | --- | --- | --- | --- | --- | --- | --- | --- | --- |
|  | Cycle | Baseline | Endline | Baseline | Endline | Baseline | Endline | Baseline | Endline |
| *1* | *Labour rooms and postnatal wards*  *(Delivery and essential newborn care)* |  |  |  |  |  |  |  |  |
|  | Respondents (n) | 22 | 24 | 20 | 22 | 22 | 24 | 64 | 70 |
| 1.1 | Delivery (%) | 48 | 71 | 34 | 40 | 46 | 71 | 43 | 61 |
| 1.2 | Essential newborn care (%) | 77 | 92 | 72 | 75 | 86 | 90 | 78 | 86 |
| 1.3 | Resuscitation (%) | 48 | 71 | 42 | 63 | 48 | 77 | 46 | 70 |
| 1.4 | Infection control (%) | 68 | 86 | 60 | 63 | 53 | 78 | 60 | 76 |
| 1.5 | Sub-total delivery and essential newborn care (%) | 60 | 80 | 52 | 60 | 58 | 79 | 57 | 73 |
| *2* | *Sick newborn care units*  *(essential and sick newborn care))* |  |  |  |  |  |  |  |  |
|  | Respondents (n) | 8 | 8 | 8 | 8 | 12 | 12 | 28 | 28 |
| 2.1 | Supportive care (%) | 58 | 79 | 54 | 65 | 65 | 74 | 59 | 73 |
| 2.2 | Temperature (%) | 40 | 74 | 23 | 34 | 43 | 65 | 35 | 58 |
| 2.3 | Infection control (%) | 83 | 90 | 59 | 68 | 78 | 95 | 73 | 84 |
| 2.4 | Resuscitation (%) | 39 | 83 | 25 | 33 | 36 | 67 | 33 | 61 |
| 2.5 | Feeding (%) | 52 | 74 | 58 | 61 | 39 | 81 | 50 | 72 |
| 2.6 | Case management (%) | 63 | 82 | 38 | 50 | 66 | 86 | 56 | 73 |
| 2.7 | Intravenous fluid and medication (%) | 42 | 76 | 88 | 90 | 52 | 81 | 61 | 82 |
| 2.8 | Sub-total essential and sick newborn care (%) | 54 | 80 | 49 | 57 | 54 | 78 | 52 | 72 |
